# Phylogenetic analysis of CDK and cyclin proteins in premetazoan lineages

**DOI:** 10.1186/1471-2148-14-10

**Published:** 2014-01-17

**Authors:** Lihuan Cao, Fang Chen, Xianmei Yang, Weijin Xu, Jun Xie, Long Yu

**Affiliations:** 1 State Key Laboratory of Genetic Engineering, Institute of Genetics, School of Life Sciences, Fudan University, Shanghai 200433, PR China

**Keywords:** Cell cycle, Evolution, Metazoan emergence, CDK, Cyclin

## Abstract

**Background:**

The molecular history of animal evolution from single-celled ancestors remains a major question in biology, and little is known regarding the evolution of cell cycle regulation during animal emergence. In this study, we conducted a comprehensive evolutionary analysis of CDK and cyclin proteins in metazoans and their unicellular relatives.

**Results:**

Our analysis divided the CDK family into eight subfamilies. Seven subfamilies (CDK1/2/3, CDK5, CDK7, CDK 20, CDK8/19, CDK9, and CDK10/11) are conserved in metazoans and fungi, with the remaining subfamily, CDK4/6, found only in eumetazoans. With respect to cyclins, cyclin C, H, L, Y subfamilies, and cyclin K and T as a whole subfamily, are generally conserved in animal, fungi, and amoeba *Dictyostelium discoideum*. In contrast, cyclin subfamilies B, A, E, and D, which are cell cycle-related, have distinct evolutionary histories. The cyclin B subfamily is generally conserved in *D. discoideum,* fungi, and animals, whereas cyclin A and E subfamilies are both present in animals and their unicellular relatives such as choanoflagellate *Monosiga brevicollis* and filasterean *Capsaspora owczarzaki,* but are absent in fungi and *D. discoideum*. Although absent in fungi and *D. discoideum*, cyclin D subfamily orthologs can be found in the early-emerging, non-opisthokont apusozoan *Thecamonas trahens*. Within opisthokonta, the cyclin D subfamily is conserved only in eumetazoans, and is absent in fungi, choanoflagellates, and the basal metazoan *Amphimedon queenslandica.*

**Conclusions:**

Our data indicate that the CDK4/6 subfamily and eumetazoans emerged simultaneously, with the evolutionary conservation of the cyclin D subfamily also tightly linked with eumetazoan appearance. Establishment of the CDK4/6-cyclin D complex may have been the key step in the evolution of cell cycle control during eumetazoan emergence.

## Background

Cyclin-dependent kinases (CDKs) are serine and threonine kinases whose actions are dependent on the binding of regulatory subunits known as cyclins [1,2]. Various cyclins are synthesized and destroyed at specific times during the cell cycle, thus regulating CDK activity in a timely manner [3,4]. CDK and cyclin families function in a variety of cellular processes, including cell cycle regulation, transcription, RNA processing, translation, neurogenesis, and apoptosis [1,5,6].

The evolution of metazoans from protozoans is a major milestone in the history of life. This transition has been generally marked by increases in the number of genes involved in cell differentiation, cell-cell communication, and cell adhesion [7-12]. The evolutionary histories of transcription factors (Hox transcription factors and the Myc-max network) [12-16], cell-cell communication-related genes (Wnt, catenin, and receptor tyrosine kinase families, and the Ca^2+^ signaling ‘Toolkit’ ) [17-21], and cell adhesion genes (cadherin, integrin, and laminin families) [22-27] during the unicellular to metazoan transition have been extensively investigated.

In addition to cell communication and cell adhesion proteins, other proteins may be linked with metazoan emergence. Based on a comprehensive phylogenetic analysis of sponge *Amphimedon queenslandica* proteins, it has been proposed that the emergence of metazoan multicellularity may have been related to the evolution of various genes functioning in cell cycling and growth, programmed cell death, cell-cell and cell-matrix adhesion, developmental signaling and gene regulation, allorecognition and innate immunity, and cell type specialization [28].

As implied by the aforementioned study, investigation of the evolutionary history of cell cycle control genes could enhance our understanding of metazoan emergence from single-celled ancestors. At present, however, comprehensive evolutionary analyses have been carried out only for a few cell cycle control genes, such as P53, RB, and E2F families [29,30].

The core machinery of the animal cell cycle can generally be traced back to early eukaryotes [31-33]. It was previously proposed that the eukaryotic cell cycle was controlled by the DNA damage checkpoint kinase Chk1p at early stages of evolution, and duplications of kinase genes occurred during subsequent evolution. Gradually, eukaryotic kinases were added to the cell cycle control system, with CDKs being among the last major additions [34]. However, cyclin-dependent kinases (CDKs) in yeast and animal are thought to be the cornerstone in cell cycle control [1,6,35].

According to recent reports, 20 CDK and approximately 30 cyclin genes are present in humans [6,36,37]. The evolution of CDK and cyclin families has been studied previously. An analysis of the CDK family in yeasts and animals divided the CDK family into seven subfamilies (Pho85, CDC28, CTK, BC18H.15, SRB10, KIN28, and CDK4/6) [38], while another analysis examined 123 CDK family members from animals, plants, yeasts, and four protists [39]. With respect to the cyclin family, one phylogenetic analysis covered A-, B-, D-, and E-type cyclin proteins in animals and fungi [40]; another analysis included fungal, plant, and protist cyclins, and successfully divided all cyclins in three groups [41]. These analyses only incorporated a relatively limited number of organisms, however, with several representative organisms occupying key positions in the transition from unicellular to metazoan organisms not analyzed.

Taking advantage of the increasing number of sequenced genomes, in this study we conducted a comprehensive evolutionary analysis of 176 CDK and 226 cyclin genes from 18 representative organisms. Our analysis incorporated several organisms important to the study of metazoan emergence, such as the closest known metazoan relative, the choanoflagellate *Monosiga brevicollis*[42]; the oldest surviving metazoan, *Amphimedon queenslandica*[28]; the earliest eumetazoan, *Trichoplax adhaerens*[43]; and the cnidarian *Nematostella vectensis*[44]. We also included several unicellular organisms, such as the choanoflagellate *Salpingoeca rosetta* and the filasterean *Capsaspora owczarzaki,* that are recognized as close relatives of metazoans based on data from the Origins of Multicellularity project [10]. Our results revealed detailed evolutionary information regarding CDK and cyclin proteins in metazoan organisms and their unicellular relatives, and provided evidence for simultaneous CDK4/6-cyclin D complex and eumetazoan emergence.

## Methods

### Database searching and identification of CDK and cyclin sequences

For CDK proteins, we performed PSI-Blast searches using human CDK1 and CDK7 protein sequences as queries [45] against the NCBI non-redundant protein database (http://www.ncbi.nlm.nih.gov/) for 15 organisms: *Homo sapiens, Ciona intestinalis* (*C. intestinalis*)*, Strongylocentrotus purpuratus* (*S. purpuratus*)*, Branchiostoma floridae, Drosophila melanogaster* (*D. melanogaster*)*, N. vectensis, T. adhaerens, A. queenslandica, Monosiga brevicollis, S. rosetta, C. owczarzaki, Schizosaccharomyces pombe* (*S. pombe*)*, Saccharomyces cerevisiae* (*S. cerevisiae*)*, Coprinopsis cinerea* (*C. cinerea*)*,* and *Dictyostelium discoideum* (*D. discoideum*). The search results were used as new queries in a second round of BLAST searching, which was continued until no new sequences were returned. We also performed a similar BlastP search against the Broad Institute database [10] (http://www.broadinstitute.org/annotation/genome/multicellularity_project/MultiHome.html) to collect CDK sequences from three unicellular organisms: *Sphaeroforma arctica* (*S. arctica*), *Spizellomyces punctatus* (*S. punctatus*), and *Thecamonas trahens* (*T. trahens*), as these sequences are not available in the NCBI database. For CDK genes, only the longest protein sequence encoded by each gene was retained. We also carried out a preliminary phylogenetic analysis on all putative CDK family proteins collected from Blast searching. Proteins clustering with human CDKs were used in subsequent analyses, whereas those clustering with other human protein kinases, such as MAP kinases, were discarded.

Using human cyclin B, cyclin C, and cyclin Y proteins as queries, similar Blast searches were carried out to identify cyclin proteins from related organisms in the NCBI and Broad Institute databases. Because cyclin proteins are greatly diverged, an HMM search (http://hmmer.janelia.org/search/hmmsearch; *E*-value < 1 × 10^−4^) against non-redundant proteins from GenBank was also carried out [46] using Pfam profile PF00134, which corresponds to the Cyclin-N domain—the most highly conserved cyclin protein domain [41]. For three unicellular organisms (*S. arctica*, *S. punctatus*, and *T. trahens*), Cyclin-N domain-containing proteins were also collected from the Broad Institute database [10]. Only the longest protein sequence associated with each cyclin gene was retained. We verified the putatively identified cyclin proteins by searching against Pfam (http://pfam.sanger.ac.uk/search) and SMART (http://smart.embl-heidelberg.de/) databases [47,48]. Proteins lacking Cyclin-N domains were discarded. Similar to a previous analysis [41], non-cyclin proteins possessing Cyclin-N domains (homologs of human CABLES1, CNTD1, and CNTD2) were identified by reciprocal Blast searching and removed.

### Protein alignment and phylogenetic analyses

After evaluating several multiple alignment programs, we used MSAProbs [49] for multiple alignment of most full-length proteins. Alignments that included cyclin sequences from *T. trahens* and *D. discoideum* were carried out using PROMALS [50], a program more suitable for alignment of distantly related proteins [50]. Poorly aligned positions in these alignments were removed, with only the conserved region—the CDK domain for the CDK family, and Cyclin-N and –C domains for the cyclin family—used for further phylogenetic analyses. Alignments used for phylogenetic analyses are found in Additional file 1: file S1. Phylogenetic analyses were performed using maximum likelihood (ML) and Bayesian methods, with optimum substitution models determined for each alignment based on the Akaike Information Criterion using ProtTest 2.4 [51]. ML trees were constructed using RAxML 7.2.8 [52] as implemented in the CIPRES Science Gateway v. 3.1 [53] with 1000 bootstrap resamplings. Bayesian phylogenetic analyses were carried out under an LG substitution model using PHYLOBAYES v. 3.3 [54], with Markov chain Monte Carlo runs terminated when Maxdiff < 0.1. Multiple sequence alignments and phylogenetic tree files were deposited in Labarchives (http://dx.doi.org/10.6070/H4RF5S05). Tree files were viewed using the Dendroscope program [55], and phylogenetic networks were constructed with SplitsTree v.4 [56].

### Ortholog identification

As suggested in a recent review [57], ortholog identification of different CDK and cyclin subfamilies was mainly based on results of the phylogenetic analyses; however, results from reciprocal Blast search methods (Reciprocal Best Hit method) [58,59] were also referenced for some distantly related proteins. In general, a protein was identified as an ortholog of a representative CDK or cyclin subfamily if it clustered with that subfamily in the ML phylogenetic tree with greater than 50% bootstrap support. For proteins clustering with less than 50% ML bootstrap support within a subfamily, reciprocal Blast results were consulted: only proteins in the initial BLAST query for which *E*-values returned for members of a representative CDK or cyclin subfamily were five orders of magnitude better (smaller) than those of the next best-scoring CDK or cyclin subfamily were considered to be orthologs of that subfamily. Such a “five-orders criterion” has recently been described and used to identify Arf GAP orthologs [60]. Cyclin proteins placed with low bootstrap support into a representative cyclin subfamily and not meeting the five-orders criterion were designated as unclassified cyclin proteins.

## Results and discussion

As summarized in Table 1, we identified 176 CDK and 226 cyclin proteins from 18 representative organisms. Detailed information regarding these CDKs and cyclins may be found in Additional file 2: file S2 and Additional file 3: file S3, respectively.

**Table 1:** Distribution of CDK and cyclin family proteins in representative organisms

**Species**	**Phylum**	**CDK**	**Cyclin**
*Homo sapiens*	Vertebrata	20	29
*Ciona intestinalis*	Urochordata	10	14
*Strongylocentrotus purpuratus*	Echinodermata	11	14
*Branchiostoma floridae*	Cephalochordata	12	16
*Drosophila melanogaster*	Arthropoda	11	14
*Nematostella vectensis*	Cnidaria	12	16
*Trichoplax adhaerens*	Placozoa	14	14
*Amphimedon queenslandica*	Porifera	11	13
*Monosiga brevicollis*	choanoflagellata	10	8
*Salpingoeca rosetta*	choanoflagellata	6	8
*Capsaspora owczarzaki*	Filasterea	9	10
*Sphaeroforma arctica*	Ichthyosporea	3	9
*Saccharomyces cerevisiae*	Fungus	6	15
*Schizosaccharomyces pombe*	Fungus	7	11
*Coprinopsis cinerea*	Fungus	7	9
*Spizellomyces punctatus*	Fungus	8	8
*Thecamonas trahens*	Apusoza	7	9
*Dictyostelium discoideum*	Amoeboza	8	9
Total		172	226

### The evolutionary history of CDK family

**Figure 1:**
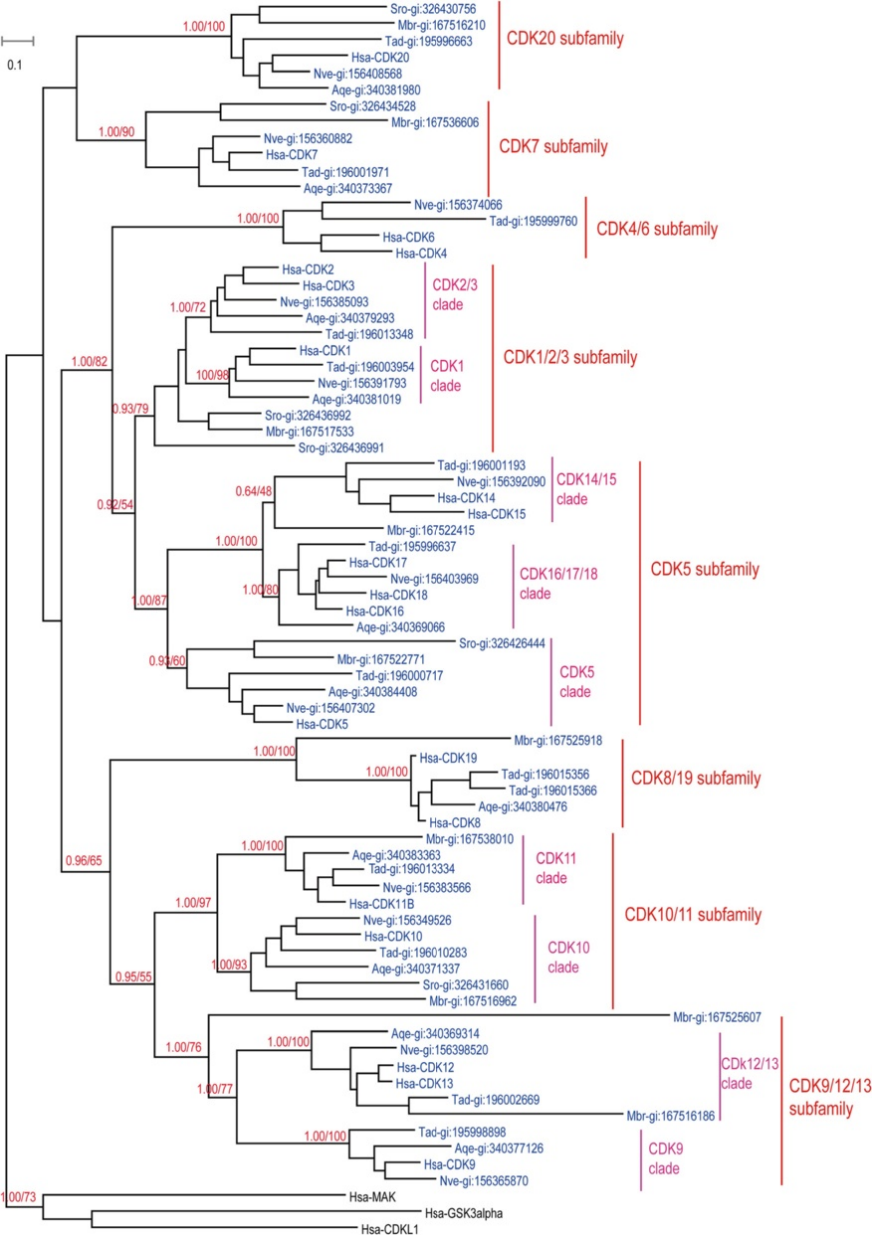
**Phylogenetic tree from analysis of CDK family proteins in *Homo sapiens*, *Nematostella vectensis*, *Thecamonas adhaerens*, *Amphimedon queenslandica*, *Monosiga brevicollis*, and *Salpingoeca rosetta*.** Maximum likelihood (ML) and Bayesian analyses were conducted using RAxML and PHYLOBAYES 3.3, respectively. Both methods produced trees with nearly identical topologies. The first numbers above branches indicate Bayesian posterior probabilities (only key branches are labeled), and the second numbers above branches indicate ML bootstrap percentages. The scale bar shows the number of substitutions per site. Sequences of Hsa-GSK3alpha, Hsa-MAK, and Hsa-HCDKL1 were used as outgroups. All proteins are labeled with species names followed by accession numbers. Species abbreviations are as follows: Hsa, *H. sapiens*; Nve, *N. vectensis*; Tad, *T. adhaerens*; Aqe, *A. queenslandica*; Mbr, *M. brevicollis*. The alignment used for this analysis is found in Additional file 1: File S1.

**Figure 2:**
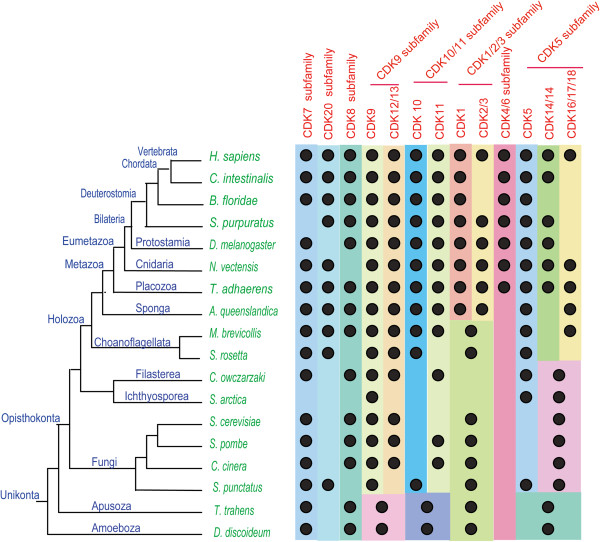
**Schematic representation of the distribution of different CDK subfamilies in eukaryotic organisms.** The results of phylogenetic analyses of CDK family proteins in different organisms are summarized. A black dot indicates the presence of clear homologs of CDK subfamilies or clades (see text for further explanations). Phylogenetic relationships of these organisms are based on recent reports [43,61,62] and the results of the Origins of Multicellularity project [10]. Detailed information regarding this figure, including CDK protein accession numbers, is given in Additional file 6: Table S1.

We attempted to perform global phylogenetic analyses using ML and Bayesian methods on all 176 CDK proteins from 18 organisms. A robust and reliable phylogenetic tree could not be obtained by either method, however, possibly because of the large number of sequences. We therefore carried out separate phylogenetic analyses on subsets of the 18 organisms. We first analyzed CDK sequences from *H. sapiens, N. vectensis, T. adhaerens, A. queenslandica, M. brevicollis,* and *S. rosetta7* as one group (Figure 1). These six organisms, except for *H. sapiens*, are all located in key positions with respect to metazoan emergence. Given that lineage-specific gene duplication and loss may have occurred in some of these organisms, any conclusions drawn from this subset may not be adequate to fully understand the evolutionary history of the CDK family during metazoan emergence. Consequently, analyses of CDK sequences from other organisms were also carried out; one analyzed subset comprised *C. intestinalis, B. floridae, S. purpuratus, D. melanogaster, T. adhaerens*, and *H. sapiens* (Additional file 4: Figure S1), while another group consisted of *C. owczarzaki, S. arctica*, *S. cerevisiae, S. pombe, S. punctatus, C. cinerea, T. trahens, D. discoideum, T. adhaerens*, and *H. sapiens* (Additional file 5: Figure S2)*.* Results of these phylogenetic analyses are summarized in Figure 2 and Additional file 6: Table S1, and are described in detail below.

Based on our analyses, the entire CDK family can be divided into eight subfamilies: CDK7, CDK20, CDK8/19 (including human CDK8 and CDK19), CDK9 (including CDK9, CDK12, and CDK13), CDK10/11 (including CDK10 and CDK11), CDK1 (including CDK1, CDK2, and CDK3), CDK4/6 (including CDK4 and CDK6), and CDK5 (including CDK5, CDK16, CDK17, CDK18, CDK14, and CDK15) (Figures 1 and 2; Additional files 4: Figure S1 and Additional file 5: Figure S2). Although these results are generally consistent with previous reports [38,39], our analysis revealed more detailed information and included human CDK20 and its orthologs.

We found that orthologs of seven CDK subfamilies (CDK7, CDK20, CDK8/19, CDK9/12/13, CDK10/11, CDK1/2/3, and CDK5) are present in basal metazoans *T. adhaerens* and *A. queenslandica*, the choanoflagellate *M. brevicollis*, and/or *S. rosetta* (Figure 1). In further analysis, orthologs of 6 CDK subfamilies (CDK7, CDK8/19, CDK9/12/13, CDK10/11, CDK1/2/3, and CDK5 subfamily) could be identified in fungi, *T. trahens,* and *D. discoideum.* A CDK20 representative is present in the fungus *S. punctatus* (SPPG_01972)*,* but is absent in *S. cerevisiae*, *S. pombe, T. trahens,* and *D. discoideum* (Additional file 5: Figure S2). Compared with other CDK subfamilies, the CDK4/6 subfamily is unique, as it is only found in eumetazoan organisms such as *T. adhaerens* (gi:195999760) and *N. vectensis* (gi:156374066), and is absent in the basal metazoan *A. queenslandica*, choanoflagellates, and other investigated unicellular organisms (Figures 1 and 2; Additional file 5: Figure S2). The different functions of these CDK subfamilies have been described in an excellent review [63].

The CDK4/6 subfamily is generally recognized as animal-specific, but previous phylogenetic analyses supporting this conclusion have only included a relatively small number of organisms [38,39]. In the tree shown in Figure 1, subfamilies CDK4/6, CDK1/2/3, and CDK5 generally cluster together. As phylogenetic networks are useful for describing complex evolutionary scenarios such as horizontal gene transfer and recombination [56], we carried out a phylogenetic network analysis for CDK4/6, CDK1/2/3, and CDK5 subfamily proteins from *H. sapiens, N. vectensis, T. adhaerens, A. queenslandica, M. brevicollis,* and *S. rosetta* (Additional file 7: Figure S3). The results of that analysis were generally consistent with our phylogenetic tree topology, CDK4/6 subfamily is located between CDK1/2/3 subfamily and CDK5 subfamily. The detail evolutionary information among CDK4/6 subfamily, CDK1/2/3 subfamily and CDK5 subfamily are still requiring further study. Anyway, our analysis is the first to map the detailed evolutionary history of the CDK4/6 subfamily in representative organisms occupying key positions along the transition from unicellular organisms to metazoans. Our results indicate that the CDK4/6 subfamily is linked simultaneously with eumetazoan appearance.

Subfamilies CDK9, CDK10/11, CDK1/2/3, and CDK5 all contains more than one CDK members in metazoan organisms and every subfamily could be divided into two or three clades. Our analysis provided some detailed information about how and when these clades were formed in different subfamilies.

The CDK9 subfamily consists of two clades, CDK9 and CDK12/13. Basal metazoan organisms *T. adhaerens* and *A. queenslandica* have representative members in both clades (Figure 1). In addition, consistent with previous reports [38], *S. pombe* Lsk1 and *S. cerevisiae* Ctk1p belong to the CDK12/13 clade, while *S. cerevisiae* SGv1p and *S. pombe* CDK9 are members of the CDK9 clade (Additional file 5: Figure S2). These results imply that the CDK9 subfamily split into two clades (CDK9 and CDK12/13) before the divergence of metazoans and fungi. In humans, CDK9 is reported to regulate transcription by phosphorylating the C-terminal domain of RNA polymerase II [63,64].

The CDK10/11 subfamily comprises clades CDK10 and CDK11 (Figure 1). Basal metazoans *A. queenslandica and T. adhaerens* have members in both clades; similarly, unicellular *M. brevicollis* is represented in both CDK10 (gi:167516962) and CDK11 (gi:167538010). In further analysis*,* we found that one *S. punctatus* protein (SPPG_05640) was classified into the CDK10 clade, and that one *S. pombe* protein (Ppk23/gi:19112531) and one *C. cinerea* protein *(*gi:299755758) were placed in the CDK11 clade (see Additional file 5: Figure S2). Proteins from *D. discoideum* (gi:66827511 and gi:66810856) and *T. trahen*s (AMSG_04682) were only grouped into the CDK10/11 subfamily, and were not found in clades CDK10 and CDK11. In addition to their roles in regulating transcription, CDK10 and CDK11 display distinct functions during the G2/M transition [63,65].

The CDK1/2/3 subfamily can be divided into CDK1 and CDK2/3 clades in metazoans. Bona fide CDK1 clade members are found in *A. queenslandica* (gi:340381019) and *T. adhaerens* (gi:196003954), and similarly CDK2/3 clade members are present in *A. queenslandica* (gi:340379293) and *T. adhaerens* (gi:196013348) (Figure 1). CDK1/2/3 subfamily members from *M. brevicollis* (gi:167517533), *S. rosetta*, fungi, *T. trahen*s*,* and *D. discoideum*, however, are not placed into CDK1 or CDK2/3 clades (Figure 1; Additional file 5: Figure S2). Our data indicate that genes of the ancient CDK1/2/3 subfamily arose in the common ancestor of amoebozoans and fungi, and then diverged via gene duplication into clades CDK1and CDK2/3 in metazoans.

In the tree in Figure 1, the CDK5 subfamily is divided into clades CDK5, CDK16/17/18, and CDK14/15. The placozoan *T. adhaerens* possesses three CDK5 subfamily members, which are classified into CDK5 (gi:196000717), CDK16/17/18 (gi:195996637), and CDK14/15 (gi:196001193) clades (Figure 1). We found that one *M. brevicollis* protein (gi:167522771) is clustered into CDK5, whereas another (gi:167522415) is placed into CDK16/17/18 (Figure 1). In the comprehensive analysis, some fungal CDK5 subfamily members (SARC_06703 and SPPG_00440) were classified into the CDK5 clade, while others (SARC_10569, Pho85p/gi:6325226, Pef1/gi:19075421, SPPG_06236) were placed into clades CDK16/17/18 and CDK14/15 (see Additional file 5: Figure S2). These data indicate that the CDK5 subfamily originated in single-celled ancestors, and subsequently split into three clades before or during eumetazoan emergence. In humans, CDK5 is essential for neuronal cell cycle arrest and differentiation [63,66].

### The evolutionary history of cyclin family

**Figure 3:**
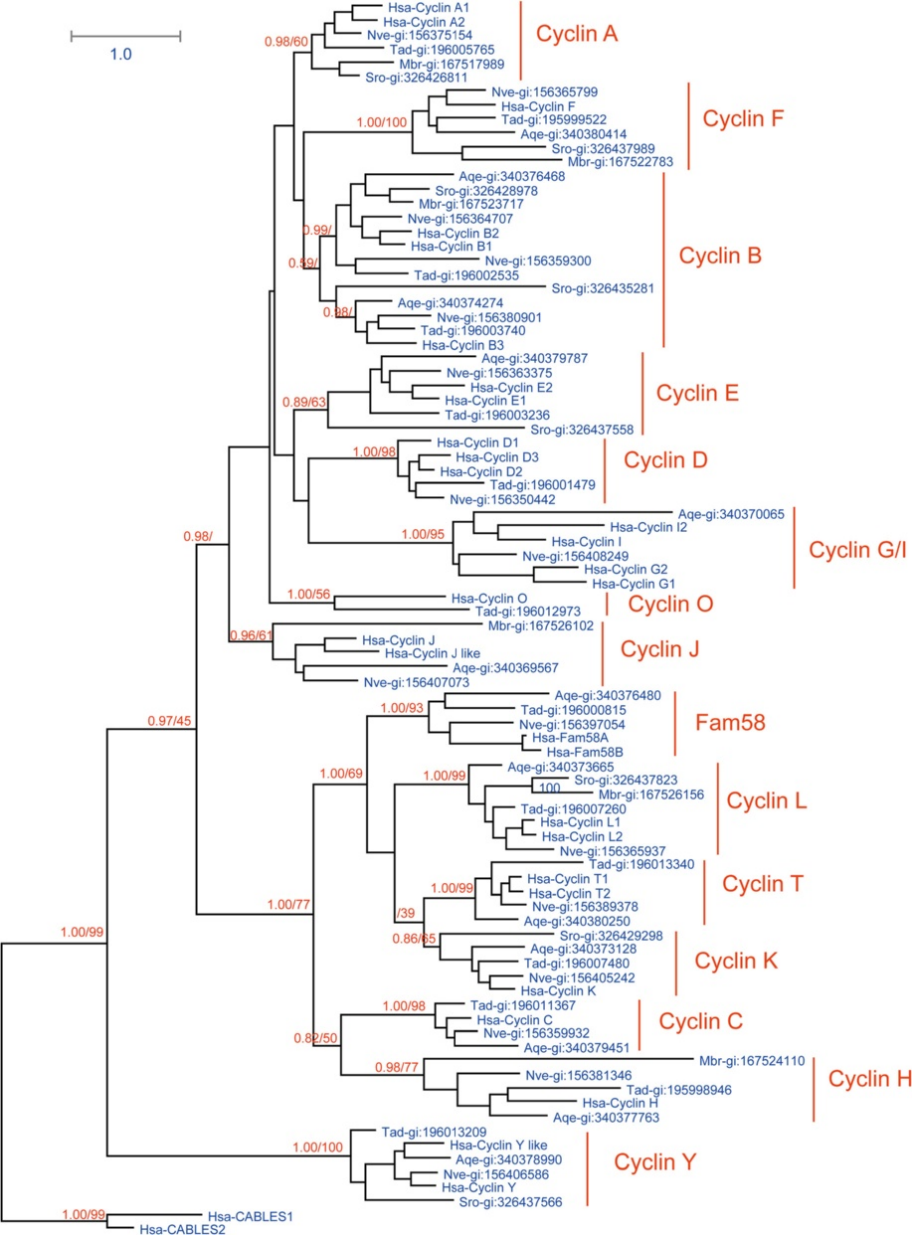
**Tree derived from phylogenetic analysis of cyclin family proteins in *H. sapiens*, *N. vectensis*, *T. adhaerens*, *A. queenslandica*, *M. brevicollis*, and *S. rosetta*.** Maximum likelihood (ML) and Bayesian analyses were conducted using RAxML and PHYLOBAYES 3.3, respectively. Both methods produced trees with nearly identical topologies. The first numbers above branches indicate Bayesian posterior probabilities (only key branches are labeled), and the second numbers above branches indicate ML bootstrap percentages. The scale bar shows the number of substitutions per site. Sequences of Hsa-Cables1 and Hsa-Cables2 were used as outgroups. All proteins are labeled with their accession numbers preceded by their species names. Species abbreviations are as follows: Hsa, *H. sapiens*; Nve, *N. vectensis*; Tad, *T. adhaerens*; Aqe, *A. queenslandica*; MBr, *M. brevicollis.* The alignment used for this analysis is found in Additional file 1: File S3.

**Figure 4:**
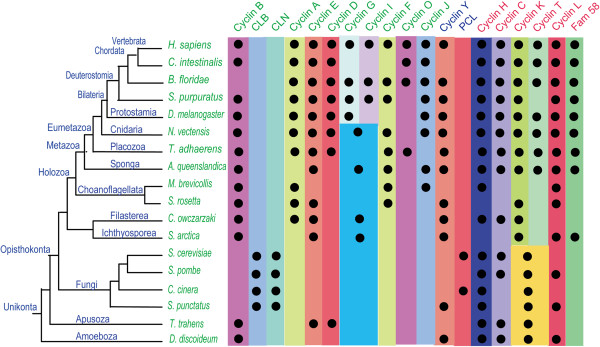
**Schematic representation of the distribution of different cyclin subfamilies in eukaryotic organisms.** The results of phylogenetic analyses of cyclin family proteins in different organisms are summarized. A black dot indicates the presence of clear homologs of cyclin subfamily members (see text for further explanations). Phylogenetic relationships illustrated for these organisms are derived form a proteome-based phylogeny [43,61,62] and the results of the Origins of Multicellularity project [10]. The names of cyclin B-like (cyclins B, A, D, E, J, F, G, I, O, CLB, and CLN); cyclin Y-like (cyclins Y and PCL), and cyclin C-like (cyclins C, H, L, K, T, and Fam58) group members are indicated by different colors. Detailed information regarding this figure, including cyclin protein accession numbers, may be found in Additional file 12: Table S2.

Based on the same reasons as in the CDK family, we carried out separate phylogenetic analyses on subsets of cyclin family members. As for the CDKs, we performed analyses of cyclin proteins from the group *H. sapiens*, *N. vectensis, T. adhaerens, A. queenslandica, M. brevicollis,* and *S. rosetta* (Figure 3), and then from a subset consisting of *C. intestinalis, B. floridae, S. purpuratus, D. melanogaster, T. adhaerens*, and *H. sapiens* (Additional file 8: Figure S4). Because their cyclin sequences diverged greatly, we failed to obtain a reliable cyclin phylogenetic tree from eight organisms: *C. owczarzaki, S. arctica*, *S. cerevisiae, S. pombe, S. punctatus, C. cinerea, T. trahens, D. discoideum*, *T. adhaerens*, and *H. sapiens*. We thus divided these organisms into smaller subsets for analysis: one group for filasterean and ichthyosporean organisms *(C. owczarzaki*, *S. arctica, T. adhaerens*, and *H. sapiens*; Additional file 9: Figure S5)*,* one group for fungi (*S. cerevisiae, S. pombe, C. cinerea, S. punctatus, T. adhaerens*, and *H. sapiens)* (Additional file 10: Figure S6)*,* and one group for Apusozoa and Amoebozoa *(T. trahens, D. discoideum, T. adhaerens*, and *H. sapiens)* (Additional file 11: Figure S7). These subgroups were analyzed, and orthologs of different cyclin subfamilies were classified (Figure 4; Additional file 12: Table S2) based on the phylogenetic results and those of reciprocal Blast analysis.

According to our phylogenetic analyses, the metazoan cyclin family could be divided into 16 subfamilies (Figure 3 and Figure 4), and fungi organisms owns three fungi specific subfamily (CLB, CLN, PCL) (see Additional file 10: Figure S6, Figure 4). A recent cyclin family analysis indicated that the cyclin family could be divided three groups (Group I, Group II, and Group III) [41], and our analysis confirmed it (Figure 3, Figure 4, see Additional file 10: Figure S6). In this manuscript, we will refer Group I as cyclin B like group (cyclin B, A, D, E, J, F, G, I, O, CLB, CLN), Group II as cyclin Y like Group ( cyclin Y, PCL), and Group III as cyclin C like group (cyclin C, H, L, K, T, and Fam58). Though our results is general consistent with previous analysis [41], some new information was revealed by our analysis. For example, our analysis successfully identificated cyclin C ortholog (gi:198414966) and cyclin J ortholog (gi:198425946) in *C. intestinalis*, the previous analysis [41] which also included *C. Intestinalis* missed this information.

Most subfamilies in the cyclin C-like group are conserved in metazoans, choanoflagellates, fungi, and *D. discoideum.* We found that cyclins C, H, and L are all conserved in metazoans, choanoflagellates, fungi, *T. trahens*, and *D. discoideum* (Figures 3 and 4; Additional file 9: Figure S5, Additional file 10: Figure S6, and Additional file 11: Figure S7)*.* Orthologs of cyclins T and K were found in metazoans (Figure 3); in fungi and *D. discoideum,* however, homologous proteins of cyclin T and/or cyclin K subfamily could only be identified as the common ancestor of the two subfamilies (Additional file 10: Figure S6 and Additional file 11: Figure S7). Our data indicate that the common ancestor of cyclins K and T originated early in the course of evolution, and then diverged no later than during the period of metazoan emergence*.* Fam58 is generally conserved only in metazoans (Figure 3), although an ortholog is also found in *S. arctica* (Additional file 9: Figure S5).

We found that the cyclin Y subfamily is conserved in metazoans, choanoflagellates, and *D. discoideum* (Figure 3; Additional file 11: Figure S7). Interestingly, one fungal protein in *S. punctatus* (SPPG_07965) was identified as an ortholog of cyclin Y (Additional file 10: Figure S6). PCL subfamily members are found in *S. cerevisiae* and *C. cinerea* (Additional file 10: Figure S6). In fact, cyclin Y and PCL subfamilies cluster together tightly in the phylogenetic tree (Additional file 10: Figure S6). Cyclins Y and PCL are binding partners of CDK5 subfamilies in metazoans and fungi, respectively [41]; although we list them as two separate subfamilies, as previously reported [41], we believe they share a common ancestor.

Evolutionary conservation varied greatly among different subfamilies in the cyclin B-like group. These subfamilies are described in detail as follows.

**Figure 5:**
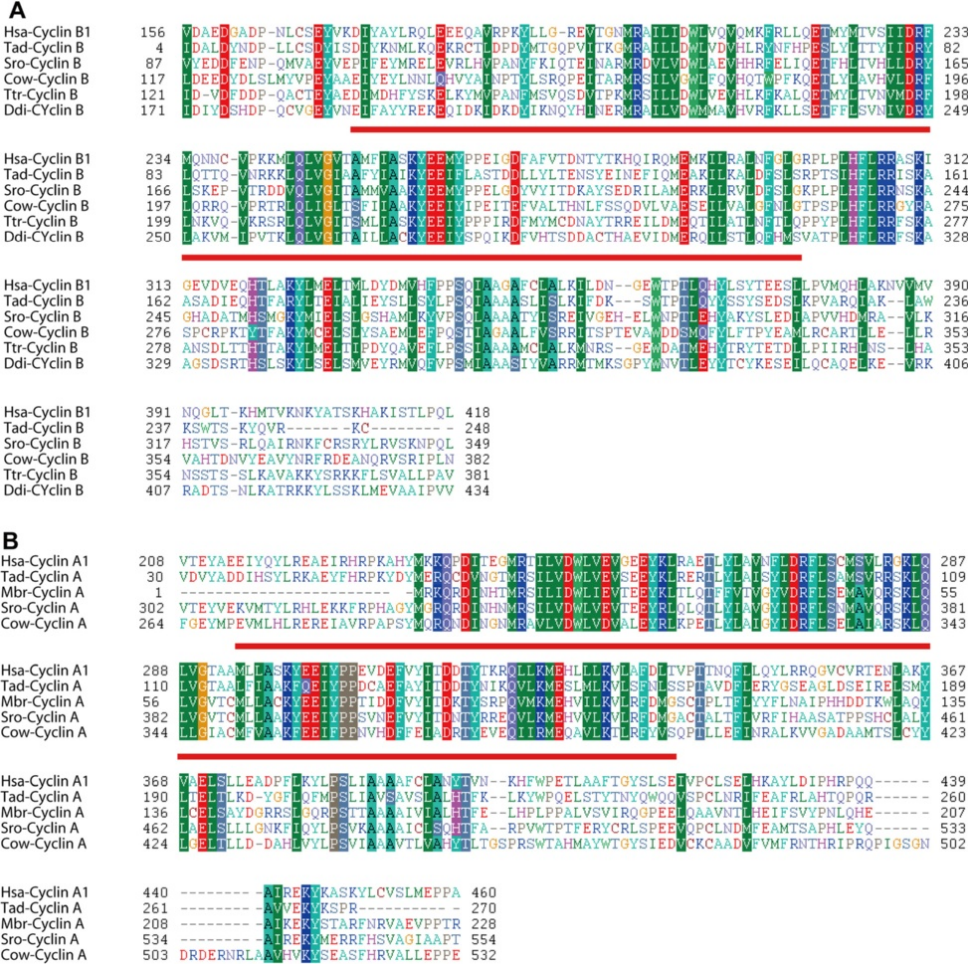
**Alignments of cyclin B and cyclin A proteins. A**. Alignment of cyclin B proteins from representative organisms. The region of Cyclin_N domain was underlined. Protein accession numbers are as follows: Hsa-cyclin B1: gi:14327896 from *H. sapiens*; Tad-cyclin B: gi:196002535 from *T. adhaerens*; Sro-cyclin B: gi:326428978 from *S. rosetta*; Cow-cyclin B: gi:320166256 from *C. owczarzaki*; Ttr-cyclin B: AMSG_03352 from *T. trahens*; Ddi-cyclin B: gi:66819865 from *D. discoideum*. **B**. Alignment of cyclin A proteins from representative organisms. The region of Cyclin_N domain was underlined. Protein accession numbers are as follows: Hsa-cyclin A1:gi:4502611 from *H. sapiens*; Tad-cyclin A: gi:196005765 from *T. adhaerens*; Mbr-cyclin A: gi:167517989 from *M. brevicollis*; Sro-cyclin A: gi:326426811 from *S. rosetta*; Cow-cyclin A: gi:320169862 from *C. owczarzaki*.

The cyclin B subfamily is conserved in metazoans, choanoflagellates, fungi, *T. trahens*, and *D. discoideum* (Figures 3 and 4; Additional files 9: Figure S5, Additional file 10: Figure S6, and Additional file 11: Figure S7)*.* The fungus-specific subfamily CLB is related to the cyclin B family, with Blast *E*-values as low as approximately 1 × 10^−60^. Cyclins B and CLB are binding partners of CDK1 subfamilies in metazoans and fungi, respectively [35,67]. Consequently, although we treat cyclins B and CLB as two subfamilies, as previously reported [41], they appear to share a common ancestor in early eukaryotic lineages, as suggested by previous analyses [40,41]. Alignments of representative cyclin B subfamily proteins from metazoan organisms *H. sapiens* and *T. adhaerens* and unicellular organisms *S. rosetta, C. owczarzaki*, *T. trahens*, and *D. discoideum* are shown in Figure 5.

Our analysis results indicate that the cyclin A subfamily is conserved in metazoans, unicellular choanoflagellates, and *C. owczarzaki*, but is absent in fungi, *T. trahens*, and *D. discoideum* (Figures 3 and 4; Additional file 9: Figure S5, Additional file 10: Figure S6, and Additional file 11: Figure S7)*.* Alignments of representative cyclin A subfamily proteins from metazoans *H. sapiens, T. adhaerens*, and unicellular organisms *M. brevicollis, S. rosetta*, and *C. owczarzaki* are given in Figure 5.

The fungus-specific subfamily CLN, which functions in cell cycle regulation, is the binding partner of fungus CDK1. Results of Blast analysis revealed similar genetic distances between the CLN subfamily and the metazoan cyclin A subfamily, and between CLN and the metazoan cyclin B subfamily.

**Figure 6:**
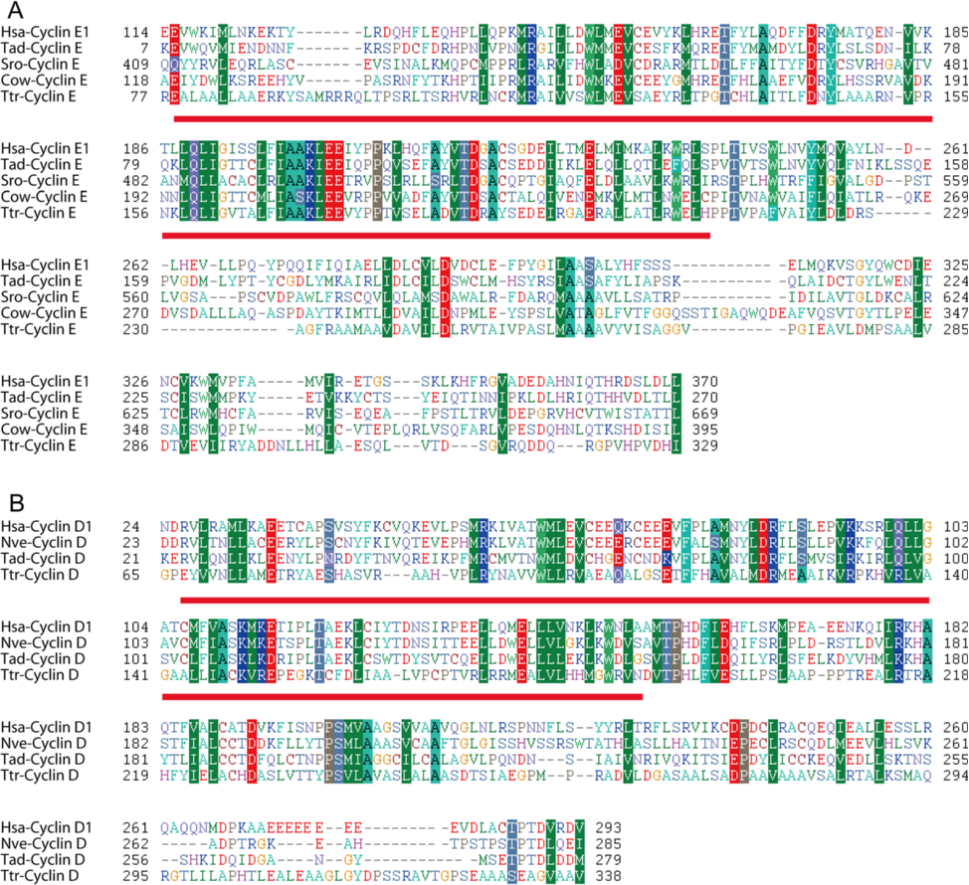
**Alignments of cyclin E and cyclin D proteins. A**. Alignment of cyclin E proteins from representative organisms. The region of Cyclin_N domain was underlined. Cyclin accession numbers are as follows: Hsa-cyclin E1: gi:17318559 from *H. sapiens*; Tad-cyclin E:gi:196003236 from *T. adhaerens;* Sro-cyclin E: gi:326437558 from *S. rosetta*; Cow-cyclin E:gi:320167008 from *C.owczarzaki;* Ttr-cyclin E: AMSG_07694 from *T. trahens*. **B**. Alignment of cyclin D proteins from representative organisms. The region of Cyclin_N domain was underlined. Cyclin accession numbers are as follows: Hsa-cyclin D1: gi|16950655 from *H. sapiens*; Nve-cyclin D: gi:156350442 from *N. vectensis;* Tad-cyclin D: gi:196001479 from *T. adhaerens*; Ttr-cyclin D: AMSG_02061 from *T. trahens*.

The cyclin E subfamily is not only conserved in metazoans, but is also present in several unicellular organisms such as choanoflagellate *S. rosetta, C. owczarzaki,* and *T. trahens* (Figures 3 and 4; Additional file 9: Figure S5, Additional file 10: Figure S6, and Additional file 11: Figure S7). This result conflicts with previous studies indicating that cyclin E is animal-specific [28,41]. This inconsistency may be due to the failure of previous analyses to incorporate several important unicellular organisms, such as *S. rosetta* and *C. owczarzaki*. Alignments of representative cyclin E subfamily proteins from metazoans *H. sapiens* and *T. adhaerens* and unicellular organisms *S. rosetta, C. owczarzaki*, and *T. trahens* are shown in Figure 6.

We found that orthologs of cyclin D are present in *T. adhaerens* (gi:196001479) and *N. vectensis* (gi:156350442), but are absent in *M. brevicollis, S. rosetta*, and *A. queenslandica* (Figures 3 and 4). Comprehensive analysis unexpectedly revealed that cyclin D orthologs are present in *T. trahens* (AMSG_02061) (Additional file 11: Figure S7)*,* although absent in fungi and *D. discoideum* (Additional file 10: Figure S6 and Additional file 11: Figure S7). Although plant D-type cyclin is generally considered to be homologous to animal cyclin D [31,68], plants do not possess CDK4/6 orthologs; instead, plant D-type cyclin functions together with plant CDKA, a CDK1 homolog, in the G1 phase [31,68]. Possibly because of low sequence similarity (Blast *E*-value approximately 1 × 10^−7^ for plant D-type cyclin against human cyclin D), plant D-type cyclin did not cluster together with animal cyclin D in a previous phylogenetic analysis [69]. Our phylogenetic results are the first to reveal the presence of a bona fide ortholog of the animal cyclin D subfamily in a non-opisthokont, *T. trahens*. The Blast *E*-value for this protein against human cyclin D is approximately 1 × 10^−15^. This result indicates that the cyclin D subfamily arose in early eukaryotes, and that the absence of cyclin D in fungi and many other unicellular organisms may be due to lineage-specific gene loss in these organisms. Similar to plant organisms, the non-opisthokont *T. trahens* does not possess an ortholog of the CDK4/6 subfamily. Our alignment of representative cyclin D subfamily proteins from *H. sapiens, N. vectensis*, *T. adhaerens*, and *T. trahens* is displayed in Figure 6.

In our analyses, cyclins I and G always clustered together. Cyclins I and G collectively have representative members in unicellular organisms *C. owczarzaki, S. arctica, A. queenslandica, and N. vectensis* (Figures 3 and 4)*.* These data indicate that cyclin subfamilies I and G are derived from a common ancestral gene that was present in unicellular organisms, with this common ancestor differentiating into cyclins I and G after the emergence of *N. vectensis*.

In addition to the above phylogenetic analyses, we conducted a phylogenetic network analysis of cyclin B-like group proteins from *H. sapiens, N. vectensis, T. adhaerens, A. queenslandica, M. brevicollis,* and *S. rosetta* (Additional file 13: Figure S8). It was found that the cyclin D subfamily is located between subfamily E and subfamily G/I. The detail evolutionary information among cyclin D subfamily, cyclin E, and cyclin G/I will be an interesting topic for further study.

### Cell cycle related CDK/cyclin evolutionary histories during animal emergence

Information is limited regarding evolution of cell cycle regulation in eukaryotes. It is generally believed, however, that early eukaryotes already possessed complex cell cycle regulation, with key cell cycle regulators having subsequently undergone divergent functional specializations in different organisms [31]. For example, the RB-E2F pathway, which functions in cell cycle regulation, is conserved in animals and plants, but has been lost in fungi [30,31].

The eukaryotic cell cycle is controlled by a complicated regulatory network [70]. CDK-cyclin complexes, as key regulators of the cell cycle, phosphorylate a variety of substrates during the cell cycle [71,72]. In humans, for example, CDK4/cyclin D phosphorylates pRB during the G1 phase [73], and CDK1-cyclin B phosphorylates Cdc25C and Wee1A during the M phase [74,75]. A recent structural study [76] revealed that the conformation of t CDK4/ cyclin D1 diverges from that of previously known CDK-cyclin binary complexes, and CDK4 might have a unique regulation and activation mechanism compared with that of CDK2-cyclin A [76]. Another study has also found that the structural mechanism of CDK4-cyclin D3 activation differs markedly from that of previously studied CDK2-cyclin A complexes [77].

Our analysis has provided detailed evolutionary information on CDK and cyclin subfamilies in metazoans and related organisms. Our data are the first to reveal that cyclin D orthologs are present in a non-opisthokont (*T. trahens*)*,* but have generally been lost in fungi and most other unicellular opisthokonts, such as *M. brevicollis, S. rosetta, C. owczarzaki,* and *S. arctica.* Our analysis also found that cyclin E is not restricted to animals, but is present in several unicellular organisms.

**Figure 7:**
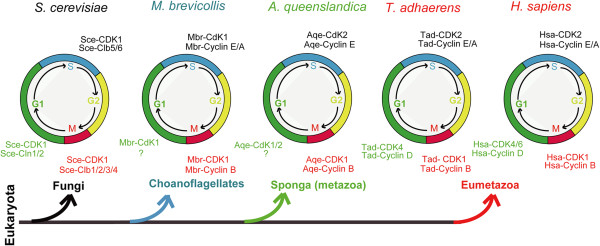
**Schematic scenarios of CDK and cyclin protein function in cell cycle regulation of different representative organisms.** Schemes for organisms *S. cerevisiae* and *H. sapiens* were drawn based on previous reports [6,37,67,70], and schemes for *M. brevicollis*, *A. queenslandica*, and *T. adhaerens* were drawn based on inferences derived from our evolutionary analysis (see text for further explanations). Accession numbers of CDK and cyclin proteins in the figure are as follows: Sce-CDK1: gi:6319636; Sce-Cln1/2: gi:6323855, gi:6324999; Sce-Clb5/6: gi:6325377, gi:6321546; Sce-Clb1/2/3/4: gi:6321545, gi:6325376, gi:6320046, gi:6323239; Mbr-CdK1: gi:167517533; Mbr-cyclin B: gi:167523717, gi:167524669; Mbr-cyclin E: gi:167519314; Mbr-cyclin A: gi:167517989; Aqe-CDK1: gi:340381019, Aqe-CdK2: gi:340379293; Aqe-cyclin B: gi:340376468, gi:340374274; Aqe-cyclin E: gi:340379787; Tad- CDK1: gi:196003954; Tad-CDK2: 196013348; Tad-CDK4: gi:195999760; Tad-cyclin B: gi:196002535; gi:196003740; Tad-cyclin E: gi:196003236; Tad-cyclin A: gi:196005765; Tad-cyclin D: gi:196001479.

Investigations of cell cycle regulation have primarily been carried out in animals (e.g., *D. melanogaster*, *Caenorhabditis elegans*, *Xenopus laevis*, and *H. sapiens*) and yeasts (*S. cerevisiae* and *S. pombe*). In animals, CDK4/6 and cyclin D have been determined to function in the G1 phase, human CDK2 and cyclin A/E in S and G2 phases, and CDK1 and cyclin B in the M phase [5,37]. In yeasts, *S. cerevisiae* CDK1 (Sce-CDC28/gi:6319636) functions in G1, S, G2, and M phases with different cyclins [67,78]. Given the large evolutionary distance between yeasts and animals, it has proved difficult to elucidate the evolutionary history of cell cycle regulation and its relationship to the emergence of metazoans from their single-celled ancestors. In this study, we analyzed cell cycle-related CDKs (CDK1/2/3 and CDK4/6 subfamilies) and cyclins (cyclin A, B, D, and E subfamilies) in several representative organisms, such as *M. brevicollis*[42]*, A. queenslandica*[28]*,* and *T. adhaerens*[43]*,* which occupy key positions for metazoans origination from their single-celled ancestors organisms (Figure 7). We discovered that the number of cell cycle-related CDK and cyclin proteins has gradually increased from *M. brevicollis* and *A. queenslandica* to *T. adhaerens*: *M. brevicollis* possesses orthologs for CDK1, cyclin B, cyclin A, and cyclin E, *A. queenslandica* has orthologs for CDK1, CDK2, cyclin B, cyclin A, and cyclin E, and *T. adhaerens* features orthologs for CDK1, CDK2, CDK4, cyclin B, cyclin A, cyclin E, and cyclin D (Figures 2 and 4; Additional files 6: Table S1 and Additional file 12: Table S2). Based on the evolutionary information uncovered for these CDK and cyclin proteins, we are able to propose different scenarios for the function of CDK and cyclin proteins in cell cycle control in representative organisms *M. brevicollis, A. queenslandica,* and *T. adhaerens* (Figure 7)*.* Because *M. brevicollis* does not possess CDK4/6 and cyclin D orthologs, we speculate that the ortholog of CDK1 in *M. brevicollis (*gi:167517533*)* may function throughout the cell cycle with different cyclins, similar to *S. cerevisiae* CDK1 (Sce-cdc28/gi:6319636) (Figure 7). As indicated in Figure 7, we have inferred that the CDK4/6-cyclin D complex appeared at the same time as the emergence of the eumetazoan *T. adhaerens*.

It is interesting that the CDK4/6-cyclin D complex first emerged in *T. adhaerens*, as *T. adhaerens* has four morphologically identifiable somatic cell types and is thought to represent the earliest eumetazoan lineage [43]. The CDK4/6-cyclin D complex functions in the G1 phase, the first phase within interphase [5]. The duration of the G1 phase is highly variable among different cells in animals, and is affected by limiting growth factors, nutrient supply, temperature, and additional inhibiting factors [79]. For example, human embryonic stem cells are characterized by an abbreviated G1 phase and lack the classical restriction (R) point that normally controls commitment for progression into the S phase [80,81]. In contrast, somatic cell proliferation is linked to growth factor-dependent passage through the R point in the G1 phase [82,83]. In fission yeast, a single oscillation of p34cdc2 kinase activity provided by a single B-type cyclin can promote ordered progression into both DNA replication and mitosis [84]. The function of CDK4-cyclin complexes in animals has been extensively studied and reviewed [85]. With respect to the cyclin D family, mice lacking cyclin D1, D2, or D3 exhibit different developmental anomalies [85-90]. Mice expressing cyclin D1, but not D2 and D3, have been observed to die before embryonic day (E) 18.5 [85,91], while mice lacking all three cyclins die before E16.5 [85,92]. In regard to CDK4 and CDK6, mice lacking Cdk4 or Cdk6 also exhibit different developmental anomalies [85,93-96], with mice lacking both CDK4 and CDK6 displaying progressive embryonic lethality from E14.5 onward, and the few live pups dying shortly after birth [85,96]. These data clearly indicate that the CDK4-cyclin D complex plays critical roles during mouse early embryonic development. It would be interesting to study the function of CDK4-cyclin D in early eumetazoan organisms such as *N. vectensis* and *T. adhaerens.*

Based on a comparative analysis of cell cycle regulatory networks in animals, yeasts, and plants, Harashima et al. [31] have recently suggested that the CycD/CycE clade has undergone lineage-specific expansion and specialization in both metazoans and plants. They further speculate that this expansion and specialization of cell cycle protein families has occurred to meet the challenges of a complex multicellular lifestyle. The comprehensive evolutionary histories of CDKs and cyclins outlined in our study provided new evidence for their hypotheses. We believe that the emergence of the CDK4/6-cyclin D complex may have contributed to the formation of eumetazoan-specific G1 phase regulation, and may represent a key step in the development of cell cycle regulation during eumetazoan evolution.

## Conclusions

In this study, we conducted a comprehensive evolutionary analysis of CDK and cyclin proteins in metazoans and their unicellular relatives. Our results indicated that CDK family could be divided into eight subfamilies. Seven subfamilies (CDK1/2/3, CDK5, CDK7, CDK 20, CDK8/19, CDK9, and CDK10/11) are conserved in metazoans and fungi, with CDK4/6 subfamily found only in eumetazoans. As to cyclins, cyclin C, H, L, Y subfamilies, and cyclin K and T as a whole subfamily, are conserved in animal, fungi, and amoeba *Dictyostelium discoideum.* The cyclin B subfamily is conserved in *D. discoideum,* fungi, and animals, whereas cyclin A and E subfamilies are both present in animals and their unicellular relatives such as choanoflagellate *Monosiga brevicollis* and filasterean *Capsaspora owczarzaki,* but are absent in fungi and *D. discoideum*. Cyclin D subfamily orthologs can be found in the early-emerging, non-opisthokont apusozoan *Thecamonas trahens*. Within opisthokonta, the cyclin D subfamily is conserved only in eumetazoans, and is absent in fungi, choanoflagellates, and the basal metazoan *Amphimedon queenslandica.*

Our data indicate that the CDK4/6 subfamily and eumetazoans emerged simultaneously, with the evolutionary conservation of the cyclin D subfamily also tightly linked with eumetazoan appearance. We speculated that establishment of the CDK4/6-cyclin D complex may have been the key step in the evolution of cell cycle control during eumetazoan emergence.

## Abbreviations

H. sapiens: Homo sapiens; C. intestinalis: Ciona intestinalis; S. purpuratus: Strongylocentrotus purpuratus; B. floridae: Branchiostoma floridae; D. melanogaster: Drosophila melanogaster; N. vectensis: Nematostella vectensis; T. adhaerens: Trichoplax adhaerens; A. queenslandica: Amphimedon queenslandica; M. brevicollis: Monosiga brevicollis; S. rosetta: Salpingoeca rosetta; C. owczarzaki: Capsaspora owczarzaki; S. arctica: Sphaeroforma arctica; S. pombe: Schizosaccharomyces pombe; S. cerevisiae: Saccharomyces cerevisiae; C. cinerea: Coprinopsis cinerea; S. punctatus: Spizellomyces punctatus; T. trahens: Thecamonas trahens; D. discoideum: Dictyostelium discoideum.

## Competing interests

The authors declare that they have no competing interests.

## Authors’ contributions

LC and LY conceived the study. LC, FC, JC, and JX collected the data. LC and FC performed the phylogenetic analyses, and JX conducted the reciprocal blast analysis. LC and XY wrote the manuscript. All authors read and approved the final manuscript.

## Supplementary Material

Additional file 1: File S1 All Multiple alignments of CDK or cyclin proteins which were used for phylogenetic analysis. Multiple alignments of full-length proteins were mainly carried out using MSAProbs program [44], however, the protein alignment which include the cyclin sequence from *T. trahens* and *D. discoideum* was carried out using PROMALS program [45], Then the poorly aligned positions in these alignments were removed, only the conserved region (the CDK domain for CDK family, the Cyclin_N domain and Cyclin_C domain for cyclin family) in these alignments were used for further phylogenetic analysis. Click here for file

Additional file 2: File S2 CDK sequences from 18 organisms. Click here for file

Additional file 3: File S3 Cyclin sequences from 18 organisms. Click here for file

Additional file 4: Figure S1 Phylogenetic analysis of CDK family proteins in H. sapiens, *T. adhaerens*, *C. intestinalis*, *B. floridae*, *S. purpuratus* and *D. melanogaster*. Maximum likelihood analysis was conducted using RAxML program, and Bayesian analysis was carried out using PHYLOBAYES 3.3. Both methods produced trees with nearly identical topologies. The first numbers above branches indicate Bayesian posterior probabilities (only these key branches are labeled), and the second numbers above branches indicate ML bootstrap percentages. The scale bar shows the number of substitutions per site. The sequences of Hsa-GSK3alpha, Hsa-MAK, and Hsa-HCDKL1 were used as outgroup. All proteins are labeled with their accession numbers and their specie name as prefix. Abbreviations: Hsa: *H. sapiens*; Tad: *T. adhaerens*; Cin: *C. intestinalis*; Bfl: *B. floridae*; Spu: *S. purpuratus;* Dme: *D. melanogaster*. Click here for file

Additional file 5: Figure S2 Phylogenetic analysis of CDK family proteins in H. sapiens, *T.* adhaerens, *C.owczarzaki , S. arctica, S.cerevisiae, S.pombe, C. cinerea, S. punctatus, T. trahens* and *D. discoideum.* Maximum likelihood analysis was conducted using RAxML program, and Bayesian analysis was carried out using PHYLOBAYES 3.3. Both methods produced trees with nearly identical topologies. The first numbers above branches indicate Bayesian posterior probabilities (only these key branches are labeled), and the second numbers above branches indicate ML bootstrap percentages. The scale bar shows the number of substitutions per site. The sequences of Hsa-GSK3alpha, Hsa-MAK, and Hsa-HCDKL1 were used as outgroup. All proteins are labeled with their accession numbers and their specie name as prefix. Abbreviations: Hsa: *H. sapiens*; Tad: *T. adhaerens*; Cow: *C.owczarzaki;* Sar*: S. arctica;* Sce*:S.cerevisiae;* Spo*:S.pombe;* Cci*:C. cinerea;* Spu*:S. punctatus;* Ttr*:T. trahens*; Ddi:*D. discoideum.* Click here for file

Additional file 6: Table S1 Evolutionary relationship of CDK family proteins from 18 representative organisms. Summary of the evolutionary relationship of CDK family proteins based on the results of phylogenetic analyses of CDK family proteins (Figure 1, see Additional file 4: Figure S1 and Additional file 5: Figure S2). Click here for file

Additional file 7: Figure S3 Phylogenetic network analysis for CDK4/6, CDK1/2/3, and CDK5 subfamily proteins from *H. sapiens, N. vectensis, T. adhaerens, A. queenslandica, M. brevicollis, and S. rosetta.* Neighbor-Net analysis was conducted using SplitsTree v.4 program [56] with 100 bootstrap resamplings. All proteins are labeled with their accession numbers preceded by their species names. Species abbreviations are as follows: Hsa, *H. sapiens*; Nve, *N. vectensis*; Tad, *T. adhaerens*; Aqe, *A. queenslandica*; MBr, *M. brevicollis.* The alignment used for this analysis is found in Additional file 1: File S3. Click here for file

Additional file 8: Figure S4 Phylogenetic analysis of cyclin family proteins in H. sapiens, T. *adhaerens*, *C. intestinalis*, *B. floridae*, *S. purpuratus* and *D. melanogaster*. Maximum likelihood analysis was conducted using RAxML program, and Bayesian analyses were carried out using PHYLOBAYES 3.3. Both methods produced trees with nearly identical topologies. The first numbers above branches indicate Bayesian posterior probabilities (only these key branches are labeled), and the second numbers above branches indicate ML bootstrap percentages. The scale bar shows the number of substitutions per site. The sequences of Hsa-Cables1 and Hsa-Cables2 were used as the outgroup. All proteins are labeled with their accession numbers and their specie name as prefix. Abbreviations: Hsa: *H. sapiens*; Tad: *T. adhaerens*; Cin: *C. intestinalis*; Bfl: *B. floridae*; Spu: *S. purpuratus;* Dme: *D. melanogaster*. Click here for file

Additional file 9: Figure S5 Phylogenetic analysis of cyclin family proteins in H. sapiens, *T.* adhaerens, *C.owczarzaki ,* and *S. arctica.* Maximum likelihood analysis was conducted using RAxML program, and Bayesian analyses were carried out using PHYLOBAYES 3.3. Both methods produced trees with nearly identical topologies. The first numbers above branches indicate Bayesian posterior probabilities (only these key branches are labeled), and the second numbers above branches indicate ML bootstrap percentages. The scale bar shows the number of substitutions per site. The sequences of Hsa-Cables1 and Hsa-Cables2 were used as the outgroup. All proteins are labeled with their accession numbers and their specie name as prefix. Abbreviations: Hsa: *H. sapiens*; Tad: *T. adhaerens*; Cow: *C.owczarzaki;* Sar*: S. arctica.* Click here for file

Additional file 10: Figure S6 Phylogenetic analysis of cyclin family proteins in H. sapiens, *T.* adhaerens, *S.cerevisiae, S.pombe, C. cinerea,* and *punctatus.* Maximum likelihood analysis was conducted using RAxML program, and Bayesian analyses were carried out using PHYLOBAYES 3.3. Both methods produced trees with nearly identical topologies. The first numbers above branches indicate Bayesian posterior probabilities (only these key branches are labeled), and the second numbers above branches indicate ML bootstrap percentages. The scale bar shows the number of substitutions per site. The sequences of Hsa-Cables1 and Hsa-Cables2 were used as the outgroup. All proteins are labeled with their accession numbers and their specie name as prefix. Abbreviations: Hsa: *H. sapiens*; Tad: *T. adhaerens*; Sce*: S.cerevisiae;* Spo*: S.pombe;* Cci*: C. cinerea;* Spu*: S. punctatus.* Click here for file

Additional file 11: Figure S7 Phylogenetic analysis of cyclin family proteins in H. sapiens, *T.* adhaerens, *T. trahens* and *D. discoideum.* Maximum likelihood analysis was conducted using RAxML program, and Bayesian analyses were carried out using PHYLOBAYES 3.3. Both methods produced trees with nearly identical topologies. The first numbers above branches indicate Bayesian posterior probabilities (only these key branches are labeled), and the second numbers above branches indicate ML bootstrap percentages. The scale bar shows the number of substitutions per site. The sequences of Hsa-Cables1 and Hsa-Cables2 were used as the outgroup. All proteins are labeled with their accession numbers and their specie name as prefix. Abbreviations: Hsa: *H. sapiens*; Tad: *T. adhaerens*; Ttr*:T. trahens*; Ddi:*D. discoideum.* Click here for file

Additional file 12: Table S2 Evolutionary relationship of cyclin family proteins from 18 representative organisms. Summary of the evolutionary relationship of cyclin family proteins based on the results of phylogenetic analyses of cyclin family proteins(Figure 3, see Additional file 8: Figure S4, Additional file 9: Figure S5, Additional file 10: Figure S6, and Additional file 11: Figure S7), and also refer the reciprocal blast search results (detail see material and methods section). Click here for file

Additional file 13: Figure S8 phylogenetic network analysis for Cyclin B like group proteins from *H. sapiens, N. vectensis, T. adhaerens, A. queenslandica, M. brevicollis,* and *S. rosetta.* Neighbor-Net analysis was conducted using SplitsTree v.4 program [56] with 100 bootstrap resamplings. All proteins are labeled with their accession numbers preceded by their species names. Species abbreviations are as follows: Hsa, *H. sapiens*; Nve, *N. vectensis*; Tad, *T. adhaerens*; Aqe, *A. queenslandica*; MBr, *M. brevicollis.* The alignment used for this analysis is found in Additional file 1: File S3. Click here for file
