# Supplementary material for: Phylogenetic analysis of CDK and cyclin proteins in premetazoan lineages
Source: BMC Evol Biol. 2014 Jan 17;14:10. doi: 10.1186/1471-2148-14-10 (PMC3923393; doi:10.1186/1471-2148-14-10)
Supplement: Additional file 1: File S1 — All Multiple alignments of CDK or cyclin proteins which were used for phylogenetic analysis. Multiple alignments of full-length proteins were mainly carried out using MSAProbs program [44], however, the protein alignment which include the cyclin sequence from T. trahens and D. discoideum was carried out using PROMALS program [45], Then the poorly aligned positions in these alignments were removed, only the conserved region (the CDK domain for CDK family, the Cyclin_N domain and Cyclin_C domain for cyclin family) in these alignments were used for further phylogenetic analysis. [file 1471-2148-14-10-S1.doc]

**File S1. All Multiple alignments of CDK or cyclin proteins which were used for phylogenetic analysis.**

Multiple alignments of full-length proteins were mainly carried out using MSAProbs program , however, the protein alignment which include the cyclin sequence from *T. trahens* and *D. discoideum* was carried out using PROMALS program , Then the poorly aligned positions in these alignments were removed, only the conserved region (the CDK domain for CDK family, the Cyclin_N domain and Cyclin_C domain for cyclin family) in these alignments were used for further phylogenetic analysis. Here we list all these alignments.

**Contents:**

1. Multiple alignments for phylogenetic analysis of CDK family proteins in *H. sapiens*, *N. vectensis*, *T. adhaerens*, *A.queenslandica*, *M. brevicollis*, and *S. rosetta*.----------------------------------------------------------------------pages:3-10
2. Multiple alignments for phylogenetic analysis of CDK family proteins in H. sapiens, *T. adhaerens*, *C. intestinalis*, *B. floridae*, *S. purpuratus* and *D. melanogaster*. ------------------------------------------------------------pages:11-19
3. Multiple alignments for phylogenetic analysis of CDK family proteins in H. sapiens, *T.* adhaerens, *C.owczarzaki , S. arctica, S.cerevisiae, S.pombe, C. cinerea, S. punctatus, T. trahens* and *D. discoideum.----------pages:20-29*
4. Multiple alignments for phylogenetic analysis of cyclin family proteins in *H. sapiens*, *N. vectensis*, *T. adhaerens*, *A. queenslandica*, *M. brevicollis*, and *S. rosetta*.-----------------------------------------------------------------------pages:30-37
5. Multiple alignments for phylogenetic analysis of cyclin family proteins in H. sapiens, T. *adhaerens*, *C. intestinalis*, *B. floridae*, *S. purpuratus* and *D. melanogaster*.-----------------------------------------------------------pages:38-46
6. Multiple alignments for phylogenetic analysis of cyclin family proteins in H. sapiens, *T.* adhaerens, *C.owczarzaki ,* and *S. arctica.-------pages:47-52*
7. Multiple alignments for phylogenetic analysis of cyclin family proteins in H. sapiens, *T.* adhaerens, *S.cerevisiae, S.pombe, C. cinerea,* and *punctatus---------------------------------------------------------------------pages:53-60*
8. Multiple alignments for phylogenetic analysis of cyclin family proteins in H. sapiens, *T.* adhaerens, *T. trahens* and *D. discoideum.* ---------pages:61-66
9. Multiple alignments for phylogenetic analysis of CDK4/6, CDK1/2/3, and CDK subfamily proteins in *H. sapiens*, *N. vectensis*, *T. adhaerens*, *A.queenslandica*, *M. brevicollis*, and *S. rosetta*.------------------------------------------Pages67-70
10. Multiple alignments for phylogenetic analysis of Cyclin B like group proteins in *H. sapiens*, *N. vectensis*, *T. adhaerens*, *A.queenslandica*, *M. brevicollis*, and *S. rosetta*.---------------------------------------------------------------------Pages71-75
11. **Multiple alignments for phylogenetic analysis of CDK family proteins in *H. sapiens*, *N. vectensis*, *T. adhaerens*, *A.queenslandica*, *M. brevicollis*, and *S. rosetta*.**

>Hsa-CDK6

YECVAEIGEGAYGKVFKARDLKGGRFVALKRVRVQTGEEGMPLSTIREVAVLRHLEHPNVVRLFDVCTVETKLTLVFEHVDQDLTTYLDKVPEGVPTETIKDMMFQLLRGLDFLHSHRVVHRDLKPQNILVTSSGQIKLADFGLARIYSFQ-MALTSVVVTLWYRAPEVLLQS-SYATPVDLWSVGCIFAEMFRRKPLFRGSSDVDQLGKILDVIGLPGEEDWPRDVALPRQA---FHSKSAQPIEKFVTDIDELGKDLLLKCLTFNPAKRISAYSALSHPYF

>Hsa-CDK4

YEPVAEIGVGAYGTVYKARDPHSGHFVALKSVRVPNGGGGLPISTVREVALLRRLEHPNVVRLMDVCATEIKVTLVFEHVDQDLRTYLDKAPPGLPAETIKDLMRQFLRGLDFLHANCIVHRDLKPENILVTSGGTVKLADFGLARIYSYQ-MALTPVVVTLWYRAPEVLLQS-TYATPVDMWSVGCIFAEMFRRKPLFCGNSEADQLGKIFDLIGLPPEDDWPRDVSLPRGA---FPPRGPRPVQSVVPEMEESGAQLLLEMLTFNPHKRISAFRALQHSYL

>Hsa-CDK2

FQKVEKIGEGTYGVVYKARNKLTGEVVALKKIRLDTETEGVPSTAIREISLLKELNHPNIVKLLDVIHTENKLYLVFEFLHQDLKKFMDASALGIPLPLIKSYLFQLLQGLAFCHSHRVLHRDLKPQNLLINTEGAIKLADFGLARAFGVPVRTYTHEVVTLWYRAPEILLGCKYYSTAVDIWSLGCIFAEMVTRRALFPGDSEIDQLFRIFRTLGTPDEVVWPGVTSMPDYKPS-FPKWARQDFSKVVPPLDEDGRSLLSQMLHYDPNKRISAKAALAHPFF

>Hsa-CDK3

FQKVEKIGEGTYGVVYKAKNRETGQLVALKKIRLDLEMEGVPSTAIREISLLKELKHPNIVRLLDVVHNERKLYLVFEFLSQDLKKYMDSTPGELPLHLIKSYLFQLLQGVSFCHSHRVIHRDLKPQNLLINELGAIKLADFGLARAFGVPLRTYTHEVVTLWYRAPEILLGSKFYTTAVDIWSIGCIFAEMVTRKALFPGDSEIDQLFRIFRMLGTPSEDTWPGVTQLPDYKGS-FPKWTRKGLEEIVPNLEPEGRDLLMQLLQYDPSQRITAKTALAHPYF

>Hsa-CDK1

YTKIEKIGEGTYGVVYKGRHKTTGQVVAMKKIRLESEEEGVPSTAIREISLLKELRHPNIVSLQDVLMQDSRLYLIFEFLSMDLKKYLDSIPPYMDSSLVKSYLYQILQGIVFCHSRRVLHRDLKPQNLLIDDKGTIKLADFGLARAFGIPIRVYTHEVVTLWYRSPEVLLGSARYSTPVDIWSIGTIFAELATKKPLFHGDSEIDQLFRIFRALGTPNNEVWPEVESLQDYKNT-FPKWKPGSLASHVKNLDENGLDLLSKMLIYDPAKRISGKMALNHPYF

>Hsa-CDK5

YEKLEKIGEGTYGTVFKAKNRETHEIVALKRVRLDDDDEGVPSSALREICLLKELKHKNIVRLHDVLHSDKKLTLVFEFCDQDLKKYFDSCNGDLDPEIVKSFLFQLLKGLGFCHSRNVLHRDLKPQNLLINRNGELKLADFGLARAFGIPVRCYSAEVVTLWYRPPDVLFGAKLYSTSIDMWSAGCIFAELANGRPLFPGNDVDDQLKRIFRLLGTPTEEQWPSMTKLPDYKPY--PMYATTSLVNVVPKLNATGRDLLQNLLKCNPVQRISAEEALQHPYF

>Hsa-CDK16

YIKLDKLGEGTYATVYKGKSKLTDNLVALKEIRLEH-EEGAPCTAIREVSLLKDLKHANIVTLHDIIHTEKSLTLVFEYLDKDLKQYLDDCGNIINMHNVKLFLFQLLRGLAYCHRQKVLHRDLKPQNLLINERGELKLADFGLARAKSIPTKTYSNEVVTLWYRPPDILLGSTDYSTQIDMWGVGCIFYEMATGRPLFPGSTVEEQLHFIFRILGTPTEETWPGILSNEEFKTYNYPKYRAEALLSHAPRLDSDGADLLTKLLQFEGRNRISAEDAMKHPFF

>Hsa-CDK17

YIKLEKLGEGTYATVYKGRSKLTENLVALKEIRLEH-EEGAPCTAIREVSLLKDLKHANIVTLHDIVHTDKSLTLVFEYLDKDLKQYMDDCGNIMSMHNVKLFLYQILRGLAYCHRRKVLHRDLKPQNLLINEKGELKLADFGLARAKSVPTKTYSNEVVTLWYRPPDVLLGSSEYSTQIDMWGVGCIFFEMASGRPLFPGSTVEDELHLIFRLLGTPSQETWPGISSNEEFKNYNFPKYKPQPLINHAPRLDSEGIELITKFLQYESKKRVSAEEAMKHVYF

>Hsa-CDK18

YVKLDKLGEGTYATVFKGRSKLTENLVALKEIRLEH-EEGAPCTAIREVSLLKNLKHANIVTLHDLIHTDRSLTLVFEYLDSDLKQYLDHCGNLMSMHNVKIFMFQLLRGLAYCHHRKILHRDLKPQNLLINERGELKLADFGLARAKSVPTKTYSNEVVTLWYRPPDVLLGSTEYSTPIDMWGVGCIHYEMATGRPLFPGSTVKEELHLIFRLLGTPTEETWPGVTAFSEFRTYSFPCYLPQPLINHAPRLDTDGIHLLSSLLLYESKSRMSAEAALSHSYF

>Hsa-CDK14

YEKLEKLGEGSYATVYKGKSKVNGKLVALKVIRLQE-EEGTPFTAIREASLLKGLKHANIVLLHDIIHTKETLTLVFEYVHTDLCQYMDKHPGGLHPDNVKLFLFQLLRGLSYIHQRYILHRDLKPQNLLISDTGELKLADFGLARAKSVPSHTYSNEVVTLWYRPPDVLLGSTEYSTCLDMWGVGCIFVEMIQGVAAFPGMKDIDQLERIFLVLGTPNEDTWPGVHSLPHFKPERFTLYSSKNLRQAWNKLVNHAEDLASKLLQCSPKNRLSAQAALSHEYF

>Hsa-CDK15

YLNLEKLGEGSYATVYKGISRINGQLVALKVISMNA-EEGVPFTAIREASLLKGLKHANIVLLHDIIHTKETLTFVFEYMHTDLAQYMSQHPGGLHPHNVRLFMFQLLRGLAYIHHQHVLHRDLKPQNLLISHLGELKLADFGLARAKSIPSQTYSSEVVTLWYRPPDALLGATEYSSELDIWGAGCIFIEMFQGQPLFPGVSNIEQLEKIWEVLGVPTEDTWPGVSKLPNYNPEWFPLPTPRSLHVVWNRLVPEAEDLASQMLKGFPRDRVSAQEALVHDYF

>Hsa-CDK11B

FQCLNRIEEGTYGVVYRAKDKKTDEIVALKRLKMEKEKEGFPITSLREINTILKAQHPNIVTVREIVVGMDKIYIVMNYVEHDLKSLMETMKQPFLPGEVKTLMIQLLRGVKHLHDNWILHRDLKTSNLLLSHAGILKVGDFGLAREYGSPLKAYTPVVVTLWYRAPELLLGAKEYSTAVDMWSVGCIFGELLTQKPLFPGKSEIDQINKVFKDLGTPSEKIWPGYSELPAVKKMTFSEHPYNNLRKRFGLLSDQGFDLMNKFLTYFPGRRISAEDGLKHEYF

>Hsa-CDK10

FEKLNRIGEGTYGIVYRARDTQTDEIVALKKVRMDKEKDGIPISSLREITLLLRLRHPNIVELKEVVVGLESIFLVMGYCEQDLASLLENMPTPFSEAQVKCIVLQVLRGLQYLHRNFIIHRDLKVSNLLMTDKGCVKTADFGLARAYGVPVKPMTPKVVTLWYRAPELLLGTTTQTTSIDMWAVGCILAELLAHRPLLPGTSEIHQIDLIVQLLGTPSENIWPGFSKLPLVGQYSLRKQPYNNLKHKFPWLSEAGLRLLHFLFMYDPKKRATAGDCLESSYF

>Hsa-CDK9

YEKLAKIGQGTFGEVFKARHRKTGQKVALKKVLMENEKEGFPITALREIKILQLLKHENVVNLIEICRTKGSIYLVFDFCEHDLAGLLSNVLVKFTLSEIKRVMQMLLNGLYYIHRNKILHRDMKAANVLITRDGVLKLADFGLARAFSLQPNRYTNRVVTLWYRPPELLLGERDYGPPIDLWGAGCIMAEMWTRSPIMQGNTEQHQLALISQLCGSITPEVWPNVDNYELYEKLELVKGQKRKVKDRLKARDPYALDLIDKLLVLDPAQRIDSDDALNHDFF

>Hsa-CDK13

FDIIGIIGEGTYGQVYKARDKDTGEMVALKKVRLDNEKEGFPITAIREIKILRQLTHQSIINMKEIVTDKGAFYLVFEYMDHDLMGLLESGLVHFNENHIKSFMRQLMEGLDYCHKKNFLHRDIKCSNILLNNRGQIKLADFGLARLYSSESRPYTNKVITLWYRPPELLLGEERYTPAIDVWSCGCILGELFTKKPIFQANQELAQLELISRICGSPCPAVWPDVIKLPYFNTMKPKKQYRRKLREEFVFIPAAALDLFDYMLALDPSKRCTAEQALQCEFL

>Hsa-CDK12

FDIIGIIGEGTYGQVYKAKDKDTGELVALKKVRLDNEKEGFPITAIREIKILRQLIHRSVVNMKEIVTDKGAFYLVFEYMDHDLMGLLESGLVHFSEDHIKSFMKQLMEGLEYCHKKNFLHRDIKCSNILLNNSGQIKLADFGLARLYNSESRPYTNKVITLWYRPPELLLGEERYTPAIDVWSCGCILGELFTKKPIFQANLELAQLELISRLCGSPCPAVWPDVIKLPYFNTMKPKKQYRRRLREEFSFIPSAALDLLDHMLTLDPSKRCTAEQTLQSDFL

>Hsa-CDK7

YEKLDFLGEGQFATVYKARDKNTNQIVAIKKIKLGHAKDGINRTALREIKLLQELSHPNIIGLLDAFGHKSNISLVFDFMETDLEVIIKDNSLVLTPSHIKAYMLMTLQGLEYLHQHWILHRDLKPNNLLLDENGVLKLADFGLAKSFGSPNRAYTHQVVTRWYRAPELLFGARMYGVGVDMWAVGCILAELLLRVPFLPGDSDLDQLTRIFETLGTPTEEQWPDMCSLPDYVT--FKSFPGIPLHHIFSAAGDDLLDLIQGLFLFNPCARITATQALKMKYF

>Hsa-CDK19

FEYECKVGRGTYGHVYKARRKDGKKEYALKQI----EGTGISMSACREIALLRELKHPNVIALQKVFLSDRKVWLLFDYAEHDLWHIIKFHPMQLPRSMVKSLLYQILDGIHYLHANWVLHRDLKPANILVMERGRVKIADMGFARLFNSPLKPLDPVVVTFWYRAPELLLGARHYTKAIDIWAIGCIFAELLTSEPIFHCRQEHDQLDRIFSVMGFPADKDWEDIRKMPEYPTLQFRRTANSSLIKYMVKPDSKVFLLLQKLLTMDPTKRITSEQALQDPYF

>Hsa-CDK8

FEYECKVGRGTYGHVYKAKRKDGKKDYALKQI----EGTGISMSACREIALLRELKHPNVISLQKVFLSDRKVWLLFDYAEHDLWHIIKFHPVQLPRGMVKSLLYQILDGIHYLHANWVLHRDLKPANILVMERGRVKIADMGFARLFNSPLKPLDPVVVTFWYRAPELLLGARHYTKAIDIWAIGCIFAELLTSEPIFHCRQEHDQLDRIFNVMGFPADKDWEDIKKMPEHSTLMFRRNTNCSLIKYMVKPDSKAFHLLQKLLTMDPIKRITSEQAMQDPYF

>Hsa-CDK20

YCILGRIGEGAHGIVFKAKHVETGEIVALKKVALRRLEDGFPNQALREIKALQEMENQYVVQLKAVFPHGGGFVLAFEFMLSDLAEVVRHAQRPLAQAQVKSYLQMLLKGVAFCHANNIVHRDLKPANLLISASGQLKIADFGLARVFSPDSRLYTHQVATRWYRAPELLYGARQYDQGVDLWSVGCIMGELLNGSPLFPGKNDIEQLCYVLRILGTPNPQVWPELTELPDYNKISFKEQVPMPLEEVLPDVSPQALDLLGQFLLYPPHQRIAASKALLHQYF

>Hsa-CDKL1

YEKIGKIGEGSYGVVFKCRNRDTGQIVAIKKFLESEDDPVIKKIALREIRMLKQLKHPNLVNLLEVFRRKRRLHLVFEYCDHTVLHELDRYQRGVPEHLVKSITWQTLQAVNFCHKHNCIHRDVKPENILITKHSVIKLCDFGFARLLTGPSDYYTDYVATRWYRSPELLVGDTQYGPPVDVWAIGCVFAELLSGVPLWPGKSDVDQLYLIRKTLGDLIPRHQQVFSTNQYFSGVKIPDEDMEPLELKFPNISYPALGLLKGCLHMDPTQRLTCEQLLHHPYF

>Hsa-GSK3alpha

YTDIKVIGNGSFGVVYQARLAETRELVAIKKVLQDK------RFKNRELQIMRKLDHCNIVRLRYFFYSKLYLNLVLEYVPETVYRVARKAKLTIPILYVKVYMYQLFRSLAYIHSQGVCHRDIKPQNLLVDPTAVLKLCDFGSAKQLVR-GEPNVSYICSRYYRAPELIFGATDYTSSIDVWSAGCVLAELLLGQPIFPGDSGVDQLVEIIKVLGTPTREQIREMN--PNYTEFKFPQIKAHPWTKVFKRTPPEAIALCSSLLEYTPSSRLSPLEACAHSFF

>Hsa-MAK

YTTMRQLGDGTYGSVLMGKSNESGELVAIKRMKRKF-YSWDECMNLREVKSLKKLNHANVIKLKEVIRENDHLYFIFEYMKENLYQLMKDRNKLFPESVIRNIMYQILQGLAFIHKHGFFHRDMKPENLLCMGPELVKIADFGLARELRSQ-PPYTDYVSTRWYRAPEVLLRSSVYSSPIDVWAVGSIMAELYMLRPLFPGTSEVDEIFKICQVLGTPKKSDWPEGYQLASSMNFRFPQCVPINLKTLIPNASNEAIQLMTEMLNWDPKKRPTASQALKHPYF

>Tad-gi|196001971|

YKKIKLLGEGQFAVVYQAEDTHKNKIVAVKKIKLGPANDGINRTALREIKLLQELKHENIIGLLDVFGHKSNISLVFDYMEADLEVIIKDSSIILTNAHIKQYIIMTLRGLEYIHSNWILHRDMKPNNLLIDSNGILKLADFGLARYFGSPNRYYTNQVVTVWYRAPELLFGANSYGTGVDMWAVGCILAELLLRVPFLPGDSDLDQLTRIFQTLGTPIEDKWADIHKLPGYIK--FKTLPAIPLNEIFTAASDDMLDLLRRLFEYNPSSRITATEALQMKYF

>Tad-gi|195996663|

FTILGRVGEGAHGVVFKAKHVGNGEVVALKKIHLKKIDDGIPNNILREIKTLQAITHENIVRLFDVFPDGSSLVLAFEYMVTDLSEILRSSQNTLPE-VSSYYMIMLLRGISFCHENHIIHRDLKPANLLISSSGQLKLADFGLARVMSVEERLYSHQVATRWYRAPELLYGSRTYDEGVDLWYLGCIFAELINKSPLFPGDSDIKQLGCVLSILGTPTTSTWPGLIELPDYNKITFSGFLPIPFESIVPDATSEVGKIK-----------------------

>Tad-gi|196013334|

FEWLNRIEEGTYGVVYRAKDLKSDEVVALKRLKMEKEREGFPITSLREINTLLKADHPNIVHVREIVVGMDKIYIVMEYVEHDLKTLMESMSQPFSISEVKCLMKQLLSAVQHLHDNWILHRDLKTSNLLLSHQGILKVGDFGLAREYGSPLKVYTSIVVTLWYRCPELLLGVKEYSTAVDMWSVGCIFGEFLVKKPLFPGKSEIDQLNKIFKDLGTPNDQIWSGFSELPVAKKVTFTEQPYNRLRDRFGYLTDQGFDLLNRFLTYDPKKRISAEDALNHEYF

>Tad-gi|196010283|

FDKLNRIGEGSYGVVYRARDLDSKEIVAIKKIRMENERDGIPVSSLREITLLVNLKHINIVNLKDVVVGLDSIFLVMEYCEQDLSSLLYNMKAPFTEPQVKCLSLQLIHGVQYLHHNFVIHRDLKVSNLLLTDKGILKVADFGLARNYGLPAAPMTPTIVSLWYRAPEVLLGCTKHTLAVDMWSVGCIIAELFDHNVFLKGKSEKDQLDLMCQMLGTPNEAIWEDIRDMPLYGKIILRQQPYNNLKHKFSWLSAAGLNLLNSLLTYDPGRRITADETLKLSYF

>Tad-gi|196002669|

--ILKQIGEGTYGQVFKAKIKDTDKLVALKKVRTDHEREGFPITAVREIKILKQLNHQSIVNLLGIVSNRCAFYLVFEYMDHDLMGLLESGLVTFDEEHIRSFMRQIMEGLNYCHKRQLLHRDIKCSNLLMNNKGQIKIADFGLARFYNPKSRPYTNKVITLWYRPPELLLGEERYGPSVDVWSCGCILGEFFTKKPIFQANSEINQLDLISQICGTPCPENWPSVVELPYYNNFKLRKY-ERRLEQEFHDLPELAVDLMQYMLILDPSMRYNAEQSLQHPFI

>Tad-gi|195998898|

YEKITKIGHGTFGEVFKARNRISKEFVALKRVLLGNEKEGFPITSLREIKILRALKHDNIVRLQEICRSRGSIYLVFEFCAHDLAGLLQNPQVKFNLSEIKRMMKHLLSGLFYIHSNKVLHRDLKAANVLVTRDGVLKLADFGLARVYSRKTHCFTNRVVTLWYRAPELLLGCRDYGPAIDMWAIGCIMAEFWTRSAIMQGNSETNQLTLITQLCGSITPEVYPDVDKLDLFKKFDLPASQKRRVKERLSHRDRHALDLIDRCLTIDPAKRIDSDSALNHDFF

>Tad-gi|196013348|

FHKLEKIGEGTYGKVYKACNKITGQTVALKKIRLDSDKEGVPSTTLREISILRSLNHSFVVRLYDVVHSDQCLYLVFEYLDHDLKHYLDHA-YKIPPALLKSYLYQMLRAISYCHSRRVLHRDLKPQNLLIDSTGTLKLADFGLARIFGLPVRQYTHEVITLWYRAPEILLGSTYYSTPVDIWSIGCIFVEMINRRPLFAGDSEIDQLFRVFRTLGTPDEITWPGVSEMSDYKST-FPKWPSRDLNSVIYSHDEDCVDLIKQMLVYEPNGRISARLALQHPYF

>Tad-gi|196003954|

YLKIEKIGEGTYGVVYKGKNRNTQQLVALKKIRLENEEEGIPSTAIREVSLLKELKHPNIVDLIEVLYEESKLYLVFEFLDMDLKRYLDTLPKTIDAMLMKSYLYQILLGVVYCHSHRVLHRDLKPQNLLINSKGCIKLADFGLGRAFGVPVRVYTHEVVTLWYRAPEVLLGSTRYSCPLDIWSTGTIFAEMWLRRPLFQGDSEIDELFRIFRILGTPDDDIWPGVSSLPEFKSS-FPKWSKQSYDTFVPNMSESGIDLLSKMLIYDPANRISGKRALSHPYF

>Tad-gi|196001193|

YEKLEPLGEGSYATVYKGYSIGHQKLVALKEITLNE-EEGTPFTAIREASLLKQLKHANIVVLHDIIQTPTKLTFVFEYVTTDLSQYLNLHPGGLNMKNVKLFLYQLLRGLSYCHQRRILHRDIKPQNILVSEIGEIKLADFGLARAKSVPSKTYSPDVVTLWYRPPDVLLGSTNYSTSLDIWGVGCIFTEMVSGIATFPGVRNIDQLDKIWHKLGTPTEETWPGVTSYPEYCAAETMFYESRTIAEVIPRLDPGAADLATKMLQYQPFKRIFCQAAMNHQYF

>Tad-gi|195996637|

YDKLHKLGEGTYATVFKGRSKLTNDFVALKEIRLEH-EEGAPCTAIREVSLLKDLKHANIVTLHDTIHTERSLVLVFEYLDRDLKQYMDSCGSILDMSNVKIFLFQLLRGLAYCHSRRVLHRDLKPQNLLINERGELKLADFGLARAKSVPSKTYSSEVVTLWYRPPDVLLGSTEYSTSIDMWGVGCIFYEMATGLPMFPGSSTENQLQTIWEILGTPTEEEWSGLTRNLKVNSLSFHDCKGEPLRNRAPRLEADGLDLLAKFLQYKAKSRISSADAMKHKYF

>Tad-gi|196000717|

YEKLVKIGEGTYGTVYKAVNHDTGEIVALKKVRIDDENEGIPSFALREICLLKELKHKNIVMLYDVIHGNKELMIVFEYCDQDLKRYCDACQGKIDPSIVQSFTNQLLQGLAYCHSHHILHRDITPQNILVTGNGDIKLADFGLARNFGIPVKSFSAEVVTLWYRSPDVLLGATLYDTSIDIWSTGCIFAELSNGQPLLPGKDVADQLKIIFKIFGTPNEQIWPGVSQLMKDKDY--PSYNAMSILHVVPNLNQLGCDLFQLMMVLDPSKRCTAEQALQHAYF

>Tad-gi|195999760|

YVQVTEIANGAYGRVYKARDLNHNRLVALKKIAVINDEQGIPISTIREITSLKSLGHQNIVRLYDIFANTMDLTLVFEHVEQDLQTYIRNCPSGIDTRKIKDIIYQIVNAIDFLHANRIVHRDLKPQNVLISRQGIVKVADFGLAKVFCEN-VPITSVVVTLWYRCPEVLLQS-TYATPVDMWSVGCIMAELYLREPLFCGQTDIDQLQKIFSMTGLPDESEWPVNIPFSRST---FCQYTRRQYSEMMPEICQDGVDLLQKLLKFNPKERLTAEESLQHPYF

>Tad-gi|196015356|

FEFECKVGRGTYGHVYKAKMKSSGKEYALKLI----EGSGISMSACREIALLREVHHPNVISLQGVFLTNRKVWLLFDFAEHDLWHIIKYHAISLDSKMVKSLLKQILEGIHYLHANWILHRDLKPANILVMERGRVKIADMGFARHYWSPLKPLDPVVVTSWYRAPELLLGARHYTKAIDIWAIGCIFAELLTSEPIFHCRQERDQLERIFMVMGYPHEKDWEDIKKTPNYAKLAFRKITNCSLAKYMIKQDSKEFILLQKCLTIDPNKRISSEQAIDDAYF

>Tad-gi|196015366|

FDFECKVGRGTYGHVYKAKMKTSGKEYALKLI----EGTGISMSACREIAILREISHTHVISLQGVFLTSRKVWLLLDFAEHDLWHIIKFHSVPIDTKIVKSLLRQILDGIQYLHSNWILHRDLKPANILVMERGRVKIADMGFARHFWAPLKPLDPVVVTFWYRAPELLLGARHYTKAIDIWAIGCIFAELLTSEPIFHCRQEHDQLDRIFTVMGFPHERDWEDIKIMPEYKRLQFKKPASCSLAKYMIRHDSREFSLLQKFLIADPNKRISAELAMDDAYF

>Nve-gi|156349526|

FEKLNRIGEGTYGIVYRAKDTKSGKIVALKKVRMEQERDGIPISGLREITLLLNLRHENIVQLLEVVVGLDSLFLSMEYCEQDIASLLDNMSCPFSEAQIKCLMIQLLEGTKYLHEHFIVHRDLKVSNLLLTGKGVLKIADFGLARTFGYPYKPMTPVVVTLWYRSPELLLGAKVHTTAVDMWAVGCIFGELLGNKPLLAGKSEINQLQLIVDLLGTPNDHIWPGYSSLPGVKSISLKHQPYNNLKHKFSWVSQAGLSLLNYMLMYDPCKRATAAESLQSSYF

>Nve-gi|156383566|

FQWLNRIEEGTYGVVYRAKEKASGEVVALKRLKMEKEKEGFPITSLREINTLLKAQHPNIVHVREIVVGMDRIYIVMDYVEHDLKTLMEHMTSPFTVGEVKTLLIQLLRATAHLHDNWILHRDLKTSNLLLNNRGILKVGDFGLAREYGSPLRHYTPIVVTLWYRAPELLLGIKEYSCPIDMWSVGCIFAELLTMEPLFPGRSEIDQINRIFKELGTPSDKIWPGPSELPHVKKMTFTEYPYNQLRNRFGYLTDKGFSLLNRFLTYDPKKRITAETALKEDYF

>Nve-gi|156398520|

FDIIEQIGEGTYGQVYKAKDKITGELVGLKKVRTDNEKEGFPITAVREIKILCQLNHPNIINLKEIVTDKGAFYLVFEYMDHDLMGLLESGLVHLTEDHIKSFIRQLLDGLNYCHKKNFLHRDIKCSNILLNNKGEIKLADFGLARLYEAERRPYTNKVITLWYRPPELLLGEERYGPGIDIWSVGCILAELFTKKPIFPAYQEIGQLELISRVCGTPTPAVWPSIINLPHFHSIKPKRQYRRRIREEFNFLPEDALDLFDAMLTLDPSQRITAEKALEHPFL

>Nve-gi|156365870|

YERQAKIGQGTFGEVFKAKNRKNKEIVALKKVLMDNEKEGFPITALREIKILQLLNHENVVKLLEICRTKASIYLVFEFCEHDLAGLLNNQAVKFSPPEMKKIMQMLLNALYFIHSNKILHRDMKAANILITKNGVLKLAEFGLARAIHIQKQRYTNRVVTLWYRPPELLLGERNYGPPIDLWGAGCIMAELWTRTPIMQGNTEQHQLTLISHLCGSITPEVWPGVDKLELFDKMVLPSGQKRRVKERLRMKDHNALDLIDKMLSLDPGPRIDADSALNHDYF

>Nve-gi|156360882|

YKKIDFLGEGQFATVFKAEDQETGNIVAVKKIKLGNAKDGINRTALREIKLLQELSHENVIGLLDVFGHKSSISLVFPFMETDLEVLIKDTCIVLSPADVKSFTIMTLKGLEYLHSHWILHRDMKPNNLLLDDKGVLKIGDFGLARAFGSPSRVYTHQVVTRWYRPPELLFGARIYGTGVDMWAEGCILAELLLRVPFLPGATDLDQLSRTFQTLGTPTEETWPGMTSLPDYVE--FKPFPGIPLKDIFSAAGDDMLDLLDRLLDCNPNGRVNATQALNMPYF

>Nve-gi|156408568|

YTILGRIGEGAHGIVFKAKHIESGEIVALKKVPLRRLEDGIPNTALREIKSLQENENPYVVKLIDVFPHGTGFVLVFEYMWSDLSEVLRNSERPLTEAQIKGYLLMLLKGVAYCHNKGIMHRDLKPANLLISSTGHLKIADFGLARVFSNEERQYSHQVATRWYRAPELLYGARKYDEGVDLWAVGCIFGELLNNSPLFPGENDIEQLCCVLKTLGTPNEEIWPGMTDLPDYNKITFPDMPAIPLEKIVPDASPEAMDLLKRFLVYPSKKRIPASEALLHPYF

>Nve-gi|156385093|

FEKVEKIGEGTYGVVYKARDKTTGRMVALKKIRLDTESEGVPSTAIREISLLKELNHPNVVSLLDVVHNQKSLYLVFEFLSQDLKKYMDCLPPGISTSLIKSYVYQLLSGVAYCHSHRVLHRDLKPQNLLIDKNGAIKLADFGLARAFGVPVRSYTHEVVTLWYRAPEILLGSRYYATPVDVWSIGCIFAEMKTRRALFPGDSEIDQLFRIFRTLGTPDDKVWPGVSELPDYKTS-FPKWPVQSIRHVLPTLDNTAIDLLQKMLTYQPNARISAKAALSHEFF

>Nve-gi|156391793|

FSKIEKIGEGTYGVVYKAKNLKTGGFAALKKIRLEVEDEGIPSTAVREISLLKELRHPNVVELQHILHQEPKLYLVFEYLTCDLKKHLDTTRGMLDKTLVKSYLYQITNAIYFCHARRILHRDLKPQNLLIDSKGLIKLADFGLGRAFGIPVRAYTHEVVTLWYRAPEVLLGGQRYSCPIDVWSIGTIFAEMVTKRPLFHGDSEIDQLFRIFRILGTPTEETWKGVTSLPDYKPT-FPKWAGDGLKKAVPQLDSDGLDLLKKMLIYDPALRISAKTSLKHPYF

>Nve-gi|156407302|

YDKLEKIGEGTYGTVFKGKNKETREILALKRVRLDDDDEGVPSSALREICLLKELKHNNIVRLYDVLHSEKKLTLVFEFCDQDLKKYFDSCQGEVDASVVKSFMFQLLRGLAFCHSHNVLHRDLKPQNLLINKDGELKLADFGLARAFGIPVRCFSAEVVTLWYRPPDVLMGAKLYSTSIDMWSAGCIFAEMANGRPLFPGNDVDDQLRRIFKILGTPTEESWPNVSKLPDYKEF--PPQPSVSLGMVVPKLSSTGRDLLQKLLVSNPAHRISAEDAMKHAYF

>Nve-gi|156392090|

YQKLEPLGEGSYATVFKGLCTANKKIVALKQIRLQE-EEGAPFTAIREASLLKQLKHGNIVKLHDIIHTKDTLMFVFEFLDTDLNCYLEKYSRGICPHNTQLFCFQLLRGLAYIHDRKILHRDIKPQNLLISERGELKLADFGLARAKSVPSQTYSHEVVTLWYRPPDVLLGSKNYTTSLDIWGAGCIFVEMLTGIALFPGLNHIDQLNKIWQVLGTPTDETWPGVSKLPEYDPDIFINFRPRRIGQCIPRLVEGAEQLVIRMIQLDPKNRISAREAMRSTYF

>Nve-gi|156403969|

YTKLDKLGEGTYATVFKGKSKLTDNIVALKEIRLEH-EEGAPCTAIREVSLLKGLKHANIVTLHDTVHTQKSLTLVFEYLEKDLKQYMDDCGGIMSMNNVRIFLFQLLRGLDYCHKRKVLHRDLKPQNLLINDKGELKLADFGLARAKSVPTKTYSNEVVTLWYRPPDVLLGSTAYSTQIDMWGVGCIFFEMATGRPLFPGSTVEDELLLIFKVLGTPSEEVWPGISANEAFIAGKFPDYPRENLIIHAPRLDNSGLELLEKFLEYTVKDRVSAHDAMRHDYF

>Nve-gi|156374066|

YEEVAEIGTGAYGTVYKAKDLLDGKFVALKRVRIQNSEEGMPLSTIREIALLKQIAHPNVVRLLDIFHIETHLNLVFEHVDQDLAAYLEYCPQGLGEWKIKDLTYQILNGVDFLHTHRIVHRDIKPQNILVTKDGQVKIADFGLARVYKDA-MALTSVVVTLWYRAPEVLLQS-SYATSVDIWSVACILAELFNRRPLFEGKNDVDQLDKIFSIIGSPSQDEWPQNVSLPWTS---FSRYTTGSFQALVPEMCTEGTTLLKEMLQFLPRSRPSASEAMNHPFF

>Aqu-gi|340373367|

YRKLDILGEGQFATVYRAEDTERNAIVAVKKIKIGQARDGINRTALREIKLLQELHHPHILGLLDVLGHKSNISLIFDFMDTDLEMIIKDKSLLLPPGVVKAYSLMILQGLEYLHAHWILHRDLKPNNLLINKDGVLKITDFGLAKAYGSPNRIMTHQVVTRWYRSPELLFGARLYSTGVDLWAVGCIIAEMLLRLPFLPGETDLGQLSTIFEFFGSPNEENWPSVKSLPDYVE--FKPSPPQSFRDVFSAAGDDLIHLLESCMKLDPSKRCTASQALQSSYF

>Aqu-gi|340381980|

YKILGRIGEGAHGVVLKARHTQTGDLVALKRVHLKKPADGIPNSALREIKALQESGNHHVICLRDMFPHGPGFVLVFDYMLSDLAEVIRNAEKPLTEAQVKSYMTMLLKGVAYLHDNKIMHRDLKPANLLISQTGHLKIADFGLARVLSTEGRLYSHQVATRWYRAPELLYGARQYDTGIDMWAVGCIFGELLNTSPLFPGENDIDQLCCVLRILGTPSERIWPGMSQLPDYHKISFSEMSPTPMEVVVPDALPEAVDLLKSFLVYDSRHRLSAAKALLHSYF

>Aqu-gi|340371337|

FNKLNRIGEGTYGVVYRAEDKKSKEIVALKRIRMENEEEGLPICSVREIGLLLSLSHENIVQLKEIAVGLDNMFLVMNYCEQDLASLIDNMASPFTEPQVKCIMLQLLEGLSYLHNNHVIHRDLKVSNLLLTDKGILKIADFGLARTLGRPLKPLTPTVVTLWYRAPELLFGSREYSCSLDMWSVGCIFGELLLNKPLLPGKSEANQIELITNLIGSPNEGIWPGYSKLPLVASLEIKRQPYNNLKEKVYWISETGRGLLNDLLTYNPEYRMSSSRALRCKYF

>Aqu-gi|340369066|

YEKLEKLGEGTYATVYKGKSNITGKLVALKEIRLEH-EEGAPCTAIREVSLLKDLKHANIVFLHDIIHTARSLTLIFEYVEQDLKQYLDQCSGMMAMPNVKLFLFQLMRGLQYCHSRKILHRDLKPQNLLISEQGDLKLADFGLARAKSVPTKTYSNEVVTLWYRPPDVLLGSIDYADSIDMWGVGCIFYEMIVGRPMFPGANVEEELVLIWKSLGTPNEKTWPGITKNKEFISHSFLRYDPQPLGLIVPRLDKEGINLMSKLLSYESQERLLARDGMKHNYF

>Aqu-gi|340384408|

YERLEKIGEGTYGTVFKAKEKESGEIVALKIVRLDEDDEGVPSAALREICLLKELKHKNIVRLTDVLHKNLKLTMVFEYIDQDLKKYFDVSGGIISPQVVQSFFFQLLQGLAFCHYNNILHRDLKPQNILISKKGDLKLADFGLARAFGIPVRLFSAEVVTLWYRPPDVLMGAQVYNTSIDMWSAGTIFAELANGRPLFPGSDVDEQLKRIFKLVGTPTERSWPGLTKLPEFKEF--PPYPPACIESVVPALNDAGVDLLQRHLICHPTERISAEEAMRHEYF

>Aqu-gi|340379293|

YERLEKIGEGTYGVVYKARQATHGRVVALKKIRLDAECEGVPSTAIREISILKELDHVNIVSLLDVLYCDRKLFMVFEFLDYDLKKYMDRHAP---TGIPTDYLYQLLEGVAYCHAHRVLHRDLKPQNLLISSDGRIKLADFGLARAFGVPVRTYTHEVVTLWYRSPELLLGSQYYSTPVDIWSIGCIFAEMVTKRPLFPGDSEIDQLFRIFRTLGTPDESVWPGISSFPDYKSS-FPKWPRQNLQRIVKSLDTLGINLLEQMLCYEPCKRITAINGMRHPFF

>Aqu-gi|340381019|

YTKLEKIGEGTYGVVYKARHKVTGKTVALKKIRLENEEEGVPSTAIREISILKEVQHTNVVKLEDIIHQDLKLYLVFEFMCMDLKKYLDSLPAFMEPDLVKSYTYQILKGIVFCHGRRIIHRDLKPQNLLIDNNGGIKIADFGLGRAFGIPVRAYTHEVVTLWYRAPEVLLGCPRYSCPLDIWSIGCIFAEMSNKKPFFQGDSEIDQLFRIFRILGTPSDAIWPSVTTMPNFKST-FPKWTGKSLSQLCPHLDSIGCDLLMQMVIYNPGKRISAKRAMEHPYF

>Aqu-gi|340383363|

YEWLNRIEEGTYGVVFRARDIRTDEIVALKKLKMEKEREGFPITSLREISTLLKANHENIVNVREIVVGMDKIFIVMDYVEHDLKSLMETMKQPFLEGEVKTLLIQLLKAVHHLHDNWIIHRDLKTSNLLLSHKGILKVADFGLAREYGSPLKNYTPIVVTLWYRAPELLLGAKEYSTAIDVWSVGCIFAELLQHKPLFMGKSEIDELNLIFKELGVPNESIWPGFGELPVAKKVQFTQQPLNNLRKRFPMITKNGFVLLNKFFAYDPKRRVTAEDALKHEYF

>Aqu-gi|340377126|

YEKLTKVGQGTFGEVFKAKDRKTGRLVALKKVCMENEKEGFPMTALREIRILQLLQHNNIVNLVEICRSKGSIYLVLDFCEHDLAGLLECKEIKFSLSEIKNIMQQLFNALAYIHGNNILHRDMKSCNILVTRKGELKLADFGLARALNKANQRYTNRVVTLWYRPPELFLGERNYGPPIDMWGAGCIMAEMWTRRPIMQGDTEQKQITLICQLCGSISPTEWAGVEKLEYYQKLELPQKENRKLKERLRHEDPYALDLIDKLLMLDPRKRIDADSTLEHDFF

>Aqu-gi|340369314|

FEILSQIGEGTFGKVYKAKDLKTGEVIALKKVLIDSEREGFPITAVREIKILRQLRHENIVTLKEIISDSSSFYLVFEYCAHDLMGLIDSGMVVFSESHIQSLMRQLMEALCYCHSKNFLHRDLKCSNILINNKGQLKLGDWGLARYYFAHSRLYTNHVITLWYRPPELLLGAEHYGPAVDIWSCGCILGELFTKKPLFHGSIEMEQLDAISRVCGTPTPANWPEVIKLPLFQTFKFKKLYRRRVKEEYSNIPEVPLDLLDKLISIDPSKRISSEEALNHPFL

>Aqu-gi|340380476|

FEYEKKIGRGTYGHVYKAKKRSGDKEYALKQI----EGTGISMSACREIALLRELKHSNVISLQRVFLSDRKVWLLFDYAEHDLWHIIKYHQVQVPKTMVKSLMYQILSGIHYLHSNWVLHRDLKPANILVMERGRVKIADMGFARLFNAPLKPLDPVVVTFWYRAPELLLGAKHYTKAIDIWAIGCIFAELLTFEPIFHCRQEREQLERIFLVMGFPHDRDWDDMKHMPEYSTLMFGRQANCALQKYMVKHDTSAFKLLTKFLVMDPNKRITSDQAMLDTYF

>Mbr-gi|167536606|

YEKVKYLGEGQFGTVYLERDSQTGQLYAIKRIRLGDAKEGLNQSAFREIMFLREVHHPNIIDLHDVFLKKGNLHLVLELASTDLEKLIRNKRLDFAPGDVKSLLLQTYQALDYLHARWILHRDLKPNNILITTGGQVKLTDFGLACTFGSPSREMTTQVVTIFYRAPELLLGARHYGVGVDIWAMACIHMELELRTPILPGDGPFDQLDKIMAFLSSFGHAAQPNYRELSSSWE--FKPRDPTPIRALLPAVSDAAIDLLEKQFTYDPLKRPTARETLMHPYF

>Mbr-gi|167516210|

FRILGRIGEGAHGVVFKAKHIESGVVVALKRVGLKRIEAGIPVALLREIQALRHVQHPNVVRLHDAFAH------------------------------------------AACVLRGIIHRDLKPSNLLISPQGQLKIADFGLARIWHQDGRQYSHQVATRWYRAPELLFGARHYDLGVDLWAVGCIFAEMINSAPLFPGENDIDQLSCVLHTLGTPTPENWPEASTFPDYGKITFDETPGVPPEDLVRNTSPEGRTLFASFVPYSAKRRQSARSALRSLYF

>Mbr-gi|167517533|

YLKIEKIGEGTYGTVYKAKVKATGNLVALKKIKLEAEEEGVPSTAIREISLLKELSHPNVVSLMEVIHSENKLYLVFEFLDQDLKKHIDSQRNGLSMELIKSYMLQLLKGIDFCHARRILHRDLKPQNLLINREGFIKLADFGLARAFGIPIRAYTHEVVTLWYRAPEILLGQRQYACPVDMWSIGCIFAEMVTRRPLFPGDSEIDELFRIFRVLGTPTEQTWPGVSQLPDYKDC-FPRWSGEGLASLIPGLDAMGLDLLQKMLRYEPSQRISARQALTHPWF

>Mbr-gi|167522771|

YAKIDKLGEGTYGVVFKARDRHDGSIVALKRISLESAAEGVPSNAVREISLLKSLHHPNIVRLYDVLHSEHKLTMVFEYCDQDLKKFLDSCRGTPEHHVIQSFMFQLLQGIRHCHEERVLHRDLKPQNLLINKRGQLKLADFGLARPYGVPVRSYSHEVVTLWYRAPDVLLGATGYDTSIDMWSAGCILAEMANGSPLFPGTSVQDQLDLIFRVLGTPTIESWPGLHELPNYSGPFLPHVDGVGLEAEVSSLFPEGLDLLQQLLRYVPDERLSADRALRHRFF

>Mbr-gi|167522415|

YKKLHKLGEGTYATVFKGISHINGKIVALKEIRLEH-EEGAPCTGIREVSLLKGLKHANIVTLHDVIHTKDNLIMVFEFLSKDLKAYMDDCNSYIDLRNAKLFLFQLLRGVGFCHSRKVLHRDLKPQNLLINHAGELKLADFGLARAKSVPIKTYSNEVVTLWYRPPDVLLGSVDYSGDIDMWGVGCIFGEMISGRPMFPGATNADQLELIFKTLGSPSESTWPGVMALPEAKSNELGAYSPQPVNAILPRLDKQGGALLKSLLKLDPHGRVSAVKAMQHPYF

>Mbr-gi|167516962|

FEKLNILGEGTYGVVYRARDSRTGHQVAVKQVKMNQERGGLPLSSLREITALQQLRHTNVLQLLHVAAGLTSIFLIMEYCEHDLAALVDNMPAPFPEPAVKCLMQQLFAGLDAMHRECLIHRDLKLSNLLLTDHGILKVADFGLTRVIEDPAHHMSPTVVTLWYRPPELVFGMKNYTRAVDIWSCGCIFAELLAHEPLFPAKTEVALLEMVIGLLGAPHESIWPAFRDLPLAHRFHMPHQPYSNLKQRFGFLSSTGLDLMQDLLMYDPEKRLSAIAASVHPYF

>Mbr-gi|167538010|

YEHLNRIDEGTYGVVFRVRDRSSGHIRAVKRLKMEKEKSGFPVTSLREINTMLKVRHENIVRVEEIVVGMDDIFIVMEFVEHDVKALLESIQKPLLQAEVKTLMLQLLSGVAHLHDNWILHRDLKTSNLLLSHRGILKIADFGLAREYGDPLKEYTTLVVTLWYRAPELLLGAKSYSTAIDMWSVGCIFSELLTRKPLFDGNGEIDTMNKMFKMLGVPDETRWPGLNDLPVPRSMSLRGPSENRLPRSYGILPSSGVNLLSDLLCYDPAQPYLEQQ--KQSRF

>Mbr-gi|167516186|

FDLVNEVGQGTYGQVSKARIKNTDQLVALKMLKMEADRDGFPVTALREIKILRQLRHESIINLIGIVADPAAFYLVFEYMEHDLYGLLSSKQCTLDQEQIRYLMFQLMDGLRYCHAKHFIHRDIKGANLLVDNQCRLKIADFGLARLYEDRSRAYTNNVITLWYRPPELLYGAEVYGPEVDVWSAGCILGEFFLCRPMFRAGTEIEQLHAISMACGTPDPTNWPEAQNLPAFKTLRPRKRYERNLAGFFRALYVRRLGLLSPLMGYSPTSR------------

>Mbr-gi|167525607|

FSPVGTGGEGTYGEVKRCRHVATQQIVALKKIKTEQEANGFPQTALREIQILKQLRHENIVQLQSVAVVGKFCYMVLEFVDHDLNRILTAIKSPVSTTIFKGLALQMLRGLDHLEKNCVLHRDLKPANILLSAQGVIKLADFGLAKYHHPDRKGHTALVCSQWYRPPELLAGQVSYPEKIDSWSLAVVMGELFNLDPLFDGRTSTYQFIQIQKLCGTDSFAWLEDCPLLPAKGH--KKRQVSQRFNTLIARSSEAVVAFMDKALTVDPNHRLLAGELLKWEHK

>Mbr-gi|167525918|

FANAGCVGQGTYGIVHKVYRLEGRKPYALKGMVTVSRTEGFSTANLRELALLRELHHDNVISLREAFIHTKEVWLLFDYAEYDLWWLCNDHNLRLQEPMLKSIMFQLLQGVHYLHEQWVLHRDLKPANVFLTRGGSIKIGDLGMARVFVAPPKAFDPVVVTYWYRAPEIMLGAKHYTKAIDIWSIGCIFAELINLVPIFHVKNDAAQLEAIFKVLGMPDEQKWSLLKHMPRYPDLKFKTK-----DDVLEPESSTRLSLMRRMFHYDPEKRISADEALTAPWF

>Sro-gi|326436992|

YEKIEKIGEGTYGTVYKAKLITSGELVALKKIKLETEEEGVPSTAIREISLLKELNHRNVVRLIEVIHSEHDLHLVFEFLDCDLKKHMEVS-RQLAPDLVRSYLFQLLKGIEFCHTHRILHRDLKPQNLLIDSDGNIKIADFGLARAFGIPVRAYTHEVVTLWYRAPEILLGARQYACPVDIWSIGCIFAEMVTTRPLFPGDSEIDELFRIFRYLGTPNEHVWPGVSELPDFKTT-FPQWKRQDLAKLVPGLDPTGLDLLEQMLRYAPSARISATRALRHPYF

>Sro-gi|326436991|

FEKTEKLGEGTYGSVYKAIDKTTMAVVALKKIKLNDQEFGVPASALREIALLRELDHPNIVQLLDVIPSSSELHLILEYVYEDLRKFMHRVK-VLERPMYQSFLRQLLLGLEYCHIHRILHRDLKPENLLINHTGALKLADFGLARAFGIPVRAYTHEVVTLWYRAPEILLGSKQYACPVDMWAVGCIFAEMASSKPLFPGDSEVDQIMRIFRYLGTPTEKTWPGVSNLPDFRAN-FPRFPAIDLAPIVPQMDPVSMALLQHMLVYLPASRIPANQALKHPFF

>Sro-gi|326426444|

YVRICKVGEGTYGQVYKAQDRQNSQIVAIKAITLNGGAEGVPSNAVREISLLKRLDHPNIVRLLDVLYSQTRLMMVFEFCDQDLKQFLSHKPIQHDPNLAQTIMFQLLRAVAYIHSQHILHRDLKPQNILLDRKGRLKLADFGLARPIGVPVSKYTADVVTLWYRAPDVLLGSEHYGPCIDLWSTGVIFAEVLTGQPPFQGNSINEQLLLIFDMLGTPPP-SWD-MRQYERYKDFSMRHATSVGLETFLSRAAPVAVDLVKALLRYNPDARITAEAAMEHEFF

>Sro-gi|326430756|

YRIERRVGEGAHGVVFLGVHLASGQRVALKKVTLARLDDGIPTQVIREIRALCQLTHKNVVTLHDVFPSGMGIMLCFEYMASDLARVLQGQNLPLSAPHVKRYMSMLLSGVDFCHSHAIVHRDLKPANLLISATGQLKIADFGLARVYDE-ARPMSHQVATRWYRAPELLYGARVYDFGVDIWAVGCIFGELLNNSPLFPGENDIDQLSCVIQALGTPTRQDWPELDSLPDFAKINFDPTDPQPMHEILPDATQDAINLCSQFLVYSSSRRLPAAKALVHPYF

>Sro-gi|326431660|

FERLGRLGEGTYGIVYKAKDIETGAIVAVKRIKMKDEREGMPQTSLREVTTLKAMEHENVVQLLDIAVGHDQVYLIFEYCEHDLAWLVDNLPAPFPETVAKSLTVQLLKGLRALHSMFIVHRDIKLSNLLLNSRGYLKIADFGLARRSGDPPRPKTTNVVTLWYRAPELLFGDKAYTSKVDCWSAGCVMGELLAHKPILPGKSEVSQLDLIIQLLGTPNEAIWPGFSSLPLASRFQLTAQPYSNLKDEFRFISDRGIDLLQRLLTYDPHQRWSCDRALGHAYF

>Sro-gi|326434528|

YKKIAVLGEGQFGTVFLAEHTDTKERFAVKKIKVGSAEEGLHRTAFREIKFLQELRHANIIQLRDIFAKGFNIHLVLELCKCDMRAIILEN-IQLTPSDIKSLMLQCLQGLEYLHSHWIIHRDLKPENIFITRKGIVKLADFGLASTFGSPSRAYTAQVVTIYYRAPELLFNSKAYGAGVDVWAMGCVHGELELRRPLLPGTSEIDQLSRIFALRGSVNEHNWPGVTKLPGFLE—FDQQNPTPLRHVMPAASDLALSLMDGLLTCDPAKRLTIKQALKHAYF

1. **Multiple alignments for phylogenetic analysis of CDK family proteins in H. sapiens, *T. adhaerens*, *C. intestinalis*, *B. floridae*, *S. purpuratus* and *D. melanogaster*.**

>Hsa-CDK6

YECVAEIGEGAYGKVFKARDLKGGRFVALKRVRVQTGEEGMPLSTIREVAVLRHLEHPNVVRLFDVCTVETKLTLVFEHVDQDLTTYLDKVPEGVPTETIKDMMFQLLRGLDFLHSHRVVHRDLKPQNILVTSSGQIKLADFGLARIYSFQM-ALTSVVVTLWYRAPEVLLQS-SYATPVDLWSVGCIFAEMFRRKPLFRGSSDVDQLGKILDVIGLPGEEDWPRDVALPRQA---FHSKSAQPIEKFVTDIDELGKDLLLKCLTFNPAKRISAYSALSHPYF

>Hsa-CDK4

YEPVAEIGVGAYGTVYKARDPHSGHFVALKSVRVPNGGGGLPISTVREVALLRRLEHPNVVRLMDVCATEIKVTLVFEHVDQDLRTYLDKAPPGLPAETIKDLMRQFLRGLDFLHANCIVHRDLKPENILVTSGGTVKLADFGLARIYSYQM-ALTPVVVTLWYRAPEVLLQS-TYATPVDMWSVGCIFAEMFRRKPLFCGNSEADQLGKIFDLIGLPPEDDWPRDVSLPRGA---FPPRGPRPVQSVVPEMEESGAQLLLEMLTFNPHKRISAFRALQHSYL

>Hsa-CDK2

FQKVEKIGEGTYGVVYKARNKLTGEVVALKKIRLDTETEGVPSTAIREISLLKELNHPNIVKLLDVIHTENKLYLVFEFLHQDLKKFMDASALGIPLPLIKSYLFQLLQGLAFCHSHRVLHRDLKPQNLLINTEGAIKLADFGLARAFGVPVRTYTHEVVTLWYRAPEILLGCKYYSTAVDIWSLGCIFAEMVTRRALFPGDSEIDQLFRIFRTLGTPDEVVWPGVTSMPDYKP-SFPKWARQDFSKVVPPLDEDGRSLLSQMLHYDPNKRISAKAALAHPFF

>Hsa-CDK3

FQKVEKIGEGTYGVVYKAKNRETGQLVALKKIRLDLEMEGVPSTAIREISLLKELKHPNIVRLLDVVHNERKLYLVFEFLSQDLKKYMDSTPGELPLHLIKSYLFQLLQGVSFCHSHRVIHRDLKPQNLLINELGAIKLADFGLARAFGVPLRTYTHEVVTLWYRAPEILLGSKFYTTAVDIWSIGCIFAEMVTRKALFPGDSEIDQLFRIFRMLGTPSEDTWPGVTQLPDYKG-SFPKWTRKGLEEIVPNLEPEGRDLLMQLLQYDPSQRITAKTALAHPYF

>Hsa-CDK1

YTKIEKIGEGTYGVVYKGRHKTTGQVVAMKKIRLESEEEGVPSTAIREISLLKELRHPNIVSLQDVLMQDSRLYLIFEFLSMDLKKYLDSIPPYMDSSLVKSYLYQILQGIVFCHSRRVLHRDLKPQNLLIDDKGTIKLADFGLARAFGIPIRVYTHEVVTLWYRSPEVLLGSARYSTPVDIWSIGTIFAELATKKPLFHGDSEIDQLFRIFRALGTPNNEVWPEVESLQDYKN-TFPKWKPGSLASHVKNLDENGLDLLSKMLIYDPAKRISGKMALNHPYF

>Hsa-CDK5

YEKLEKIGEGTYGTVFKAKNRETHEIVALKRVRLDDDDEGVPSSALREICLLKELKHKNIVRLHDVLHSDKKLTLVFEFCDQDLKKYFDSCNGDLDPEIVKSFLFQLLKGLGFCHSRNVLHRDLKPQNLLINRNGELKLADFGLARAFGIPVRCYSAEVVTLWYRPPDVLFGAKLYSTSIDMWSAGCIFAELANGRPLFPGNDVDDQLKRIFRLLGTPTEEQWPSMTKLPDYKP--YPMYPATSLVNVVPKLNATGRDLLQNLLKCNPVQRISAEEALQHPYF

>Hsa-CDK16

YIKLDKLGEGTYATVYKGKSKLTDNLVALKEIRLEH-EEGAPCTAIREVSLLKDLKHANIVTLHDIIHTEKSLTLVFEYLDKDLKQYLDDCGNIINMHNVKLFLFQLLRGLAYCHRQKVLHRDLKPQNLLINERGELKLADFGLARAKSIPTKTYSNEVVTLWYRPPDILLGSTDYSTQIDMWGVGCIFYEMATGRPLFPGSTVEEQLHFIFRILGTPTEETWPGILSNEEFKTYNYPKYRAEALLSHAPRLDSDGADLLTKLLQFEGRNRISAEDAMKHPFF

>Hsa-CDK17

YIKLEKLGEGTYATVYKGRSKLTENLVALKEIRLEH-EEGAPCTAIREVSLLKDLKHANIVTLHDIVHTDKSLTLVFEYLDKDLKQYMDDCGNIMSMHNVKLFLYQILRGLAYCHRRKVLHRDLKPQNLLINEKGELKLADFGLARAKSVPTKTYSNEVVTLWYRPPDVLLGSSEYSTQIDMWGVGCIFFEMASGRPLFPGSTVEDELHLIFRLLGTPSQETWPGISSNEEFKNYNFPKYKPQPLINHAPRLDSEGIELITKFLQYESKKRVSAEEAMKHVYF

>Hsa-CDK18

YVKLDKLGEGTYATVFKGRSKLTENLVALKEIRLEH-EEGAPCTAIREVSLLKNLKHANIVTLHDLIHTDRSLTLVFEYLDSDLKQYLDHCGNLMSMHNVKIFMFQLLRGLAYCHHRKILHRDLKPQNLLINERGELKLADFGLARAKSVPTKTYSNEVVTLWYRPPDVLLGSTEYSTPIDMWGVGCIHYEMATGRPLFPGSTVKEELHLIFRLLGTPTEETWPGVTAFSEFRTYSFPCYLPQPLINHAPRLDTDGIHLLSSLLLYESKSRMSAEAALSHSYF

>Hsa-CDK14

YEKLEKLGEGSYATVYKGKSKVNGKLVALKVIRLQ-EEEGTPFTAIREASLLKGLKHANIVLLHDIIHTKETLTLVFEYVHTDLCQYMDKHPGGLHPDNVKLFLFQLLRGLSYIHQRYILHRDLKPQNLLISDTGELKLADFGLARAKSVPSHTYSNEVVTLWYRPPDVLLGSTEYSTCLDMWGVGCIFVEMIQGVAAFPGMKDQDQLERIFLVLGTPNEDTWPGVHSLPHFKPERFTLYSSKNLRQAWNKLVNHAEDLASKLLQCSPKNRLSAQAALSHEYF

>Hsa-CDK15

YLNLEKLGEGSYATVYKGISRINGQLVALKVISMN-AEEGVPFTAIREASLLKGLKHANIVLLHDIIHTKETLTFVFEYMHTDLAQYMSQHPGGLHPHNVRLFMFQLLRGLAYIHHQHVLHRDLKPQNLLISHLGELKLADFGLARAKSIPSQTYSSEVVTLWYRPPDALLGATEYSSELDIWGAGCIFIEMFQGQPLFPGVSNLEQLEKIWEVLGVPTEDTWPGVSKLPNYNPEWFPLPTPRSLHVVWNRLVPEAEDLASQMLKGFPRDRVSAQEALVHDYF

>Hsa-CDK11B

FQCLNRIEEGTYGVVYRAKDKKTDEIVALKRLKMEKEKEGFPITSLREINTILKAQHPNIVTVREIVVGMDKIYIVMNYVEHDLKSLMETMKQPFLPGEVKTLMIQLLRGVKHLHDNWILHRDLKTSNLLLSHAGILKVGDFGLAREYGSPLKAYTPVVVTLWYRAPELLLGAKEYSTAVDMWSVGCIFGELLTQKPLFPGKSEIDQINKVFKDLGTPSEKIWPGYSELPAVKKMTFSEHPYNNLRKRFGLLSDQGFDLMNKFLTYFPGRRISAEDGLKHEYF

>Hsa-CDK10

FEKLNRIGEGTYGIVYRARDTQTDEIVALKKVRMDKEKDGIPISSLREITLLLRLRHPNIVELKEVVVGLESIFLVMGYCEQDLASLLENMPTPFSEAQVKCIVLQVLRGLQYLHRNFIIHRDLKVSNLLMTDKGCVKTADFGLARAYGVPVKPMTPKVVTLWYRAPELLLGTTTQTTSIDMWAVGCILAELLAHRPLLPGTSEIHQIDLIVQLLGTPSENIWPGFSKLPLVGQYSLRKQPYNNLKHKFPWLSEAGLRLLHFLFMYDPKKRATAGDCLESSYF

>Hsa-CDK9

YEKLAKIGQGTFGEVFKARHRKTGQKVALKKVLMENEKEGFPITALREIKILQLLKHENVVNLIEICRTKGSIYLVFDFCEHDLAGLLSNVLVKFTLSEIKRVMQMLLNGLYYIHRNKILHRDMKAANVLITRDGVLKLADFGLARAFSLAPNRYTNRVVTLWYRPPELLLGERDYGPPIDLWGAGCIMAEMWTRSPIMQGNTEQHQLALISQLCGSITPEVWPNVDNYELYEKLELVKGQKRKVKDRLKYRDPYALDLIDKLLVLDPAQRIDSDDALNHDFF

>Hsa-CDK13

FDIIGIIGEGTYGQVYKARDKDTGEMVALKKVRLDNEKEGFPITAIREIKILRQLTHQSIINMKEIVTDKGAFYLVFEYMDHDLMGLLESGLVHFNENHIKSFMRQLMEGLDYCHKKNFLHRDIKCSNILLNNRGQIKLADFGLARLYSSESRPYTNKVITLWYRPPELLLGEERYTPAIDVWSCGCILGELFTKKPIFQANQELAQLELISRICGSPCPAVWPDVIKLPYFNTMKPKKQYRRKLREEFVFIPAAALDLFDYMLALDPSKRCTAEQALQCEFL

>Hsa-CDK12

FDIIGIIGEGTYGQVYKAKDKDTGELVALKKVRLDNEKEGFPITAIREIKILRQLIHRSVVNMKEIVTDKGAFYLVFEYMDHDLMGLLESGLVHFSEDHIKSFMKQLMEGLEYCHKKNFLHRDIKCSNILLNNSGQIKLADFGLARLYNSESRPYTNKVITLWYRPPELLLGEERYTPAIDVWSCGCILGELFTKKPIFQANLELAQLELISRLCGSPCPAVWPDVIKLPYFNTMKPKKQYRRRLREEFSFIPSAALDLLDHMLTLDPSKRCTAEQTLQSDFL

>Hsa-CDK7

YEKLDFLGEGQFATVYKARDKNTNQIVAIKKIKLGHAKDGINRTALREIKLLQELSHPNIIGLLDAFGHKSNISLVFDFMETDLEVIIKDNSLVLTPSHIKAYMLMTLQGLEYLHQHWILHRDLKPNNLLLDENGVLKLADFGLAKSFGSPNRAYTHQVVTRWYRAPELLFGARMYGVGVDMWAVGCILAELLLRVPFLPGDSDLDQLTRIFETLGTPTEEQWPDMCSLPDYVT--FKSFPGIPLHHIFSAAGDDLLDLIQGLFLFNPCARITATQALKMKYF

>Hsa-CDK19

YE-GCKVGRGTYGHVYKARRKDGEKEYALKQI----EGTGISMSACREIALLRELKHPNVIALQKVFLSDRKVWLLFDYAEHDLWHIIKFHPMQLPRSMVKSLLYQILDGIHYLHANWVLHRDLKPANILVMERGRVKIADMGFARLFNSPLKPLDPVVVTFWYRAPELLLGARHYTKAIDIWAIGCIFAELLTSEPIFHCRQEHDQLDRIFSVMGFPADKDWEDIRKMPEYPTLQFRRYANSSLIKKHVKPDSKVFLLLQKLLTMDPTKRITSEQALQDPYF

>Hsa-CDK8

YE-GCKVGRGTYGHVYKAKRKDGDKDYALKQI----EGTGISMSACREIALLRELKHPNVISLQKVFLSDRKVWLLFDYAEHDLWHIIKFHPVQLPRGMVKSLLYQILDGIHYLHANWVLHRDLKPANILVMERGRVKIADMGFARLFNSPLKPLDPVVVTFWYRAPELLLGARHYTKAIDIWAIGCIFAELLTSEPIFHCRQEHDQLDRIFNVMGFPADKDWEDIKKMPEHSTLMFRRYTNCSLIKKHVKPDSKAFHLLQKLLTMDPIKRITSEQAMQDPYF

>Hsa-CDK20

YCILGRIGEGAHGIVFKAKHVETGEIVALKKVALRRLEDGFPNQALREIKALQEMENQYVVQLKAVFPHGGGFVLAFEFMLSDLAEVVRHAQRPLAQAQVKSYLQMLLKGVAFCHANNIVHRDLKPANLLISASGQLKIADFGLARVFSPDSRLYTHQVATRWYRAPELLYGARQYDQGVDLWSVGCIMGELLNGSPLFPGKNDIEQLCYVLRILGTPNPQVWPELTELPDYNKISFKEQVPMPLEEVLPDVSPQALDLLGQFLLYPPHQRIAASKALLHQYF

>Hsa-CDKL1

YEKIGKIGEGSYGVVFKCRNRDTGQIVAIKKFLESEDDPVIKKIALREIRMLKQLKHPNLVNLLEVFRRKRRLHLVFEYCDHTVLHELDRYQRGVPEHLVKSITWQTLQAVNFCHKHNCIHRDVKPENILITKHSVIKLCDFGFARLLTGPSDYYTDYVATRWYRSPELLVGDTQYGPPVDVWAIGCVFAELLSGVPLWPGKSDVDQLYLIRKTLGDLIPRHQQVFSTNQYFSGVKIPDEDMEPLELKFPNISYPALGLLKGCLHMDPTQRLTCEQLLHHPYF

>Hsa-GSK3alpha

YTDIKVIGNGSFGVVYQARLAETRELVAIKKVLQDKR------FKNRELQIMRKLDHCNIVRLRYFFYSELYLNLVLEYVPETVYRVARKAKLTIPILYVKVYMYQLFRSLAYIHSQGVCHRDIKPQNLLVDDTAVLKLCDFGSAKQLVRG-EPNVSYICSRYYRAPELIFGATDYTSSIDVWSAGCVLAELLLGQPIFPGDSGVDQLVEIIKVLGTPTREQIREMN--PNYTEFKFPQIKAHPWTKVFKRTPPEAIALCSSLLEYTPSSRLSPLEACAHSFF

>Hsa-MAK

YTTMRQLGDGTYGSVLMGKSNESGELVAIKRMKRKF-YSWDECMNLREVKSLKKLNHANVIKLKEVIRENDHLYFIFEYMKENLYQLMKDRNKLFPESVIRNIMYQILQGLAFIHKHGFFHRDMKPENLLCMGPELVKIADFGLARELRSQ-PPYTDYVSTRWYRAPEVLLRSSVYSSPIDVWAVGSIMAELYMLRPLFPGTSEVDEIFKICQVLGTPKKSDWPEGYQLASSMNFRFPQCVPINLKTLIPNASNEAIQLMTEMLNWDPKKRPTASQALKHPYF

>Tad-gi|196001971|

YKKIKLLGEGQFAVVYQAEDTHDNKIVAVKKIKLGPANDGINRTALREIKLLQELKHENIIGLLDVFGHKSNISLVFDYMEADLEVIIKDSSIILTNAHIKQYIIMTLRGLEYIHSNWILHRDMKPNNLLIDSNGILKLADFGLARYFGSPNRYYTNQVVTVWYRAPELLFGANSYGTGVDMWAVGCILAELLLRVPFLPGDSDLDQLTRIFQTLGTPIEDKWADIHKLPGYIK--FKTLPAIPLNEIFTAASDDMLDLLRRLFEYNPSSRITATEALQMKYF

>Tad-gi|195996663|

FTILGRVGEGAHGVVFKAKHVGNGEVVALKKIHLKKIDDGIPNNILREIKTLQAITHENIVRLFDVFPDGSSLVLAFEYMVTDLSEILRSSQNTLPE-VSSYYMIMLLRGISFCHENHIIHRDLKPANLLISSSGQLKLADFGLARVMSVEERLYSHQVATRWYRAPELLYGSRTYDEGVDLWYLGCIFAELINKSPLFPGDSDIKQLGCVLSILGTPTTSTWPGLIELPDYNKITFSGFLPIPFESIVPDATSEVGKI------------------------

>Tad-gi|196013334|

FEWLNRIEEGTYGVVYRAKDLKSDEVVALKRLKMEKEREGFPITSLREINTLLKADHPNIVHVREIVVGMDKIYIVMEYVEHDLKTLMESMSQPFSISEVKCLMKQLLSAVQHLHDNWILHRDLKTSNLLLSHQGILKVGDFGLAREYGSPLKVYTSIVVTLWYRCPELLLGVKEYSTAVDMWSVGCIFGEFLVKKPLFPGKSEIDQLNKIFKDLGTPNDQIWSGFSELPVAKKVTFTEQPYNRLRDRFGYLTDQGFDLLNRFLTYDPKKRISAEDALNHEYF

>Tad-gi|196010283|

FDKLNRIGEGSYGVVYRARDLDSKEIVAIKKIRMENERDGIPVSSLREITLLVNLKHINIVNLKDVVVGLDSIFLVMEYCEQDLSSLLYNMKAPFTEPQVKCLSLQLIHGVQYLHHNFVIHRDLKVSNLLLTDKGILKVADFGLARNYGLPAAPMTPTIVSLWYRAPEVLLGCTKHTLAVDMWSVGCIIAELFDHNVFLKGKSEKDQLDLMCQMLGTPNEAIWEDIRDMPLYGKIILRQQPYNNLKHKFSWLSAAGLNLLNSLLTYDPGRRITADETLKLSYF

>Tad-gi|196002669|

--ILKQIGEGTYGQVFKAKIKDTDKLVALKKVRTDHEREGFPITAVREIKILKQLNHQSIVNLLGIVSNRCAFYLVFEYMDHDLMGLLESGLVTFDEEHIRSFMRQIMEGLNYCHKRQLLHRDIKCSNLLMNNKGQIKIADFGLARFYNPDSRPYTNKVITLWYRPPELLLGEERYGPSVDVWSCGCILGEFFTKKPIFQANSEINQLDLISQICGTPCPENWPSVVELPYYNNFKLRKY-ERRLEQEFHDLPELAVDLMQYMLILDPSMRYNAEQSLQHPFI

>Tad-gi|195998898|

YEKITKIGHGTFGEVFKARNRISKEFVALKRVLLGNEKEGFPITSLREIKILRALKHDNIVRLQEICRSRGSIYLVFEFCAHDLAGLLQNPQVKFNLSEIKRMMKHLLSGLFYIHSNKVLHRDLKAANVLVTRDGVLKLADFGLARVYSRKTHCFTNRVVTLWYRAPELLLGCRDYGPAIDMWAIGCIMAEFWTRSAIMQGNSETNQLTLITQLCGSITPEVYPDVDKLDLFKKFDLPASQKRRVKERLSYRDRHALDLIDRCLTIDPAKRIDSDSALNHDFF

>Tad-gi|196013348|

FHKLEKIGEGTYGKVYKACNKITGQTVALKKIRLDSDKEGVPSTTLREISILRSLNHSFVVRLYDVVHSDQCLYLVFEYLDHDLKHYLDHA-YKIPPALLKSYLYQMLRAISYCHSRRVLHRDLKPQNLLIDSTGTLKLADFGLARIFGLPVRQYTHEVITLWYRAPEILLGSTYYSTPVDIWSIGCIFVEMINRRPLFAGDSEIDQLFRVFRTLGTPDEITWPGVSEMSDYKS-TFPKWPSRDLNSVIYSHDEDCVDLIKQMLVYEPNGRISARLALQHPYF

>Tad-gi|196003954|

YLKIEKIGEGTYGVVYKGKNRNTQQLVALKKIRLENEEEGIPSTAIREVSLLKELKHPNIVDLIEVLYEESKLYLVFEFLDMDLKRYLDTLPKTIDAMLMKSYLYQILLGVVYCHSHRVLHRDLKPQNLLINSKGCIKLADFGLGRAFGVPVRVYTHEVVTLWYRAPEVLLGSTRYSCPLDIWSTGTIFAEMWLRRPLFQGDSEIDELFRIFRILGTPDDDIWPGVSSLPEFKS-SFPKWSKQSYDTFVPNMSESGIDLLSKMLIYDPANRISGKRALSHPYF

>Tad-gi|196001193|

YEKLEPLGEGSYATVYKGYSIGHQKLVALKEITLN-EEEGTPFTAIREASLLKQLKHANIVVLHDIIQTPTKLTFVFEYVTTDLSQYLNLHPGGLNMKNVKLFLYQLLRGLSYCHQRRILHRDIKPQNILVSEIGEIKLADFGLARAKSVPSKTYSPDVVTLWYRPPDVLLGSTNYSTSLDIWGVGCIFTEMVSGIATFPGVRNSDQLDKIWHKLGTPTEETWPGVTSYPEYCAAETMFYESRTIAEVIPRLDPGAADLATKMLQYQPFKRIFCQAAMNHQYF

>Tad-gi|195996637|

YDKLHKLGEGTYATVFKGRSKLTNDFVALKEIRLEH-EEGAPCTAIREVSLLKDLKHANIVTLHDTIHTERSLVLVFEYLDRDLKQYMDSCGSILDMSNVKIFLFQLLRGLAYCHSRRVLHRDLKPQNLLINERGELKLADFGLARAKSVPSKTYSSEVVTLWYRPPDVLLGSTEYSTSIDMWGVGCIFYEMATGLPMFPGSSTENQLQTIWEILGTPTEEEWSGLTRNLKVNSLSFHDCKGEPLRNRAPRLEADGLDLLAKFLQYKAKSRISSADAMKHKYF

>Tad-gi|196000717|

YEKLVKIGEGTYGTVYKAVNHDTGEIVALKKVRIDDENEGIPSFALREICLLKELKHKNIVMLYDVIHGNKELMIVFEYCDQDLKRYCDACQGKIDPSIVQSFTNQLLQGLAYCHSHHILHRDITPQNILVTGNGDIKLADFGLARNFGIPVKSFSAEVVTLWYRSPDVLLGATLYDTSIDIWSTGCIFAELSNGQPLLPGKDVADQLKIIFKIFGTPNEQIWPGVSQLMKDKD--YPSYNAMSILHVVPNLNQLGCDLFQLMMVLDPSKRCTAEQALQHAYF

>Tad-gi|195999760|

YVQVTEIANGAYGRVYKARDLNHNRLVALKKIAVINDEQGIPISTIREITSLKSLGHQNIVRLYDIFANTMDLTLVFEHVEQDLQTYIRNCPSGIDTRKIKDIIYQIVNAIDFLHANRIVHRDLKPQNVLISRQGIVKVADFGLAKVFCENV-PITSVVVTLWYRCPEVLLQS-TYATPVDMWSVGCIMAELYLREPLFCGQTDIDQLQKIFSMTGLPDESEWPVNIPFSRST---FCQYTRRQYSEMMPEICQDGVDLLQKLLKFNPKERLTAEESLQHPYF

>Tad-gi|196015356|

FE-GCKVGRGTYGHVYKAKMKSSGKEYALKLI----EGSGISMSACREIALLREVHHPNVISLQGVFLTNRKVWLLFDFAEHDLWHIIKYHAISLDSKMVKSLLKQILEGIHYLHANWILHRDLKPANILVMERGRVKIADMGFARHYWSPLKPLDPVVVTSWYRAPELLLGARHYTKAIDIWAIGCIFAELLTSEPIFHCRQERDQLERIFMVMGYPHEKDWEDIKKTPNYAKLAFRKYTNCSLAKKFIKQDSKEFILLQKCLTIDPNKRISSEQAIDDAYF

>Tad-gi|196015366|

FE-GCKVGRGTYGHVYKAKMKTSGKEYALKLI----EGTGISMSACREIAILREISHTHVISLQGVFLTSRKVWLLLDFAEHDLWHIIKFHSVPIDTKIVKSLLRQILDGIQYLHSNWILHRDLKPANILVMERGRVKIADMGFARHFWAPLKPLDPVVVTFWYRAPELLLGARHYTKAIDIWAIGCIFAELLTSEPIFHCRQEHDQLDRIFTVMGFPHERDWEDIKIMPEYKRLQFKKFASCSLAKKFIRHDSREFSLLQKFLIADPNKRISAELAMDDAYF

>Bfl-gi|260821968|

YQKIDFLGEGQFATVYKAKDTKTGQIVAVKKIKLGQAKDGINRTALREIKLLQELSHTNIIGLYDVFGHKSNISLVFDFMDTDMEVVIKDTSIVMTGPHIKAYAIMTLEGLEYLHNNWILHRDLKPNNLLVNSQGILKITDFGLAKTFGSPNRVYTHQVVTRWYRAPELLYGARIYGTGVDMWAVGCILAELLLRV-----RTDIDIRCRFL-----------------------------------------------------------------------

>Bfl-gi|260799041|

YTIMERIGEGAHGIVFKAKHVESGEVVALKKVPLRRLEDGIPNTALREIKALQEIENQHVVKLREVFPHGTGFVLVFEYMLSDLSEVLRNSNRPLTEAQVKSYMMMLLKGVAFCHENNIMHRDLKPANLLISETGHLKIADFGLARVFANEGRLYSHQVATRWYRAPELLYGARKYEEGVDLWAVGCIFGELLNNSPLFPGENDIEQLCCVLRVLGTPNEKIWPGMSELPDYNKITFPENPPIPLEVVVPDASPQAIDLLKRFLVYPSSQRVSAKEALLHPYF

>Bfl-gi|260838232|

YVKIEKIGEGTYGVVYKGRNKKTGLTVALKKIRLESEEEGVPSTAIREISLLKELVHPNIVNLQDVLMQESKLYLVFEFLTMDLKKYMDSIPSYMDSMLVKSYLYQILQGITFCHSRRVLHRDLKPQNLLIDNKGIIKLADFGLARAFGIPVRVYTHEVVTLWYRAPEVLLGGARYSTPIDIWSIGTIFAEMATKRPLFHGDSEIDQLFRIFRTMGTPTEDIWPGVTQMPDYKP-SFPSWKPNQLKTSVKNMDDQALDLLQKTLIYDPANRISAKAALIHPYF

>Bfl-gi|260818988|

FEKLNRLGEGTYGIVYRARDTRSGEIVALKKMRMDREKDGLPISGLREITLLLNVTHRNIVDLKEVVVGLESIFLVMEYCEQDLASLLDNMDTPFSEAQVKCIMLQVFNGLEYLHDNFIIHRDLKVSNLLMTDKGCIKIADFGLARTFGLPPKPMTPRVVTLWYRSPELLLGAKTQTTAVDMWAAGCIFGELLAHKPLLPGRSEIHQLELIVELLGTPSEAIWPGFSQLPALEQISLKKQPYNNLKHRFPWLSEAGLRLLNFLFMYDPKKRGSAEECMKSSYF

>Bfl-gi|260787719|

YEKLEKIGEGTYGTVFKAKNKESHEIVALKRVPLDDDHEGVPSSALREICLLKELKHKNIVRLHDVLHSEKKLTLVFEYCDQDLKKYFDSCSGDIDPETVKSFMYQLLRGLAFCHSHNVLHRDLKPQNLLINKNGELKLADFGLARAFGIPVRCYSAEVVTLWYRPPDVLFGAKLYSTSIDMWSAGCIFAELANGRPLFPGNDVDDQLKRIFRYFKNLHEDTWPGMSRLPDYKP--FPIYQVTSLAVVVPKLCPKGRDLLQRLLVCNPAHRLSADEGLNHPYF

>Bfl-gi|260789724|

FSCLNRIEEGTYGVVYRAKDKKTGEIVALKRLKMEKEKEGFPITSLREINTLLKAQHPNIVTVREIVVGMDKIYIVMDYVEHDLKSLMETMKQPFLVGETKTLLIQLLRAVQHLHDNWILHRDLKTSNLLLSHKGILKVGDFGLAREYGSPLKPYTPIVVTLWYRAPELLLGVKEYSTHIDLWSVGCIFAEFLTMKPLWSGKSEIDQLNKIFKDLGTPSERIWPGYNELPAVKKCTFAEYPYNNLRSRFGYLSDLGFELLNKFLTYCPSKRITAEDALKHEFF

>Bfl-gi|260797491|

YEKLAKIGQGTFGEVFKARHRKTKQFVALKKVLMENEKEGFPITALREIKILQMVKHENVVQLLEICRTKGSIYLVFDFCEHDLAGLLSNANVKFTLSEIKKVMQQLLNGLYYIHRNKILHRDMKAANILINKHGVLKLADFGLARAFSVTANRYTNRVVTLWYRPPELLLGERNYGPPIDLWGAGCIMAEMWTRSPIMQGNTEQHQLTLISQLCGSISAEVWPSVEKLDLFSKLELPKGQKRKVKERLRYKDPYALDLIDRLLTLDPTKRIDSDDALNHDFF

>Bfl-gi|260807641|

YAFLNLINEGTYGVVYKAMHKTTGDVVAIKMLKSENQPHGVSGTGLREVNIMSKARHINVISLREVVYGIDKAYLVMEYAETDLKQLMYNLQRPFSVSETKGLLVQLLYAVQYLHDKDILHRDIKTENLLLNLHGILKVTDFGLARTFSKGDKHLSPVVVTLWYRAPELLLGSKTYSTPVDLWSVGCVFAELLTGNPFWDGESEIDQLHQIFCDLGTPSEKIWPGYSRLPFLKTCILPDFPYNRLRRRLGTLTELGLHLLNWFLTYSPARRVTAVQALQHCEK

>Bfl-gi|260809423|

FEILSQVGEGTYGQVYKAKDKQTKEVVALKKVRLDNEKEGFPITAVREIKILRQLCHRSIVNLKEIVTDKGAFYLVFEYVDHDLMGLLESGLVQFNEDQIKSMMKQLMQGLDYCHKKNFLHRDIKCSNILINNRWQVKLADFGLARLYHAEARPYTNKVITLWYRPPELLLGEEQYGPAIDIWSCGCILGELFTRKPIFQANQEPAQLELISRICGAPCPAVWPDVIKLPYFHTIKPKKQYRRRLREEFAYFPTPALDLMDHMLTLDPSKRCTADQALESSWL

>Bfl-gi|260829231|

----------------------------------------VPLCVHCEPFL-----LSSISRLLDICHGETKLTLVFEHVDQDLHTYLEKCPAGLGPDRIKDIMHQLLSGVEFLHMHRVVHRDLKPQNILVTSTGQVKLADFGLARHYSFQM-ALTSVVVTLWYRSPEVLLQA-SYATPVDIWSVGCIFAELHTRKPIFQGNSDIDQLNKIFDVIGTPIKEEWPEEVSLPWSS---FQPRPGIPLESLLPEVEPLAKDMLEKMLCFNPHRRITAKDALQHAYI

>Bfl-gi|260783497|

YE-GCKVGRGTYGHVYKAKRKDGDKEYALKQI----EGTGISMSACREIALLRELKHPNVITLHRVFLSDRKVWLLFDFAEHDLWHIIKWHPVPIPRQMVKSLLYQILDGIHYLHANWILHRDLKPANILVMERGRVKIADMGFARLFNSPLKPLDPVVVTFWYRAPELLLAARHYTKAIDIWAIGCIFAELLTSEPIFHCRQEHDQLDRIFNVMGFPQEKDWEDIRKMPEHATLMFRR--------------------------------------------

>Bfl-gi|260783491|

YE-GCKVGRGTYGHVYKAKRKDGDKEYALKQI----EGTGISMSACREIALLRELKHPNVITLHRVFLSDRKVWLLFDFAEHDLWHIIKWHPVPIPRQMVKSLLYQILDGIHYLHANWILHRDLKPANILVMERGRVKAADMGFARLFNSPLKPLDPVVVTFWYRAPELLLAARHYTKAIDIWAIGCIFAELLTSEPIFHCRQEHDQLDRIFNVMGFPQEKDWEDIRKMPEHATLMFRRYQNCCLLKKHVKADSKAFHLLSKLLTMDPTKRISSDQAMADPYF

>Cin-gi|198412574|

YEKIEFLGEGQFATVYKAKDTKDDRIVAVKKIKLGNAKDGINRTALREIKLLQELHHENIIGLLDVFGQKSNISLVFDFMETDLEVIIKDMSIVLTQAHIKSYMIMTLHGLEYLHSLWILHRDLKPNNLLFDSHGVLKIGDFGLAKTFGSPSREYTHQVVTRWYRSPELLFGSRLYGVGVDMWAVGCILAELLLRVPFLPGDSDLDQLSKIFETLGTPSDAEWPGMKDLPDYIC--FKEFPGIPLSQCFSAARDDLLELISGLLRYNPGLRVTAVQALHFSFF

>Cin-gi|198420048|

YHILGRIGEGAHGIVFKAKHIERGEVVALKKVPLRKIEDGIPNQALREIKALQEIGAQNVVKLHDVFPHGTGFVLVFEYMLSDLSEVIRNSERSLTESQIKSYMMMLLKGVAFCHQNNIMHRDLKPANLLISSTGHLKIADFGLARVFDNDERLYSHQVATRWYRSPELLYGARRYDEGVDLWAVGCIFGEMLNNSPLFPGENDIEQLCCVLRVLGTPNETIWPGMSILPDYNKITFPENPPIPLEEIVPDASEDALDLLKKFLVYPSNQRIAATHALLHPYF

>Cin-gi|198425580|

YVKIEKIGEGTYGVVYKGRNKKTNQIVALKKIRLESEEEGVPSTAIREISILKELQHPNIVSLQDVVLQESNLFLVFEFLQMDLKKYMDTIGSYMDKDLVKSYTYQILQGITYCHSRRVLHRDMKPQNLLIDRNGIIKLADFGLARAFGIPVRVYTHEVVTLWYRAPEVLLGSSRYSTPVDVWSIGTIFAEMATKRPLFHGDSEIDQLFRIFRVLGTPTDDIWPGVTQLKDYKQ-TFPKWKKGCLNDSVKNLDEDGIDLLTKCLVYNPAKRISAKVALCHPYF

>Cin-gi|198422386|

YEKIEKIGEGTYGTVFKAKNRESGEVVALKRVQLDDDDEGVPSSALREICILKELKHKNVVRLHDVLHSERKMTLVFEYCEQDLKKYFDSCGGEIDRPTVQSFMYQLLKGLAFCHQQNILHRDLKPQNLLINKNGELKLADFGLARSFGIPVRCYSAEVVTLWYRPPDVLFGAKLYSTTIDTWSAGCIFAEISNGVPLFPGNDVEDQLKRIFKVLGTPTEQSWPGVSKLPDFKI--FPLYPSAHWAAITPRLSSSGHDLLKCLIVANPSERLTASNALKHRYF

>Cin-gi|198413913|

FQCLNRIEEGTYGVVYRAKDKKTDNVVALKRLKMEKEREGFPITSLREVCTLLKAHHPNCVRVQEIVVGVDKIYIVMDYVEHDLKSLMETMKQPFLTGEVKTLMIQLLQGVHHLHDNWILHRDLKTSNLLLSHRGILKIGDFGLAREYGSPLKPYTPIVVTLWYRCPELLLGAKEYSTAVDMWSVGCIFAEFLNKKPLFPGKSETMQLNLIFKELGTPSEKIWPGYNDLPIVKKTTFVEYPYNTLRKRFGDISQKGFDLLNRFLTYSPERRISAYNALKHDWF

>Cin-gi|198420046|

FEKLNRIGEGTYGIVYRARDKVSKEIVALKKVRTENEKEGISISSIREITLLLNLKHKNIVELKEVVVGLDSIFLVMEYCEQDLANLLDNMTTPFSEAQVKCITLQLLRGLAFLHESFIIHRDLKVSNLLMTDGGVLKIADFGLARLYSIPQTSMTPRVVTLWYRAPELLFGATKYTKSIDTWAAGCILAELLAHKPIFPGKSEIEMIELLIQMLGSPSEEIWPGFSELPAIKTIYLKKQPYNNLKHRFPWVSEAGLRLLNLMLLYNPSKRISAQDCIEMSYF

>Cin-gi|198436212|

YKKLGVLGEGSYATVYKGQSKHTGQLVALKEISLN-AEEGAPFTAIREASLLKTLKHANIITLHDIVHATTTLTLVFEYMVTDLSTYMEWYGCGIHPSNAVLFTFQLLRGLDYCHQRRILHRDLKPQNLLLSDLGELKLADFGLARAKSIPTNTYSNEVVTLWYRPPDVLLGSRNYTTSLDMWGVGCIFLEMLTGMPVFPGHSDNDQLTKIFKVLGTPTPQTWRKLPSFPCYEDYSSCVRNRVTFDETFTKIIEFGVEFALDLLQFEPDNRLSGAEALHHPIF

>Cin-gi|198436214|

YAFISITGEGTFGQVYKARDKHTDEICALKKVRLDNEREGFPITAVREIKILRQLQHRNIVCLKDVLTDECAFYLVFEYMDHDLMGLLESGMVHFNENHIKSFMKQLLDGLNHCHKKGFLHRDIKCSNILLNNKGEIKLADFGLARFFNKDQRPYTNRVITLWYRPPELLLGEEMYTPSIDIWSCGCILAELFTKKPLFQADRELAQLECISRVCGSPCPAVWPDVIKLPHFHTMKPKRQHRRKLREDFSYLPTLAIDLLDQMLTLDPSKRFTAEEALNCPWL

>Cin-gi|198427109|

YERITKIGQGTFGEVFKARDRKTDRLVALKKVIMENEKEGFPITALREIKILQLLKHENVVDLIEICRTKGSIYLVFEFCAHDLAGLLSNATVKFTLGEIKKTMLQLLEGLFYIHRNKILHRDMKAANILITKNGVLKLADFGLARAFSYTANRYTNRVVTLWYRPPELLLGDRDYGPPIDLWGAGCIMTEMWTRSPIMQGHTEQQQLTLISQLCGSITKQVWPGVEKYDLFTKMELPTGQKRRVKERLKYRDQYALDLIDKLLSLDPKHRIDSDEALNHDFF

>Cin-gi|198431481|

YHTLSEIGVGAYGVVFKAQDMQENKFVAIKCVRVENSEQGMPLSTVREIALLRQLEHPNVVRLLDICAGETQLMLVFEYVDQDLDVFLKKCPDGLEPEKVTDIMRQMLGGLDFLHSMRVVHRDMKPQNILITTTGQVKIADFGLARIYSIDM-ALTEVVVTLWYRSPEVLLLD-SYATPVDIWSAGCIFAELFNRRPLFRGTGDVNQLKKIFDFIGRPSEAEWPLNVAVPIDS---FPPQPPRPPQKFVPSITEDAADFLMKLLVFDPGKRLTAQSALQHKYV

>Dme-cdk7-gi|17530793|

YAKLSFLGEGQFATVYKARDTVTNQIVAVKKIKKGSARDGINRTALREIKILQELQHENIIGLVDVFGQLSNVSLVFDFMDTDLEVIIKDNKIILTQANIKAYAIMTLKGLEYLHLNWILHRDLKPNNLLVNSDGILKIGDFGLAKSFGSPNRIYTHHVVTRWYRSPELLFGARQYGTGVDMWAVGCILAELMLRVPFMPGDSDLDQLTRIFSTLGTPTEAEWPHLSKLHDYLQ--FRNFPGTPLDNIFTAAGNDLIHLMQRLFAMNPLRRVSCREALSMPYF

>Dme-cdc2-1-gi|17738075|

FQRAEKIGEGTYGIVYKARSNSTGQDVALKKIRLEGETEGVPSTAIREISLLKNLKHPNVVQLFDVVISGNNLYMIFEYLNMDLKKLMDKKKDVFTPQLIKSYMHQILDAVGFCHTNRILHRDLKPQNLLVDTAGKIKLADFGLARAFNVPMRAYTHEVVTLWYRAPEILLGTKFYSTGVDIWSLGCIFSEMIMRRSLFPGDSEIDQLYRIFRTLSTPDETNWPGVTQLPDFKT-KFPRWEGTNMPQ--PITEHEAHELIMSMLCYDPNLRISAKDALQHAYF

>Dme-cdk5-gi|17137070|

YDKMEKIGEGTYGTVFKGRNRDTMEIVALKRVRLDEDDEGVPSSALREICLLKELKHKNIVRLIDVLHSDKKLTLVFEHCDQDLKKYFDSLNGEIDMAVCRSFMLQLLRGLAFCHSHNVLHRDLKPQNLLINKNGELKLADFGLARAFGIPVKCYSAEVVTLWYRPPDVLFGAKLYTTSIDMWSAGCILAELADGRPLFPGSDVLDQLMKIFRVLGTPNEDSWPGVSHLSDYVA--LPSFPATSWSQLVPRLNSKGRDLLQKLLICRPNQRISAEAAMQHPYF

>Dme-cdc2-gi|17136606|

FEKIEKIGEGTYGVVYKGRNRLTGQIVAMKKIRLESDDEGVPSTAIREISLLKELKHENIVCLEDVLMEENRIYLIFEFLSMDLKKYMDSLPVHMESELVRSYLYQITSAILFCHRRRVLHRDLKPQNLLIDKSGLIKVADFGLGRSFGIPVRIYTHEIVTLWYRAPEVLLGSPRYSCPVDIWSIGCIFAEMATRKPLFQGDSEIDQLFRMFRILKTPTEDIWPGVTSLPDYKN-TFPCWSTNQLTNQLKNLDANGIDLIQKMLIYDPVHRISAKDILEHPYF

>Dme-pitslre-gi|24667662|

FQCLNRIEEGTYGVVYRAKDKRTNEIVALKRLKMEKEKEGFPITSLREINTLLKGQHPNIVTVREIVVGMDKIFIVMDYVEHDLKSLMETMKQSFFPGEVKCLTQQLLRAVAHLHDNWILHRDLKTSNLLLSHKGILKVGDFGLAREYGSPIKKYTSLVVTLWYRAPELLLCSPVYSTPIDVWSVGCIFAEFLQMLPLFPGKSEIDELNRIFKELGTPNEKIWPGYTELPAVKNMQFTEYPVSQLRKHFQKTSEMGLSLLQGLLTYDPKQRLSADAALKHGFF

>Dme-cdc2rk-gi|17647247|

FEKLNRVGEGSYGIVYRARDTRSNEIVALKKVRMDQEKDGLPISGLREIMILKQCHHENIVRLREVVVGLDSIFLVMDFCEQDLASVLDNMSQPFTESEVKCITLQVLKALKYLHSRFMIHRDLKVSNLLMTDKGCIKVADFGLARMFSNPPKPMTPQMVTLWYRAPELLLGCRTHTTAVDMWAFGCILGELLLGKPLLPGNSEIAQLDMIIDLLGAPSESIWPGFADLPAVQNFTLSQQPYNNLTPKFHMIGQSGRNLLNILFIYNPKTRATAEECLKSKYF

>Dme-Eip63e-gi|24656896|

YVKLEPLGEGSYATVYKGFSKLTYQRVALKEIRLQ-EEEGAPFTAIREASLLKELKHSNIVTLHDIVHTRETLTFVFEYVNTDLSQYMEKHPGGLDHRNVRLFLFQLLRGLSYCHKRRVLHRDVKPQNLLISDCGELKLADFGLARAKSVPSHTYSHEVVTLWYRPPDVLLGSTEYSTSLDMWGVGCIFVEMVTGMPTFPGIRDYDQLDKIFKLLGTPTEDTWPGVTHFPGYKPHKLGFYRPRKLGHNFPRLIIEGETIANGFLQLNPEQRLGADDALQHPYF

>Dme-cdk9-gi|24658274|

YEKVAKIGQGTFGEVFKAREKKGKKFVAMKKVLMDNEKEGFPITALREIRILQLLKHENVVNLIEICRTRSTFYLVFDFCEHDLAGLLSNMNVKFSLGEIKKVMQQLLNGLYYIHSNKILHRDMKAANVLITKHGILKLADFGLARAFSIPKNRYTNRVVTLWYRPPELLLGDRNYGPPVDMWGAGCIMAEMWTRSPIMQGNTEQQQLTFISQLCGSFTPDVWPGVEELELYKSIELPKNQKRRVKERLRYKDQTGCDLLDKLLTLDPKKRIDADTALNHDFF

>Dme-cg7597-gi|24668137|

FEMIAQIGEGTYGQVYKARDHHTNDMVALKKVRLEHEKEGFPITAVREIKILRQLNHRNIVNLHEIVTDKGSFYLVFEYMDHDLMGLLESGMVDFNEENNASIMKQLLDGLNYCHKKNFLHRDIKCSNILMNNRGKVKLADFGLARLYNADERPYTNKVITLWYRPPELLLGEERYGPSIDVWSCGCILGELFVKRPLFQANAEMAQLETISKICGSPVPAVWPNVIKLPLFHTLKQKKTHRRRLREDFEFMPAPALDLLDKMLDLDPDKRITAEDALRSPWL

>Dme-cdk8-gi|24661985|

YE-GCKVGRGTYGHVYKAKWKETGKEYALKQI----DGTGLSMSACREIALLRELKHQNVITLIRVFLSDRKVFLLIDYAEHDLWHIIKFHQVVVPRGMVKSLLYQILDGIHYLHSNWVLHRDLKPANILVMERGRVKIADMGFARLFNAPLKPLDPVVVTFWYRAPELLLGARHYTKAIDIWAIGCIFAELLTSEPIFHCRQEHDQLDRIFNVMGFPQDKDWEDIKKMPEHHTLTFKRYSTCSLAKRHIKPDSKAFHLLQKLLLMDPNKRITSEQAMQDQYF

>Dme-cdk4-gi|17137264|

YQELNIIGEGAYGTVYRARDVITGNIVALKKVRISLNENGVPMSTLREISLLKQLNHANIVKLYEVCQFQLLILLVFEHVEQDLSDLIDRLPKGMSPPTIQRLSRELLTGVDFLHSHRIIHRDLKPQNLLVSSQGHLKIADFGLAKTYGSEM-KLTSVVVTLWYRAPEVLLAQ-PYNSTVDIWSAACIIFEMFNRRALFPGTSEKNQLDRIFELTGRPTEQQWPQTISVALEH---FPQRHPKRPKDFCPHLCKYADDLLNKMLSYDLHLRPSALACLEHDYF

>Spu-gi|115964384|

YSILDRIGEGAHGIVFKAKHIETGEIVALKKVPLRKLDDGIPNTALREIKALQEIENQYVVKLKDVFPHGTGFVLVFEFMLSDLSEVIRNSDQPLTEAQVKSYMLMLLKGITHCHENSIMHRDLKPANLLISETGHLKIADFGLARVFSNDGRQYSHQVATRWYRAPELLYGARKYDEGADLWAVGCIFGELLNNSPIFPGENDIEQLCCVLRILGTPTEKTWPGMKDLPDYNKITFPENPPIPLEQIVPDASPEALDLLKKFLVYPSRQRISASEALLHPYF

>Spu-gi|115702469|

FEKIEKIGEGTYGVVYKARDLKSGKTVALKKIRLDTESEGVPSTAIREIALLKELDHKNIVKLHDVVHSDKKLYLVFEFMNQDLKKYMDVAPPGLPPGLVKSYLHQLLQGIAFCHAHRVLHRDLKPQNLLIDADGRIKLADFGLARAFGVPVRTYTHEVVTLWYRAPEILLGCRYYSTAVDIWSLGCIFVEMITRRALFPGDSEIDQLFRIFRTMGTPDEKLWPGVTSLPDYKT-SFPRWTPQDFTKIVPMLNKDGKDLLKSMLCYEPDKRISAKTGLSHPYF

>Spu-gi|72012799|

FMKIEKLGEGTYGVVYKGKHKRTGKIVALKKIRLESEEEGVPSTAIREISLLKELYHPNIVMLEDVLMEPNRLYLVFEYLTMDLKKYMESLKGQMDPALVKSYLHQMVDGILFCHSRRILHRDLKPQNLLIDNNGTIKLADFGLARAFGIPVRVYTHEVVTLWYRAPEVLLGSTRYACPIDMWSLGCIFAEMVTKRPLFHGDSEIDQLFRIFRTLGTPTDDIWPGVTQLQDYKS-TFPMWTKPNIKGAVKGMDEGGLDLLEQMLIYDPAKRITAKASMRHPYF

>Spu-gi|72158568|

FEKLNRVGEGTYGIVYRARDMKSKEIVALKKVRMEKEKDGLPISGLREIHLLINLRHENVVELHEVVVGLDSIFLVMQYCEQDLASLLDNMPSPFTETQVKCLALQMLRGLRYLHDNFVIHRDLKVSNLLLADNGCLKIADFGLARRYGLPVRPMTPRVVTLWYRAPELLFGSLEQTTAIDMWAAGCILGELLVNKPLMPGASELHQINHIIDLLGTPNDTIWPGFSELPMVQNFTLKKQPYNNLKAKFTWLSQSGLRLLNFLLMYNPKKRATAEESLESSYF

>Spu-gi|115954076|

FQCLNRIAEGTYGVVYRANEKKKNDIVALKRLKMEKEKEGFPITSLREISTLLKAQHRNIVTVREIVVGMDKIYIVMDYVEHDLKSLMETMKQPFTIGETKCLILQLLRGVHHLHDNWILHRDLKTSNLLLNHQGCLKIGDFGLAREYGSPIKPYTSIVVTLWYRAPELLLGTKVYSTPIDMWSVGCIFAEFLTIKPIFNGRSEIDQLNRIFKELGTPSEKIWPGYNELPAVKKTTFAHHPYNNLRNRFGYLTDVGFELLNRFLTYDPVRRISAEDALKHPYF

>Spu-gi|72004784|

YKKICPLGEGSYAKVYKAVSCINQQVVALKEIRLQ-QDEGTPFTAIREASLLKDLKHANIVCLHDIIHTKTTLSFVFEYVHTDLSTYLERHPGGLNPNNVRLFLFQLIRGLSFCHKRKILHRDLKPQNLLISEAGELKLADFGLARAKSIPSRTYSHEVVTLWYRPPDVLMGSTDYSTQLDIWGVGCIFLEMMWGQPAFPGLKDTDQLEKIFKVLGTPTEQTWHGVSRLPNYGKVAAAFHKPKLLCEILPSIIPGAESLMTDMLQLEPRKRISTVQAMTCQYF

>Spu-gi|72087356|

FEIINQIGEGTYGQVYKARDKDTGELVALKKVRTDNEKEGFPITAVREIKILRQLNHDSVIRLHEIVTDKGAFYLVFEYMDHDLMGLLESGLVNFSEEHVRSFMKQLLDGLNYCHKRNFLHRDIKCSNILLNNKGHIKLADFGLARLYHADTRPYTNKVITLWYRPPELLLGEERYGPAVDVWSCGCILGELFTQRPIFQANQELAQLELISRICGTPTPAVWPDVIRLPLFNTMKPKKMYNRRLREEFSLLPKDALDLLDGMLTLDPDKRTTAEDALNCGWL

>Spu-gi|115682385|

AKEYYTTAERTYGTVFKAKNRETQEIVALKRVRLDDDDEV--ITVVRKIVTQSECNFFSLPSLHENS--DDMYNKVNEYCDEMFIYSLCR-----VNVIVESFMYQLLRGLAFCHSHHVLHRDLKPQNLLINKNGELKLADFGLARAFGIPVRCYSAEVVTLWYRPPDVLFGAKVYTTSIDMWSAGCIFAEMANGRPLFPGNDVEDQLKRIFKLLGTPTEDTWPGISKLPDFKPY--PIYPVTPLASVVPSLSATGRDLLQRLMMCNPALRMSAEEGLMHQYF

>Spu-gi|72136390|

YDKLAKIGQGTFGEVFKAKHKKNKNIVALKKVLMENEKEGFPITALREIRILQLLRHENVVPLYEICRTKGSIYLVFEFCEHDLAGLLSNTNVKFSLGEIKSVIKQLLNGLYYIHSNKVLHRDMKAANILITKAGVLKLADFGLARAFSLPPNRYTNRVVTLWYRPPELLLGERNYGPAIDLWGAGCIMAEMWTRSPIMQGNTEQHQLTLISHLCGSITSAVWPGLEKLELSNTLELPKGHKRKVKDRLRYKDQQALDLIDKLLNIDPKRRMDAAIALDHDFF

>Spu-gi|47551029|

YVQAAEIGSGAYGIVYKARDTETGHFVALKSVRIPIGEEGMPVSTIREISLLRHLDHPNIVKLLDVCDVEMMLTLVFELVDQDLAQYLEKCPPGLSSCTIKFLMHQLLSGVEYLHSHRVTHRDLKPQNILVASDKKLKLTDFGLSRVYSFQM-ALTPVVVTMWYRAPEVLLQA-SYATPVDMWSVGCIFAELYRRRPLFRGQSDKDQLHKIFEVIGLPPEDQWPDVA-LPWSS---FRQTGQRSFLDLVQEICNQGLDLLERMLCFNPDHRMTAEQGLLHGFF

>Spu-gi|115722990|

FE-GCKVGRGTYGHVYKATTKKEKKEYALKQI----EGTGISMSACREVALLRELRHPNVICLRRVFLSDRKVYLLCDFSEHDLWHIIKYHPTSVPPVMVKSLLYQILDGIHYLHSNWVLHRDLKPANILVMERGRVKIADMGFARLFNSPLKPLDPVVVTFWYRAPELLLGARHYTKAIDIWAIGCIFAELLTSEPIFHCRQEHDQLDRIFNVMGFPQGRIHASTIK-------------------------------------------------------

1. **Multiple alignments for phylogenetic analysis of CDK family proteins in H. sapiens, *T.* adhaerens, *C.owczarzaki , S. arctica, S.cerevisiae, S.pombe, C. cinerea, S. punctatus, T. trahens* and *D. discoideum.***

>Hsa-CDK6

YECVAEIGEGAYGKVFKARDLNGGRFVALKRVRVQTGEEGMPLSTIREVAVLRHLEHPNVVRLFDVCTVETKLTLVFEHVDQDLTTYLDKVPPGVPTETIKDMMFQLLRGLDFLHSHRVVHRDLKPQNILVTSSGQIKLADFGLARIYSFQM-ALTSVVVTLWYRAPEVLLQS-SYATPVDLWSVGCIFAEMFRRKPLFRGSSDVDQLGKILDVIGLPGEEDWPRDVALPRQA---FHSKSAQPIEKFVTDIDELGKDLLLKCLTFNPAKRISAYSALSHPYF

>Hsa-CDK4

YEPVAEIGVGAYGTVYKARDPHSGHFVALKSVRVPNGGGGLPISTVREVALLRRLEHPNVVRLMDVCATEIKVTLVFEHVDQDLRTYLDKAPPGLPAETIKDLMRQFLRGLDFLHANCIVHRDLKPENILVTSGGTVKLADFGLARIYSYQM-ALTPVVVTLWYRAPEVLLQS-TYATPVDMWSVGCIFAEMFRRKPLFCGNSEADQLGKIFDLIGLPPEDDWPRDVSLPRGA---FPPRGPRPVQSVVPEMEESGAQLLLEMLTFNPHKRISAFRALQHSYL

>Hsa-CDK2

FQKVEKIGEGTYGVVYKARNKLTGEVVALKKIRLDTETEGVPSTAIREISLLKELNHPNIVKLLDVIHTENKLYLVFEFLHQDLKKFMDASATGIPLPLIKSYLFQLLQGLAFCHSHRVLHRDLKPQNLLINTEGAIKLADFGLARAFGVPVRTYTHEVVTLWYRAPEILLGCKYYSTAVDIWSLGCIFAEMVTRRALFPGDSEIDQLFRIFRTLGTPDEVVWPGVTSMPDYKP-SFPKWARQDFSKVVPPLDEDGRSLLSQMLHYDPNKRISAKAALAHPFF

>Hsa-CDK3

FQKVEKIGEGTYGVVYKAKNRETGQLVALKKIRLDLEMEGVPSTAIREISLLKELKHPNIVRLLDVVHNERKLYLVFEFLSQDLKKYMDSTPSELPLHLIKSYLFQLLQGVSFCHSHRVIHRDLKPQNLLINELGAIKLADFGLARAFGVPLRTYTHEVVTLWYRAPEILLGSKFYTTAVDIWSIGCIFAEMVTRKALFPGDSEIDQLFRIFRMLGTPSEDTWPGVTQLPDYKG-SFPKWTRKGLEEIVPNLEPEGRDLLMQLLQYDPSQRITAKTALAHPYF

>Hsa-CDK1

YTKIEKIGEGTYGVVYKGRHKTTGQVVAMKKIRLESEEEGVPSTAIREISLLKELRHPNIVSLQDVLMQDSRLYLIFEFLSMDLKKYLDSIPQYMDSSLVKSYLYQILQGIVFCHSRRVLHRDLKPQNLLIDDKGTIKLADFGLARAFGIPIRVYTHEVVTLWYRSPEVLLGSARYSTPVDIWSIGTIFAELATKKPLFHGDSEIDQLFRIFRALGTPNNEVWPEVESLQDYKN-TFPKWKPGSLASHVKNLDENGLDLLSKMLIYDPAKRISGKMALNHPYF

>Hsa-CDK5

YEKLEKIGEGTYGTVFKAKNRETHEIVALKRVRLDDDDEGVPSSALREICLLKELKHKNIVRLHDVLHSDKKLTLVFEFCDQDLKKYFDSCNGDLDPEIVKSFLFQLLKGLGFCHSRNVLHRDLKPQNLLINRNGELKLADFGLARAFGIPVRCYSAEVVTLWYRPPDVLFGAKLYSTSIDMWSAGCIFAELANGRPLFPGNDVDDQLKRIFRLLGTPTEEQWPSMTKLPDYKP--YPMYPATSLVNVVPKLNATGRDLLQNLLKCNPVQRISAEEALQHPYF

>Hsa-CDK16

YIKLDKLGEGTYATVYKGKSKLTDNLVALKEIRLEH-EEGAPCTAIREVSLLKDLKHANIVTLHDIIHTEKSLTLVFEYLDKDLKQYLDDCGNIINMHNVKLFLFQLLRGLAYCHRQKVLHRDLKPQNLLINERGELKLADFGLARAKSIPTKTYSNEVVTLWYRPPDILLGSTDYSTQIDMWGVGCIFYEMATGRPLFPGSTVEEQLHFIFRILGTPTEETWPGILSNEEFKTYNYPKYRAEALLSHAPRLDSDGADLLTKLLQFEGRNRISAEDAMKHPFF

>Hsa-CDK17

YIKLEKLGEGTYATVYKGRSKLTENLVALKEIRLEH-EEGAPCTAIREVSLLKDLKHANIVTLHDIVHTDKSLTLVFEYLDKDLKQYMDDCGNIMSMHNVKLFLYQILRGLAYCHRRKVLHRDLKPQNLLINEKGELKLADFGLARAKSVPTKTYSNEVVTLWYRPPDVLLGSSEYSTQIDMWGVGCIFFEMASGRPLFPGSTVEDELHLIFRLLGTPSQETWPGISSNEEFKNYNFPKYKPQPLINHAPRLDSEGIELITKFLQYESKKRVSAEEAMKHVYF

>Hsa-CDK18

YVKLDKLGEGTYATVFKGRSKLTENLVALKEIRLEH-EEGAPCTAIREVSLLKNLKHANIVTLHDLIHTDRSLTLVFEYLDSDLKQYLDHCGNLMSMHNVKIFMFQLLRGLAYCHHRKILHRDLKPQNLLINERGELKLADFGLARAKSVPTKTYSNEVVTLWYRPPDVLLGSTEYSTPIDMWGVGCIHYEMATGRPLFPGSTVKEELHLIFRLLGTPTEETWPGVTAFSEFRTYSFPCYLPQPLINHAPRLDTDGIHLLSSLLLYESKSRMSAEAALSHSYF

>Hsa-CDK14

YEKLEKLGEGSYATVYKGKSKVNGKLVALKVIRLQE-EEGTPFTAIREASLLKGLKHANIVLLHDIIHTKETLTLVFEYVHTDLCQYMDKHPGGLHPDNVKLFLFQLLRGLSYIHQRYILHRDLKPQNLLISDTGELKLADFGLARAKSVPSHTYSNEVVTLWYRPPDVLLGSTEYSTCLDMWGVGCIFVEMIQGVAAFPGMKDQDQLERIFLVLGTPNEDTWPGVHSLPHFKPERFTLYSSKNLRQAWNKLVNHAEDLASKLLQCSPKNRLSAQAALSHEYF

>Hsa-CDK15

YLNLEKLGEGSYATVYKGISRINGQLVALKVISMNA-EEGVPFTAIREASLLKGLKHANIVLLHDIIHTKETLTFVFEYMHTDLAQYMSQHPGGLHPHNVRLFMFQLLRGLAYIHHQHVLHRDLKPQNLLISHLGELKLADFGLARAKSIPSQTYSSEVVTLWYRPPDALLGATEYSSELDIWGAGCIFIEMFQGQPLFPGVSNLEQLEKIWEVLGVPTEDTWPGVSKLPNYNPEWFPLPTPRSLHVVWNRLVPEAEDLASQMLKGFPRDRVSAQEALVHDYF

>Hsa-CDK11B

FQCLNRIEEGTYGVVYRAKDKKTDEIVALKRLKMEKEKEGFPITSLREINTILKAQHPNIVTVREIVVGMDKIYIVMNYVEHDLKSLMETMKQPFLPGEVKTLMIQLLRGVKHLHDNWILHRDLKTSNLLLSHAGILKVGDFGLAREYGSPLKAYTPVVVTLWYRAPELLLGAKEYSTAVDMWSVGCIFGELLTQKPLFPGKSEIDQINKVFKDLGTPSEKIWPGYSELPAVKKMTFSEHPYNNLRKRFGLLSDQGFDLMNKFLTYFPGRRISAEDGLKHEYF

>Hsa-CDK10

FEKLNRIGEGTYGIVYRARDTQTDEIVALKKVRMDKEKDGIPISSLREITLLLRLRHPNIVELKEVVVGLESIFLVMGYCEQDLASLLENMPTPFSEAQVKCIVLQVLRGLQYLHRNFIIHRDLKVSNLLMTDKGCVKTADFGLARAYGVPVKPMTPKVVTLWYRAPELLLGTTTQTTSIDMWAVGCILAELLAHRPLLPGTSEIHQIDLIVQLLGTPSENIWPGFSKLPLVGQYSLRKQPYNNLKHKFPWLSEAGLRLLHFLFMYDPKKRATAGDCLESSYF

>Hsa-CDK9

YEKLAKIGQGTFGEVFKARHRKTGQKVALKKVLMENEKEGFPITALREIKILQLLKHENVVNLIEICRTKGSIYLVFDFCEHDLAGLLSNVLVKFTLSEIKRVMQMLLNGLYYIHRNKILHRDMKAANVLITRDGVLKLADFGLARAFSLAKNRYTNRVVTLWYRPPELLLGERDYGPPIDLWGAGCIMAEMWTRSPIMQGNTEQHQLALISQLCGSITPEVWPNVDNYELYEKLELVKGQKRKVKDRLKARDPYALDLIDKLLVLDPAQRIDSDDALNHDFF

>Hsa-CDK13

FDIIGIIGEGTYGQVYKARDKDTGEMVALKKVRLDNEKEGFPITAIREIKILRQLTHQSIINMKEIVTDKGAFYLVFEYMDHDLMGLLESGLVHFNENHIKSFMRQLMEGLDYCHKKNFLHRDIKCSNILLNNRGQIKLADFGLARLYSSESRPYTNKVITLWYRPPELLLGEERYTPAIDVWSCGCILGELFTKKPIFQANQELAQLELISRICGSPCPAVWPDVIKLPYFNTMKPKKQYRRKLREEFVFIPAAALDLFDYMLALDPSKRCTAEQALQCEFL

>Hsa-CDK12

FDIIGIIGEGTYGQVYKAKDKDTGELVALKKVRLDNEKEGFPITAIREIKILRQLIHRSVVNMKEIVTDKGAFYLVFEYMDHDLMGLLESGLVHFSEDHIKSFMKQLMEGLEYCHKKNFLHRDIKCSNILLNNSGQIKLADFGLARLYNSESRPYTNKVITLWYRPPELLLGEERYTPAIDVWSCGCILGELFTKKPIFQANLELAQLELISRLCGSPCPAVWPDVIKLPYFNTMKPKKQYRRRLREEFSFIPSAALDLLDHMLTLDPSKRCTAEQTLQSDFL

>Hsa-CDK7

YEKLDFLGEGQFATVYKARDKNTNQIVAIKKIKLGEAKDGINRTALREIKLLQELSHPNIIGLLDAFGHKSNISLVFDFMETDLEVIIKDNSLVLTPSHIKAYMLMTLQGLEYLHQHWILHRDLKPNNLLLDENGVLKLADFGLAKSFGSPNRAYTHQVVTRWYRAPELLFGARMYGVGVDMWAVGCILAELLLRVPFLPGDSDLDQLTRIFETLGTPTEEQWPDMCSLPDYVT--FKSFPGIPLHHIFSAAGDDLLDLIQGLFLFNPCARITATQALKMKYF

>Hsa-CDK19

FEEGCKVGRGTYGHVYKARRKDGEKEYALKQI----EGTGISMSACREIALLRELKHPNVIALQKVFLSDRKVWLLFDYAEHDLWHIIKFHPMQLPRSMVKSLLYQILDGIHYLHANWVLHRDLKPANILVMGRGRVKIADMGFARLFNSPLKPLDPVVVTFWYRAPELLLGARHYTKAIDIWAIGCIFAELLTSEPIFHCRQEHDQLDRIFSVMGFPADKDWEDIRKMPEYPTLQFRRYANSSLIKHKVKPDSKVFLLLQKLLTMDPTKRITSEQALQDPYF

>Hsa-CDK8

FEEGCKVGRGTYGHVYKAKRKDGDKDYALKQI----EGTGISMSACREIALLRELKHPNVISLQKVFLSDRKVWLLFDYAEHDLWHIIKFHPVQLPRGMVKSLLYQILDGIHYLHANWVLHRDLKPANILVMGRGRVKIADMGFARLFNSPLKPLDPVVVTFWYRAPELLLGARHYTKAIDIWAIGCIFAELLTSEPIFHCRQEHDQLDRIFNVMGFPADKDWEDIKKMPEHSTLMFRRYTNCSLIKHKVKPDSKAFHLLQKLLTMDPIKRITSEQAMQDPYF

>Hsa-CDK20

YCILGRIGEGAHGIVFKAKHVETGEIVALKKVALRRLEDGFPNQALREIKALQEMENQYVVQLKAVFPHGGGFVLAFEFMLSDLAEVVRHAQRPLAQAQVKSYLQMLLKGVAFCHANNIVHRDLKPANLLISASGQLKIADFGLARVFSPGSRLYTHQVATRWYRAPELLYGARQYDQGVDLWSVGCIMGELLNGSPLFPGKNDIEQLCYVLRILGTPNPQVWPELTELPDYNKISFKEQVPMPLEEVLPDVSPQALDLLGQFLLYPPHQRIAASKALLHQYF

>Hsa-CDKL1

YEKIGKIGEGSYGVVFKCRNRDTGQIVAIKKFLESEDDPVIKKIALREIRMLKQLKHPNLVNLLEVFRRKRRLHLVFEYCDHTVLHELDRYQRGVPEHLVKSITWQTLQAVNFCHKHNCIHRDVKPENILITKHSVIKLCDFGFARLLTGPSDYYTDYVATRWYRSPELLVGDTQYGPPVDVWAIGCVFAELLSGVPLWPGKSDVDQLYLIRKTLGDLIPRHQQVFSTNQYFSGVKIPDEDMEPLELKFPNISYPALGLLKGCLHMDPTQRLTCEQLLHHPYF

>Hsa-GSK3alpha

YTDIKVIGNGSFGVVYQARLAETRELVAIKKVLQDKR------FKNRELQIMRKLDHCNIVRLRYFFYSELYLNLVLEYVPETVYRVARHAKLTIPILYVKVYMYQLFRSLAYIHSQGVCHRDIKPQNLLVDPTAVLKLCDFGSAKQLVRG-EPNVSYICSRYYRAPELIFGATDYTSSIDVWSAGCVLAELLLGQPIFPGDSGVDQLVEIIKVLGTPTREQIREMN--PNYTEFKFPQIKAHPWTKVFKRTPPEAIALCSSLLEYTPSSRLSPLEACAHSFF

>Hsa-MAK

YTTMRQLGDGTYGSVLMGKSNESGELVAIKRMKRKF-YSWDECMNLREVKSLKKLNHANVIKLKEVIRENDHLYFIFEYMKENLYQLMKDRNKLFPESVIRNIMYQILQGLAFIHKHGFFHRDMKPENLLCMGPELVKIADFGLARELRSQ-PPYTDYVSTRWYRAPEVLLRSSVYSSPIDVWAVGSIMAELYMLRPLFPGTSEVDEIFKICQVLGTPKKSDWPEGYQLASSMNFRFPQCVPINLKTLIPNASNEAIQLMTEMLNWDPKKRPTASQALKHPYF

>Tad-gi|196001971|

YKKIKLLGEGQFAVVYQAEDTHDNKIVAVKKIKLGEANDGINRTALREIKLLQELKHENIIGLLDVFGHKSNISLVFDYMEADLEVIIKDSSIILTNAHIKQYIIMTLRGLEYIHSNWILHRDMKPNNLLIDSNGILKLADFGLARYFGSPNRYYTNQVVTVWYRAPELLFGANSYGTGVDMWAVGCILAELLLRVPFLPGDSDLDQLTRIFQTLGTPIEDKWADIHKLPGYIK--FKTLPAIPLNEIFTAASDDMLDLLRRLFEYNPSSRITATEALQMKYF

>Tad-gi|195996663|

FTILGRVGEGAHGVVFKAKHVGNGEVVALKKIHLKKIDDGIPNNILREIKTLQAITHENIVRLFDVFPDGSSLVLAFEYMVTDLSEILRSSQNTL-PEVSSYYMIMLLRGISFCHENHIIHRDLKPANLLISSSGQLKLADFGLARVMSVNERLYSHQVATRWYRAPELLYGSRTYDEGVDLWYLGCIFAELINKSPLFPGDSDIKQLGCVLSILGTPTTSTWPGLIELPDYNKITFSGFLPIPFESIVPDATSE----VGKIK-------------------

>Tad-gi|196013334|

FEWLNRIEEGTYGVVYRAKDLKSDEVVALKRLKMEKEREGFPITSLREINTLLKADHPNIVHVREIVVGMDKIYIVMEYVEHDLKTLMESMSQPFSISEVKCLMKQLLSAVQHLHDNWILHRDLKTSNLLLSHQGILKVGDFGLAREYGSPLKVYTSIVVTLWYRCPELLLGVKEYSTAVDMWSVGCIFGEFLVKKPLFPGKSEIDQLNKIFKDLGTPNDQIWSGFSELPVAKKVTFTEQPYNRLRDRFGYLTDQGFDLLNRFLTYDPKKRISAEDALNHEYF

>Tad-gi|196010283|

FDKLNRIGEGSYGVVYRARDLDSKEIVAIKKIRMENERDGIPVSSLREITLLVNLKHINIVNLKDVVVGLDSIFLVMEYCEQDLSSLLYDMKAPFTEPQVKCLSLQLIHGVQYLHHNFVIHRDLKVSNLLLTDKGILKVADFGLARNYGLPAAPMTPTIVSLWYRAPEVLLGCTKHTLAVDMWSVGCIIAELFDHNVFLKGKSEKDQLDLMCQMLGTPNEAIWEDIRDMPLYGKIILRQQPYNNLKHKFSWLSAAGLNLLNSLLTYDPGRRITADETLKLSYF

>Tad-gi|196002669|

--ILKQIGEGTYGQVFKAKIKDTDKLVALKKVRTDHEREGFPITAVREIKILKQLNHQSIVNLLGIVSNRCAFYLVFEYMDHDLMGLLESGLVTFDEEHIRSFMRQIMEGLNYCHKRQLLHRDIKCSNLLMNNKGQIKIADFGLARFYNPDSRPYTNKVITLWYRPPELLLGEERYGPSVDVWSCGCILGEFFTKKPIFQANSEINQLDLISQICGTPCPENWPSVVELPYYNN--FKRKYERRLEQEFHDLPELAVDLMQYMLILDPSMRYNAEQSLQHPFI

>Tad-gi|195998898|

YEKITKIGHGTFGEVFKARNRISKEFVALKRVLLGNEKEGFPITSLREIKILRALKHDNIVRLQEICRSRGSIYLVFEFCAHDLAGLLQNPQVKFNLSEIKRMMKHLLSGLFYIHSNKVLHRDLKAANVLVTRDGVLKLADFGLARVYSRKEHCFTNRVVTLWYRAPELLLGCRDYGPAIDMWAIGCIMAEFWTRSAIMQGNSETNQLTLITQLCGSITPEVYPDVDKLDLFKKFDLPASQKRRVKERLSHRDRHALDLIDRCLTIDPAKRIDSDSALNHDFF

>Tad-gi|196013348|

FHKLEKIGEGTYGKVYKACNKITGQTVALKKIRLDSDKEGVPSTTLREISILRSLNHSFVVRLYDVVHSDQCLYLVFEYLDHDLKHYLDHA-YKIPPALLKSYLYQMLRAISYCHSRRVLHRDLKPQNLLIDSTGTLKLADFGLARIFGLPVRQYTHEVITLWYRAPEILLGSTYYSTPVDIWSIGCIFVEMINRRPLFAGDSEIDQLFRVFRTLGTPDEITWPGVSEMSDYKS-TFPKWPSRDLNSVIYSHDEDCVDLIKQMLVYEPNGRISARLALQHPYF

>Tad-gi|196003954|

YLKIEKIGEGTYGVVYKGKNRNTQQLVALKKIRLENEEEGIPSTAIREVSLLKELKHPNIVDLIEVLYEESKLYLVFEFLDMDLKRYLDTLPKTIDAMLMKSYLYQILLGVVYCHSHRVLHRDLKPQNLLINSKGCIKLADFGLGRAFGVPVRVYTHEVVTLWYRAPEVLLGSTRYSCPLDIWSTGTIFAEMWLRRPLFQGDSEIDELFRIFRILGTPDDDIWPGVSSLPEFKS-SFPKWSKQSYDTFVPNMSESGIDLLSKMLIYDPANRISGKRALSHPYF

>Tad-gi|196001193|

YEKLEPLGEGSYATVYKGYSIGHQKLVALKEITLNE-EEGTPFTAIREASLLKQLKHANIVVLHDIIQTPTKLTFVFEYVTTDLSQYLNLHPGGLNMKNVKLFLYQLLRGLSYCHQRRILHRDIKPQNILVSEIGEIKLADFGLARAKSVPSKTYSPDVVTLWYRPPDVLLGSTNYSTSLDIWGVGCIFTEMVSGIATFPGVRNSDQLDKIWHKLGTPTEETWPGVTSYPEYCAAETMFYESRTIAEVIPRLDPGAADLATKMLQYQPFKRIFCQAAMNHQYF

>Tad-gi|195996637|

YDKLHKLGEGTYATVFKGRSKLTNDFVALKEIRLEH-EEGAPCTAIREVSLLKDLKHANIVTLHDTIHTERSLVLVFEYLDRDLKQYMDSCGSILDMSNVKIFLFQLLRGLAYCHSRRVLHRDLKPQNLLINERGELKLADFGLARAKSVPSKTYSSEVVTLWYRPPDVLLGSTEYSTSIDMWGVGCIFYEMATGLPMFPGSSTENQLQTIWEILGTPTEEEWSGLTRNLKVNSLSFHDCKGEPLRNRAPRLEADGLDLLAKFLQYKAKSRISSADAMKHKYF

>Tad-gi|196000717|

YEKLVKIGEGTYGTVYKAVNHDTGEIVALKKVRIDDENEGIPSFALREICLLKELKHKNIVMLYDVIHGNKELMIVFEYCDQDLKRYCDACQGKIDPSIVQSFTNQLLQGLAYCHSHHILHRDITPQNILVTGNGDIKLADFGLARNFGIPVKSFSAEVVTLWYRSPDVLLGATLYDTSIDIWSTGCIFAELSNGQPLLPGKDVADQLKIIFKIFGTPNEQIWPGVSQLMKDKD--YPSYNAMSILHVVPNLNQLGCDLFQLMMVLDPSKRCTAEQALQHAYF

>Tad-gi|195999760|

YVQVTEIANGAYGRVYKARDLNHNRLVALKKIAVINDEQGIPISTIREITSLKSLGHQNIVRLYDIFANTMDLTLVFEHVEQDLQTYIRNCPAGIDTRKIKDIIYQIVNAIDFLHANRIVHRDLKPQNVLISRQGIVKVADFGLAKVFCENV-PITSVVVTLWYRCPEVLLQS-TYATPVDMWSVGCIMAELYLREPLFCGQTDIDQLQKIFSMTGLPDESEWPVNIPFSRST---FCQYTRRQYSEMMPEICQDGVDLLQKLLKFNPKERLTAEESLQHPYF

>Tad-gi|196015356|

FEEGCKVGRGTYGHVYKAKMKSSGKEYALKLI----EGSGISMSACREIALLREVHHPNVISLQGVFLTNRKVWLLFDFAEHDLWHIIKYHAISLDSKMVKSLLKQILEGIHYLHANWILHRDLKPANILVMGRGRVKIADMGFARHYWSPLKPLDPVVVTSWYRAPELLLGARHYTKAIDIWAIGCIFAELLTSEPIFHCRQERDQLERIFMVMGYPHEKDWEDIKKTPNYAKLAFRKYTNCSLAKFKIKQDSKEFILLQKCLTIDPNKRISSEQAIDDAYF

>Tad-gi|196015366|

FDEGCKVGRGTYGHVYKAKMKTSGKEYALKLI----EGTGISMSACREIAILREISHTHVISLQGVFLTSRKVWLLLDFAEHDLWHIIKFHSVPIDTKIVKSLLRQILDGIQYLHSNWILHRDLKPANILVMGRGRVKIADMGFARHFWAPLKPLDPVVVTFWYRAPELLLGARHYTKAIDIWAIGCIFAELLTSEPIFHCRQEHDQLDRIFTVMGFPHERDWEDIKIMPEYKRLQFKKFASCSLAKFKIRHDSREFSLLQKFLIADPNKRISAELAMDDAYF

>Cow-gi|320162599|

YDRIEKLGEGTYGE--------TGEIVALKSIRLDNEDEGVPCTAIREISLLKELKHPNIVRLHDVLHADKRLTLVFEYCDQDLKKYLDECAGDIGVMTMKSFLFQLLRGIAFCHEHRILHRDLKPQNLLINKRGELKLADFGLARAFGIPVRAYSHEVVTLWYRAPDVLLGSRRYSTSIDIWSAGCIFAEMAMGRPLFPGSSTLDQLMRIFKVLGTPNEEIWPGVSSLPEWKP-DFSVCRRVPLSSVVTTVDSYGIDLLARMLMYLPDARISADDAMCHPYF

>Cow-gi|320167054|

YKKLEKLGEGTYAIVYKGMSCITGDYVALKEIKLEQ-EEGYPCTALREVTLLKELKHANVVTLHDVIPAESSLTLVFEYVPMDLKNCMDKSLGFLDLFNIKLYMFQLLRGLAFCHRKKILHRDLKPQNLLIHHNGELKLCDFGLARAKGVPIKTFTNEVVTLWYRPPDVLMGSTDYTSSIDVWSAGCIFAEMVGGRPLFPAANPTEELLLIFKTRGTPNPQSFPNIEKLPGYST-SFPQYPVQPLSSFAPRLSADGLDLLEKMLQLDPSKRVTCEEAMRHGYF

>Cow-gi|320165016|

YELSTKLGEGTYASVFRAIHKPSSTLVALKQINLNR-DEGTPCTALREISLLKELRHANIVALLDVAHTRERLTLIFEHLDCDLKQHMDACGKNLAPANVQLILYQVLRGIAYCHSKSILHRDLKPQNLLLNRTGDVKLADFGLARAFGIPVKAFSHEVVTLWYRPPDVLMGSQVYSTSIDMWSIGCIFGEMTTGRPLFAGKNVDEQLARIFKQRGTPTELTWPGVSQLPNFRG-DFPVTPAVQLASIVPKMDSLGVTLLNRLLQYNPAMRVSAAEALQHVYF

>Cow-gi|320166971|

YERLDFLGEGQFATVYRARNKSTGEIVAIKKINLGDAQNGLNRTALREIKLLQELHHTNVIGLVDVFGHSNNISIVFEFMDADLEKIIRDPRNVFQPGDYKSFMLMTLQGIEYMHDRWILHRDLKPNNLLISGAGVVKLADFGLARDYGSPDKIYTNQVVTLWYRAPELLYGARCYGTGIDIWATGCIFAELLLRKALLPGNNEMSQLTQICSLFGAPTEKTWPGVTSLPTYVS--VKDYQPTPLRQLFTAASPDCVDLIGKMLTMNPSGRCTATEALQHAYF

>Cow-gi|320167092|

FDADYKIGEGTYGVVYKARKKIVPIPYALKKIKATVQTEGLSTSACREISLLREIKHENVICAPEIFLNTRDVWLLFDYAEYDLYQIIEYYPVFLEEHMIKSLLWQSLNGIHYLHSNWILHRDLKPANILVMGHGRVKIGDLGMARLFQSPLRNLDPVVVTIWYRAPELLLGAKHYTKAIDMWAIGCIFGELITARPIFLGEQIGDQLDKIFQVLGFPNEKDWPELSSLPDHPILMFLNYDKCSIEHQRGPLSPAGLGLLSEMLTMDPNKRITAEAALRSNYF

>Cow-gi|320165708|

FDKGNKVGDGQFGEVFRAYDKLNNRPVALKSVKMQEEQEGFPITSVREIKILRQSSHENIVRLYDLVREPETFYLCFEFMDCDLEALIKTPTVVLSSGIIKCYVKQIMTGLNHMHLNNVVHRDLKAANILINNRGQLKLGDFGLARVLLEKKGGYTNRVVTLWYRCPELLLGDTAYNTAIDMWSVGCIVYEMYTRSTLFREESEMAMLRKIIELCGSPAGESWPDVEKLPNFKDVSGLN-CKRRVREALNSQDLEAVNLIDCLLTLNPAKRYSATQCLDHDYF

>Cow-gi|320165526|

YIPLGKVGEGSYGTVYKVVATFHEKFYALKKIKSRTQSNGMPRAVIREVSILRELSHDNIISLVDVIYFDTESHRFLQWLQENFEQVLRRAKDRLHPLTIKAIMWQLLQGLDYLHDNWIVHADLKPNNILISSQGQVRIGDFGLSRLFLGPREAQEPVLVTMWYRAPELLLGDSKCSRAIDMWSVGCILAEMFLLQPIFRGKPEQDQAMKIFHALGLPTVEAWPEVTTMRFWPLIEHPKVYKFSLDSVLPD-ASIELQLLRGLFTFDPSKRLRTRQALTHAYF

>Cow-gi|320167411|

YVILEQVGQGAYGKVYKASNRATGEIVALKRIRLDREKEGFPVSAVREINLLRRLRHPNILCLKDIVTESDSVYLVFEYMDHDLSGLLHSGLMQFSVDQVRSLMYQIIQGVNHCHQNNIFHRDLKGANLLVNKHGEVKLADFGLARAFIEERRAYTNEVITLYYRPPELLLKETEYGPEVDVWSCGCILAEMLGNQVAFPGRTETEQLDLIARVCGTPCEENWPGVSNLLQQTT--FKRQYPRRLHEHFHSFPPDALDLLDKLLVLDPRRRITAAEALNHPFF

>Cow-gi|470363760|

YTRLNQIEEGSYGVVFRARDVRSGRIYALKRLKMEKEKDGFPITSLREIDTLLKSPHPNIVLVREIVVGMDHIFLVMEFVEHDLKTLMESMRQPFSGGEVKTLMLHLLAGVNHLHDNWIIHRDLKTSNLLLSNQGVLKLADFGLAREYGSPLHAMTALVVTLWYRSPELLLGETKYTTAVDMWSVGCIFAELLIHEPLFPGQRELQQLRMISDMLGPPSKEIWPGYENLPNAQVLSFSKQPYNRLPTKIPGLSAQGLKLLNGLLTYDPKKRMTAEQALRHPYF

>SARC_08063.1

YRKLGLVGKGTFGEVWKSKDLDPSKFVVLKKILEETEVEGFPITALREIMLMQRLEHKNILHAKRVVYNKSTFYMVSSWMDHDLAGLLNRG-KAFSVAQIKCLFKQLLVGLDFLHVRKILHRDIKCANILVNSAGLLKIADFGLARTFDDKQNGLTNRVVTLWYRPPELLLGERMYDTKIDMWGAGCVLGEMANTAPLMKGNSEIEQLDCIFKLCGTPNADNWAGGTELEVFQHTQYPSMLWQELEKMEPGRMEPFVHIMEGLLCLDPRKRLSASDALDHIFF

>SARC_10569.1

YQKLEKLGEGTYAQVYRGVSTITGEQCALKEINLDA-EEGAPCTAIREASLLRELKHVNIVILHDIVHTHTSMTFVFEYLNMDLKDYIDRAAPYVALTNVRLLFYQLLRALECCARKRILHRDIKPQNILLNEIGELKLADFGLARAKGVPIKSFSHEVVTLWYRPLDILLGSVDYNSSLDIWSAGCILAELVTGTPLFPGKDHDSQLTTVFSKLGTPSPDTWPELENLPLYSK-IWTMYAPKPFTYLVPRLESEGIRLLSEMVVYEPAKRISAKEAMRHEYF

>SARC_06703.1

-----------------------GDVVALKRIRLDSEDEGVPCTAIREISLLKELRHPNIVRLYDVLHTDKKLTLVFEYCEQDLKKYLDGCGGTVEPFQVKSFLYQLLKGVAFCHEHRVLHRDLKPQNLLINKFGKLKLADFGLARAFGIPVRSYSHEVVTLWYRAPDVLLGSRRYSTSIDIWSAGCIFAEMVQGRPLFPGLAAPDQLLRIFKFLGTPTEEIWYGVTELPEWSKYEFPMYAAQPLSSAVNNLDMSGLDLLKSMLVYMPDFRISAEDALRHRYF

>Sce-kin28p-gi|6320095|

YTKEKKVGEGTYAVVYLGCQHSTGRKIAIKEIKTSEFKDGLDMSAIREVKYLQEMQHPNVIELIDIFMAYDNLNLVLEFLPTDLEVVIKDKSILFTPADIKAWMLMTLRGVYHCHRNFILHRDLKPNNLLFSPDGQIKVADFGLARAIPAPHEILTSNVVTRWYRAPELLFGAKHYTSAIDIWSVGVIFAELMLRIPYLPGQNDVDQMEVTFRALGTPTDRDWPEVSSFMTYNKLQYPPPSRDELRKRFIAASEYALDFMCGMLTMNPQKRWTAVQCLESDYF

>Sce-cdc28p-gi|6319636|

YKRLEKVGEGTYGVVYKALDLRPQRVVALKKIRLESEDEGVPSTAIREISLLKELKDDNIVRLYDIVHSDHKLYLVFEFLDLDLKRYMEGIPQPLGADIVKKFMMQLCKGIAYCHSHRILHRDLKPQNLLINKDGNLKLGDFGLARAFGVPLRAYTHEIVTLWYRAPEVLLGGKQYSTGVDTWSIGCIFAEMCNRKPIFSGDSEIDQIFKIFRVLGTPNEAIWPDIVYLPDFKP-SFPQWRRKDLSQVVPSLDPRGIDLLDKLLAYDPINRISARRAAIHPYF

>Sce-pho85p-gi|6325226|

FKQLEKLGNGTYATVYKGLNKTTGVYVALKEVKLDS-EEGTPSTAIREISLMKELKHENIVRLYDVIHTENKLTLVFEFMDNDLKKYMDSRTRGLELNLVKYFQWQLLQGLAFCHENKILHRDLKPQNLLINKRGQLKLGDFGLARAFGIPVNTFSSEVVTLWYRAPDVLMGSRTYSTSIDIWSCGCILAEMITGKPLFPGTNDEEQLKLIFDIMGTPNESLWPSVTKLPKYNP-NIQQRPPRDLRQVLQPLDGNLMDFLHGLLQLNPDMRLSAKQALHHPWF

>Sce-ctk1p-gi|6322710|

YLRIMQVGEGTYGKVYKAKNTNTEKLVALKKLRLQGEREGFPITSIREIKLLQSFDHPNVSTIKEIMVEQKTVYMIFEYADNDLSGLLLNKEVQISHSQCKHLFKQLLLGMEYLHDNKILHRDVKGSNILIDNQGNLKITDFGLARKMNSR-ADYTNRVITLWYRPPELLLGTTNYGTEVDMWGCGCLLVELFNKTAIFQGSNELEQIESIFKIMGTPTINSWPTLYDMPWFFMI-MPQQTTNNFSEKFKSPSSKCLQLAINLLCYDQTKRFSATEALQSDYF

>Sce-sgv1p-gi|6325419|

YREDEKLGQGTFGEVYKGIHLETQRQVAMKKIIVSVEKDLFPITAQREITILKRLNHKNIIKLIEMVYDHKSFYMILPYMVADLSGVLHNPRINLEMCDIKNMMLQILEGLNYIHCAKFMHRDIKTANILIDHNGVLKLADFGLARLYYCPPAKYTSVVVTRWYRAPELVLGDKQYTTAVDIWGVGCVFAEFFEKKPILQGKTDIDQGHVIFKLLGTPTEEDWAVARYLPGAEL-TTTNYK-PTLRERFKYLSETGLDFLGQLLALDPYKRLTAMSAKHHPWF

>Sce-ssn3p-gi|6325215|

YEVIGYIAAGTYGKVYKAKRQINTVFYAIKKFKTEKDYTGISQSACREMALCRELHNKHLTTLVEIFLERKCVHMVYEYAEHDLLQIIHFHKRMIPPRMVRSIMWQLLDGVSYLHQNWVLHRDLKPANIMVTIDGCVKIGDLGLARKFHNMLQTLDKVVVTIWYRAPELLLGARHYTPAVDLWSVGCIFAELIGLQPIFKGEEAVNQLQRILEVLGTPDQKIWPYLEKYPEYDQITFPKYRD-NLATSAGGRDKHALSLLYHLLNYDPIKRIDAFNALEHKYF

>Spo-srb10-gi|19114301|

YKIIGFISSGTYGKVYKAVSSNSKRLFAIKKFKAESKQTGVSQSAIREMMLCREIQHENIVSLVQVLLKDGTISMVFEYAEHDLLQIIHFHTRQIPPSILKSILWQIINGVAYLHENWIMHRDLKPANIMITATGKVKIGDLGLGRLIRDPILPFDRVVVTIWYRAPELLLGAHDYTPAIDVWAIGCIYGEMLALSPLFKGDEISTQMLRIMELLGTPTEERWPGLKNYPEYYQLSFEVYWN-NLPQTVKNRDPQGLDLLMKMLQYDPKSRITAKQALEHVFF

>Spo-ppk23-gi|19112531|

YEILEKIEEGSYGIVYRGLDKSTNTLVALKKIKFDPNGIGFPITSLREIESLSSIRHDNIVELEKVVVGLKDVYLVMEFMEHDLKTLLDNMPEDFLQSEVKTLMLQLLAATAFMHHHWYLHRDLKPSNLLMNNTGEIKLADFGLARPVSEPKSSLTRLVVTLWYRAPELLLGAPSYGKEIDMWSIGCIFAEMITRTPLFSGKSELDQLYKIFNLLGYPTREEWPQYFLLPYANKIKHPTVPHSKIRTSIPNLTGNAYDLLNRLLSLNPAKRISAKEALEHPYF

>Spo-cdc2-gi|19112421|

YQKVEKIGEGTYGVVYKARHKLSGRIVAMKKIRLEDESEGVPSTAIREISLLKEVNDSNCVRLLDILHAESKLYLVFEFLDMDLKKYMDRISTSLDPRLVQKFTYQLVNGVNFCHSRRIIHRDLKPQNLLIDKEGNLKLADFGLARSFGVPLRNYTHEIVTLWYRAPEVLLGSRHYSTGVDIWSVGCIFAEMIRRSPLFPGDSEIDEIFKIFQVLGTPNEEVWPGVTLLQDYKS-TFPRWKRMDLHKVVPNGEEDAIELLSAMLVYDPAHRISAKRALQQNYL

>Spo-pef1-gi|19075421|

YQRLEKLGEGTYAHVYKGQNRVTGEIVALKVIRIDA-DEGTPSTAIREISLMKELRHPNIMSLSDVLQTENKLMLVFEYMEKDLKKYMDTYGGALPPSQVKNFTQQLLKGISFCHENRVLHRDLKPQNLLINSRGELKLADFGLARSIGIPVNTFSNEVVTLWYRAPDVLLGSRVYSTSIDIWSVGCIMAEMATGRPLFAGSNNEDQLLKIFRLLGTPTEQSWPGISLLPEYKP-TFPIYKAQDLAYLFPTFDPLGLDLLRRMLRLQPELRTTGQDALQHAWF

>Spo-cdk9-gi|19112408|

YHLMEKLGEGTFGEVYKSQRRKDGKVYALKRILMHTEKEGFPITAIREIKILKSIKHENIIPLSDMTVVRGSIYMVTPYMDHDLSGLLENPSVKFTEPQIKCYMKQLFAGTKYLHDQLILHRDLKAANLLIDNHGILKIADFGLARVITEERREYTGCVVTRWYRSPELLLGERRYTTAIDMWSVGCIMAEMYKGRPILQGSSDLDQLDKIFRLCGSPTQATMPNWEKLPGCEGVRFPSH-PRTLETAFFTFGKEMTSLCGAILTLNPDERLSASMALEHEYF

>Spo-lsk1-gi|19115305|

YEKIDQIGEGTYGKVYKAINTVTGDLVALKRIRLEQEKDGFPITTVREVKILQRLRHKNIVRLLEIMVEKSSVYMVFEYMDHDLTGVLLNSQLHFTPGNIKHLSKQIFEALAYLHHRGVLHRDIKGSNILLNNNGDLKFADFGLARFNTSSSANYTNRVITLWFRPPELLLGETAYDTAVDIWSAGCIVMELFTGKPFFQGRDEISQLEVIYDMMGTPDVHSWPEVKNLPWYELLKPVEEKKSRFVETFEILSPAAIDLCQKLLALNPFCRPSAHETLMHEYF

>Spo-mcs6-gi|19113141|

YVKERKVGEGTYAVVFLGRQKETNRRVAIKKIKVGQFKDGIDISALREIKFLRESRHDNVIELVDVFSTKSNLNIILEFLDSDLEMLIKDKFIVFQPAHIKSWMVMLLRGLHHIHSRFILHRDLKPNNLLISSDGVLKLADFGLSRDFGTPS-HMSHQVITRWYRPPELFMGCRSYGTGVDMWSVGCIFAELMLRTPYLPGESDLDQLNVIFRALGTPEPEVIKSMQQLPNYVE--MKHIPNGGMEALFSAAGHEEIDLLKMMLDYNPYRRPTAQQALEHHYF

>Cci-gi|116507718|

WLKDRKVGEGAYAVVYQGREASTGRKVAIKKIKVGQFKDGLDMSAIREVKFLRELKHQNVIELLDVFSSKKNLNLVLEFLDTDLEIIIRDRSLVFLPADIKSWMAMTFRGLEFCHRNWILHRDLKPNNLLIASDGQLKIADFGLARDFADPGYKMTCQVITRWYRPPELLYGCRYYGTGVDIWSVGCIFAELMLRIPYLAGESDMDQLKTIFRALGTPTEEEWPGHTKLPDYVP--VGQFPKTPLRDLFTAASADALNLLSKCLVYEPRKRISAREALNHPYF

>Cci-gi|299740157|

YEVTTKLGEGTFGEVHKAIQKATGASVALKRILMHHEKEGMPVTALREIKILKALKHPCIVNILDMFVVPLSVYMVFPYMDHDLAGLLENERVKLQPSQIKLYMKQLLEGTEYMHRNHILHRDMKAANLLISNTGNLRIADFGLARSFDTSTRKYTNCVVTRWYRPPELLLGARQYGGEVDIWGIGCVLGEMFNRRPILPGSSDLDQLEKIWYLCGTPTQHSWPNFDALPGCDGVKFKSNHIRRVKMTYESVGAETADLLDKLLVCNPKERITAAQALEHEYF

>Cci-gi|299755918|

YIQLEKLGEGTYATVYKGRSRTTNEIVALKEIHLDA-EEGTPSTAIREISLMKELKHVNIVRLHDVIHTETKLVLIFEYCEQDLKKYMDQHGGALEPEVVRSFMYQLLKGTAFCHENQVLHRDLKPQNLLINRKGELKLGDFGLARAFGVPVNTFSNEVVTLWYRAPDVLLGSRTYSTSIDVWSCGCIFAEMISGVPLFRGRDNQDQLLHIMRIIGTPSPAQFAKICKTPEIQPKQFPNYPRLPFHQVLPKASPQALDLLDKLLKFDPAERISAADALAHPYF

>Cci-gi|169865137|

YAILGFISSGTYGRVYKAQSINGGELLAIKKFKPDKEYTGISQSAIREIALNREINHENVVALREVILEDKSIYMVFEYAEHDFLQVIHHYRTSIPTAVLKSLIYQLFNGLIYLHASHILHRDLKPANILITSQGVVKIGDLGLARLCYEPLQPLDKVVVTIWYRAPELLMGAKHYNKAIDCWAVGCVMAELASLRPIFKGEEARDQLIKIFEVLGTPDERDWPGVVDMPEYRNMKLDHFSN-RLSDT-RIRSPQGYDLLRQLFAYDPDNRLTAEQAIQHKWF

>Cci-gi|299755758|

YERLNQIEEGSYGVVFRARDRQTGDIVALKKLKLDEEKNGFPITALREIYALMTCQHENVVRIREVVLPP--------PLSTDAPSALHLASIRSLDEEFKTLMMQLLSAVHHCHQNWILHRDLKTSNLLMNNRGTIKVADFGLARRYGDPVGGLTQLVVTLWYRAPEILLGATEYSTAVDMWSVGCIFAELLLKEPLFQAKGELELISMIFKLLGPPTKNSWPEYFDLPMAKTIALPSPQPHQFRSKFPYLTTNGLDLLMCLLTYDPERRITAEEALQHPYF

>CCi-gi|299748916|

-------MAGTYGVVYKARDTSTNTIVALKKIRLEAEDEGVPSTAIREISLLKELKDDNIVKLLDIVHADQKLYLVFEFLDVDLKRFIETGNSPITPALVKKFTHQLNSGLLYCHSHRILHRDLKPQNLLIDKHNNLKLADFGLARAFGIPMRTYTHEVVTLWYRAPEVLLGSRHYSTAIDMWSVGCIFAEMAMGAPLFPGDSEIDQIFKIFRLLGTPNEDIWPGVSTLPDYKP-TFPNWSRQDLAKAVPTLDEAGIDMLKRTLTYDSAKRLSAKRALVHPYF

>Cci-gi|299747693|

YVILNQVGEGTFGKVYKARNTVAKVHVALKRIRMETERDGFPVTAMREIKLLQSLKHPNVVQLYEMMVSNGSVFMVFEYMDHDLTGILSQTQFKFSDSHLKSLCHQMLAGLAYLHHKGVIHRDIKGSNILLNNRGELKLADFGLARFYQKRRRDYTNRVITLWYRPPELLFGATVYGPEVDMWSAGCIMLELFTKKPVFQGNDEINQLHVIFKILGTPTTERWTGLNNLPWFELIKPKESLPNRFRDLFQWMSPAALDLAERLLTYDPELRVSAQEAMEAPYF

>SPPG_01972.2

TNKKLQIGEGAHGVVLKAKSIETGLVVALKKVPLRRLEDGIPNTILREIKALQEIDYQNVVKLLSVHPSGPSFVLVFEYMLSDLSHILRTT--PLTSSQVKAYMLMLLRGLSYCHENNIMHRDLKPANLLISPTGVLKLADFGLARVHRYPKRPYSHQVATRWYRAPELLYGARMYDAGVDLWAVGCIFGELLNHSPLFPGQNDIDQLYCVLSILGTPTKESWPDLETLPDYNKIQFPNMPAVALERVCPDASQEAVGLLKKFLVYSCKNRIPAKEALLEPYF

>SPPG_03982.2

FIKDKKVGEGTYATVYLGWA------VPIRKEKVAE-RLAHDHTGERERNIYNRRFYAYSLQLIDVFSHKTNLNLVLEYLDADLEMVIKNKTVVFSAADVKSWMLMTLRGLYHCHRNFILHRDLKPNNLLLASDGQLKLADFGMARDYGDPHKKMTSVVVTRWYRAPELLLGATRYGYAIDMWAVGCIFAELMLRTPFMAGDSDIGQLQIIFKALGTPTEQDWPVGAILQGMKELEFQQYPKPPLRSILTAASPDALNLLEQFLLFDPLKRITAEDALKHFYF

>SPPG_06236.2

YIRLEKLGEGTYATVYKGKNRNDGSIVALKEIHLDN-EEGAPSTAIREISLMKELRHPNIVRLHDVIHTEKTLTLVFEYMDQDLKKYMDSLGGMIQPHMAKWFMYQLLKGIAFCHDNRVLHRDLKPQNLLINSKGELKLGDFGLARAFGIPVNTFSNEVVTLWYRAPDVLLGSRNYSTSIDIWSAGCIMAEMYSGKPLFPGKTNEDQLYKIFKLLGTPTDEIWPRVSELPEYKP-NWPIYQGQVLSSKIPNMDWMGYDLLSKMLVYEPTRRISAKDALNHVYF

>SPPG_05640.2

YEKLNRVGEGTYGIVYRARDKASGTIVALKRIRMEQEQEGLPLSSLREISLLKSLRHENVVSVLDVVVGNGLEKLKVNSAGQCIPSVLGW-LLILSCITVKCLMSQLLTGLAYLHDNYIIHRDLKLSNLLLTSSGILKIADFGLARKFGTPVRPMTPKVVTLWYRAPELLFGEKSYTTAIDMWAVGCIFGELIRAKPLLPGKVEQQQLDLICRLLGTPNARIWPGFDKLPYAKSVKLPSIPYDDIAGQFSEQKESVRKLLKSLLTYYPSSRLTVHEALRHDYF

>SPPG_00051.2

YDNRERIGKGSFGEVTIATHKETQRKVALKRIIVHKEKDGLPITAVREINILKGLRHKSVIELLGMAVAPATIYMIFPYMHHDLVGLLENRYVTLEPNQIKSFTKQLLEGVAYLHRNHFLHRDLKSSNILIDNEGTLKIADFGLARSYNAADKELTPNVITLWYRPPELLLGHKTYTSAVDMWGVGCIFAEMWDRKAIFKGETELDLLDKILRVCGTPDAKAWPEFTKLTASASI-RPKQESRKILDIYPRLDFQTIDLLNTFLVLNPARRPTAEAALKHEYF

>SPPG_00975.2

FESSKKVGEGTFGEVTIAKHKSTGRKVALKKIILHKDRDGLPITAVREIAILKSLRHPNLINLEEIAVRVATLFMVFPYMKHDLSGLLDNPQVTFDPSHIKSFTKQMLDGILYLHQRNILHRDMKSANILIDNGGNLRIADFGLARSFDPSGRRLTPTVVTLWYRAPELLLGKQDYTAAVDMWGIGCIFAEMWDRRPIFKGATEVELIEKIFSICGTPDPADYRSSNGFPKLEDGTTPQPQPRRIWDAYERLDFQTIAFIDYLLNLDPDRRPTADEALRHDYF

>SPPG_00440.2

YQKIEKLGEGTYGIVYKAQNKDTGDIVALKRIRLDNEEEGVPCTAIREISLLKELKHSNIVRLYDVIHTEKKLTLVFEYLDSDLKKFLDAYGGDIDVPTLKHLLYQLLKGIAFCHEHRVLHRDLKPQNLLINKKLELKLADFGLARAFGIPVRSYSHEVCVRIFNVNRFMVGRMHHGRNLDKSFARCLVDAAWSGRPLFPGSSIKDQLLRIFKLLGTPDEKSWPKVKELPDYKP-DFPIYPRASLESISSKLDAHGLDLLSKLIEYQPEKRISAERALQHPYF

>SPPG_07418.2

YDKIEKVGEGTYGVVYKARDRHSGEIVALKKIRLETEDEGVPSTAIREISLLKELKHPNIVRLLDIVHNDAKLYLIFEFLDLDLKKYMDTQSNGLSAPLIKSYMYQLIKGIHYCHCHRILHRDLKPQNLLIDQQGMLKLADFGLARAFGVPLRTYTHEVVTLWYRAPEILLGSKHYSTAVDMWSVGCIFAEMCLRHPLFPGDSEIDEIFRIFRLLGTPNEEIWPNVTTLPDYKE-NFPIWTAQNLAKVLPNLESEGVDLLSRLLVYDPAQRLSAKRALSHPYF

>AMSG_07969.1

YDKLSEAGKGQYGVVYRARRKEDGKTVAIKRINQGDAREGVNFSAVREIRLLQELRHPHVVSLQEVFVHRGGIHLVMDFCETDLEAIVRDPSLRLGTPEIKAYMKMTLEGLAAMHDTWVLHRDLKPENLLVSATEGVKIADFGMARMYGSPNRRMTHQVATRWWRAPELLFGARAYGAGVDMWAMGCIFAELYMRAPLFQGETDLDQLSCIFAILGTVNNAVWPGVSELPDFVE--FEPSNGIALGKVVASAPADAVDLMVAMLAYNPASRISATEALQHPYF

>AMSG_03791.1

YMKLEKIGEGTYGVVYKARDLLTNDFIALKKIRLDAEDEGVPSTAIREISLLKELEHPNIVRLHDVVHSDKKLYLVFEYLDQDLKKYMDSVSGLLKPALVKSYLQQLLEGIAFCHSHRVLHRDLKPQNLLIDRNGVLKLADFGLARAFGIPVRTYTHEVVTLWYRAPEILLGSRHYSCPVDVWSIGCIFAEMASKIPLFPGDSEIDNLFRIFKILGTPNETIWPGVSALPDFKS-SFPKWQPKNLASVATNLGPDGIDLLSQMLEYEPSRRISAKAALSHPYF

>AMSG_11394.1

FQKERHVGEGTFGMVYLATDKATGERVALKKVRTESERQGFPITAIREIKILKGLNHPNVVSLKEVVMSDGTVYLVFEYASSDLVGILDNPEIALTLAQIKTYLKQTLEGLYYVHAQKIMHRDIKAANLLVH-DGVLKLADFGLAKPIDRNADGFTNQVVTLWYRAPEILLGERMYGLPIDIWSVGCIMVELLTGRAFLPGKDEAHQVDLICRQLGTPTEATWPGCTRLPRFAT--LVKKYPPMIARTLKDLQPDAYDLVTKLLSLDPAKRPTAQEALEHKFF

>AMSG_02688.1

YTRIAKLGEGTYGVVYKAQNRQTGLIVALKRIRLDSEEQGVPCTAIREIALLKELKHPNVVELLDVLHSDKKLTLVFEYLDQDLKKYMDGIGGGIEPHIVKTYMYQLLKGLAFCHEHRVLHRDLKPQNLLTSSRHELKLADFGLARAFGLPVRSYSTEVVTLWYRAPDVLLGSSTYSTSIDLWSAGCIFAEMVNGSPLFPGTSVENQLERIFNVVPLPPEDSLGEGVELPVGVGACEPAVLATTLGEVVPGLSAEGVDLLGLLLVADPAGRITATDALTHPYF

>AMSG_04337.1

FGDVDQIGEGTYGQVYRATDKATGDVVALKKIRADRKREGFPITAIREIKILKHLKHPNIVNLKEVVISRGSVYMVFEYVDHDLSGLLESVNPKMEAGQVKDFMLQLLASVAYTHQMGILHRDIKAANLLVSKDGVLKLADFGLARQIDQSA-RYTNKVITLQYRPPELLLGGDSYGPAVDMWSIGCIFVELLTGRTLFRADKEKDLMMAIVKVCGTPTEAEWPGLSALPLFHSFNFPR-TRRRLRDSLPSIDAQAFDLLDKLFTYDPDKRISAAAAIAHPYF

>AMSG_09913.1

YVLEAEVGRGTYGKVYKAQMVRGSEIVALKEFNGADRVDGIPLSGIREMALLRELKHPSLVTLHDVVVESGSTFLVLEYVEHDLEKLLIHHKHPLMAETVRSMMWQLVSGIEYMHANWVMHRDMKPSNVLVYGFGHIKITDFGMARIFKTPAVALNPVVATLWYRAPELLLGARHYTPAIDVWALGVIFAELLFLQVLFKGKQISHQMEAIVNILGTPDPATWPGLADLAHANQLAFTRVES-RLDAPQLLLSSQACDLLKRMVAYNPDDRITCADALRHPYF

>AMSG_04682.1

YRKVSTIAEGQYGLVFKAVRKADAAPVALKKLKLGNVEEGFPVTALRELAALRSLESPHVVRLLEVVVGMDEVYMVLEHHPLDLKVILDAHGGAFTSAQAKLLVWQLFSGLAFLHASWLIHRDIKPANLLYSADGRLVIADFGLVRPFGDPPAVLTPTVVTLWYRAPELIFGEAAYNTAVDVWSAGAVTVELLTGEPLLRADSETAYVQKMCLLLGAPSDAVWPGFSLLPGGSKYVLPAQRDSYLDDRLPDTSLAGRRLISSMLTYDPAARLTASDVVSADWF

>Ddi-gi|7579907|

YNIEALIGEGTYGVVSRATVKATGQIVAIKKIRKIQTDDGINFSAIREIKILQELKHDNVVNLLDIFAHKSNVYLVFELMQWDLQEVIEDKSIILKPADIKSYMKMLLQGIEACHRNWVLHRDLKPNNLLMSINGDLKLADFGLARQYGSPNKVFSPQAVTIFYRAPELLFGAKSYGPSVDIWSIGCIFAELMLRTPYLPGTGEIDQLRKICSALGTPNESNWPGVTCLPNYIK--FTDHPATPFKQLFTAASDEAIDLISKMLLFNPSNRISAADALNHPYF

>Ddi-gi|66805759|

YSKIEKLGEGTYGIVYKAKNRETGEIVALKRIRLDSEDEGVPCTAIREISLLKELKHPNIVRLHDVIHTERKLTLVFEYLDQDLKKYLDECGGEISKPTIKSFMYQLLKGVAFCHDHRVLHRDLKPQNLLINRKGELKLADFGLARAFGIPVRTYSHEVVTLWYRAPDVLMGSRKYSTPIDIWSAGCIFAEMASGRPLFPGSGTSDQLFRIFKILGTPNEESWPSITELPEYKT-DFPVHPAHQLSSIVHGLDEKGLNLLSKMLQYDPNQRITAAAALKHPYF

>Ddi-gi|66823249|

YQKLEKLGEGTYGKVYKAKEKATGRMVALKKIRLE--DDGVPSTALREISLLKEVPHPNVVSLFDVLHCQNRLYLVFEYLDQDLKKYMDSV--ALCPQLIKSYLYQLLKGLAYSHGHRILHRDLKPQNLLIDRQGALKLADFGLARAVSIPVRVYTHEIVTLWYRAPEVLLGSKSYSVPVDMWSVGCIFGEMLNKKPLFSGDCEIDQIFRIFRVLGTPDDSIWPGVTKLPEYVS-TFPNWPGQPYNKIFPRCEPLALDLIAKMLQYEPSKRISAKEALLHPYF

>Ddi-gi|66828493|

YTFSYEIGSGTYGMVYKADDKKRNNKVAVKKFRSTKEGEGLSLTAYREIGLLKELSNENIVKLLDVCLNDKLLYLIFDYAEFDLFGIIKYHGSHFSDATIKSLIWQVLNGIHYLHSNWVIHRDLKPSNILVMGCGTVKIGDFGLARIFQSPLKPLNGVVVTIWYRSPELLLGSKHYTRAVDIWAIGCIFAELITTKPLFPGKEKDDQVEKIIRVLGKPTLDMWPDIKHLPEWKRLSME-FPN-SLA-VGIDENSQAYDLLSKMILYDPSKRITASEALDHPYF

>Ddi-gi|66827511|

FEKLDSIGEGTYGIVSKGRDKETGRIVALKKVKIGQDKDGIPLTSLREIQILKEIKHPNIVSLLEVVIGGDKIYLVFEYLEHDVASLIDNINKPFKLSEIKCFLLQLLRAVEYLHSHWIIHRDLKCSNLLYGNNGNLKLADFGLARKFGYPIESITPCMVTLWYRSPELLLGCQKYSTAVDLWSIGSIFGELLIGRPLITGNNEVDQIMRIFNLLGEPNEQIWPGFSSLPNFKRLNIPHQPYNNLRELVPTISDTAFDLLNQLLTYDPTKRITASDAIKHPFF

>Ddi-gi|66822245|

HEKIEQIGEGTFGQVYKAKNKSNGDIVALKKVIMDNEVEGFPITAIREIKILKELNHANVVNLKEVVTSKGSVYMVFEYMDHDLNGLMDSPAKYFAPGQIKCYLKQLLEGLDYCHRNNVLHRDIKGSNLLLDNNGILKLADFGLARPFNSSEQILTNRVITLWYRPPELLLGTFHYGPEIDMWSVGCIMAELLSKKTLFPGRNSIDQLDKIYQVCGSPNANNWPEAMDLPFWDAL-KPKYNSLSLKDFYSFFTKEAFDLLDKLLCMDPKKRITASEALDHQYF

>Ddi-gi|66810856|

FKKLYTINEGAFGVVYCAQDKETEEIVALKKIKMEREREGIPITSVREIKVLMELKHDNIVQIKEIVLGINSIFMAMEFIDHDLRGLMEVIKKPFLPSEIKTLIQQLLNGVSYMHDNWVIHRDLKTANLLYTNKGVLKIADFGLAREYGSPLKPLSKGVVTLWYRAPELLLDTEIYTPAIDIWSVGCIFAEIISKEVLLQGSSEIDQMDKIFKLFGTPTEKSWPAFFKLPLAKYFNLTDQPYNNLKSKFPHITDNAFDLLNKLLELNPEARISASDALKHPYF

>Ddi-gi|66816795|

YEIISKIGEGISGSVFKAIKKGTEEMVALKNFKGWTEGD---RASKEECSLLQQLRHIYITPVIDIYTNTSEYIIVFPYFEHDLSGLLSEHR--LSIPQVKCYFKQLLEGINEIHNAGVMHRDIKAANLLVNNKGSLFIGDLGTATSYTK-RSVFSSKVVTLWYRAPELLLGSTQYGPEIDMWSIGCVLIELVTSRNFLPGSSEQQQLEAICKLCGTPTDEIWPNVSQLQNFNQISLPVY-PSRLRTVFKNFSNDFIELLEGLLTLNPKKRLTAEQALQSPFF

1. **Multiple alignments for phylogenetic analysis of cyclin family proteins in *H. sapiens*, *N. vectensis*, *T. adhaerens*, *A. queenslandica*, *M. brevicollis*, and *S. rosetta*.**

>Hsa-CyclinA1

----EIYQYLREAEIRHRPKAHYMK-KQPDITEGMRTILVDWLVEVGEEYKLRAETLYLAVNFLDRFLSCMSVLRGKLQLVGTAAMLLASKYEEIYPPEVDEFVYITDDTYTKRQLLKMEHLLLKVLAFDLTVPTTNQFLLQYLRRQGVCVRTENLAKYVAELSLLADPFLKYLPSLIAAAAFCLANYTVNKHFWPETLAAFTGYSLSEIVPCLSELKAYLDIPHRPQQAIREKY

>Hsa-CyclinA2

----DIHTYLREMEVKCKPKVGYMK-KQPDITNSMRAILVDWLVEVGEEYKLQNETLHLAVNYIDRFLSSMSVLRGKLQLVGTAAMLLASKFEEIYPPEVAEFVYITDDTYTKKQVLRMEHLVLKVLTFDLAAPTVNQFLTQYFHQQPANCKVESLAMFLGELSLIADPYLKYLPSVIAGAAFHLALYTVTGQSWPESLIRKTGYTLESLKPCLMDLQTYLKAPQHAQQSIREKY

>Hsa-CyclinB2

----DIYQYLRQLEVLQSINPHFLD--GRDINGRMRAILVDWLVQVHSKFRLLQETLYMCVGIMDRFLQVQPVSRKKLQLVGITALLLASKYEEMFSPNIEDFVYITDNAYTSSQIREMETLILKELKFELGRPLPLHFLRRASKAGEVDVEQHTLAKYLMELTLIDYDMVHYHPSKVAAAASCLSQKVLGQGKWNLKQQYYTGYTENEVLEVMQHMKVKVNENLTKFIAIKNKY

>Hsa-CyclinB1

----DIYAYLRQLEEEQAVRPKYL--LGREVTGNMRAILIDWLVQVQMKFRLLQETMYMTVSIIDRFMQNNCVPKKMLQLVGVTAMFIASKYEEMYPPEIGDFAFVTDNTYTKHQIRQMEMKILRALNFGLGRPLPLHFLRRASKIGEVDVEQHTLAKYLMELTMLDYDMVHFPPSQIAAGAFCLALKILDNGEWTPTLQHYLSYTEESLLPVMQHLKVMVNQGLTKHMTVKNKY

>Hsa-CyclinD3

----RVLQSLLRLEERYVPRASYFQCVQREIKPHMRKMLAYWMLEVCEEQRCEEEVFPLAMNYLDRYLSCVPTRKAQLQLLGAVCMLLASKLRETTPLTIEKLCIYTDHAVSPRQLRDWEVLVLGKLKWDLAAVIAHDFLAFILHRLSLPRDRKKHAQTFLALCATDYTFAMYPPSMIATGSIGAAVQGLG-CSMTELLAGITGTEVDCLRACQEQI------------------

>Hsa-CyclinB3

----EIFSYMKEREEQF-ILTDYMN-RQIEITSDMRAILVDWLVEVQVSFEMTHETLYLAVKLVDLYLMKAVCKKDKLQLLGATAFMIAAKFEEHNSPRVDDFVYICDDNYQRSEVLSMEINILNVLKCDINIPIAYHFLRRYARCIHTNMKTLTLSRYICEMTLQEYHYVQEKASKLAAASLLLALYMKKLGYWVPFLEHYSGYSISELHPLVRQLKLLTFSSYDSLKAVYYKY

>Hsa-CyclinD2

----RVLQNLLTIEERYLPQCSYFKCVQKDIQPYMRRMVATWMLEVCEEQKCEEEVFPLAMNYLDRFLAGVPTPKSHLQLLGAVCMFLASKLKETSPLTAEKLCIYTDNSIKPQELLEWELVVLGKLKWNLAAVTPHDFIEHILRKLPQQREKRKHAQTFIALCATDFKFAMYPPSMIATGSVGAAICGLQQSSLTELLAKITNTDVDCLKACQE--------------------

>Hsa-CyclinE1

----EVWKIMLNKEKTYLRDQHFLE-QHPLLQPKMRAILLDWLMEVCEVYKLHRETFYLAQDFFDRYMATQNVVKTLLQLIGISSLFIAAKLEEIYPPKLHQFAYVTDGACSGDEILTMELMIMKALKWRLSPLTIVSWLNVYMQVAY----NVLLIAELLDLCVLDVDCLEFPYGILAASALYH-F-SS-----SELMQKVSGYQWCDIENCVKWMGKLKHFRGVADEDAHNIQ

>Hsa-CyclinE2

----EVWLNMLKKESRYVHDKHFEV-LHSDLEPQMRSILLDWLLEVCEVYTLHRETFYLAQDFFDRFMLTQDINKNMLQLIGITSLFIASKLEEIYAPKLQEFAYVTDGACSEEDILRMELIILKALKWELCPVTIISWLNLFLQVDA----KVLLIAQLLDLCILAIDSLEFQYRILTAAALC-HF-TS-----IEVVKKASGLEWDSISECVDWMKSPVKLKTFKKIPMEDRH

>Hsa-CyclinK

----HTKPCWYWDKKDLAHTPSQLEGLDPATEARYRREGARFIFDVGTRLGLHYDTLATGIIYFHRFYMFHSFKQFPRYVTGACCLFLAGKVEETP-KKCKDIIKTARSDDPKEEVMVLERILLQTIKFDLQVEHPYQFLLKYAKQLKGDKNLVQMAWTFVNDSLCTTLSLQWEPEIIAVAVMYLAGRLCKFRRWWEQFVD---VPVDVLEDCHQDYQQSSEPSQPQQKDPQQPA

>Hsa-CyclinJ

----DIHQALRYKELK---LPSYKG-QS--PQLSLRRYFADLIAIVSNRFTLCPSARHLAVYLLDLFMDRYDISIQQLHLVALSCLLLASKFEEKEVPKLEQLNSLTNLVLTKQNLLHMELLLLETFQWNLCLPTAAHFIEYYLSEAVHDLHMAKYADYFLEVSLQDYAFLNYAPSLVAAACVASSRIILRLPTWPTRLHRLTAYSWDFLVQCIERL------------------

>Hsa-CyclinF

----SEIVCQLFQASQAVSKQQVFS-VQKGLNDTMRYILIDWLVEVATMKDFTSLCLHLTVECVDRYLRRRLVPRYRLQLLGIACMVICTRFISKEILTIREAVWLTDNTYKYEDLVRMMGEIVSALEGKIRVPTVVDYKEVLLTLVPVELRTQHLCSFLCELSLLHTSLSAYAPARLAAAALLLARLTHGQQPWTTQLWDLTGFSYEDLIPCVLSLY-----RQVSLTAVKQRF

>Hsa-CyclinO

----SCYAFRKAQE-SHFHPREALA-RQPQVTAESRCKLLSWLIPVHRQFGLSFESLCLTVNTLDRFLTTTPVAADCFQLLGVTSLLIACKQVEVHPPRVKQLLALCCGAFSRQQLCNLECIVLHKLHFTLGAPTISFFLEHFTHA-RVALEAQALARGVAELSLADYAFTSYSPSLLAICCLALADRMLRVS--RPVDLRLGDHPEAALEDCMGKL------------------

>Hsa-CyclinD1

----RVLRAMLKAEETCAPSVSYFKCVQKEVLPSMRKIVATWMLEVCEEQKCEEEVFPLAMNYLDRFLSLEPVKKSRLQLLGATCMFVASKMKETIPLTAEKLCIYTDNSIRPEELLQMELLLVNKLKWNLAAMTPHDFIEHFLSKMPEAEENRKHAQTFVALCATDVKFISNPPSMVAAGSVVAAVQGLNLNFLTRFLSRVIKCDPDCLRACAL--------------------

>Hsa-CyclinT2

----GASSRWFFTREQLENTPSRRCGVEADKELSCRQQAANLIQEMGQRLNVSQLTINTAIVYMHRFYMHHSFTKFNKNIISSTALFLAAKVEEQA-RKLEHVIKVAHALQQTQELVILETIMLQTLGFEITIEHPHTDVVKCTQLVRASK-LAQTSYFMATNSLLTTFCLQYKPTVIACVCIHLACKWSNWKHWWEYVDT---VTLELLDETHEQ-------------------

>Hsa-CyclinL2

----ITLENCLLPDDKLRFTPSMSSGLDTDTETDLRVVGCELIQAAGILLRLPQVAMATGQVLFQRFFYTKSFVKHSMEHVSMACVHLASKIEEAP-RRIRDVINVFHRVNLKNQIIKAERRVLKELGFCVHVKHPHKIIVMYLQVLECERNLVQTSWNYMNDSLRTDVFVRFQPESIACACIYLAARTLEIPHWFLLFG----ATEEEIQECLKQY------------------

>Hsa-CyclinG2

----ERFQPREKGLSL---IEATPE-NDNTLCPGLRNAKVEDLRSLANFFGSCTETFVLAVNILDRFLALMKVKPKHLSCIGVCSFLLAARIVEEDIPSTHDVIRISQCKCTASDIKRMEKIISEKLHYELEATTALNFLHLYHTII-----LDKLEAQ-LKACNCRLIFSKAKPSVLALCLLNLEVE--------D--------------------------------------

>Hsa-CyclinT1

----NNNKRWYFTREQLENSPSRRFGVDPDKELSYRQQAANLLQDMGQRLNVSQLTINTAIVYMHRFYMIQSFTQFPGNSVAPAALFLAAKVEEQP-KKLEHVIKVAHTLQQVQDLVILESIILQTLGFELTIDHPHTHVVKCTQLVRASK-LAQTSYFMATNSLLTTFSLQYTPPVVACVCIHLACKWSNWKHWWEYVDT---VTLELLDETHEQ-------------------

>Hsa-CyclinL1

----LTIDHSLIPEERLSPTPSMQDGLDLPSETDLRILGCELIQAAGILLRLPQVAMATGQVLFHRFFYSKSFVKHSFEIVAMACINLASKIEEAP-RRIRDVINVFHHINTKNQVIKAERRVLKELGFCVHVKHPHKIIVMYLQVLECERNLVQTAWNYMNDSLRTNVFVRFQPETIACACIYLAARALQIPHWFLLFG----TTEEEIQECIERY------------------

>Hsa-CABLES1

----KHKRVLIFPSYMTTVIDYVKPSDLKMNETFKEKLKREMRKLAQEDCGLEEPTVAMAFVYFEKLALKGKLNKQNRKLCAGACVLLAAKIGSDLKHEVKHLIDKLEEKLNRRELIAFEFPVLVALEFALHLPEH-EVMPHY--------------------------------------------------------------------------------------------

>Hsa-CyclinC

----HYLQWILDKQDLLKERQKDLKFLSEEEYWKLQIFFTNVIQALGEHLKLRQQVIATATVYFKRFYARYSLKSIDPVLMAPTCVFLASKVEEFG-VVNTRLIAAATSPYRMNHILECEFYLLELMDCCLIVYHPYRPLLQYVQDMGQE--LLPLAWRIVNDTYRTDLCLLYPPFMIALACLHVACVVQQKRQWFAELS----VDMEKILEIRVK-------------------

>Hsa-CyclinG1

----SRCQPKVCGLRL---IESAHD-NGLRMTARLRDFEVKDLLSLTQFFGFDTETFSLAVNLLDRFLSKMKVQPKHLGCVGLSCFYLAVKSIEEEVPLATDLIRISQYRFTVSDLMRMEKIVLEKVCWKVKATTAFQFLQLYYSLLQ----FERLEAQ-LKACHCRIIFSKAKPSVLALSIIALEIQ--------E--------------------------------------

>Hsa-CyclinH

----ANRKFRCKAVANGKVLPNDPVFLEPHEEMTLCKYYEKRLLEFCSVFAMPRSVVGTACMYFKRFYLNNSVMEYHPRIIMLTCAFLACKVDEFN-VSSPQFVGNLREEKALEQILEYELLLIQQLNFHLIVHNPYRPFEGFLIDLKTILELRKTADDFLNRIALTDAYLLYTPSQIALTAILSSASRAGI-------------------------------------------

>Hsa-CyclinI

----KAITREAQMWKV---NVRKMP-SNQNVSPSQRDEVIQWLAKLKYQFNLYPETFALASSLLDRFLATVKAHPKYLSCIAISCFFLAAKTVEEDIPVLKVLARDSFCGCSSSEILRMERIILDKLNWDLHTATPLDFLHIFHAIAVSTRPLAVLTKQ-LLHCMACNQLLQFRGSMLALAMVSLEMEKL--PDWLF--------------------------------------

>Hsa-Fam58A

------------PEGGGGGPAARGPEGQPAPEARVHFRVARFIMEAGVKLGMRSIPIATACTIYHKFFCETNLDAYDPYLIAMSSIYLAGKVEEQH-LRTRDIINVSNRWELRDSIVQCELLMLRVLRFQVSFQHPHKYLLHYLVSLQNHSWVAVTAWALLRDSYHGALCLRFQAQHIAVAVLYLALQVYGVKPWWQVFND---LTKPIIDNVSDQY------------------

>Hsa-CABLES2

----GKHKRVLIFASYMTTVIEYVKPSDLDMNETFRESLKREMRSLSEECSLEPVTVAMAYVYFEKLVLQGKLSKQNRKLCAGACVLLAAKISSDLKSGVTQLIDKLEERFNRRDLIGFEFTVLVALELALYLPEN-QVLPHY--------------------------------------------------------------------------------------------

>Hsa-Fam58B

--------MEDAGEEAGEDAGEDAREGAAAPAARVHFRVARFIMEAGVKLGMQSIPIATACTIYPKFFCETILDAFDPYLIAMSSIYLAGKVEEQP-LWAHDIISVSNRWELRDSIVQRELLMLRVLRFQVSFQHPHKYLLYYLVSLKNHSWVAVTAWALLRDSYHGGLCLRFQAQHIAVVVLYLALQVYGVKLWWQAFSD---LTKPIIDTVSDQY------------------

>Hsa-CyclinI2

----CHLQLAQDREAR---LWRGGK-PQDEICDAFE-EVVLWLLRLQNTFYFSQSTFNLALTIFGRLLISVKVKEKYLHCATITSLRLAAKVNEEEIPQVKDFTKHYGSDYSPNELLRMELAILDRLHWDLYIGTPLDFLTIFHALVVLSWPVASLTRQ-LQHCMAGHQLLQFKGSTLALVIITLELERL--PGWCA--------------------------------------

>Hsa-CyclinJL

----DVHCTLREKELK---LPTFRA-HS--PLLKSRRFFVDILTLLSSHCQLCPAARHLAVYLLDHFMDRYNVTSKQLYTVAVSCLLLANGVEDREVPKLEQILSSQNFTLTKKELLSTELLLLEAFSWNLCLPTPAHFLDYYLLASVSHHCLKEYAHYFLEVTLQDHIFYKFQPSVVAAACVGASRICLQLPYWTRDLQRISSYSLEHLSTCIEIL------------------

>Hsa-CyclinYL

----FLSKSQTDVREKRKSNHLNHCDLSNPHKEQREKFIYRFVRTLFSAAQLTAECAIVTLVYLERLLTYAEICPTNWKRIVLGAILLASKVWDDQAVWNVDYCQILKD-ITVEDMNEMERHFLELLQFNINVPASVY-AKYYF------------DL--RSLA--DDNNLNFAP------------------------------------------------------------

>Hsa-CyclinY

----CVALAIYYHIKNRDPDGRMLLDIFDLHPLSKSEQIYRFVRTLFSAAQLTAECAIVTLVYLERLLTYAEICPANWKRIVLGAILLASKVWDDQAVWNVDYCQILKD-ITVEDMNELERQFLELLQFNINVPSSVY-AKYYF------------DLRSLAEA--NNLSFPLLS------------------------------------------------------------

>Tad-gi|196003740|

----IIFENMKQREAQL-VVNDYLE-RQNDITEQMRMILIDWLCEVQQNFELFHETLYLAVKIVDRFLSARVVSRDALQLIGATAMLMSSKIEERYPPLVDDFVYICDDAYSRQAVLDMERDICYALDFDLNIPIPYRFLRRYGKVASLSMENLTLARYILELTLQEYQFVTFKPSMLAAGCLCLALKMKNCGEWTQTLVHYSGYEESELNELVQKLAMIAKPAPENCKVVKTKY

>Tad-gi|196005765|

----DIHSYLRKAEYFHRPKYDYME-RQCDVNGTMRSILVDWLVEVSEEYKLRERTLYLAISYIDRFLSAMSVRRSKLQLVGTAALFIAAKFQEIYPPDCAEFAYITDDTYNIKQVLKMESLMLKVLSFNLSSPTAVDFLERYGSEAGLDSEIRELSMYLTELTLKDYGFLQFMPSLIAVSAVSLALHTFKLKYWPQELSTYTNYQWQQVSPCLNRIEAFRLAHTQPQRAVVEKY

>Tad-gi|196002535|

----DIYKNMLKQEKRCTLDPDYMT-GQPVITKGMRAILLDWLVDVHLRYNFHPESLYLTTYIIDRYLQTTQVNRKKLQLVGIAAFYIAIKYEEIFLASTDDLLYLTENSYEINEFIQMEAKILKALDFSLSRPTSIHFLRRISKAASADIEQHTFARYLTEIALIEYSLLSYLPSQIAAAASLISLKIFD-KSWTPTLQYYSSYSEDSLKPVARQI------------------

>Tad-gi|195999522|

----TAYVRKMVESCRNINIKLVNR-IQLELNDRMRYILIDWLVEVAEMKEFSSEMLCNAIDLVDRYLEINPIPRSNLQLLGISCMVIASRYHCVDIMTIREAAWLTDNTYKYDEVVRMIGEVFAAVNGEIRTPSAFDYLKIFCTISEVSQKCTYLASFILELSWLFLENSRYKSAVKAAASLLLARVLMGNLPWTEELKSYTGLSLEDLSSCVLHLK-----KCLAEKPPKDYY

>Tad-gi|196003236|

----EVWQVMIENDNNFKRSPDCFD-RHPNLVPNMRGILLDWMMEVCESFKMQRETFYMAMDYLDRYLSLSNILKQKLQLIGTTCLFIAAKIEEIQPPQVSEFAYVTDSACSEDDIIKLELQLLQTLEFQLSPVTVTSWLNVYVQLFNIKLSEMLYAIRLIDLCILDSWCLMHSYRSIAASAFYLIA-PS-----KQLAIDCTGYLWENLTSCISWM------------------

>Tad-gi|196001479|

----RVLQNLLKLEENYLPNRDYFTNVQREIKPFMRCMVTNWMLDVCHGENCNDKVFPLAVNYLDRFLSMVSIRKIRLQLVGSVCLFLASKLKDRIPLTAEKLCSWTDYSVTCQELLDWELLLLEKLKWDLGSVTPLDFLDQILYRLSFEKDYKKHAYTLIALCCTDFQLCTNPPSMIAGGCILCALA----NDNNRIVQKITSIEPDYLICC----------------------

>Tad-gi|196011367|

----HYSQWVLDRQEILAGREEDLSYLSEDEIFKIHMFFANFIRHLGDLLKLRQQVIATAIVYFKRFYSRNSLKSIAPLLLAPTCILLASKAEECG-IITGRFINACTNPYKMPVILECEFFLLELLDCSLIVFHPYRPLLQFVEDFEKK--LLPCAWRAINDSYNTDICLMYPPYIIALACLHTACIIQSITQWFAELS---------------L-------------------

>Tad-gi|196007260|

----LSLENTIYPDEKLQETPSQLDGLSKEMETNLRIAGCEYIQAAGILLKLPQVAMATAQVLYQRYYYSKSFVKYNYEICAMACIFLAAKIEEHP-RRIRDVVNVFYHFHKKSEVIKAERRVLKELGFCVHVKHPHKCIVTYLKILECNDNLIQKAWNYMNDSLRTDVFMRYTPETIACACIYLSARQLGIPPWYEVF------------------------------------

>Tad-gi|196000815|

-------GEKSKVVMPPVVKISAQQLIEDKDHWPEYLKIGKFIAESGIKLKLGSVVIARAATIYHRFYFLCDISQFDRYLVAVTCLYLASKVEDTP-RRARDVITTSYKWQLRDSVVNFELFMLRMLKFDVSSELPHKYLLHYLKSLQDSNWINQLCWQLLQDTSLLPFILLYPPSVIATAVIYLAVKCNNIKPWWNVFSN---LNEEGLQQCYKEY------------------

>Tad-gi|196007480|

---------WYFCSNEIVNSPSRSDGIEITAECRYRREGARFIMDVGNRMNLRYETVATGIVFYHRFYMMHSFKTINRLIGAAACLYLAGKAEETP-KKCRDLVKAVRTDDPKEEIISHERLLLQTIKFDLCVQHPYKYIVKFAKNLKDDRAVVQMAWNFVNDSLSTTLCLQWKPQVVAVSLLHLAAKLSKYKSWWQHFLE---INSDVLEDCLQDY------------------

>Tad-gi|196012973|

------------------------------ITPTQREALISSMINLSRKHNIKRDTLFMAVNYLDRFLKVVTVTEDCFELVGLTCMMIACKVEECQPPKMEEFLTSCTHYYKKAEMKRLEIIILNYIDFRLSPPIAPHFLEYIIHFHQHYIELVNIANQVLLKILPTYRFNHIKSSILAASAFEY--------------------------------------------------

>Tad-gi|195998946|

----LHNQFFQKLKDDDSRHIRTKDLPSVEELDHLCTFYEFELMDLCRRF----PMPATAAVYMKRFYLVCSVMDYHPCDIMLACVYLATKVDEYN-ISIDKFLTMVPEERAKSRTLGFELLVMEKLKFHLTIHCPYRPVEGLLINIMVIADLRRHIDKFLSKILYCQAMLIYPPSQVGEIALFPSQ------------------------------------------------

>Tad-gi|196013340|

----FAVTNFYFSDTELQNTPSRRNDISVATELYYRQTCALCIQELGMKLGANQLTINTALVYMHRFYMFHSLASYNLKNIAACAIFLASKSEEHP-NKLNKVITAAYELKLSQDLVDNEYAMFFTTGFDIEIMHPHTHVIKCLHG-----------------------------------------------------------------------------------------

>Tad-gi|196013209|

----CTLKCFICFALRMYRHDPPRLKDIFELHPLMRPHVYKFFKTLFSSAQLTAECAIISLIYVERLMEYAEIHPSNWRRVVLGAILLASKVWDDQAVWNIDYCQILKD-TTVEDMNALEREILQLIMFNINVPSSIY-AKYYF-------------------------------------------------------------------------------------------

>Nve-gi|156375154|

----DIFKYLKQAELNNRAKPGYMR-KQPDINNSMRAILVDWLVEVAEEYKLLPQTLYLTVNYIDRFLSAMSVLRGKLQLVGTACMLLASKFEEIYPPEVSEFVYITDDTYTAKQVLKMEQLVLKVLTFDLSVPTILNFLERFIKATNVPPKVEALARYLCEISLLSEPFLKYLPSTIAASAIVLSLHTLGLSYWNNTLSHYTGFELHDLQTCIQDLRSFAYAPNHPQQATREKY

>Nve-gi|156364707|

----EIMRFLRAMEEHYSVSPTYMN-NQQEVNEKMRAILLDWLVQVHLKFRLLQETLYITMSIIDRFLAVHQVSKRELQLVGVGAMLLASKYEEMFAPEIGDFVYITDHAYTKKQIRQMESLIFRKLDFSLGKPLCLHFLRRNSKAGAVGAEEHTMAKYLMELTLIDYQSIKFLPSEIAAASLSLAMRVMGKSEWTPTLEHYSGYSEKKLSTCMQRLQLVLGARDSKQKAVYNKY

>Nve-gi|156380901|

--------------------------KQKELTISMRAILVDWLVEVQESFELYHETLYLGVRVLDNYLMRSYVERENLQLVGAVSLYIACKVEERHPPCLDDFLYICDDAYQQKAFVAMEKKILNSLEFNINMPIPYRFLRRFAKVASADVKTLTLSRFILETTLHHYKFIVHKPSFLAAACLRLALRMKGCDDWTPTVVHYTGYSVAQLDGCVIELEMISEPPKQNLMTVRNKY

>Nve-gi|156363375|

SPLPELWSYMLEKDRKYTKDHLYLR-QHPHLQPRMRAILLDWLIEVCEVYRLHRETYFLAVDFVDRYLSVKDIPKQRLQLVGTTALFIAAKLEEIYPPKLSEFAYVTDGACKEDEILQQELLMLQDLNWKLCPITSNTWLNIYMQLHSCEALHFVIVSQLLDICSLDIESLQFSYSVLAAAAMYHV---I-----PVSIEEITCHKREDLSPCIQWM------------------

>Nve-gi|156350442|

----RVLTNLLACEERYLPSCNYFKIVQTEVEPHMRKLVATWMLEVCEEERCEEEVFALSMNYLDRILSLLPVKKFQLQLLGAVCMFIASKMKETSPLTAEKLCIYTDNSITTEELLDWELLVLGKLKWDVSAVTPHDFLDQIFSRLPLDRSTRKHASTFIALCCTDDKFLLYTPSMLAAASVCAAFTGLGIRSWASLLHAITNIEPECLRSC----------------------

>Nve-gi|156359300|

----QIMRYAMALENKYQLPENFLE-KQEEVSHQARAVLIDWLIEVHLFYNFPQDCLYLIVALVDRYMSLRTVPVAHFQLLGMACLLVACKYEDRFVPTREELVAMADQAFDQSELMHMETRLLTCLEFDLAQPLPTFFLRPIARASAIDLETYVVSKFIMEAAMLDAIMVTFKPSIIAATAFFMA-------------------------------------------------

>Nve-gi|156389378|

----SSDERWYFTKEQLQNSPSRRMGMDAERELSYRQQAATLIQDMGQRLSVSQLTINTSIVYMHRFYMCHPFQKFHRHAMAPCCLFLSAKVEEQP-RKLEHVIRVAHALQQAQDLIENESILLQTLGFEVTVHHPHTYVVKGIQLVRASK-LGQASYFMATNSLLTTLCLQFKPPVVACACIHLACKWCNYKYWWQYINT---VTKKLLDEAQE--------------------

>Nve-gi|156407073|

----DIHEVLREKEAR---IPNFMA-AS--PQLKIRRYLVDWLAVIGEKLGSSHGVVHLAIYYMDFFMDKFIIQESQLHLLALTALLLAAKFDENEIPDISTLNKFVNNTYQHAEYHQMELLLLEFFNWNIDLPTPVQFLEYYLAKATIDYKLRKYVYYFLEISFQDHTFLSFSPSLITSSCIAASRICLNLPSWTNELSKVTNYDWDKIAHCTEIM------------------

>Nve-gi|156397054|

--------------------ERAARRKATYAYLKEHFKVTQFIMESGAKLSLPQNAMSSACVLYHQFWKGCDPKDFDPYLIGMTAIYLASKAEECP-CKVRDVINVCYRWELRESVVNCELLMLRVLGFRVSYDNPHKYLLHYLKVLQDGMWVPQISWSYLLDSHHIPLCLEYPPAHVAVALLHFAVECVGLRPWWKALCD---VTPELIQSTEDDY------------------

>Nve-gi|156405242|

---------WLFTHDGLNRTPSRLDGIDYATECRYRREGTRFIMECGNKMGLRYDTMATGAVYFHRFYMIQSFKNFPRWVTGAACLFLAGKVEETP-KKCRDIIKTANSPDPKEEVMIYERILLQTIKFDLQVEHPYPCLLKLGKGLKGDRALVQMAWTFINDSLSTTLCLKHRSEVIANAMLALAAKLNN--------------------------------------------

>Nve-gi|156381346|

----NVNSAYKSRHSEAYPDKKNVKYLTVEEEKKLVEYYELVIVEVSAKFPVPRSVTATAITYLKRFYVKTSVMDHPPKEMFLVCLFMACKVEEYN-ISVENFVQILPREKVMDFILAHELLLMERLDFHLTIHHPFRPMEGFLIDIKYGKVWRIKAEEFLLRAMRTDVAFHFSPSQIALAALSVGST-----------------------------------------------

>Nve-gi|156365799|

----AEFLTQFLQKSKALNSHKLFG-FQTELNNTMRYILVDWLVEVALMKDFSSQIVHIAVHCVDQYLMKRKVQRSELQLLGITCILIAARFQGKDIVTIREASWLTDDTYSYEEVVRMMGEVMSCLRGEV--------------------------------------------------------------------------------------------------------

>Nve-gi|156359932|

----HCNQWMLDVDEIMIGRQQDLQFLTEVEYQKVHIFYSNFMQSLGEHLDLRQQVIATATVFFKRFYSKNSLKSIDPLLIAPTCVYLASKVEECG-AINNKLISASSVPYRMNQVLECEFYLLEMLDCCLIIYHPYRPLTQYVSDLGME--ILPTAWRIINDSLRTDIFLIYPPYLIALAAIHMACVIQQKKQWFAELS----VDMDQIVETHHR-------------------

>Nve-gi|156408249|

-----------------------------------RDKSVCILLHINRHCGFQPETFALAVNLLDRFLSVVKANPKYLPCISISCMFLAAKMVEEDIPTAGNLIGVSGLSCTPSDLLRMERIILDKLGWNLSAVTPLQLLQVFHALCV----LHHITLK-LEELLCNHKFTFFKPSTLALSLLSCEISSL--NVW----------------------------------------

>Nve-gi|156365937|

----ITLENCILSPDKLTETPSVKDGLDKNVEEDLRIIGCEFIQTSGLLLKLPQVAMATGQVLFQRFYYTKSFVKHDVEVGSCTCYYCTSKRNGQI-KATKHRIVSVRGFNRKNQVVKAERRVLKELGFCVHVKHPHKIIITYLQILECETNLAQLAWNHMNDSLRTSAFVRFAPETIACACIFLASRLLKIPPWYELFD-----------------------------------

>Nve-gi|156406586|

----KCVSLAVYYHIKNRDPEVRTVDIFDLHPLMNDPHIYKFIKTLFHAAQLTAECAIITLVYLERLLTYAEVYPGNWKRILLGAILLSSKVWDDQAVWNVDYCQILKD-VTVEDMNELERAFLEFLQFNINVPSSVY-AKYYF------------DLRSLADA--NDLMFPLLS------------------------------------------------------------

>Aqu-gi|340374274|

----DIYQYMREREVKFK-VSSYLD-HQPLINSSMRSILIDWLVEVQENFELFHETLYLAVKIVDRYLEKKEVKKEYLQLVGATSMLIAAKFEELSPPLVDDFIYLCDDAYQHDELLSMERNILATLEYDVNAPVAYRFLRRLARAAGADMETHTLARYICESTLQEYEFVSDDPSHIAGAAMYLSIRMKGLGGWTPTLQHYSQYEASNLLPMVQRLD-LISRPAGNTSTVRSKY

>Aqu-gi|340380414|

----YQYVQQIVRSSPSLLSASIYD-AQPDVNEEMVFILLDWIVEVAEMKSFSTKTLHLAISLIQRYMVARKLKRSRLQLLGVTALLLAARWTAVPIITIREAAWLTDNTYRYDEVVCMMGEIVSTLHGEIQKPTVPDYLEMFELLVNADKKSSCLAAYVSESAVLFPDFGRYTAAQIAAGCLLLARVLLEQLPWPSALVEATGLTVPDLYHCTSLLQ-----RRSSNSSLLKYR

>Aqu-gi|340379787|

----LWREMRLKDTSQAAPGTELRL-RHPSIMPTMRTILLDWMLEVCEEYRIHRETYYLSLELFDRFMDTQNVQKEQLQLIGVTCLFIASKIEEIYPPKLADFAYVTDGACNSEEIVFMELMICKALKWRLHHCSVNTWVNLYMQL-SYFRPLFEYVMQVLDLCTLDITSRQFCNSILAASALYLVSEKC-----QMHLNLVTGFQLADIHVCVQWLIHEVNINMLEEAQDIIKY

>Aqu-gi|340376468|

----QLESSLREKSEDLARAKQLHSAELANLMDEMRALRVNYDQKMKKRKKQVAATSSSAVDPDGRFVQVTEIPKNKLQLVGVTAMLIASKYEEMYAPEVADFVYITDSTYSNTEIKAMERNILKTLDYSFGNPLCLHFLRRNSRAGDATPQMHTMAKFLMELCLPDYSMLEYLPSLVAAAALYISNKLYSDGEWTPALRHYSQYTEPDVLPCVGKMSLVLSMHTAKQQAVKNKY

>Aqu-gi|340379451|

----FKEWLLDKQEIEVWRQKDVSYFSSSEDYQKLMIFFANFIQTLGEQLKLRQQVISTAIIYFRRFYSRHSLGDVDPFLLGPTCLYLASKVEECG-VVPGTL-YIRKSSYKAQLIMECEFLLLEMLDCCLIVYHPYRPLTQYVTDLGQE--LLPTAWKIVNDTYRSDICMLYPPYLIALVAIHMAAVVHKKKAWFAELS----IDMNKIIETNLD-------------------

>Aqu-gi|340373128|

----EDIKWIFSAEVLEHLTPSRKKGISHEMERRYRREGARFISNTSNTLKLRRDTLATGTVFFHRFYMVQNFADFDKYVVAAACVLLAGKVEETP-KKCKDIVRVAKREKPLEELISFERVLLQTIRFDLQVDHPYGYLLKFAKHMKGEKQVLQMAWTFINDSLCTTLCLQWEPPVVAVALLYLAGKLSKFRSWWRQFVT---VDAHDLESCHQDY------------------

>Aqu-gi|340369567|

----PIHETLKKREAA---ISSIQF-KS--PQLHLRRELVEFITAVSKDLGLSDGTRFLAIRLADQFMDGHNVMEYRLRLMGLTCLLLAAKSEEIDVPSIEMLQHANTSAYSRQEFHTLELYILKYFKWCLSHPSVAHFIDYYLHTSLKENLMKEFTAYFMEVTLRGI-KANSLSRHFLINCKDAAGILPAACKW----------------------------------------

>Aqu-gi|340380250|

----APPRRWYYSREQLSNSPSRADGVDPEKELRYRQDAASLIQDMGPKLNLNVLCMSTAIVYMHRFYMINSFKAFDRVLLATAALFLAAKVEEHP-RKLEHVAKCSYSTKLIDDITYHELVLLQTLGFDVQVKHPHPHVVQCMNLVGVSR-LSQAAFFLAHNSQLTTFCLEHPPTVVACMCIHLTCAWKGLKNWWEYVDS---VTYDKLEGATEN-------------------

>Aqu-gi|340373665|

----LSSSNLLVPKERLINTPSRKDGMSRDLEIDMRVTGCHYIQSAGILLKLPQVAMATAQILYHRFYYAKSFVKFKCYYTMMACLFLAAKLEESS-RRLRDVINVFHHFRLRNLIIKHERYILKELGFCVHVQHPHKLIISCLQILELEKNLIQKAWNYMNDSLRTNIFLRYNVQTIACSCIYIATGHLKVPPWWELFD----VNYTDMKTSLE--------------------

>Aqu-gi|340370065|

----SKTYTYSQAIYH---IEENPR-SYKYVTARDRDEQVSWFRNITHDLSFSISVFYQSTLLLDTFLSTKKTKREFLQTMAASCYSIATKLVESCVALSHKLVSKYYSGCSVTDVEKMEAFLIQTPDIASALHTIQDYIKKFHRLA--ERSLCHLYKC-AQHVVCNHELMKYRPSMLGLAILGCHLKQL--CDWLSTLLEILQIRGGELSICY---------------------

>Aqu-gi|340377763|

----AETNASFCRKYSDAAISKECQFLTPTEEESVCLYYMKKLFEFCNVFPVPRGVLGTAGAYFKRFYLLTSVMDYHPKEIFLSCAYLAFKIEEYN-VSLDEFVYMLSPQSSSEMILNNELMMLKRLKFHLTIHSPFRPLEGFLIDMKTSIPLRKEADSFLMSSLYSDVLFLYPPSQIALAALYYASTVIEV-------------------------------------------

>Aqu-gi|340376480|

-------------------AAGTEDKNHEKSGYSPVLEETLFVAKAGLRLRMSEVAIATALQYYHTFQSTMEKNRFDENLVAMACLFLAAKAQEVS-LRLSDLVNTCYHWQLKESVAKMELVLLRALKFEFQLDLPHRYLLHHLLSLSHSQWVTRLSWSLLQDSFHTTLNHIHPPNKMAVAVLYLAVKVSRLYQWWEVMCG---VTEPELQTCEANY------------------

>Aqu-gi|340378990|

----ASLAVFYIVKGRPRDRPPKTLDIFDLHPLTRDPLIYKFIKTLFHAAQLTSECAIITIIYLERLLQYSELHPCNWKRILLGAILLASKVWDDQAVWNVDYCQILRE-ITVEDMNELERVYLEQIQFNINVAAKMY-AKYY--------------FDLRTLS--EENGLTFEY------------------------------------------------------------

>Mbr-gi|167523717|

----DIYNYMREMEVRLCCDPAYLQ-SQPEVNERMRAILIDWLVEVHYRFELLQETLYLTVDVLDRFLSSERTSRSQLQLVGVTAMLIASKYEEMYPPEVGDFVYISDNAYRREQILAMEQTMLRVLDFNLGKPLPLHFLRRDSRAGHADGTMHTFAKYFMELTLCSPRFLGYKPSQVAAAATYISREVVGEQLWTPTIEFFADYTLTDIMPVILDMAILRESPTAKQQAVRTKF

>Mbr-gi|167517989|

------------------------R-KQRDINHTMRSILIDWLIEVTEEYKLTLQTFFVTVGYVDRFLSEMAVQRSKLQLVGVTCMLLACKYEEIYPPTIDDFVYITDKTYSRPQVMKMEHVILKVLRFDMGSCTPLTFLYYFLNAIPHHDDTKWLAQYLCELSAYGRRSLGQRPSTTAAAAIVIALHTFELHPLPPALVSVIRQGPEELQAAVNTLEIFSVYPNLQHEAIKEKY

>Mbr-gi|167522783|

----CMAFSREAFARGAAPVRRGGL-RQPDREGKMRFILMDWLIEVADLKTFGGETLFVAMDLVDRFLQHCRITRKTLQLLGIACMVMAARYLEEGVITIREAAWLTDSMYSYDQIVRTIGQVLVDVSGNVIRPTTFHYLNLLLQIGGATPAVFLLGQHMAEALILTIPLTEFPPAKLAAAMACCTFALAGVQPWSSTMERWSGLELRTIYDSA---------------------

>Mbr-gi|167526102|

----EVHRNMRVQESQ---LQLAQY-CG--PLADLRPRCVQRIHRLARAFRFHRLTRDAAIFYFDRMLFLFHMHESHLELAVQTAFLMAAKCQEAEVPTHHDLHRAGCAVVPTAHLKAFEASYLERVDWILTSVTPSDFLDYYARFSISDCLTLDLADQVLQEASMANHVATFLPSHRAAAAITTARLIVDIPAWSPTLQAVSGLTWREISPCVDAMQ-----------------

>Mbr-gi|167524110|

---------------------------------------------IYHGITPSANALYFAIAYLQRYAVTTTLMEHHPDLICLTCWLLAVKASEAK-VPLHCLVAVAQRKDVEDQVRQLESSLAQKLNYEFYVSLPSRAMRAILIELQGPAPDEKSIMAALLNLWASPAQLQMAPVQLCLAALG---------------------------------------------------

>Mbr-gi|167526156|

----FPCLDSDLPRFDLANTPSRRRGIPAELELRLRIAGCELIQKTGMLLGCKQVVMACAQMLLQRAYCRLDISRHSLQWVGLACLFLAAKTEEDH-QRLRSILLVGRVHELKNNVIKSERRVLKELGFCVHLKHPHKDV-------------AQLAWNYMNDALRSDVFLRFEVAVIACACIDLATRKLDIDLWFQSFG----VHPDDFEQCATQY------------------

>Sro-gi|326426811|

----KVMTYLRHLEKKFRPHAGYMG-RQRDINHNMRSILVDWLVEVTEEYRLQLQTLYIAVGYIDRFLSNMAVQRSKLQLVGVTCMLLAAKYEEIYPPSVNEFVYITDNTYRREQVLKMEHVVLKVLRFDMGACTALTFLVRFIHAASATPPSHCLALYLAELSLLGNKFIQYLPSVKAAAAICLSQHTFARPVWTPTFERYCRLSPEEVQPCLNDMEAMTSAPHLEYQAIREKY

>Sro-gi|326435281|

----AIYAKNLQREE---KYLLNPR-PSRGINLAMRTVVIDWMIEIQVSFKLRDETLFCAVDILDRYLAARPEQRHDYQCCGATSLWIASKFLEVLPPELADFEYVCAGLYPRQAFIDKELTMLTALRFYVMNVTPLDFISVYAIVLQLSLEGMALAEYLITLPLQEQRFYGLRPSVRGAAAVHIASKTCDGPGWSEDHSALFKLDHRHIMLVAQ--------------------

>Sro-gi|326437566|

----VALALSYVVEDGHKQDNPRLFSEKFKRHPITDRRIYKFMFQLFNSAQLTAECAIITLVYVNRLIAYTSLHASTWKRVVLGAILLASKVWDDQAVWNVDFCSMLPS-VAVEDMNDLERTFLEMLDFNIDVDSCV------Y------------AKYYFELR--ALAEKFEFP------------------------------------------------------------

>Sro-gi|326437558|

----QYYRVLEQRLASCEVSINALK-MQPCMPPRLRARVFHWLADVCDRARMTLDTLFFAITYFDTYCSVRAVTVANMQLLACACLRLAAKIEETRVPSLRLLSRLTDGACQPTGIAQFELDLAAVLKWRLIRSTPLHWTRFFIGVA---LGSSAPSCQVLQLAMSDAWALRFDARQMAAAAVLLSA-TR-----PIDILAVTGLDKCALRTCLRWVRAVGFCHDGSSCGASGLK

>Sro-gi|326437989|

----FRETVELGMEKLRLRSDAPPP-AQPDSQGKMRFILVDWLLEVASLKMYSIDTLHCAVDMVDRYLATRTITRRTLQLLGITCMVIAARFLEQDVVTIREAAWLTENTYDYEDVVQMVAAVLAVADGHVRRPSPNDYITIFAELSNVPVHIRCFMDYVSESCLLHQPTLTHAPAALGAAIYFVSMHLVGCSAWPSSLTSNSDLRVSH---EI---------------------

>Sro-gi|326428978|

----PIFEYMRELEVRLHVPANYFK-IQTEINARMRDVLVDWLAEVHHRFELIQETFHLTVHLLDRYLSKEPVTRDDVQLVGITAMMVAAKYEEMYPPELGDYVYITDKAYSEDRILAMERKLLRVLDFSLGKPLPLHFLRRNSKAGHADATMHSMGKYMIELSLGSHAMLKYVPSQLAAAATYISREIVGEELWNPTLEHYAKYSLEDIAPVVHDMAVLKHSTVSRLQAIRNKF

>Sro-gi|326429298|

-----------LTKEQVADTPSRKDGIDADREARYRRECIHFIKQLAIRFQLSPRVYMTAMVIFHRFFLTHSFKDLSRLNFAAACLFIGGKIEEQP-KRMQDFLPVVHEARLRFILQGCERAVLQTIDFELSYDHPLEPLLQYA------------------------------KTEIAAALLELASRKSSSRPWFE--------------------------------------

>Sro-gi|326437823|

----------SLPRYDLQNTPSRAHGVSEELETRLRVAGCEIIQSAGILLNCNQVVMACAQILYQRFYYRQSFATQRFEVTAMGCLFLASKVEEEQ-QRLRILMNVCRHHNLKHRVIKAERLVLKELGFCVHLDHPHKLIISMQSVLSLEDNLAQRAWNYMNDGLRTTVFVRYTTATIACACLDLACTDVGIDQWYELFG----LMGFAPAPMGAAA------------------

1. **Multiple alignments for phylogenetic analysis of cyclin family proteins in H. sapiens, T. *adhaerens*, *C. intestinalis*, *B. floridae*, *S. purpuratus* and *D. melanogaster*.**

>Hsa-CyclinA1

EIYQYLREAIRHRPKAHYMK-KQPDITEGMRTILVDWLVEVGEEYKLRAETLYLAVNFLDRFLSCMSVLRGKLQLVGTAAMLLASKYEEIYPPEVDEFVYITDDTYTKRQLLKMEHLLLKVLAFDLTVPTTNQFLLQYLRRQGVCVRTENLAKYVAELSLLEDPFLKYLPSLIAAAAFCLANYTVNK-HFWPETLAAFTGYSLSEIVPCLSELKAY-L-DIPHRPQQAIREKY

>Hsa-CyclinA2

DIHTYLREMVKCKPKVGYMK-KQPDITNSMRAILVDWLVEVGEEYKLQNETLHLAVNYIDRFLSSMSVLRGKLQLVGTAAMLLASKFEEIYPPEVAEFVYITDDTYTKKQVLRMEHLVLKVLTFDLAAPTVNQFLTQYFLQQPANCKVESLAMFLGELSLIDDPYLKYLPSVIAGAAFHLALYTVTG-QSWPESLIRKTGYTLESLKPCLMDLQTY-L-KAPQHAQQSIREKY

>Hsa-CyclinB2

DIYQYLRQLVLQSINPHFLD--GRDINGRMRAILVDWLVQVHSKFRLLQETLYMCVGIMDRFLQVQPVSRKKLQLVGITALLLASKYEEMFSPNIEDFVYITDNAYTSSQIREMETLILKELKFELGRPLPLHFLRRASKAGEVDVEQHTLAKYLMELTLIDYDMVHYHPSKVAAAASCLSQKVLGQ-GKWNLKQQYYTGYTENEVLEVMQHMKNVVKVNENLTKFIAIKNKY

>Hsa-CyclinB1

DIYAYLRQLEEQAVRPKYLL--GREVTGNMRAILIDWLVQVQMKFRLLQETMYMTVSIIDRFMQNNCVPKKMLQLVGVTAMFIASKYEEMYPPEIGDFAFVTDNTYTKHQIRQMEMKILRALNFGLGRPLPLHFLRRASKIGEVDVEQHTLAKYLMELTMLDYDMVHFPPSQIAAGAFCLALKILDN-GEWTPTLQHYLSYTEESLLPVMQHLKNVVMVNQGLTKHMTVKNKY

>Hsa-CyclinD3

RVLQSLLRLERYVPRASYFC-VQREIKPHMRKMLAYWMLEVCEEQRCEEEVFPLAMNYLDRYLSCVPTRKAQLQLLGAVCMLLASKLRETTPLTIEKLCIYTDHAVSPRQLRDWEVLVLGKLKWDLAAVIAHDFLAFILHRLSLPRDRQKHAQTFLALCATDYTFAMYPPSMIATGSIGAAVQGLG--ACSMELLAGITGTEVDCLRACQEQIS-------------------

>Hsa-CyclinB3

EIFSYMKEREQFIL-TDYMN-RQIEITSDMRAILVDWLVEVQVSFEMTHETLYLAVKLVDLYLMKAVCKKDKLQLLGATAFMIAAKFEEHNSPRVDDFVYICDDNYQRSEVLSMEINILNVLKCDINIPIAYHFLRRYARCIHTNMKTLTLSRYICEMTLQEYHYVQEKASKLAAASLLLALYMKKL-GYWVPFLEHYSGYSISELHPLVRQLKLL-T-FSSYDSLKAVYYKY

>Hsa-CyclinD2

RVLQNLLTIERYLPQCSYFC-VQKDIQPYMRRMVATWMLEVCEEQKCEEEVFPLAMNYLDRFLAGVPTPKSHLQLLGAVCMFLASKLKETSPLTAEKLCIYTDNSIKPQELLEWELVVLGKLKWNLAAVTPHDFIEHILRKLPQQREKLKHAQTFIALCATDFKFAMYPPSMIATGSVGAAICGLQQDVSSLELLAKITNTDVDCLKACQEQ-E-------------------

>Hsa-CyclinE1

EVWKIMLNKKTYLRDQHFLE-QHPLLQPKMRAILLDWLMEVCEVYKLHRETFYLAQDFFDRYMATENVVKTLLQLIGISSLFIAAKLEEIYPPKLHQFAYVTDGACSGDEILTMELMIMKALKWRLSPLTIVSWLNVYMQVAYLNDHEVLLIAELLDLCVLDVDCLEFPYGILAASALYHFS-SS-------ELMQKVSGYQWCDIENCVKW---------------------

>Hsa-CyclinE2

EVWLNMLKKSRYVHDKHFEV-LHSDLEPQMRSILLDWLLEVCEVYTLHRETFYLAQDFFDRFMLTKDINKNMLQLIGITSLFIASKLEEIYAPKLQEFAYVTDGACSEEDILRMELIILKALKWELCPVTIISWLNLFLQVDALKDPKVLLIAQLLDLCILAIDSLEFQYRILTAAALCHFT-SI-------EVVKKASGLEWDSISECVDW---------------------

>Hsa-CyclinK

-----WDKKLAHTP--SQLEGLDPATEARYRREGARFIFDVGTRLGLHYDTLATGIIYFHRFYMFHSFKQFPRYVTGACCLFLAGKVEETP-KKCKDIIKTARSDDPKEEVMVLERILLQTIKFDLQVEHPYQFLLKYAKQLKGDKKLVQMAWTFVNDSLCTTLSLQWEPEIIAVAVMYLAGRLCKFERRWWEQFQD---VPVDVLEDIILDL--QQMPHHTPHQLQQPPSLQ

>Hsa-CyclinJ

DIHQALRYKLKLPS---YKG-QSP--QLSLRRYFADLIAIVSNRFTLCPSARHLAVYLLDLFMDRYDISIQQLHLVALSCLLLASKFEEKEVPKLEQLNSLMNLVLTKQNLLHMELLLLETFQWNLCLPTAAHFIEYYLSEAVHETYMAKYADYFLEVSLQDYAFLNYAPSLVAAACVASSRIILRS-PTWPTRLHRLTAYSWDFLVQCIERLV-------------------

>Hsa-CyclinF

ANQLGIVCQAQAVSKQQVFS-VQKGLNDTMRYILIDWLVEVATMKDFTSLCLHLTVECVDRYLRRRLVPRYRLQLLGIACMVICTRFISKEILTIREAVWLTDNTYKYEDLVRMMGEIVSALEGKIRVPTVVDYKEVLLTLVPVELRTQHLCSFLCELSLLHTSLSAYAPARLAAAALLLARLTHGT-QPWTTQLWDLTGFSYEDLIPCVLSLKKDAPKDYRQVSLTAVKQRF

>Hsa-CyclinO

SCYAFRKAQSHFHP-REALA-RQPQVTAESRCKLLSWLIPVHRQFGLSFESLCLTVNTLDRFLTTTPVAADCFQLLGVTSLLIACKQVEVHPPRVKQLLALCCGAFSRQQLCNLECIVLHKLHFTLGAPTISFFLEHFTHA-REALEAQALARGVAELSLADYAFTSYSPSLLAICCLALADRMLR------LR---LGDHPEAALEDCMGKL--------------------

>Hsa-CyclinD1

RVLRAMLKAETCAPSVSYFC-VQKEVLPSMRKIVATWMLEVCEEQKCEEEVFPLAMNYLDRFLSLEPVKKSRLQLLGATCMFVASKMKETIPLTAEKLCIYTDNSIRPEELLQMELLLVNKLKWNLAAMTPHDFIEHFLSKMPEAEENKKHAQTFVALCATDVKFISNPPSMVAAGSVVAAVQGLNLRNNFLRFLSRVIKCDPDCLRACQEL-S-------------------

>Hsa-CyclinL2

-----LPDDLRFTP--SMSSGLDTDTETDLRVVGCELIQAAGILLRLPQVAMATGQVLFQRFFYTKSFVKHSMEHVSMACVHLASKIEEAP-RRIRDVINVFHRVNLKNQIIKAERRVLKELGFCVHVKHPHKIIVMYLQVLECERHLVQTSWNYMNDSLRTDVFVRFQPESIACACIYLAARTLEIPPHWFLLF-----ATEEEIQEIILQL--------------------

>Hsa-CyclinG2

NVYLE---QERFQPGLSTPE-NDNTLCPGLRNAKVEDLRSLANFFGSCTETFVLAVNILDRFLALMKVKPKHLSCIGVCSFLLAARIVEENIPSTHDVIRISQCKCTASDIKRMEKIISEKLHYELEATTALNFLHLYHTIILC---KLSLDKLQLKACNCRLIFSKAKPSVLALCLLNLEVETSP--ECCKPDLVWIVSRRTAQNLHNSYY---------------------

>Hsa-CyclinT1

-----FTRELENSP--SRRFGVDPDKELSYRQQAANLLQDMGQRLNVSQLTINTAIVYMHRFYMIQSFTQFPGNSVAPAALFLAAKVEEQP-KKLEHVIKVAHTLQQVQDLVILESIILQTLGFELTIDHPHTHVVKCTQLVRASKDLAQTSYFMATNSLHTTFSLQYTPPVVACVCIHLACKWSNWEKHWWEYVAT---VTLELLDELFLQIQKNMEANVKSQYAYAAQNLL

>Hsa-CyclinT2

-----FTRELENTP--SRRCGVEADKELSCRQQAANLIQEMGQRLNVSQLTINTAIVYMHRFYMHHSFTKFNKNIISSTALFLAAKVEEQA-RKLEHVIKVAHALQQTQELVILETIMLQTLGFEITIEHPHTDVVKCTQLVRASKDLAQTSYFMATNSLHTTFCLQYKPTVIACVCIHLACKWSNWEKHWWEYVPT---VTLELLDELFLQISTSSHQEWPQHQDSARTEQL

>Hsa-CyclinL1

-----IPEELSPTP--SMQDGLDLPSETDLRILGCELIQAAGILLRLPQVAMATGQVLFHRFFYSKSFVKHSFEIVAMACINLASKIEEAP-RRIRDVINVFHHINTKNQVIKAERRVLKELGFCVHVKHPHKIIVMYLQVLECERTLVQTAWNYMNDSLRTNVFVRFQPETIACACIYLAARALQIPPHWFLLF-----TTEEEIQEITLRL--------------------

>Hsa-CABLES1

TVIDYVPSDKK-----DF-KEKFPHILSKIRSLKREMRKLAQEDCGLEEPTVAMAFVYFEKLALKGKLNKQNRKLCAGACVLLAAKIGSDLKHEVKHLIDKLEERLNRRELIAFEFPVLVALEFALHLPEH-EVMPHYRRL--------------------------------------------------------------------------------------------

>Hsa-CyclinC

-----DKQDLKERQ--KDLKFLSEEEYWKLQIFFTNVIQALGEHLKLRQQVIATATVYFKRFYARYSLKSIDPVLMAPTCVFLASKVEEFGVVSNTRLIAAATSPYRMNHILECEFYLLELMDCCLIVYHPYRPLLQYVQDMGQEDMLLPLAWRIVNDTYRTDLCLLYPPFMIALACLHVACVVQQKDRQWFAEL-----VDMEKILEIILKL--------------------

>Hsa-CyclinG1

NALLE---QSRCQPGLRAHD-NGLRMTARLRDFEVKDLLSLTQFFGFDTETFSLAVNLLDRFLSKMKVQPKHLGCVGLSCFYLAVKSIEENVPLATDLIRISQYRFTVSDLMRMEKIVLEKVCWKVKATTAFQFLQLYYSLLQEN--RINFERLQLKACHCRIIFSKAKPSVLALSIIALEIQA---------QELTEGIECLQKHSKINGR---------------------

>Hsa-CyclinH

-----EQLALRADA--NRKFRCKAVANGKVLPNDPKRLLEFCSVFAMPRSVVGTACMYFKRFYLNNSVMEYHPRIIMLTCAFLACKVDEFN-VSSPQFVGNLREEKALEQILEYELLLIQQLNFHLIVHNPYRPFEGFLIDLKTYLILRKTADDFLNRIALTDAYLLYTPSQIALTAILSSASRAG-----------------------------------------------

>Hsa-CyclinI

RLSFL---LKAITRMWKKMP-SNQNVSPSQRDEVIQWLAKLKYQFNLYPETFALASSLLDRFLATVKAHPKYLSCIAISCFFLAAKTVEERIPVLKVLARDSFCGCSSSEILRMERIILDKLNWDLHTATPLDFLHIFHAIAVSTRPSQHLAVLQLLHCMACNQLLQFRGSMLALAMVSLEMEKLI--PDWLSLLLQKAQMDSSQLIHCV-----------------------

>Hsa-Fam58A

------PEGGGGPA--ARGPEGQPAPEARVHFRVARFIMEAGVKLGMRSIPIATACTIYHKFFCETNLDAYDPYLIAMSSIYLAGKVEEQH-LRTRDIINVSNRWELRDSIVQCELLMLRVLRFQVSFQHPHKYLLHYLVSLQNW-PVAVTAWALLRDSYHGALCLRFQAQHIAVAVLYLALQVYGVEKPWWQVFDD---LTKPIIDNILIQI--------------------

>Hsa-CABLES2

TVIEYVKPSLK-----KT-FREKFPHTLSKIRSLKREMRSLSEECSLEPVTVAMAYVYFEKLVLQGKLSKQNRKLCAGACVLLAAKISSDLKSGVTQLIDKLEERFNRRDLIGFEFTVLVALELALYLPEN-QVLPHYRRL--------------------------------------------------------------------------------------------

>Hsa-Fam58B

-----AGEEGEDAG--EDAREGAAAPAARVHFRVARFIMEAGVKLGMQSIPIATACTIYPKFFCETILDAFDPYLIAMSSIYLAGKVEEQP-LWAHDIISVSNRWELRDSIVQRELLMLRVLRFQVSFQHPHKYLLYYLVSLKNW-PVAVTAWALLRDSYHGGLCLRFQAQHIAVVVLYLALQVYGVEKLWWQAFDD---LTKPIIDTILIQI--------------------

>Hsa-CyclinI2

LDERR---LCHLQLREAGGK-PQDEICDAFE-EVVLWLLRLQNTFYFSQSTFNLALTIFGRLLISVKVKEKYLHCATITSLRLAAKVNEEFIPQVKDFTKHYGSDYSPNELLRMELAILDRLHWDLYIGTPLDFLTIFHALVVLSWPNLHVASLQLQHCMAGHQLLQFKGSTLALVIITLELERLM--PGW------------------------------------------

>Hsa-CyclinJL

DVHCTLREKLKLPT---FRA-HSP--LLKSRRFFVDILTLLSSHCQLCPAARHLAVYLLDHFMDRYNVTSKQLYTVAVSCLLLANGFEDREVPKLEQILSSQNFTLTKKELLSTELLLLEAFSWNLCLPTPAHFLDYYLLASVSQDCLKEYAHYFLEVTLQDHIFYKFQPSVVAAACVGASRICLQS-PYWTRDLQRISSYSLEHLSTCIEILS-------------------

>Hsa-CyclinYL

EDLALRASTSSQTDKRKNHLSNILPHKEQREKFIYRFVRTLFSAAQLTAECAIVTLVYLERLLTYAEICPTNWKRIVLGAILLASKVWDDQAVWNVDYCQILKD-ITVEDMNEMERHFLELLQFNINVPASVY-AKYYFD-----------------LRLADDNNL-NFLFAP-------------QLMQR------------------------------------------

>Hsa-CyclinY

IFLDDKYTIAAIYYNRDMLIFDENLHPLSKSKQIYRFVRTLFSAAQLTAECAIVTLVYLERLLTYAEICPANWKRIVLGAILLASKVWDDQAVWNVDYCQILKD-ITVEDMNELERQFLELLQFNINVPSSVY-AKYYAI------------------------------------------------RRW------------------------------------------

>Tad-gi|196003740|

IIFENMKQRAQLVV-NDYLE-RQNDITEQMRMILIDWLCEVQQNFELFHETLYLAVKIVDRFLSARVVSRDALQLIGATAMLMSSKIEERYPPLVDDFVYICDDAYSRQAVLDMERDICYALDFDLNIPIPYRFLRRYGKVASLSMENLTLARYILELTLQEYQFVTFKPSMLAAGCLCLALKMKNC-GEWTQTLVHYSGYEESELNELVQKLAMI-A-KPAPENCKVVKTKY

>Tad-gi|196005765|

DIHSYLRKAYFHRPKYDYME-RQCDVNGTMRSILVDWLVEVSEEYKLRERTLYLAISYIDRFLSAMSVRRSKLQLVGTAALFIAAKFQEIYPPDCAEFAYITDDTYNIKQVLKMESLMLKVLSFNLSSPTAVDFLERYGSEAGLDSEIRELSMYLTELTLKDYGFLQFMPSLIAVSAVSLALHTFKL-KYWPQELSTYTNYQWQQVSPCLNRIEAF-R-LAHTQPQRAVVEKY

>Tad-gi|196002535|

DIYKNMLKQKRCTLDPDYMT-GQPVITKGMRAILLDWLVDVHLRYNFHPESLYLTTYIIDRYLQTTQVNRKKLQLVGIAAFYIAIKYEEIFLASTDDLLYLTENSYEINEFIQMEAKILKALDFSLSRPTSIHFLRRISKAASADIEQHTFARYLTEIALIEYSLLSYLPSQIAAAASLISLKIFD--KSWTPTLQYYSSYSEDSLKPVARQIK-------------------

>Tad-gi|195999522|

KKFTGYVRKSRNINIKLVNR-IQLELNDRMRYILIDWLVEVAEMKEFSSEMLCNAIDLVDRYLEINPIPRSNLQLLGISCMVIASRYHCVDIMTIREAAWLTDNTYKYDEVVRMIGEVFAAVNGEIRTPSAFDYLKIFCTISEVSQKCTYLASFILELSWLFLENSRYKSAVKAAASLLLARVLIGE-LPWTEELKSYTGLSLEDLSSCVLHLKKKPPKDYYNSEVKSVHNRY

>Tad-gi|196003236|

EVWQVMIENNNFKRSPDCFD-RHPNLVPNMRGILLDWMMEVCESFKMQRETFYMAMDYLDRYLSLDNILKQKLQLIGTTCLFIAAKIEEIQPPQVSEFAYVTDSACSEDDIIKLELQLLQTLEFQLSPVTVTSWLNVYVQLFNIKPGDMLYAIRLIDLCILDSWCLMHSYRSIAASAFYLIP-SK-------QLAIDCTGYLWENLTSCISW---------------------

>Tad-gi|196001479|

RVLQNLLKLENYLPNRDYFN-VQREIKPFMRCMVTNWMLDVCHGENCNDKVFPLAVNYLDRFLSMVSIRKIRLQLVGSVCLFLASKLKDRIPLTAEKLCSWTDYSVTCQELLDWELLLLEKLKWDLGSVTPLDFLDQILYRLSFELKYVKHAYTLIALCCTDFQLCTNPPSMIAGGCILCALAG---VQNDNRIVQKITSIEPDYLICCKE--V-------------------

>Tad-gi|196011367|

-----DRQELAGRE--EDLSYLSEDEIFKIHMFFANFIRHLGDLLKLRQQVIATAIVYFKRFYSRNSLKSIAPLLLAPTCILLASKAEECGIINTGRFINACTNPYKMPVILECEFFLLELLDCSLIVFHPYRPLLQFVEDFEKKDALLPCAWRAINDSYNTDICLMYPPYIIALACLHTACIIQSIDTQWFAEL-----VDLDLLFEVIVAL--------------------

>Tad-gi|196007260|

-----YPDELQETP--SQLDGLSKEMETNLRIAGCEYIQAAGILLKLPQVAMATAQVLYQRYYYSKSFVKYNYEICAMACIFLAAKIEEHP-RRIRDVVNVFYHFHKKSEVIKAERRVLKELGFCVHVKHPHKCIVTYLKILECNDELIQKAWNYMNDSLRTDVFMRYTPETIACACIYLSARQLGIGPPWYEVF--------------------------------------

>Tad-gi|196000815|

-----KVVMPVVKI--SAQQLIEDKDHWPEYLKIGKFIAESGIKLKLGSVVIARAATIYHRFYFLCDISQFDRYLVAVTCLYLASKVEDTP-RRARDVITTSYKWQLRDSVVNFELFMLRMLKFDVSSELPHKYLLHYLKSLQDW-HINQLCWQLLQDTSLLPFILLYPPSVIATAVIYLAVKCNNIEKPWWNVFPN---LNEEGLQQLFMEL--------------------

>Tad-gi|196007480|

-----FCSNIVNSP--SRSDGIEITAECRYRREGARFIMDVGNRMNLRYETVATGIVFYHRFYMMHSFKTINRLIGAAACLYLAGKAEETP-KKCRDLVKAVRTDDPKEEIISHERLLLQTIKFDLCVQHPYKYIVKFAKNLKDDRKVVQMAWNFVNDSLSTTLCLQWKPQVVAVSLLHLAAKLSKYNKSWWQHFPE---INSDVLEDIMLDF--------------------

>Tad-gi|196012973|

-------------------------ITPTQREALISSMINLSRKHNIKRDTLFMAVNYLDRFLKVVTVTEDCFELVGLTCMMIACKVEECQPPKMEEFLTSCTHYYKKAEMKRLEIIILNYIDFRLSPPIAPHFLEYIIHFHQTYIELVNIANQVLLKILPTYRFNHIKSSILAASAFEY-----------------------------------------------------

>Tad-gi|195998946|

-----EEIAHKGAL--HNQFFQKLKDDDSRHIRTKFELMDLCRRF--DPPMPATAAVYMKRFYLVCSVMDYHPCDIMLACVYLATKVDEYN-ISIDKFLTMVPEERAKSRTLGFELLVMEKLKFHLTIHCPYRPVEGLLINIMVPAELRRHIDKFLSKILYCQAMLIYPPSQVGEIALFPS----------------------------------------------------

>Tad-gi|196013340|

-----FSDTLQNTP--SRRNDISVATELYYRQTCALCIQELGMKLGANQLTINTALVYMHRFYMFHSLASYNLKNIAACAIFLASKSEEHP-NKLNKVITAAY-LKLSQDLVDNEYAMFFTTGFDIEIMHPHTHVIKCLHGLK------------------------------------------------------------------------------------------

>Tad-gi|196013209|

SCSSIQPNLLCFICRMYPRDIFDEELHPLMRRHVYKFFKTLFSSAQLTAECAIISLIYVERLMEYAEIHPSNWRRVVLGAILLASKVWDDQAVWNIDYCQILKD-TTVEDMNALEREILQLIMFNINVPSSIY-AKYYFD------------------------------------------------PVI------------------------------------------

>Bfl-gi|260806975|

EIFQYLREALRHRPKPGYMK-KQPDITNSMRCILVDWLVEVAEEYKLHNETLYLAVSYIDRFLSSMSVLRSKLQLVGTAAMFLASKYEEIYPPDVGEFVYITDDTYTKKQVLRMEHLILKVLSFDVAVPTINCFQKRFLQAAKVNSKTESLAMYLAELTLQEETFLKYVPSTIAAASLCLAQHTLNM-QPWTPTLMHYSGYTLADLLPCVQDMRSF-Q-AAPSSQQQAVREKY

>Bfl-gi|260800992|

GLYGGVVRQSQPSYSHRIFE-IQKGMNNIMRFILVDWLVEVASMKDFSTQVLHAAVRCVDRYLMTHKTPRSKLQLVGVASMVLVTRYLAKDILTVREAVWLTDNTYKYEDVVRMMGELTATLRGEIRALTSADYLDLFFKLQLLDQRTKCLAAYICDLALLQTEMGTYSPAVIAASALLLAKMTTNA-ELWSSHMTQFTGLQVVDLLTCTLQVHKPPPRDHHNNPLTAVRQHY

>Bfl-gi|260789607|

RVLNNLLITDKYLPSTTYFA-VQDEVKPHMRQMVATWMYEVCEEQRCEDEVFPLAMNYLDRFLSQVPIRKNHLQLLGAVCMFIASKLKETIPLTAEKLVIYTDNSIRCQELMDWELLVLMRLKWDLSAITPCDFLEHILSRLPIERERSKHAQTFIALCCTEFKFAIYPPSMIAAGSVGAAVNGL-VGGIWAEQMQKITNIDMDCLRACQEQIQ-------------------

>Bfl-gi|260786520|

ASTLE---EIAKEAKPRKVN-QDAEIGPSERDEAVRWLATLSTKVQAYPETFAMSVSILDRFLNAVKARPKYLRCISVSCFFLAAKINEEAIPSAGELVRVSECGCTANELLRMERIILDKLGWNLKDVTALDFLHIYHALLTTYQPTRHLAHLKLQRCMACHQVLGFPGSVVSLGLLSLDLEMLI--PDWLAALQKMVKVQNESLIRCR-----------------------

>Bfl-gi|260786518|

TAVRR---ASYQPNLDMEDL-RDPVVSSTQRDDAVDRLRCLNRCLRFKHEAFALAVNILDRFLSIMKVRMRFLGCLTITCYFIAINMLEEELPSPVHLIRISQCRCTEADLFRMEGIVCQKLYHDFGAVTPLTLLQLYHGICAIL--TEHLERTKLEACLCRSPFTKFTAPVLAMSLLMCDLD-NN--PDLCSTLKVASQINEQHLVECQKK---------------------

>Bfl-gi|260825762|

-----DKQDLRDRQ--DDLTNFPDDEYQKVHIFYCGVIQAVGEQLKLRQQVIATATVYFKRFYSKYSFRTIDPLLMGPTCVFLASKVEEFGVISNSRLITACQTPYRINHVLECEFYLLEMMDCCLVVYHPYRPLTSYVQDMGQEDTVLPLAWRIVNDSYRTDVCLLYPPFMIALAALHMACVILQKDKHWFAEL--------------------------------------

>Bfl-gi|260798658|

ELWSPMLEKALYNRDHNYLN-RHPGLAPRMRAILLDWLIEVCEVYRLHRETFYLAQDFIDRFLSTRDLPKHRLQHIGITALFIAAKLEEIYPPKVTEFAYVTDGACTDEEILDMELVLLKALNWELSPMTVNSWLNVYLQLANLDDEEFYLVAQLVDLCMLDISSLQFSYAAIATAALYHNS-CR-------DICLRVSGFSWEEVAPCVQWM--------------------

>Bfl-gi|260803926|

-----YEKKLLCSP--SATAGVDYATECRYRREGARFIIDAGTALGLKPQTFATGVVYFHRFYMFHTFKDFNRYVTGACCLFLAGKVEETP-KKCRDIIKTARADDQQEEVMTLERILLQTIKFDLQVEHPYMYILKYAKSLKEDAKLVQMAWTFVNDSLCTTLCLQWEPQIIAIAIMHLAGRLTKFDKNWWDRFED---VSLELLEDIVLDLQSPPPSHQPGFPPPGHPPPN

>Bfl-gi|260813108|

-----LPSELDATP--SVNDGLEHETEGDLRILGCEYIQTAGVLLRLPQTAMATGQVLFQRFFYSKSLVKHNMEIVAMACVYLASKIEEAP-RRIRDTINVFHHINTKNQVIKAERRVLKELGFCVHVKHPHKLIVMYLQVLDCEKKLVQTAWNFMNDSLRTDVFVRFSPETIACACIFLAARQLKVPCHWYELF-----ASEDEVEEIILKI--------------------

>Bfl-gi|260817673|

DIHETLRTKAELPP---FKG-LSP--QLGLRRYLVDWLSIINEKQGVHCTALHLAVYLLDQFMDSYDIQESRMHLVALGCLLVACKFEEEEVPRIKKLNQYVREVYSEEEYLQMELTILKFFQWNISLPTPAHFLDYYMTEGVSQSYLEKYCHYFLEVSLQGYKLVRKTRT--------------------------------------------------------------

>Bfl-gi|260780863|

-----SSEDLRKLR--CQANQDIRQKHEASGKDCSNRLREFCVKFPMPKSVMASASSYFKRVYLQNSVMEHHPKIIMLTCVYMACKVEEFN-VSIMQFVGNIRAERAVDIILNNELQLLQLLNFHLTVHNPIRPLEGLLIDMKTHATLRRWAEEYLDKSFLSDACLLFPPSQVALAALWHSGNTVGMD---------------------------------------------

>Bfl-gi|260786596|

-----SSEDLRKLR--CQANQDIRQKHEASGKDCSNRLREFCVKFPMPKSVMASASSYFKRVYLQNSVMEHHPKIIMLTCVYMACKVEEFN-VSIMQFVGNIRAERAVDIILNNELQLLQLLNFHLTVHNPIRPLEGLLIDMKTHATLRRWAEEYLDKSFLSDACLLFPPSQVALAALWHSGNTVGMD---------------------------------------------

>Bfl-gi|260809954|

------------------------------HFRIVRFMMESGIKLRMTSVPMATAAIIYHRFFSICILQDYDPYLIGMTAISLASKVEEEH-LKIRDVINVCYRADLRQAMASCELLIMRVLGFNVTKELPHKYLLHYLKSLGDW-PIRDTAWAMLRDLYHGKVCLQHEAQHLAVAVLYFSLLCFGIETKWWKVFED---ITEEEIKNIIMDV--------------------

>Bfl-gi|260835834|

-----FTPQLMDTP--TRKCGVDADKELSYRQQAANLIQDMGQRLTVNQLCINTAIVYMHRFYMYHSFTKFHRNALAAACLFLAAKVEEQP-RKLEHVIRVAHVLQQAQDLVINESILLQTLGFEVAIDHPHTHVVKTTQLIRAPKDLAQTAYFMATNSLHTAFSLQYKPTVVACMCIHLACKWASWEKYWWEYVPN---VTLDLLDSLFLHI--------------------

>Bfl-gi|260807385|

WLHVQTTVFQHSTESSILYP-VTSFVRNRRGLTAVDWVVAVFQDVTV-----------------------------GVEAEEDAWKKEEVHPLCAEEIVSLCDGQYTRGQLRMLERKVLSTLGFHLTVPTSILFLEHLAEAGRYDHLTSCLARHLLVTSLQDYVICQHAPSSLALAALNLAASLVNN----------------------------------------------

>Bfl-gi|260820262|

CVAPQRSDVEEVPKSANSDVAEDHEEDPSNHPNGKVLDGLLVHHSSKKRNNHHINHVYLERLLTYAEIAPCNWKRIVLGAILLASKVWDDQAVWNVDYCQILRD-ITVEDMNALERQFLELLQFNINVPSSVY-AKYYFD-----------------LSADANELI-FPVEQL-------------REERL------------------------------------------

>Cin-gi|198428764|

EIFQHLREA------PNYMR-KQQDITVGMRAILVDWLVEVADEYKLHTETTHLAVNYIDRFLSHMAVLRGKLQLVGAAAMFIAAKFEEIYPPDVGEFVYITDDTYTKKQVLRMEHLILKVLNFDVAVPTSNQFLKRYLKSAGADKKTEFLAQFLCELALVEFDCTQYLPSMIAASSVCLASYTVSG-KIWDETMEHYMQYQLQDLAPCIKRLEIL-A-GASKNSLQALFEKY

>Cin-gi|198433631|

DIYHYMLYLSESPIRRNYFK--DTGFKPRVRCILVDWLVQVHHRFQLLQETLYLTIAILDRFLQVHPVPKVKLQLAGVTAMLLASKYEEMYAPEVSDFVYITDKAFTQAQILSMEILMLKTINFSLGRPLPLHFLRRNSKAGQVDATQHTLAKYLMELSLVDNDMCHVPPSQLAAGALCLSIKLLED-SEWTPTLEHYSTYTKEDLIPVVCHLKNL-KSAEKSSYQQAVKSKF

>Cin-gi|198423591|

TIFEYMRARQSFPINENYME-KQTEITPEMRSILVDWMVEVQENFELNHETLYLAVKLVDCYLQQVKIKKEKLQLIGATSLLIAAKFDERQAPYLDDFLYICDDAYNKQQMMQMERTLLKTIGFDINIPIAYRFLRRYAKCAKSSMEVLTLARYIMELSLQDISFIGKSASLMAASALWLAFKMKKN-FQWNDTLVYYSSHNEQDIIELAVQLHML---SSRDTKLKTIFTKY

>Cin-gi|198421112|

DILRYMVYSAKYQPRKDYLE-KQNEISSTMRVKLIDWLIEVQDEYKLQNETLHLAVAYVDRFLSEMSVSRPKLQLLGTTSMFLAAKFEEIYPPDADEFAYVTADTYARSEVLLMERLMLSQFKCTLAVPTTLQFLNIFHKKSNLSEDAKQLSFYLSELALLHDVYLQYSPSVRAAAAISLAVCTLRQ-NGIKSVLQRCMKDKLQKVFDCTHELARSHVVESNEKNRHFIFEKY

>Cin-gi|198428427|

-----YDKALKKTP--SFLDGVNPETEGRYRREGPRFIFDMGTRMGLHHDTIATGIVFFHRFYMFHSFKKFPRHITATCCLFLAGKVEETP-KKCKDLIKVARGNDPKEEVLTFEKVLLQTIKFDLTVEHPYKYMLQYAKKLKGDRKLVQMSWIFINDSFYTTLCLQWEPAIVAVAVMHLAGRLCKFEGRWWEQFDD---ISMELLEDIVLDQ--------------------

>Cin-gi|198425946|

DIHDTLKARNFMLR---LTG-SSK--LMYWRRYLVDWLALTCQKYRLNSNAQHLAVCLYDRFTDQFQLGVEDLQMLVLCCLLVASKFEEREIPKFKILMDHLQWNLNAAEYMTMEIRLLSAFEWDIGFPTASHFKEYYMQVALGTRYLEKNVSYFLEVSLQDQAFLVFKPSLITASCVAASRICLHA-PTWTVELHKVTNFAWHHLVPCIEILS-------------------

>Cin-gi|198417861|

QLWKSMVNKTVYCRNSSYMD-RHADLQPRMRSILIDWIMEVCEVYSLHRETFYLAVDYIDRYLSAKNIHKTRLQLVGVTALFIAAKLEEIYPPKLSDFAYVTDGACTDDEILSQELIMLTALKWSLSPITAISWLNVYLQTAHAASASFFLISQLLDLCVLDIESLEFSSGLLAASALYH-S-SR-------ELATQVSGFHFKDLAACVHWM--------------------

>Cin-gi|198417413|

RVLRNLLELDRYLVSTSYFC-VQKDVQPYMRKVVATWMMQVCEEQKCEDDVFPLSMNYLDRFLSVHPIHRTQLQSLGSACMLIASKVKETLPLTTEKLVVYTDHSVGQDELLKFELLLLMRLKWDVLSITPIDFVDQILHRLHMDESTVKHAHTFIHLCCTDHTFSVYTPSMVAAGSVGAAVVGLQ-SRIWTEKLHEITGVDLDILRECLAQTL-------------------

>Cin-gi|198429958|

-----YSRELDQTP--SRKDGIDADKELGYRQQAANLIQDMGQRLSVNQLTINTAIVYMHRFYVYHSFTVFSRYAIAPTALFLAAKVEEQP-KKLEHVLKICYVLKQAQELVQNELVLLQTLGFDISVDHPHTHVVKCTQLVKASRDLSQMAYFMATNSLHTTFCLLYKPTVVAAMCIHLSCKWSKYEKAYWTYMPI---ITEPLLDTIFLKQKLKQKAAAVALSTEEKNLLG

>Cin-gi|198429948|

DVYWQMKAKYYSHP-STSLC-EQKIITQRCRFILVEWLVMVTHHYKIQGETLHLAVNILDRYLERETIRKKYLQLIGITSLLIAAKQLEVEIPPVSACLGLCRNLYTRPQLLSLERVLLITIGFDLNVPSSHLFYDTIIVVLVYFIALLCLGRSFLEKGLTNYQFVQFPPSVAARAAFRLSTSILDE-PSWPEE---LLEYEEENVLSC------------------------

>Cin-gi|198414425|

-----FPTEIEHTP--SSSDGLNASTEEDLRLLGCEYIQEAGIMLKVPQVAMANAQVLFQRFFFAKSFVKNKMEEVAMACIWLASKVEEAP-RRVRDVINVFHYIMLKNNVIKSERRLLKELGFCVHVKHPHKIIVVYLQVLEMEKDLVQTAWNYMNDSLRTTVFVRYTPETIACACIYMAARVLQVPPHWFCLF-----ATEEDIQQILMRL--------------------

>Cin-gi|198414966|

-----SPEFVRERE--LDLQVMGEVDYQKLMMFFANVIQSIGEQLKLRQQVIATATVYFKRFYSKHSLNSCDPLLLAPTCIFLSSKVEEFGVISNSRLISVVTTNYRIHHVWECEFYLLELMDCCLVVFHPYRPLVQYVNALGMADALLPIAWRIANDSLRTDVILLYPPFQIALACLHMACVVQNAAFQWFADL-----VDMEKIIEIILKL--------------------

>Cin-gi|198438473|

--------------------------DQKTHIEVVKFIVKCSIKLSLQDAVQASSSILYHRFFKHCSVEEYDPYTIAATAICLATKVEEQH-TRLRDIVNVCHRWNLRDTIASCELLMLRVLKFNVTCIHPHKYLLHYLMSLSHL-MVSDVAWALLNDSYISNTCLNHGPEIYAISVIDLALQSCKIKKKWWQVFLM---VQRDIAHTINSIA--------------------

>Cin-gi|198427565|

-----LIKLNNANT--KYRTSYCDVNNHVTPDADTRKLQEICWKFPMPLNVVGTSCMYLKRLNLRKSVMDYHPRLMHLACIWLACKTEEFN-ISMDQFVQQVAHEEIGDAILTIELILIQELNFHLTIHNPFRPLEGFLIDLKTYLQLRKPAKDFLVRSLNTDVGLLYAPSQLALAALLSAASMRNLI---------------------------------------------

>Dme-cycA-gi|24662962|

DILEYFRESKKHRPKPLYMR-RQKDISHNMRSILIDWLVEVSEEYKLDTETLYLSVFYLDRFLSQMAVVRSKLQLVGTAAMYIAAKYEEIYPPEVGEFVFLTDDSYTKAQVLRMEQVILKILSFDLCTPTAYVFINTYAVLCDMPEKLKYMTLYISELSLMEETYLQYLPSLMSSASVALARHILGM-EMWTPRLEEITTYKLEDLKTVVLHLHTH-K-TAKELNTQAMREKY

>Dme-cycB3-gi|24649824|

DIFNYLKVRAEFPI-ADYMP-RQIHLTTWMRTLLVDWMVEVQETFELNHETLYLAVKIVDLYLCREVINKEKLQLLGAAAFFIACKYDERQPPLIEDFLYICDGAYNHDELVRMERETLRVIKYDLGIPLSYRFLRRYARCAKVPMPTLTLARYILELSLMDYANISFSDSQMASAALFMALRMHDK-QTWTSTLIYYTGYQLADFAEIVTALAGL-H-RKPRATIKTIRNKY

>Dme-cycB-gi|24658567|

DIYDYLYQVLEQPIHKDHLA-GQKEVSHKMRAVLIDWINEVHLQFHLAAETFQLAVAIIDRYLQVVKTKRTYLQLVGVTALFIATKYEELFPPAIGDFVFITDDTYTARQIRQMELQIFKAIDCNLSRPLPIHFLRRYSKAAGAEDEHHTMSKYFIELASVDYEMATYRPSEIAAASLFLSLHLLND-RHWTPTLTFYSRYSAAHLRPITRLIKL--ARDAPQAKLKAIYNKY

>Dme-cyc-gi|17136874|

DVWRLMCHRDSRLRSISMLE-QHPGLQPRMRAILLDWLIEVCEVYKLHRETFYLAVDYLDRYLHVHKVQKTHLQLIGITCLFVAAKVEEIYPPKIGEFAYVTDGACTERDILNHEKILLQALDWDISPITITGWLGVYMQLNVGRADAFIYTSQLLDLCTLDVGMANYSYSVLAAAAISHTF-SR-------EMALRCSGLDWQVIQPCARWM--------------------

>Dme-gi|21357017|

-----SMQQVELNK--AQTMKPIDYRKMNKPGVVPMYIFECAAKLKMKPLTAACAAIVFHRFFREVKASDYDEFLIAAGSLYLAGKIKEDESVKIRDVINVAYCWSMRDAIVQAELLITRTLCFDLNIDLAHKYLLHYMKTLQDW-PIAKAAASYLQDFHHSANILKYKPTHVAIGCLSLALQTYGIQAMWYKPLKD---FTRENQWEIVIEV--------------------

>Dme-gi|18921115|

-----IPEGIDVTP--SSQDGLDHETEKDLRILGCELIQTAGILLRLPQVAMATGQVLFQRFFYSKSFVRHNMETVAMSCVCLASKIEEAP-RRIRDVINVFHHTNLKMQVIKAERRVLKELGFCVHVKHPHKLIVMYLQVLQYEKKLMQLSWNFMNDSLRTDVFMRYTPEAIACACIYLSARKLNIPPPWFGIF-----VPMADITDIVMEL--------------------

>Dme-cycC-gi|17136688|

-----DKPDLRERQ--HDLLALNEDEYQKVFIFFANVIQVLGEQLKLRQQVIATATVYFKRFYARNSLKNIDPLLLAPTCILLASKVEEFGVISNSRLISICQSPYRTNHILECEFYLLENLDCCLIVYQPYRPLLQLVQDMGQEDQLLTLSWRIVNDSLRTDVCLLYPPYQIAIACLQIACVILQDAKQWFAEL-----VDLDKVQEIIVNL--------------------

>Dme-cycT-gi|24666004|

-----FSNDLANSP--SRRCGIKGDDELQYRQMTAYLIQEMGQRLQVSQLCINTAIVYMHRFYAFHSFTHFHRNSMASASLFLAAKVEEQP-RKLEHVIRAANKAELAQELVFNENVLLQTLGFDVAIDHPHTHVVRTCQLVKACKDLAQTSYFLASNSLHTSMCLQYRPTVVACFCIYLACKWSRWEKHWFYYVKT---VSLDLLKQLFIAITPNGIETTPDLVRSLLKESL

>Dme-cycD-gi|281360953|

RCLENFLKVEHHKIPDTYFS-IQKDITPPMRKIVAEWMMEVCAEENCQEEVVLLALNYMDRFLSSKSVRKTQLQILAAACLLLASKLREPSALSVDLLVVYTDNSIYKDDLIKWELYVLSRLGWDLSSVTPLDFLELLMMRLPIGSKNIGHAQAFISLAAKEHKFAKFSASTIAASSIAASMNGLK----WHSLMTDLTSVEQAQVRDCMLPLC-------------------

>Dme-cycJ-gi|24656859|

DIFLTMREQLSRRP---LFY-LSP--QLNERRRMLQLLKLATSAHKLSRCALHLAVYYMDRFVDYYKIRPDKLLLVAITCLHIAAQIENTDIPRYSEMNRLVKNAYTAFEYKAVERKILCFLNFELIRPTTASFVELFACSFYERHILAQLLLRMADYTLYISRFANDLPSLLAAACIAAVRQVSGV-RRWSEYLVGLTSYTEANVEPYMNVLS-------------------

>Dme-cycK-gi|28574303|

-----YDKKLRETP--SILDGISFETERRYRKEGARFIMECGTKMGLGHNTMATGVVYFHRFYMFHSFRSFPRYVTACCCLFFAGKVEETP-KKCRDIIKTARGDDPKEEVMTLERILLQTIKFDLQVEHPYTFLLKYAKCFKGDQKMVQMAWNFVNDSLSTVVCLQWEPEIIAVALIHLASKLSKFTQRWWDMFSD---VTMEILEDIVLDL--------------------

>Dme-cycG-gi|24651671|

ELYET---LEYDVLFHTPKE-SRREVTAGGRDGSAYVLRCLKMWYELPSDVLFSAMSLVDRFLDRMAVKPKHMACMSVASFHLAIKQLDLKPIPAEDLVTISQCGCTAGDLERMAGVIANKLGVQMGHITSVSYLRIYYAL--LAKEQIKLEELRLEILMCDVKTTVITPSTLALVLICLHL---------------------------------------------------

>Dme-cycH-gi|17737725|

-----GQLMFRVEQ--NSKYIESHEEEAQGRDLNEIYLFDFCRRFTMPKCVVGTAFHYFKRFYLNNSPMDYHPKEILATCVFVACKVEEFN-VSINQFVNNIKGNKATDIVLSNELLLIGQLNYYLTIHNPFRPIEGFLIDIKTRMRLRPHIDSFIDSTYYSDACLLHTPSQIALAAVLHAASRE--E---------------------------------------------

>Dme-cycY-gi|19921156|

STIYLNLKNCSLAIIKNRRIFDEKLHPLTHDRQIYKFVRTLFNAAQLTAECAIITLVYLERLLTYAEVGPCNWKRMVLGAILLASKVWDDQAVWNVDYCQILKD-ITVEDMNELERQFLELLQFNINVPSSVY-AKYYFV------------------------------------------------KRR------------------------------------------

>Spu-gi|47550945|

EIYQYLKTASKHRPKHGYMR-KQPDITNSMRCILVDWLVEVSEEYRLHNETLYLAAAFIDRFLSQMSVLRAKLQLVGTASMFVASKYEEIYPPDVKEFVYITDDTYSIKQVLRMEHLILKVLSFDLAAPTINSFLPRFIKAAKANSKTEHLTQYLAELTLQEYDFIKYAPSMIAASAVCLANHTLNN-EEWTPTMAHYTDYQLGDIYPCVQDLQLF-I-KAPTMEQQAVREKY

>Spu-gi|75677617|

EIYLYMRTLNQMKVPAGYLD-REGQVTGRMRHILVDWLVQVHLRFHLLQETLFLTVQLIDRFLVDHAVSKGKLQLVGVTAMFIASKYEEMYPPEINDFVYITDQAYTKTQIRQMEVFMLKGLKYSLGKPLCLHFLRRNSKAAGVDPQKHTLAKYLMEITLPEYSMVQYDPSEIAAAAIYLSMALLGE-DNWGAKMTHYSMYSEDHIKPIIQKMTAVTREDAMSEKYHAVKTKY

>Spu-gi|72065482|

GIFRYLKEKESSKI-DDYFD-MQKDITRHMRSVLVDWLVEVQENFELNHETLYLAVKLTDMYLAKCKIAKDLLQLLGATSLFIACKFDERIPPALDDFLYICDDAYSRNQFTDMERKVLKMVNFALGVPLSYRFLRRYAKCAHATLETLTLARFILELSLMESSFITVADSLIAASALLLAFRMKNN-GTWDVTLRHYSGYVEEDLKQCMNQLSML-N-SPPNKQLATVRNKY

>Spu-gi|115707311|

GKLGGAMRGEQPTKVHTLLR-SKNGLNSAMRYILVDWLVEVGSMKDYSCLTIHSAVQLVDRYLMARNISRSTLQLVGITCMVICSRLLEDDIITIREAAWLTDGTYKYEDVVRMLGDVVATLKGNLRELTILDYLHLFCQVVSADSKMEYLALYISELSLLHADFGQYSRALIAACSLFLARLVLGD-FPWPTPLVEHTGFEICDLVQCTLHLNKDPVKDHRDVTLAAVKQRF

>Spu-gi|72015188|

EVWTIMTRKALCPRKHDCLK-SHPSLGERMRAILLDWLIEVCEVYRLHRESFYLAADFVDRYLAAENVPKTKLQLIGITSLFVAAKLEEIYPPKLHEFAYVTDGACTDDQILDQELIMLMTLNWDLTPITVNTWLNAFMQICNAERTNFHFVAQLLDVCTLDIGSMDFDYSILAASALYHVT-NE-------EVTLSVTGLKWDDIAACVQWM--------------------

>Spu-gi|115727280|

EVYASLCHESRILP---FQG-KSP--QLNLRRFLVDWLAIVSENLDIESPARHLAVYLLDRTMDRFTVSEAYLQRLALVCLLIATKFEEKEVVKLTNLANQNTDETAKKEFFQMELLLLDFFDWNISVPTSLHFVDYFLMDAVG-PYLERYAQYFLEISLQA------------AICIAASRICLQS-PTWTNQLKKLTKYSWGQISPFIETMK-------------------

>Spu-gi|115926275|

-----YEKELEHTP--SIKDGIDPGTEARYRREGSRFIIEAGTTQKLRYDTMATGVVYFHRFYMFHSFKEFPRYIMGAACLFLAGKVEETP-KKCKDIIKIAKNDDPKEEIMTHERILLQTIKFDLQVEHPYSYLLKYAKTFKGDKKLVQMAWTFVNDSLCTRLCLQWEPHIVAVGFLYLAGRLSKSDSKWWEQLED---ISLDIMEEILLDLNPPAYTHPNPPVSTYAAPQY

>Spu-gi|115712073|

NSRLC---SINKERKPLPQD-SETEIGPNQRDEMVQWLLELNVKFRFCPETYMLSVTLLDQCLMAVKARPKYLRCITITCFFLAAKMKEEMVPATHDFVRDSQCGCTVSEVLRMERVVLDKLKWELNFVNGLDFLQIFHALLMTQRPTRHLSMLRLSQCMTNHQLAACRGSTLALAMLSLELEVLA--EDW--------------LPLTT-----------------------

>Spu-gi|72089181|

KMMQV---AETEDFYLEQQE-DSDCILPYMHDVILDRLRSLSRFFQLCPETFFLAVNTMDRFLSLVKARPHHLMCIAISSYNLAIKALEPSLISAEDLVRISQCGCSVNDVLRMERIILQKLQCDLQAPTAHRFFKLFHAYSVIL--MTQLESSKLEACLCYFPLTLYKPSVLALALLTHEL--ND--IRWMRIIQRVSQVSDNDLVSCRSQV--------------------

>Spu-gi|47550981|

KNLDNLLAVEQYVLSADYFH-FQRELRPSMRKLVVDWMFEVCEEQQREEDVFPLSVNYLDRFLSIERISRDKFQLLGATCMFLASKLLETIPLTSEKLIIYTDNSITLEQLLKFEQLVLTKLKWDLMAITPNAFLEHIFHRLPVDKEQAKHAQTFIVLCATDYNFAMQPPSLIAASGVAAAANGLRMH----LLHRITKIETDYLILVRDRSLK-------------------

>Spu-gi|115620254|

-----IPDALSQTP--SANDGLETETEMDLRILGCEFIQMAGILLKLPQVAMATGQVLFQRFYYSKSLVKHNMETVAMGCINLASKIEEAP-RRLRDVINVFHHINLKNQVIKAERRLLKELGFCVHVKHPHKMIVTYIQALECESQLVRTAWNYMNDSLRTDVFVRYTPETVACACISLSARQIGLPPPWYGLM-----ATDEQVEDIILRL--------------------

>Spu-gi|72137687|

-----------DGG--EVFNIAREDLDKKTHFKVIHYVMEAGIKLHLESVTLASACCIYHRFFAECELNNYDPYLIGATAIYLATKVEEQH-VKLRDIINVCYRWELRDSLVNCELLLVRMLKYNPKIGLPHKYLVHYLKSLSHW-PVCRTAWAMLRDSYHSDIALRTKPQHMAVAVMYFSLQCYGLENPWWKAFED---ISEEIIQKILIEL--------------------

>Spu-gi|115841184|

-----EELNKRAAS--NRRYREEHQAKSEGKDPATFLLRAFCKKFPVPPAVVGTSCAYFKRFYIYNTAMDYHPKYIMLTCVYLACKVEEFN-VSISQFCGNLQPEKMAELILSHELLVMQQLNYQLTIHNPYRPMEGLFIDIKRFMLLRKGAEEFINRSLATNACLLCSPSQIALAALVSSSA--------------------------------------------------

>Spu-gi390364883|

STIFLNLKSCALAIIKNRQIFDEKQYPLSKTRHIYKFIRMLFNAAQLTAECAIVTLVYLERLLTYAEITPSNWKRMVLGAILLASKVWDDQAVWNVDYCQILRE-LTVEDMNELERQFLELLQFNINVSASVY-AKYYSK------------------------------------------------KRF------------------------------------------

1. **Multiple alignments for phylogenetic analysis of cyclin family proteins in H. sapiens, *T.* adhaerens, *C.owczarzaki ,* and *S. arctica.***

>Hsa-CyclinA1

EIYQYLREAEIRHRPKAHYMK-KQPDITEGMRTILVDWLVEVGEEYKLRAETLYLAVNFLDRFLSCMSVLRGKLQLVGTAAMLLASKYEEIYPPEVDEFVYITDDTYTKRQLLKMEHLLLKVLAFDLTVPTTNQFLLQYLRRQGVCVRTENLAKYVAELSLLEDPFLKYLPSLIAAAAFCLANYTVNKHFWPETLAAFTGYSLSEIVPCLSELHKALDIPHRPQQAIREKY

>Hsa-CyclinA2

DIHTYLREMEVKCKPKVGYMK-KQPDITNSMRAILVDWLVEVGEEYKLQNETLHLAVNYIDRFLSSMSVLRGKLQLVGTAAMLLASKFEEIYPPEVAEFVYITDDTYTKKQVLRMEHLVLKVLTFDLAAPTVNQFLTQYFLQQPANCKVESLAMFLGELSLIDDPYLKYLPSVIAGAAFHLALYTVTGQSWPESLIRKTGYTLESLKPCLMDLHQTLKAPQHAQQSIREKY

>Hsa-CyclinB2

DIYQYLRQLEVLQSINPHFLD--GRDINGRMRAILVDWLVQVHSKFRLLQETLYMCVGIMDRFLQVQPVSRKKLQLVGITALLLASKYEEMFSPNIEDFVYITDNAYTSSQIREMETLILKELKFELGRPLPLHFLRRASKAGEVDVEQHTLAKYLMELTLIDYDMVHYHPSKVAAAASCLSQKVLGQGKWNLKQQYYTGYTENEVLEVMQHMAKNVNENLTKFIAIKNKY

>Hsa-CyclinB1

DIYAYLRQLEEEQAVRPKYLL--GREVTGNMRAILIDWLVQVQMKFRLLQETMYMTVSIIDRFMQNNCVPKKMLQLVGVTAMFIASKYEEMYPPEIGDFAFVTDNTYTKHQIRQMEMKILRALNFGLGRPLPLHFLRRASKIGEVDVEQHTLAKYLMELTMLDYDMVHFPPSQIAAGAFCLALKILDNGEWTPTLQHYLSYTEESLLPVMQHLAKNVNQGLTKHMTVKNKY

>Hsa-CyclinD3

RVLQSLLRLEERYVPRASYFQCVQREIKPHMRKMLAYWMLEVCEEQRCEEEVFPLAMNYLDRYLSCVPTRKAQLQLLGAVCMLLASKLRETTPLTIEKLCIYTDHAVSPRQLRDWEVLVLGKLKWDLAAVIAHDFLAFILHRLSLPRDVKKHAQTFLALCATDYTFAMYPPSMIATGSIGAAVQGLG--ELTELLAGITGTEVDCLRACQEQIEA----------------

>Hsa-CyclinB3

EIFSYMKEREEQFIL-TDYMN-RQIEITSDMRAILVDWLVEVQVSFEMTHETLYLAVKLVDLYLMKAVCKKDKLQLLGATAFMIAAKFEEHNSPRVDDFVYICDDNYQRSEVLSMEINILNVLKCDINIPIAYHFLRRYARCIHTNMKTLTLSRYICEMTLQEYHYVQEKASKLAAASLLLALYMKKLGYWVPFLEHYSGYSISELHPLVRQLNKLTFSSYDSLKAVYYKY

>Hsa-CyclinD2

RVLQNLLTIEERYLPQCSYFKCVQKDIQPYMRRMVATWMLEVCEEQKCEEEVFPLAMNYLDRFLAGVPTPKSHLQLLGAVCMFLASKLKETSPLTAEKLCIYTDNSIKPQELLEWELVVLGKLKWNLAAVTPHDFIEHILRKLPQQREIRKHAQTFIALCATDFKFAMYPPSMIATGSVGAAICGLQQDALTELLAKITNTDVDCLKACQEQI------------------

>Hsa-CyclinE1

EVWKIMLNKEKTYLRDQHFLE-QHPLLQPKMRAILLDWLMEVCEVYKLHRETFYLAQDFFDRYMATQNVVKTLLQLIGISSLFIAAKLEEIYPPKLHQFAYVTDGACSGDEILTMELMIMKALKWRLSPLTIVSWLNVYMQVAYLNDQIFIQIAELLDLCVLDVDCLEFPYGILAASALYHF---------SSLMQKVSGYQWCDIENCVKWLD-----------------

>Hsa-CyclinE2

EVWLNMLKKESRYVHDKHFEV-LHSDLEPQMRSILLDWLLEVCEVYTLHRETFYLAQDFFDRFMLTQDINKNMLQLIGITSLFIASKLEEIYAPKLQEFAYVTDGACSEEDILRMELIILKALKWELCPVTIISWLNLFLQVDALKDETFIQIAQLLDLCILAIDSLEFQYRILTAAALCH-F--------TSVVKKASGLEWDSISECVDWLA-----------------

>Hsa-CyclinK

KPCWYWDKKDL-AH--PSQLEGLDPATEARYRREGARFIFDVGTRLGLHYDTLATGIIYFHRFYMFHSFKQFPRYVTGACCLFLAGKVEETP-KKCKDIIKTARGDDPKEEVMVLERILLQTIKFDLQVEHPYQFLLKYAKQLKGDKKLVQMAWTFVNDSLCTTLSLQWEPEIIAVAVMYLAGRLCKFRRWWEQF----VQPVDVLEDICHQILDQQQPAQQQQPAQQPKK

>Hsa-CyclinJ

DIHQALRYKELK----PSYKG-QSPQ--LSLRRYFADLIAIVSNRFTLCPSARHLAVYLLDLFMDRYDISIQQLHLVALSCLLLASKFEEKSVPKLEQLNTNMNLVLTKQNLLHMELLLLETFQWNLCLPTAAHFIEYYLSEAVHETYMAKYADYFLEVSLQDYAFLNYAPSLVAAACVASSRIILRSPTWPTRLHRLTAYSWDFLVQCIERLL-----------------

>Hsa-CyclinF

EIVCQLFQ---ASQASQQVFS-VQKGLNDTMRYILIDWLVEVATMKDFTSLCLHLTVECVDRYLRRRLVPRYRLQLLGIACMVICTRFISKEILTIREAVWLTDNTYKYEDLVRMMGEIVSALEGKIRVPTVVDYKEVLLTLVPVELRTQHLCSFLCELSLLHTSLSAYAPARLAAAALLLARLTHGTQPWTTQLWDLTGFSYEDLIPCVLSLHKRKQRFEDKRYGEISQE

>Hsa-CyclinO

SCYAFRKAQESHFHP-REALA-RQPQVTAESRCKLLSWLIPVHRQFGLSFESLCLTVNTLDRFLTTTPVAADCFQLLGVTSLLIACKQVEVHPPRVKQLLALCCGAFSRQQLCNLECIVLHKLHFTLGAPTISFFLEHFTHARVEALEAQALARGVAELSLADYAFTSYSPSLLAICCLALADRMLRVS--RPVDLRLGDHPEAALEDCMGKL--------QLLVAINSTS

>Hsa-CyclinD1

RVLRAMLKAEETCAPSVSYFKCVQKEVLPSMRKIVATWMLEVCEEQKCEEEVFPLAMNYLDRFLSLEPVKKSRLQLLGATCMFVASKMKETIPLTAEKLCIYTDNSIRPEELLQMELLLVNKLKWNLAAMTPHDFIEHFLSKMPEAEEIRKHAQTFVALCATDVKFISNPPSMVAAGSVVAAVQGLNLRRLTRFLSRVIKCDPDCLRACQEQI-E----------------

>Hsa-CyclinT2

SSRWFFTREQL-EN--PSRRCGVEADKELSCRQQAANLIQEMGQRLNVSQLTINTAIVYMHRFYMHHSFTKFNKNIISSTALFLAAKVEEQA-RKLEHVIKVAHYLQQTQELVILETIMLQTLGFEITIEHPHTDVVKCTQLVRASKDLAQTSYFMATNSLHTTFCLQYKPTVIACVCIHLACKWSNWKHWWEYV----DPTLELLDELTHEFLQLSGLHHRPDKISDHSS

>Hsa-CyclinL2

LENCLLPDDKL-RF--PSMSSGLDTDTETDLRVVGCELIQAAGILLRLPQVAMATGQVLFQRFFYTKSFVKHSMEHVSMACVHLASKIEEAP-RRIRDVINVFHYVNLKNQIIKAERRVLKELGFCVHVKHPHKIIVMYLQVLECERHLVQTSWNYMNDSLRTDVFVRFQPESIACACIYLAARTLEIPHWFLLF----GATEEEIQEICLKILQ----------------

>Hsa-CyclinG2

GLLNVYLEQEERFQLIEATP--NDNTLCPGLRNAKVEDLRSLANFFGSCTETFVLAVNILDRFLALMKVKPKHLSCIGVCSFLLAARIVEENIPSTHDVIRISQCKCTASDIKRMEKIISEKLHYELEATTALNFLHLYHTIILC-HEILSLDKLQLKACNCRLIFSKAKPSVLALCLLNLEVE-------------------------C---------------------

>Hsa-CyclinT1

NKRWYFTREQL-EN--PSRRFGVDPDKELSYRQQAANLLQDMGQRLNVSQLTINTAIVYMHRFYMIQSFTQFPGNSVAPAALFLAAKVEEQP-KKLEHVIKVAHYLQQVQDLVILESIILQTLGFELTIDHPHTHVVKCTQLVRASKDLAQTSYFMATNSLHTTFSLQYTPPVVACVCIHLACKWSNWKHWWEYV----DATLELLDELTHEFLQVKADKTALKMRIPVAG

>Hsa-CyclinL1

IDHSLIPEERL-SP--PSMQDGLDLPSETDLRILGCELIQAAGILLRLPQVAMATGQVLFHRFFYSKSFVKHSFEIVAMACINLASKIEEAP-RRIRDVINVFHYINTKNQVIKAERRVLKELGFCVHVKHPHKIIVMYLQVLECERTLVQTAWNYMNDSLRTNVFVRFQPETIACACIYLAARALQIPHWFLLF----GTTEEEIQEICIETLR----------------

>Hsa-CABLES1

VIDYVKP-------DNTFK---EFPHILSKIRSLKREMRKLAQEDCGLEEPTVAMAFVYFEKLALKGKLNKQNRKLCAGACVLLAAKIGSDLKHEVKHLIDKLEERLNRRELIAFEFPVLVALEFALHLPEH-EVMPHYRR------------------------------------------------------------------------------------------

>Hsa-CyclinC

YLQWILDKQDLLKE--QKDLKFLSEEEYWKLQIFFTNVIQALGEHLKLRQQVIATATVYFKRFYARYSLKSIDPVLMAPTCVFLASKVEEFG-VSNTRLIAAATFPYRMNHILECEFYLLELMDCCLIVYHPYRPLLQYVQDMGQEDMLLPLAWRIVNDTYRTDLCLLYPPFMIALACLHVACVVQQ-RQWFAEL----S-DMEKILEIIRVILK----------------

>Hsa-CyclinG1

HQLNALLEQESRCQLIESAH--NGLRMTARLRDFEVKDLLSLTQFFGFDTETFSLAVNLLDRFLSKMKVQPKHLGCVGLSCFYLAVKSIEENVPLATDLIRISQYRFTVSDLMRMEKIVLEKVCWKVKATTAFQFLQLYYSLLQENLNSINFERLQLKACHCRIIFSKAKPSVLALSIIALEIQ-------------------------L---------------------

>Hsa-CyclinH

NRKFRCKAVANGKV--PNDPVFLEPHEEMTLCKYYEKRLLEFCSVFAMPRSVVGTACMYFKRFYLNNSVMEYHPRIIMLTCAFLACKVDEFN-VSSPQFVGNLRLEKALEQILEYELLLIQQLNFHLIVHNPYRPFEGFLIDLKT--ILRKTADDFLNRIALTDAYLLYTPSQIALTAILSSASRAGI-------------------------------------------

>Hsa-CyclinI

ENQRLSFLLEKAITVNVRKM--SNQNVSPSQRDEVIQWLAKLKYQFNLYPETFALASSLLDRFLATVKAHPKYLSCIAISCFFLAAKTVEERIPVLKVLARDSFCGCSSSEILRMERIILDKLNWDLHTATPLDFLHIFHAIAVSFSPSQHLAVLQLLHCMACNQLLQFRGSMLALAMVSLEMEKL---DWLLELLQKAQMDSSQLIHCD---------------------

>Hsa-Fam58A

-------PEGGGGG--AARGPEGQPAPEARVHFRVARFIMEAGVKLGMRSIPIATACTIYHKFFCETNLDAYDPYLIAMSSIYLAGKVEEQH-LRTRDIINVSNFWELRDSIVQCELLMLRVLRFQVSFQHPHKYLLHYLVSLQNRTPVAVTAWALLRDSYHGALCLRFQAQHIAVAVLYLALQVYGVKPWWQVF----NDTKPIIDNIVSDLIQ----------------

>Hsa-CABLES2

TVIEYVK-------KMETF---RKFPHTLSKIRSLKREMRSLSEECSLEPVTVAMAYVYFEKLVLQGKLSKQNRKLCAGACVLLAAKISSDLKSGVTQLIDKLEERFNRRDLIGFEFTVLVALELALYLPEN-QVLPHYRR------------------------------------------------------------------------------------------

>Hsa-Fam58B

---MEDAGEEAGED--GEDAREGAAAPAARVHFRVARFIMEAGVKLGMQSIPIATACTIYPKFFCETILDAFDPYLIAMSSIYLAGKVEEQP-LWAHDIISVSNLWELRDSIVQRELLMLRVLRFQVSFQHPHKYLLYYLVSLKNRTPVAVTAWALLRDSYHGGLCLRFQAQHIAVVVLYLALQVYGVKLWWQAF----SDTKPIIDTIVSDLIQ----------------

>Hsa-CyclinI2

EGDLDERRLLCHLQRLWRGG--PQDEICDAFEE-VVLWLLRLQNTFYFSQSTFNLALTIFGRLLISVKVKEKYLHCATITSLRLAAKVNEEFIPQVKDFTKHYGSDYSPNELLRMELAILDRLHWDLYIGTPLDFLTIFHALVVLELPSLHVASLQLQHCMAGHQLLQFKGSTLALVIITLELERL---GW----------------------------------------

>Hsa-CyclinJL

DVHCTLREKELK----PTFRA-HSPL--LKSRRFFVDILTLLSSHCQLCPAARHLAVYLLDHFMDRYNVTSKQLYTVAVSCLLLANGFEDRHVPKLEQINSSQNFTLTKKELLSTELLLLEAFSWNLCLPTPAHFLDYYLLASVSKDCLKEYAHYFLEVTLQDHIFYKFQPSVVAAACVGASRICLQSPYWTRDLQRISSYSLEHLSTCIEILLV----------------

>Hsa-CyclinYL

AVVEPAELDFGEGESRMPEDALSPSDHKEQREKFIYRFVRTLFSAAQLTAECAIVTLVYLERLLTYADICPTNWKRIVLGAILLASKVWDDQAVWNVDYCQILK-DITVEDMNEMERHFLELLQFNINVPASVY-AKYYF-----------------DLRLADDNNLNFSK------------------------------------------------------------

>Hsa-CyclinY

VREKRKSLFINHHPYSSTIFDDTSQPHPLSKSKQIYRFVRTLFSAAQLTAECAIVTLVYLERLLTYADICPANWKRIVLGAILLASKVWDDQAVWNVDYCQILK-DITVEDMNELERQFLELLQFNINVPSSVY-AKYYF-----------------DLREANNLSFPLSP------------------------------------------------------------

>Tad-gi|196003740|

IIFENMKQREAQLVV-NDYLE-RQNDITEQMRMILIDWLCEVQQNFELFHETLYLAVKIVDRFLSARVVSRDALQLIGATAMLMSSKIEERYPPLVDDFVYICDDAYSRQAVLDMERDICYALDFDLNIPIPYRFLRRYGKVASLSMENLTLARYILELTLQEYQFVTFKPSMLAAGCLCLALKMKNCGEWTQTLVHYSGYEESELNELVQKLNAMAKPAPENCKVVKTKY

>Tad-gi|196005765|

DIHSYLRKAEYFHRPKYDYME-RQCDVNGTMRSILVDWLVEVSEEYKLRERTLYLAISYIDRFLSAMSVRRSKLQLVGTAALFIAAKFQEIYPPDCAEFAYITDDTYNIKQVLKMESLMLKVLSFNLSSPTAVDFLERYGSEAGLDSEIRELSMYLTELTLKDYGFLQFMPSLIAVSAVSLALHTFKLKYWPQELSTYTNYQWQQVSPCLNRIFEARLAHTQPQRAVVEKY

>Tad-gi|196002535|

DIYKNMLKQEKRCTLDPDYMT-GQPVITKGMRAILLDWLVDVHLRYNFHPESLYLTTYIIDRYLQTTQVNRKKLQLVGIAAFYIAIKYEEIFLASTDDLLYLTENSYEINEFIQMEAKILKALDFSLSRPTSIHFLRRISKAASADIEQHTFARYLTEIALIEYSLLSYLPSQIAAAASLISLKIFD-KSWTPTLQYYSSYSEDSLKPVARQIAK----------------

>Tad-gi|195999522|

AYVRKMVE---SCRNNKLVNR-IQLELNDRMRYILIDWLVEVAEMKEFSSEMLCNAIDLVDRYLEINPIPRSNLQLLGISCMVIASRYHCVDIMTIREAAWLTDNTYKYDEVVRMIGEVFAAVNGEIRTPSAFDYLKIFCTISEVSQKCTYLASFILELSWLFLENSRYKSAVKAAASLLLARVLMGELPWTEELKSYTGLSLEDLSSCVLHLYKKKDYYNSEVKSVHNRY

>Tad-gi|196003236|

EVWQVMIENDNNFKRSPDCFD-RHPNLVPNMRGILLDWMMEVCESFKMQRETFYMAMDYLDRYLSLSNILKQKLQLIGTTCLFIAAKIEEIQPPQVSEFAYVTDSACSEDDIIKLELQLLQTLEFQLSPVTVTSWLNVYVQLFNIEPDLYMKAIRLIDLCILDSWCLMHSYRSIAASAFYLIA--------PSLAIDCTGYLWENLTSCISWMM-----------------

>Tad-gi|196001479|

RVLQNLLKLEENYLPNRDYFTNVQREIKPFMRCMVTNWMLDVCHGENCNDKVFPLAVNYLDRFLSMVSIRKIRLQLVGSVCLFLASKLKDRIPLTAEKLCSWTDYSVTCQELLDWELLLLEKLKWDLGSVTPLDFLDQILYRLSFEKDLKKHAYTLIALCCTDFQLCTNPPSMIAGGCILCALAG---VIVNRIVQKITSIEPDYLICCKEQ-------------------

>Tad-gi|196011367|

YSQWVLDRQEILAG--EEDLSYLSEDEIFKIHMFFANFIRHLGDLLKLRQQVIATAIVYFKRFYSRNSLKSIAPLLLAPTCILLASKAEECG-INTGRFINACTYPYKMPVILECEFFLLELLDCSLIVFHPYRPLLQFVEDFEKKDALLPCAWRAINDSYNTDICLMYPPYIIALACLHTACIIQS-TQWFAEL----------------SVDL----------------

>Tad-gi|196007260|

LENTIYPDEKL-QE--PSQLDGLSKEMETNLRIAGCEYIQAAGILLKLPQVAMATAQVLYQRYYYSKSFVKYNYEICAMACIFLAAKIEEHP-RRIRDVVNVFHYFHKKSEVIKAERRVLKELGFCVHVKHPHKCIVTYLKILECNDELIQKAWNYMNDSLRTDVFMRYTPETIACACIYLSARQLGIPPWYEVF------------------------------------

>Tad-gi|196000815|

--GEKSKVVMPPVV--ISAQQLIEDKDHWPEYLKIGKFIAESGIKLKLGSVVIARAATIYHRFYFLCDISQFDRYLVAVTCLYLASKVEDTP-RRARDVITTSYYWQLRDSVVNFELFMLRMLKFDVSSELPHKYLLHYLKSLQDTNHINQLCWQLLQDTSLLPFILLYPPSVIATAVIYLAVKCNNIKPWWNVF----SPNEEGLQQLCYKFME----------------

>Tad-gi|196007480|

---WYFCSNEI-VN--PSRSDGIEITAECRYRREGARFIMDVGNRMNLRYETVATGIVFYHRFYMMHSFKTINRLIGAAACLYLAGKAEETP-KKCRDLVKAVRGDDPKEEIISHERLLLQTIKFDLCVQHPYKYIVKFAKNLKDDRKVVQMAWNFVNDSLSTTLCLQWKPQVVAVSLLHLAAKLSKYKSWWQHF----LPNSDVLEDICLQMLD----------------

>Tad-gi|196012973|

--------------------------ITPTQREALISSMINLSRKHNIKRDTLFMAVNYLDRFLKVVTVTEDCFELVGLTCMMIACKVEECQPPKMEEFLTSCTHYYKKAEMKRLEIIILNYIDFRLSPPIAPHFLEYIIHFQHTYIELVNIANQVLLKILPTYRFNHIKSSILAASAFEY--------------------------------------------------

>Tad-gi|195998946|

HNQFFQKLKDDDSR--IRTKDLPSVEELDHLCTFYEFELMDLCRRF--DPPMPATAAVYMKRFYLVCSVMDYHPCDIMLACVYLATKVDEYN-ISIDKFLTMVPKERAKSRTLGFELLVMEKLKFHLTIHCPYRPVEGLLINI-MTVELRRHIDKFLSKILYCQAMLIYPPSQVGEIALFPS-------------------------------------------------

>Tad-gi|196013340|

VTNFYFSDTEL-QN--PSRRNDISVATELYYRQTCALCIQELGMKLGANQLTINTALVYMHRFYMFHSLASYNLKNIAACAIFLASKSEEHP-NKLNKVITAAYFLKLSQDLVDNEYAMFFTTGFDIEIMHPHTHVIKCLHGL----------------------------------------------------------------------------------------

>Tad-gi|196013209|

--------------IKYSSCSILDSTLHPLMRRHVYKFFKTLFSSAQLTAECAIISLIYVERLMEYADIHPSNWRRVVLGAILLASKVWDDQAVWNIDYCQILK-DTTVEDMNALEREILQLIMFNINVPSSIY-AKYYF-----------------DLR---------PM------------------------------------------------------------

>Cow-gi|320162755|

QTTPRAERRLMRLLPCSSYLAKQRGEITPVMRDRLVSWLEKLNNQFEYTTETFFLAVNYVDRFLSRVRVKPRHLQLIGLASFMIAAKMQEEVKPTLQELVFCCDHAYSASEMLRMEKTILEKLKWQVHAVSHESMFFHLHERDEGFPKDAIARLGRLVSCYSNYESLAYRPSVLAAAVL------------------------------T---------------------

>Cow-gi|320167008|

EIYDWLKSREEHYVPSRNFYT-KHPTIIPRMRAILIDWMKEVCEEYGMHRETFHLAAEFVDRYLHSSAVDKNNLQLIGTTCMLIASKLEEVRPPVVADFAYVTDSACTALQIVENEMKVLMTLNWELCPITVNAWVAIFLQIATLRVDAYTKIMTLLDVAILDNPMLEYSPSLVATAGLFVTFGGQSGAQWQDFVQSVTGYTLPELESAISWQR-----------------

>Cow-gi|320166256|

ELMYTMRQAEADFTVIQDFME-GQPEISPRMRAILVDWMLEVRLELHLSNETFYLAVNILDRFLELVDTARDTLQLVGLTAMFVAAKHEETVIPVISDWLYMCDGQFQQEHLLHMELMVLDNVRFRLNVPTTFLSLMKFISGTSVDQRILYQARYFCDLASVSYSFVPVRPSMLSASALLLARMSCDEPLWSDLHQRVTGYSATTLMPFVILLAD-QRVSHLPATAVRNLY

>Cow-gi|320166453|

YSNSKRKKKANSVVFSSTFFDAVQPDHPLAKGEKIYQFIKTLFVSAALTAECAIITLVYVERLIMSATIHATNWKRITLGAVLLACKVWDDQAVWNVDFCTIFP-DVTVEDFNKLEKYYITQIMFNVSVPASVY-TKYYL-----------------DLA---------AM------------------------------------------------------------

>Cow-gi|320168069|

CNKWLVDAQQLELS--RKDLVHGSETDINLLKILYGNFAQAMGRRMRLRQLVVATALVYFRRFYFRVDWAACDPLLAITTCLYLSAKVEETG-IPVYSIITQAQFNFTVNDVVESEFYILEELGCYLIIFHPYRPLTHYCHGLD-DKQLLTTAWFILNDSYRTDLCLQYPPYMIALAALYLACIMKEKVEWFAEL----N-NPEELIEIATPILA----------------

>Cow-gi|320169044|

KILANMLQTQHGYLPTLNFLRRIKDEIQEFMRRDILEWLLRVSQHFEHHAETFATAVNLFDRFLSTLKVKPTHLQLIAATALLIAAKSQEQWHPTLSSLINASNAAFACSDILRMERIILARLKWTLATVTPHLLIHQMVPCLEQFSALIRDAEAYSDAALIEYRYANQLPSTIACGALLCALART---DLISGLLSLSGTEYDAASACFMDM------------------

>Cow-gi|320169862|

EVMLHLREREIAVRPAPSYMQ-RQNDINGNMRAVLVDWLVDVALEYRLKPETLYLAIGYIDRFLSELAIARSKLQLLGIACMFVAAKFEEIFPPNVHDFFEIADRTYEVEQIIRMEQAVLKTLRFYVSQPTLLEFINRALKVVGADAAMTSLCYYLGELTLLDDAHLVYLPSVIAAAVTLVAHYTLTPRSWTAHMAYWTGYSIEDVCKCAADVFVMDRDERNRLAAVHVKY

>Cow-gi|320169480|

AVDRLTRIHATHGT--DDGKYLTSEDEQLYLAFYLQQLAEAVVPAFPFPLAVKATAIAYLKRFYLTTSVMDYHPKHIILTCFYMACKTEEIN-IDLNAFVTNLELESDSALILQLEIILVQRLHFHLVVFHPMRSLRGFFYDVRARGAAYRDAKALIDQSFMTDACFLAPPSQLALAALCIACANHGI-------------------------------------------

>Cow-gi|320165363|

EIYEYLNNLQHVYAINPTYLS-RQPEITARMRSILVGWLFQVHQTWPFKQETLYLAVHVLDRFLQRRQVPRTRLQLIGLTSFIIAAKYEEIYIPEITEFVALTHNLFSSQDVLVAESEILVALGFNLGTPSPLHFLRRGYRASPCRPKTYTFAKYMCELSLYSAEMLEFPQSTIAGAALFVSRRITSEVAWDDSMQFYLFTPYEAMLRCARTLLELVAHTDNVYEAVYNRF

>Cow-gi|470360436|

GARWYYSDEEL-DK--PSREDGISAETEMRYRLEGVALIKEIGQQRPMSQQAIATGIVFFHRFFMCQSFKDFEASKMACTCLLLAGKVEESH-RKCYDILDVVSYYQAKEEMLVNERILLQAIAFELAVEHPYPFVMKFCKKLKRQGAFAQLVWNYVNDSLRTTLCLRYKPVLIAVAAMHLAAVTQRAEPWWKLL------------------DA----------------

>SARC_05472.1

DVYGYLFKRESTFHITDDYMSEMQTDITPKMRGVLIDWLIEVHLKFKLLQETLHLTINTIDRYLSVTVIDRNRLQLVGVTAMLIASKFEEIYAPEVRDFIYITDNAYNREEILVMERKILNTLGFKFGVPLCLHFLRRMSKSAEADPQTHTLAKYFMEMTLTDYHLQNYKQSEIAAASLCLARKMMDLDNWNENIVYYSTYDEDDITPCVMEIFQ-KNSPRAKFQACRKKY

>SARC_05471.1

DESWYFTEEELKYQ--ASRADNISFEEEEKLRRVGAGFIQDVGESIRVPQLTIATASIFFQRFYVYQSFKEYDVNIMATCCLFLAGKVEETP-KKARDVIIHSAFKNLKDQVLKCERLLLQTISFDLAVEHAYKYLLQYVKDIKGDRSLAQTAWNFVNDSLRTTLGLRYKPQVIACAGILLSSKYNKSKKWWSAF-----VSEDTLKYIGDEIIK----------------

>SARC_05809.1

LDNVTLSEEDL-KH--PSAADGLSASDEMDLRIYGCEYIQLAGKLLKLPQIAMAVAMNMYQRYYYRKSMVVHDFETCAKACIFLAAKIEENS-RRSNDVLNVCHFMVLKNEMIKAERRVLNELGFSVHIQHPHKLIANYLQVLTLYAELAQIAWSYMNDSFRTNVFCRFPPHIIAAATIYLATNMKNIPPWYELF------NKKDIEEVSK------------AILFVVHR

>SARC_07852.1

ASRWSADRWLVLLLSVNGAGRHDCDLVNEEDRMKACAELFKLQVGYHLSKETFFLALTYLDRFLSVRRVLSKHFRLVLFTCFMLAAKMQEERLPMALDLARACSPNVRVSDLKRMELKILIGLNWEMSSASSLSFLYKYLEFLRNTNALETEIALVLDLG--TKELFKYSGSQAAYIALTVATELDKSSFW----------------------------------------

>SARC_08730.1

TNGLVDVVAIAARRNCTLFVQTSPNLFPLTAQTSIYDFLRAFFKAAVLTADVAIVTMVYISRLHN-TVLQPANWKRVVLGAIMLASKVWDDQAVWNADYCKVLP-DVTVESINQLEREVLERLFYNVSVSSA---------------------------HLRIYCLRADSN------------------------------------------------------------

>SARC_09958.1

KMLFNICEKQTQFLANPQYLTRVKGDIEEGMRRDIVEWMYKLNVHFEYLHETFAVAVNLFDRFLSQVKVKPLHLQLIAATSLLLAAKIQEAWHPAYNDLVPAGNYSFSACDLQKMEKLIITKLSWNLNPVTPHVVLHQLMRFVGMKNEVIEEGENLVNSCLLESHFMIFRPSTLAACVAMMALNEM---KTLRQILFLTSTKPEIYNQCTDDL------------------

>SARC_09649.1

TVHRIDSEDENAKSSSSTVYYEICPDGPMLKQSSVYKFLCRIFLQTSLSAEIVIVATVYVERLVFKTDLHATNWRRIVLGAVILASKVWDDEAVWNVDFISLFP-NMNSADVNELERTFLELIEYNVGVKVSVF-AKYYF-----------------DLT---------RV------------------------------------------------------------

>SARC_09120.1

------------------------------DIVKAIDFAGHIGRKLRLPKEAVNVAQVLIHSFFDSNSTKEFDAPLISLSALYLASKVVECP-CRLRDAISVAYYWELRDSIVTCEMLILRCVNYNVDIVLSHKYLYIYAMALCTARQTTQLAYMIINDSFRSADVVAKAPNVIAASALYLSLRLLDKSQWWETM----GCDELLTTVDGMMAAC----------------

>SARC_11916.1

DDWLARCEVDTRYAITDEFLD-KHPEITDKMRCVLLHWLGLVCVEHELQTRTYQTACMNVDRFLSESPVLKEHFQLLGATALMMASKVHESYPIEVSRLAALTCGACFADEIVLMETHMCQVLRWQLTPMTTCEWLSFYLSLLPNTSLTTNEQKEYYKKALEILNVVTLLPNGKAVSARVLA-----------------FAALSWVLPQTTTQA-----------------

1. **Multiple alignments for phylogenetic analysis of cyclin family proteins in H. sapiens, *T.* adhaerens, *S.cerevisiae, S.pombe, C. cinerea,* and *punctatus.***

>Hsa-CyclinA1

EIYQYLREAEIRHRPKAHYMKKQPDITEGMRTILVDWLVEVGEEYKLRAETLYLAVNFLDRFLSCMSVLRGKLQLVGTAAMLLASKYEEIYPPEVDEFVYITDDTYTKRQLLKMEHLLLKVLAFDLTVPTTNQFLLQYLRRQGVCVRTENLAKYVAELSLLEDPFLKYLPSLIAAAAFCLANYTVNKHFWPETLAAFTGYSLSEIVPCLSELHKAYLDIPHRPQQAIREKY

>Hsa-CyclinA2

DIHTYLREMEVKCKPKVGYMKKQPDITNSMRAILVDWLVEVGEEYKLQNETLHLAVNYIDRFLSSMSVLRGKLQLVGTAAMLLASKFEEIYPPEVAEFVYITDDTYTKKQVLRMEHLVLKVLTFDLAAPTVNQFLTQYFLQQPANCKVESLAMFLGELSLIDDPYLKYLPSVIAGAAFHLALYTVTGQSWPESLIRKTGYTLESLKPCLMDLHQTYLKAPQHAQQSIREKY

>Hsa-CyclinB2

DIYQYLRQLEVLQSINPHFLDG-RDINGRMRAILVDWLVQVHSKFRLLQETLYMCVGIMDRFLQVQPVSRKKLQLVGITALLLASKYEEMFSPNIEDFVYITDNAYTSSQIREMETLILKELKFELGRPLPLHFLRRASKAGEVDVEQHTLAKYLMELTLIDYDMVHYHPSKVAAAASCLSQKVLGQGKWNLKQQYYTGYTENEVLEVMQHMAKVKVNENLTKFIAIKNKY

>Hsa-CyclinB1

DIYAYLRQLEEEQAVRPKYLLG-REVTGNMRAILIDWLVQVQMKFRLLQETMYMTVSIIDRFMQNNCVPKKMLQLVGVTAMFIASKYEEMYPPEIGDFAFVTDNTYTKHQIRQMEMKILRALNFGLGRPLPLHFLRRASKIGEVDVEQHTLAKYLMELTMLDYDMVHFPPSQIAAGAFCLALKILDNGEWTPTLQHYLSYTEESLLPVMQHLAKVMVNQGLTKHMTVKNKY

>Hsa-CyclinD3

RVLQSLLRLEERYVPRASYFQVQREIKPHMRKMLAYWMLEVCEEQRCEEEVFPLAMNYLDRYLSCVPTRKAQLQLLGAVCMLLASKLRETTPLTIEKLCIYTDHAVSPRQLRDWEVLVLGKLKWDLAAVIAHDFLAFILHRLSLPALVKKHAQTFLALCATDYTFAMYPPSMIATGSIGAAVQGLG--ELTELLAGITGTEVDCLRAC-----------------------

>Hsa-CyclinB3

EIFSYMKEREEQFIL-TDYMNRQIEITSDMRAILVDWLVEVQVSFEMTHETLYLAVKLVDLYLMKAVCKKDKLQLLGATAFMIAAKFEEHNSPRVDDFVYICDDNYQRSEVLSMEINILNVLKCDINIPIAYHFLRRYARCIHTNMKTLTLSRYICEMTLQEYHYVQEKASKLAAASLLLALYMKKLGYWVPFLEHYSGYSISELHPLVRQL--LLTFSSYDSLKAVYYKY

>Hsa-CyclinD2

RVLQNLLTIEERYLPQCSYFKVQKDIQPYMRRMVATWMLEVCEEQKCEEEVFPLAMNYLDRFLAGVPTPKSHLQLLGAVCMFLASKLKETSPLTAEKLCIYTDNSIKPQELLEWELVVLGKLKWNLAAVTPHDFIEHILRKLPQQSLIRKHAQTFIALCATDFKFAMYPPSMIATGSVGAAICGL---SSLTCDALTELLAKITNTDV-----------------------

>Hsa-CyclinE1

EVWKIMLNKEKTYLRDQHFLEQHPLLQPKMRAILLDWLMEVCEVYKLHRETFYLAQDFFDRYMATENVVKTLLQLIGISSLFIAAKLEEIYPPKLHQFAYVTDGACSGDEILTMELMIMKALKWRLSPLTIVSWLNVYMQVAYLNDLHVLLIAELLDLCVLDVDCLEFPYGILAASALYH--------FSSSELQKVSGYQWCDIENCLD---------------------

>Hsa-CyclinE2

EVWLNMLKKESRYVHDKHFEVLHSDLEPQMRSILLDWLLEVCEVYTLHRETFYLAQDFFDRFMLTKDINKNMLQLIGITSLFIASKLEEIYAPKLQEFAYVTDGACSEEDILRMELIILKALKWELCPVTIISWLNLFLQVDALKDAPVLLIAQLLDLCILAIDSLEFQYRILTAAALC-H-------FTSIEVKKASGLEWDSISECTN---------------------

>Hsa-CyclinK

KPCWYWDKKDLAHTP--SGL--DPATEARYRREGARFIFDVGTRLGLHYDTLATGIIYFHRFYMFHSFKQFPRYVTGACCLFLAGKVEETPK-KCKDIIKTAFGDDPKEEVMVLERILLQTIKFDLQVEHPYQFLLKYAKQLKGDNKLVQMAWTFVNDSLCTTLSLQWEPEIIAVAVMYLAGRLCKGPPAVPPP----PIPPPGMPP------------------------

>Hsa-CyclinJ

DIHQALRYKELK-L--PSYKGQSPQ--LSLRRYFADLIAIVSNRFTLCPSARHLAVYLLDLFMDRYDISIQQLHLVALSCLLLASKFEEKEVPKLEQLNSNMNLVLTKQNLLHMELLLLETFQWNLCLPTAAHFIEYYLSEAVHELYMAKYADYFLEVSLQDYAFLNYAPSLVAAACVASSRIILRLPTWPTRLHRLTAYSWDFLVQCIE---------------------

>Hsa-CyclinF

QLFQASQA-----VSKQQVFSVQKGLNDTMRYILIDWLVEVATMKDFTSLCLHLTVECVDRYLRRRLVPRYRLQLLGIACMVICTRFISKEILTIREAVWLTDNTYKYEDLVRMMGEIVSALEGKIRVPTVVDYKEVLLTLVPVELRTQHLCSFLCELSLLHTSLSAYAPARLAAAALLLARLTHGQQPWTTQLWDLTGFSYEDLIPCVLSLFHAPKDYRQVSLTAVKQRF

>Hsa-CyclinO

SCYAFRKAQESHFHPR-EALARQPQVTAESRCKLLSWLIPVHRQFGLSFESLCLTVNTLDRFLTTTPVAADCFQLLGVTSLLIACKQVEVHPPRVKQLLALCCGAFSRQQLCNLECIVLHKLHFTLGAPTISFFLEHFTHA-RVALEAQALARGVAELSLADYAFTSYSPSLLAICCLALADRMLRVS--RPVDLRLGDHPEAALEDCM---------GKLQLLVAINSTS

>Hsa-CyclinD1

RVLRAMLKAEETCAPSVSYFKVQKEVLPSMRKIVATWMLEVCEEQKCEEEVFPLAMNYLDRFLSLEPVKKSRLQLLGATCMFVASKMKETIPLTAEKLCIYTDNSIRPEELLQMELLLVNKLKWNLAAMTPHDFIEHFLSKMPEAQIIRKHAQTFVALCATDVKFISNPPSMVAAGSVVAAVQGLN--YRLTRFLSRVIKCDPDCLRA-----------------------

>Hsa-CyclinT2

SSRWFFTREQLENTP--SGV--EADKELSCRQQAANLIQEMGQRLNVSQLTINTAIVYMHRFYMHHSFTKFNKNIISSTALFLAAKVEEQAR-KLEHVIKVAAYLQQTQELVILETIMLQTLGFEITIEHPHTDVVKCTQLVR---DLAQTSYFMATNSLHTTFCLQYKPTVIACVCIHLACKWSN-KHWWKKQ----EYTHKAGSSKHHSHSSGSKHSADGIPPTVLRSP

>Hsa-CyclinL2

LENCLLPDDKLRFTP--SGL--DTDTETDLRVVGCELIQAAGILLRLPQVAMATGQVLFQRFFYTKSFVKHSMEHVSMACVHLASKIEEAPR-RIRDVINVFDYVNLKNQIIKAERRVLKELGFCVHVKHPHKIIVMYLQVLECEQHLVQTSWNYMNDSLRTDVFVRFQPESIACACIYLAARTLEIPHWFLLF----GATEEEIQEICAPRKGIRGSRKSKDCKYPQKPH

>Hsa-CyclinG2

GLLNVYLEQEERFQPREATPENDNTLCPGLRNAKVEDLRSLANFFGSCTETFVLAVNILDRFLALMKVKPKHLSCIGVCSFLLAARIVEENIPSTHDVIRISQCKCTASDIKRMEKIISEKLHYELEATTALNFLHLYHTI-ILCSERKSLDKLEAQLCNCRLIFSKAKPSVLALCLLNLE--TLFFEGC--------------------------------------FDE

>Hsa-CyclinT1

NKRWYFTREQLENSP--SGV--DPDKELSYRQQAANLLQDMGQRLNVSQLTINTAIVYMHRFYMIQSFTQFPGNSVAPAALFLAAKVEEQPK-KLEHVIKVAAYLQQVQDLVILESIILQTLGFELTIDHPHTHVVKCTQLVR---DLAQTSYFMATNSLHTTFSLQYTPPVVACVCIHLACKWSN-KHWWMDK----AASSKPEEIKMKLDGAHNTTQTIDYQDTVNMLH

>Hsa-CyclinL1

IDHSLIPEERLSPTP--SGL--DLPSETDLRILGCELIQAAGILLRLPQVAMATGQVLFHRFFYSKSFVKHSFEIVAMACINLASKIEEAPR-RIRDVINVFNYINTKNQVIKAERRVLKELGFCVHVKHPHKIIVMYLQVLECEQTLVQTAWNYMNDSLRTNVFVRFQPETIACACIYLAARALQIPHWFLLF----GTTEEEIQEICRRHSPKAKHTRDDLKSSNRHGH

>Hsa-CABLES1

VIDYVKPSDLKKDMNETFK--EKHIKTLKIRSLKREMRKLAQEDCGLEEPTVAMAFVYFEKLALKGKLNKQNRKLCAGACVLLAAKIGSDKKHEVKHLIDKLEEKLNRRELIAFEFPVLVALEFALHLPEH-VHYRRL---------------------------------------------------------------------------------------------

>Hsa-CyclinC

LQWILDKQDLLKERQ--KFL--SEEEYWKLQIFFTNVIQALGEHLKLRQQVIATATVYFKRFYARYSLKSIDPVLMAPTCVFLASKVEEFGVVSNTRLIARFEFPYRMNHILECEFYLLELMDCCLIVYHPYRPLLQYVQDMG---MLLPLAWRIVNDTYRTDLCLLYPPFMIALACLHVACVVQ--RQWFAEL----SVDMEKI--------------------------

>Hsa-CyclinG1

HQLNALLEQESRCQPKESAHDNGLRMTARLRDFEVKDLLSLTQFFGFDTETFSLAVNLLDRFLSKMKVQPKHLGCVGLSCFYLAVKSIEENVPLATDLIRISQYRFTVSDLMRMEKIVLEKVCWKVKATTAFQFLQLYYSL-QENLERRNFERLEAQLCHCRIIFSKAKPSVLALSIIALE-------CL--------------------------------------TEY

>Hsa-CyclinH

RKFRCKAVANGKVLP--NFL--EPHEEMTLCKYYEKRLLEFCSVFKMPRSVVGTACMYFKRFYLNNSVMEYHPRIIMLTCAFLACKVDEFNV-SSPQFVGNLGQEKALEQILEYELLLIQQLNFHLIVHNPYRPFEGFLIDLPILPEIRKTADDFLNRIALTDAYLLYTPSQIALTAILSSAS--G-----------------------SLMRTSQLLDIMKSMRNLVKKY

>Hsa-CyclinI

ENQRLSFLLEKAITREVRKMPSNQNVSPSQRDEVIQWLAKLKYQFNLYPETFALASSLLDRFLATVKAHPKYLSCIAISCFFLAAKTVEERIPVLKVLARDSFCGCSSSEILRMERIILDKLNWDLHTATPLDFLHIFHAIALLFPKLSHLAVLTKQLCMACNQLLQFRGSMLALAMVSLEMELQKGLVD--------------------------------------DFY

>Hsa-Fam58A

------PEGGGGGPA--AEG--QPAPEARVHFRVARFIMEAGVKLGMRSIPIATACTIYHKFFCETNLDAYDPYLIAMSSIYLAGKVEEQHL-RTRDIINVSRFWELRDSIVQCELLMLRVLRFQVSFQHPHKYLLHYLVSLQRHWPVAVTAWALLRDSYHGALCLRFQAQHIAVAVLYLALQVYGVKPWWQKP----IIDNIVSDL------------------------

>Hsa-CABLES2

TVIEYVKPSDLKKDMNETF--REPHVLTSKIRSLKREMRSLSEECSLEPVTVAMAYVYFEKLVLQGKLSKQNRKLCAGACVLLAAKISSDRKSGVTQLIDKLEERFNRRDLIGFEFTVLVALELALYLPEN-VHYRRL---------------------------------------------------------------------------------------------

>Hsa-Fam58B

--MEDAGEEAGEDAG--EEG--AAAPAARVHFRVARFIMEAGVKLGMQSIPIATACTIYPKFFCETILDAFDPYLIAMSSIYLAGKVEEQPL-WAHDIISVSRLWELRDSIVQRELLMLRVLRFQVSFQHPHKYLLYYLVSLKCHWPVAVTAWALLRDSYHGGLCLRFQAQHIAVVVLYLALQVYGVKLWWQKP----IIDTIVSDL------------------------

>Hsa-CyclinI2

EGDLDERRLLCHLQLAWRGGKPQDEICDAF-EEVVLWLLRLQNTFYFSQSTFNLALTIFGRLLISVKVKEKYLHCATITSLRLAAKVNEEFIPQVKDFTKHYGSDYSPNELLRMELAILDRLHWDLYIGTPLDFLTIFHALVVLEPQRNHVASLTRQLCMAGHQLLQFKGSTLALVIITLEL------GW--------------------------------------CAP

>Hsa-CyclinJL

DVHCTLREKELK-L--PTFRAHSPL--LKSRRFFVDILTLLSSHCQLCPAARHLAVYLLDHFMDRYNVTSKQLYTVAVSCLLLANGFEDREVPKLEQINSSQNFTLTKKELLSTELLLLEAFSWNLCLPTPAHFLDYYLLASSQKECLKEYAHYFLEVTLQDHIFYKFQPSVVAAACVGASRICLQLPYWTRDLQRISSYSLEHLSTCIEIL-------------------

>Hsa-CyclinYL

KSNHLNHCDLSNILPHKEQRKH---D--PEHKFIYRFVRTLFSAAQLTAECAIVTLVYLERLLTYAEIDPTNWKRIVLGAILLASKVWDDQAVWNVDYCQILKD-ITVEDMNEMERHFLELLQFNINVPASVY------------------AKYYFDLRSLDNLNFLFAP-------------------------------------------------------------

>Hsa-CyclinY

DPDGRMLLDIFDENLHPLSKKH---N--PEQKQIYRFVRTLFSAAQLTAECAIVTLVYLERLLTYAEIDPANWKRIVLGAILLASKVWDDQAVWNVDYCQILKD-ITVEDMNELERQFLELLQFNINVPSSVY------------------AKLEA---------------------------------------------------------------------------

>Tad-gi|196003740|

IIFENMKQREAQLVVN-DYLERQNDITEQMRMILIDWLCEVQQNFELFHETLYLAVKIVDRFLSARVVSRDALQLIGATAMLMSSKIEERYPPLVDDFVYICDDAYSRQAVLDMERDICYALDFDLNIPIPYRFLRRYGKVASLSMENLTLARYILELTLQEYQFVTFKPSMLAAGCLCLALKMKNCGEWTQTLVHYSGYEESELNELVQKLNAMIAKPAPENCKVVKTKY

>Tad-gi|196005765|

DIHSYLRKAEYFHRPKYDYMERQCDVNGTMRSILVDWLVEVSEEYKLRERTLYLAISYIDRFLSAMSVRRSKLQLVGTAALFIAAKFQEIYPPDCAEFAYITDDTYNIKQVLKMESLMLKVLSFNLSSPTAVDFLERYGSEAGLDSEIRELSMYLTELTLKDYGFLQFMPSLIAVSAVSLALHTFKLKYWPQELSTYTNYQWQQVSPCLNRIFEAFRLAHTQPQRAVVEKY

>Tad-gi|196002535|

DIYKNMLKQEKRCTLDPDYMTGQPVITKGMRAILLDWLVDVHLRYNFHPESLYLTTYIIDRYLQTTQVNRKKLQLVGIAAFYIAIKYEEIFLASTDDLLYLTENSYEINEFIQMEAKILKALDFSLSRPTSIHFLRRISKAASADIEQHTFARYLTEIALIEYSLLSYLPSQIAAAASLISLKIFD-KSWTPTLQYYSSYSEDSLKPVARQI-------------------

>Tad-gi|195999522|

KMVESCRN-----INIKLVNRIQLELNDRMRYILIDWLVEVAEMKEFSSEMLCNAIDLVDRYLEINPIPRSNLQLLGISCMVIASRYHCVDIMTIREAAWLTDNTYKYDEVVRMIGEVFAAVNGEIRTPSAFDYLKIFCTISEVSQKCTYLASFILELSWLFLENSRYKSAVKAAASLLLARVLIGNLPWTEELKSYTGLSLEDLSSCVL-----HLYKKCLAEKPPKDYY

>Tad-gi|196003236|

EVWQVMIENDNNFKRSPDCFDRHPNLVPNMRGILLDWMMEVCESFKMQRETFYMAMDYLDRYLSLDNILKQKLQLIGTTCLFIAAKIEEIQPPQVSEFAYVTDSACSEDDIIKLELQLLQTLEFQLSPVTVTSWLNVYVQLSSQEPVGMLYAIRLIDLCILDSWCLMHSYRSIAASAFYLI-------APSKQLIDCTGYLWENLTSCIS---------------------

>Tad-gi|196001479|

RVLQNLLKLEENYLPNRDYFTVQREIKPFMRCMVTNWMLDVCHGENCNDKVFPLAVNYLDRFLSMVSIRKIRLQLVGSVCLFLASKLKDRIPLTAEKLCSWTDYSVTCQELLDWELLLLEKLKWDLGSVTPLDFLDQILYRLSFEHMLKKHAYTLIALCCTDFQLCTNPPSMIAGGCILCALA-----QNDNSIAIVNRIVQKITSIE-----------------------

>Tad-gi|196011367|

SQWVLDRQEILAGRE--EYL--SEDEIFKIHMFFANFIRHLGDLLKLRQQVIATAIVYFKRFYSRNSLKSIAPLLLAPTCILLASKAEECGIINTGRFINKYDYPYKMPVILECEFFLLELLDCSLIVFHPYRPLLQFVEDFE---ALLPCAWRAINDSYNTDICLMYPPYIIALACLHTACIIQ--TQWFA---------------------------------------

>Tad-gi|196007260|

LENTIYPDEKLQETP--SGL--SKEMETNLRIAGCEYIQAAGILLKLPQVAMATAQVLYQRYYYSKSFVKYNYEICAMACIFLAAKIEEHPR-RIRDVVNVYEYFHKKSEVIKAERRVLKELGFCVHVKHPHKCIVTYLKILECNTELIQKAWNYMNDSLRTDVFMRYTPETIACACIYLSARQLGIPPWY----------------------------------------

>Tad-gi|196000815|

-GEKSKVVMPPVVKI--SLI--EDKDHWPEYLKIGKFIAESGIKLKLGSVVIARAATIYHRFYFLCDISQFDRYLVAVTCLYLASKVEDTPR-RARDVITTSFYWQLRDSVVNFELFMLRMLKFDVSSELPHKYLLHYLKSLQESWHINQLCWQLLQDTSLLPFILLYPPSVIATAVIYLAVKCNNIKPWW-SP----NLNEEGLQQ------------------------

>Tad-gi|196007480|

---WYFCSNEIVNSP--SGI--EITAECRYRREGARFIMDVGNRMNLRYETVATGIVFYHRFYMMHSFKTINRLIGAAACLYLAGKAEETPK-KCRDLVKAVFGDDPKEEIISHERLLLQTIKFDLCVQHPYKYIVKFAKNLKDDAKVVQMAWNFVNDSLSTTLCLQWKPQVVAVSLLHLAAKLSKVDFYDKYN----MISPPKITM------------------------

>Tad-gi|196012973|

-------------------------ITPTQREALISSMINLSRKHNIKRDTLFMAVNYLDRFLKVVTVTEDCFELVGLTCMMIACKVEECQPPKMEEFLTSCTHYYKKAEMKRLEIIILNYIDFRLSPPIAPHFLEYIIHFHQHYIELVNIANQVLLKILPTYRFNHIKSSILAASAFEYA--------------------------------------------------

>Tad-gi|195998946|

NQFFQKLKDDDSRHI--RLP--SVEELDHLCTFYEFELMDLCRRFD--PPMPATAAVYMKRFYLVCSVMDYHPCDIMLACVYLATKVDEYNI-SIDKFLTMVDKERAKSRTLGFELLVMEKLKFHLTIHCPYRPVEGLLINIEIAIDERRHIDKFLSKILYCQAMLIYPPSQVGEIALFPS--------------------------------------------------

>Tad-gi|196013340|

VTNFYFSDTELQNTP--SDI--SVATELYYRQTCALCIQELGMKLGANQLTINTALVYMHRFYMFHSLASYNLKNIAACAIFLASKSEEHPN-KLNKVITAAKFLKLSQDLVDNEYAMFFTTGFDIEIMHPHTHVIKC---------------------------------------------------------------------------------------------

>Tad-gi|196013209|

YRHDPPRLKDIFDEELHPLMIN---L--PEHRHVYKFFKTLFSSAQLTAECAIISLIYVERLMEYAEIDPSNWRRVVLGAILLASKVWDDQAVWNIDYCQILKD-TTVEDMNALEREILQLIMFNINVPSSIY------------------AKYYF---------------------------------------------------------------------------

>Sce-CLB4p-gi|6323239|

DIFYYLRELEVKYRPNPYYMQNQVELTWPFRRTMIDWLVQLHFRFQLLPETLYLTINIVDRFLSKKTVTLNRFQLVGVSALFIAAKFEEINCPTLDDLVYMLENTYTRDDIIRAEQYMIDTLEFEIGWPGPMPFLRRISKADDYDFEPRTLAKYLLETTIVEPKLVAAAPSWLAAGAYFLSRTILGSNDWSLKHVFYSGYTSSQIIPLASLILE-NCKNASRRHHSIWKKY

>Sce-CLB2p-gi|6325376|

DIFEYLHQLEVITLPKKEDLYQHRNI-HQNRDILVNWLVKIHNKFGLLPETLYLAINIMDRFLGKELVQLDKLQLVGTSCLFIASKYEEVYSPSIKHFASETDGACTEDEIKEGEKFILKTLKFNLNYPNPMNFLRRISKADDYDIQSRTLAKFLLEISLVDFRFIGILPSLCAAAAMFMSRKMLGKGKWDGNLIHYSGYTKEELAPVCHMIMD---LVSPIVHDEFHRKY

>Sce-CLB1p-gi|6321545|

DIFDYLHHLEIITLPNKANLYKHKNI-KQNRDILVNWIIKIHNKFGLLPETLYLAINIMDRFLCEEVVQLNRLQLVGTSCLFIASKYEEIYSPSIKHFAYETDGACSVEDIKEGERFILEKLDFQISFANPMNFLRRISKADDYDIQSRTLAKFLMEISIVDFKFIGILPSLCASAAMFLSRKMLGKGTWDGNLIHYSGYTKAKLYPVCQLLMD---LVGSTIHDEFLKKY

>Sce-CLB3p-gi|6320046|

EIFEYMRKLEDLYKPNPYYMDKQPELRWSFRSTLIDWIVQVHEKFQLLPETLYLCINIIDRYLCKEVVPVNKFQLVGAASLFIAAKYEEINCPTIKDFVYMSENCYSRNDLLDAERTILNGLEFELGWPGPMSFLRRISKADDYEHDTRTLAKYLLESTIMDHRLVSAQPSWLAAGAYFLSKIILGQNQWSLAHVYYSNYTQEQILPLATIILE-NCRYASKRHNAIWRKY

>Sce-CLB6p-gi|6321546|

SIFSHLYEKEIQMLPTHNYLMTQYHLKSSMRALLIDWLVEVHEKFHCLPETLFLAINLLDRFLSQNVVKLNKLQLLCITCLFIACKFEEVKLPKITNFAYVTDGAATVEGIRKAELFVLSSLGYNISLPNPLNFIRRISKADNYCIETRNMAKFIMEYSICCNKFIHLKPSYLAAMSMYIARKIKNESKWDETFIHYSGGIDIESDPISELVED---AVPDTNLDSLRLKY

>Sce-Clb5p-gi|6325377|

EIFAFLYRRELETLPSHNYLLKTYYLRPSMRTILVDWLVEVHEKFQCYPETLFLSINLMDRFLAKNKVTMNKLQLLAVTSLFIAAKFEEVNLPKLAEYAYITDGAASKNDIKNAEMFMLTSLEFNIGWPNPLNFLRRISKADDYDPVNRNIGKFILEYAYCCHQFIHLPPSTVSAMAMYIARRMTNRELWNGTLQHYSGGIDPIHDECIDLVKD---ASSKTHLDSLILKY

>Sce-CCL1p-gi|6325282|

REAHNLTEEEIKVLE--APL--TMEEELDLVNFYAKKVQVIAQHLNLPTEVVATAISFFRRFFLENSVMQIDPKSIVHTTIFLACKSENYFI-SVDSFAQ----KSTRDSVLKFEFKLLESLKFSLLNHHPYKPLHGFFLDIVDLMGQYDRCKKRITAALLTDVVYFYTPPQITLATLLIEEG--E-----------------------PQNTEKEKSTESEEYSIDSAKL

>Sce-CTK2-gi|6322454|

FFSRPFLSKRQIQRA--QIS--DYRNYNQKKLAVFKFLSDLCVQLKFPRKTLETAVYFYQRYHLFNRFETEVCYTVATSCLTLGCKEVETIK-KTNDICLR-ILENFKKRVFQIELRILESCSFDYRVNNYVEYVIKIGRELSFDYKLCNLAWVIAYDALKLETILVIPQHSIALAILKIAYELLDNKNW-------------------VNFFDDLQRHLPADLLPIGVER

>Sce-CLN2p-gi|6324999|

EISTNVIAQSCKFKPNPKLIDQQPEMNPETRSNIITFLFELSVVTRVTNGIFFHSVRLYDRYCSKRIVLRDQAKLVVATCLWLAAKTWGGCIPRLSELVHYCGDVFDESMFLQMERHILDTLNWNIYEPMINDYVLNVDCQQSQDKIKINLKKFLIDVSAWQYDLLRYELFEVSHGI------------------------------------------------------

>Sce-SSN8p-gi|6324303|

SKQNGIEQSITKNIP--IDL--HYDKDYNLRIYCYFLIMKLGRRLNIRQYALATAHIYLSRFLIKASVREINLYMLVTTCVYLACKVEECPQ-YIRTLVSEAFIPPDPTKVTEFEFYLLEELESYLIVHHPYQSLKQIVQVLT---DDLQNCWSLINDSYINDVHLLYPPHIIAVACLFITISIHG-ASEAIRD----PKNSSSP--------------------------

>Sce-CLN1p-gi|6323855|

EISQNVLVQSSKTKPDIKLIDQQPEMNPQTREAIVTFLYQLSVMTRVSNGIFFHAVRFYDRYCSKRVVLKDQAKLVVGTCLWLAAKTWGGCIPRLSELVHYCGGLFDESMFIQMERHILDTLNWDVYEPMINDYILNLQNRDEEGKIKINLKRFLIDLSCWQYNLLKFE--------------------------------------------------------------

>Sce-CLN3p-gi|6319277|

DQLDHYFRLSHTERPNLTNFNSQPQVNPKMRFLIFDFIMYCHTRLNLSTSTLFLTFTILDKYSSRFIIKSYNYQLLSLTALWISSKFWDSKMATLKVLQNLCCNQYSIKQFTTMEMHLFKSLDWSICQSATFSYI----FPAQQGNEILGAIMLCELASFNLELSFKYDRSLIALGAINLIKLS----LW-----------------------------------------

>Sce-PCL1p-gi|6324040|

DIIKFLTDTTLRVVPSSNYPKH---L--TRLPSLMTFITRLVRYTNVYTPTLLTAACYLNKLKRILATGPSTIHRIFLACLILSAKFHNDSSPLNKHWARYTDGLFTLEDINLMERQLLQLLNWDLRVNTEDL------------------NRNVSIASDLSLVDSCNDL-------------------------------------------------------------

>Sce-PCL9p-gi|6320022|

KKPVSQEMIQFLATSTASIIIQ---G--CRPPDLSIFIKNVVIQSNVQTPTLMATSVYLNKLKSVIVYGNTTRHRIFLGCLILAAKTLNDSSPWNKHWTTYTEGLLRIREVNTIERELLEYLNWDVRITTPDLSLSYFLG--FLQRRQEMLSSDNFNKKNHPNNNDICNN-------------------------------------------------------------

>Sce-PCL2p-gi|42742057|

KAVSKEMVQYLASTTASIIKDI---A--LPAPPLTKFINRLIKHSNVQTPTLMATSVYLAKLRSIIVYGETTRHRIFLGCLILAAKTLNDSSPLNKHWAEYTDGLLILREVNTIERELLEYFDWDVTISTDDLFLKPIKE--QRDCRTLKNTNISDKFSPRYDSKHDNKE-------------------------------------------------------------

>Spo-cdc13-gi|19111963|

DIFEYLNELEIETMPSPTYMDRQKELAWKMRGILTDWLIEVHSRFRLLPETLFLAVNIIDRFLSLRVCSLNKLQLVGIAALFIASKYEEVMCPSVQNFVYMADGGYDEEEILQAERYILRVLEFNLAYPNPMNFLRRISKADFYDIQTRTVAKYLVEIGLLDHKLLPYPPSQQCAAAMYLAREMLGRGPWNRNLVHYSGYEEYQLISVVKKMIN---LQKPVQHEAFFKKY

>Spo-cig2-gi|19114801|

EIFEYIRKLDLKCLPNPKYMDQQKELTWKMREILNEWLVEIHSNFCLMPETLYLAVNIIDRFLSRRSCSLSKFQLTGITALLIASKYEEVMCPSIQNFVYMTDGAFTVEDVCVAERYMLNVLNFDLSYPSPLNFLRKISQAEGYDAQTRTLGKYLTEIYLFDHDLLRYPMSKIAAAAMYLSRRLLRRGPWTPKLVESSGYEEHELKEIAYIMLH--YHNKPLEHKAFFQKY

>Spo-cig1-gi|63054436|

EIFHYMQSLERKLAPPPNYMSVQQEIDWVTRHMLVDWIVQVQIHFRLLPETLFLAVNLIDRFLSIKVVSLQKVQLVGLSALLIACKYEEIHPPSIYNFAHVVQGIFTVDEIIRAERYMLMLLDFDISWPGPMSFLRRISRADSYDHDIRMLAKYLQEVTLMDEIFIGAHISFIAATAYYLSMQMLGHLDWTPCHVYYSGYTARQLKPCAIIIME--LVDAPNHHNAIYRKY

>Spo-rem1-gi|19112590|

EILSHMEKLEIRFMPDYRHMSAQPYYVTEMRASVINWIVGVHTCINLLPESLFLSINVLDRFLSLQNVPASKMKLCGATALFIACKYEEIHPPTVKDLEIVLEGEWIGEDICGMEKYMLMVLQYQLGWPGPVSFLRLLTIVNKWESQLRIMIKYFLEVSLVEQRFSSLRASQLVATCAYTGQSILQEENWSNTLPQITGYDYMSLVSYVHLLLK--LENPFDHHYAIYSKY

>Spo-puc1-gi|19113331|

DIIHHLITREKNFLLNVHLSNQQPELRWSMRPALVNFIVEIHNGFDLSIDTLPLSISLMDSYVSRRVVYCKHIQLVACVCLWIASKFHETEVPLLQELKLACKNIYAEDLFIRMERHILDTLDWDISIPTPASYIPVLDP-----------FFLVLDASMFVPNLFKFPASKIACSVMNIVN----------------------EHVGNE---------------------

>Spo-mcs2-gi|19112568|

MLEELSLQNKEASLE--VTL--TVEEELELVNYYSFQLNALSSALSLPTHIRSTAILFFKRFYLINSVMEYSPKIISFTSLFLATKCNDHYI-SIEQFCKNMP-KTTPEEVLEYEFNVCQSLKWDLYVWLPFRPLQGFLLD-VAVFYEHDLSKKFLIETLHSDIYFLHSPSIIALGAIYHTN---------------------------QYIIPQPLIISISANLKATKKF

>Spo-SPBC530-gi|19112118|

LSIRMSHPYYSEKEI--SST--RDPKENNLRMQAFAWISTLSKTLKFPVRTSGLAMLLYSRFQLFFPVNEIPLLECATACLVVASKIEDTAK-KFRDILLA-VDAHSQKRILGLERMTLELICFDFRVRHPHNYMVKFAKSLKFSSSTASIAWNVCTDAYKTYTMLKYPAHIVAVASISIACKLQSFFAP-------------------CIGTELGLCVDFQRAQKNSGRP

>Spo-srb11-gi|19112745|

--YWASSQLTQLFLS--TSL--EPTCLSKDTIYQWKVVQTFGDRLRLRQRVLATAIVLLRRYMLKKNEKGFSLEALVATCIYLSCKVEECPV-HIRTICNEAKVKLSRSNISEIEFEIISVLDAFLIVHHPYTSLEQAFH--I---KQLEFAWSIVNDSYASSLCLMAHPHQLAYAALLISC-------------------------------------------------

>Spo-SPAC1296-gi|19113957|

------LVHSLASSS--QFD--SFEYAEELCTLGSEWIQEAGVLLNLTQNCVIVCLILFRRYCTLYPPRVPDLDAIVMACVSIGSKTTETPA-SVQDICNVEEMYSSRNRLSNMELEVLRALNFDTHIVIPHKLAIHYLQTLQLIKKLLQITWNFLNDASRTRLCVLYPPFSLACGCIAMAARVIGMKDWYRVF----DTTKEEIDS------------------------

>Spo-pch1-gi|19112941|

TSQWIISKDQLVFTP--SGI--PLDQEEIQRSKGCNFIINVGLRLKLPQTALATANIYFHRFYLRFSLKNYHYYEVAATCIFLATKVEDSVR-KLRDIVIN-EYWRWRDVILYTEEVLLEALCFDFTVEHPYPYVLSFIKKFVADKNVTKVAWTYINDSTRSIACLLYSPKTIAAAAFQFALEKNEIPVW-------------------INPLPQKNGSHASSVAPGTPSS

>Spo-crs1-gi|295442880|

NLRRVPSVNSIIEQEKPSIL---QNIYKEKRQQLFSVLYEETVG-YVSMDTLCIAISLLDRCFTVPTIPTTSFKIYAIGCLFIAFKLTSD--------------------YSKYEKIVLALLNFDIYVPSVESFLTVFFKSLSCDQ-MMVEWQYLLVEVMKDCQFIEYRPTEILCASFWVLLEII----WPSA--------------------------------------

>Cci-gi|169844260|

KAIRSKIEANEPGSS--AFL--TPDEEVLLVKLYVAQVIAAGNKVRAQQETVSTAMSYLKRFYLRNTVMDWHPRNVMLTCLFLATKTCNAPL-SIEYFVQQFQ-KTEPSDVLDLEFLVAQSLSFEFSSLPDL-----------TIQESYNAAMAQVQASRFTDAEFIYTPSQIALASLALTLSKQD-----------------------LITPPDTVREIDRRLKLCKNPE

>Cci-gi|169844310|

EIRRYMHDMERNTMSSTQSMDQQPEIRWHMRPCLVDFLVEIHFTFRLRPETLYLTLNIIDRYVSRRVVYIKHYQLVGCAALWIAAKFEDAKVPTVQDLCHICRDTYEESAFIQMEGHVLSTIQWTLGHPTAEAWLRLFCTGVPEDNKVQHVARFLMEITLFYREFVKFSPSTIAMASLTLARYICGK-FWEET------ECLEVVNILDT----RLATNVNDLSETLIKKY

>Cci-gi|299753464|

---NSQWFFPLSALQ--AAC--SLERELYDRARGVEFLFRLGSSLQLPTSAMCTAATWLHRFYMRYPLEEFHRQEVAAACIFLATKTEECGR-KLVDVAKV-EVEDCQKAILFTEEVLLEALCFDFVVENPHSELVDLFDSCESDPLVQEYAWSLAHDSFRTPVCLLYPPRIIATACLVLAQRLFD-----------------------MLEQDSYPYLSRIASVTPPTTS

>Cci-gi|169867711|

EIFNYMKKLELTTMANPNYMESQKELAWKMRGILTDWLVQVHVRFRLLPETLFLCVNIIDRFLSARVVSLAKLQLVGITCLFIASKFEEIVSPGVSHFLSVADSTYTEAEILQAERYVLKTLDWNLSYPNPVHFLRRVSKADDYDVAVRTVGKYLLEIGCLEWRLIAAPPSLMAAAAIWLARLAMGHEEWTPNLAHYSSYRESALVPTANLMLN---ILKPIKHEAFFKKY

>Cci-gi|299740023|

DVCAYWKKTELATLPKANYMEGQQELTWDHRGILIDWILQVHARFNLLPESLFLTVNLLDRFLSARPISLNKLQLVGLACFFIASKFEETCAPSVNEIVFLADNQYTVAEVLKAEMYILRVLDWDLSCPGPMSWLRRGSKADECESTARTVAKYLLEIGCLEHRLVGIVPSHMAAAALWLGRLAVGREEWTPTLEHYTTFTEKEILPVATIMLE--IITNPIQHESLYKKY

>Cci-gi|299750771|

RKRVKDTELAFICARFLIHQRD---S--STSKNLVKFISTLIRRAALEPSVPYAALLLLQRLKTRYPTA-ATGHFLYFAAFLVASKVLSDTDLTVRSWLYSAQWSFSPVLIGKMERELCQYLDWDLTFDYE---------------------LLRLSRRMDLRRGTTTLY-------------------------------------------------------------

>Cci-gi|299749762|

EIFKYMEEMEDEIMPNPDYMDGQNEITWSMRQTLVDWLLQVHLRYHMLPETLWIAINIVDRFLTKRVVSLVKLQLVGVTAMFVAAKYEEILAPSVDEFVFMTESGYTKEEILKGERIMLQTLDFRISHCSPYSWMRKISKADDYDVQTRTLSKFLTEITLLDYRFLRVKPSMIAAIGMYCSRRMLG-GDWNEAFVFYSGYTEEQLIPGFDLIIS-KLVEENFSKLYVCKKY

>Cci-gi|299752835|

IETVDHAMNRPSTLLPASSSPR---T--PTQQKFSTFTTTVLTRAEITTPTLLLALIYIHRARPYLHIAEWALERVFLGALIVASKYANDSTLKNVHWA-LCTGVFGKRDVGRIEREFLAVLEWDLGVTESEG------------------EKSFPFPGHHHQQLPTAKR-------------------------------------------------------------

>Cci-gi|298409332|

DPFYGHEPMARMCARFITHLAA---T--HSQAKLPHFIAYALHRTKLHSSVTFAALVLLQRLKARFPTAGSSGHRLFISAFMIASKVICDDTYSNKSWGIVAQGMFSLREINQMEREMCGYLEWELTVDNPI----LANF--DFSEDKKSYASGTPSPTYSSPASSASPQ-------------------------------------------------------------

>SPPG_00434.3

KLHLFRLEMEPRYRPNPFYMEQRGSIGTDHRRELIEWLLELARHFRYYSEIFQLSVSILDRICSIETLQLSHYQALGAASFLIASKICETRTPTCVELEELAAGAFNVEGLKAMELWVLQMLNWNLNAVTPNMFLEFFLDLFKYD-TTKLIADQVLNRIQPYYHYVRFRPSVQAAAALRYAFETIGINPHVTPMHPRGKTHLHHGLAT-----------------------

>SPPG_01522.3

EIVGVMHEAEERTLPDPYYMHSQTELTWQMREALIIWLIQVHSEYDLRPETLFLNINIMDRICSTRLVTKDQYQLLGIAALWVAAKYEENHVPSLKNLSFICMHRYREKDFVRTERLILKTLEFDLNYPTCESFLRSHCKFCHVGPETRAVARYLMELGLVAGFFVGKRSSMIATASLVLAEMIMGNGPLAPR------QKDIEVIDCIN----VLAQCAGKPCDVIRFKY

>SPPG_02504.3

LRNSVLTVEQLEQSP--SGI--SQELETELRVFGCELIQSAGILLRLPQVATATAQVLFQRFYYMASLKLVAVRDIAMGAVFLASKVEETPR-KVKDIVNVFLFNELKEGIIAGELHILCKLGFNVQVQQPHGFMMNYLQGMNMVEDLTQRAWNYMNDGLRTSIYVCYQPSTIACAVIYLAARVCKVPPWWEVF----DADLEDLENVSANPENLDSAPAPCNVPLRSPTS

>SPPG_02065.3

EHAKADWEAEV------RMCNVARPFTCQQRAKAINTLFAIHSGLKCSPDVLFTAIRTFDLFIAKWKYVRRGLLLAVVATLSLAAKGDSQNPGTYKKLMGTMAPTWTPNEFMMMESKILEVVGV-YTYPTPLQFLRQINGKTRFEKKYHQTAKYLVEVAQYDQKFVRVKPSQLAAVANYTARKVMNDDPWNQHLVASSGYMEDALKELSRPL--MLVLDQNPKTSNVVKKY

>SPPG_02846.3

FETPYYYTLSDLAAH--AGF--SEEEEVEAMQRATRVLRRVYRRLKIPAKTFATSSYLLHRFYAVWPRKSYTDEEVCLACIDMACKLEETPI-GMSQLA---AKTAMEGKLAPHQQKLLEAVQFDTNIVHPFSSCAKFVKQLVTRTNLYQRASSIILDSYSIPLCIQFPPHVIAMASLYLASKFWS-----------------------DRERERDRDRERERDRDWDRER

>SPPG_05676.3

EIFEYMRQLEIDTLPGSDYMDEQKELQWNMRSILVDWLIEVHYKFRLLPETLYLAVNIVDRFLTLRVVSLVKLQLVGVTALFIAAKYEEVVAPSIQNFIYMADGGYTDEEILKAERYVLQVLDFALQYPSPMSFLRRCSKADQYDIHTRTVAKYLMEISLVDHRFLVHVPSKVAAASMSLARRMLGRVAWDAQLEYYAGYPEEEIEPAVNLMISLENESMVEKCSSLFKKY

>SPPG_08602.3

WKTDVEREEVLMENRRPDWISKHMEMNADHRGLLIDWMAEVCDENALSRRAYHLAVNFLDMYMSKAEIPLKKLQLVGVAMLNCASKLEEQQSLDVWKICEFETQGYHPDRVVKYEADFMIKMNWNLCISTAHDFLTRSFQDNLPVGERIFQAFFVLQACMAVDTTIRFRNSMLAAAVFFLTYPFED--TQAEDLHIVTGYTKAQLEE-RC---------------------

>SPPG_08550.3

FAEEWSVTKGAEVGP--PCI--TSEEQLALCRFYEGKLVHYCNYLKFDRSVQATALAFYKRFYLLNTVMDYCPKLYLMTCIFLSSKVENSHM-SLDLFLGKIPNPPTSDKMVELEFTLSRGIRFEYMIHHPYWPLHGFFSA-HLFLQKYDRAQHFTQLATLSDLIFTHMPSQIALGCLLAAARETD-----------------------DPELRLEEVADDVTTQGDKAVN

1. **Multiple alignments for phylogenetic analysis of cyclin family proteins in H. sapiens, *T.* adhaerens, *T. trahens* and *D. discoideum.***

>AMSG_08210.2

QRDVWLLTASEA-DLGVGDSLDATVATLLGIYFPQKIQELGKRLHLPQVVIATAIVYFKRFYTRHTLETVSPFTMLAAAVFVACKVDECP-QKLASLVKQVTEIVTAEDVATTEISLLATLEYSLVVFHPYTALKDYVADAGLADELLETAWYIVNDSYCTAVFLYFPPYLIALAAIYMAGAINRAESSVAEWFGSMMVDMAEVHTVAAHLVTLYDTWSLPTDQRAKQRI

>Ddi-gi_66821868_

HCTEWLLRNKIESNPKDKQYLTPIELKKLRTHYCFVIQNLGNALKLRQRAISTAIVYFKRFYLKNSFVDCEPRLVAVTCLYLSSKVEECI-TQAKKCAAKMKEIYLMNDILECEFFVLEELDFCLIIYHPYKSLPFYLQSSGLDPASIEIIWGIVNDSYRTDVCLLYPPFVVGLGCILLGSYLLKKDI--KQWLSELNVEMKDIWEVSKDLIDYYEFEKQQSEDLLYNKL

>Tad-gi_196011367_

HYSQWVLRQEILGREEDLSYLSEDEIFKIHMFFANFIRHLGDLLKLRQQVIATAIVYFKRFYSRNSLKSIAPLLLAPTCILLASKAEECG-IITGRFINACTNVYKMPVILECEFFLLELLDCSLIVFHPYRPLLQFVEDFEKKDALLPCAWRAINDSYNTDICLMYPPYIIALACLHTACIIQSIDC--TQWFAELSVDLDLLFEVTRQIVALYELLKTYEEKSLLDKI

>Hsa-CyclinC

HYLQWILKQDLLERQKDLKFLSEEEYWKLQIFFTNVIQALGEHLKLRQQVIATATVYFKRFYARYSLKSIDPVLMAPTCVFLASKVEEFG-VVNTRLIAAATSVYRMNHILECEFYLLELMDCCLIVYHPYRPLLQYVQDMGQEDMLLPLAWRIVNDTYRTDLCLLYPPFMIALACLHVACVVQQKDA--RQWFAELSVDMEKILEIIRVILKLYEQWKNFDEATILSKM

>Hsa-CyclinH

QKRHWTFSEEQLVLPNDPVFLEPHEEMTLCKYYEKRLLEFCSVFKPPRSVVGTACMYFKRFYLNNSVMEYHPRIIMLTCAFLACKVDEFN-VSSPQFVGNLRESKALEQILEYELLLIQQLNFHLIVHNPYRPFEGFLIDLKTPEILRKTADDFLNRIALTDAYLLYTPSQIALTAILSSASRAGITESESLMLKENRTCLSQLLDIMKSMRNLVKKYEPPREAVLKQKL

>AMSG_07694.2

EALAALLAAERKSATPRLTSRHVRLNCKMRAIVVSWLMEVSAEYRLTPGTCHLAITLFDNYLAAANVPRNKLQLIGVTALFIAAKLEEVYPPTVSELADVTDRAYSEDEIRGAERALLATLRWELHPPTVPAFVAIYLDLDRS--AGFRAAMAAVDAVILDLRVTAIVASLMAAAAVYVISAGGVPAALVDTVEVIIRYADDNLLHLLAESQLVTDSGVRQHPVDHIQNF

>Tad-gi_196003740_

IIFENMKQREAQLVV-DYLERQNDITEQMRMILIDWLCEVQQNFELFHETLYLAVKIVDRFLSARVVSRDALQLIGATAMLMSSKIEERYPPLVDDFVYICDDAYSRQAVLDMERDICYALDFDLNIPIPYRFLRRYGKVASLSMENLTLARYILELTLQEYQFVTFKPSMLAAGCLCLALKMKNCGEWTQTLVHYSGYEESELNELVQKLNAMIAKPAPENCKVVKTKY

>Hsa-CyclinB3

-EIFSYMKEREEQFILDYMNRQIEITSDMRAILVDWLVEVQVSFEMTHETLYLAVKLVDLYLMKAVCKKDKLQLLGATAFMIAAKFEEHNSPRVDDFVYICDDNYQRSEVLSMEINILNVLKCDINIPIAYHFLRRYARCIHTNMKTLTLSRYICEMTLQEYHYVQEKASKLAAASLLLALYMKKLGYWVPFLEHYSGYSISELHPLVRQLNKLLTFSSYDSLKAVYYKY

>Tad-gi_196012973_

------------------------ITPTQREALISSMINLSRKHNIKRDTLFMAVNYLDRFLKVVTVTEDCFELVGLTCMMIACKVEECQPPKMEEFLTSCTHYYKKAEMKRLEIIILNYIDFRLSPPIAPHFLEYIIHFHQHHIELVNIANQVLLKILPTYRFNHIKSSILAASAFEYAK-------------------------------------------------

>AMSG_04018.2

DVYAALRENETRTAPLNYMAAQRDITPAMRAILMDWLVEVAEEYRLKSETLFLTINYIDRFLSKRNVKRCKLQLVGVAAMLVASKFEEIYPPLVKDFVFITDNTYSRAQVIQMETIILQTLKFELVVSTPLPFLVRYARAVDDPDEILSLARYYCECTIHSYDFLKYPPSLTAAASLCLALSTRGWNYWSPSLSYYSGFTSDDLQACILDLTGVLANEPSAQLQGVWEKY

>AMSG_03352.2

DIMDHFYSKELKYMVPNFMSVQSDVTPKMRSILLDWLVEVHLKFKALQETMYLTVNVMDRFLNKVQVKRSRLQLVGITSMLIASKYEEIYPPPIRDFMYMCDNAYTRREILDMEQTILATLNFTLQPPYPLHFLRRFSKAANSDLTTHTTAKYLMELTIPDYQAVEFLPSSIAAAAMCLALKMNRSGEWDATMEHYTRYTETDLLPIIRHLNSLHANSSTSSLKAVAKKY

>Ddi-gi_66819865_

EIFAYYREKEQIDKIDDYIKNQYHINERMRAILVDWMMAVHVRFKLLSETFFLSVNIVDRYLAKVMIPVTKLQLVGITAILLACKYEEIYSPQIKDFVHTSDDACTHAEVIDMERQILSTLQFHMSVATPLHFLRRFSKAAGSDSRTHSLSKYLSELSMVEYRMVQFVPSMIAAASIYVARRMTMKSYWNVTLEYYTCYKESEILQCAQELKEVRKRADTSNLKATRKKY

>Tad-gi_196005765_

DIHSYLRKAEYFHRPKDYMERQCDVNGTMRSILVDWLVEVSEEYKLRERTLYLAISYIDRFLSAMSVRRSKLQLVGTAALFIAAKFQEIYPPDCAEFAYITDDTYNIKQVLKMESLMLKVLSFNLSSPTAVDFLERYGSEAGLDSEIRELSMYLTELTLKDYGFLQFMPSLIAVSAVSLALHTFKLKYWPQELSTYTNYQWQQVSPCLNRIFEAFRLAHTQPQRAVVEKY

>Hsa-CyclinA2

DIHTYLREMEVKCKPKGYMKKQPDITNSMRAILVDWLVEVGEEYKLQNETLHLAVNYIDRFLSSMSVLRGKLQLVGTAAMLLASKFEEIYPPEVAEFVYITDDTYTKKQVLRMEHLVLKVLTFDLAAPTVNQFLTQYFLHQQANCKVESLAMFLGELSLIDAPYLKYLPSVIAGAAFHLALYTVTGQSWPESLIRKTGYTLESLKPCLMDLHQTYLKAPQHAQQSIREKY

>Hsa-CyclinA1

EIYQYLREAEIRHRPKHYMKKQPDITEGMRTILVDWLVEVGEEYKLRAETLYLAVNFLDRFLSCMSVLRGKLQLVGTAAMLLASKYEEIYPPEVDEFVYITDDTYTKRQLLKMEHLLLKVLAFDLTVPTTNQFLLQYLRRQGVCVRTENLAKYVAELSLLEAPFLKYLPSLIAAAAFCLANYTVNKHFWPETLAAFTGYSLSEIVPCLSELHKAYLDIPHRPQQAIREKY

>Tad-gi_196002535_

DIYKNMLKQEKRCTLDDYMTGQPVITKGMRAILLDWLVDVHLRYNFHPESLYLTTYIIDRYLQTTQVNRKKLQLVGIAAFYIAIKYEEIFLASTDDLLYLTENSYEINEFIQMEAKILKALDFSLSRPTSIHFLRRISKAASADIEQHTFARYLTEIALIEYSLLSYLPSQIAAAASLISLKIFD-KSWTPTLQYYSSYSEDSLKPVARQIAKLAWKSWTSKYQVRKC-I

>Hsa-CyclinB1

DIYAYLRQLEEEQAVRKYL-LGREVTGNMRAILIDWLVQVQMKFRLLQETMYMTVSIIDRFMQNNCVPKKMLQLVGVTAMFIASKYEEMYPPEIGDFAFVTDNTYTKHQIRQMEMKILRALNFGLGRPLPLHFLRRASKIGEVDVEQHTLAKYLMELTMLDYDMVHFPPSQIAAGAFCLALKILDNGEWTPTLQHYLSYTEESLLPVMQHLAKNVVMVNQTKHMTVKNKY

>Hsa-CyclinB2

DIYQYLRQLEVLQSINHFL-DGRDINGRMRAILVDWLVQVHSKFRLLQETLYMCVGIMDRFLQVQPVSRKKLQLVGITALLLASKYEEMFSPNIEDFVYITDNAYTSSQIREMETLILKELKFELGRPLPLHFLRRASKAGEVDVEQHTLAKYLMELTLIDYDMVHYHPSKVAAAASCLSQKVLGQGKWNLKQQYYTGYTENEVLEVMQHMAKNVVKVNETKFIAIKNKY

>Tad-gi_195999522_

KMVESCRNINIK-L---VNRIQLELNDRMRYILIDWLVEVAEMKEFSSEMLCNAIDLVDRYLEINPIPRSNLQLLGISCMVIASRYHCVDIMTIREAAWLTDNTYKYDEVVRMIGEVFAAVNGEIRTPSAFDYLKIFCTISEVSQKCTYLASFILELSWLFLENSRYKSAVKAAASLLLARVLIMGNPWTEELKSYTGLSLEDLSSCVLHLYKKCLAEKPSEVKSVHNRY

>Tad-gi_196003236_

EVWQVMIENDNNFKRSDCFDRHPNLVPNMRGILLDWMMEVCESFKMQRETFYMAMDYLDRYLSLSNILKQKLQLIGTTCLFIAAKIEEIQPPQVSEFAYVTDSACSEDDIIKLELQLLQTLEFQLSPVTVTSWLNVYVQLFNICGDLYMKAIRLIDLCILDSWCLMHSYRSIAASAFYLIAPS------KQLAIDCTGYLWENLTSCISWMMPKYETV--------KKYC

>Hsa-CyclinE2

EVWLNMLKKESRYVHDHFEVLHSDLEPQMRSILLDWLLEVCEVYTLHRETFYLAQDFFDRFMLTQDINKNMLQLIGITSLFIASKLEEIYAPKLQEFAYVTDGACSEEDILRMELIILKALKWELCPVTIISWLNLFLQVDALSQETFIQIAQLLDLCILAIDSLEFQYRILTAAALCHFTS-------IEVVKKASGLEWDSISECVDWMVPFVNVVKSTVKLKTFKKI

>Hsa-CyclinE1

EVWKIMLNKEKTYLRDHFLEQHPLLQPKMRAILLDWLMEVCEVYKLHRETFYLAQDFFDRYMATQNVVKTLLQLIGISSLFIAAKLEEIYPPKLHQFAYVTDGACSGDEILTMELMIMKALKWRLSPLTIVSWLNVYMQVAYLPQQIFIQIAELLDLCVLDVDCLEFPYGILAASALYHFSS-------SELMQKVSGYQWCDIENCVKWMVPFAMVIRETSKLKHFRGV

>AMSG_05464.2

ASSWYVTLDELKKHPSREDGMDYATECAERQKQCMAILHLGAKLNVPTLAKATAVLFFHRFFLRRSFKKHDAWLVAVTCLFLATKVEESS-KKLRNVVDMAFEQQLKEQILIHERKVLQTLAFDLTVVHPYKDLMPMASDIHASRELAQSAWSFINDSLSMTLCLRYQPREVAAAAIYLSALHQKVNDEGKSWIDIVGVPLKTLEMISNEILDMYARHGGTVVAQGSAGA

>Hsa-CyclinT1

NKRWYFTREQLENSPSRRFGVDPDKELSYRQQAANLLQDMGQRLNVSQLTINTAIVYMHRFYMIQSFTQFPGNSVAPAALFLAAKVEEQP-KKLEHVIKVAHTCQQVQDLVILESIILQTLGFELTIDHPHTHVVKCTQLVRASKDLAQTSFMATNSLHLTTFSLQYTPPVVACVCIHLACKWSNWEPKHWWEYVDATVTLELLDELTHEFLQILEKTPNRLKRIWNWRA

>Tad-gi_196013340_

VTNFYFSDTELQNTPSRRNDISVATELYYRQTCALCIQELGMKLGANQLTINTALVYMHRFYMFHSLASYNLKNIAACAIFLASKSEEHP-NKLNKVITAAYEYKLSQDLVDNEYAMFFTTGFDIEIMHPHTHVIKCLHGLKGKCIIFHSFYNILWI-------------------------------------------------------------------------

>Tad-gi_196007480_

-SSWYFCSNEIVNSPSRSDGIEITAECRYRREGARFIMDVGNRMNLRYETVATGIVFYHRFYMMHSFKTINRLIGAAACLYLAGKAEETP-KKCRDLVKAVRTIDPKEEIISHERLLLQTIKFDLCVQHPYKYIVKFAKNLKDDEKVVQMAWNFVNDSLSTTLCLQWKPQVVAVSLLHLAAKLSKYNSKSWWQHFLPEINSDVLEDICLQMLDFYDKTDVGASNYNMISP

>Hsa-CyclinK

KPCWYWDKKDLAHTPSQLEGLDPATEARYRREGARFIFDVGTRLGLHYDTLATGIIYFHRFYMFHSFKQFPRYVTGACCLFLAGKVEETP-KKCKDIIKTARSLDPKEEVMVLERILLQTIKFDLQVEHPYQFLLKYAKQLKGDQKLVQMAWTFVNDSLCTTLSLQWEPEIIAVAVMYLAGRLCKFEQRRWWEQFVQDVPVDVLEDICHQILDLYSQGKQQMPHHTPHQR

>Ddi-gi_66808797_

--NLYFSDEEIQNSPSRKDGISFEIEDNLRRYGTEVIQEAGILLELPQATTVTGQVIFQRFYCRKSLKEYDVKTLAMGSLFVSTKFTEPQ-RKIRDILNVFTLIDLKGDVIAAEFDILKEFGFLMYVDLPHKYILNYMKLLERSKELAQKSWNYLNDSMRTTITIQYKPESIAASSIFLASRILGTQPEPYPWWELFDTTKEEIELISFEINNLYSKPSAYYIDVFNP-T

>Ddi-gi_66807697_

NSHWFFSKEQVLKHYSL--GIELKLEVAYRRASAAFIQDVGIKLKMPQLTIATAISYFHRFFIRHQLINIDSPVVATACLFLAGKVEETP-RKLDDVIKVSYMINLKNKILQNEHLILTTIAFELAVEHPYKYLLEYMKSIQGSKNLCQVAWNFVNDSLRTSLCLHYPPDLISYASIYLATRFLNYQINKKEWWEMLGIKFEVLEDISKQILDLYEANPLQQTATIPSSS

>Hsa-CABLES1

TVIDYVKPSDLKKDMNEFKEKFPHIKSKIRSLKREMRKLAQEDCGLEEPTVAMAFVYFEKLALKGKLNKQNRKLCAGACVLLAAKIGSDLKHEVKHLIDKLEEKLNRRELIAFEFPVLVALEFALHLP------------------------------------------------------------------------EHEVMPHYRRLVQS---S------------

>Hsa-CyclinL1

IDHSLIPEERLSPTPSMQDGLDLPSETDLRILGCELIQAAGILLRLPQVAMATGQVLFHRFFYSKSFVKHSFEIVAMACINLASKIEEAP-RRIRDVINVFHHLNTKNQVIKAERRVLKELGFCVHVKHPHKIIVMYLQVLECNQTLVQTAWNYMNDSLRTNVFVRFQPETIACACIYLAARALQIPPTRPHWFLLFGTTEEEIQEICIETLRLYTRKKPNYELLEKEVE

>AMSG_04120.2

-------------------------------------------------------------------------------MLVAGKMFEKKVPSLETLNQLAGYQYTPDAILAMEGNILHTLSWKLRVVTPLFFWGYFASIGVCPALRKVSHTILAEICLLSMAFLDCMPSMTASAALLVARNKLGITDWAPRFQVRIGYSRSDVAVSAAKLSLLFDEKFPSGSPSLTTPP

>AMSG_02061.2

EYVVNLLAMETRYAES--HASVRAAHVPLRYNAVVWLLRVAEAQALGSETFFHAVALMDRMEAAIKVRPKHVRLVAGAALLIACKVREPEGKTCFDLIAALVP-CPTVRLRRMEALVLHHMGWRVNDVTPHLFVESLLPSLAAPEALRTRAHFYIELACHDASLVTTYPSVLAVASLALAASDTSIAGPLDGASAALSADPAAVAAAVSALRTALKSMAQRGTLILAPHT

>Hsa-CyclinF

EIVCQLFQASQAVSKQQVFSVQKGLNDTMRYILIDWLVEVATMKDFTSLCLHLTVECVDRYLRRRLVPRYRLQLLGIACMVICTRFISKEILTIREAVWLTDNTYKYEDLVRMMGEIVSALEGKIRVPTVVDYKEVLLTLVPVELRTQHLCSFLCELSLLHTSLSAYAPARLAAAALLLARLTHGQTPWTTQLWDLTGFSYEDLIPCVLSLHKKCFHDDAVSLTAVKQRF

>Hsa-CyclinO

SCYAFRKAQESHFHPREALARQPQVTAESRCKLLSWLIPVHRQFGLSFESLCLTVNTLDRFLTTTPVAADCFQLLGVTSLLIACKQVEVHPPRVKQLLALCCGAFSRQQLCNLECIVLHKLHFTLGAPTISFFLEHFTHARVEALEAQALARGVAELSLADYAFTSYSPSLLAICCLALADRMLRVSPVDLRLGDHPEAALEDCMGKLQLLVAINSTSLTHLPVQICEKC

>Hsa-CyclinI2

RLLCHLQLAQDREARLWRGGKPQDEICDAFEEVVLWLLRLQNTFYFSQSTFNLALTIFGRLLISVKVKEKYLHCATITSLRLAAKVNEEEIPQVKDFTKHYGSDYSPNELLRMELAILDRLHWDLYIGTPLDFLTIFHALVVLSSLHVASLTRQLQHCMAGHQLLQFKGSTLALVIITLELERLMPGAPISDLLKKAQVGDMQYSCCKELVMQQLRSLQS---SSCTDNF

>Tad-gi_196000815_

-------GMPPVVKISAQQLIEDKDHWPEYLKIGKFIAESGIKLKLGSVVIARAATIYHRFYFLCDISQFDRYLVAVTCLYLASKVEDTP-RRARDVITTSYKVQLRDSVVNFELFMLRMLKFDVSSELPHKYLLHYLKSLQDWNHINQLCWQLLQDTSLLPFILLYPPSVIATAVIYLAVKCNNIEPKPWWNVFSPNLNEEGLQQLCYKFMELYDT-------------

>Hsa-Fam58B

----MGEEAGEDAGEDAREGA-AAPAARVHFRVARFIMEAGVKLGMQSIPIATACTIYPKFFCETILDAFDPYLIAMSSIYLAGKVEEQP-LWAHDIISVSNRYELRDSIVQRELLMLRVLRFQVSFQHPHKYLLYYLVSLKNWTPVAVTAWALLRDSYHGGLCLRFQAQHIAVVVLYLALQVYGVEPKLWWQAFSDDLTKPIIDTIVSDLIQIYTIDTEIP--------

>AMSG_02174.2

AKHWLFTEDTILMRPADDAFPSVEDETVLVDFYAQQMWVLCKKVELPPKVFGCAVIFLKRFYLKTSVLEYVPKEVMLTALVLAAKSEEVH-IAPSAFAAKF---VKPEAIIALEIMMLEKLSFHLMVYQVWHPIGGFVDMFRRLQTLRDEAMEWARDAMLSDLILQYPPSQLALAATCLAADEAGLDD-TFLAHQFPGPENDALRATIRELENALDTASQTTLDALSKHL

>Tad-gi_195998946_

RKYWTFPNEEEIHKIRTKDLPSVEELDHLCTFYEFELMDLCRRFDPP--MPATAAVYMKRFYLVCSVMDYHPCDIMLACVYLATKVDEYN-ISIDKFLTMVPENRAKSRTLGFELLVMEKLKFHLTIHCPYRPVEGLLINIMTVDELRRHIDKFLSKILYCQAMLIYPPSQVGEIALFPSQIALAAIE-A--------GEKAGLDLFESVISAVLNNNS-----------

>Tad-gi_196001479_

RVLQNLLKLEENYLPNDYFNVQREIKPFMRCMVTNWMLDVCHGENCNDKVFPLAVNYLDRFLSMVSIRKIRLQLVGSVCLFLASKLKDRIPLTAEKLCSWTDYSVTCQELLDWELLLLEKLKWDLGSVTPLDFLDQILYRLSFEHMLKKHAYTLIALCCTDFQLCTNPPSMIAGGCILCALAGVLPQDVNRIVQKITSIEPDYLICCKEQVEDLLSKTNSSDQIDGANGY

>Hsa-CyclinD1

RVLRAMLKAEETCAPSSYFCVQKEVLPSMRKIVATWMLEVCEEQKCEEEVFPLAMNYLDRFLSLEPVKKSRLQLLGATCMFVASKMKETIPLTAEKLCIYTDNSIRPEELLQMELLLVNKLKWNLAAMTPHDFIEHFLSKMPEAQIIRKHAQTFVALCATDVKFISNPPSMVAAGSVVAAVQGLNLRPLTRFLSRVIKCDPDCLRACQEQIEALLESSLRQAQQNMDPKA

>Hsa-CyclinD3

RVLQSLLRLEERYVPRSYFCVQREIKPHMRKMLAYWMLEVCEEQRCEEEVFPLAMNYLDRYLSCVPTRKAQLQLLGAVCMLLASKLRETTPLTIEKLCIYTDHAVSPRQLRDWEVLVLGKLKWDLAAVIAHDFLAFILHRLSLPALVKKHAQTFLALCATDYTFAMYPPSMIATGSIGAAVQGLGACMLTELLAGITGTEVDCLRACQEQIEAALRESLREASQTSSSPA

>Hsa-CyclinJL

DVHCTLREKELKLP--T-F-RAHSPLLKSRRFFVDILTLLSSHCQLCPAARHLAVYLLDHFMDRYNVAHCNLHLPGSSNSPASAPHPPPTPPQVAETTGKFEDRLTKKELLSTELLLLEAFSWNLCLPTPAHFLDYYLLASVSQECLKEYAHYFLEVTLQDHIFYKFQPSVVAAACVGASRICLQLSYWTRDLQRISSYSLEHLSTCIEILLVVYDNVLKDAVAVKSQAL

>Hsa-CyclinJ

DIHQALRYKELKLP--S-Y-KGQSPQLSLRRYFADLIAIVSNRFTLCPSARHLAVYLLDLFMDRYDISIQQLHLVALSCLLLASKFEEKEVPKLEQLNSLGCMTLTKQNLLHMELLLLETFQWNLCLPTAAHFIEYYLSEAVHELYMAKYADYFLEVSLQDYAFLNYAPSLVAAACVASSRIILRLSTWPTRLHRLTAYSWDFLVQCIERLLIAHDNDVK--EANKQRGQ

>Ddi-gi_66817430_

ALLYHIERGSQFPQKTEIFSKIDLKSNPTVETIYKFIRDIFKAEKLDSECAIMCLAYIERIITYTGISSINWRRIVLSALILASKVWEDQSVWNVDFLPVFDN-LTAADLNCLERQFLAMLQYNVSLNA-SIYAKYY--------------FELRNFSKLDSNQFPLKPLDK-------------------SGVR----RLEDHSKASEYRVKPFKRSA-----------

>Hsa-CyclinY

DIFDENLHPLSKSE----VPPDYDKHNPEQKQIYRFVRTLFSAAQLTAECAIVTLVYLERLLTYAEICPANWKRIVLGAILLASKVWDDQAVWNVDYCQILKD-ITVEDMNELERQFLELLQFNINVPS-SVYAKYY--------------FDLRSLAEANNLSFPLEPLSR-------------------ERAH----KLEAISRLCEDKYKSAKRSA-----------

>Ddi-gi_66817546_

DHYSNLINKQYEYQDRTYNTSKSPCASHCRTDVIDFLVDLTSTIRMNKRTLQMTISIYDDFMDYIVGEVDVMQLVAVSSLSIASKLEELEQPSIRELNYSTNNSYTHDGIVKTEMGILSILKYKVMYPTPSHFLDYLLLITIQPNQFKLINDQILNISYKNLKYRQYLPSIIACASIASTRTIMGIEAWKPSLRKISNIPMREIYPLYIIFIKEYNTLYKIIPEFISKKF

>Ddi-gi_66826993_

QCNNQHKQVILEKTPSEPNILSPDDELSLIHYYETKTLEIAMALNLPDKVSAPAIIYIKRFYLKNSIMQYGAKLVMLSCLFIACKTEDNH-LDIDYYSNITK--ASPSDITNLEIIILESLNFNLIVYHPFRPMYGYILDINDNDTLWETCKKSIQKSLFSDCCFEFHPQIIALACLNLNWDGFNMYCINNNNNNNNNNNNNNNNNNNNNL-------------------

>Ddi-gi_111226646_

EIFDNARKNQWKTQPTDYMQNQSELKPGMRAILIDWIVDIGCELGVKNETIYLSINILDRYLSLQPVTRNEFQMIGACAFFIAAKYEEYKGAQPQFIIQSAGEFFNVDQLLECECKMLKTLNFSLCTPTIKFFLGRYLIAVGD-SDISHVAHLFGELSLLEYNLINYPPSVIAAACVYLACLVLQKQ-WTTTLTYYCRVEVNDFQKCVRFIYDKFQSNESTYSRTIKSKY

>Ddi-gi_66811552_

DIKNFYIYHQKNVNNS--EEQDWKRFNNCFKRMLVFMRKVCDSLSLSSLAYLHSISLLKRFLNRKFIFDYNQYFVSMTCILICIKVNSSSPMKVRDLLNVSYYILFKDNILSMEQKILQILSYDISVSFKENFAGIYNNNNNNNHVLSQISFNFLSDSSLIIESPFFKAPEIALFCILLAFEYTKIDPSRNNLFLILGLKINNNNNNNNNNNNNNNNNNNDQKYEIFKNY

>Hsa-CyclinI

LLEKAITREAQMWKVNRKMPSNQNVSPSQRDEVIQWLAKLKYQFNLYPETFALASSLLDRFLATVKAHPKYLSCIAISCFFLAAKTVEERIPVLKVLARDSFCGCSSSEILRMERIILDKLNWDLHTATPLDFLHIFHAIAVSTSQHLAVLTKQLLHCMACNQLLQFRGSMLALAMVSLEMEKLIPDLLTIELLQKAQMDSSQLIHCRELVAHHLSTLQSSLPLNSVYVY

>Hsa-CyclinG1

LLHQLNALLEQECQPKSAHDNGLRMTARLRDFEVKDLLSLTQFFGFDTETFSLAVNLLDRFLSKMKVQPKHLGCVGLSCFYLAVKSIEENVPLATDLIRISQYRFTVSDLMRMEKIVLEKVCWKVKATTAFQFLQLYYSLLQENSINFERLEAQLKACHCRIIFSKAKPSVLALSIIALEIQAQKCVLGIECLQKHSKINGRDLTFWQELVSKCLTEYSSNKGTARQLKH

>Hsa-CyclinG2

GLLNVYLEQEERFQPRATPENDNTLCPGLRNAKVEDLRSLANFFGSCTETFVLAVNILDRFLALMKVKPKHLSCIGVCSFLLAARIVEENIPSTHDVIRISQCKCTASDIKRMEKIISEKLHYELEATTALNFLHLYHTIILCHILSLDKLEAQLKACNCRLIFSKAKPSVLALCLLNLEVETLKSVLILLLVKKHSKINDTEFFYWRELVSKCLAEYSSPECCKPDLKK

>Hsa-CyclinT2

SSRWFFTREQLENTPSRRCGVEADKELSCRQQAANLIQEMGQRLNVSQLTINTAIVYMHRFYMHHSFTKFNKNIISSTALFLAAKVEEQA-RKLEHVIKVAHACQQTQELVILETIMLQTLGFEITIEHPHTDVVKCTQLVRASKDLAQTSFMATNSLHLTTFCLQYKPTVIACVCIHLACKWSNWEPKHWWEYVDPTVTLELLDELTHEFLQILEKTPNRLKKIRNWRA

>Hsa-CABLES2

TVIEYVKPSDLKKDMNEFREKFPHVKSKIRSLKREMRSLS-EECSLEPVTVAMAYVYFEKLVLQGKLSKQNRKLCAGACVLLAAKISSDLKSGVTQLIDKLEERFNRRDLIGFEFTVLVALELALYLP------------------------------------------------------------------------ENQVLPHYRRLTQQ---F------------

>Tad-gi_196007260_

LENTIYPDEKLQETPSQLDGLSKEMETNLRIAGCEYIQAAGILLKLPQVAMATAQVLYQRYYYSKSFVKYNYEICAMACIFLAAKIEEHP-RRIRDVVNVFYHIHKKSEVIKAERRVLKELGFCVHVKHPHKCIVTYLKILECNTELIQKAWNYMNDSLRTDVFMRYTPETIACACIYLSARQLGIGPSNPPWYEVFDATTKEM-EVYLLIVSV--------------ID

>Hsa-CyclinL2

LENCLLPDDKLRFTPSMSSGLDTDTETDLRVVGCELIQAAGILLRLPQVAMATGQVLFQRFFYTKSFVKHSMEHVSMACVHLASKIEEAP-RRIRDVINVFHRLNLKNQIIKAERRVLKELGFCVHVKHPHKIIVMYLQVLECNQHLVQTSWNYMNDSLRTDVFVRFQPESIACACIYLAARTLEIPPNRPHWFLLFGATEEEIQEICLKILQLYARKKVDLTHLEGEVE

>Hsa-Fam58A

----PG-----G-GPAAREGQ-PAPEARVHFRVARFIMEAGVKLGMRSIPIATACTIYHKFFCETNLDAYDPYLIAMSSIYLAGKVEEQH-LRTRDIINVSNRYELRDSIVQCELLMLRVLRFQVSFQHPHKYLLHYLVSLQNWTPVAVTAWALLRDSYHGALCLRFQAQHIAVAVLYLALQVYGVEPKPWWQVFNDDLTKPIIDNIVSDLIQIYTMDTEIP--------

>Hsa-CyclinD2

RVLQNLLTIEERYLPQSYFCVQKDIQPYMRRMVATWMLEVCEEQKCEEEVFPLAMNYLDRFLAGVPTPKSHLQLLGAVCMFLASKLKETSPLTAEKLCIYTDNSIKPQELLEWELVVLGKLKWNLAAVTPHDFIEHILRKLPQQSLIRKHAQTFIALCATDFKFAMYPPSMIATGSVGAAICGLQQDELTELLAKITNTDVDCLKACQEQIEAVLLNSLQQYRQDQ----

>Tad-gi_196013209_

D-IFDEELHPLMRP----VRKETIINLPEHRHVYKFFKTLFSSAQLTAECAIISLIYVERLMEYAEIHPSNWRRVVLGAILLASKVWDDQAVWNIDYCQILKD-TTVEDMNALEREILQLIMFNINVPS-SIYAKYY--------------FDLRTVADENNYILPTEPLST-------------------ERAR----KLE-V--------------------------

>Hsa-CyclinYL

------------------VPEEYFKHDPEHKFIYRFVRTLFSAAQLTAECAIVTLVYLERLLTYAEICPTNWKRIVLGAILLASKVWDDQAVWNVDYCQILKD-ITVEDMNEMERHFLELLQFNINVPA-SVYAKYY--------------FDLRSLADDNNLNFLFAPLSK-------------------ERAQ----NLEAISRLCE-D-KAARRSF-----------

9.**Multiple alignments for phylogenetic analysis of CDK4/6, CDK1/2/3, and CDK subfamily proteins in *H. sapiens*, *N. vectensis*, *T. adhaerens*, *A.queenslandica*, *M. brevicollis*, and *S. rosetta*.**

>Hsa-cdk6

YECVAEIGEGAYGKVFKARDLKGGRFVALKRVRVQTGEEGMPLSTIREVAVLRHLEHPNVVRLFDVCTVETKLTLVFEHVDQDLTTYLDKVPEPGVPTETIKDMMFQLLRGLDFLHSHRVVHRDLKPQNILVTS-SGQIKLADFGLARIYSFQM-ALTSVVVTLWYRAPEVLLQS-SYATPVDLWSVGCIFAEMFRRKPLFRGSSDVDQLGKILDVIGLPGEEDWPRDVALPR--Q-AFHSKSAQPIEKFVTDIDELGKDLLLKCLTFNPAKRISAYSALSHPYF

>Hsa-cdk4

YEPVAEIGVGAYGTVYKARDPHSGHFVALKSVRVPNGGGGLPISTVREVALLRRLEHPNVVRLMDVCATEIKVTLVFEHVDQDLRTYLDKAPPPGLPAETIKDLMRQFLRGLDFLHANCIVHRDLKPENILVTS-GGTVKLADFGLARIYSYQM-ALTPVVVTLWYRAPEVLLQS-TYATPVDMWSVGCIFAEMFRRKPLFCGNSEADQLGKIFDLIGLPPEDDWPRDVSLPR--G-AFPPRGPRPVQSVVPEMEESGAQLLLEMLTFNPHKRISAFRALQHSYL

>Nve-gi|156374066|

YEEVAEIGTGAYGTVYKAKDLLDGKFVALKRVRIQNSEEGMPLSTIREIALLKQIAHPNVVRLLDIFHIETHLNLVFEHVDQDLAAYLEYCPQPGLGEWKIKDLTYQILNGVDFLHTHRIVHRDIKPQNILVTK-DGQVKIADFGLARVYKDAM-ALTSVVVTLWYRAPEVLLQS-SYATSVDIWSVACILAELFNRRPLFEGKNDVDQLDKIFSIIGSPSQDEWPQNVSLPW--T-SFSRYTTGSFQALVPEMCTEGTTLLKEMLQFLPRSRPSASEAMNHPFF

>Tad-gi|195999760|

YVQVTEIANGAYGRVYKARDLNHNRLVALKKIAVINDEQGIPISTIREITSLKSLGHQNIVRLYDIFANTMDLTLVFEHVEQDLQTYIRNCPSAGIDTRKIKDIIYQIVNAIDFLHANRIVHRDLKPQNVLISR-QGIVKVADFGLAKVFCENV-PITSVVVTLWYRCPEVLLQS-TYATPVDMWSVGCIMAELYLREPLFCGQTDIDQLQKIFSMTGLPDESEWPVNIPFSR--S-TFCQYTRRPVSEMMPEICQDGVDLLQKLLKFNPKERLTAEESLQHPYF

>Hsa-CDK5

YEKLEKIGEGTYGTVFKAKNRETHEIVALKRVRLDDDDEGVPSSALREICLLKELKHKNIVRLHDVLHSDKKLTLVFEFCDQDLKKYFDSCNG-DLDPEIVKSFLFQLLKGLGFCHSRNVLHRDLKPQNLLINR-NGELKLADFGLARAFGIPVRCYSAEVVTLWYRPPDVLFGAKLYSTSIDMWSAGCIFAELANGRPLFPGNDVDDQLKRIFRLLGTPTEEQWPSMTKLPDYKP--YPMYATTSLVNVVPKLNATGRDLLQNLLKCNPVQRISAEEALQHPYF

>Nve-gi|156407302|

YDKLEKIGEGTYGTVFKGKNKETREILALKRVRLDDDDEGVPSSALREICLLKELKHNNIVRLYDVLHSEKKLTLVFEFCDQDLKKYFDSCQG-EVDASVVKSFMFQLLRGLAFCHSHNVLHRDLKPQNLLINK-DGELKLADFGLARAFGIPVRCFSAEVVTLWYRPPDVLMGAKLYSTSIDMWSAGCIFAEMANGRPLFPGNDVDDQLRRIFKILGTPTEESWPNVSKLPDYKE--FPPQPSVSLGMVVPKLSSTGRDLLQKLLVSNPAHRISAEDAMKHAYF

>Aqu-gi|340384408|

YERLEKIGEGTYGTVFKAKEKESGEIVALKIVRLDEDDEGVPSAALREICLLKELKHKNIVRLTDVLHKNLKLTMVFEYIDQDLKKYFDVSGG-IISPQVVQSFFFQLLQGLAFCHYNNILHRDLKPQNILISK-KGDLKLADFGLARAFGIPVRLFSAEVVTLWYRPPDVLMGAQVYNTSIDMWSAGTIFAELANGRPLFPGSDVDEQLKRIFKLVGTPTERSWPGLTKLPEFKE--FPPYPPACIESVVPALNDAGVDLLQRHLICHPTERISAEEAMRHEYF

>Tad-gi|196000717|

YEKLVKIGEGTYGTVYKAVNHDTGEIVALKKVRIDDENEGIPSFALREICLLKELKHKNIVMLYDVIHGNKELMIVFEYCDQDLKRYCDACQG-KIDPSIVQSFTNQLLQGLAYCHSHHILHRDITPQNILVTG-NGDIKLADFGLARNFGIPVKSFSAEVVTLWYRSPDVLLGATLYDTSIDIWSTGCIFAELSNGQPLLPGKDVADQLKIIFKIFGTPNEQIWPGVSQLMKDKD--YPSYNAMSILHVVPNLNQLGCDLFQLMMVLDPSKRCTAEQALQHAYF

>Mbr-gi|167522771|

YAKIDKLGEGTYGVVFKARDRHDGSIVALKRISLESAAEGVPSNAVREISLLKSLHHPNIVRLYDVLHSEHKLTMVFEYCDQDLKKFLDSCRG-TPEHHVIQSFMFQLLQGIRHCHEERVLHRDLKPQNLLINK-RGQLKLADFGLARPYGVPVRSYSHEVVTLWYRAPDVLLGATGYDTSIDMWSAGCILAEMANGSPLFPGTSVQDQLDLIFRVLGTPTIESWPGLHELPNYSG-PFPHVDGVGLEAEVSSLFPEGLDLLQQLLRYVPDERLSADRALRHRFF

>Sro-gi|326426444|

YVRICKVGEGTYGQVYKAQDRQNSQIVAIKAITLNGGAEGVPSNAVREISLLKRLDHPNIVRLLDVLYSQTRLMMVFEFCDQDLKQFLSHKPI-QHDPNLAQTIMFQLLRAVAYIHSQHILHRDLKPQNILLDR-KGRLKLADFGLARPIGVPVSKYTADVVTLWYRAPDVLLGSEHYGPCIDLWSTGVIFAEVLTGQPPFQGNSINEQLLLIFDMLGTPPP-SWDM-RQYERYKD-FAPVLTSVGLETFLSRAAPVAVDLVKALLRYNPDARITAEAAMEHEFF

>Hsa-CDK3

FQKVEKIGEGTYGVVYKAKNRETGQLVALKKIRLDLEMEGVPSTAIREISLLKELKHPNIVRLLDVVHNERKLYLVFEFLSQDLKKYMDSTPGSELPLHLIKSYLFQLLQGVSFCHSHRVIHRDLKPQNLLINE-LGAIKLADFGLARAFGVPLRTYTHEVVTLWYRAPEILLGSKFYTTAVDIWSIGCIFAEMVTRKALFPGDSEIDQLFRIFRMLGTPSEDTWPGVTQLPDYKG-SFPKWTRKGLEEIVPNLEPEGRDLLMQLLQYDPSQRITAKTALAHPYF

>Hsa-CDK2

FQKVEKIGEGTYGVVYKARNKLTGEVVALKKIRLDTETEGVPSTAIREISLLKELNHPNIVKLLDVIHTENKLYLVFEFLHQDLKKFMDASALTGIPLPLIKSYLFQLLQGLAFCHSHRVLHRDLKPQNLLINT-EGAIKLADFGLARAFGVPVRTYTHEVVTLWYRAPEILLGCKYYSTAVDIWSLGCIFAEMVTRRALFPGDSEIDQLFRIFRTLGTPDEVVWPGVTSMPDYKP-SFPKWARQDFSKVVPPLDEDGRSLLSQMLHYDPNKRISAKAALAHPFF

>Nve-gi|156385093|

FEKVEKIGEGTYGVVYKARDKTTGRMVALKKIRLDTESEGVPSTAIREISLLKELNHPNVVSLLDVVHNQKSLYLVFEFLSQDLKKYMDCLPPSGISTSLIKSYVYQLLSGVAYCHSHRVLHRDLKPQNLLIDK-NGAIKLADFGLARAFGVPVRSYTHEVVTLWYRAPEILLGSRYYATPVDVWSIGCIFAEMKTRRALFPGDSEIDQLFRIFRTLGTPDDKVWPGVSELPDYKT-SFPKWPVQSIRHVLPTLDNTAIDLLQKMLTYQPNARISAKAALSHEFF

>Aqu-gi|340379293|

YERLEKIGEGTYGVVYKARQATHGRVVALKKIRLDAECEGVPSTAIREISILKELDHVNIVSLLDVLYCDRKLFMVFEFLDYDLKKYMDRHAPTG----IPTDYLYQLLEGVAYCHAHRVLHRDLKPQNLLISS-DGRIKLADFGLARAFGVPVRTYTHEVVTLWYRSPELLLGSQYYSTPVDIWSIGCIFAEMVTKRPLFPGDSEIDQLFRIFRTLGTPDESVWPGISSFPDYKS-SFPKWPRQNLQRIVKSLDTLGINLLEQMLCYEPCKRITAINGMRHPFF

>Tad-gi|196013348|

FHKLEKIGEGTYGKVYKACNKITGQTVALKKIRLDSDKEGVPSTTLREISILRSLNHSFVVRLYDVVHSDQCLYLVFEYLDHDLKHYLDHAY--KIPPALLKSYLYQMLRAISYCHSRRVLHRDLKPQNLLIDS-TGTLKLADFGLARIFGLPVRQYTHEVITLWYRAPEILLGSTYYSTPVDIWSIGCIFVEMINRRPLFAGDSEIDQLFRVFRTLGTPDEITWPGVSEMSDYKS-TFPKWPSRDLNSVIYSHDEDCVDLIKQMLVYEPNGRISARLALQHPYF

>Hsa-CDk1

YTKIEKIGEGTYGVVYKGRHKTTGQVVAMKKIRLESEEEGVPSTAIREISLLKELRHPNIVSLQDVLMQDSRLYLIFEFLSMDLKKYLDSIPPGYMDSSLVKSYLYQILQGIVFCHSRRVLHRDLKPQNLLIDD-KGTIKLADFGLARAFGIPIRVYTHEVVTLWYRSPEVLLGSARYSTPVDIWSIGTIFAELATKKPLFHGDSEIDQLFRIFRALGTPNNEVWPEVESLQDYKN-TFPKWKPGSLASHVKNLDENGLDLLSKMLIYDPAKRISGKMALNHPYF

>Tad-gi|196003954|

YLKIEKIGEGTYGVVYKGKNRNTQQLVALKKIRLENEEEGIPSTAIREVSLLKELKHPNIVDLIEVLYEESKLYLVFEFLDMDLKRYLDTLPKGTIDAMLMKSYLYQILLGVVYCHSHRVLHRDLKPQNLLINS-KGCIKLADFGLGRAFGVPVRVYTHEVVTLWYRAPEVLLGSTRYSCPLDIWSTGTIFAEMWLRRPLFQGDSEIDELFRIFRILGTPDDDIWPGVSSLPEFKS-SFPKWSKQSYDTFVPNMSESGIDLLSKMLIYDPANRISGKRALSHPYF

>Aqu-gi|340381019|

YTKLEKIGEGTYGVVYKARHKVTGKTVALKKIRLENEEEGVPSTAIREISILKEVQHTNVVKLEDIIHQDLKLYLVFEFMCMDLKKYLDSLPAGFMEPDLVKSYTYQILKGIVFCHGRRIIHRDLKPQNLLIDN-NGGIKIADFGLGRAFGIPVRAYTHEVVTLWYRAPEVLLGCPRYSCPLDIWSIGCIFAEMSNKKPFFQGDSEIDQLFRIFRILGTPSDAIWPSVTTMPNFKS-TFPKWTGKSLSQLCPHLDSIGCDLLMQMVIYNPGKRISAKRAMEHPYF

>Nve-gi|156391793|

FSKIEKIGEGTYGVVYKAKNLKTGGFAALKKIRLEVEDEGIPSTAVREISLLKELRHPNVVELQHILHQEPKLYLVFEYLTCDLKKHLDTTRG-MLDKTLVKSYLYQITNAIYFCHARRILHRDLKPQNLLIDS-KGLIKLADFGLGRAFGIPVRAYTHEVVTLWYRAPEVLLGGQRYSCPIDVWSIGTIFAEMVTKRPLFHGDSEIDQLFRIFRILGTPTEETWKGVTSLPDYKP-TFPKWAGDGLKKAVPQLDSDGLDLLKKMLIYDPALRISAKTSLKHPYF

>Mbr-gi|167517533|

YLKIEKIGEGTYGTVYKAKVKATGNLVALKKIKLEAEEEGVPSTAIREISLLKELSHPNVVSLMEVIHSENKLYLVFEFLDQDLKKHIDSQRN-GLSMELIKSYMLQLLKGIDFCHARRILHRDLKPQNLLINR-EGFIKLADFGLARAFGIPIRAYTHEVVTLWYRAPEILLGQRQYACPVDMWSIGCIFAEMVTRRPLFPGDSEIDELFRIFRVLGTPTEQTWPGVSQLPDYKD-CFPRWSGEGLASLIPGLDAMGLDLLQKMLRYEPSQRISARQALTHPWF

>Sro-gi|326436992|

YEKIEKIGEGTYGTVYKAKLITSGELVALKKIKLETEEEGVPSTAIREISLLKELNHRNVVRLIEVIHSEHDLHLVFEFLDCDLKKHMEVSR--QLAPDLVRSYLFQLLKGIEFCHTHRILHRDLKPQNLLIDS-DGNIKIADFGLARAFGIPVRAYTHEVVTLWYRAPEILLGARQYACPVDIWSIGCIFAEMVTTRPLFPGDSEIDELFRIFRYLGTPNEHVWPGVSELPDFKT-TFPQWKRQDLAKLVPGLDPTGLDLLEQMLRYAPSARISATRALRHPYF

>Sro-gi|326436991|

FEKTEKLGEGTYGSVYKAIDKTTMAVVALKKIKLNDQEFGVPASALREIALLRELDHPNIVQLLDVIPSSSELHLILEYVYEDLRKFMHRVK--VLERPMYQSFLRQLLLGLEYCHIHRILHRDLKPENLLINHRTGALKLADFGLARAFGIPVRAYTHEVVTLWYRAPEILLGSKQYACPVDMWAVGCIFAEMASSKPLFPGDSEVDQIMRIFRYLGTPTEKTWPGVSNLPDFRA-NFPRFPAIDLAPIVPQMDPVSMALLQHMLVYLPASRIPANQALKHPFF

>Hsa-cdk17

YIKLEKLGEGTYATVYKGRSKLTENLVALKEIRLEH-EEGAPCTAIREVSLLKDLKHANIVTLHDIVHTDKSLTLVFEYLDKDLKQYMDDCGN-IMSMHNVKLFLYQILRGLAYCHRRKVLHRDLKPQNLLINE-KGELKLADFGLARAKSVPTKTYSNEVVTLWYRPPDVLLGSSEYSTQIDMWGVGCIFFEMASGRPLFPGSTVEDELHLIFRLLGTPSQETWPGISSNEEFKNYNFPKYKPQPLINHAPRLDSEGIELITKFLQYESKKRVSAEEAMKHVYF

>Nve-gi|156403969|

YTKLDKLGEGTYATVFKGKSKLTDNIVALKEIRLEH-EEGAPCTAIREVSLLKGLKHANIVTLHDTVHTQKSLTLVFEYLEKDLKQYMDDCGG-IMSMNNVRIFLFQLLRGLDYCHKRKVLHRDLKPQNLLIND-KGELKLADFGLARAKSVPTKTYSNEVVTLWYRPPDVLLGSTAYSTQIDMWGVGCIFFEMATGRPLFPGSTVEDELLLIFKVLGTPSEEVWPGISANEAFIAGKFPDYPRENLIIHAPRLDNSGLELLEKFLEYTVKDRVSAHDAMRHDYF

>Hsa-cdk16

YIKLDKLGEGTYATVYKGKSKLTDNLVALKEIRLEH-EEGAPCTAIREVSLLKDLKHANIVTLHDIIHTEKSLTLVFEYLDKDLKQYLDDCGN-IINMHNVKLFLFQLLRGLAYCHRQKVLHRDLKPQNLLINE-RGELKLADFGLARAKSIPTKTYSNEVVTLWYRPPDILLGSTDYSTQIDMWGVGCIFYEMATGRPLFPGSTVEEQLHFIFRILGTPTEETWPGILSNEEFKTYNYPKYRAEALLSHAPRLDSDGADLLTKLLQFEGRNRISAEDAMKHPFF

>Hsa-cdk18

YVKLDKLGEGTYATVFKGRSKLTENLVALKEIRLEH-EEGAPCTAIREVSLLKNLKHANIVTLHDLIHTDRSLTLVFEYLDSDLKQYLDHCGN-LMSMHNVKIFMFQLLRGLAYCHHRKILHRDLKPQNLLINE-RGELKLADFGLARAKSVPTKTYSNEVVTLWYRPPDVLLGSTEYSTPIDMWGVGCIHYEMATGRPLFPGSTVKEELHLIFRLLGTPTEETWPGVTAFSEFRTYSFPCYLPQPLINHAPRLDTDGIHLLSSLLLYESKSRMSAEAALSHSYF

>Tad-gi|195996637|

YDKLHKLGEGTYATVFKGRSKLTNDFVALKEIRLEH-EEGAPCTAIREVSLLKDLKHANIVTLHDTIHTERSLVLVFEYLDRDLKQYMDSCGS-ILDMSNVKIFLFQLLRGLAYCHSRRVLHRDLKPQNLLINE-RGELKLADFGLARAKSVPSKTYSSEVVTLWYRPPDVLLGSTEYSTSIDMWGVGCIFYEMATGLPMFPGSSTENQLQTIWEILGTPTEEEWSGLTRNLKVNSLSFHDCKGEPLRNRAPRLEADGLDLLAKFLQYKAKSRISSADAMKHKYF

>Aqu-gi|340369066|

YEKLEKLGEGTYATVYKGKSNITGKLVALKEIRLEH-EEGAPCTAIREVSLLKDLKHANIVFLHDIIHTARSLTLIFEYVEQDLKQYLDQCSG-MMAMPNVKLFLFQLMRGLQYCHSRKILHRDLKPQNLLISE-QGDLKLADFGLARAKSVPTKTYSNEVVTLWYRPPDVLLGSIDYADSIDMWGVGCIFYEMIVGRPMFPGANVEEELVLIWKSLGTPNEKTWPGITKNKEFISHSFLRYDPQPLGLIVPRLDKEGINLMSKLLSYESQERLLARDGMKHNYF

>Mbr-gi|167522415|

YKKLHKLGEGTYATVFKGISHINGKIVALKEIRLEH-EEGAPCTGIREVSLLKGLKHANIVTLHDVIHTKDNLIMVFEFLSKDLKAYMDDCNS-YIDLRNAKLFLFQLLRGVGFCHSRKVLHRDLKPQNLLINH-AGELKLADFGLARAKSVPIKTYSNEVVTLWYRPPDVLLGSVDYSGDIDMWGVGCIFGEMISGRPMFPGATNADQLELIFKTLGSPSESTWPGVMALPEAKSNELGAYSPQPVNAILPRLDKQGGALLKSLLKLDPHGRVSAVKAMQHPYF

>Hsa-cdk14

YEKLEKLGEGSYATVYKGKSKVNGKLVALKVIRLQE-EEGTPFTAIREASLLKGLKHANIVLLHDIIHTKETLTLVFEYVHTDLCQYMDKHPG-GLHPDNVKLFLFQLLRGLSYIHQRYILHRDLKPQNLLISD-TGELKLADFGLARAKSVPSHTYSNEVVTLWYRPPDVLLGSTEYSTCLDMWGVGCIFVEMIQGVAAFPGMKIQDQLERIFLVLGTPNEDTWPGVHSLPHFKPERFTLYSSKNLRQAWNKLSNHAEDLASKLLQCSPKNRLSAQAALSHEYF

>Hsa-cdk15

YLNLEKLGEGSYATVYKGISRINGQLVALKVISMNA-EEGVPFTAIREASLLKGLKHANIVLLHDIIHTKETLTFVFEYMHTDLAQYMSQHPG-GLHPHNVRLFMFQLLRGLAYIHHQHVLHRDLKPQNLLISH-LGELKLADFGLARAKSIPSQTYSSEVVTLWYRPPDALLGATEYSSELDIWGAGCIFIEMFQGQPLFPGVSILEQLEKIWEVLGVPTEDTWPGVSKLPNYNPEWFPLPTPRSLHVVWNRLGPEAEDLASQMLKGFPRDRVSAQEALVHDYF

>Tad-gi|196001193|

YEKLEPLGEGSYATVYKGYSIGHQKLVALKEITLNE-EEGTPFTAIREASLLKQLKHANIVVLHDIIQTPTKLTFVFEYVTTDLSQYLNLHPG-GLNMKNVKLFLYQLLRGLSYCHQRRILHRDIKPQNILVSE-IGEIKLADFGLARAKSVPSKTYSPDVVTLWYRPPDVLLGSTNYSTSLDIWGVGCIFTEMVSGIATFPGVRISDQLDKIWHKLGTPTEETWPGVTSYPEYCAAETMFYESRTIAEVIPRLMPGAADLATKMLQYQPFKRIFCQAAMNHQYF

>Nve-gi|156392090|

YQKLEPLGEGSYATVFKGLCTANKKIVALKQIRLQE-EEGAPFTAIREASLLKQLKHGNIVKLHDIIHTKDTLMFVFEFLDTDLNCYLEKYSR-GICPHNTQLFCFQLLRGLAYIHDRKILHRDIKPQNLLISE-RGELKLADFGLARAKSVPSQTYSHEVVTLWYRPPDVLLGSKNYTTSLDIWGAGCIFVEMLTGIALFPGLNHIDQLNKIWQVLGTPTDETWPGVSKLPEYDPDIFINFRPRRIGQCIPRLVEGAEQLVIRMIQLDPKNRISAREAMRSTYF

**10.Multiple alignments for phylogenetic analysis of Cyclin B like group proteins in *H. sapiens*, *N. vectensis*, *T. adhaerens*, *A.queenslandica*, *M. brevicollis*, and *S. rosetta*.**

>Hsa-CyclinA1

EIYQYLREAEIRHRPKAHYMKKQPDITEGMRTILVDWLVEVGEEYKLRAETLYLAVNFLDRFLSCMSVLRGKLQLVGTAAMLLASKYEEIYPPEVDEFVYITDDTYTKRQLLKMEHLLLKVLAFDLTVPTTNQFLLQYLRRQGVCVRTENLAKYVAELSLLEDPFLKYLPSLIAAAAFCLANYTVNKHFWPETLAAFTGYSLSEIVPCLSELHKAYLDIPHRPQQAIREKY

>Nve-gi|156375154|

DIFKYLKQAELNNRAKPGYMRKQPDINNSMRAILVDWLVEVAEEYKLLPQTLYLTVNYIDRFLSAMSVLRGKLQLVGTACMLLASKFEEIYPPEVSEFVYITDDTYTAKQVLKMEQLVLKVLTFDLSVPTILNFLERFIKATNVPPKVEALARYLCEISLLDEPFLKYLPSTIAASAIVLSLHTLGLSYWNNTLSHYTGFELHDLQTCIQDLHRSFAYAPNHPQQATREKY

>Hsa-CyclinA2

DIHTYLREMEVKCKPKVGYMKKQPDITNSMRAILVDWLVEVGEEYKLQNETLHLAVNYIDRFLSSMSVLRGKLQLVGTAAMLLASKFEEIYPPEVAEFVYITDDTYTKKQVLRMEHLVLKVLTFDLAAPTVNQFLTQYFHQQPANCKVESLAMFLGELSLIDDPYLKYLPSVIAGAAFHLALYTVTGQSWPESLIRKTGYTLESLKPCLMDLHQTYLKAPQHAQQSIREKY

>Tad-gi|196005765|

DIHSYLRKAEYFHRPKYDYMERQCDVNGTMRSILVDWLVEVSEEYKLRERTLYLAISYIDRFLSAMSVRRSKLQLVGTAALFIAAKFQEIYPPDCAEFAYITDDTYNIKQVLKMESLMLKVLSFNLSSPTAVDFLERYGSEAGLDSEIRELSMYLTELTLKDYGFLQFMPSLIAVSAVSLALHTFKLKYWPQELSTYTNYQWQQVSPCLNRIFEAFRLAHTQPQRAVVEKY

>Mbr-gi|167517989|

-------------------MRKQRDINHTMRSILIDWLIEVTEEYKLTLQTFFVTVGYVDRFLSEMAVQRSKLQLVGVTCMLLACKYEEIYPPTIDDFVYITDKTYSRPQVMKMEHVILKVLRFDMGSCTPLTFLYYFLNAIPHHDDTKWLAQYLCELSAYDRRSLGQRPSTTAAAAIVIALHTFELHPLPPALVSVIRQGPEELQAAVNTLHEIFSVYPNLQHEAIKEKY

>Sro-gi|326426811|

KVMTYLRHLEKKFRPHAGYMGRQRDINHNMRSILVDWLVEVTEEYRLQLQTLYIAVGYIDRFLSNMAVQRSKLQLVGVTCMLLAAKYEEIYPPSVNEFVYITDNTYRREQVLKMEHVVLKVLRFDMGACTALTFLVRFIHAASATPPSHCLALYLAELSLLLNKFIQYLPSVKAAAAICLSQHTFARPVWTPTFERYCRLSPEEVQPCLNDMFEAMTSAPHLEYQAIREKY

>Hsa-CyclinB2

DIYQYLRQLEVLQSINPHFLD-GRDINGRMRAILVDWLVQVHSKFRLLQETLYMCVGIMDRFLQVQPVSRKKLQLVGITALLLASKYEEMFSPNIEDFVYITDNAYTSSQIREMETLILKELKFELGRPLPLHFLRRASKAGEVDVEQHTLAKYLMELTLIDYDMVHYHPSKVAAAASCLSQKVLGQGKWNLKQQYYTGYTENEVLEVMQHMAKNVVKVNLTKFIAIKNKY

>Hsa-CyclinB1

DIYAYLRQLEEEQAVRPKYLL-GREVTGNMRAILIDWLVQVQMKFRLLQETMYMTVSIIDRFMQNNCVPKKMLQLVGVTAMFIASKYEEMYPPEIGDFAFVTDNTYTKHQIRQMEMKILRALNFGLGRPLPLHFLRRASKIGEVDVEQHTLAKYLMELTMLDYDMVHFPPSQIAAGAFCLALKILDNGEWTPTLQHYLSYTEESLLPVMQHLAKNVVMQGLTKHMTVKNKY

>Nve-gi|156364707|

EIMRFLRAMEEHYSVSPTYMNNQQEVNEKMRAILLDWLVQVHLKFRLLQETLYITMSIIDRFLAVHQVSKRELQLVGVGAMLLASKYEEMFAPEIGDFVYITDHAYTKKQIRQMESLIFRKLDFSLGKPLCLHFLRRNSKAGAVGAEEHTMAKYLMELTLIDYQSIKFLPSEIAAASLSLAMRVMGKSEWTPTLEHYSGYSEKKLSTCMQRLAQLVLGARDSKQKAVYNKY

>Tad-gi|196002535|

DIYKNMLKQEKRCTLDPDYMTGQPVITKGMRAILLDWLVDVHLRYNFHPESLYLTTYIIDRYLQTTQVNRKKLQLVGIAAFYIAIKYEEIFLASTDDLLYLTENSYEINEFIQMEAKILKALDFSLSRPTSIHFLRRISKAASADIEQHTFARYLTEIALIEYSLLSYLPSQIAAAASLISLKIFD-KSWTPTLQYYSSYSEDSLKPVARQIAKLAWKSW-------TSKY

>Mbr-gi|167523717|

DIYNYMREMEVRLCCDPAYLQSQPEVNERMRAILIDWLVEVHYRFELLQETLYLTVDVLDRFLSSERTSRSQLQLVGVTAMLIASKYEEMYPPEVGDFVYISDNAYRREQILAMEQTMLRVLDFNLGKPLPLHFLRRDSRAGHADGTMHTFAKYFMELTLCSPRFLGYKPSQVAAAATYISREVVGEQLWTPTIEFFADYTLTDIMPVILDMKAILRESPTAKQQAVRTKF

>Sro-gi|326428978|

PIFEYMRELEVRLHVPANYFKIQTEINARMRDVLVDWLAEVHHRFELIQETFHLTVHLLDRYLSKEPVTRDDVQLVGITAMMVAAKYEEMYPPELGDYVYITDKAYSEDRILAMERKLLRVLDFSLGKPLPLHFLRRNSKAGHADATMHSMGKYMIELSLGSHAMLKYVPSQLAAAATYISREIVGEELWNPTLEHYAKYSLEDIAPVVHDMRAVLKHSTVSRLQAIRNKF

>Hsa-CyclinB3

EIFSYMKEREEQFIL-TDYMNRQIEITSDMRAILVDWLVEVQVSFEMTHETLYLAVKLVDLYLMKAVCKKDKLQLLGATAFMIAAKFEEHNSPRVDDFVYICDDNYQRSEVLSMEINILNVLKCDINIPIAYHFLRRYARCIHTNMKTLTLSRYICEMTLQEYHYVQEKASKLAAASLLLALYMKKLGYWVPFLEHYSGYSISELHPLVRQLNKLLTFSSYDSLKAVYYKY

>Nve-gi|156380901|

-------------------MEKQKELTISMRAILVDWLVEVQESFELYHETLYLGVRVLDNYLMRSYVERENLQLVGAVSLYIACKVEERHPPCLDDFLYICDDAYQQKAFVAMEKKILNSLEFNINMPIPYRFLRRFAKVASADVKTLTLSRFILETTLHHYKFIVHKPSFLAAACLRLALRMKGCDDWTPTVVHYTGYSVAQLDGCVIELNEMISEPPKQNLMTVRNKY

>Tad-gi|196003740|

IIFENMKQREAQLVV-NDYLERQNDITEQMRMILIDWLCEVQQNFELFHETLYLAVKIVDRFLSARVVSRDALQLIGATAMLMSSKIEERYPPLVDDFVYICDDAYSRQAVLDMERDICYALDFDLNIPIPYRFLRRYGKVASLSMENLTLARYILELTLQEYQFVTFKPSMLAAGCLCLALKMKNCGEWTQTLVHYSGYEESELNELVQKLNAMIAKPAPENCKVVKTKY

>Aqu-gi|340374274|

DIYQYMREREVKFKV-SSYLDHQPLINSSMRSILIDWLVEVQENFELFHETLYLAVKIVDRYLEKKEVKKEYLQLVGATSMLIAAKFEELSPPLVDDFIYLCDDAYQHDELLSMERNILATLEYDVNAPVAYRFLRRLARAAGADMETHTLARYICESTLQEYEFVSDDPSHIAGAAMYLSIRMKGLGGWTPTLQHYSQYEASNLLPMVQRLNDLISR-PAGNTSTVRSKY

>Nve-gi|156359300|

QIMRYAMALENKYQLPENFLEKQEEVSHQARAVLIDWLIEVHLFYNFPQDCLYLIVALVDRYMSLRTVPVAHFQLLGMACLLVACKYEDRFVPTREELVAMADQAFDQSELMHMETRLLTCLEFDLAQPLPTFFLRPIARASAIDLETYVVSKFIMEAAMLDAIMVTFKPSIIAATAFFMAR-------------------------------------------------

>Hsa-CyclinO

SCYAFRKAQESHFHP-REALARQPQVTAESRCKLLSWLIPVHRQFGLSFESLCLTVNTLDRFLTTTPVAADCFQLLGVTSLLIACKQVEVHPPRVKQLLALCCGAFSRQQLCNLECIVLHKLHFTLGAPTISFFLEHFTHARVEALEAQALARGVAELSLADYAFTSYSPSLLAICCLALADRMLRVS--RPVDLRLGDHPEAALEDCMGKLQLLVA--------------

>Tad-gi|196012973|

-------------------------ITPTQREALISSMINLSRKHNIKRDTLFMAVNYLDRFLKVVTVTEDCFELVGLTCMMIACKVEECQPPKMEEFLTSCTHYYKKAEMKRLEIIILNYIDFRLSPPIAPHFLEYIIHFHQHYIELVNIANQVLLKILPTYRFNHIKSSILAASAFEYA--------------------------------------------------

>Hsa-CyclinE1

EVWKIMLNKEKTYLRDQHFLEQHPLLQPKMRAILLDWLMEVCEVYKLHRETFYLAQDFFDRYMATQNVVKTLLQLIGISSLFIAAKLEEIYPPKLHQFAYVTDGACSGDEILTMELMIMKALKWRLSPLTIVSWLNVYMQVAYLNDHEFIQIAELLDLCVLDVDCLEFPYGILAASALYHFS-------SSELMQKVSGYQWCDIENCVKWMVPFAMVDEHNIQTHRDSML

>Hsa-CyclinE2

EVWLNMLKKESRYVHDKHFEVLHSDLEPQMRSILLDWLLEVCEVYTLHRETFYLAQDFFDRFMLTQDINKNMLQLIGITSLFIASKLEEIYAPKLQEFAYVTDGACSEEDILRMELIILKALKWELCPVTIISWLNLFLQVDALKDAKFIQIAQLLDLCILAIDSLEFQYRILTAAALCHFT-------SIEVVKKASGLEWDSISECVDWMVPFVNV-----------IN

>Nve-gi|156363375|

ELWSYMLEKDRKYTKDHLYLRQHPHLQPRMRAILLDWLIEVCEVYRLHRETYFLAVDFVDRYLSVKDIPKQRLQLVGTTALFIAAKLEEIYPPKLSEFAYVTDGACKEDEILQQELLMLQDLNWKLCPITSNTWLNIYMQLHWLSRNNFIKVSQLLDICSLDIESLQFSYSVLAAAAMYHVI--------PVSIEEITCHKREDLSPCIQWMGPFAAT-----------RD

>Tad-gi|196003236|

EVWQVMIENDNNFKRSPDCFDRHPNLVPNMRGILLDWMMEVCESFKMQRETFYMAMDYLDRYLSLSNILKQKLQLIGTTCLFIAAKIEEIQPPQVSEFAYVTDSACSEDDIIKLELQLLQTLEFQLSPVTVTSWLNVYVQLFNIKLSDYMKAIRLIDLCILDSWCLMHSYRSIAASAFYLIAP------SKQLAIDCTGYLWENLTSCISWMMPYETV-----------KY

>Aqu-gi|340379787|

NLWREMRLKDTSQAPGTELRLRHPSIMPTMRTILLDWMLEVCEEYRIHRETYYLSLELFDRFMDTQNVQKEQLQLIGVTCLFIASKIEEIYPPKLADFAYVTDGACNSEEIVFMELMICKALKWRLHHCSVNTWVNLYMQLVSSY--EFIRVMQVLDLCTLDITSRQFCNSILAASALYLVSEK-----CQMHLNLVTGFQLADIHVCVQWLNAFVSV--NRMAQPVQKQQ

>Hsa-CyclinD2

RVLQNLLTIEERYLPQCSYFCVQKDIQPYMRRMVATWMLEVCEEQKCEEEVFPLAMNYLDRFLAGVPTPKSHLQLLGAVCMFLASKLKETSPLTAEKLCIYTDNSIKPQELLEWELVVLGKLKWNLAAVTPHDFIEHILRKLPQQREKRKHAQTFIALCATDFKFAMYPPSMIATGSVGAAICGLQQDALTELLAKITNTDVDCLKACQEQIEAVLLNS-QQYRQDQRDG-

>Hsa-CyclinD1

RVLRAMLKAEETCAPSVSYFCVQKEVLPSMRKIVATWMLEVCEEQKCEEEVFPLAMNYLDRFLSLEPVKKSRLQLLGATCMFVASKMKETIPLTAEKLCIYTDNSIRPEELLQMELLLVNKLKWNLAAMTPHDFIEHFLSKMPEAEENRKHAQTFVALCATDVKFISNPPSMVAAGSVVAAVQGLNLYRLTRFLSRVIKCDPDCLRACQEQIEALLQAQ-NMDPKAAEEE-

>Hsa-CyclinD3

RVLQSLLRLEERYVPRASYFCVQREIKPHMRKMLAYWMLEVCEEQRCEEEVFPLAMNYLDRYLSCVPTRKAQLQLLGAVCMLLASKLRETTPLTIEKLCIYTDHAVSPRQLRDWEVLVLGKLKWDLAAVIAHDFLAFILHRLSLPRDRKKHAQTFLALCATDYTFAMYPPSMIATGSIGAAVQGLGADELTELLAGITGTEVDCLRACQEQIEAALASQ-SSSPAPKAPR-

>Nve-gi|156350442|

RVLTNLLACEERYLPSCNYFIVQTEVEPHMRKLVATWMLEVCEEERCEEEVFALSMNYLDRILSLLPVKKFQLQLLGAVCMFIASKMKETSPLTAEKLCIYTDNSITTEELLDWELLVLGKLKWDVSAVTPHDFLDQIFSRLPLDRSTRKHASTFIALCCTDDKFLLYTPSMLAAASVCAAFTGLGIRSWTALLHAITNIEPECLRSCQDLMEE----V-HLSVKADPTR-

>Tad-gi|196001479|

RVLQNLLKLEENYLPNRDYFNVQREIKPFMRCMVTNWMLDVCHGENCNDKVFPLAVNYLDRFLSMVSIRKIRLQLVGSVCLFLASKLKDRIPLTAEKLCSWTDYSVTCQELLDWELLLLEKLKWDLGSVTPLDFLDQILYRLSFELKYKKHAYTLIALCCTDFQLCTNPPSMIAGGCILCALAGV--AIVNRIVQKITSIEPDYLICCKEQVED--LSK-NSSHKIDQID-

>Hsa-CyclinG2

LLN-VYLEQEEQI---END----NTLCPGLRNAKVEDLRSLANFFGSCTETFVLAVNILDRFLALMKVKPKHLSCIGVCSFLLAARIVEENIPSTHDVIRISQCKCTASDIKRMEKIISEKLHYELEATTALNFLHLYHTIILCHT-LDKLEA-QLKACNCRLIFSKAKPSVLALCLLNLEVE--TLSELLEILLLVSKINDTEFFYWRELVS-----------------S

>Hsa-CyclinG1

QLN-ALLEQESQI---ENG----LRMTARLRDFEVKDLLSLTQFFGFDTETFSLAVNLLDRFLSKMKVQPKHLGCVGLSCFYLAVKSIEENVPLATDLIRISQYRFTVSDLMRMEKIVLEKVCWKVKATTAFQFLQLYYSLLQENL-FERLEA-QLKACHCRIIFSKAKPSVLALSIIALEIQ--AQCELTEGIECLSKINGRDLTFWQELVS-----------------R

>Hsa-CyclinI

LLE-KAITREAQM---PSN----QNVSPSQRDEVIQWLAKLKYQFNLYPETFALASSLLDRFLATVKAHPKYLSCIAISCFFLAAKTVEERIPVLKVLARDSFCGCSSSEILRMERIILDKLNWDLHTATPLDFLHIFHAIAVSTRPLAVLTK-QLLHCMACNQLLQFRGSMLALAMVSLEME--KLPDWLSLTIELAQMDSSQLIHCRELVA-----------------D

>Nve-gi|156408249|

------------------------------RDKSVCILLHINRHCGFQPETFALAVNLLDRFLSVVKANPKYLPCISISCMFLAAKMVEEAIPTAGNLIGVSGLSCTPSDLLRMERIILDKLGWNLSAVTPLQLLQVFHALCVSKG-LHHITL-KLEELLCNHKFTFFKPSTLALSLLSCEIS--SLNVWIEATIMLAQ--------------------------------

>Hsa-CyclinI2

HLQ-LAQDREARG---KPQ----DEICDAFE-EVVLWLLRLQNTFYFSQSTFNLALTIFGRLLISVKVKEKYLHCATITSLRLAAKVNEEFIPQVKDFTKHYGSDYSPNELLRMELAILDRLHWDLYIGTPLDFLTIFHALVVLSWPVASLTR-QLQHCMAGHQLLQFKGSTLALVIITLELE--RLPGWCAPISDLAQVGDMQYSCCKELVM------------------

>Hsa-CyclinJ

DIHQALRYKELKL---PSYKG--QSPQLSLRRYFADLIAIVSNRFTLCPSARHLAVYLLDLFMDRYDISIQQLHLVALSCLLLASKFEEKSVPKLEQLNSLTNLVLTKQNLLHMELLLLETFQWNLCLPTAAHFIEYYLSEAVHETDMAKYADYFLEVSLQDYAFLNYAPSLVAAACVASSRIILRLPTWPTRLHRLTAYSWDFLVQCIERLLIA---------------S

>Hsa-CyclinJL

DVHCTLREKELKL---PTFRA--HSPLLKSRRFFVDILTLLSSHCQLCPAARHLAVYLLDHFMDRYNVTSKQLYTVAVSCLLLANGFEDRHVPKLEQINSTSNFTLTKKELLSTELLLLEAFSWNLCLPTPAHFLDYYLLASVSQKDLKEYAHYFLEVTLQDHIFYKFQPSVVAAACVGASRICLQLPYWTRDLQRISSYSLEHLSTCIEILLVV---------------T

>Nve-gi|156407073|

DIHEVLREKEARI---PNFMA--ASPQLKIRRYLVDWLAVIGEKLGSSHGVVHLAIYYMDFFMDKFIIQESQLHLLALTALLLAAKFDENQIPDISTLNKFVNNTYQHAEYHQMELLLLEFFNWNIDLPTPVQFLEYYLAKATIDYKLRKYVYYFLEISFQDHTFLSFSPSLITSSCIAASRICLNLPSWTNELSKVTNYDWDKIAHCTEIM-------------------

>Hsa-CyclinF

QL----FQASQAV-SKQQVFSVQKGLNDTMRYILIDWLVEVATMKDFTSLCLHLTVECVDRYLRRRLVPRYRLQLLGIACMVICTRFISKEILTIREAVWLTDNTYKYEDLVRMMGEIVSALEGKIRVPTVVDYKEVLLTLVPVELRTQHLCSFLCELSLLHTSLSAYAPARLAAAALLLARLTHGQQPWTTQLWDLTGFSYEDLIPCVLSLHKKCFDYRQVSLTAVKQRF

>Nve-gi|156365799|

QF----LQKSKAL-NSHKLFGFQTELNNTMRYILVDWLVEVALMKDFSSQIVHIAVHCVDQYLMKRKVQRSELQLLGITCILIAARFQGKDIVTIREASWLTDDTYSYEEVVRMMGEVMSCLRGEVR--------------------------------------------------------------------------------------------------------

>Aqu-gi|340380414|

QI----VRSSPSL-LSASIYDAQPDVNEEMVFILLDWIVEVAEMKSFSTKTLHLAISLIQRYMVARKLKRSRLQLLGVTALLLAARWTAVPIITIREAAWLTDNTYRYDEVVCMMGEIVSTLHGEIQKPTVPDYLEMFELLVNADKKSSCLAAYVSESAVLFPDFGRYTAAQIAAGCLLLARVLLEQLPWPSALVEATGLTVPDLYHCTSLLYSKCLDYRGVKLCAIKTRY

>Tad-gi|195999522|

KM----VESCRNI-NIKLVNRIQLELNDRMRYILIDWLVEVAEMKEFSSEMLCNAIDLVDRYLEINPIPRSNLQLLGISCMVIASRYHCVDIMTIREAAWLTDNTYKYDEVVRMIGEVFAAVNGEIRTPSAFDYLKIFCTISEVSQKCTYLASFILELSWLFLENSRYKSAVKAAASLLLARVLMGNLPWTEELKSYTGLSLEDLSSCVLHLYKKCLDYYNSEVKSVHNRY

>Aqu-gi|340376468|

QLESSLREKSEDLARAKQLHAELANLMDEMRALRVNYDQKMKEYEELFDATSSIDVDPDGRFVQVTEIPKNKLQLVGVTAMLIASKYEEMYAPEVADFVYITDSTYSNTEIKAMERNILKTLDYSFGNPLCLHFLRRNSRAGDATPQMHTMAKFLMELCLPDYSMLEYLPSLVAAAALYISNKLYSDGEWTPALRHYSQYTEPDVLPCVGKMASLVLSMHTAKQQAVKNKY

>Aqu-gi|340369567|

PIHETLKKREAAI---SSIQF--KSPQLHLRRELVEFITAVSKDLGLSDGTRFLAIRLADQFMDGHNVMEYRLRLMGLTCLLLAAKSEEINVPSIEMLQHANTSAYSRQEFHTLELYILKYFKWCLSHPSVAHFIDYYLHTSLKGDEMKEFTAYFMEVTLRGIKANSLSRNCKDAAGI-----LPAACKWLVYFKTYI---------------------------------

>Mbr-gi|167522783|

E-----AFARGAA-PVRRGGLRQPDREGKMRFILMDWLIEVADLKTFGGETLFVAMDLVDRFLQHCRITRKTLQLLGIACMVMAARYLEEGVITIREAAWLTDSMYSYDQIVRTIGQVLVDVSGNVIRPTTFHYLNLLLQIGGATPAVFLLGQHMAEALILTIPLTEFPPAKLAAAMACCTFALAGVQPWSSTMERWSGLELRTIYDLAIKCFSLF-DHRGTELRAVKDRY

>Sro-gi|326437989|

L-----GMEKLRL-RSDAPPPAQPDSQGKMRFILVDWLLEVASLKMYSIDTLHCAVDMVDRYLATRTITRRTLQLLGITCMVIAARFLEQDVVTIREAAWLTENTYDYEDVVQMVAAVLAVADGHVRRPSPNDYITIFAELSNVPVHIRCFMDYVSESCLLHQPTLTHAPAALGAAIYFVSMHLVGCSAWPSSLTSNSDLRVSHFQEEIMEVFRCL-DHRGLELRAVEDRY

>Aqu-gi|340370065|

QLR-AVLETYTQP---RSY----KYVTARDRDEQVSWFRNITHDLSFSISVFYQSTLLLDTFLSTKKTKREFLQTMAASCYSIATKLVESSVALSHKLVSKYYSGCSVTDVEKMEAFLIQTPDIASALHTIQDYIKKFHRLAERSE-LCHLYK-CAQHVVCNHELMKYRPSMLGLAILGCHLK--QLCDWLSTLLYLLQIRGGELSICYEAVA-----------------R

>Sro-gi|326435281|

AIYAKNLQREEKYLLNP---RPSRGINLAMRTVVIDWMIEIQVSFKLRDETLFCAVDILDRYLAARPEQRHDYQCCGATSLWIASKFLEVLPPELADFEYVCAGLYPRQAFIDKELTMLTALRFYVMNVTPLDFISVYAIVLQLSLEGMALAEYLITLPLQEQRFYGLRPSVRGAAAVHIASKTCDGPGWSEDHSALFKLDHRHIMLVAQQLRGLANE--PEKYINCRSRF

>Sro-gi|326437558|

QYYRVLEQRLASCEVSINALKMQPCMPPRLRARVFHWLADVCDRARMTLDTLFFAITYFDTYCSVRAVTVANMQLLACACLRLAAKIEETRVPSLRLLSRLTDGACQPTGIAQFELDLAAVLKWRLIRSTPLHWTRFFIGVALGDPSTLFRSCQVLQLAMSDAWALRFDARQMAAAAVLLSAT------RPIDILAVTGLDKCALRTCLRWMHCFAGGFSDASEEGVGVSS

>Mbr-gi|167526102|

EVHRNMRVQESQL---QLAQY--CGPLADLRPRCVQRIHRLARAFRFHRLTRDAAIFYFDRMLFLFHMHESHLELAVQTAFLMAAKCQEAVVPTHHDLHRAGCAVVPTAHLKAFEASYLERVDWILTSVTPSDFLDYYARFSISSQDTLDLADQVLQEASMANHVATFLPSHRAAAAITTARLIVDIPAWSPTLQAVSGLTWREISPCVDAMLQLNLNP-HREMAALRQCT
