# Supplementary material for: Phylogenetic analysis of CDK and cyclin proteins in premetazoan lineages
Source: BMC Evol Biol. 2014 Jan 17;14:10. doi: 10.1186/1471-2148-14-10 (PMC3923393; doi:10.1186/1471-2148-14-10)
Supplement: Additional file 2: File S2 — CDK sequences from 18 organisms. [file 1471-2148-14-10-S2.doc]

**File S2. CDK sequences from 18 organisms.**

>Hsa-CDK7 gi|4502743|ref|NP_001790.1| cyclin-dependent kinase 7 [Homo sapiens]

MALDVKSRAKRYEKLDFLGEGQFATVYKARDKNTNQIVAIKKIKLGHRSEAKDGINRTALREIKLLQELSHPNIIGLLDA

FGHKSNISLVFDFMETDLEVIIKDNSLVLTPSHIKAYMLMTLQGLEYLHQHWILHRDLKPNNLLLDENGVLKLADFGLAK

SFGSPNRAYTHQVVTRWYRAPELLFGARMYGVGVDMWAVGCILAELLLRVPFLPGDSDLDQLTRIFETLGTPTEEQWPDM

CSLPDYVTFKSFPGIPLHHIFSAAGDDLLDLIQGLFLFNPCARITATQALKMKYFSNRPGPTPGCQLPRPNCPVETLKEQ

SNPALAIKRKRTEALEQGGLPKKLIF

>Hsa-CDK3 gi|4557439|ref|NP_001249.1| cell division protein kinase 3 [Homo sapiens]

MDMFQKVEKIGEGTYGVVYKAKNRETGQLVALKKIRLDLEMEGVPSTAIREISLLKELKHPNIVRLLDVVHNERKLYLVF

EFLSQDLKKYMDSTPGSELPLHLIKSYLFQLLQGVSFCHSHRVIHRDLKPQNLLINELGAIKLADFGLARAFGVPLRTYT

HEVVTLWYRAPEILLGSKFYTTAVDIWSIGCIFAEMVTRKALFPGDSEIDQLFRIFRMLGTPSEDTWPGVTQLPDYKGSF

PKWTRKGLEEIVPNLEPEGRDLLMQLLQYDPSQRITAKTALAHPYFSSPEPSPAARQYVLQRFRH

>Hsa-CDK2 gi|16936528|ref|NP_001789.2| cell division protein kinase 2 isoform 1 [Homo sapiens]

MENFQKVEKIGEGTYGVVYKARNKLTGEVVALKKIRLDTETEGVPSTAIREISLLKELNHPNIVKLLDVIHTENKLYLVF

EFLHQDLKKFMDASALTGIPLPLIKSYLFQLLQGLAFCHSHRVLHRDLKPQNLLINTEGAIKLADFGLARAFGVPVRTYT

HEVVTLWYRAPEILLGCKYYSTAVDIWSLGCIFAEMVTRRALFPGDSEIDQLFRIFRTLGTPDEVVWPGVTSMPDYKPSF

PKWARQDFSKVVPPLDEDGRSLLSQMLHYDPNKRISAKAALAHPFFQDVTKPVPHLRL

>Hsa-CDK5 gi|4826675|ref|NP_004926.1| cell division protein kinase 5 isoform 1 [Homo sapiens]

MQKYEKLEKIGEGTYGTVFKAKNRETHEIVALKRVRLDDDDEGVPSSALREICLLKELKHKNIVRLHDVLHSDKKLTLVF

EFCDQDLKKYFDSCNGDLDPEIVKSFLFQLLKGLGFCHSRNVLHRDLKPQNLLINRNGELKLADFGLARAFGIPVRCYSA

EVVTLWYRPPDVLFGAKLYSTSIDMWSAGCIFAELANAGRPLFPGNDVDDQLKRIFRLLGTPTEEQWPSMTKLPDYKPYP

MYPATTSLVNVVPKLNATGRDLLQNLLKCNPVQRISAEEALQHPYFSDFCPP

>Hsa-CDk1 gi|4502709|ref|NP_001777.1| cell division cycle 2 isoform 1 [Homo sapiens]

MEDYTKIEKIGEGTYGVVYKGRHKTTGQVVAMKKIRLESEEEGVPSTAIREISLLKELRHPNIVSLQDVLMQDSRLYLIF

EFLSMDLKKYLDSIPPGQYMDSSLVKSYLYQILQGIVFCHSRRVLHRDLKPQNLLIDDKGTIKLADFGLARAFGIPIRVY

THEVVTLWYRSPEVLLGSARYSTPVDIWSIGTIFAELATKKPLFHGDSEIDQLFRIFRALGTPNNEVWPEVESLQDYKNT

FPKWKPGSLASHVKNLDENGLDLLSKMLIYDPAKRISGKMALNHPYFNDLDNQIKKM

>Hsa-cdk11a gi|148763347|ref|NP_076916.2| cell division protein kinase 11A isoform 1 [Homo sapiens]

MGDEKDSWKVKTLDEILQEKKRRKEQEEKAEIKRLKNSDDRDSKRDSLEEGELRDHCMEITIRNSPYRREDSMEDRGEED

DSLAIKPPQQMSRKEKVHHRKDEKRKEKCRHHSHSAEGGKHARVKEREHERRKRHREEQDKARREWERQKRREMAREHSR

RERDRLEQLERKRERERKMREQQKEQREQKERERRAEERRKEREARREVSAHHRTMREDYSDKVKASHWSRSPPRPPRER

FELGDGRKPVKEEKMEERDLLSDLQDISDSERKTSSAESSSAESGSGSEEEEEEEEEEEEEGSTSEESEEEEEEEEEEEE

ETGSNSEEASEQSAEEVSEEEMSEDEERENENHLLVVPESRFDRDSGESEEAEEEVGEGTPQSSALTEGDYVPDSPALLP

IELKQELPKYLPALQGCRSVEEFQCLNRIEEGTYGVVYRAKDKKTDEIVALKRLKMEKEKEGFPITSLREINTILKAQHP

NIVTVREIVVGSNMDKIYIVMNYVEHDLKSLMETMKQPFLPGEVKTLMIQLLRGVKHLHDNWILHRDLKTSNLLLSHAGI

LKVGDFGLAREYGSPLKAYTPVVVTQWYRAPELLLGAKEYSTAVDMWSVGCIFGELLTQKPLFPGNSEIDQINKVFKELG

TPSEKIWPGYSELPVVKKMTFSEHPYNNLRKRFGALLSDQGFDLMNKFLTYFPGRRISAEDGLKHEYFRETPLPIDPSMF

PTWPAKSEQQRVKRGTSPRPPEGGLGYSQLGDDDLKETGFHLTTTNQGASAAGPGFSLKF

>Hsa-cdk20 gi|89903018|ref|NP_001034892.1| cell division protein kinase 20 isoform 3 [Homo sapiens]

MDQYCILGRIGEGAHGIVFKAKHVETGEIVALKKVALRRLEDGFPNQALREIKALQEMEDNQYVVQLKAVFPHGGGFVLA

FEFMLSDLAEVVRHAQRPLAQAQVKSYLQMLLKGVAFCHANNIVHRDLKPANLLISASGQLKIADFGLARVFSPDGSRLY

THQVATRWYRAPELLYGARQYDQGVDLWSVGCIMGELLNGSPLFPGKNDIEQLCYVLRILGTPNPQVWPELTELPDYNKI

SFKEQVPMPLEEVLPDVSPQALDLLGQFLLYPPHQRIAASKALLHQYFFTAPLPAHPSELPIPQRLGGPAPKAHPGPPHI

HDFHVDRPLEESLLNPELIRPFILEG

>Hsa-cdk11b gi|16332358|ref|NP_277021.1| cell division protein kinase 11B isoform 2 [Homo sapiens]

MGDEKDSWKVKTLDEILQEKKRRKEQEEKAEIKRLKNSDDRDSKRDSLEEGELRDHRMEITIRNSPYRREDSMEDRGEED

DSLAIKPPQQMSRKEKVHHRKDEKRKEKRRHRSHSAEGGKHARVKEKEREHERRKRHREEQDKARREWERQKRREMAREH

SRRERDRLEQLERKRERERKMREQQKEQREQKERERRAEERRKEREARREVSAHHRTMREDYSDKVKASHWSRSPPRPPR

ERFELGDGRKPVKEEKMEERDLLSDLQDISDSERKTSSAESSSAESGSGSEEEEEEEEEEEEEGSTSEESEEEEEEEEEE

EEETGSNSEEASEQSAEEVSEEEMSEDEERENENHLLVVPESRFDRDSGESEEAEEEVGEGTPQSSALTEGDYVPDSPAL

SPIELKQELPKYLPALQGCRSVEEFQCLNRIEEGTYGVVYRAKDKKTDEIVALKRLKMEKEKEGFPITSLREINTILKAQ

HPNIVTVREIVVGSNMDKIYIVMNYVEHDLKSLMETMKQPFLPGEVKTLMIQLLRGVKHLHDNWILHRDLKTSNLLLSHA

GILKVGDFGLAREYGSPLKAYTPVVVTLWYRAPELLLGAKEYSTAVDMWSVGCIFGELLTQKPLFPGKSEIDQINKVFKD

LGTPSEKIWPGYSELPAVKKMTFSEHPYNNLRKRFGALLSDQGFDLMNKFLTYFPGRRISAEDGLKHEYFRETPLPIDPS

MFPTWPAKSEQQRVKRGTSPRPPEGGLGYSQLGDDDLKETGFHLTTTNQGASAAGPGFSLKF

>Hsa-cdk10 gi|148596926|ref|NP_443714.3| cyclin-dependent kinase 10 isoform a [Homo sapiens]

MAEPDLECEQIRLKCIRKEGFFTVPPEHRLGRCRSVKEFEKLNRIGEGTYGIVYRARDTQTDEIVALKKVRMDKEKDGIP

ISSLREITLLLRLRHPNIVELKEVVVGNHLESIFLVMGYCEQDLASLLENMPTPFSEAQVKCIVLQVLRGLQYLHRNFII

HRDLKVSNLLMTDKGCVKTADFGLARAYGVPVKPMTPKVVTLWYRAPELLLGTTTQTTSIDMWAVGCILAELLAHRPLLP

GTSEIHQIDLIVQLLGTPSENIWPGFSKLPLVGQYSLRKQPYNNLKHKFPWLSEAGLRLLHFLFMYDPKKRATAGDCLES

SYFKEKPLPCEPELMPTFPHHRNKRAAPATSEGQSKRCKP

>Hsa-cdk16 gi|5453860|ref|NP_006192.1| cell division protein kinase 16 isoform 1 [Homo sapiens]

MDRMKKIKRQLSMTLRGGRGIDKTNGAPEQIGLDESGGGGGSDPGEAPTRAAPGELRSARGPLSSAPEIVHEDLKMGSDG

ESDQASATSSDEVQSPVRVRMRNHPPRKISTEDINKRLSLPADIRLPEGYLEKLTLNSPIFDKPLSRRLRRVSLSEIGFG

KLETYIKLDKLGEGTYATVYKGKSKLTDNLVALKEIRLEHEEGAPCTAIREVSLLKDLKHANIVTLHDIIHTEKSLTLVF

EYLDKDLKQYLDDCGNIINMHNVKLFLFQLLRGLAYCHRQKVLHRDLKPQNLLINERGELKLADFGLARAKSIPTKTYSN

EVVTLWYRPPDILLGSTDYSTQIDMWGVGCIFYEMATGRPLFPGSTVEEQLHFIFRILGTPTEETWPGILSNEEFKTYNY

PKYRAEALLSHAPRLDSDGADLLTKLLQFEGRNRISAEDAMKHPFFLSLGERIHKLPDTTSIFALKEIQLQKEASLRSSS

MPDSGRPAFRVVDTEF

>Hsa-cdk14 gi|6912584|ref|NP_036527.1| cell division protein kinase 14 [Homo sapiens]

MHGYFGCNAAAEPGYSAFVGTPQICVTKMSTRNCQGMDSVIKPLDTIPEDKKVRVQRTQSTFDPFEKPANQVKRVHSENN

ACINFKTSSTGKESPKVRRHSSPSSPTSPKFGKADSYEKLEKLGEGSYATVYKGKSKVNGKLVALKVIRLQEEEGTPFTA

IREASLLKGLKHANIVLLHDIIHTKETLTLVFEYVHTDLCQYMDKHPGGLHPDNVKLFLFQLLRGLSYIHQRYILHRDLK

PQNLLISDTGELKLADFGLARAKSVPSHTYSNEVVTLWYRPPDVLLGSTEYSTCLDMWGVGCIFVEMIQGVAAFPGMKDI

QDQLERIFLVLGTPNEDTWPGVHSLPHFKPERFTLYSSKNLRQAWNKLSYVNHAEDLASKLLQCSPKNRLSAQAALSHEY

FSDLPPRLWELTDMSSIFTVPNVRLQPEAGESMRAFGKNNSYGKSLSNSKH

>Hsa-cdk17 gi|37595545|ref|NP_002586.2| cell division protein kinase 17 isoform 1 [Homo sapiens]

MKKFKRRLSLTLRGSQTIDESLSELAEQMTIEENSSKDNEPIVKNGRPPTSHSMHSFLHQYTGSFKKPPLRRPHSVIGGS

LGSFMAMPRNGSRLDIVHENLKMGSDGESDQASGTSSDEVQSPTGVCLRNRIHRRISMEDLNKRLSLPADIRIPDGYLEK

LQINSPPFDQPMSRRSRRASLSEIGFGKMETYIKLEKLGEGTYATVYKGRSKLTENLVALKEIRLEHEEGAPCTAIREVS

LLKDLKHANIVTLHDIVHTDKSLTLVFEYLDKDLKQYMDDCGNIMSMHNVKLFLYQILRGLAYCHRRKVLHRDLKPQNLL

INEKGELKLADFGLARAKSVPTKTYSNEVVTLWYRPPDVLLGSSEYSTQIDMWGVGCIFFEMASGRPLFPGSTVEDELHL

IFRLLGTPSQETWPGISSNEEFKNYNFPKYKPQPLINHAPRLDSEGIELITKFLQYESKKRVSAEEAMKHVYFRSLGPRI

HALPESVSIFSLKEIQLQKDPGFRNSSYPETGHGKNRRQSMLF

>Hsa-cdk18 gi|47078233|ref|NP_997668.1| cell division protein kinase 18 isoform a [Homo sapiens]

MIMNKMKNFKRRFSLSVPRTETIEESLAEFTEQFNQLHNRRNENLQLGPLGRDPPQECSTFSPTDSGEEPGQLSPGVQFQ

RRQNQRRFSMEVRASGALPRQVAGCTHKGVHRRAAALQPDFDVSKRLSLPMDIRLPQEFLQKLQMESPDLPKPLSRMSRR

ASLSDIGFGKLETYVKLDKLGEGTYATVFKGRSKLTENLVALKEIRLEHEEGAPCTAIREVSLLKNLKHANIVTLHDLIH

TDRSLTLVFEYLDSDLKQYLDHCGNLMSMHNVKIFMFQLLRGLAYCHHRKILHRDLKPQNLLINERGELKLADFGLARAK

SVPTKTYSNEVVTLWYRPPDVLLGSTEYSTPIDMWGVGCIHYEMATGRPLFPGSTVKEELHLIFRLLGTPTEETWPGVTA

FSEFRTYSFPCYLPQPLINHAPRLDTDGIHLLSSLLLYESKSRMSAEAALSHSYFRSLGERVHQLEDTASIFSLKEIQLQ

KDPGYRGLAFQQPGRGKNRRQSIF

>Hsa-cdk15 gi|21040235|ref|NP_631897.1| cyclin-dependent kinase 15 [Homo sapiens]

MTSFHPRGLQAARAQKFKSKRPRSNSDCFQEEDLRQGFQWRKSLPFGAASSYLNLEKLGEGSYATVYKGISRINGQLVAL

KVISMNAEEGVPFTAIREASLLKGLKHANIVLLHDIIHTKETLTFVFEYMHTDLAQYMSQHPGGLHPHNVRLFMFQLLRG

LAYIHHQHVLHRDLKPQNLLISHLGELKLADFGLARAKSIPSQTYSSEVVTLWYRPPDALLGATEYSSELDIWGAGCIFI

EMFQGQPLFPGVSNILEQLEKIWEVLGVPTEDTWPGVSKLPNYNPEWFPLPTPRSLHVVWNRLGRVPEAEDLASQMLKGF

PRDRVSAQEALVHDYFSALPSQLYQLPDEESLFTVSGVRLKPEMCDLLASYQKGHHPAQFSKCW

>Hsa-cdk9 gi|4502747|ref|NP_001252.1| cell division protein kinase 9 [Homo sapiens]

MAKQYDSVECPFCDEVSKYEKLAKIGQGTFGEVFKARHRKTGQKVALKKVLMENEKEGFPITALREIKILQLLKHENVVN

LIEICRTKASPYNRCKGSIYLVFDFCEHDLAGLLSNVLVKFTLSEIKRVMQMLLNGLYYIHRNKILHRDMKAANVLITRD

GVLKLADFGLARAFSLAKNSQPNRYTNRVVTLWYRPPELLLGERDYGPPIDLWGAGCIMAEMWTRSPIMQGNTEQHQLAL

ISQLCGSITPEVWPNVDNYELYEKLELVKGQKRKVKDRLKAYVRDPYALDLIDKLLVLDPAQRIDSDDALNHDFFWSDPM

PSDLKGMLSTHLTSMFEYLAPPRRKGSQITQQSTNQSRNPATTNQTEFERVF

>Hsa-cdk13 gi|145309302|ref|NP_003709.3| cell division protein kinase 13 isoform 1 [Homo sapiens]

MPSSSDTALGGGGGLSWAEKKLEERRKRRRFLSPQQPPLLLPLLQPQLLQPPPPPPPLLFLAAPGTAAAAAAAAAASSSC

FSPGPPLEVKRLARGKRRAGGRQKRRRGPRAGQEAEKRRVFSLPQPQQDGGGGASSGGGVTPLVEYEDVSSQSEQGLLLG

GASAATAATAAGGTGGSGGSPASSSGTQRRGEGSERRPRRDRRSSSGRSKERHREHRRRDGQRGGSEASKSRSRHSHSGE

ERAEVAKSGSSSSSGGRRKSASATSSSSSSRKDRDSKAHRSRTKSSKEPPSAYKEPPKAYREDKTEPKAYRRRRSLSPLG

GRDDSPVSHRASQSLRSRKSPSPAGGGSSPYSRRLPRSPSPYSRRRSPSYSRHSSYERGGDVSPSPYSSSSWRRSRSPYS

PVLRRSGKSRSRSPYSSRHSRSRSRHRLSRSRSRHSSISPSTLTLKSSLAAELNKNKKARAAEAARAAEAAKAAEATKAA

EAAAKAAKASNTSTPTKGNTETSASASQTNHVKDVKKIKIEHAPSPSSGGTLKNDKAKTKPPLQVTKVENNLIVDKATKK

AVIVGKESKSAATKEESVSLKEKTKPLTPSIGAKEKEQHVALVTSTLPPLPLPPMLPEDKEADSLRGNISVKAVKKEVEK

KLRCLLADLPLPPELPGGDDLSKSPEEKKTATQLHSKRRPKICGPRYGETKEKDIDWGKRCVDKFDIIGIIGEGTYGQVY

KARDKDTGEMVALKKVRLDNEKEGFPITAIREIKILRQLTHQSIINMKEIVTDKEDALDFKKDKGAFYLVFEYMDHDLMG

LLESGLVHFNENHIKSFMRQLMEGLDYCHKKNFLHRDIKCSNILLNNRGQIKLADFGLARLYSSEESRPYTNKVITLWYR

PPELLLGEERYTPAIDVWSCGCILGELFTKKPIFQANQELAQLELISRICGSPCPAVWPDVIKLPYFNTMKPKKQYRRKL

REEFVFIPAAALDLFDYMLALDPSKRCTAEQALQCEFLRDVEPSKMPPPDLPLWQDCHELWSKKRRRQKQMGMTDDVSTI

KAPRKDLSLGLDDSRTNTPQGVLPSSQLKSQGSSNVAPVKTGPGQHLNHSELAILLNLLQSKTSVNMADFVQVLNIKVNS

ETQQQLNKINLPAGILATGEKQTDPSTPQQESSKPLGGIQPSSQTIQPKVETDAAQAAVQSAFAVLLTQLIKAQQSKQKD

VLLEERENGSGHEASLQLRPPPEPSTPVSGQDDLIQHQDMRILELTPEPDRPRILPPDQRPPEPPEPPPVTEEDLDYRTE

NQHVPTTSSSLTDPHAGVKAALLQLLAQHQPQDDPKREGGIDYQAGDTYVSTSDYKDNFGSSSFSSAPYVSNDGLGSSSA

PPLERRSFIGNSDIQSLDNYSTASSHSGGPPQPSAFSESFPSSVAGYGDIYLNAGPMLFSGDKDHRFEYSHGPIAVLANS

SDPSTGPESTHPLPAKMHNYNYGGNLQENPSGPSLMHGQTWTSPAQGPGYSQGYRGHISTSTGRGRGRGLPY

>Hsa-cdk12 gi|157817023|ref|NP_057591.2| cell division protein kinase 12 isoform 1 [Homo sapiens]

MPNSERHGGKKDGSGGASGTLQPSSGGGSSNSRERHRLVSKHKRHKSKHSKDMGLVTPEAASLGTVIKPLVEYDDISSDS

DTFSDDMAFKLDRRENDERRGSDRSDRLHKHRHHQHRRSRDLLKAKQTEKEKSQEVSSKSGSMKDRISGSSKRSNEETDD

YGKAQVAKSSSKESRSSKLHKEKTRKERELKSGHKDRSKSHRKRETPKSYKTVDSPKRRSRSPHRKWSDSSKQDDSPSGA

SYGQDYDLSPSRSHTSSNYDSYKKSPGSTSRRQSVSPPYKEPSAYQSSTRSPSPYSRRQRSVSPYSRRRSSSYERSGSYS

GRSPSPYGRRRSSSPFLSKRSLSRSPLPSRKSMKSRSRSPAYSRHSSSHSKKKRSSSRSRHSSISPVRLPLNSSLGAELS

RKKKERAAAAAAAKMDGKESKGSPVFLPRKENSSVEAKDSGLESKKLPRSVKLEKSAPDTELVNVTHLNTEVKNSSDTGK

VKLDENSEKHLVKDLKAQGTRDSKPIALKEEIVTPKETETSEKETPPPLPTIASPPPPLPTTTPPPQTPPLPPLPPIPAL

PQQPPLPPSQPAFSQVPASSTSTLPPSTHSKTSAVSSQANSQPPVQVSVKTQVSVTAAIPHLKTSTLPPLPLPPLLPGDD

DMDSPKETLPSKPVKKEKEQRTRHLLTDLPLPPELPGGDLSPPDSPEPKAITPPQQPYKKRPKICCPRYGERRQTESDWG

KRCVDKFDIIGIIGEGTYGQVYKAKDKDTGELVALKKVRLDNEKEGFPITAIREIKILRQLIHRSVVNMKEIVTDKQDAL

DFKKDKGAFYLVFEYMDHDLMGLLESGLVHFSEDHIKSFMKQLMEGLEYCHKKNFLHRDIKCSNILLNNSGQIKLADFGL

ARLYNSEESRPYTNKVITLWYRPPELLLGEERYTPAIDVWSCGCILGELFTKKPIFQANLELAQLELISRLCGSPCPAVW

PDVIKLPYFNTMKPKKQYRRRLREEFSFIPSAALDLLDHMLTLDPSKRCTAEQTLQSDFLKDVELSKMAPPDLPHWQDCH

ELWSKKRRRQRQSGVVVEEPPPSKTSRKETTSGTSTEPVKNSSPAPPQPAPGKVESGAGDAIGLADITQQLNQSELAVLL

NLLQSQTDLSIPQMAQLLNIHSNPEMQQQLEALNQSISALTEATSQQQDSETMAPEESLKEAPSAPVILPSAEQTTLEAS

STPADMQNILAVLLSQLMKTQEPAGSLEENNSDKNSGPQGPRRTPTMPQEEAAACPPHILPPEKRPPEPPGPPPPPPPPP

LVEGDLSSAPQELNPAVTAALLQLLSQPEAEPPGHLPHEHQALRPMEYSTRPRPNRTYGNTDGPETGFSAIDTDERNSGP

ALTESLVQTLVKNRTFSGSLSHLGESSSYQGTGSVQFPGDQDLRFARVPLALHPVVGQPFLKAEGSSNSVVHAETKLQNY

GELGPGTTGASSSGAGLHWGGPTQSSAYGKLYRGPTRVPPRGGRGRGVPY

>Hsa-cdk6 gi|4502741|ref|NP_001250.1| cyclin-dependent kinase 6 [Homo sapiens]

MEKDGLCRADQQYECVAEIGEGAYGKVFKARDLKNGGRFVALKRVRVQTGEEGMPLSTIREVAVLRHLETFEHPNVVRLF

DVCTVSRTDRETKLTLVFEHVDQDLTTYLDKVPEPGVPTETIKDMMFQLLRGLDFLHSHRVVHRDLKPQNILVTSSGQIK

LADFGLARIYSFQMALTSVVVTLWYRAPEVLLQSSYATPVDLWSVGCIFAEMFRRKPLFRGSSDVDQLGKILDVIGLPGE

EDWPRDVALPRQAFHSKSAQPIEKFVTDIDELGKDLLLKCLTFNPAKRISAYSALSHPYFQDLERCKENLDSHLPPSQNT

SELNTA

>Hsa-cdk19 gi|30387611|ref|NP_055891.1| cell division protein kinase 19 [Homo sapiens]

MDYDFKAKLAAERERVEDLFEYEGCKVGRGTYGHVYKARRKDGKDEKEYALKQIEGTGISMSACREIALLRELKHPNVIA

LQKVFLSHSDRKVWLLFDYAEHDLWHIIKFHRASKANKKPMQLPRSMVKSLLYQILDGIHYLHANWVLHRDLKPANILVM

GEGPERGRVKIADMGFARLFNSPLKPLADLDPVVVTFWYRAPELLLGARHYTKAIDIWAIGCIFAELLTSEPIFHCRQED

IKTSNPFHHDQLDRIFSVMGFPADKDWEDIRKMPEYPTLQKDFRRTTYANSSLIKYMEKHKVKPDSKVFLLLQKLLTMDP

TKRITSEQALQDPYFQEDPLPTLDVFAGCQIPYPKREFLNEDDPEEKGDKNQQQQQNQHQQPTAPPQQAAAPPQAPPPQQ

NSTQTNGTAGGAGAGVGGTGAGLQHSQDSSLNQVPPNKKPRLGPSGANSGGPVMPSDYQHSSSRLNYQSSVQGSSQSQST

LGYSSSSQQSSQYHPSHQAHRY

>Hsa-cdk4 gi|4502735|ref|NP_000066.1| cell division protein kinase 4 [Homo sapiens]

MATSRYEPVAEIGVGAYGTVYKARDPHSGHFVALKSVRVPNGGGGGGGLPISTVREVALLRRLEAFEHPNVVRLMDVCAT

SRTDREIKVTLVFEHVDQDLRTYLDKAPPPGLPAETIKDLMRQFLRGLDFLHANCIVHRDLKPENILVTSGGTVKLADFG

LARIYSYQMALTPVVVTLWYRAPEVLLQSTYATPVDMWSVGCIFAEMFRRKPLFCGNSEADQLGKIFDLIGLPPEDDWPR

DVSLPRGAFPPRGPRPVQSVVPEMEESGAQLLLEMLTFNPHKRISAFRALQHSYLHKDEGNPE

>Hsa-CDKL1,gi|37596297|ref|NP_004187.2|cyclin-dependent kinase-like 1 [Homo sapiens]

MMEKYEKIGKIGEGSYGVVFKCRNRDTGQIVAIKKFLESEDDPVIKKIALREIRMLKQLKHPNLVNLLEVFRRKRRLHLV

FEYCDHTVLHELDRYQRGVPEHLVKSITWQTLQAVNFCHKHNCIHRDVKPENILITKHSVIKLCDFGFARLLTGPSDYYT

DYVATRWYRSPELLVGDTQYGPPVDVWAIGCVFAELLSGVPLWPGKSDVDQLYLIRKTLGDLIPRHQQVFSTNQYFSGVK

IPDPEDMEPLELKFPNISYPALGLLKGCLHMDPTQRLTCEQLLHHPYFENIREIEDLAKEHNKPTRKTLRKSRKHHCFTE

TSKLQYLPQLTGSSILPALDNKKYYCDTKKLNYRFPNI

>Hsa-GSK3alpha gi|49574532|ref|NP_063937.2| glycogen synthase kinase-3 alpha [Homo sapiens]

MSGGGPSGGGPGGSGRARTSSFAEPGGGGGGGGGGPGGSASGPGGTGGGKASVGAMGGGVGASSSGGGPGGSGGGGSGGP

GAGTSFPPPGVKLGRDSGKVTTVVATLGQGPERSQEVAYTDIKVIGNGSFGVVYQARLAETRELVAIKKVLQDKRFKNRE

LQIMRKLDHCNIVRLRYFFYSSGEKKDELYLNLVLEYVPETVYRVARHFTKAKLTIPILYVKVYMYQLFRSLAYIHSQGV

CHRDIKPQNLLVDPDTAVLKLCDFGSAKQLVRGEPNVSYICSRYYRAPELIFGATDYTSSIDVWSAGCVLAELLLGQPIF

PGDSGVDQLVEIIKVLGTPTREQIREMNPNYTEFKFPQIKAHPWTKVFKSRTPPEAIALCSSLLEYTPSSRLSPLEACAH

SFFDELRCLGTQLPNNRPLPPLFNFSAGELSIQPSLNAILIPPHLRSPAGTTTLTPSSQALTETPTSSDWQSTDATPTLT

NSS

>Hsa-MAK gi|11496279|ref|NP_005897.1| serine/threonine-protein kinase MAK isoform 1 [Homo sapiens]

MNRYTTMRQLGDGTYGSVLMGKSNESGELVAIKRMKRKFYSWDECMNLREVKSLKKLNHANVIKLKEVIRENDHLYFIFE

YMKENLYQLMKDRNKLFPESVIRNIMYQILQGLAFIHKHGFFHRDMKPENLLCMGPELVKIADFGLARELRSQPPYTDYV

STRWYRAPEVLLRSSVYSSPIDVWAVGSIMAELYMLRPLFPGTSEVDEIFKICQVLGTPKKSDWPEGYQLASSMNFRFPQ

CVPINLKTLIPNASNEAIQLMTEMLNWDPKKRPTASQALKHPYFQVGQVLGPSSNHLESKQSLNKQLQPLESKPSLVEVE

PKPLPDIIDQVVGQPQPKTSQQPLQPIQPPQNLSVQQPPKQQSQEKPPQTLFPSIVKNMPTKPNGTLSHKSGRRRWGQTI

FKSGDSWEELEDYDFGASHSKKPSMGVFKEKRKKDSPFRLPEPVPSGSNHSTGENKSLPAVTSLKSDSELSTAPTSKQYY

LKQSRYLPGVNPKKVSLIASGKEINPHTWSNQLFPKSLGPVGAELAFKRSNAGNLGSYATYNQSGYIPSFLKKEVQSAGQ

RIHLAPLNATASEYTWNTKTGRGQFSGRTYNPTAKNLNIVNRAQPIPSVHGRTDWVAKYGGHR

>Tad-gi|196001971| ref|XP_00211 TRIADDRAFT_23304 hypothetical protein [ Trichoplax adhaerens ] Gene ID: 6751534 TRIADDRAFT_23304

MALDDDSSSESLIEDDAPKPTNIKRYKKIKLLGEGQFAVVYQAEDTHKDNKIVAVKKIKLGPRSEANDGINRTALREIKL

LQELKHENIIGLLDVFGHKSNISLVFDYMEADLEVIIKDSSIILTNAHIKQYIIMTLRGLEYIHSNWILHRDMKPNNLLI

DSNGILKLADFGLARYFGSPNRYYTNQVVTRQVFIGTNLINVWYRAPELLFGANSYGTGVDMWAVGCILAELLLRVPFLP

GDSDLDQLTRIFQTLGTPIEDKWADIHKLPGYIKFKTLPAIPLNEIFTAASDDMLDLLRRLFEYNPSSRITATEALQMKY

FFNLPAPASCSALPRATVDAENDNYDLRKDDMSMKRKVDDEEFGN

>Tad-gi|195996663| ref|XP_00210 TRIADDRAFT_20272 hypothetical protein [ Trichoplax adhaerens ] Gene ID: 6750149 TRIADDRAFT_20272

MESFTILGRVGEGAHGVVFKAKHVQVRGNGEVVALKKIHLKKIDDGIPNNILREIKTLQAITEHENIVRLFDVFPDGSSL

VLAFEYMVTDLSEILRSSQNTLPEVSSYYMIMLLRGISFCHENHIIHRDLKPANLLISSSGQLKLADFGLARVMSVENER

LYSHQVATRWYRAPELLYGSRTYDEGVDLWYLGCIFAELINKSPLFPGDSDIKQLGCVLSILGTPTTSTWPGLIELPDYN

KITFSGFLPIPFESIVPDATSEVGKIK

>Tad-gi|196013334| ref|XP_00211 TRIADDRAFT_31125 hypothetical protein [ Trichoplax adhaerens ] Gene ID: 6757804 TRIADDRAFT_31125

MHNQNLDSIKVKTLEEILQEKRNRSRSDSENIAETEDSPGSQFLTDTINFITKNVTSDTKDETTSAALELDEGEIISSGE

ENLNHIDDTKPTKRNHASQNDELDDYEEGEIKDRDEVERKEEFVPAADSSIISNRQISTTKLPPYLPAVQGCRSVEAFEW

LNRIEEGTYGVVYRAKDLKSDEVVALKRLKMEKEREGFPITSLREINTLLKADHPNIVHVREIVVGSNMDKIYIVMEYVE

HDLKTLMESMSQPFSISEVKCLMKQLLSAVQHLHDNWILHRDLKTSNLLLSHQGILKVGDFGLAREYGSPLKVYTSIVVT

LWYRCPELLLGVKEYSTAVDMWSVGCIFGEFLVKKPLFPGKSEIDQLNKIFKDLGTPNDQIWSGFSELPVAKKVTFTEQP

YNRLRDRFGAYLTDQGFDLLNRFLTYDPKKRISAEDALNHEYFQQEPRPLDPSMFPTWPAKSELMKRPSKANRSPTAPEG

GGMCKIAGAADGFRITYATQGKAAGGAGFNLKF

>Tad-gi|196010283| ref|XP_00211 TRIADDRAFT_28456 hypothetical protein [ Trichoplax adhaerens ] Gene ID: 6756219 TRIADDRAFT_28456

MVDLPYHSIYGNCRNITEFDKLNRIGEGSYGVVYRARDLDSKEIVAIKKIRMENERDGIPVSSLREITLLVNLKHINIVN

LKDVVVGKQLDSIFLVMEYCEQDLSSLLYDNMKAPFTEPQVKCLSLQLIHGVQYLHHNFVIHRDLKVSNLLLTDKGILKV

ADFGLARNYGLPAAPMTPTIVSLWYRAPEVLLGCTKHTLAVDMWSVGCIIAELFDHNVFLKGKSEKDQLDLMCQMLGTPN

EAIWEDIRDMPLYGKIILRQQPYNNLKHKFSWLSAAGLNLLNSLLTYDPGRRITADETLKLSYFRESPLPIEPEMMPTFP

QRRNRSSKE

>Tad-gi|196002669| ref|XP_00211 TRIADDRAFT_23127 hypothetical protein [ Trichoplax adhaerens ] Gene ID: 6752415 TRIADDRAFT_23127

ILKQIGEGTYGQVFKAKIKDTDKLVALKKVRTDHEREGFPITAVREIKILKQLNHQSIVNLLGIVSNVDANNFKTDRCAF

YLVFEYMDHDLMGLLESGLVTFDEEHIRSFMRQIMEGLNYCHKRQLLHRDIKCSNLLMNNKGQIKIADFGLARFYNPDDK

SRPYTNKVITLWYRPPELLLGEERYGPSVDVWSCGCILGEFFTKKPIFQANSEINQLDLISQICGTPCPENWPSVVELPY

YNNFKLRKYERRLEQEFHDLPELAVDLMQYMLILDPSMRYNAEQSLQHPFIRDAPSTPQNLPNFPSQDCHELWYKNLKRQ

KRKEERLEAQSGNKSVVATNNSSLESGRQFTVHSARGSNQSIAGSQVKPANPGGIPSSGITVPKSPPPHGTVCVIFMKRK

WLL

>Tad-gi|195998898| ref|XP_00210 TRIADDRAFT_20854 hypothetical protein [ Trichoplax adhaerens ] Gene ID: 6751094 TRIADDRAFT_20854

MSKEPYNYPFIHDVGKYEKITKIGHGTFGEVFKARNRISKEFVALKRVLLGNEKEGFPITSLREIKILRALKHDNIVRLQ

EICRSKGTPQSRKRGSIYLVFEFCAHDLAGLLQNPQVKFNLSEIKRMMKHLLSGLFYIHSNKVLHRDLKAANVLVTRDGV

LKLADFGLARVYSRKEKTHCFTNRVVTLWYRAPELLLGCRDYGPAIDMWAIGCIMAEFWTRSAIMQGNSETNQLTLITQL

CGSITPEVYPDVDKLDLFKKFDLPASQKRRVKERLSHYVRDRHALDLIDRCLTIDPAKRIDSDSALNHDFFWSDPLPASK

ISCLSRLNVSMFEFLIDRKTTNPQHRPEQSQMSQQPGYDRVF

>Tad-gi|196013348| ref|XP_00211 TRIADDRAFT_50896 hypothetical protein [ Trichoplax adhaerens ] Gene ID: 6757820 TRIADDRAFT_50896

MENFHKLEKIGEGTYGKVYKACNKITGQTVALKKIRLDSDKEGVPSTTLREISILRSLNHSFVVRLYDVVHSDQCLYLVF

EYLDHDLKHYLDHAYKIPPALLKSYLYQMLRAISYCHSRRVLHRDLKPQNLLIDSTGTLKLADFGLARIFGLPVRQYTHE

VITLWYRAPEILLGSTYYSTPVDIWSIGCIFVEMINRRPLFAGDSEIDQLFRVFRTLGTPDEITWPGVSEMSDYKSTFPK

WPSRDLNSVIYSHDEDCVDLIKQMLVYEPNGRISARLALQHPYFRDVLSPDNGSVTELIRLYQKSLSK

>Tad-gi|196003954| ref|XP_00211 TRIADDRAFT_55283 hypothetical protein [ Trichoplax adhaerens ] Gene ID: 6752576 TRIADDRAFT_55283

MDKYLKIEKIGEGTYGVVYKGKNRNTQQLVALKKIRLENEEEGIPSTAIREVSLLKELKHPNIVDLIEVLYEESKLYLVF

EFLDMDLKRYLDTLPKGKTIDAMLMKSYLYQILLGVVYCHSHRVLHRDLKPQNLLINSKGCIKLADFGLGRAFGVPVRVY

THEVVTLWYRAPEVLLGSTRYSCPLDIWSTGTIFAEMWLRRPLFQGDSEIDELFRIFRILGTPDDDIWPGVSSLPEFKSS

FPKWSKQSYDTFVPNMSESGIDLLSKMLIYDPANRISGKRALSHPYFDDLDKSTLPTDRWS

>Tad-gi|196001193| ref|XP_00211 TRIADDRAFT_50028 hypothetical protein [ Trichoplax adhaerens ] Gene ID: 6752216 TRIADDRAFT_50028

MKKLRRRLSATLLGLNNDGSATGSAFGTNTTFRPKLFPFRRYSSMYESETVRQKINAAKETIENSGNSKQISNQRRHSAA

IESPSIPFGTEDAYEKLEPLGEGSYATVYKGYSIGHQKLVALKEITLNEEEGTPFTAIREASLLKQLKHANIVVLHDIIQ

TPTKLTFVFEYVTTDLSQYLNLHPGGLNMKNVKLFLYQLLRGLSYCHQRRILHRDIKPQNILVSEIGEIKLADFGLARAK

SVPSKTYSPDVVTLWYRPPDVLLGSTNYSTSLDIWGVGCIFTEMVSGIATFPGVRNISDQLDKIWHKLGTPTEETWPGVT

SYPEYCAAETMFYESRTIAEVIPRLMKDPGAADLATKMLQYQPFKRIFCQAAMNHQYFTDIPEGIIKLPPTVSIFCNGLV

KLAPEENEFEGDSLTSVIS

>Tad-gi|195996637| ref|XP_00210 TRIADDRAFT_20204 hypothetical protein [ Trichoplax adhaerens ] Gene ID: 6750135 TRIADDRAFT_20204

SELGFGKLESYDKLHKLGEGTYATVFKGRSKLTNDFVALKEIRLEHEEGAPCTAIREVSLLKDLKHANIVTLHDTIHTER

SLVLVFEYLDRDLKQYMDSCGSILDMSNVKIFLFQLLRGLAYCHSRRVLHRDLKPQNLLINERGELKLADFGLARAKSVP

SKTYSSEVVTLWYRPPDVLLGSTEYSTSIDMWGVGCIFYEMATGLPMFPGSSTENQLQTIWEILGTPTEEEWSGLTRNLK

VNSLSFHDCKGEPLRNRAPRLEADGLDLLAKFLQYKAKSRISSADAMKHKYFDCFGPEVQILKDSKILFNSS

>Tad-gi|196000717| ref|XP_00211 TRIADDRAFT_49937 hypothetical protein [ Trichoplax adhaerens ] Gene ID: 6751441 TRIADDRAFT_49937

MESYEKLVKIGEGTYGTVYKAVNHDTGEIVALKKVRIDDENEGIPSFALREICLLKELKHKNIVMLYDVIHGNKELMIVF

EYCDQDLKRYCDACQGKIDPSIVQSFTNQLLQGLAYCHSHHILHRDITPQNILVTGNGDIKLADFGLARNFGIPVKSFSA

EVVTLWYRSPDVLLGATLYDTSIDIWSTGCIFAELSNGGQPLLPGKDVADQLKIIFKIFGTPNEQIWPGVSQLMKDKDYP

SYNAMSILHVVPNLNQLGCDLFQLMMVLDPSKRCTAEQALQHAYFKGVSEGYT

>Tad-gi|195999760| ref|XP_00210 TRIADDRAFT_52937 hypothetical protein [ Trichoplax adhaerens ] Gene ID: 6750388 TRIADDRAFT_52937

MENYVQVTEIANGAYGRVYKARDLNHNRLVALKKIAVINDEQGIPISTIREITSLKSLQSFGHQNIVRLYDIFANYTVDR

RTMDLTLVFEHVEQDLQTYIRNCPSAGIDTRKIKDIIYQIVNAIDFLHANRIVHRDLKPQNVLISRQGIVKVADFGLAKV

FCENVPITSVVVTLWYRCPEVLLQSTYATPVDMWSVGCIMAELYLREPLFCGQTDIDQLQKIFSMTGLPDESEWPVNIPF

SRSTFCQYTRRPVQYSEMMPEICQDGVDLLQKLLKFNPKERLTAEESLQHPYFQDVLPSEKSDNHGDVNGRQ

>Tad-gi|196015356| ref|XP_00211 TRIADDRAFT_32638 hypothetical protein [ Trichoplax adhaerens ] Gene ID: 6758748 TRIADDRAFT_32638

MDPEFKNRLEKKRRKVQDTFEFEGCKVGRGTYGHVYKAKMKSSGKEYALKLIEGSGISMSACREIALLREVHHPNVISLQ

GVFLTHTNRKVWLLFDFAEHDLWHIIKYHRSKAGDKAISLDSKMVKSLLKQILEGIHYLHANWILHRDLKPANILVMGEG

PERGRVKIADMGFARHYWSPLKPLAELDPVVVTSWYRAPELLLGARHYTKAIDIWAIGCIFAELLTSEPIFHCRQEDFKT

SNPYHRDQLERIFMVMGYPHEKDWEDIKKTPNYAKLASDFRKISTYTNCSLAKYMDKFKIKQDSKEFILLQKCLTIDPNK

RISSEQAIDDAYFREEPLPTEDVFDHKTIPYPKREFLNEDDNEDKGDKHKVYCDGVDYIY

>Tad-gi|196015366| ref|XP_00211 TRIADDRAFT_32711 hypothetical protein [ Trichoplax adhaerens ] Gene ID: 6758753 TRIADDRAFT_32711

MDPQFKKILQQKRQNVEDLFDFEGCKVGRGTYGHVYKAKMKTSGKEYALKLIEGTGISMSACREIAILREISHTHVISLQ

GVFLTHASRKVWLLLDFAEHDLWHIIKFHRPKGGKKSVPIDTKIVKSLLRQILDGIQYLHSNWILHRDLKPANILVMGDG

LERGRVKIADMGFARHFWAPLKPLADLDPVVVTFWYRAPELLLGARHYTKAIDIWAIGCIFAELLTSEPIFHCRQEDLKA

TTPYHHDQLDRIFTVMGFPHERDWEDIKIMPEYKRLQEDFKKPSYVNFASCSLAKYMDKFKIRHDSREFSLLQKFLIADP

NKRISAELAMDDAYFREEPYPTDDVFDGKPIPYPKREFLHDDEHEEKHDKNKVRHCVFIFSPEIIQI

>Bfl-gi|260821968| ref|XP_002606375.1| hypothetical protein BRAFLDRAFT_118518 [Branchiostoma floridae] BRAFLDRAFT_118518

MAADRSKRYQKIDFLGEGQFATVYKAKDTKTGQIVAVKKIKLGQRAEAKDGINRTALREIKLLQELSHTNIIGLYDVFGH

KSNISLVFDFMDTDMEVVIKDTSIVMTGPHIKAYAIMTLEGLEYLHNNWILHRDLKPNNLLVNSQGILKITDFGLAKTFG

SPNRVYTHQVVTRWYRAPELLYGARIYGTGVDMWAVGCILAELLLRVRTDIDIRCRFL

>Bfl-gi|260799041| ref|XP_002594508.1| hypothetical protein BRAFLDRAFT_124968 [Branchiostoma floridae] BRAFLDRAFT_124968

MEQYTIMERIGEGAHGIVFKAKHVESGEVVALKKVPLRRLEDGIPNTALREIKALQEIEENQHVVKLREVFPHGTGFVLV

FEYMLSDLSEVLRNSNRPLTEAQVKSYMMMLLKGVAFCHENNIMHRDLKPANLLISETGHLKIADFGLARVFANEDGRLY

SHQVATRWYRAPELLYGARKYEEGVDLWAVGCIFGELLNNSPLFPGENDIEQLCCVLRVLGTPNEKIWPGMSELPDYNKI

TFPENPPIPLEVVVPDASPQAIDLLKRFLVYPSSQRVSAKEALLHPYFFTEPLPAHHSELPIPQRSSRRHRQRGHVHEFD

VDAPLAQSLVDPDLLAPHVR

>Bfl-gi|260838232| ref|XP_002613753.1| hypothetical protein BRAFLDRAFT_114825 [Branchiostoma floridae] BRAFLDRAFT_114825

MEDYVKIEKIGEGTYGVVYKGRNKKTGLTVALKKIRLESEEEGVPSTAIREISLLKELVHPNIVNLQDVLMQESKLYLVF

EFLTMDLKKYMDSIPSGQYMDSMLVKSYLYQILQGITFCHSRRVLHRDLKPQNLLIDNKGIIKLADFGLARAFGIPVRVY

THEVVTLWYRAPEVLLGGARYSTPIDIWSIGTIFAEMATKRPLFHGDSEIDQLFRIFRTMGTPTEDIWPGVTQMPDYKPS

FPSWKTNPNQLKTSVKNMDDQALDLLQKTLIYDPANRISAKAALIHPYFDDLDKASLPGTNFKPC

>Bfl-gi|260818988| ref|XP_002604664.1| hypothetical protein BRAFLDRAFT_228782 [Branchiostoma floridae] BRAFLDRAFT_228782

HQLGKCRSVQEFEKLNRLGEGTYGIVYRARDTRSGEIVALKKMRMDREKDGLPISGLREITLLLNVTHRNIVDLKEVVVG

TKSLESIFLVMEYCEQDLASLLDNMDTPFSEAQVKCIMLQVFNGLEYLHDNFIIHRDLKVSNLLMTDKGCIKIADFGLAR

TFGLPPKPMTPRVVTLWYRSPELLLGAKTQTTAVDMWAAGCIFGELLAHKPLLPGRSEIHQLELIVELLGTPSEAIWPGF

SQLPALEQISLKKQPYNNLKHRFPWLSEAGLRLLNFLFMYDPKKRGSAEECMKSSYFKEKPLPTEPELMPTFPHHRNRRR

SVQRPEARSGMVAL

>Bfl-gi|260787719| ref|XP_002588899.1| hypothetical protein BRAFLDRAFT_115150 [Branchiostoma floridae] BRAFLDRAFT_115150

MQKYEKLEKIGEGTYGTVFKAKNKESHEIVALKRVPLDDDHEGVPSSALREICLLKELKHKNIVRLHDVLHSEKKLTLVF

EYCDQDLKKYFDSCSGDIDPETVKSFMYQLLRGLAFCHSHNVLHRDLKPQNLLINKNGELKLADFGLARAFGIPVRCYSA

EVVTLWYRPPDVLFGAKLYSTSIDMWSAGCIFAELANAGRPLFPGNDVDDQLKRIFRYPFMGFTFCQFFKNLHEDTWPGM

SRLPDYKPFPIYQVTTSLAVVVPKLCPKGRDLLQRLLVCNPAHRLSADEGLNHPYFNDLSSAVRTG

>Bfl-gi|260789724| ref|XP_002589895.1| hypothetical protein BRAFLDRAFT_281557 [Branchiostoma floridae] BRAFLDRAFT_281557

MGCRNVEEFSCLNRIEEGTYGVVYRAKDKKTGEIVALKRLKMEKEKEGFPITSLREINTLLKAQHPNIVTVREIVVGSNM

DKIYIVMDYVEHDLKSLMETMKQPFLVGETKTLLIQLLRAVQHLHDNWILHRDLKTSNLLLSHKGILKVGDFGLAREYGS

PLKPYTPIVVTLWYRAPELLLGVKEYSTHIDLWSVGCIFAEFLTMKPLWSGKSEIDQLNKIFKDLGTPSERIWPGYNELP

AVKKCTFAEYPYNNLRSRFGAYLSDLGFELLNKFLTYCPSKRITAEDALKHEFFRESPQPVDPSMFPTWPAKSEQTTTRV

KRGSSPRPPEGGLGYAQQLAEDGDIEPGLGFHFNTSQQGVSAKGAGFNLKF

>Bfl-gi|260797491| ref|XP_002593736.1| hypothetical protein BRAFLDRAFT_259646 [Branchiostoma floridae] BRAFLDRAFT_259646

MSSQASSINAVRDKLEAIEYPYCDDVSKYEKLAKIGQGTFGEVFKARHRKTKQFVALKKVLMENEKEGFPITALREIKIL

QMVKHENVVQLLEICRTKASPLNRFKGSIYLVFDFCEHDLAGLLSNANVKFTLSEIKKVMQQLLNGLYYIHRNKILHRDM

KAANILINKHGVLKLADFGLARAFSVTKSGQANRYTNRVVTLWYRPPELLLGERNYGPPIDLWGAGCIMAEMWTRSPIMQ

GNTEQHQLTLISQLCGSISAEVWPSVEKLDLFSKLELPKGQKRKVKERLRAYVKDPYALDLIDRLLTLDPTKRIDSDDAL

NHDFFWEDPLPVDLQKMLSMHNTSMFEYWAPPRRRGHAHGPQAPGRNQPANNDATYDRVF

>Bfl-gi|260807641| ref|XP_002598617.1| hypothetical protein BRAFLDRAFT_118351 [Branchiostoma floridae] BRAFLDRAFT_118351

MPPSKIKLAFASSKSSTKDDDQNMAQDDMGDVGGNQITLKHGLSSMDSARITDCGDSSSLVINSYENDITSVEMKRKKEA

DDWKLREEDDKENSEEWWPELLDITNEPSPEETQTAKCSTPGQFTNICGGKDTRRCLTDGCVQTVLSHPNQEICQDSAEM

ETQEDDNNQIISNCHPIPVIQPAFVCRTRDSGMHQSTPGESFSTVNNNVNIPVQLQCLTENSDSLLADGSQVENVLHKED

NYSAVPLVNSSTYKEASQQGLVTNPPPCQPASTVYNYAFLNLINEGTYGVVYKAMHKTTGDVVAIKMLKSENQPHGVSGT

GLREVNIMSKARHINVISLREVVYGNNIDKAYLVMEYAETDLKQLMYNLQRPFSVSETKGLLVQLLYAVQYLHDKDILHR

DIKTENLLLNLHGILKVTDFGLARTFSKGDKHLSPVVVTLWYRAPELLLGSKTYSTPVDLWSVGCVFAELLTGNPFWDGE

SEIDQLHQIFCDLGTPSEKIWPGYSRLPFLKTCILPDFPYNRLRRRLGWTLTELGLHLLNWFLTYSPARRVTAVQALQHC

EKMSSIHTEEVQRQLREVLNRDKVKLWESPYSREDGTADQIPENLILKYSDELRLSKDVVASNMEELRLHAVRKLASRRK

FQTSGVATLKVKLAGKHIGNNAQVLVICLSESEAEAQRKEEQMSKVRRAREGAELLANTFSA

>Bfl-gi|260809423| ref|XP_002599505.1| hypothetical protein BRAFLDRAFT_265807 [Branchiostoma floridae] BRAFLDRAFT_265807

MIEWGERCVDVFEILSQVGEGTYGQVYKAKDKQTKEVVALKKVRLDNEKEGFPITAVREIKILRQLCHRSIVNLKEIVTD

KSDALDFRKDKGAFYLVFEYVDHDLMGLLESGLVQFNEDQIKSMMKQLMQGLDYCHKKNFLHRDIKCSNILINNRWQVKL

ADFGLARLYHAEEARPYTNKVITLWYRPPELLLGEEQYGPAIDIWSCGCILGELFTRKPIFQANQEPAQLELISRICGAP

CPAVWPDVIKLPYFHTIKPKKQYRRRLREEFAYFPTPALDLMDHMLTLDPSKRCTADQALESSWLKGVDVDRGLTQELPT

WQDCHELWSKKRRR

>Bfl-gi|260829231| ref|XP_002609565.1| hypothetical protein BRAFLDRAFT_241390 [Branchiostoma floridae] BRAFLDRAFT_241390

VPLCVHCEPFLLSSISRLLDICHGTVAEKETKLTLVFEHVDQDLHTYLEKCPAPGLGPDRIKDIMHQLLSGVEFLHMHRV

VHRDLKPQNILVTSTGQVKLADFGLARHYSFQMALTSVVVTLWYRSPEVLLQASYATPVDIWSVGCIFAELHTRKPIFQG

NSDIDQLNKIFDVIGTPIKEEWPEEVSLPWSSFQPRPGIPLESLLPEVEPLAKDMLEKMLCFNPHRRITAKDALQHAYI

>Bfl-gi|260783497| ref|XP_002586811.1| hypothetical protein BRAFLDRAFT_243130 [Branchiostoma floridae] BRAFLDRAFT_243130

MDYEFKLKTAAQREKVEDLFDYEGCKVGRGTYGHVYKAKRKDGSDDKEYALKQIEGTGISMSACREIALLRELKHPNVIT

LHRVFLSHSDRKVWLLFDFAEHDLWHIIKWHRASKANKKPVPIPRQMVKSLLYQILDGIHYLHANWILHRDLKPANILVM

GEGPERGRVKIADMGFARLFNSPLKPLADLDPVVVTFWYRAPELLLAARHYTKAIDIWAIGCIFAELLTSEPIFHCRQED

IKTSNPYHHDQLDRIFNVMGFPQEKDWEDIRKMPEHATLMKDFRRSQ

>Bfl-gi|260783491| ref|XP_002586808.1| hypothetical protein BRAFLDRAFT_174614 [Branchiostoma floridae] BRAFLDRAFT_174614

MDYEFKLKTAAQREKVEDLFDYEGCKVGRGTYGHVYKAKRKDGSDDKEYALKQIEGTGISMSACREIALLRELKHPNVIT

LHRVFLSHSDRKVWLLFDFAEHDLWHIIKWHRASKANKKPVPIPRQMVKSLLYQILDGIHYLHANWILHRDLKPANILVM

GEGPERGRVKIGMDLLYGSFIKNFSLLRYPLAADMGFARLFNSPLKPLADLDPVVVTFWYRAPELLLAARHYTKAIDIWA

IGCIFAELLTSEPIFHCRQEDIKTSNPYHHDQLDRIFNVMGFPQEKDWEDIRKMPEHATLMKDFRRSQYQNCCLLKYMEK

HKVKADSKAFHLLSKLLTMDPTKRISSDQAMADPYFMEDPQPTSDVFAGVPIPYPKREFLTDEDNDDKTD

>Cin-gi|198412574| ref|XP_002121840.1| LOC100182642 similar to cyclin-dependent kinase 7 [ Ciona intestinalis ] Gene ID: 100182642 LOC100182642

MASKSSRYEKIEFLGEGQFATVYKAKDTKDDDRIVAVKKIKLGNRAEAKDGINRTALREIKLLQELHHENIIGLLDVFGQ

KSNISLVFDFMETDLEVIIKDMSIVLTQAHIKSYMIMTLHGLEYLHSLWILHRDLKPNNLLFDSHGVLKIGDFGLAKTFG

SPSREYTHQVVTRWYRSPELLFGSRLYGVGVDMWAVGCILAELLLRVPFLPGDSDLDQLSKIFETLGTPSDAEWPGMKDL

PDYICFKEFPGIPLSQCFSAARDDLLELISGLLRYNPGLRVTAVQALHFSFFTNQPYPTAYQNLPLPASCSSVANKQPKA

VAGTKRKRDNDTDSMAGLAKKLVF

>Cin-gi|198420048| ref|XP_002119796.1| LOC100179255 similar to cell cycle related kinase [ Ciona intestinalis ] Gene ID: 100179255 LOC100179255

MEQYHILGRIGEGAHGIVFKAKHIERGEVVALKKVPLRKIEDGIPNQALREIKALQEIGGDSEDAQNVVKLHDVFPHGTG

FVLVFEYMLSDLSEVIRNSERSLTESQIKSYMMMLLKGVAFCHQNNIMHRDLKPANLLISSTGHLKIADFGLARVFDNDA

ERLYSHQVATRWYRSPELLYGARRYDEGVDLWAVGCIFGEMLNNSPLFPGENDIEQLCCVLRVLGTPNETIWPGMSILPD

YNKITFPENPPIPLEEIVPDASEDALDLLKKFLVYPSNQRIAATHALLHPYFFTEPLPAHHSELPIPQRARKSSRHRSNF

KEYDIDVPIEHSCIDPALIAEFANV

>Cin-gi|198425580| ref|XP_002131194.1| LOC100185295 similar to Cdc2 homologue [ Ciona intestinalis ] Gene ID: 100185295 LOC100185295

MALHDDYVKIEKIGEGTYGVVYKGRNKKTNQIVALKKIRLESEEEGVPSTAIREISILKELQHPNIVSLQDVVLQESNLF

LVFEFLQMDLKKYMDTIGSGKYMDKDLVKSYTYQILQGITYCHSRRVLHRDMKPQNLLIDRNGIIKLADFGLARAFGIPV

RVYTHEVVTLWYRAPEVLLGSSRYSTPVDVWSIGTIFAEMATKRPLFHGDSEIDQLFRIFRVLGTPTDDIWPGVTQLKDY

KQTFPKWKKGCLNDSVKNLDEDGIDLLTKCLVYNPAKRISAKVALCHPYFDDIDKKALPGTHVPLVPRIAG

>Cin-gi|198422386| ref|XP_002129982.1| LOC100181289 similar to cyclin-dependent protein kinase 5 [ Ciona intestinalis ] Gene ID: 100181289 LOC100181289

MHKYEKIEKIGEGTYGTVFKAKNRESGEVVALKRVQLDDDDEGVPSSALREICILKELKHKNVVRLHDVLHSERKMTLVF

EYCEQDLKKYFDSCGGEIDRPTVQSFMYQLLKGLAFCHQQNILHRDLKPQNLLINKNGELKLADFGLARSFGIPVRCYSA

EVVTLWYRPPDVLFGAKLYSTTIDTWSAGCIFAEISNAGVPLFPGNDVEDQLKRIFKVLGTPTEQSWPGVSKLPDFKIFP

LYPSNAHWAAITPRLSSSGHDLLKCLIVANPSERLTASNALKHRYFDDIHTS

>Cin-gi|198413913| ref|XP_002130670.1| LOC100184330 similar to PITSLRE protein kinase beta 1 [ Ciona intestinalis ] Gene ID: 100184330 LOC100184330

MEVRRISPQGKKLEILKKNKDIVEEIVSSENSSDSSASSDSESENEKPEVEEQDEEEETNIQDESATQSDEETAASSSNS

SSAEEVGESANSEDDESASEESGDESAEMSAEEEDPSSRFDSPGKDDRLDEDEEHNDEDANTPEHEVDVHEEIQDPELPF

YLPAIQGCRSVDEFQCLNRIEEGTYGVVYRAKDKKTDNVVALKRLKMEKEREGFPITSLREVCTLLKAHHPNCVRVQEIV

VGSNVDKIYIVMDYVEHDLKSLMETMKQPFLTGEVKTLMIQLLQGVHHLHDNWILHRDLKTSNLLLSHRGILKIGDFGLA

REYGSPLKPYTPIVVTLWYRCPELLLGAKEYSTAVDMWSVGCIFAEFLNKKPLFPGKSETMQLNLIFKELGTPSEKIWPG

YNDLPIVKKTTFVEYPYNTLRKRFGATDISQKGFDLLNRFLTYSPERRISAYNALKHDWFLETPKPVEPSMFPTWPAKSE

LDKKRAGPAASSPPKAPAGAMGYSKLINEEEKLGFQLTAPKHGISKQGMGFSLKF

>Cin-gi|198420046| ref|XP_002119686.1| LOC100183111 similar to cyclin-dependent kinase 10 [ Ciona intestinalis ] Gene ID: 100183111 LOC100183111

MEDNEIVELFTGKKTKIPEKDRYGECRDVTTFEKLNRIGEGTYGIVYRARDKVSKEIVALKKVRTENEKEGISISSIREI

TLLLNLKHKNIVELKEVVVGQRLDSIFLVMEYCEQDLANLLDNMTTPFSEAQVKCITLQLLRGLAFLHESFIIHRDLKVS

NLLMTDGGVLKIADFGLARLYSIPQTSMTPRVVTLWYRAPELLFGATKYTKSIDTWAAGCILAELLAHKPIFPGKSEIEM

IELLIQMLGSPSEEIWPGFSELPAIKTIYLKKQPYNNLKHRFPWVSEAGLRLLNLMLLYNPSKRISAQDCIEMSYFKENP

HPCGPDMMPTFPHHRNKRKSNTEHKTVPSKRRESLGGCSKHA

>Cin-gi|198436212| ref|XP_002131308.1| LOC100179889 similar to GK20517 [ Ciona intestinalis ] Gene ID: 100179889 LOC100179889

MKKFRRRFSEKITKLSAASSTYEDSVVEEDASSDVEKSSQFVHSKINNCTPIKENVQRLPSLSEEEIDDSVLRKRFLSAT

TSSDVWNQSTNHEKIARRHSSPAWGIGELSPYGKLETYKKLGVLGEGSYATVYKGQSKHTGQLVALKEISLNAEEGAPFT

AIREASLLKTLKHANIITLHDIVHATTTLTLVFEYMVTDLSTYMEWYGSCGIHPSNAVLFTFQLLRGLDYCHQRRILHRD

LKPQNLLLSDLGELKLADFGLARAKSIPTNTYSNEVVTLWYRPPDVLLGSRNYTTSLDMWGVGCIFLEMLTGMPVFPGHS

DANDQLTKIFKVLGTPTPQTWRKLPSFPCYEDYSSCFTPVRNRVTFDETFTKISRIEFGVEFALDLLQFEPDNRLSGAEA

LHHPIFHHFPDNIHTIPDRASVINIPGVRLMKEETIPSSESVKFTIG

>Cin-gi|198436214| ref|XP_002131391.1| LOC100177537 similar to Cdc2-related kinase, arginine/serine-rich [ Ciona intestinalis ] Gene ID: 100177537 LOC100177537

MPLSRHPAPLQESKWEKKHQNQEKNIKSVKPLVAYQSFSSSSDSDSKPLRNKVEKVVKTVSKSVKSDQHKSSQRTVTIKS

SVIPTTSKREVKVKVESKEHKRSKDKKEFRFNSNHTNTSKQRKEKSKESSTRRKSSSSKSTADRNFSDFTSESEENCDAM

DRRPHHPYDARGYPPRSPFRRHNDGWGRYDGPRSPPHGYPPRYVQRPALPIIRRSVTPPHSPRYHSPPSRREVYNEPRFM

DQDRRFDNQSPHYGHPMHSPRQKSGFGSGNQDIDMRPETAALNPHIDMLHNTSLNSKQNITAHPRTDERKSRDRLPQDEN

QRKSAARDRSSSRSHSRSKHRKHKKHKRSRSSHHRHHSKRSRSKRHSSSNSSAESTDSNNDDQHSDSSQTTRSSQKSKTE

ALTSNLMTVKDLAAQLSENRKKLKEEEEKSHRKRKHKHHKRRKDKKRKTSRKRKAESSESHSEVENEIHSVPLPSEATLT

HNEQNTSINQQVLVTVSSNEVPQAQDNEWKSPIQTPPLPNDLLAVAMDAHTKDVDHRVAMQHAQSIKFEEMASKVNSSVK

DEISSALESSAFPNRLVEPDPPADQSATSDLQPTNDKKPVKEDVVPTKKGLHDLPLPPILPSLSTTDDEPAHTPPHSDKD

QVKKKRPKICGPRTREVVQTENDWGSMCVDEYAFISITGEGTFGQVYKARDKHTDEICALKKVRLDNEREGFPITAVREI

KILRQLQHRNIVCLKDVLTDKSDATDFRKEKECAFYLVFEYMDHDLMGLLESGMVHFNENHIKSFMKQLLDGLNHCHKKG

FLHRDIKCSNILLNNKGEIKLADFGLARFFNKDEQRPYTNRVITLWYRPPELLLGEEMYTPSIDIWSCGCILAELFTKKP

LFQADRELAQLECISRVCGSPCPAVWPDVIKLPHFHTMKPKRQHRRKLREDFSYLPTLAIDLLDQMLTLDPSKRFTAEEA

LNCPWLKNVDTKNMTMPDFPHWQDCHEMWSKKRRKEMRETARLAEGKSATNDKKPAVKKDTDLRSNKSSDTSNNSSSQPE

ASSAASADNKSSEQDALVRLMQEKPDMNLAQLAKAWKMPIDEESVKLLSSVNIKVLFAALSKKNVKDTSSEGLTGLLDQL

QKTLGNAVKNSAKEEPPTQQVNVAPLPSRPGLQGPSSHETSERSVIASSQGKHKRTTKDGERRTEVQKNDHGHKTNEIHD

KFKVENHNPHHHHSSRAGNLNQSSHSSNEMKHPSASYQSAERRSEKRDSRSDKQFFYSNGRHNS

>Cin-gi|198427109| ref|XP_002131004.1| LOC100181821 similar to cyclin-dependent kinase 9 (CDC2-related kinase) [ Ciona intestinalis ] Gene ID: 100181821 LOC100181821

MAKGLDPNMDFPFCQDVLKYERITKIGQGTFGEVFKARDRKTDRLVALKKVIMENEKEGFPITALREIKILQLLKHENVV

DLIEICRTKPTQYNRSKGSIYLVFEFCAHDLAGLLSNATVKFTLGEIKKTMLQLLEGLFYIHRNKILHRDMKAANILITK

NGVLKLADFGLARAFSYTKTGQANRYTNRVVTLWYRPPELLLGDRDYGPPIDLWGAGCIMTEMWTRSPIMQGHTEQQQLT

LISQLCGSITKQVWPGVEKYDLFTKMELPTGQKRRVKERLKAYVRDQYALDLIDKLLSLDPKHRIDSDEALNHDFFWTEP

LPCDLTNMLSQHKTSMFEYLAPPRKPTTSAAHPHYRNPPAQAARQQPTDPAYDRIF

>Cin-gi|198431481| ref|XP_002131701.1| LOC100175205 similar to cyclin-dependent kinase [ Ciona intestinalis ] Gene ID: 100175205 LOC100175205

MTSEQSHDADEAPSARASQSSLHAEPTSSPWTNQRYHTLSEIGVGAYGVVFKAQDMQSDDENKFVAIKCVRVENSEQGMP

LSTVREIALLRQLESCEHPNVVRLLDICAGHHSKRETQLMLVFEYVDQDLDVFLKKCPDSGLEPEKVTDIMRQMLGGLDF

LHSMRVVHRDMKPQNILITTTGQVKIADFGLARIYSIDMALTEVVVTLWYRSPEVLLLDSYATPVDIWSAGCIFAELFNR

RPLFRGTGDVNQLKKIFDFIGRPSEAEWPLNVAVPIDSFPPQPPRPPQKFVPSITEDAADFLMKLLVFDPGKRLTAQSAL

QHKYVQKASTSNEPNFLSSAVNQAVMYGSTSQGGSSSSHRRLPYTKQMTTLQNHVSRSYDERYDSSHRGVDCPDSSRRSR

VTPAMENITTDDVKTPTNDDQKQSLDDRLSGRRKDKSSSHSASPSQHHQDESEFLTPPSDLASSSSGFMTSSSSRSSSPS

ESGVPSFVTDSTQQSSTFEVETSLRRSVTPAERIDEVQLRPKKSTDIDDQSRMSWAFEPITDAESQLEIESQIQSKNQIT

QFKTPTKTPTYQMLESGKYVPYVTQKPHTEASHAICGHSSQSSYSSSEGDSQASQPRPEK

>Dme-cdk7-gi|17530793| ref|NP_511044.1| Cyclin-dependent kinase 7 [Drosophila melanogaster] Gene ID: 31441 Cdk7

MLPNANDKTERYAKLSFLGEGQFATVYKARDTVTNQIVAVKKIKKGSREDARDGINRTALREIKILQELQHENIIGLVDV

FGQLSNVSLVFDFMDTDLEVIIKDNKIILTQANIKAYAIMTLKGLEYLHLNWILHRDLKPNNLLVNSDGILKIGDFGLAK

SFGSPNRIYTHHVVTRWYRSPELLFGARQYGTGVDMWAVGCILAELMLRVPFMPGDSDLDQLTRIFSTLGTPTEAEWPHL

SKLHDYLQFRNFPGTPLDNIFTAAGNDLIHLMQRLFAMNPLRRVSCREALSMPYFANKPAPTVGPKLPMPSAILAAKEGA

NPQTGDTKPALKRKLVETTVRGNGLAQKKRLQF

>Dme-cdc2-1-gi|17738075| ref|NP_524420.1| cdc2c, isoform B [Drosophila melanogaster] cdc2c

MTTILDNFQRAEKIGEGTYGIVYKARSNSTGQDVALKKIRLEGETEGVPSTAIREISLLKNLKHPNVVQLFDVVISGNNL

YMIFEYLNMDLKKLMDKKKDVFTPQLIKSYMHQILDAVGFCHTNRILHRDLKPQNLLVDTAGKIKLADFGLARAFNVPMR

AYTHEVVTLWYRAPEILLGTKFYSTGVDIWSLGCIFSEMIMRRSLFPGDSEIDQLYRIFRTLSTPDETNWPGVTQLPDFK

TKFPRWEGTNMPQPITEHEAHELIMSMLCYDPNLRISAKDALQHAYFRNVQHVDHVALPVDPNAGSASRLTRLV

>Dme-cdk5-gi|17137070| ref|NP_477080.1| Cyclin-dependent kinase 5 [Drosophila melanogaster] Gene ID: 36727 Cdk5

MQKYDKMEKIGEGTYGTVFKGRNRDTMEIVALKRVRLDEDDEGVPSSALREICLLKELKHKNIVRLIDVLHSDKKLTLVF

EHCDQDLKKYFDSLNGEIDMAVCRSFMLQLLRGLAFCHSHNVLHRDLKPQNLLINKNGELKLADFGLARAFGIPVKCYSA

EVVTLWYRPPDVLFGAKLYTTSIDMWSAGCILAELADAGRPLFPGSDVLDQLMKIFRVLGTPNEDSWPGVSHLSDYVALP

SFPAITSWSQLVPRLNSKGRDLLQKLLICRPNQRISAEAAMQHPYFTDSSSSGH

>Dme-cdc2-gi|17136606| ref|NP_476797.1| cdc2 [Drosophila melanogaster] cdc2

MEDFEKIEKIGEGTYGVVYKGRNRLTGQIVAMKKIRLESDDEGVPSTAIREISLLKELKHENIVCLEDVLMEENRIYLIF

EFLSMDLKKYMDSLPVDKHMESELVRSYLYQITSAILFCHRRRVLHRDLKPQNLLIDKSGLIKVADFGLGRSFGIPVRIY

THEIVTLWYRAPEVLLGSPRYSCPVDIWSIGCIFAEMATRKPLFQGDSEIDQLFRMFRILKTPTEDIWPGVTSLPDYKNT

FPCWSTNQLTNQLKNLDANGIDLIQKMLIYDPVHRISAKDILEHPYFNGFQSGLVRN

>Dme-pitslre-gi|24667662| ref|NP_649251.2| pitslre, isoform A [Drosophila melanogaster] Pitslre

MVNSSGSEDGQLRSPNDVHYHSRGEEDEHEGDADALYIQPPQASRESGSGPRREKKKHSRERRRHKERDDVGGAALALER

DHRYDYRSREEHYHHHQRERSSNAAAAYAKHHLGHAYHYPQPPQQQQQPLPPAPSYAAHHYHHHQHLSGARAAPREYHSY

PSGYHSGSRHGDYPMEEPTRRSSKYAESKDAESLEQDLRSRLLKKRHNYVKDYETEENYEHRVERSDRREGGRKERERTV

RSTHKQNRHDRVIELLDSPEQEHHHQHQHKSHRSKWREEVEVIRRKVPEDLELLARREKLLAAERESRQRKQTAREELEA

RRELLRERNEHSDALSPTTVAASVTAGLNIHVKRKSKPDNYEKEIKLKKRREDDIEVIRDDDDEESEESDSNEEVPEQDS

EGSATESGSEDSYASKKKSKIKSKSQLEDDDEDLPLPDSPLSVGELYKSPKQRQRSRSVSSKSSSQSSRSSRSRSRSRSQ

SSLEDEVDRQDVGADASPSSSTRSEERGMTQEQPEEKPEEKLKEKQKSLEEQIPCDDKGIPLPNYYPGVQGCRSVEEFQC

LNRIEEGTYGVVYRAKDKRTNEIVALKRLKMEKEKEGFPITSLREINTLLKGQHPNIVTVREIVVGSNMDKIFIVMDYVE

HDLKSLMETMKNRKQSFFPGEVKCLTQQLLRAVAHLHDNWILHRDLKTSNLLLSHKGILKVGDFGLAREYGSPIKKYTSL

VVTLWYRAPELLLCSPVYSTPIDVWSVGCIFAEFLQMLPLFPGKSEIDELNRIFKELGTPNEKIWPGYTELPAVKNMLSQ

NSQFTEYPVSQLRKHFQEKTSEMGLSLLQGLLTYDPKQRLSADAALKHGFFKELPLPIDPSMFPTWPAKSELGARKAQAS

SPKPPSGGSQFKQLGRDEPIIVGPGNKLSSGIITGNKKSHGAGGSSASTGFVLNAGITQRQLAMGPGFSLKF

>Dme-cdc2rk-gi|17647247| ref|NP_523674.1| cdc2-related-kinase [Drosophila melanogaster] cdc2rk

MSSLKSNDVDTNPAPDPQAPITRKGFLMSLNTGTPMPIPEQNLFGRCRPVSEFEKLNRVGEGSYGIVYRARDTRSNEIVA

LKKVRMDQEKDGLPISGLREIMILKQCHHENIVRLREVVVGKSLDSIFLVMDFCEQDLASVLDNMSQPFTESEVKCITLQ

VLKALKYLHSRFMIHRDLKVSNLLMTDKGCIKVADFGLARMFSNPPKPMTPQMVTLWYRAPELLLGCRTHTTAVDMWAFG

CILGELLLGKPLLPGNSEIAQLDMIIDLLGAPSESIWPGFADLPAVQNFTLSQQPYNNLTPKFHMIGQSGRNLLNILFIY

NPKTRATAEECLKSKYFVDPPQACDPGMMPTFPQHRNNAAPAPAVQPPADIPISDLLNVFIKRQRME

>Dme-Eip63e-gi|24656896| ref|NP_728889.1| Ecdysone-induced protein 63E, isoform A [Drosophila melanogaster] Eip63E

MAELISQPTKINNNNFGAGVTMREKKGGALQKLKKRLSHSFGRLTISREDGDESTHHHHHHHHHRGGHGGHHNHGNKVPY

NGYNSEEYLDRLEPNGNIPADKTNCRYGDWRSTDSTDHHDRVQRQLSVSSDSKLLDEDIREEMKYHSHVVMRPKKPPRPK

SEVFLNKQETHPRRKRFSAFGGDSPFGKQEAYVKLEPLGEGSYATVYKGFSKLTYQRVALKEIRLQEEEGAPFTAIREAS

LLKELKHSNIVTLHDIVHTRETLTFVFEYVNTDLSQYMEKHPGGLDHRNVRLFLFQLLRGLSYCHKRRVLHRDVKPQNLL

ISDCGELKLADFGLARAKSVPSHTYSHEVVTLWYRPPDVLLGSTEYSTSLDMWGVGCIFVEMVTGMPTFPGIRDTYDQLD

KIFKLLGTPTEDTWPGVTHFPGYKPHKLGFYRPRKLGHNFPRLYDIIEGETIANGFLQLNPEQRLGADDALQHPYFAQLP

KKLYELPDETSIFTVEGVQLYTEPNRQNK

>Dme-cdk9-gi|24658274| ref|NP_477226.2| Cyclin-dependent kinase 9 [Drosophila melanogaster] Gene ID: 37586 Cdk9

MAHMSHMLQQPSGSTPSNVGSSSSRTMSLMEKQKYIEDYDFPYCDESNKYEKVAKIGQGTFGEVFKAREKKGNKKFVAMK

KVLMDNEKEGFPITALREIRILQLLKHENVVNLIEICRTKATATNGYRSTFYLVFDFCEHDLAGLLSNMNVKFSLGEIKK

VMQQLLNGLYYIHSNKILHRDMKAANVLITKHGILKLADFGLARAFSIPKNESKNRYTNRVVTLWYRPPELLLGDRNYGP

PVDMWGAGCIMAEMWTRSPIMQGNTEQQQLTFISQLCGSFTPDVWPGVEELELYKSIELPKNQKRRVKERLRPYVKDQTG

CDLLDKLLTLDPKKRIDADTALNHDFFWTDPMPSDLSKMLSQHLQSMFEYLAQPRRSNQMRNYHQQLTTMNQKPQDNSMI

DRVW

>Dme-cg7597-gi|24668137| ref|NP_649325.2| CG7597, isoform A [Drosophila melanogaster] CG7597

MHASSAAATALVEYSDVSSEDFSDQEAGDLDADAGKGAGNIKKPKPAPDNQFSKGRLDAKPDKEGYDNYRSRRAEDSSDP

VAAGSRQTSSSEATNPREEPSQASNTSKDELWGREIYMSSDSIDTDELEAEMKRQKRKKQKKEKHKHKSKKKSKKRKKKR

AKSYSSIDSMSDNDINALLDRRYTPPTAPSKSNERTVSAAPSSFTPHNLKESSSPATPPPVRRPNTNSNYYGESSLETAN

SALGSNLQVTVTNKQSISNRLRSPPPSSRSSGNGPRFGNSPRTPPPSHYSSSGGGGVGSGSVVRDSRSSRYVNSPHKEDV

SAHHRSSHDHGYQGRYSGAGSSSHDTRKVKRLSPELDRYNHQPSTPPHKRRKFSDGREVGLGNFEHSRHHSGKYERYSRD

RYSRRSSRSPSVQHSRSRQSPSGGLSSGSNAFRHGGSHKHKYGTTVSSTPSHTTRTSKRASGTGTSGDRYSRSPRTSSRY

MESSPPSPVGASGSHHYHHRRSPRMRQRTRGDSRRRSPSSASSESSASRSRSPTSRDLKHKREEYIKKISETSLFAELVK

DRHKRQKALKEIIERQEENSNSNSNGALTINDNSSSVDGNTPNAADGRSAPGSGTPAAASTTSNGLQALGSKPDLDLNNI

PMPNKQNDSVVSNPASNADVPDSVAQLKQPLLVPPFSASKNNIKPKSLTSLPLPPGMNVLDLAGARSPSPGQKKESDEKN

VTSSGSANKSVLNLPMPPVIPGSEELSGDDDVIDSPEDFDAPAVGTVHGHGGGPGTTRQRPVILNRRDSRNNVRDWGERC

VDVFEMIAQIGEGTYGQVYKARDHHTNDMVALKKVRLEHEKEGFPITAVREIKILRQLNHRNIVNLHEIVTDKQDAVEFR

KDKGSFYLVFEYMDHDLMGLLESGMVDFNEENNASIMKQLLDGLNYCHKKNFLHRDIKCSNILMNNRGKVKLADFGLARL

YNADDRERPYTNKVITLWYRPPELLLGEERYGPSIDVWSCGCILGELFVKRPLFQANAEMAQLETISKICGSPVPAVWPN

VIKLPLFHTLKQKKTHRRRLREDFEFMPAPALDLLDKMLDLDPDKRITAEDALRSPWLRKINPDEMPTPQLPTWQDCHEL

WSKKRRRQMREQQESLPPTVIASTKYQQHGATMVGDA

>Dme-cdk8-gi|24661985| ref|NP_536735.2| Cyclin-dependent kinase 8 [Drosophila melanogaster] Cdk8

MDYDFKMKTQIERTKVEDLFNYEGCKVGRGTYGHVYKAKWKETSDGKEYALKQIDGTGLSMSACREIALLRELKHQNVIT

LIRVFLSHNDRKVFLLIDYAEHDLWHIIKFHRAAKATKKQVVVPRGMVKSLLYQILDGIHYLHSNWVLHRDLKPANILVM

GDGNERGRVKIADMGFARLFNAPLKPLADLDPVVVTFWYRAPELLLGARHYTKAIDIWAIGCIFAELLTSEPIFHCRQED

IKTSNPYHHDQLDRIFNVMGFPQDKDWEDIKKMPEHHTLTKDFKRSTYSTCSLAKYMERHKIKPDSKAFHLLQKLLLMDP

NKRITSEQAMQDQYFQEEPQPTQDVFAGCPIPYPKREFLTDDDQEDKSDNKRQQQQQQQQQQQQQQQQQQQQQQQQQQQQ

MNAEPNAKRVRLSGAGNQQDFHHQQQQQQQQQQQQQQQQQQMMFNQQQNFQRFN

>Dme-cdk4-gi|17137264| ref|NP_477196.1| Cyclin-dependent kinase 4, isoform B [Drosophila melanogaster] Cdk4

MSYVRQLKRQKMSQAKKFGDGDPFNYQELNIIGEGAYGTVYRARDVITGNIVALKKVRISLNENGVPMSTLREISLLKQL

NASNHANIVKLYEVCQFLERDGQLLILLVFEHVEQDLSDLIDRLPKSGMSPPTIQRLSRELLTGVDFLHSHRIIHRDLKP

QNLLVSSQGHLKIADFGLAKTYGSEMKLTSVVVTLWYRAPEVLLAQPYNSTVDIWSAACIIFEMFNRRALFPGTSEKNQL

DRIFELTGRPTEQQWPQTISVALEHFPQRHPKRPKDFCPHLCKYADDLLNKMLSYDLHLRPSALACLEHDYFQQEPL

>Spu-gi|115964384| ref|XP_001204469.1| LOC767535 hypothetical protein LOC767535 [ Strongylocentrotus purpuratus ] Gene ID: 767535 LOC767535

MNQYSILDRIGEGAHGIVFKAKHIETGEIVALKKVPLRKLDDGIPNTALREIKALQEIEENQYVVKLKDVFPHGTGFVLV

FEFMLSDLSEVIRNSDQPLTEAQVKSYMLMLLKGITHCHENSIMHRDLKPANLLISETGHLKIADFGLARVFSNDEGRQY

SHQVATRWYRAPELLYGARKYDEGADLWAVGCIFGELLNNSPIFPGENDIEQLCCVLRILGTPTEKTWPGMKDLPDYNKI

TFPENPPIPLEQIVPDASPEALDLLKKFLVYPSRQRISASEALLHPYFFTEPLPAHHSELPIPQRSRRGRRSQHAKEYDI

DVPLEQTLIDPQEIAPFV

>Spu-gi|115702469| ref|XP_790847.2| LOC585950 similar to cyclin dependent kinase 2 [ Strongylocentrotus purpuratus ] Gene ID: 585950 LOC585950

MNNFEKIEKIGEGTYGVVYKARDLKSGKTVALKKIRLDTESEGVPSTAIREIALLKELDHKNIVKLHDVVHSDKKLYLVF

EFMNQDLKKYMDVAPPSGLPPGLVKSYLHQLLQGIAFCHAHRVLHRDLKPQNLLIDADGRIKLADFGLARAFGVPVRTYT

HEVVTLWYRAPEILLGCRYYSTAVDIWSLGCIFVEMITRRALFPGDSEIDQLFRIFRTMGTPDEKLWPGVTSLPDYKTSF

PRWTPQDFTKIVPMLNKDGKDLLKSMLCYEPDKRISAKTGLSHPYFKDVKMVPPPRLPT

>Spu-gi|72012799| ref|XP_781415.1| LOC575965 similar to p34cdc2 [ Strongylocentrotus purpuratus ] Gene ID: 575965 LOC575965

MEDFMKIEKLGEGTYGVVYKGKHKRTGKIVALKKIRLESEEEGVPSTAIREISLLKELYHPNIVMLEDVLMEPNRLYLVF

EYLTMDLKKYMESLKGKQMDPALVKSYLHQMVDGILFCHSRRILHRDLKPQNLLIDNNGTIKLADFGLARAFGIPVRVYT

HEVVTLWYRAPEVLLGSTRYACPIDMWSLGCIFAEMVTKRPLFHGDSEIDQLFRIFRTLGTPTDDIWPGVTQLQDYKSTF

PMWTKPNIKGAVKGMDEGGLDLLEQMLIYDPAKRITAKASMRHPYFDNILPLSDRLQPIRS

>Spu-gi|72158568| ref|XP_797002.1| LOC592381 similar to cyclin-dependent kinase (CDC2-like) 10 [ Strongylocentrotus purpuratus ] Gene ID: 592381 LOC592381

MSTGDGQLSSQLRRQSSENSGSPPGLSEKVTHLTSIATGQVMKIPEEDRLGLCRSVSEFEKLNRVGEGTYGIVYRARDMK

SKEIVALKKVRMEKEKDGLPISGLREIHLLINLRHENVVELHEVVVGQHLDSIFLVMQYCEQDLASLLDNMPSPFTETQV

KCLALQMLRGLRYLHDNFVIHRDLKVSNLLLADNGCLKIADFGLARRYGLPVRPMTPRVVTLWYRAPELLFGSLEQTTAI

DMWAAGCILGELLVNKPLMPGASELHQINHIIDLLGTPNDTIWPGFSELPMVQNFTLKKQPYNNLKAKFTWLSQSGLRLL

NFLLMYNPKKRATAEESLESSYFKEQPLPCDKALMPTFPHHRNKRKSNTRPEHRPAMYGKNEFGVAFGSGDSKKSKR

>Spu-gi|115954076| ref|XP_001192015.1| LOC585189 similar to cell division cycle 2-like 1 (PITSLRE proteins) isoform 1 [ Strongylocentrotus purpuratus ] Gene ID: 585189 LOC585189

VDDDEDDALNIQPPQAKPSSLKDTRHGKDEKRKHARSHSRDKDRKKTKRSRSAEKGKPSRSKDSDRDRDRDRDRGRERRR

DRDRDDNSRDKDKKSRKEPQEDSRAAPSGGHSHHEDVRPSGSSSQSHQPDLRQMIVDRRDDTRQVTVVGDNRAQQKETER

KPREGKKESEREKERREDRHEERPRDREKKEKSRDSSTKHEGSKEKSRSRRDELIEERREGSRPSSKERTSGGSNRSDDR

DSRSKRSDQSSSHRDRSPHRSSRDDKRKRDERRKEERESEHDSDQRRSKSSSKASKSNERERWKEDSEERQRREERKMEE

EEEEEEKEEEYERKEIEELEEEHEVEEENASSSEEEEESDDEEEDDEEEESSEAESESEEEEEVEEIKEASSSEEEGSAE

EGEASAGDLEEGEHKDSDDEEEEEEEEEEEEEEEEEEEESEEEESDSDSESQSEEESEKSVSEVDHEEMQQEEEKAKVKQ

EKETDSSKIYVHAKSKFDDDSPSPIRELPGLVHEEEEIEDEVEDQSDTEYLQDSPVASPVELEETLPPYLPAIQGCRSVE

EFQCLNRIAEGTYGVVYRANEKKKNDIVALKRLKMEKEKEGFPITSLREISTLLKAQHRNIVTVREIVVGSNMDKIYIVM

DYVEHDLKSLMETMKQPFTIGETKCLILQLLRGVHHLHDNWILHRDLKTSNLLLNHQGCLKIGDFGLAREYGSPIKPYTS

IVVTLWYRAPELLLGTKVYSTPIDMWSVGCIFAEFLTIKPIFNGRSEIDQLNRIFKELGTPSEKIWPGYNELPAVKKTTF

AHHPYNNLRNRFGTYLTDVGFELLNRFLTYDPVRRISAEDALKHPYFSESPQPISENMFPTWPAKSEMVRTNKPRSPQAP

EGGQQFAKLEKEEEDSGFRMALPTQGSSAASAGFSLKF

>Spu-gi|72004784| ref|XP_782022.1| LOC576645 similar to ENSANGP00000015862 [ Strongylocentrotus purpuratus ] Gene ID: 576645 LOC576645

MPILVPTILTMPKSSTRRARFIKPLPVMVTGPTCNHKRFGKSPYCKSCSPFGKVETYKKICPLGEGSYAKVYKAVSCINQ

QVVALKEIRLQQDEGTPFTAIREASLLKDLKHANIVCLHDIIHTKTTLSFVFEYVHTDLSTYLERHPGGLNPNNVRLFLF

QLIRGLSFCHKRKILHRDLKPQNLLISEAGELKLADFGLARAKSIPSRTYSHEVVTLWYRPPDVLMGSTDYSTQLDIWGV

GCIFLEMMWGQPAFPGLKDATDQLEKIFKVLGTPTEQTWHGVSRLPNYGKVAAAGRFHKPKLLCEILPSILLIPGAESLM

TDMLQLEPRKRISTVQAMTCQYFKDLPPKIFDLPPGASVFNVPGMKLEPEKNNLSK

>Spu-gi|72087356| ref|XP_789337.1| LOC584384 similar to CDC2L5 protein kinase [ Strongylocentrotus purpuratus ] Gene ID: 584384 LOC584384

MPGNHKNRGGSPTYYHEHKDSPSKRKHAKSSKSGRHKSKRSRDKVKEQVVKPLVEYDDVSSEDYSLSPPPSPTPPPKRRA

SPQPQKKRRHVSPATAIKIYKTQQGKESPHVSNSKNSSSRRQPSPETYSVSQKSHSSSRKGRASEASRPRAYQSSPPPPT

RYHTSPPRYSASPKRSKQSKSKQKYRKSPTSSRKNRSPSPDYYQTSRQSNKSRSRSPSYSKSKTKSRKRHQRISRSPSGS

PYRRSKHSPSRSPSPYRGKKRARSPTPNFPATKFHSSIGGEVQKYRKKTVQAAKAKVVKQEKEPSPEPVPEPVKVKQVKQ

PKPESPMVFTIKNDLKETASPQRTVVELPDRGLIIKKELKPVVPSPVPAPAPVRTTPPPKQTSARPPLPLPPMIDAVDSP

VIAQKGKGDVAERMRNLSRDRARSRTSITDLPLPPMAPTPDESPVDTPSPQTQLHLRAKEKDTKDPKVDLGKREMIRQRR

AKMAGSSLDWGERCVDVFEIINQIGEGTYGQVYKARDKDTGELVALKKVRTDNEKEGFPITAVREIKILRQLNHDSVIRL

HEIVTDKQDALDFKKDKGAFYLVFEYMDHDLMGLLESGLVNFSEEHVRSFMKQLLDGLNYCHKRNFLHRDIKCSNILLNN

KGHIKLADFGLARLYHADDKTRPYTNKVITLWYRPPELLLGEERYGPAVDVWSCGCILGELFTQRPIFQANQELAQLELI

SRICGTPTPAVWPDVIRLPLFNTMKPKKMYNRRLREEFSLLPKDALDLLDGMLTLDPDKRTTAEDALNCGWLQTSGTGKL

SQPDLPHWQDCHELWSKRRRRQERQQQQQLAAQNKAGNGPDPSHSKSSRKEPPPPAPAVTEEEAPQIPGLGSEEPSSSKK

GSKAASRSNTPQAPREGKDSSSDGGKATGEKKLAKLINLLQAQPQLNVNKLAETLNVQVDASTIKLLENLNMQLLIAAHA

TKQKSKEGGDDAKPLPLPTKEVTTDLLVKALEGSKGSGGALEQVFGPSSSEQKKHAKEIATSLAQYVSALFGKPVNPSKG

PTDPAPSSVVSQGKSHGNAHQSTSIVTPPSDSRSSYIDNLSGSQGGESSSVPFSRKNSKPDFGVSDDSQQSWKPPTPLGP

PPESPEAEEQEQQQEEDTSLVTPGVKAALMQMIAQQGLSELGIDNQPVDPNLPQPPEPSLPPPDNAPQDPFLPQSKAIFP

SGQYCKEDPSKASGSGDRANSVGAGKSKGKNWSSPFNRGGGRGGFKGSHRGNKKGPVGPGTGWS

>Spu-gi|115682385| ref|XP_001200386.1| LOC581028 similar to cyclin-dependent protein kinase 5 [ Strongylocentrotus purpuratus ] Gene ID: 581028 LOC581028

MASHYCMRQRSTEAKEYYTTAERTYGTVFKAKNRETQEIVALKRVRLDDDDEVITVVRKIVTQSECNFFSLPSLHENSDD

MYNKVNEYCDEMFIYSLCRVNVIVESFMYQLLRGLAFCHSHHVLHRDLKPQNLLINKNGELKLADFGLARAFGIPVRCYS

AEVVTLWYRPPDVLFGAKVYTTSIDMWSAGCIFAEMANAGRPLFPGNDVEDQLKRIFKLLGTPTEDTWPGISKLPDFKPY

PIYPVTTPLASVVPSLSATGRDLLQRLMMCNPALRMSAEEGLMHQYFADLNSVVN

>Spu-gi|72136390| ref|XP_798269.1| LOC593706 hypothetical LOC593706 [ Strongylocentrotus purpuratus ] Gene ID: 593706 LOC593706

MAESKGASFSGTSGPPVQSSANQGYKMAMAPYQRESPEFKYCEEVSKYDKLAKIGQGTFGEVFKAKHKKNKNIVALKKVL

MENEKEGFPITALREIRILQLLRHENVVPLYEICRTKATAYNRYKGSIYLVFEFCEHDLAGLLSNTNVKFSLGEIKSVIK

QLLNGLYYIHSNKVLHRDMKAANILITKAGVLKLADFGLARAFSLPKGDAPNRYTNRVVTLWYRPPELLLGERNYGPAID

LWGAGCIMAEMWTRSPIMQGNTEQHQLTLISHLCGSITSAVWPGLEKLELSNTLELPKGHKRKVKDRLRSYVKDQQALDL

IDKLLNIDPKRRMDAAIALDHDFFWSDPMPCSLERMLSTHTQSMFEYLTPRRRQQHPAAAAAANHRNPHHANPPNRPSTA

APDQNFDRVF

>Spu-gi|47551029| ref|NP_999689.1| cdk4 cyclin-dependent kinase 4 [ Strongylocentrotus purpuratus ] Gene ID: 373289 cdk4

MASSVPSRSTPERYVQAAEIGSGAYGIVYKARDTETGHFVALKSVRIPIGEEGMPVSTIREISLLRHLCQLDHPNIVKLL

DVCDVMDVGRSEMMLTLVFELVDQDLAQYLEKCPPPGLSSCTIKFLMHQLLSGVEYLHSHRVTHRDLKPQNILVASDKKL

KLTDFGLSRVYSFQMALTPVVVTMWYRAPEVLLQASYATPVDMWSVGCIFAELYRRRPLFRGQSDKDQLHKIFEVIGLPP

EDQWPDVALPWSSFRQTGQRSFLDLVQEICNQGLDLLERMLCFNPDHRMTAEQGLLHGFFGDEEEDDEEDDDTEVEDDDD

EEDEDDEGVDVGQERSQSASTSSMSQATDDSGSHSQFFSDSSQSQDVTPTNKR

>Spu-gi|115722990| ref|XP_792916.2| LOC588123 similar to Cdk-8-prov protein [ Strongylocentrotus purpuratus ] Gene ID: 588123

MDYEFKQQTSGVRESVEDLFDFEGCKVGRGTYGHVYKATTKKEPKKEYALKQIEGTGISMSACREVALLRELRHPNVICL

RRVFLSHSDRKVYLLCDFSEHDLWHIIKYHRANKASKKGPTSVPPVMVKSLLYQILDGIHYLHSNWVLHRDLKPANILVM

GEGSERGRVKIADMGFARLFNSPLKPLADLDPVVVTFWYRAPELLLGARHYTKAIDIWAIGCIFAELLTSEPIFHCRQED

IKTSNPYHHDQLDRIFNVMGFPQGRIHASTIK

>Nve-gi|156349526| ref|XP_001622095.1| predicted protein [Nematostella vectensis]

MSTKDEIIQLASLKGGEKKSIPERYMFGSCRPVAEFEKLNRIGEGTYGIVYRAKDTKSGKIVALKKVRMEQERDGIPISG

LREITLLLNLRHENIVQLLEVVVGKHLDSLFLSMEYCEQDIASLLDNMSCPFSEAQIKCLMIQLLEGTKYLHEHFIVHRD

LKVSNLLLTGKGVLKIADFGLARTFGYPYKPMTPVVVTLWYRSPELLLGAKVHTTAVDMWAVGCIFGELLGNKPLLAGKS

EINQLQLIVDLLGTPNDHIWPGYSSLPGVKSISLKHQPYNNLKHKFSWVSQAGLSLLNYMLMYDPCKRATAAESLQSSYF

VEKPLPVDADMMPTFPEHRNFKNRSPTEGVEKKDKAHSRGRISRDLFGPDLQRTAKKRKV

>Nve-gi|156383566| ref|XP_001632904.1| predicted protein [Nematostella vectensis]

ESDDNSSGSDDESEESSEEEENQESAVNKQETATQDESSDSEESSEGNSEEDESETSEEERGEETTPDPEIQKQNEVESL

VLTELERKQFNTEVVDVESEESSDTDSSESAEEPEHDDSAQEEKVEPEEEKPGLPDYLPAIQGCRNVEEFQWLNRIEEGT

YGVVYRAKEKASGEVVALKRLKMEKEKEGFPITSLREINTLLKAQHPNIVHVREIVVGSNMDRIYIVMDYVEHDLKTLME

HMTSPFTVGEVKTLLIQLLRATAHLHDNWILHRDLKTSNLLLNNRGILKVVGDFGLAREYGSPLRHYTPIVVTLWYRAPE

LLLGIKEYSCPIDMWSVGCIFAELLTMEPLFPGRSEIDQINRIFKELGTPSDKIWPGPPAYSELPHVKKMTFTEYPYNQL

RNRFGTYLTDKGFSLLNRFLTYDPKKRITAETALKEDYFLEAPKPIDPSLFPTWPAKSEMQKMPRRKDHGHSPKPPEGGE

MFSKLKDEGDSDGSGGFHMLTSKKGTAAVPGFSIKF

>Nve-gi|156398520| ref|XP_001638236.1| predicted protein [Nematostella vectensis]

MFDIIEQIGEGTYGQVYKAKDKITGELVGLKKVRTDNEKEGFPITAVREIKILCQLNHPNIINLKEIVTDKPNALDFRKD

KGAFYLVFEYMDHDLMGLLESGLVHLTEDHIKSFIRQLLDGLNYCHKKNFLHRDIKCSNILLNNKGEIKLADFGLARLYE

ADERRPYTNKVITLWYRPPELLLGEERYGPGIDIWSVGCILAELFTKKPIFPAYQEIGQLELISRVCGTPTPAVWPSIIN

LPHFHSIKPKRQYRRRIREEFNFLPEDALDLFDAMLTLDPSQRITAEKALEHPFLTDPFVPQCK

>Nve-gi|156365870| ref|XP_001626865.1| predicted protein [Nematostella vectensis]

MSKDTLDFPYCHEVSKYERQAKIGQGTFGEVFKAKNRKNPKEIVALKKVLMDNEKEGFPITALREIKILQLLNHENVVKL

LEICRTKAQPFNRNKASIYLVFEFCEHDLAGLLNNQAVKFSPPEMKKIMQMLLNALYFIHSNKILHRDMKAANILITKNG

VLKLAEFGLARAIHINKEQKQRYTNRVVTLWYRPPELLLGERNYGPPIDLWGAGCIMAELWTRTPIMQGNTEQHQLTLIS

HLCGSITPEVWPGVDKLELFDKMVLPSGQKRRVKERLRMYVKDHNALDLIDKMLSLDPGPRIDADSALNHDYFWTDPMPT

DLTRTLAMHNQSMFEYLAPPRRGGQRQPAPPRPGPTQPSIVQSGTYDRVF

>Nve-gi|156360882| ref|XP_001625252.1| predicted protein [Nematostella vectensis]

MAAKVERSKRYKKIDFLGEGQFATVFKAEDQETGNIVAVKKIKLGNRAEAKDGINRTALREIKLLQELSHENVIGLLDVF

GHKSSISLVFPFMETDLEVLIKDTCIVLSPADVKSFTIMTLKGLEYLHSHWILHRDMKPNNLLLDDKGVLKIGDFGLARA

FGSPSRVYTHQVVTRWYRPPELLFGARIYGTGVDMWAEGCILAELLLRVPFLPGATDLDQLSRTFQTLGTPTEETWPGMT

SLPDYVEFKPFPGIPLKDIFSAAGDDMLDLLDRLLDCNPNGRVNATQALNMPYFSNKPAPTPPHLLPRPKSSGKVTKKEE

EKSLKRKLESATDGAIG

>Nve-gi|156408568| ref|XP_001641928.1| predicted protein [Nematostella vectensis]

MEQYGQYTILGRIGEGAHGIVFKAKHIESGEIVALKKVPLRRLEDGIPNTALREIKSLQENEENPYVVKLIDVFPHGTGF

VLVFEYMWSDLSEVLRNSERPLTEAQIKGYLLMLLKGVAYCHNKGIMHRDLKPANLLISSTGHLKIADFGLARVFSNEGE

RQYSHQVATRWYRAPELLYGARKYDEGVDLWAVGCIFGELLNNSPLFPGENDIEQLCCVLKTLGTPNEEIWPGMTDLPDY

NKITFPDMPAIPLEKIVPDASPEAMDLLKRFLVYPSKKRIPASEALLHPYFFMEPLPAHHSELPIPSRNSRKASLRASHG

KRFDISTKLEKSLVNPARLFHHNLNFQ

>Nve-gi|156385093| ref|XP_001633466.1| predicted protein [Nematostella vectensis]

MENFEKVEKIGEGTYGVVYKARDKTTGRMVALKKIRLDTESEGVPSTAIREISLLKELNHPNVVSLLDVVHNQKSLYLVF

EFLSQDLKKYMDCLPPSGISTSLIKSYVYQLLSGVAYCHSHRVLHRDLKPQNLLIDKNGAIKLADFGLARAFGVPVRSYT

HEVVTLWYRAPEILLGSRYYATPVDVWSIGCIFAEMKTRRALFPGDSEIDQLFRIFRTLGTPDDKVWPGVSELPDYKTSF

PKWPVQSIRHVLPTLDNTAIDLLQKMLTYQPNARISAKAALSHEFFKDVQRKILIEN

>Nve-gi|156391793| ref|XP_001635734.1| predicted protein [Nematostella vectensis]

MDDFSKIEKIGEGTYGVVYKAKNLKTGGFAALKKIRLEVEDEGIPSTAVREISLLKELRHHPNVVELQHILHQEPKLYLV

FEYLTCDLKKHLDTTRGMLDKTLVKSYLYQITNAIYFCHARRILHRDLKPQNLLIDSKGLIKLADFGLGRAFGIPVRAYT

HEVVTLWYRAPEVLLGGQRYSCPIDVWSIGTIFAEMVTKRPLFHGDSEIDQLFRIFRILGTPTEETWKGVTSLPDYKPTF

PKWAGDGLKKAVPQLDSDGLDLLKKMLIYDPALRISAKTSLKHPYFLNDPKFDINSLPKTPEVDSVM

>Nve-gi|156407302| ref|XP_001641483.1| predicted protein [Nematostella vectensis]

MQKYDKLEKIGEGTYGTVFKGKNKETREILALKRVRLDDDDEGVPSSALREICLLKELKHNNIVRLYDVLHSEKKLTLVF

EFCDQDLKKYFDSCQGEVDASVVKSFMFQLLRGLAFCHSHNVLHRDLKPQNLLINKDGELKLADFGLARAFGIPVRCFSA

EVVTLWYRPPDVLMGAKLYSTSIDMWSAGCIFAEMANGGRPLFPGNDVDDQLRRIFKILGTPTEESWPNVSKLPDYKEFP

PQGPSVSLGMVVPKLSSTGRDLLQKLLVSNPAHRISAEDAMKHAYFADLSPTFRG

>Nve-gi|156392090| ref|XP_001635882.1| predicted protein [Nematostella vectensis]

MATATAVENGKSESDERDGSRADLIVNQTSEAFVKDDDDVFHEKDSKTEKEAKSQQSSPRSSSYSHTPPLKRPPVRRTVS

DTIGGRLRKISGILGDSRRLSDEFVKEDLPIPPLSPSYGRLESYQKLEPLGEGSYATVFKGLCTANKKIVALKQIRLQEE

EGAPFTAIREASLLKQLKHGNIVKLHDIIHTKDTLMFVFEFLDTDLNCYLEKYSRGICPHNTQLFCFQLLRGLAYIHDRK

ILHRDIKPQNLLISERGELKLADFGLARAKSVPSQTYSHEVVTLWYRPPDVLLGSKNYTTSLDIWGAGCIFVEMLTGIAL

FPGLNGHIDQLNKIWQVLGTPTDETWPGVSKLPEYDPDIFINFRPRRIGQCIPRLVPIEGAEQLVIRMIQLDPKNRISAR

EAMRSTYFSNLPKEIYTIRDEDTIYSVSGVEIQKEPA

>Nve-gi|156403969| ref|XP_001640180.1| predicted protein [Nematostella vectensis]

SELGYGKMETYTKLDKLGEGTYATVFKGKSKLTDNIVALKEIRLEHEEGAPCTAIREVSLLKGLKHANIVTLHDTVHTQK

SLTLVFEYLEKDLKQYMDDCGGIMSMNNVRIFLFQLLRGLDYCHKRKVLHRDLKPQNLLINDKGELKLADFGLARAKSVP

TKTYSNEVVTLWYRPPDVLLGSTAYSTQIDMWGVGCIFFEMATGRPLFPGSTVEDELLLIFKVLGTPSEEVWPGISANEA

FIAGKFPDYPRENLIIHAPRLDNSGLELLEKFLEYTVKDRVSAHDAMRHDYFYSLGTRCLNLGDLDSLFSLEEIRLTKDP

GYR

>Nve-gi|156374066| ref|XP_001629630.1| predicted protein [Nematostella vectensis]

MATKYEEVAEIGTGAYGTVYKAKDLLNDGKFVALKRVRIQNSEEGMPLSTIREIALLKQIDNFAHPNVVRLLDIFHIPML

TARETHLNLVFEHVDQDLAAYLEYCPQPGLGEWKIKDLTYQILNGVDFLHTHRIVHRDIKPQNILVTKDGQVKIADFGLA

RVYKDAMALTSVVVTLWYRAPEVLLQSSYATSVDIWSVACILAELFNRRPLFEGKNDVDQLDKIFSIIGSPSQDEWPQNV

SLPWTSFSRYTTGSFQALVPEMCTEGTTLLKEMLQFLPRSRPSASEAMNHPFFKELHDNNKENSNTVSNNINEKNLTVC

>Aqu-gi|340373367| ref|XP_003385213.1| PREDICTED: cyclin-dependent kinase 7-like [Amphimedon queenslandica]

MSKRYRKLDILGEGQFATVYRAEDTERDNAIVAVKKIKIGQRREARDGINRTALREIKLLQELHHPHILGLLDVLGHKSN

ISLIFDFMDTDLEMIIKDKSLLLPPGVVKAYSLMILQGLEYLHAHWILHRDLKPNNLLINKDGVLKITDFGLAKAYGSPN

RIMTHQVVTRWYRSPELLFGARLYSTGVDLWAVGCIIAEMLLRLPFLPGETDLGQLSTIFEFFGSPNEENWPSVKSLPDY

VEFKPSPPQSFRDVFSAAGDDLIHLLESCMKLDPSKRCTASQALQSSYFSNPPGPTPGPRLPMPRSRKEIEADIEHEIMA

SRKRALDDSQEGQKVAKRLNF

>Aqu-gi|340381980| ref|XP_003389499.1| PREDICTED: cyclin-dependent kinase 20-like [Amphimedon queenslandica]

MENYKILGRIGEGAHGVVLKARHTQTGDLVALKRVHLKKPADGIPNSALREIKALQESGENHHVICLRDMFPHGPGFVLV

FDYMLSDLAEVIRNAEKPLTEAQVKSYMTMLLKGVAYLHDNKIMHRDLKPANLLISQTGHLKIADFGLARVLSTELGRLY

SHQVATRWYRAPELLYGARQYDTGIDMWAVGCIFGELLNTSPLFPGENDIDQLCCVLRILGTPSERIWPGMSQLPDYHKI

SFSEMSPTPMEVVVPDALPEAVDLLKSFLVYDSRHRLSAAKALLHSYFFTPPLPAHHSELPIPSKAPPGRKTFDVFAPLQ

DSLVDTTALQTILS

>Aqu-gi|340371337| ref|XP_003384202.1| PREDICTED: cyclin-dependent kinase 10-like [Amphimedon queenslandica]

MEAKEGKKSLSKGTIVSPSTLKPVPFACKDVLGSCRSVDEFNKLNRIGEGTYGVVYRAEDKKSKEIVALKRIRMENEEEG

LPICSVREIGLLLSLSHENIVQLKEIAVGRELDNMFLVMNYCEQDLASLIDNMASPFTEPQVKCIMLQLLEGLSYLHNNH

VIHRDLKVSNLLLTDKGILKIADFGLARTLGRPLKPLTPTVVTLWYRAPELLFGSREYSCSLDMWSVGCIFGELLLNKPL

LPGKSEANQIELITNLIGSPNEGIWPGYSKLPLVASLEIKRQPYNNLKEKVYWISETGRGLLNDLLTYNPEYRMSSSRAL

RCKYFNENPLPVEPSMMPTYPHLRNTKPQVHRAIPVDTRPAKKQRIN

>Aqu-gi|340369066| ref|XP_003383070.1| PREDICTED: cyclin-dependent kinase 17-like [Amphimedon queenslandica]

MQRRASLSEIGFGKLQTYEKLEKLGEGTYATVYKGKSNITGKLVALKEIRLEHEEGAPCTAIREVSLLKDLKHANIVFLH

DIIHTARSLTLIFEYVEQDLKQYLDQCSGMMAMPNVKLFLFQLMRGLQYCHSRKILHRDLKPQNLLISEQGDLKLADFGL

ARAKSVPTKTYSNEVVTLWYRPPDVLLGSIDYADSIDMWGVGCIFYEMIVGRPMFPGANVEEELVLIWKSLGTPNEKTWP

GITKNKEFISHSFLRYDPQPLGLIVPRLDKEGINLMSKLLSYESQERLLARDGMKHNYFSSLPPQIHDLPHIVSIFTIPG

VELVKNPGDRKSQKDTKTSYDRDRRRSSLY

>Aqu-gi|340384408| ref|XP_003390704.1| PREDICTED: cyclin-dependent kinase 5-like [Amphimedon queenslandica]

MSTMWVKNTMEKYERLEKIGEGTYGTVFKAKEKESGEIVALKIVRLDEDDEGVPSAALREICLLKELKHKNIVRLTDVLH

KNLKLTMVFEYIDQDLKKYFDVSGGIISPQVVQSFFFQLLQGLAFCHYNNILHRDLKPQNILISKKGDLKLADFGLARAF

GIPVRLFSAEVVTLWYRPPDVLMGAQVYNTSIDMWSAGTIFAELANAGRPLFPGSDVDEQLKRIFKLVGTPTERSWPGLT

KLPEFKEFPPYPPACIESVVPALNDAGVDLLQRHLICHPTERISAEEAMRHEYFADIDPSPASWSSLQNTHNYLRSRTQW

SDYNKSQRIPLILSVLKKIKMTL

>Aqu-gi|340379293| ref|XP_003388161.1| PREDICTED: cyclin-dependent kinase 2-like [Amphimedon queenslandica]

MDGYERLEKIGEGTYGVVYKARQATHGNRVVALKKIRLDAECEGVPSTAIREISILKELDHVNIVSLLDVLYCDRKLFMV

FEFLDYDLKKYMDRHAPTGIPTDYLYQLLEGVAYCHAHRVLHRDLKPQNLLISSDGRIKLADFGLARAFGVPVRTYTHEV

VTLWYRSPELLLGSQYYSTPVDIWSIGCIFAEMVTKRPLFPGDSEIDQLFRIFRTLGTPDESVWPGISSFPDYKSSFPKW

PRQNLQRIVKSLDTLGINLLEQMLCYEPCKRITAINGMRHPFFSE

>Aqu-gi|340381019| ref|XP_003389019.1| PREDICTED: cyclin-dependent kinase 1-like [Amphimedon queenslandica]

MSVDDYTKLEKIGEGTYGVVYKARHKVTGKTVALKKIRLENEEEGVPSTAIREISILKEVQHTNVVKLEDIIHQDLKLYL

VFEFMCMDLKKYLDSLPAGKFMEPDLVKSYTYQILKGIVFCHGRRIIHRDLKPQNLLIDNNGGIKIADFGLGRAFGIPVR

AYTHEVVTLWYRAPEVLLGCPRYSCPLDIWSIGCIFAEMSNKKPFFQGDSEIDQLFRIFRILGTPSDAIWPSVTTMPNFK

STFPKWTGKSLSQLCPHLDSIGCDLLMQMVIYNPGKRISAKRAMEHPYFQGLRKSILPL

>Aqu-gi|340383363| ref|XP_003390187.1| PREDICTED: cyclin-dependent kinase 11B-like [Amphimedon queenslandica]

MAYNEERGRGDEEAFFSKDIPSKGSLKSTVSRSYRSRDKGSYGDSHQDDIDTYRREMKRQAPPTTDEGRQTERRHGDRRR

DNEGRHHGRSVEHKSKRKSRKETTNPKRERRTRSRSPIYSTQSRSHSDRREPRSQYSHSDSQSKSHSGAWSSSHRHTHSH

KSEYDRGEGGSGGRRDVYRLVRDLESSSSGSDSDEGVVSKEATPTRLKDGTEESKKESPKKTWKDIMYSDSEEEEEGESP

LRKSASPRESDQSDSRQGEDDVKDVSATPPLAESVKKEDEPAQPTAESAVEIKVSSATPPGGPVSPSPPPSPHEEVLYLP

ALMGCRSVECYEWLNRIEEGTYGVVFRARDIRTDEIVALKKLKMEKEREGFPITSLREISTLLKANHENIVNVREIVVGS

NMDKIFIVMDYVEHDLKSLMETMKQPFLEGEVKTLLIQLLKAVHHLHDNWIIHRDLKTSNLLLSHKGILKVADFGLAREY

GSPLKNYTPIVVTLWYRAPELLLGAKEYSTAIDVWSVGCIFAELLQHKPLFMGKSEIDELNLIFKELGVPNESIWPGFGE

LPVAKKVQFTQQPLNNLRKRFPMITKNGFVLLNKFFAYDPKRRVTAEDALKHEYFEESPLPVDPSMFPTWPAKSEMTKKS

VRPKSPTAPEGGAFFFNQKVLELLMN

>Aqu-gi|340377126| ref|XP_003387081.1| PREDICTED: cyclin-dependent kinase 9-like [Amphimedon queenslandica]

MAGPVRTVNLPYCPEVGKYEKLTKVGQGTFGEVFKAKDRKTGRLVALKKVCMENEKEGFPMTALREIRILQLLQHNNIVN

LVEICRSKATPYNRDKGSIYLVLDFCEHDLAGLLECKEIKFSLSEIKNIMQQLFNALAYIHGNNILHRDMKSCNILVTRK

GELKLADFGLARALNKGANQRYTNRVVTLWYRPPELFLGERNYGPPIDMWGAGCIMAEMWTRRPIMQGDTEQKQITLICQ

LCGSISPTEWAGVEKLEYYQKLELPQKENRKLKERLRHFVEDPYALDLIDKLLMLDPRKRIDADSTLEHDFFWKDPLPTD

LSKTLSTLRSSMFVMHTGSHKPLPGPRPTQSQPPNVVSGNVHDRVF

>Aqu-gi|340369314| ref|XP_003383193.1| PREDICTED: hypothetical protein LOC100638755 [Amphimedon queenslandica]

MAAKLLLECCRKEAVPSFPPSFFPTGSLSWSTDDRISLVCSDVIYVITLKGFLPQNTKSLVPISVAIIEAPPMSFPLSLN

LSLDISKCIYGEHTPSNEGHSPFNEGPLVDGVFGVLFDPLVSGCGSKGRFLSAKWSPIRSNWSNRCILAGLTNSSSVLVY

DETTRGQWDLVFDFTDIYYRVLVSVDFDVKLLPATSEQSPPPTTKTNISQSEYQRRRDFFVSVAMSWLPVFLSEGVVLLA

IAMRSGHLVLWSMAVPVDRLVPPSLLNVIHLPAGISSVTTVEWTIINKNYLLSVGSASGHVLLLEWVESAFPYEEPMDVK

WLIYSKGCYTFAVLIKYMNNELMTSDPYLLSSNHTHPVSGITSLHNNDVITCSTDGTITSRPLPNMEGVTVGASASDTVI

NGKEEWSPVSLGGKITAGSCFGMDSSKNDLYTIIHVKTVDKVSTQLLILPLILPEDVSLIWQRSSSWDLYESLRSNLSLI

YEEGETRIESLPLAMRRHAAALSHDQELMFQIDGELLSQHCRRYLSEFMKWTEDNEIQNGDKSIISIGNTAALLKRHAQN

EFDFSIAENCLSFVVNNLAQVEKCPICSEKMSSEATGRPHNDELVSPGELSPEDAPKKKKRSKTRKSSTHHHTSTSGGKT

SKQKRRKTSGEGEVGGVYKSPSDKHKKDSKRRKKRRISGGGHYIETGRRRLSSSDNDHAYPMYDMISPPGSPYGRRHHSP

SPRRGHTPIYKRGRSPYSPRNSHTPSHYSPGRRGYSPRSRDYSPRYSPRRSHTPRRGYSPRGHYSPRRGHSPSPRRGHTP

SRRGYSPVHKRRYSPVRRGHTPSFKRRGSPKATHRRSSPHYFTRRSRSPRRRSYTPRSPSPPPFSPARRVVHRSRSPLTT

PPLHRQHRSPVYRHVKKVNSKRSSIDHGEVHEDLPSKELSPNGDALVEKCTSKSGPKTPPMTTPSETTPSSPSKPPPPVD

ELPPLPAEAPPPPPPEEEKPPLPPIPLPPTLIGQSEPKLPPPPLINDLSHRPVKSKAIPTPPELAALQLSHRCVSSFEIL

SQIGEGTFGKVYKAKDLKTGEVIALKKVLIRTDSEREGFPITAVREIKILRQLRHENIVTLKEIISDTPQAASLKHDKSS

SFYLVFEYCAHDLMGLIDSGMVVFSESHIQSLMRQLMEALCYCHSKNFLHRDLKCSNILINNKGQLKLGDWGLARYYFAD

DHSRLYTNHVITLWYRPPELLLGAEHYGPAVDIWSCGCILGELFTKKPLFHGSIEMEQLDAISRVCGTPTPANWPEVIKL

PLFQTFKFKKLYRRRVKEEYSNIIPEVPLDLLDKLISIDPSKRISSEEALNHPFLINATKDSIPPPLLPSHQDCHEMWSK

KKKKKEGKVTEGPPAEKPNLHNPPKGGVASVSPSNHQSSSSSSYRNESTDHQYNSSCDRPDGVHQNVSRQHHSLGPPHSL

SVPLPPAPLSTTSILESLNSSLADGGSVPAPGSTDERLHLKQVIAKAQRSLIEEITQTLGKGRGGRGEVPMVASITGRPT

QSFDYGNQSNKGLEEKAAAQLYDKDKD

>Aqu-gi|340380476| ref|XP_003388748.1| PREDICTED: cyclin-dependent kinase 8-like [Amphimedon queenslandica]

MDSGFIDALSQKREKVEDLFEYEGKKIGRGTYGHVYKAKKRSGDDPKEYALKQIEGTGISMSACREIALLRELKHSNVIS

LQRVFLSHADRKVWLLFDYAEHDLWHIIKYHRAVKGGKKPQVQVPKTMVKSLMYQILSGIHYLHSNWVLHRDLKPANILV

MGDGPERGRVKIADMGFARLFNAPLKPLADLDPVVVTFWYRAPELLLGAKHYTKAIDIWAIGCIFAELLTFEPIFHCRQE

DVEASSPYHREQLERIFLVMGFPHDRDWDDMKHMPEYSTLMRDFGRQKANFANCALQKYMDKHNVKHDTSAFKLLTKFLV

MDPNKRITSDQAMLDTYFTEEPSHTSDVFSSMQIPYPKRKFIFDGEPSKEEKPDPHKVMSQSFLLLVLLMNL

>Mbr-gi|167536606| ref|XP_001749974.1| hypothetical protein [Monosiga brevicollis MX1]

MATTVKGSRPAGKKGSGAKKAGYEKVKYLGEGQFGTVYLERDSQTGQLYAIKRIRLGDKSMAKEGLNQSAFREIMFLREV

HHPNIIDLHDVFLKKGNLHLVLELASTDLEKLIRNKRLDFAPGDVKSLLLQTYQALDYLHARWILHRDLKPNNILITTGG

QVKLTDFGLACTFGSPSREMTTQVVTIFYRAPELLLGARHYGVGVDIWAMACIHMELELRTPILPGDGPFDQLDKIMAFL

SSFGHAFAQPNYRELSSSWEFKPRDPTPIRALLPAVSDAAIDLLEKQFTYDPLKRPTARETLMHPYFSEAPGPTPPDQLP

LALHSSYNSKLHKT

>Mbr-gi|167516210| ref|XP_001742446. MONBRDRAFT_14035 hypothetical protein [ Monosiga brevicollis MX1 ] Gene ID: 5887597 MONBRDRAFT_14035

MEEFRILGRIGEGAHGVVFKAKHIESGVVVALKRVGLKRIEAGIPVALLREIQALRHVQHPNVVRLHDAFAHSAACVLRG

IIHRDLKPSNLLISPQGQLKIADFGLARIWHQDRQDGRQYSHQVATRWYRAPELLFGARHYDLGVDLWAVGCIFAEMINS

APLFPGENDIDQLSCVLHTLGTPTPENWPEASTFPDYGKITFDETPGVPPEDLVRNTSPEGRTLFASFVPYSAKRRQSAR

SALRSLYFFEEPLPLHHSQLPSPLQREVGGQINDPAASHAWGAAYEPH

>Mbr-gi|167517533| ref|XP_001743107. Cdc2 hypothetical protein [ Monosiga brevicollis MX1 ] Gene ID: 5888387 Cdc2

MEKYLKIEKIGEGTYGTVYKAKVKATGNLVALKKIKLEAEEEGVPSTAIREISLLKELSHPNVVSLMEVIHSENKLYLVF

EFLDQDLKKHIDSQRNGLSMELIKSYMLQLLKGIDFCHARRILHRDLKPQNLLINREGFIKLADFGLARAFGIPIRAYTH

EVVTLWYRAPEILLGQRQYACPVDMWSIGCIFAEMVTRRPLFPGDSEIDELFRIFRVLGTPTEQTWPGVSQLPDYKDCFP

RWSGEGLASLIPGLDAMGLDLLQKMLRYEPSQRISARQALTHPWFDGCAM

>Mbr-gi|167522771| ref|XP_001745723. Cdk5 hypothetical protein [ Monosiga brevicollis MX1 ] Gene ID: 5890844 Cdk5

MNRYAKIDKLGEGTYGVVFKARDRHDGSIVALKRISLESAAEGVPSNAVREISLLKSLHHPNIVRLYDVLHSEHKLTMVF

EYCDQDLKKFLDSCRGTPEHHVIQSFMFQLLQGIRHCHEERVLHRDLKPQNLLINKRGQLKLADFGLARPYGVPVRSYSH

EVVTLWYRAPDVLLGATGYDTSIDMWSAGCILAEMANKGSPLFPGTSVQDQLDLIFRVLGTPTIESWPGLHELPNYSGPF

LPHVDGVGLEAEVSSLFPEGLDLLQQLLRYVPDERLSADRALRHRFFDDIPDHVLELCRVDA

>Mbr-gi|167522415| ref|XP_001745545. Cdk2 hypothetical protein [ Monosiga brevicollis MX1 ] Gene ID: 5890866 Cdk2

MSTLDGSRRQGRRSSGFMKTLSRTFSRSGGAPHDDVGDLPATKPTKTLSRRASTGHFHLPEGAIPPTAKPAFGQVATYKK

LHKLGEGTYATVFKGISHINGKIVALKEIRLEHEEGAPCTGIREVSLLKGLKHANIVTLHDVIHTKDNLIMVFEFLSKDL

KAYMDDCNSYIDLRNAKLFLFQLLRGVGFCHSRKVLHRDLKPQNLLINHAGELKLADFGLARAKSVPIKTYSNEVVTLWY

RPPDVLLGSVDYSGDIDMWGVGCIFGEMISGRPMFPGATNADQLELIFKTLGSPSESTWPGVMALPEAKSNELGAYSPQP

VNAILPRLDKQGGALLKSLLKLDPHGRVSAVKAMQHPYFNSLGPYIKQLTNDKSIFQAPQVKYHMEPALRMAERRRFETS

RRASLQF

>Mbr-gi|167516962| ref|XP_001742822. Cdk10 hypothetical protein [ Monosiga brevicollis MX1 ] Gene ID: 5887315 Cdk10

MAGSCRSITEFEKLNILGEGTYGVVYRARDSRTGHQVAVKQVKMNQERGGLPLSSLREITALQQLRHTNVLQLLHVAAGR

RLTSIFLIMEYCEHDLAALVDNMPAPFPEPAVKCLMQQLFAGLDAMHRECLIHRDLKLSNLLLTDHGILKVADFGLTRVI

EDPAHHMSPTVVTLWYRPPELVFGMKNYTRAVDIWSCGCIFAELLAHEPLFPAKTEVALLEMVIGLLGAPHESIWPAFRD

LPLAHRFHMPHQPYSNLKQRFGFLSSTGLDLMQDLLMYDPEKRLSAIAASVHPYFRTAPLPLDPEFMPTFPRHRNYNH

>Mbr-gi|167538010| ref|XP_001750671. MONBRDRAFT_30180 hypothetical protein [ Monosiga brevicollis MX1 ] Gene ID: 5895964 MONBRDRAFT_30180

MSARDLYARRDAGGSKRRVFARDREGGPLAKRITAAGGTGGASGAGAGSKAPGAGLSDAESKLRAQLLAKMSDPAPKRPR

SRPKKAAASATKPKPTAAPTARPPVKSKPAVPDTPPHDPSSETTPLAPADTPPGSPPTFTREMDKELERRTPDPRDEPLA

VPSPPGTPPAESSYPVAQRPPSPPRVQIVEEPYYPAERGCRHINKVYEHLNRIDEGTYGVVFRVRDRSSGHIRAVKRLKM

EKEKSGFPVTSLREINTMLKVRHENIVRVEEIVVGDTMDDIFIVMEFVEHDVKALLESIQKPLLQAEVKTLMLQLLSGVA

HLHDNWILHRDLKTSNLLLSHRGILKIADFGLAREYGDPLKEYTTLVVTLWYRAPELLLGAKSYSTAIDMWSVGCIFSEL

LTRKPLFDGNGEIDTMNKMFKMLGVPDETRWPGLNDLPVPRSMSLRGPSENRLPRSYGNILPSSGVNLLSDLLCYDPAQP

YLEQQKQSRFRDRQRQCSIMATTQPDLRKRRGSQPQLLPNNAGISYPSDDEATPTEDVATNTTTTFADKNALSTEKSGAN

VVLLKDQPKKKKWRDWFIRGMLSIVMISLFAAVVWLGPMALSIMIFLLQLKCFHEIISIGYYGWDSQLPLFRSLNWYFLM

AANYFFYGEHFNFLLQEFFAGAQARACTIPHSHMRFKAQKIIQYHRFISFWLYVIGFVSFVLSLRKGFYKYQFKMFGWTH

LSLLLVVATSQLIVRSIYEGLVWYLLPSCLVICNDFMAYMFGFFFGKTQLISISPKKTWEGFLGAFFSTMVFGVLFASTL

AEYPFFYCPVDSLTAYDIDVGVTETTFALKPIVFHALVLAIFASLIAPFGGFFASGLKRAFKIKDFDTLIPGHGGVTDRF

DCQFVMAVFFYVYYHSFIKVSARKDKPTPHASRDIADATC

>Mbr-gi|167516186| ref|XP_001742434. MONBRDRAFT_13866 hypothetical protein [ Monosiga brevicollis MX1 ] Gene ID: 5888029 MONBRDRAFT_13866

VGQFDLVNEVGQGTYGQVSKARIKNTDQLVALKMLKMEADRDGFPVTALREIKILRQLRHESIINLIGIVADVHSNRDLL

KRKPAAFYLVFEYMEHDLYGLLSSKQCTLDQEQIRYLMFQLMDGLRYCHAKHFIHRDIKGANLLVDNQCRLKIADFGLAR

LYEDRSRAYTNNVITLWYRPPELLYGAEVYGPEVDVWSAGCILGEFFLCRPMFRAGTEIEQLHAISMACGTPDPTNWPEA

QNLPAFKTLRPRKRYERNLAGFFRAEQLYVRRLGLLSPLMGYSPTSR

>Mbr-gi|167525607| ref|XP_001747138. MONBRDRAFT_32964 hypothetical protein [ Monosiga brevicollis MX1 ] Gene ID: 5892268 MONBRDRAFT_32964

MASEAPSAKRKKAFSFQFTRIDNATKFSPVNDGTGGEGTYGEVKRCRHVATQQIVALKKIKTEQEANGFPQTALREIQIL

KQLRHENIVQLQSVAVVSKECLYLPQHALPLLFTDKECPNLLPPEVKNTKSGKFCYMVLEFVDHDLNRILTAIKAKKPAG

SRKSPVSTTIFKGLALQMLRGLDHLEKNCVLHRDLKPANILLSAQGVIKLADFGLAKYHHPDVHNERKGHTALVCSQWYR

PPELLAGQVSYPEKIDSWSLAVVMGELFNLDPLFDGRTSSADGRTKAETYQFIQIQKLCGTDSFAWLEDCPLLPAKGHKK

RQVSQRFNTLIARDRSSEAVVAFMDKALTVDPNHRLLAGELLKWEHKGVSLFNGPTPTLMGPGLMSELNAIM

>Mbr-gi|167525918| ref|XP_001747293. MONBRDRAFT_33116 hypothetical protein [ Monosiga brevicollis MX1 ] Gene ID: 5892560 MONBRDRAFT_33116

MASKAFRQKMAQSVVDCSTIFANAGCVGQGTYGIVHKVYRLEGTRQLNRGCALRQEGDDLDDTTKPYALKGMVTSVSRTE

GFSTANLRELALLRELHHDNVISLREAFIHSMTKEVWLLFDYAEYDLWWLCNDHRSANLRLQEPMLKSIMFQLLQGVHYL

HEQWVLHRDLKPANVFLTRGGSIKIGDLGMARVFVAPPKAFSDVDPVVVTYWYRAPEIMLGAKHYTKAIDIWSIGCIFAE

LINLVPIFHVKNDQNSRDPYYAAQLEAIFKVLGMPDEQKWSLLKHMPRYPDLKRDFKTKASKFDDVLSKRMNEPESSTRL

SLMRRMFHYDPEKRISADEALTAPWFKSVDRFDPKNCFAKATRAYPARELDKPSEVAAEAPSHGETFKHLHIALSRMPAC

LPSQQATGQNNIFVFTQMSMIKRLTFPTTTSTAVPSAPTRLRVQRLVPGDGWRVVHRSEPSQKDPVTRRTVIISASQSNP

RPMLEALGFRHSASYLHRGAENIFASQVSVRLARVSNPSTKVELTPGHDLLECICIGTNAQEATRVLEMLASTLFPQVRL

QKVDVHRSAALMTRAAR

>Sro-gi|326436992| gb|EGD82562.1| CMGC/CDK/CDC2 protein kinase [Salpingoeca sp. ATCC 50818]|PTSG_03214.1

MDRYEKIEKIGEGTYGTVYKAKLITSGELVALKKIKLETEEEGVPSTAIREISLLKELNHRNVVRLIEVIHSEHDLHLVF

EFLDCDLKKHMEVSRQLAPDLVRSYLFQLLKGIEFCHTHRILHRDLKPQNLLIDSDGNIKIADFGLARAFGIPVRAYTHE

VVTLWYRAPEILLGARQYACPVDIWSIGCIFAEMVTTRPLFPGDSEIDELFRIFRYLGTPNEHVWPGVSELPDFKTTFPQ

WKRQDLAKLVPGLDPTGLDLLEQMLRYAPSARISATRALRHPYFAAYNA

>Sro-gi|326436991| gb|EGD82561.1| CMGC/CDK/CDC2 protein kinase [Salpingoeca sp. ATCC 50818]|PTSG_03213.1

MEGGYTMDRFEKTEKLGEGTYGSVYKAIDKTTMAVVALKKIKLNDQEEFGVPASALREIALLRELDHPNIVQLLDVIPSS

SELHLILEYVYEDLRKFMHRVKVLERPMYQSFLRQLLLGLEYCHIHRILHRDLKPENLLINHRTGALKLADFGLARAFGI

PVRAYTHEVVTLWYRAPEILLGSKQYACPVDMWAVGCIFAEMASSKPLFPGDSEVDQIMRIFRYLGTPTEKTWPGVSNLP

DFRANFPRFPAIDLAPIVPQMDPVSMALLQHMLVYLPASRIPANQALKHPFFQGPEHDFAASAAEQQQQQQQQQQQQQQQ

QQQQQQQQEEEADSTATTSPSSTATTTAAANN

>Sro-gi|326426444| gb|EGD72014.1| CMGC/CDK/CDK5 protein kinase [Salpingoeca sp. ATCC 50818]|PTSG_00030.1

MSEGRYVRICKVGEGTYGQVYKAQDRQNSQIVAIKAITLNGGAEGVPSNAVREISLLKRLDHPNIVRLLDVLYSQTRLMM

VFEFCDQDLKQFLSHKPIQHDPNLAQTIMFQLLRAVAYIHSQHILHRDLKPQNILLDRKGRLKLADFGLARPIGVPVSKY

TADVVTLWYRAPDVLLGSEHYGPCIDLWSTGVIFAEVLTGQPPFQGNSINEQLLLIFDMLGTPPPSWDMRQYERYKDFAP

VLSMRHIPATSVGLETFLSRAAPVAVDLVKALLRYNPDARITAEAAMEHEFFAPLVAAVKESKKSRKPKWHKRSKDAHAQ

QAHAQQAHAQVQPQASSAHHPPHQQQQQQQQQQQQQQQQQQQQQQQYLPQPQHEQHQQLFQAQPQPQQFQYHHHYEQQQQ

QQQQQQPQPGDGDGNAAMMMSGIEQPSHPHVHAHQHLPPPPMPPMPHFNPHEYVQQQYHQPLSQFHEQIQQPSQPPQHPQ

HLFGEGMYKHDQQHHQ

>Sro-gi|326430756| gb|EGD76326.1| CMGC/CDK/CCRK protein kinase [Salpingoeca sp. ATCC 50818]|PTSG_01028.1

MDNYRIERRVGEGAHGVVFLGVHLASGQRVALKKVTLARLDDGIPTQVIREIRALCQLTHKNVVTLHDVFPSGMGIMLCF

EYMASDLARVLQGQNLPLSAPHVKRYMSMLLSGVDFCHSHAIVHRDLKPANLLISATGQLKIADFGLARVYDEARPMSHQ

VATRWYRAPELLYGARVYDFGVDIWAVGCIFGELLNNSPLFPGENDIDQLSCVIQALGTPTRQDWPELDSLPDFAKINFD

PTDPQPMHEILPDATQDAINLCSQFLVYSSSRRLPAAKALVHPYFFSYPLPAHHSELKLPTSSQPESMRLRLVSDLPFVT

FEDDDDDEL

>Sro-gi|326431660| gb|EGD77230.1| CMGC/CDK/CDK10 protein kinase [Salpingoeca sp. ATCC 50818]|PTSG_08323.1

MEGGGARFRGGRSHRGDDGDASGSSRNSVGEGVRGRLASARDSLKEAEYTIKVPRGFAGRCREIDDFERLGRLGEGTYGI

VYKAKDIETGAIVAVKRIKMKDEREGMPQTSLREVTTLKAMEHENVVQLLDIAVGGAHDQVYLIFEYCEHDLAWLVDNLP

APFPETVAKSLTVQLLKGLRALHSMFIVHRDIKLSNLLLNSRGYLKIADFGLARRSGDPPRPKTTNVVTLWYRAPELLFG

DKAYTSKVDCWSAGCVMGELLAHKPILPGKSEVSQLDLIIQLLGTPNEAIWPGFSSLPLASRFQLTAQPYSNLKDEFRFI

SDRGIDLLQRLLTYDPHQRWSCDRALGHAYFREFPYPCTPDMMPTFAVDGRGDEEATEEREEAWEDRGTGKRPQQPRQHS

RGGGGGDGGRDRDRGRDRGRGDRDRDGDDGGGGGGGRGDDGRSRRRRRSSPSHAQRSTRSKGDHRR

>Sro-gi|326434528| gb|EGD80098.1| CMGC/CDK/CDK7 protein kinase [Salpingoeca sp. ATCC 50818]|PTSG_10371.1

MSSAYKKIAVLGEGQFGTVFLAEHTDTKERFAVKKIKVGSKQDAEEGLHRTAFREIKFLQELRHANIIQLRDIFAKGFNI

HLVLELCKCDMRAIILENIQLTPSDIKSLMLQCLQGLEYLHSHWIIHRDLKPENIFITRKGIVKLADFGLASTFGSPSRA

YTAQVVTIYYRAPELLFNSKAYGAGVDVWAMGCVHGELELRRPLLPGTSEIDQLSRIFALRGSVNEHNWPGVTKLPGFLE

FDQQNPTPLRHVMPAASDLALSLMDGLLTCDPAKRLTIKQALKHAYFSKAPGPTPPHLLPLPSGSGGGGGGGDDDGDGKE

KKEGDAGAKPSKSDVSAKRRRAADAPDASGAKLSRALFQPDDDDDGDGDGKA

>Cow-gi|320162599| gb|EFW39498.1| protein serine/threonine kinase [Capsaspora owczarzaki ATCC 30864]|CAOG_00023.2

MDKYDRIEKLGEGTYGETGEIVALKSIRLDNEDEGVPCTAIREISLLKELKHPNIVRLHDVLHADKRLTLVFEYCDQDLK

KYLDECAGDIGVMTMKSFLFQLLRGIAFCHEHRILHRDLKPQNLLINKRGELKLADFGLARAFGIPVRAYSHEVVTLWYR

APDVLLGSRRYSTSIDIWSAGCIFAEMAMGGRPLFPGSSTLDQLMRIFKVLGTPNEEIWPGVSSLPEWKPDFSVCRRVPL

SSVVTTVDSYGIDLLARMLMYLPDARISADDAMCHPYFSDLQANVRAMA

>Cow-gi|320167054| gb|EFW43953.1| serine/threonine-protein kinase pctaire-2 [Capsaspora owczarzaki ATCC 30864]|CAOG_01997.2

MATTATSTAAPPPTQQPPVTTTTTTTTTTVTTTAAAAGISIHIALDEAASSSAASFSSSGLGSNKPKKSFERDRSGSLLN

KLVRRLSLKRHGDSEPSSPTHARTGFDFESDSLAVAYGAGGSADSNSNEDIASAASSSSSSSVSSASSSYDPSTTQSVPD

SPKRTSVASKLVRSFSSKEKDPNKKSMRKSSSSTTSRFLNGGEEPRKGKSPRKDAHQPSALSNESLAPAQPATETAVKAQ

RRLSLPLSLTKEGAAEASERAASPTQDQAFGQVFSYKKLEKLGEGTYAIVYKGMSCITGDYVALKEIKLEQEEGYPCTAL

REVTLLKELKHANVVTLHDVIPAESSLTLVFEYVPMDLKNCMDKSLGFLDLFNIKLYMFQLLRGLAFCHRKKILHRDLKP

QNLLIHHNGELKLCDFGLARAKGVPIKTFTNEVVTLWYRPPDVLMGSTDYTSSIDVWSAGCIFAEMVGGRPLFPAANPTE

ELLLIFKTRGTPNPQSFPNIEKLPGYSTSFPQYPVQPLSSFAPRLSADGLDLLEKMLQLDPSKRVTCEEAMRHGYFADLP

REVLTLVDNKSIFTIPSIKMKGHVYRRSSMPSSKLNRNRSKSVYNPQE

>Cow-gi|320165016| gb|EFW41915.1| protein serine/threonine kinase [Capsaspora owczarzaki ATCC 30864]|CAOG_07047.2

MSFHLSDAYARKLEQYELSTKLGEGTYASVFRAIHKPSSTLVALKQINLNRDEGTPCTALREISLLKELRHANIVALLDV

AHTRERLTLIFEHLDCDLKQHMDACGKNLAPANVQLILYQVLRGIAYCHSKSILHRDLKPQNLLLNRATGDVKLADFGLA

RAFGIPVKAFSHEVVTLWYRPPDVLMGSQVYSTSIDMWSIGCIFGEMTTGRPLFAGKNVDEQLARIFKQRGTPTELTWPG

VSQLPNFRGDFPVTPAVQLASIVPKMDSLGVTLLNRLLQYNPAMRVSAAEALQHVYFASIHAIVGNLPNEQSIFDRTGIT

MILEPPVEAAQAPAASTAAAAAAIIFVDAVIGWELMNEMNL

>Cow-gi|320166971| gb|EFW43870.1| protein serine/threonine kinase [Capsaspora owczarzaki ATCC 30864]|CAOG_01914.2

MSTETLSSAESSSSSSSSSSSAPVLATAAAARRHDALAWHLRHYISAAPAVSVSPEGADAQAAADAAAAAAAAADATPSA

TSTAAATATTTAAGATVLALPATAETGPSKRVGALLATNRSDVYERLDFLGEGQFATVYRARNKSTGEIVAIKKINLGKM

EDAQNGLNRTALREIKLLQELHHTNVIGLVDVFGHARSNNISIVFEFMDADLEKIIRDPRNVFQPGDYKSFMLMTLQGIE

YMHDRWILHRDLKPNNLLISGAGVVKLADFGLARDYGSPDKIYTNQVVTLWYRAPELLYGARCYGTGIDIWATGCIFAEL

LLRKALLPGNNEMSQLTQICSLFGAPTEKTWPGVTSLPTYVSVKDYQPTPLRQLFTAASPDCVDLIGKMLTMNPSGRCTA

TEALQHAYFSNDPPPTAPAQLPRYVLSAKEERDQAKRRFEEAEGLPSGMRPLKVARLEF

>Cow-gi|320167092| gb|EFW43991.1| cyclin-dependent kinase 8 [Capsaspora owczarzaki ATCC 30864]| CAOG_02016.2

MNPAYKEHKQAQRQTVDAWFDYADYKIGEGTYGVVYKARKKIVDPHTTPIPYALKKIKATVQTEGLSTSACREISLLREI

KHENVICAPEIFLNPLTRDVWLLFDYAEYDLYQIIEYYRDRKGQANAAAQQTQTPPQPVFLEEHMIKSLLWQSLNGIHYL

HSNWILHRDLKPANILVMGAGKEHGRVKIGDLGMARLFQSPLRNLADVDPVVVTIWYRAPELLLGAKHYTKAIDMWAIGC

IFGELITARPIFLGEQITTKQTKTPFQGDQLDKIFQVLGFPNEKDWPELSSLPDHPILMQNFLNKQLYDKCSIEHYFRGY

MKMQRGPLSPAGLGLLSEMLTMDPNKRITAEAALRSNYFKESPTPSANAFEHLKFSFPPRSPIREDKDKNNRPKPKTEAR

PAAAPPNQQQLHTQRVAHSQPPLHAGLPNSANRPQVRGPGQPGSGVHPHPSAMGQQHQYHR

>Cow-gi|320165708| gb|EFW42607.1| galactosyltransferase-associated protein kinase [Capsaspora owczarzaki ATCC 30864]|CAOG_07450.2

MPATRPAVRASSMLNPATAAAATATAASRAASSAPEHPPAASVPPTASAPVPAPAHAPAATAPPSGSHSHHQRPRPPTDL

KPYEPKHRPFPFCRSLAEFDKGNKVGDGQFGEVFRAYDKLHNNRPVALKSVKMQEEQEGFPITSVREIKILRWQSSHENI

VRLYDLVREESRDKRPETFYLCFEFMDCDLEALIKTPTVVLSSGIIKCYVKQIMTGLNHMHLNNVVHRDLKAANILINNR

GQLKLGDFGLARVLLEKKNKDMEGGYTNRVVTLWYRCPELLLGDTAYNTAIDMWSVGCIVYEMYTRSTLFREESEMAMLR

KIIELCGSPAGESWPDVEKLPNFKDVSGLNCKRRVREALNSKIQDLEAVNLIDCLLTLNPAKRYSATQCLDHDYFWKSPL

PLKPEESASRLKCIAVRHTHLAIQGKPTVIRGNPTMVIQRIHSMDGIRHHMPLRLAMLRYRR

>Cow-gi|320165526| gb|EFW42425.1| cyclin-dependent kinase [Capsaspora owczarzaki ATCC 30864]|CAOG_07268.2

MKDEDDELGLQPEHHSSLVLSSSRRRPDAFDHDNDDGDGDVNDNDNDDYRDEGADGEAGDDDDLALRSMAGNGWRETTAM

GAAAAAADVGQLVTKSSHHEQAADSAEESPDDNPDDDDDGDDETETMHQNGASRNGPSTSNTNTHTSSSATLLTIETKSS

PSSAERNRNRVVPSLDKAVLHGVHSSKAEAKPWLGDLRSFVRKRIDSLKQRSSSSSTSDAVFTSWFRHIKNLASQHSVAN

NSSVKRLDAVIAALATEQPTLHCSPDVLAADENDLSLLDLSQLLPTDAPDLYKETLDILVRAFPLLHPRDLAPCLNSCFG

KEPFAQRIKYDPWVGGAPMYIPLGKVGEGSYGTVYKVVACHGFPTFHEKFYALKKIKSRTQSNGMPRAVIREVSILRELS

HDNIISLVDVIYFPHHSVAVGKRDTESHRFLQWLQEIIRFHRYSSNFAERFTEQVLRRASGLRGYSSQGIRAIAAKDRLH

PLTIKAIMWQLLQGLDYLHDNWIVHADLKPNNILISSQGQVRIGDFGLSRLFLGPREAQEPVLVTMWYRAPELLLGDSKC

SRAIDMWSVGCILAEMFLLQPIFRGKPLPEPKTRDQFEQDQAMKIFHALGLPTVEAWPEVTTMRFWPLIEKWESVHPKVY

KFSLDSMLKHVLPDASIELQLLRGLFTFDPSKRLRTRQALTHAYFTSSPPVSLLNAFAPPPNTSQVIYPPRMYQWMYDLL

KPSSVHQQQHGQPKKQITAAQSVFLRLCHHNDMCFYLRHLQIFHGLSLPAGMVSSVDDQILMQLVIANVGCRFIAPPLAE

CFDDPEHGAWISAPASILPSGYRSLEHELLQVLCDDQVPAAELGTTFRRCLLQHSHEVLSVVDLLQELAAHVKASSAAID

DAKERQRSLATRTAVSTATVTPSDGAAPRSSRTAPTSSSRHWSSTAAADGTPSASSSSASAAASGMLEHLSDHVRKPVTP

SSAAAAAGHRDARLYEDLLVRLQLAATLYMRHLTTTNPRFLSVFWLLEVILYCDNLADTALHQLLGIPTTMYRKMVDNTD

PALPAVNAATPAKTDSKASRAQAALTPVTATGPTVALASDGLLDASRPGRANFPQGKKSGCGITAMGGRKSLTRTNVSMR

STTDLPHEPVRPDPSAVEVGSRNRGARIVVASVPQKL

>Cow-gi|320167411| gb|EFW44310.1| cell division protein kinase [Capsaspora owczarzaki ATCC 30864]|CAOG_02335.2

MEQSESGNPTTATAPAAAAAPTRPSISFNSSTNSYVRNVDASADAADADADAAADDRGGSGSGYSSRSRHRRRDTGSGSA

AAGSRSGQGAGAAGSGGFRRDERVSSSSSSASWHDRSRNGGDDRRDSHRRKDSGDHHHYHRSGSRSTHHDSRFESGNGSA

RGHGHSGNSHNSGSSDAAHRDRHDERDRDRGRERDERDERGERGDDHDQSRTFTTASSDPAAHGGSISSGSSGGDGSSND

ILSQQQQQQQQRRTTTTTTTTAGADPLVLHAEPSTQDRSKVHGEHAAEAAHDRLQPHDYHAQVGANLRSLNDNDDRAAAT

ALGPYGQHGPYGHHGADAGTVFATGSVDPVAAALGVASMSAGAAGFGFVATDASNDVDDAVVALAAAAATNDVVIPLSAV

MQHGQEKRRERKWNGTTSAPPPPPHNRHDEGLHRKFRGGGRGRSPGRRRSRSRSPSRSRSPSRSPSRHHGRSHNRDYHRR

GRSKSPRRSRSRSPRDRSRSPHHASSRGHRSGRAASQSPPPHTTSSNKGDDSNMTADPNESVSGAHPPSATTHAEPTGAA

DNPEKDVPADSKSSSKQQSRGLPSHRDSKSSSYKRSGESSSRFGSRRDTYGRKRTRSPSRSRSRSPTSRARSHSRSPSRS

RSPHDDRRSRHGGDDAGRERARQRREKDAEPAASEQEGQDTAAALPRTGEDDVAAAVAADGDAASADMPAASSEEARLRD

LELLELERERDRKKSRDRHHDSDSGFSDRRGSSRSDRRHTDDRRRDDSRSHRDDGGHRRDFEDRTRFGKSAREYRFISKN

SGGNDAMSPTRRGEAASSSGSFPPPPAGFAHPQSAGGPMYQPNAFDNPAAAAAAAAASAYAQQYQQYQQYQLQQFQFMHQ

HQQAAMAMAAAAAASGSYGFPGVGTGGGMPDAAAQAQYQALMQYQLLFRQQQMLQQQQQQQQQQQQQQQPQQELPPQQQQ

QSSDATNSTNTNPVTATGDTTAVPADASAGGAPARRVWQPAFVEDAYVILEQVGQGAYGKVYKASNRATGEIVALKRIRL

DREKEGFPVSAVREINLLRRLRHPNILCLKDIVTERSDDAASSPSPKPSELSGGEGGHATSATASVRADPVVEWSRNSPT

MQRIFQQSGPPRPRPKLSDSVYLVFEYMDHDLSGLLHSGLMQFSVDQVRSLMYQIIQGVNHCHQNNIFHRDLKGANLLVN

KHGEVKLADFGLARAFIEERRAYTNEVITLYYRPPELLLKETEYGPEVDVWSCGCILAEMLGNQVAFPGRTETEQLDLIA

RVCGTPCEENWPGVSNLLQQTTFKRQYPRRLHEHFHSFPPDALDLLDKLLVLDPRRRITAAEALNHPFFTQVAFQIYATL

PTDLDCHEFVTKKLKQKPKPQSESEPQAGDASNKQPSSSKPSGDATDAPVSMTVDQDNSSSEAKSMDLST

>Cow-gi|470363760| ref|XP_004365702.1| cdk10/11 [Capsaspora owczarzaki ATCC 30864]

MSGHSERRPRAASESDRDSHAAGSSSASEHSEDGTSAAHDSRKRPRMDAHRFHHEDGGQVPSSSTLPATDRNEQSEEGSK

DVKTKLLEQHRVTPAGAVAQHAVDVAPRRHVDDSDDNDNDAAAATSRIEKYYPAFQGCRSVDEYTRLNQIEEGSYGVVFR

ARDVRSGRIYALKRLKMEKEKDGFPITSLREIDTLLKSPHPNIVLVREIVVGSSMDHIFLVMEFVEHDLKTLMESMRQPF

SGGEVKTLMLHLLAGVNHLHDNWIIHRDLKTSNLLLSNQGVLKLADFGLAREYGSPLHAMTALVVTLWYRSPELLLGETK

YTTAVDMWSVGCIFAELLIHEPLFPGQRELQQLRMISDMLGPPSKEIWPGYENLPNAQVLSFSKDQPYNRLPTKIPGLSA

QGLKLLNGLLTYDPKKRMTAEQALRHPYFSESPLPVDPSVFRSWPAKSELQKVHTSHDEPRAPEGHPSEVAADGDAAGFI

FESDDVYRLLQHGSNAQVGGGFKLKF

>SARC_08063.1 CMGC/CDK protein kinase (Transcript:SARC_08063T0)

MPSHKTACESGTATPGSSVDSSGGPKIRLRFSRTLITDAKKLRAIHEECTKSGAGTPSISEDRTEAPSTIEDRTGAPSTN

EGRTGTGAPSTQAQPVIHQVDGEHTEDVGGVGERRSRVPADGTSSVELVRTGELRLTFRIPGNTVETASAKHTPATQPTS

SSSSSSPTQAKHTPNPTKPRARTPTPTPVPAPKPGVATSQGLKRRHDGISPSTSTGSIKGVAAKRPNYDSWEKLVSRPSS

AEPRKPPLPGVDKAVAVVGKPPVKGTGALGAKALAAMCNGTGAGNGGTQGHTATAGTAAGVIRRVSGGAVGGNRQTSASV

GSVQSDVPGAAGGVPSHVPGSTAGTVRPTSAHTAGAKDHTQGHTHSKTHISGHTTGPKKSLADSTSASRSVRSLESGEIP

PSGQSAVATSPQTLPLKDSHENGHSVRGKAPTTATVPRSTRSLESGELSSTTTHAHVSSIKYTHAHAHGRDGRDKHHSAD

QTQHSHSHGDGRERTRAHDTHRHHNERSHTQDPTRTQSTGEAVAAARHSTHSHAHRSKSHGGHHRHGPHPYNRTGAGTST

EHLAKHQSTTRARLSSRSPPKGPDVPAGALAVRTHTRESSTSTAPPTPQTLKRTHTHEAGTSTVSPTPPPPPPPLPGTAP

PSPEAAPPPPPPPPDSAPPPPPLPDNGNGPSGQQATGGPTTTHAHTQPTHRIIKAVVRPAQAINRPPVRQAVGNHWPNKA

GHPTNGSNHMAPPPPPPLEPPPATPPVPKPNQAPNPSPAEAPRNPLKLSSNSTMRIKKRDPGAKGGATVVTHMPFKPRHP

RATGCLKNPHHLGLASEYRKLGLVGKGTFGEVWKSKDLDDPSKFVVLKKILEETEVEGFPITALREIMLMQRLEHKNILH

AKRVVYNPPKAGKDKYGNELKSTFYMVSSWMDHDLAGLLNRGKAFSVAQIKCLFKQLLVGLDFLHVRKILHRDIKCANIL

VNSAGLLKIADFGLARTFDDKQLKNGLTNRVVTLWYRPPELLLGERMYDTKIDMWGAGCVLGEMANTAPLMKGNSEIEQL

DCIFKLCGTPNADNWAGGTELEVFQHTCKNWVQYPSMLWQELRKLKQDTDVTQPRKNEKMEPGRMEPFVHIMEGLLCLDP

RKRLSASDALDHIFFWTDPNPLEPEEMGLHDVASSHELRVGIDKQNHPTQFPPIKIPIKIRSTYPLGGVLVNKMTGEVEV

YEHNRNRPNNPHQNRPGGVGHPYRPMHSNVGQINQVQRPINPGQRLRGPSKPNQQHQGLDRRRLQQQQQPNRYGPGAPRP

YKPLNTGAGAGTGGGISNVKPAIGGYGYANGQGSRPDARGKGPDMNSNASASVKSAPTTGWVPQPYRPRNYNPLNGP*

>SARC_10569.1 CMGC/CDK protein kinase (Transcript:SARC_10569T0)

MHSDKPAEKEKSTEKEKPVEKGKAYTMFKKKFATLRSHSSRGSASITSFSVTGLNEAPTSRQNSTARTSIDKDIASTHHL

TPQAQIKLMNSKSSSNVRPGVSPNRHAQESKLHKSAHLLFVDSNEQPVRRKSMPHVVSTQTNKQLGYGKVESYQKLEKLG

EGTYAQVYRGVSTITGEQCALKEINLDAEEGAPCTAIREASLLRELKHVNIVILHDIVHTHTSMTFVFEYLNMDLKDYID

RAAPYVALTNVRLLFYQLLRALECCARKRILHRDIKPQNILLNEIGELKLADFGLARAKGVPIKSFSHEVVTLWYRPLDI

LLGSVDYNSSLDIWSAGCILAELVTGTPLFPGKDHDSQLTTVFSKLGTPSPDTWPELENLPLYSKIWTMYAPKPFTYLVP

RLESEGIRLLSEMVVYEPAKRISAKEAMRHEYFEDLPCEIHALRDDQSVFSVPGVRMFP*

>SARC_06703.1 CMGC/CDK protein kinase (Transcript:SARC_06703T0)

GDVVALKRIRLDSEDEGVPCTAIREISLLKELRHPNIVRLYDVLHTDKKLTLVFEYCEQDLKKYLDGCGGTVEPFQVKSF

LYQLLKGVAFCHEHRVLHRDLKPQNLLINKFGKLKLADFGLARAFGIPVRSYSHEVVTLWYRAPDVLLGSRRYSTSIDIW

SAGCIFAEMVQGGRPLFPGLAAPDQLLRIFKFLGTPTEEIWYGVTELPEWSKYEFPMYAAQPLSSAVNNLDMSGLDLLKS

MLVYMPDFRISAEDALRHRYFKDLPAHIRA*

>Sce-kin28p-gi|6320095| ref|NP_010175.1| Kin28p [Saccharomyces cerevisiae S288c] Kin28p

MKVNMEYTKEKKVGEGTYAVVYLGCQHSTGRKIAIKEIKTSEFKDGLDMSAIREVKYLQEMQHPNVIELIDIFMAYDNLN

LVLEFLPTDLEVVIKDKSILFTPADIKAWMLMTLRGVYHCHRNFILHRDLKPNNLLFSPDGQIKVADFGLARAIPAPHEI

LTSNVVTRWYRAPELLFGAKHYTSAIDIWSVGVIFAELMLRIPYLPGQNDVDQMEVTFRALGTPTDRDWPEVSSFMTYNK

LQIYPPPSRDELRKRFIAASEYALDFMCGMLTMNPQKRWTAVQCLESDYFKELPPPSDPSSIKIRN

>Sce-cdc28p-gi|6319636| ref|NP_009718.1| Cdc28p [Saccharomyces cerevisiae S288c] Cdc28p

MSGELANYKRLEKVGEGTYGVVYKALDLRPGQGQRVVALKKIRLESEDEGVPSTAIREISLLKELKDDNIVRLYDIVHSD

AHKLYLVFEFLDLDLKRYMEGIPKDQPLGADIVKKFMMQLCKGIAYCHSHRILHRDLKPQNLLINKDGNLKLGDFGLARA

FGVPLRAYTHEIVTLWYRAPEVLLGGKQYSTGVDTWSIGCIFAEMCNRKPIFSGDSEIDQIFKIFRVLGTPNEAIWPDIV

YLPDFKPSFPQWRRKDLSQVVPSLDPRGIDLLDKLLAYDPINRISARRAAIHPYFQES

>Sce-pho85p-gi|6325226| ref|NP_015294.1| Pho85p [Saccharomyces cerevisiae S288c] Pho85p

MSSSSQFKQLEKLGNGTYATVYKGLNKTTGVYVALKEVKLDSEEGTPSTAIREISLMKELKHENIVRLYDVIHTENKLTL

VFEFMDNDLKKYMDSRTVGNTPRGLELNLVKYFQWQLLQGLAFCHENKILHRDLKPQNLLINKRGQLKLGDFGLARAFGI

PVNTFSSEVVTLWYRAPDVLMGSRTYSTSIDIWSCGCILAEMITGKPLFPGTNDEEQLKLIFDIMGTPNESLWPSVTKLP

KYNPNIQQRPPRDLRQVLQPHTKEPLDGNLMDFLHGLLQLNPDMRLSAKQALHHPWFAEYYHHAS

>Sce-ctk1p-gi|6322710| ref|NP_012783.1| Ctk1p [Saccharomyces cerevisiae S288c] Ctk1p

MSYNNGNTYSKSYSRNNKRPLFGKRSPNPQSLARPPPPKRIRTDSGYQSNMDNISSHRVNSNDQPGHTKSRGNNNLSRYN

DTSFQTSSRYQGSRYNNNNTSYENRPKSIKRDETKAEFLSHLPKGPKSVEKSRYNNSSNTSNDIKNGYHASKYYNHKGQE

GRSVIAKKVPVSVLTQQRSTSVYLRIMQVGEGTYGKVYKAKNTNTEKLVALKKLRLQGEREGFPITSIREIKLLQSFDHP

NVSTIKEIMVESQKTVYMIFEYADNDLSGLLLNKEVQISHSQCKHLFKQLLLGMEYLHDNKILHRDVKGSNILIDNQGNL

KITDFGLARKMNSRADYTNRVITLWYRPPELLLGTTNYGTEVDMWGCGCLLVELFNKTAIFQGSNELEQIESIFKIMGTP

TINSWPTLYDMPWFFMIMPQQTTKYVNNFSEKFKSVLPSSKCLQLAINLLCYDQTKRFSATEALQSDYFKEEPKPEPLVL

DGLVSCHEYEVKLARKQKRPNILSTNTNNKGNGNSNNNNNNNNDDDDK

>Sce-sgv1p-gi|6325419| ref|NP_015487.1| Sgv1p [Saccharomyces cerevisiae S288c] Sgv1p

MSDNGSPAVLPKTEFNKYKIGKVKSTPAIQRDAKTNLTYIKLRKRSSEKVYGCTVFQNHYREDEKLGQGTFGEVYKGIHL

ETQRQVAMKKIIVSVEKDLFPITAQREITILKRLNHKNIIKLIEMVYDHSPDITNAASSNLHKSFYMILPYMVADLSGVL

HNPRINLEMCDIKNMMLQILEGLNYIHCAKFMHRDIKTANILIDHNGVLKLADFGLARLYYGCPPNLKYPGGAGSGAKYT

SVVVTRWYRAPELVLGDKQYTTAVDIWGVGCVFAEFFEKKPILQGKTDIDQGHVIFKLLGTPTEEDWAVARYLPGAELTT

TNYKPTLRERFGKYLSETGLDFLGQLLALDPYKRLTAMSAKHHPWFKEDPLPSEKITLPTEESHEADIKRYKEEMHQSLS

QRVPTAPRGHIVEKGESPVVKNLGAIPRGPKKDDASFLPPSKNVLAKPPPSKIRELHQNPRPYHVNSGYAKTAIPPPAAP

AGVNRYGPNNSSRNNRFSGNSTAPNNSRNPVNRFHPETNVSSKYNKVPLPLGPQSRYQGNSNESRYKNSPNDSRYHNPRY

VNKPETNFNRQPQKYSRQESNAPINKNYNPSNGSRNMAGDHHQGSRPSHPQFPISPSQGQHQLTSKPIEKKNGSFKDERA

KPDESKEFQNSDIADLY

>Sce-ssn3p-gi|6325215| ref|NP_015283.1| Ssn3p [Saccharomyces cerevisiae S288c] Ssn3p

MYNGKDRAQNSYQPMYQRPMQVQGQQQAQSFVGKKNTIGSVHGKAPMLMANNDVFTIGPYRARKDRMRVSVLEKYEVIGY

IAAGTYGKVYKAKRQINSGTNSANGSSLNGTNAKIPQFDSTQPKSSSSMDMQANTNALRRNLLKDEGVTPGRIRTTREDV

SPHYNSQKQTLIKKPLTVFYAIKKFKTEKDGVEQLHYTGISQSACREMALCRELHNKHLTTLVEIFLERKCVHMVYEYAE

HDLLQIIHFHSHPEKRMIPPRMVRSIMWQLLDGVSYLHQNWVLHRDLKPANIMVTIDGCVKIGDLGLARKFHNMLQTLYT

GDKVVVTIWYRAPELLLGARHYTPAVDLWSVGCIFAELIGLQPIFKGEEAKLDSKKTVPFQVNQLQRILEVLGTPDQKIW

PYLEKYPEYDQITKFPKYRDNLATWYHSAGGRDKHALSLLYHLLNYDPIKRIDAFNALEHKYFTESDIPVSENVFEGLTY

KYPARRIHTNDNDIMNLGSRTKNNTQASGITAGAAANALGGLGVNRRILAAAAAAAAAVSGNNASDEPSRKKNRR

>Spo-srb10-gi|19114301| ref|NP_593389.1| cyclin-dependent protein Srb mediator subunit kinase Srb10 [Schizosaccharomyces pombe 972h-] Srb10

MKDGYKIIGFISSGTYGKVYKAVSSNSNDKRLFAIKKFKAESKQVSSNAQQTGVSQSAIREMMLCREIQHENIVSLVQVL

LKDGTISMVFEYAEHDLLQIIHFHSRSRTRQIPPSILKSILWQIINGVAYLHENWIMHRDLKPANIMITATGKVKIGDLG

LGRLIRDPILPFYSSDRVVVTIWYRAPELLLGAHDYTPAIDVWAIGCIYGEMLALSPLFKGDEIKMEDKKVVPFQSTQML

RIMELLGTPTEERWPGLKNYPEYYQLSSFEVRYWNNLLPQWYQTVKNRDPQGLDLLMKMLQYDPKSRITAKQALEHVFFT

SDKLWTTRYVFFLFLFIIERNKLLLTHFLAHF

>Spo-ppk23-gi|19112531| ref|NP_595739.1| serine/threonine protein kinase Ppk23 [Schizosaccharomyces pombe 972h-] Ppk23

MAGSKWETEETNQFAIENQKLEEEWRKKRRLEKKRKRKILEEEEKAEERNIDACRLYLMGNTPELKSCNSIDDYEILEKI

EEGSYGIVYRGLDKSTNTLVALKKIKFDPNGIGFPITSLREIESLSSIRHDNIVELEKVVVGKDLKDVYLVMEFMEHDLK

TLLDNMPEDFLQSEVKTLMLQLLAATAFMHHHWYLHRDLKPSNLLMNNTGEIKLADFGLARPVSEPKSSLTRLVVTLWYR

APELLLGAPSYGKEIDMWSIGCIFAEMITRTPLFSGKSELDQLYKIFNLLGYPTREEWPQYFLLPYANKIKHPTVPTHSK

IRTSIPNLTGNAYDLLNRLLSLNPAKRISAKEALEHPYFYESPRPKDPKFFPTFPSKAKGESKEKNVFQSFRSASPKK

>Spo-cdc2-gi|19112421| ref|NP_595629.1| cyclin-dependent protein kinase Cdc2 [Schizosaccharomyces pombe 972h-] Cdc2

MENYQKVEKIGEGTYGVVYKARHKLSGRIVAMKKIRLEDESEGVPSTAIREISLLKEVNDENNRSNCVRLLDILHAESKL

YLVFEFLDMDLKKYMDRISETGATSLDPRLVQKFTYQLVNGVNFCHSRRIIHRDLKPQNLLIDKEGNLKLADFGLARSFG

VPLRNYTHEIVTLWYRAPEVLLGSRHYSTGVDIWSVGCIFAEMIRRSPLFPGDSEIDEIFKIFQVLGTPNEEVWPGVTLL

QDYKSTFPRWKRMDLHKVVPNGEEDAIELLSAMLVYDPAHRISAKRALQQNYLRDFH

>Spo-pef1-gi|19075421| ref|NP_587921.1| Pho85/PhoA-like cyclin-dependent kinase Pef1 [Schizosaccharomyces pombe 972h-] Pef1

MNYQRLEKLGEGTYAHVYKGQNRVTGEIVALKVIRIDADEGTPSTAIREISLMKELRHPNIMSLSDVLQTENKLMLVFEY

MEKDLKKYMDTYGNQGALPPSQVKNFTQQLLKGISFCHENRVLHRDLKPQNLLINSRGELKLADFGLARSIGIPVNTFSN

EVVTLWYRAPDVLLGSRVYSTSIDIWSVGCIMAEMATGRPLFAGSNNEDQLLKIFRLLGTPTEQSWPGISLLPEYKPTFP

IYKAQDLAYLFPTFDPLGLDLLRRMLRLQPELRTTGQDALQHAWFLTA

>Spo-cdk9-gi|19112408| ref|NP_595616.1| P-TEFb-associated cyclin-dependent protein kinase Cdk9 [Schizosaccharomyces pombe 972h-] Cdk9

MKRSSSVSVEDEKSARRKLDVVPKLHFVGCSHLTDYHLMEKLGEGTFGEVYKSQRRKDGKVYALKRILMHTEKEGFPITA

IREIKILKSIKHENIIPLSDMTVVRADKKHRRRGSIYMVTPYMDHDLSGLLENPSVKFTEPQIKCYMKQLFAGTKYLHDQ

LILHRDLKAANLLIDNHGILKIADFGLARVITEESYANKNPGLPPPNRREYTGCVVTRWYRSPELLLGERRYTTAIDMWS

VGCIMAEMYKGRPILQGSSDLDQLDKIFRLCGSPTQATMPNWEKLPGCEGVRSFPSHPRTLETAFFTFGKEMTSLCGAIL

TLNPDERLSASMALEHEYFTTPPYPANPSELQSYSASHEYDKRRKREQRDANSHAFEQTANGKRQFRFMTRGPSDPWYGI

RRPNYNSQPQYQRGSYNREGGNMDRSRNVNYQPKRQQNFKPLTSDLPQKNSEFSETNAMNQTSNHSHADGQRYYRPEQDR

SQRLRNPSDYGRQGRQSSQSQQPAWNVSSRYQNNSKVQTTSRASENADTNKTQHNIKYIDSYVPEYSIARQSANQKTNEQ

HPSSTSLHQQSTSDLKSPSFHENSNVDDTPK

>Spo-lsk1-gi|19115305| ref|NP_594393.1| P-TEFb-associated cyclin-dependent protein kinase Lsk1 [Schizosaccharomyces pombe 972h-] Lsk1

MSYSKSTIYRRQGTEPNSHFRRTVEEKSQLSGTNEESLGGHTLSSNAFKNNSSSISPSSSAKDPREQRKRTFPLNDTHSS

RARQHERPFRSRKSRRRKGKKAFSPRPGSPPSPSFYRSGSQKRARNLTTKDYFAKRSESSSSASVSPISPSANRNDSKRQ

ASSFRRSPPSSVHMKPSAFNGRKVSRRPSSSPPPIPSIPHETTSSDTQKKSSVSSGFPENKHGKFHFHIPNERRSRFDQP

PSKRMALTSTARESVPAPLPSPPSGPIYTYTYPKPAYEKIDQIGEGTYGKVYKAINTVTGDLVALKRIRLEQEKDGFPIT

TVREVKILQRLRHKNIVRLLEIMVEKSSVYMVFEYMDHDLTGVLLNSQLHFTPGNIKHLSKQIFEALAYLHHRGVLHRDI

KGSNILLNNNGDLKFADFGLARFNTSSKSANYTNRVITLWFRPPELLLGETAYDTAVDIWSAGCIVMELFTGKPFFQGRD

EISQLEVIYDMMGTPDVHSWPEVKNLPWYELLKPVEEKKSRFVETFKEILSPAAIDLCQKLLALNPFCRPSAHETLMHEY

FTSESPPPEPAVILKNMQGSWHEWESKKRKSKR

>Spo-mcs6-gi|19113141|ref|NP_596349.1|cyclin-dependent protein kinase/CDK-activating kinase Mcs6 [Schizosaccharomyces pombe 972h-] Mcs6

MDIEKSDKWTYVKERKVGEGTYAVVFLGRQKETNRRVAIKKIKVGQFKDGIDISALREIKFLRESRHDNVIELVDVFSTK

SNLNIILEFLDSDLEMLIKDKFIVFQPAHIKSWMVMLLRGLHHIHSRFILHRDLKPNNLLISSDGVLKLADFGLSRDFGT

PSHMSHQVITRWYRPPELFMGCRSYGTGVDMWSVGCIFAELMLRTPYLPGESDLDQLNVIFRALGTPEPEVIKSMQQLPN

YVEMKHIPPPNGGMEALFSAAGHEEIDLLKMMLDYNPYRRPTAQQALEHHYFSALPKPTHPSLLPRKGGEEGIKHVSSDL

QRQNNFPMRANIKFV

>Cci-gi|116507718| gb|EAU90613.1| CMGC/CDK/CDK7 protein kinase [Coprinopsis cinerea okayama7#130]|CC1G_00997.3

MDIVEQANAERQRKWLKDRKVGEGAYAVVYQGREASTGRKVAIKKIKVGQFKDGLDMSAIREVKFLRELKHQNVIELLDV

FSSKKNLNLVLEFLDTDLEIIIRDRSLVFLPADIKSWMAMTFRGLEFCHRNWILHRDLKPNNLLIASDGQLKIADFGLAR

DFADPGYKMTCQVITRWYRPPELLYGCRYYGTGVDIWSVGCIFAELMLRIPYLAGESDMDQLKTIFRALGTPTEEEWPGH

TKLPDYVPVGQFPKTPLRDLFTAASADALNLLSKCLVYEPRKRISAREALNHPYFFALPYPTHPSKLPKPASKHATPPLG

EVDGNAAGGKKRKGTKRKLSSPLEDGKGRSIARRLDFGKAAPPTSP

>Cci-gi|299740157| ref|XP_001839002.2| CMGC/CDK/CDK9 protein kinase [Coprinopsis cinerea okayama7#130]|CC1G_11325.3

MSLAPRLAPAPALNHPEIRRTTTRDGIEALQSLNRGLVTTGSQPMTTIIAGGLGETAGEIIEIEIFATDPQDGQWNAQGR

HDETGTVFPSAILLPEVQNGVIHHHAPVHDLEIEAMTAIETETTDPRLMNNPPPPPTAPPKIRVVKRRTASKRTDKQDME

AYGRVFVGCGQQSDYEVTTKLGEGTFGEVHKAIQKATGASVALKRILMHHEKEGMPVTALREIKILKALKHPCIVNILDM

FVVRSSEKDPLSVYMVFPYMDHDLAGLLENERVKLQPSQIKLYMKQLLEGTEYMHRNHILHRDMKAANLLISNTGNLRIA

DFGLARSFDTNITKGGSTRKYTNCVVTRWYRPPELLLGARQYGGEVDIWGIGCVLGEMFNRRPILPGSSDLDQLEKIWYL

CGTPTQHSWPNFDALPGCDGVKHFKSNHIRRVKMTYESVGAETADLLDKLLVCNPKERITAAQALEHEYFWTDPLPADPK

TLPVYEASHEFDKRGQRNHQPMMGHHQPSKYQDPSHRLPPPAGNPQRRQGNHRGPPGPWPGAPPLSHGPHAHPPGPGSYN

MNQGPPPGMSGPPPPGFRPGPPGGIPMNPNMGPGPYPGGRPQFNGPGGPRPPGPGAYSLPPNPGGRPPFRGPHNGPPGPP

GGPGRMGGPGLPPRPSVSLPPKPAAPLGGGVALGTVPRSNVPMPGRRPQPPQPHPPPPSTGSGNGYAPNGGLNYG

>Cci-gi|299755918| ref|XP_001828973.2| CMGC/CDK/CDK5 protein kinase [Coprinopsis cinerea okayama7#130]|CC1G_03767.3

MMNYIQLEKLGEGTYATVYKGRSRTTNEIVALKEIHLDAEEGTPSTAIREISLMKELKHVNIVRLHDVIHTETKLVLIFE

YCEQDLKKYMDQHGERGALEPEVVRSFMYQLLKGTAFCHENQVLHRDLKPQNLLINRKGELKLGDFGLARAFGVPVNTFS

NEVVTLWYRAPDVLLGSRTYSTSIDVWSCGCIFAEMISGVPLFRGRDNQDQLLHIMRIIGTPSPAQFAKICKETPEIQPK

QFPNYPRLPFHQVLPKASPQALDLLDKLLKFDPAERISAADALAHPYFTTATNTPFGLNSSPSSMPPPSFNFPHSHVHQA

QQQHQQRQQQQQQQQQQQQQQQQQQQVHHQQIQPHYSRPIYGQIPQHPQNPAQVKNVQVPYGNVQYPPYGAPPR

>Cci-gi|169865137| ref|XP_001839172.1| CMGC/CDK/CDK8 protein kinase [Coprinopsis cinerea okayama7#130]|CC1G_07887.3

MQQQQQQQQQVSLDEWVSNVMQHDPMRVYRARRDAHHRPVTSKYAILGFISSGTYGRVYKAQSINGGELLAIKKFKPDKE

GDVVTYTGISQSAIREIALNREINHENVVALREVILEDKSIYMVFEYAEHDFLQVIHHYYQTIRTSIPTAVLKSLIYQLF

NGLIYLHASHILHRDLKPANILITSQGVVKIGDLGLARLCYEPLQPLFAGDKVVVTIWYRAPELLMGAKHYNKAIDCWAV

GCVMAELASLRPIFKGEEAKLDSKKNVPFQRDQLIKIFEVLGTPDERDWPGVVDMPEYRNMKLLDHFSNRLSDWCHTRIR

SPQGYDLLRQLFAYDPDNRLTAEQAIQHKWFHEDPLPTWNAFQSLAQHQIPPCRRITQDEAPSMMPMATQQNTQQAMGGG

HIAGGAGAPFSKPGSTASFASLSGGGPYAQSQGGTGGHTAHTRKKARMG

>Cci-gi|299755758| ref|XP_001828865.2| cmgc/cdk/pitslre protein kinase [Coprinopsis cinerea okayama7#130]|CC1G_03659.3

MSASAGPSTPSKKRSKWEDGDDDELDHGQPQIHPQKRRVKAKSSSTPAQDSGRGTTTAADHGHSLSKRSRSVYVPERTRH

PAIQSSRSVSCYERLNQIEEGSYGVVFRARDRQTGDIVALKKLKLDEEKNGFPITALREIYALMTCQHENVVRIREVVLP

PPLSTDAPSALHLASIRLIPPSLDEEFKTLMMQLLSAVHHCHQNWILHRDLKTSNLLMNNRGTIKVADFGLARRYGDPVG

LGGLTQLVVTLWYRAPEILLGATEYSTAVDMWSVGCIFAELLLKEPLFQAKGELELISMIFKLLGPPTKNSWPEYFDLPM

AKTIALPSPQPHQFRSKFPYLTTNGLDLLMCLLTYDPERRITAEEALQHPYFTESPLPKHPDLFGSFPSVAAGEKRRVVP

DSPSAPARAANYKMITEFDIP

>CCi-gi|299748916| CC1G_02703.3 CMGC/CDK/CDC2 protein kinase (Transcript:CC1G_02703T0)

MAGTYGVVYKARDTSTNTIVALKKIRLEAEDEGVPSTAIREISLLKELKDDNIVKLLDIVHADQKLYLVFEFLDVDLKRF

IETGNQNRSPITPALVKKFTHQLNSGLLYCHSHRILHRDLKPQNLLIDKHNNLKLADFGLARAFGIPMRTYTHEVVTLWY

RAPEVLLGSRHYSTAIDMWSVGCIFAEMAMQGAPLFPGDSEIDQIFKIFRLLGTPNEDIWPGVSTLPDYKPTFPNWSRQD

LAKAVPTLDEAGIDMLKRTLTYDSAKRLSAKRALVHPYFADYNNNYKA*

>Cci-gi|299747693| ref|XP_002911207.1| CMGC/CDK/CRK7 protein kinase [Coprinopsis cinerea okayama7#130]

MLRSRSPEGDQMGWLPPRDEDRMVSRSPSPGYRRRSPSTSDRSSHRSRSPSPYERPRAVHRLPTANSSALHTAPATGKSK

KAGKQRNGHKNARGSTGKQPARYPEDDVAADSLSMPPPPPPSRPKQSRQQTPDWRDEHTPLLNPNPNGLPPKPISLPEKP

TRIVPQRNAGFKPIGQSNSALKMFFPGDEDEADYISSSNLTKDARTEPTPTKGFTRQTLLDDTRNGREAENPHQYGNDPP

PISRPPASWDPYASTYPVDPRVPTQSYTNGLTPHLPEPPPHIPHRTGQTQSYWKSPAVSPPPESDASRWSGVPVPPVHPN

LPPTYTASVPPRPGYELPYRPPVPVDNAPVSHTTKSTPQPSPIPISDPSPAWQASASASGVATPVAPEQPESKDLYVILN

QVGEGTFGKVYKARNTVAKVHVALKRIRMETERDGFPVTAMREIKLLQSLKHPNVVQLYEMMVSNGSVFMVFEYMDHDLT

GILSQTQFKFSDSHLKSLCHQMLAGLAYLHHKGVIHRDIKGSNILLNNRGELKLADFGLARFYQKRRRTDYTNRVITLWY

RPPELLFGATVYGPEVDMWSAGCIMLELFTKKPVFQGNDEINQLHVIFKILGTPTTERWTGLNNLPWFELIKPKESLPNR

FRDLFQKWMSPAALDLAERLLTYDPELRVSAQEAMEAPYFTQERPFAERPAGLASLDGEWHELETKQERLKKRKKTESTA

>SPPG_01972.2 CCRK protein (Transcript:SPPG_01972T0) 406 aa

MLQVVFLWMRGAIPNIMKVRESDLKRVRVRDPLRLLPITLNFYGPIQNPRQSTSHPFSKTLNMPLTILYTNKKLQIGEGA

HGVVLKAKSIETGLVVALKKVPLRRLEDGIPNTILREIKALQEIDYQNVVKLLSVHPSGPSFVLVFEYMLSDLSHILRTT

PLTSSQVKAYMLMLLRGLSYCHENNIMHRDLKPANLLISPTGVLKLADFGLARVHRYAGEEVPKRPYSHQVATRWYRAPE

LLYGARMYDAGVDLWAVGCIFGELLNHSPLFPGQNDIDQLYCVLSILGTPTKESWPDLETLPDYNKIQFPNMPAVALERV

CPDASQEAVGLLKKFLVYSCKNRIPAKEALLEPYFFNKPLPAHHLELPIPERTTQEQFDVDKSLDLSVFFPLGAGGGGGD

GIVED*

>SPPG_03982.2 conserved hypothetical protein (Transcript:SPPG_03982T0) 373 aa

MADLEPTRSFRSTTSSSKWSHFIKDKKVGEGTYATVYLGWAVPIRKEKVAERLAHDHTGERERNIYNRRHFYAYSLQLID

VFSHKTNLNLVLEYLDADLEMVIKNKTVVFSAADVKSWMLMTLRGLYHCHRNFILHRDLKPNNLLLASDGQLKLADFGMA

RDYGDPHKKMTSVVVTRWYRAPELLLGATRYGYAIDMWAVGCIFAELMLRTPFMAGDSDIGQLQIIFKALGTPTEQDWPV

GAILQGMKELDGYFEFQQYPKPPLRSILTAASPDALNLLEQFLLFDPLKRITAEDVCYYGCIRPGIILTLISVQALKHFY

FRNLPRPTPPHKLPRDVGGVGAKRKEGKGEQQPQENGAQQRGVKRKGEFEGR*

>SPPG_06236.2 cyclin-dependent protein kinase (Transcript:SPPG_06236T0) 383 aa

MSSMERYIRLEKLGEGTYATVYKGKNRNDGSIVALKEIHLDNEEGAPSTAIREISLMKELRHPNIVRLHDVIHTEKTLTL

VFEYMDQDLKKYMDSLGTGGMIQPHMAKWFMYQLLKGIAFCHDNRVLHRDLKPQNLLINSKGELKLGDFGLARAFGIPVN

TFSNEVVTLWYRAPDVLLGSRNYSTSIDIWSAGCIMAEMYSGKPLFPGKTNEDQLYKIFKLLGTPTDEIWPRVSELPEYK

PNWPIYQGQVLSSKIPNMDWMGYDLLSKMLVYEPTRRISAKDALNHVYFHDFLQAQYGQPVASPQFLAAGMPTPMMTPAM

MQGMPAMPVQGMPPVGVPVNLAGVQAVGPNGAVLTANNMGMPGMGGVGGVNGLGGMGGNYLS

>SPPG_05640.2 cyclin-dependent kinase 10 isoform 3 (Transcript:SPPG_05640T0) 418 aa

MPGPPVPYQPSRHDTYLTDTPTDPLPVKTVRSLSANRPSATIRQAFTGNSPSVERYEKLNRVGEGTYGIVYRARDKASGT

IVALKRIRMEQEQEGLPLSSLREISLLKSLRHENVVSVLDVVVGNGLEKLKVNSAGQCIPSVLGWLLILSCITVKCLMSQ

LLTGLAYLHDNYIIHRDLKLSNLLLTSSGILKIADFGLARKFGTPVRPMTPKVVTLWYRAPELLFGEKSYTTAIDMWAVG

CIFGELIRAKPLLPGKVEQQQLDLICRLLGTPNARIWPGFDKLPYAKSVKLPSIPYDDIAGQFSEQKESVRKLLKSLLTY

YPSSRLTVHEALRHDYFRESPPACAPVLLPTYPELRTEEYARNPRNNDRKRPRVEDRDVSRVRRSRGQLDPSILTRAREE

EEELELGVPAYQQFRFS*

>SPPG_00051.2 cyclin-dependent kinase C-1 (Transcript:SPPG_00051T0) 534 aa

MGDATIHVDPPAGDDHDESEMEEGELYEPVFVAPTSIPFTSYPGLSPTSHPHSPPTPKFSPSPTDPLYFPCTQNPLNTNL

SKTYVGTCPLEEYDNRERIGKGSFGEVTIATHKETQRKVALKRIIVHKEKDGLPITAVREINILKGLRHKSVIELLGMAV

AKGNPDEYEPATIYMIFPYMHHDLVGLLENRYVTLEPNQIKSFTKQLLEGVAYLHRNHFLHRDLKSSNILIDNEGTLKIA

DFGLARSYNAADKTIELTPNVITLWYRPPELLLGHKTYTSAVDMWGVGCIFAEMWDRKAIFKGETELDLLDKILRVCGTP

DAKAWPEFTKLTASASIRPKQESRKILDIYKPDRLDFQTIDLLNTFLVLNPARRPTAEAALKHEYFHVEPEAAKPSTPDF

PGWPESHELGIRAQIDQELRELDVLQRPIPSIRPPAHHDALIVDGQPVRRPRLHDDAETRQYNRHRDDKRPGRRHDHRRF

GRGRGRPGNERGRGGRGKSAHEASGEPTGEGSVIMRMKHPLPPKPVLRVPFSE*

>SPPG_00975.2 cyclin-dependent kinase C-2 (Transcript:SPPG_00975T0) 1506 aa

MAEETSSAVNDKSAKTNSPDHKETSLESTSPQPDTFKRPAEVSQEDGNGEKLQPKERSSEPAAGVKRPISEVGDEGKPDV

KKIKPDTKQRSPSNELEEGEMSESSTRDTSLPPESNRTSPRPSAHHKSRAAPDVRRPSPPPEFLELFKSPPTPYLSPNFH

DVDVPLSQALSAYTGTSDISEFESSKKVGEGTFGEVTIAKHKSTGRKVALKKIILHKDRDGLPITAVREIAILKSLRHPN

LINLEEIAVRRGDRRAEEVATLFMVFPYMKHDLSGLLDNPQVTFDPSHIKSFTKQMLDGILYLHQRNILHRDMKSANILI

DNGGNLRIADFGLARSFDPSAGRRLTPTVVTLWYRAPELLLGKQDYTAAVDMWGIGCIFAEMWDRRPIFKGATEVELIEK

IFSICGTPDPADYRSSNGKRQGKFPKLEDGTVTPQPQPRRIWDAYSSERLDFQTIAFIDYLLNLDPDRRPTADEALRHDY

FLVEPRAAEPNTSDFPTWEESHEYGMRLRHEQAARAFNERANLSRMIPGLAPLEHLPPPLPVHSDAFRLRSGKVVRRRRL

ADVGMSGRGGDRLDRYNSGIRDERDDWNGDRSSRYGNRADSVGKDERWQKGGHDRYVPGGESGGRDDRTERESHAASSVK

DDRGRRSGHDHYSVTSETSGRGDRERSTKDDRGRGRDVYIPNHEAREERSHRNAKNEEPILKPAQGHRTPSDRQSHRERA

REGRKREEESRDTPEQRAVEKNADEKERDVNGSHGMLDNRYLDRKADDKRKGMYHERHNQKDREVDKMSDEKQKDEEQKH

DKGNSRNGDKVSDPNRKSEGSKHDKREDRGAIDKRPDDKRGDEDHKSSKPDDREGTKTDIGRKSEEHKHDTRDYCRETDK

RSDDKRRDEDHKSSKRDDREGTKADGGRKSEDHKHDKRDDRALVDKKSDHTRTDKEHHNNKRDDREGRRIHDSRRKSDDH

KRDKRDNHMVLDKKSDEKKAAGKVDAKSEEKQRDSVVYDRRSPGVHKPRDKDQTEYKGDEGHSKSDRRSVAASQGQKEDD

SGGQRRDVRNHGTPEKTSEREVKRTESRTGDQGRGDRRDNRHGDADLPISERPKLHRASSGDAKRQSAREQPRGGRESIK

METKSDFLDSNKEEESQRSTTRKEEASEPDIHRSTAKVRISEKKDEPKSSSRDPSTENKLGTRESGSDKRKRDDEPDHPT

KRRSLDNEDLIGKREERKEETKSGTRSDEKRLDDPKSPSVTRKSAKDVKHDTSPRLRETKLEDDAKKIRESKDDGSSRSR

RTSFGDDRKQRESKDHFPSRTRRTGLEEDDNQRKESKDDDSARSRRTSLEDDPTKEKGFNDHRSSRSRRDTLEDDGRRDK

ESKDHHPSPSRRTHLEDEGRRGKDVWRKESSRSSRRTSLESDTHKTTDARREERKWETSSTRETKTDNLSAKGKDSRSMN

QPGSERDKRKDSHDQESLSPSRKPRKSESETPRRMKRSAEEDERSVKRRHPLPPRPKDSPGRRKE*

>SPPG_00440.2 cyclin-dependent kinase 5 (Transcript:SPPG_00440T0) 373 aa

MGGGLDVSLDAGWDMEKYQKIEKLGEGTYGIVYKAQNKDTGDIVALKRIRLDNEEEGVPCTAIREISLLKELKHSNIVRL

YDVIHTEKKLTLVFEYLDSDLKKFLDAYGGDIDVPTLKHLLYQLLKGIAFCHEHRVLHRDLKPQWVDVVTTLVRELLRLD

SHRNLLINKKLELKLADFGLARAFGIPVRSYSHEVCVREGSAGGGSRLTEAGESLAGGNTLVQSSGRPHGFSSIFNVNRF

MVGRMHHGRNGFRWVRSGNLDKSFARCLVDSAAWSGRPLFPGSSIKDQLLRIFKLLGTPDEKSWPKVKELPDYKPDFPIY

PRASLESISSKLDAHGLDLLSKLIEYQPEKRISAERALQHPYFNGIAAKDGE*

>SPPG_07418.2 cell division control protein 2 (Transcript:SPPG_07418T0) 387 aa S. punctatus

MVASGLKLIRHVGTEVRVGKAVHGGPMGMENIPTDVNIRRAGIIAPVKGGVAARDRIQKEIDTVMPDTTDIESLDMRKAE

NMDKYDKIEKVGEGTYGVVYKARDRHSGEIVALKKIRLETEDEGVPSTAIREISLLKELKHPNIVRLLDIVHNDAKLYLI

FEFLDLDLKKYMDTQSNGLSAPLIKSYMYQLIKGIHYCHCHRILHRDLKPQNLLIDQQGMLKLADFGLARAFGVPLRTYT

HEVVTLWYRAPEILLGSKHYSTAVDMWSVGCIFAEMCLRHPLFPGDSEIDEIFRIFRLVGRQKVIRTVTLGTPNEEIWPN

VTTLPDYKENFPIWTAQNLAKVLPNLESEGVDLLSRLLVYDPAQRLSAKRALSHPYFADVDTSKSL*

>AMSG_07969.1 CMGC/CDK/CDK7 protein kinase (Transcript:AMSG_07969T0) 380 aa T. trahens ATCC 50062

MDKYDKLSEAGKGQYGVVYRARRKEDGKTVAIKRINQGDAREGVNFSAVREIRLLQELRHPHVVSLQEVFVHRGGIHLVM

DFCETDLEAIVRDPSLRLGTPEIKAYMKMTLEGLAAMHDTWVLHRDLKPENLLVSATEGVKIADFGMARMYGSPNRRMTH

QVATRWWRAPELLFGARAYGAGVDMWAMGCIFAELYMRAPLFQGETDLDQLSCIFAILGTVNNAVWPGVSELPDFVEFEP

SNGIALGKVVASAPADAVDLMVAMLAYNPASRISATEALQHPYFTNSPPPLPISELPLPESARTRAREAALGFDSDDEAS

GAIGMDTSAASSSTNGKRAVASPVVMAESPAKRAKGLAGGAIEGGDDGEDAGPVKKLMF*

>AMSG_03791.1 CMGC/CDK/CDC2 protein kinase (Transcript:AMSG_03791T0) 288 aa

MEQYMKLEKIGEGTYGVVYKARDLLTNDFIALKKIRLDAEDEGVPSTAIREISLLKELEHPNIVRLHDVVHSDKKLYLVF

EYLDQDLKKYMDSVSGLLKPALVKSYLQQLLEGIAFCHSHRVLHRDLKPQNLLIDRNGVLKLADFGLARAFGIPVRTYTH

EVVTLWYRAPEILLGSRHYSCPVDVWSIGCIFAEMASKIPLFPGDSEIDNLFRIFKILGTPNETIWPGVSALPDFKSSFP

KWQPKNLASVATNLGPDGIDLLSQMLEYEPSRRISAKAALSHPYFLE

>AMSG_11394.1 CMGC/CDK protein kinase (Transcript:AMSG_11394T0) 565 aa

MSTGLTPHPPPPRIRTPPYPVPPVPARLSDPPPPPPPVAAAYPAPPLPPGPPPAAQQPPLPPGPPPPTSSSAAPASAGGK

ALSVPASSQSPTMVASTKPIFTSSKKAAMWGSRSVDVFQKERHVGEGTFGMVYLATDKATGERVALKKVRTESERQGFPI

TAIREIKILKGLNHPNVVSLKEVVMSDLGTVYLVFEYASSDLVGILDNPEIPALTLAQIKTYLKQTLEGLYYVHAQKIMH

RDIKAANLLVHDGVLKLADFGLAKPIDRNADGFTNQVVTLWYRAPEILLGERMYGLPIDIWSVGCIMVELLTGRAFLPGK

DEAHQVDLICRQLGTPTEATWPGCTRLPRFATLVKNKYPPMIARTLKDLQPDAYDLVTKLLSLDPAKRPTAQEALEHKFF

FNKPRPLKPENMPKFTSHELDAKEIRKKMRAEQAVGAKRAAAAAAAGADHPSKRSRNLYNQPRRGDRDRRGGGSYDRGDR

NRDRDRNRDRGPPPGPPRARGPPPGPPAPQQRHHHPGAARRGGPPQRHRHPGFNRQRRPNRPPPKSAAKNGSSGRGGAGP

AAGK*

>AMSG_02688.1 CMGC/CDK/CDK5 protein kinase (Transcript:AMSG_02688T0) 312 aa

MENYTRIAKLGEGTYGVVYKAQNRQTGLIVALKRIRLDSEEQGVPCTAIREIALLKELKHPNVVELLDVLHSDKKLTLVF

EYLDQDLKKYMDGIGGGIEPHIVKTYMYQLLKGLAFCHEHRVLHRDLKPQNLLTSSRHELKLADFGLARAFGLPVRSYST

EVVTLWYRAPDVLLGSSTYSTSIDLWSAGCIFAEMVNGSPLFPGTSVENQLERIFNVVPLPPEDSLGEGVELPVGVGDAC

EPASEAPVLATTLGEVVPGLSAEGVDLLGLLLVADPAGRITATDALTHPYFDNVLDPSASDSCLAGASATD*

>AMSG_04337.1 CMGC/CDK/CRK7 protein kinase (Transcript:AMSG_04337T0) 533 aa

MTAVATAAAVEVLTVAATAVGTVAVTAVATAAATTTVTIIAVGATSAVSGAGGSGDAGGSDRRRGDDWAHRDRDRGRGAY

HDRRGEPRGREHYPRRGEHVDQRGEPIPREYREFHDRRAGMREHHERRSNGDRAYNQTASASSGTGRGTVFSRLAAEEGS

RAPAVPRNNAAAWGSRTIAAFEFGDVDQIGEGTYGQVYRATDKATGDVVALKKIRADRQKREGFPITAIREIKILKHLKH

PNIVNLKEVVISEPEAETVHSSVRGSVYMVFEYVDHDLSGLLESVNAPKMEAGQVKDFMLQLLASVAYTHQMGILHRDIK

AANLLVSKDGVLKLADFGLARQIDQSARYTNKVITLQYRPPELLLGGDSYGPAVDMWSIGCIFVELLTGGRTLFRADKEK

DLMMAIVKVCGTPTEAEWPGLSALPLFHSFNFPRTRRRLRDSLPSIDAQAFDLLDKLFTYDPDKRISAAAAIAHPYFTLH

LSRSKTHKRKRDVPKSQPPAAATTRASVPVSAADADLPPAKRTKPLVAAATE*

>AMSG_09913.1 CMGC/CDK/CDK8 protein kinase (Transcript:AMSG_09913T0) 416 aa

MHDRYVLEAEVGRGTYGKVYKAQMVRGTTPGSEPAPQTSEIVALKEFNGADRVDGIPLSGIREMALLRELKHPSLVTLHD

VVVEPGSGSTFLVLEYVEHDLEKLLIHHAKRKHPLMAETVRSMMWQLVSGIEYMHANWVMHRDMKPSNVLVYGHGPHFGH

IKITDFGMARIFKTPAVALTANNPVVATLWYRAPELLLGARHYTPAIDVWALGVIFAELLFLQVLFKGKQIEEKASDIKT

FQSHQMEAIVNILGTPDPATWPGLADLAHANQLAAFTRVESRLDATLRKLGKLRKPQLLLSSQACDLLKRMVAYNPDDRI

TCADALRHPYFAKLPAPTCDAFAADTKPLRYPLPALKPVTSSSAGPSAPSHPPLPPGPPPPPPPPPPSGLPPPSSSARHR

PVHPNPRALKRHKPN*

>AMSG_04682.1 cmgc/cdk/pitslre protein kinase (Transcript:AMSG_04682T0)Translated sequence:337 aa

MADTRKREASTPPPTLRKSKRARLLKDLYRKVSTIAEGQYGLVFKAVRKDKADAAPVALKKLKLGNVEEGFPVTALRELA

ALRSLESPHVVRLLEVVVGETMDEVYMVLEHHPLDLKVILDAHGAGAFTSAQAKLLVWQLFSGLAFLHASWLIHRDIKPA

NLLYSADGRLVIADFGLVRPFGDPPPAVLTPTVVTLWYRAPELIFGEAAYNTAVDVWSAGAVTVELLTGEPLLRADSETA

YVQKMCLLLGAPSDAVWPGFSLLPGGSKYVLPAQRDSYLDDRLPDGTSLAGRRLISSMLTYDPAARLTASDVVSADWFAE

DPVKAAQWSMPSFRDR*

>Ddi-gi|7579907| gb|AAB35208.2| p34-cdc2 protein [Dictyostelium discoideum AX4] p34-cdc2

MDKYNIEALIGEGTYGVVSRATVKATGQIVAIKKIRKILIQNQTDDGINFSAIREIKILQELKHDNVVNLLDIFAHKSNV

YLVFELMQWDLQEVIEDKSIILKPADIKSYMKMLLQGIEACHRNWVLHRDLKPNNLLMSINGDLKLADFGLARQYGSPNK

VFSPQAVTIFYRAPELLFGAKSYGPSVDIWSIGCIFAELMLRTPYLPGTGEIDQLRKICSALGTPNESNWPGVTCLPNYI

KFTDHPATPFKQLFTAASDEAIDLISKMLLFNPSNRISAADALNHPYFTSGVKHTNPADLPVPFAKKASLLQQRQVLAQV

QQQLLQKQQQQQQQQQQQIQSQPEPIQVDNVEQTQQAQQVKK

>Ddi-gi|66805759| ref|XP_636601.1| cdk5 CDK family protein kinase [ Dictyostelium discoideum AX4 ] Gene ID: 8626776 cdk5

MEKYSKIEKLGEGTYGIVYKAKNRETGEIVALKRIRLDSEDEGVPCTAIREISLLKELKHPNIVRLHDVIHTERKLTLVF

EYLDQDLKKYLDECGGEISKPTIKSFMYQLLKGVAFCHDHRVLHRDLKPQNLLINRKGELKLADFGLARAFGIPVRTYSH

EVVTLWYRAPDVLMGSRKYSTPIDIWSAGCIFAEMASGRPLFPGSGTSDQLFRIFKILGTPNEESWPSITELPEYKTDFP

VHPAHQLSSIVHGLDEKGLNLLSKMLQYDPNQRITAAAALKHPYFDGLEPIN

>Ddi-gi|66823249| ref|XP_644979.1| cdk1 CDC2 subfamily protein kinase [ Dictyostelium discoideum AX4 ] Gene ID: 8618656 cdk1

MESDGGLSRYQKLEKLGEGTYGKVYKAKEKATGRMVALKKIRLEDDGVPSTALREISLLKEVPHPNVVSLFDVLHCQNRL

YLVFEYLDQDLKKYMDSVPALCPQLIKSYLYQLLKGLAYSHGHRILHRDLKPQNLLIDRQGALKLADFGLARAVSIPVRV

YTHEIVTLWYRAPEVLLGSKSYSVPVDMWSVGCIFGEMLNKKPLFSGDCEIDQIFRIFRVLGTPDDSIWPGVTKLPEYVS

TFPNWPGQPYNKIFPRCEPLALDLIAKMLQYEPSKRISAKEALLHPYFGDLDTSFF

>Ddi-gi|66828493| ref|XP_647600.1| cdk8 CDK family protein kinase [ Dictyostelium discoideum AX4 ] Gene ID: 8616412 cdk8

MNTHNQSSNQNGGGSGGGGGGGGSSTFSPIVLTSTYRLDVQEKYTFSYEIGSGTYGMVYKADDKKRPNNKVAVKKFRSTK

EGEGLSLTAYREIGLLKELSNENIVKLLDVCLNPKDKLLYLIFDYAEFDLFGIIKYHRENGSHFSDATIKSLIWQVLNGI

HYLHSNWVIHRDLKPSNILVMGEGKECGTVKIGDFGLARIFQSPLKPLNENGVVVTIWYRSPELLLGSKHYTRAVDIWAI

GCIFAELITTKPLFPGKEKDPKIPSLFQDDQVEKIIRVLGKPTLDMWPDIKHLPEWKRLSSMEAFPNSLAKCVGIDENSQ

AYDLLSKMILYDPSKRITASEALDHPYFKELPLPLPNAFSKPIPYPPRLPINKKKREFDD

>Ddi-gi|66827511| ref|XP_647110.1| cdk10 CDK family protein kinase [ Dictyostelium discoideum AX4 ] Gene ID: 8615914 cdk10

MRSVLSFEKLDSIGEGTYGIVSKGRDKETGRIVALKKVKIGQQDKDGIPLTSLREIQILKEIKHPNIVSLLEVVIGSTGD

KIYLVFEYLEHDVASLIDNINKPFKLSEIKCFLLQLLRAVEYLHSHWIIHRDLKCSNLLYGNNGNLKLADFGLARKFGYP

IESITPCMVTLWYRSPELLLGCQKYSTAVDLWSIGSIFGELLIGRPLITGNNEVDQIMRIFNLLGEPNEQIWPGFSSLPN

FKRLNNIPHQPYNNLRELVPTISDTAFDLLNQLLTYDPTKRITASDAIKHPFFYENPFPQSIEMMPKFPTISKSFKNQNK

KQNNNFNNFVQNNQTNQNNQTNQNNQTNQNNKTSQNNNMDSYKYSK

>Ddi-gi|66822245| ref|XP_644477.1| cdk9-2 CDK family protein kinase [ Dictyostelium discoideum AX4 ] Gene ID: 8619101 cdk9-2

MDTTTNNNIENNTTENIKKEEILEKQQHEAIKEESTTIKEEIIEKPNINNNNNNNNSNNSNNNNNNNNNNSRESTTNNNS

SGNNIINNNNNNINNNINNNNNINNNINNNSNSSSNNNNNNNLPQNGSTHKEESTTSSSSTINSNPNPNPNPNPNPNPNP

NPINKAPTIHGTVITTAMLTGIINSVSNMDNIRYNNSDSVWGSRSVDSHEKIEQIGEGTFGQVYKAKNKSNGDIVALKKV

IMDNEVEGFPITAIREIKILKELNHANVVNLKEVVTSKASASNNHKGSVYMVFEYMDHDLNGLMDSPAFKYFAPGQIKCY

LKQLLEGLDYCHRNNVLHRDIKGSNLLLDNNGILKLADFGLARPFNSSEKKQILTNRVITLWYRPPELLLGTFHYGPEID

MWSVGCIMAELLSKKTLFPGRNSIDQLDKIYQVCGSPNANNWPEAMDLPFWDALKPKREYNSLSLKDFYQHENPSFFTKE

AFDLLDKLLCMDPKKRITASEALDHQYFWTDPMPVNPKDLPQYPSCHEYRTKKRLRQQNQNQNQQQNQQQQQQQPQNQHQ

QSQYQGANVNHHNNHNNNNNQPPPYQNGSGSNNSNNFKKQRTDYNQPPPPPPSSSSTSSSTQQPFNKGPYGQPPQVQPPG

GSFVNGGNNNYPQNRNTLPQQGGGGNRPPQSQQNKNNFPTNNNSSSNSRNPSHR

>Ddi-gi|66810856| ref|XP_639135.1| cdk11 CDK family protein kinase [ Dictyostelium discoideum AX4 ] Gene ID: 8624005 cdk11

MSNIQENENKETCNIKENENKEILKENFKNKEKQYFSSLRSPYSACRSVDCFKKLYTINEGAFGVVYCAQDKETEEIVAL

KKIKMEREREGIPITSVREIKVLMELKHDNIVQIKEIVLGKNINSIFMAMEFIDHDLRGLMEVIKKPFLPSEIKTLIQQL

LNGVSYMHDNWVIHRDLKTANLLYTNKGVLKIADFGLAREYGSPLKPLSKGVVTLWYRAPELLLDTEIYTPAIDIWSVGC

IFAEIISKEVLLQGSSEIDQMDKIFKLFGTPTEKSWPAFFKLPLAKYFNLTDQPYNNLKSKFPHITDNAFDLLNKLLELN

PEARISASDALKHPYFFENPQPRDPLLMPTWPSSHKKT

>Ddi-gi|66816795| ref|XP_642396.1| DDB_G0278487 CDK family protein kinase [ Dictyostelium discoideum AX4 ] CDK9 Gene ID: 8621601 DDB_G0278487

MPSQSNNVITSSTASSSMSSSSNSSDASSTSSSNTNNAHSSNNQFVSGQLNLVGNTHTKINDNYEIISKIGEGISGSVFK

AIKKGTEEMVALKNFKGWTEGDRASKEECSLLQQLRHIPYITPVIDIYTNFETSEYIIVFPYFEHDLSGLLSEHRLSIPQ

VKCYFKQLLEGINEIHNAGVMHRDIKAANLLVNNKGSLFIGDLGTATSYTKRSVFSSKVVTLWYRAPELLLGSTQYGPEI

DMWSIGCVLIELVTSRNFLPGSSEQQQLEAICKLCGTPTDEIWPNVSQLQNFNQISHLPVYPSRLRTVFKNFSNDFIELL

EGLLTLNPKKRLTAEQALQSPFFTNHPLPFKPENMPGYQPIHVLEAVQKRVQQQQELEQQKKQEEQKKQQEEQKKLEDQK

KQEEQKKQEDLLKRQRLLKRQQELKKQQDEQRQQELIKKQQEQEQLRLKVEHEKQRQEQERLRLIQQEQERLKRQQQEHE

QRLQREQQQQLNQLQQQKESPLNNSYGKINLKRSLDLVNDIRNYCTSETESEYESDEEDFYTEEEVEDYSSDEEDDYYNA

QSNKSLYTPIKAMIQQHNNNQQHQQQYPYSQQQQEPISSNPFLQPPKKQRTASTGFNNNNGNNNNNNNLSTPLTIH
