# Supplementary material for: Phylogenetic analysis of CDK and cyclin proteins in premetazoan lineages
Source: BMC Evol Biol. 2014 Jan 17;14:10. doi: 10.1186/1471-2148-14-10 (PMC3923393; doi:10.1186/1471-2148-14-10)
Supplement: Additional file 3: File S3 — Cyclin sequences from 18 organisms. [file 1471-2148-14-10-S3.doc]

**File S3. Cyclin sequences from 18 organisms.**

>Hsa-CyclinA1 gi|4502611|ref|NP_003905.1| cyclin-A1 isoform a [Homo sapiens] Gene ID 8900 CCNA1

METGFPAIMYPGSFIGGWGEEYLSWEGPGLPDFVFQQQPVESEAMHCSNPKSGVVLATVARGPDACQILT

RAPLGQDPPQRTVLGLLTANGQYRRTCGQGITRIRCYSGSENAFPPAGKKALPDCGVQEPPKQGFDIYMD

ELEQGDRDSCSVREGMAFEDVYEVDTGTLKSDLHFLLDFNTVSPMLVDSSLLSQSEDISSLGTDVINVTE

YAEEIYQYLREAEIRHRPKAHYMKKQPDITEGMRTILVDWLVEVGEEYKLRAETLYLAVNFLDRFLSCMS

VLRGKLQLVGTAAMLLASKYEEIYPPEVDEFVYITDDTYTKRQLLKMEHLLLKVLAFDLTVPTTNQFLLQ

YLRRQGVCVRTENLAKYVAELSLLEADPFLKYLPSLIAAAAFCLANYTVNKHFWPETLAAFTGYSLSEIV

PCLSELHKAYLDIPHRPQQAIREKYKASKYLCVSLMEPPAVLLLQ

>Hsa-CyclinA2 gi|4502613|ref|NP_001228.1| cyclin-A2 [Homo sapiens] Gene ID: 890 CCNA2

MLGNSAPGPATREAGSALLALQQTALQEDQENINPEKAAPVQQPRTRAALAVLKSGNPRGLAQQQRPKTR

RVAPLKDLPVNDEHVTVPPWKANSKQPAFTIHVDEAEKEAQKKPAESQKIEREDALAFNSAISLPGPRKP

LVPLDYPMDGSFESPHTMDMSIVLEDEKPVSVNEVPDYHEDIHTYLREMEVKCKPKVGYMKKQPDITNSM

RAILVDWLVEVGEEYKLQNETLHLAVNYIDRFLSSMSVLRGKLQLVGTAAMLLASKFEEIYPPEVAEFVY

ITDDTYTKKQVLRMEHLVLKVLTFDLAAPTVNQFLTQYFLHQQPANCKVESLAMFLGELSLIDADPYLKY

LPSVIAGAAFHLALYTVTGQSWPESLIRKTGYTLESLKPCLMDLHQTYLKAPQHAQQSIREKYKNSKYHG

VSLLNPPETLNL

>Hsa-CyclinB2 gi|4757930|ref|NP_004692.1| G2/mitotic-specific cyclin-B2 [Homo sapiens] Gene ID: 9133 CCNB2

MALLRRPTVSSDLENIDTGVNSKVKSHVTIRRTVLEEIGNRVTTRAAQVAKKAQNTKVPVQPTKTTNVNK

QLKPTASVKPVQMEKLAPKGPSPTPEDVSMKEENLCQAFSDALLCKIEDIDNEDWENPQLCSDYVKDIYQ

YLRQLEVLQSINPHFLDGRDINGRMRAILVDWLVQVHSKFRLLQETLYMCVGIMDRFLQVQPVSRKKLQL

VGITALLLASKYEEMFSPNIEDFVYITDNAYTSSQIREMETLILKELKFELGRPLPLHFLRRASKAGEVD

VEQHTLAKYLMELTLIDYDMVHYHPSKVAAAASCLSQKVLGQGKWNLKQQYYTGYTENEVLEVMQHMAKN

VVKVNENLTKFIAIKNKYASSKLLKISMIPQLNSKAVKDLASPLIGRS

>Hsa-CyclinB1 gi|14327896|ref|NP_114172.1| G2/mitotic-specific cyclin-B1 [Homo sapiens] Gene ID: 891 CCNB1

MALRVTRNSKINAENKAKINMAGAKRVPTAPAATSKPGLRPRTALGDIGNKVSEQLQAKMPMKKEAKPSA

TGKVIDKKLPKPLEKVPMLVPVPVSEPVPEPEPEPEPEPVKEEKLSPEPILVDTASPSPMETSGCAPAEE

DLCQAFSDVILAVNDVDAEDGADPNLCSEYVKDIYAYLRQLEEEQAVRPKYLLGREVTGNMRAILIDWLV

QVQMKFRLLQETMYMTVSIIDRFMQNNCVPKKMLQLVGVTAMFIASKYEEMYPPEIGDFAFVTDNTYTKH

QIRQMEMKILRALNFGLGRPLPLHFLRRASKIGEVDVEQHTLAKYLMELTMLDYDMVHFPPSQIAAGAFC

LALKILDNGEWTPTLQHYLSYTEESLLPVMQHLAKNVVMVNQGLTKHMTVKNKYATSKHAKISTLPQLNS

ALVQDLAKAVAKV

>Hsa-CyclinD3 gi|4502619|ref|NP_001751.1| G1/S-specific cyclin-D3 isoform 2 Gene ID: 896 CCND3

MELLCCEGTRHAPRAGPDPRLLGDQRVLQSLLRLEERYVPRASYFQCVQREIKPHMRKMLAYWMLEVCEE

QRCEEEVFPLAMNYLDRYLSCVPTRKAQLQLLGAVCMLLASKLRETTPLTIEKLCIYTDHAVSPRQLRDW

EVLVLGKLKWDLAAVIAHDFLAFILHRLSLPRDRQALVKKHAQTFLALCATDYTFAMYPPSMIATGSIGA

AVQGLGACSMSGDELTELLAGITGTEVDCLRACQEQIEAALRESLREASQTSSSPAPKAPRGSSSQGPSQ

TSTPTDVTAIHL

>Hsa-CyclinB3 gi|90669307|ref|NP_149020.2| G2/mitotic-specific cyclin-B3 isoform 3 [Homo sapiens] Gene ID: 85417 CCNB3

MLLPLPPQSSKPVPKKSQSSKIVPSHHDPSEKTGENCQTKISPSSLQESPSSLQGALKKRSAFEDLTNAS

QCQPVQPKKEANKEFVKVVSKKINRNTHALGLAKKNKRNLKWHKLEVTPVVASTTVVPNIMEKPLILDIS

TTSKTPNTEEASLFRKPLVLKEEPTIEDETLINKSLSLKKCSNHEEVSLLEKLQPLQEESDSDDAFVIEP

MTFKKTHKTEEAAITKKTLSLKKKMCASQRKQSCQEESLAVQDVNMEEDSFFMESMSFKKKPKTEESIPT

HKLSSLKKKCTIYGKICHFRKPPVLQTTICGAMSSIKKPTTEKETLFQELSVLQEKHTTEHEMSILKKSL

ALQKTNFKEDSLVKESLAFKKKPSTEEAIMMPVILKEQCMTEGKRSRLKPLVLQEITSGEKSLIMKPLSI

KEKPSTEKESFSQEPSALQKKHTTQEEVSILKEPSSLLKSPTEESPFDEALAFTKKCTIEEAPPTKKPLI

LKRKHATQGTMSHLKKPLILQTTSGEKSLIKEPLPFKEEKVSLKKKCTTQEMMSICPELLDFQDMIGEDK

NSFFMEPMSFRKNPTTEETVLTKTSLSLQEKKITQGKMSHLKKPLVLQKITSEEESFYKKLLPFKMKSTT

EEKFLSQEPSALKEKHTTLQEVSLSKESLAIQEKATTEEEFSQELFSLHVKHTNKSGSLFQEALVLQEKT

DAEEDSLKNLLALQEKSTMEEESLINKLLALKEELSAEAATNIQTQLSLKKKSTSHGKVFFLKKQLALNE

TINEEEFLNKQPLALEGYPSIAEGETLFKKLLAMQEEPSIEKEAVLKEPTIDTEAHFKEPLALQEEPSTE

KEAVLKEPSVDTEAHFKETLALQEKPSIEQEALFKRHSALWEKPSTEKETIFKESLDLQEKPSIKKETLL

KKPLALKMSTINEAVLFEDMIALNEKPTTGKELSFKEPLALQESPTYKEDTFLKTLLVPQVGTSPNVSST

APESITSKSSIATMTSVGKSGTINEAFLFEDMITLNEKPTTGKELSFKEPLALQESPTCKEDTFLETFLI

PQIGTSPYVFSTTPESITEKSSIATMTSVGKSRTTTESSACESASDKPVSPQAKGTPKEITPREDIDEDS

SDPSFNPMYAKEIFSYMKEREEQFILTDYMNRQIEITSDMRAILVDWLVEVQVSFEMTHETLYLAVKLVD

LYLMKAVCKKDKLQLLGATAFMIAAKFEEHNSPRVDDFVYICDDNYQRSEVLSMEINILNVLKCDINIPI

AYHFLRRYARCIHTNMKTLTLSRYICEMTLQEYHYVQEKASKLAAASLLLALYMKKLGYWVPFLEHYSGY

SISELHPLVRQLNKLLTFSSYDSLKAVYYKYSHPVFFEVAKIPALDMLKLEEILNCDCEAQGLVL

>Hsa-CyclinD2 gi|4502617|ref|NP_001750.1| G1/S-specific cyclin-D2 [Homo sapiens] Gene ID: 894 CCND2

MELLCHEVDPVRRAVRDRNLLRDDRVLQNLLTIEERYLPQCSYFKCVQKDIQPYMRRMVATWMLEVCEEQ

KCEEEVFPLAMNYLDRFLAGVPTPKSHLQLLGAVCMFLASKLKETSPLTAEKLCIYTDNSIKPQELLEWE

LVVLGKLKWNLAAVTPHDFIEHILRKLPQQREKLSLIRKHAQTFIALCATDFKFAMYPPSMIATGSVGAA

ICGLQQDEEVSSLTCDALTELLAKITNTDVDCLKACQEQIEAVLLNSLQQYRQDQRDGSKSEDELDQAST

PTDVRDIDL

>Hsa-CyclinE1 gi|17318559|ref|NP_001229.1| G1/S-specific cyclin-E1 isoform 1 [Homo sapiens] Gene ID: 898 CCNE1

MPRERRERDAKERDTMKEDGGAEFSARSRKRKANVTVFLQDPDEEMAKIDRTARDQCGSQPWDNNAVCAD

PCSLIPTPDKEDDDRVYPNSTCKPRIIAPSRGSPLPVLSWANREEVWKIMLNKEKTYLRDQHFLEQHPLL

QPKMRAILLDWLMEVCEVYKLHRETFYLAQDFFDRYMATQENVVKTLLQLIGISSLFIAAKLEEIYPPKL

HQFAYVTDGACSGDEILTMELMIMKALKWRLSPLTIVSWLNVYMQVAYLNDLHEVLLPQYPQQIFIQIAE

LLDLCVLDVDCLEFPYGILAASALYHFSSSELMQKVSGYQWCDIENCVKWMVPFAMVIRETGSSKLKHFR

GVADEDAHNIQTHRDSLDLLDKARAKKAMLSEQNRASPLPSGLLTPPQSGKKQSSGPEMA

>Hsa-CyclinE2 gi|17318565|ref|NP_477097.1| G1/S-specific cyclin-E2 [Homo sapiens] Gene ID: 9134 CCNE2

MSRRSSRLQAKQQPQPSQTESPQEAQIIQAKKRKTTQDVKKRREEVTKKHQYEIRNCWPPVLSGGISPCI

IIETPHKEIGTSDFSRFTNYRFKNLFINPSPLPDLSWGCSKEVWLNMLKKESRYVHDKHFEVLHSDLEPQ

MRSILLDWLLEVCEVYTLHRETFYLAQDFFDRFMLTQKDINKNMLQLIGITSLFIASKLEEIYAPKLQEF

AYVTDGACSEEDILRMELIILKALKWELCPVTIISWLNLFLQVDALKDAPKVLLPQYSQETFIQIAQLLD

LCILAIDSLEFQYRILTAAALCHFTSIEVVKKASGLEWDSISECVDWMVPFVNVVKSTSPVKLKTFKKIP

MEDRHNIQTHTNYLAMLEEVNYINTFRKGGQLSPVCNGGIMTPPKSTEKPPGKH

>Hsa-CyclinK gi|150417989|ref|NP_001092872.1| cyclin-K [Homo sapiens] Gene ID: 8812 CCNK

MKENKENSSPSVTSANLDHTKPCWYWDKKDLAHTPSQLEGLDPATEARYRREGARFIFDVGTRLGLHYDT

LATGIIYFHRFYMFHSFKQFPRYVTGACCLFLAGKVEETPKKCKDIIKTARSLLNDVQFGQFGDDPKEEV

MVLERILLQTIKFDLQVEHPYQFLLKYAKQLKGDKNKIQKLVQMAWTFVNDSLCTTLSLQWEPEIIAVAV

MYLAGRLCKFEIQEWTSKPMYRRWWEQFVQDVPVDVLEDICHQILDLYSQGKQQMPHHTPHQLQQPPSLQ

PTPQVPQVQQSQPSQSSEPSQPQQKDPQQPAQQQQPAQQPKKPSPQPSSPRQVKRAVVVSPKEENKAAEP

PPPKIPKIETTHPPLPPAHPPPDRKPPLAAALGEAEPPGPVDATDLPKVQIPPPAHPAPVHQPPPLPHRP

PPPPPSSYMTGMSTTSSYMSGEGYQSLQSMMKTEGPSYGALPPAYGPPAHLPYHPHVYPPNPPPPPVPPP

PASFPPPAIPPPTPGYPPPPPTYNPNFPPPPPRLPPTHAVPPHPPPGLGLPPASYPPPAVPPGGQPPVPP

PIPPPGMPPVGGLGRAAWMR

>Hsa-CyclinJ gi|197276674|ref|NP_001127847.1| cyclin-J isoform 1 [Homo sapiens] Gene ID: 54619 CCNJ

MELEGQWWRGQLAADIHQALRYKELKLPSYKGQSPQLSLRRYFADLIAIVSNRFTLCPSARHLAVYLLDL

FMDRYDISIQQLHLVALSCLLLASKFEEKEDSVPKLEQLNSLGCMTNMNLVLTKQNLLHMELLLLETFQW

NLCLPTAAHFIEYYLSEAVHETDLHDGWPMICLEKTKLYMAKYADYFLEVSLQDYAFLNYAPSLVAAACV

ASSRIILRLSPTWPTRLHRLTAYSWDFLVQCIERLLIAHDNDVKEANKQRGQAGPQSAQLSVFQTASQPS

RPVHFQQPQYLHQTHQTSLQYRHPTSEQPSCQQIVSTTHTSSYTLQTCPAGFQTSVQGLGHMQTGVGMSL

AIPVEVKPCLSVSYNRSYQINEHYPCITPCFER

>Hsa-CyclinF gi|118572588|ref|NP_001752.2| cyclin-F [Homo sapiens] Gene ID: 899 CCNF

MGSGGVVHCRCAKCFCYPTKRRIRRRPRNLTILSLPEDVLFHILKWLSVEDILAVRAVHSQLKDLVDNHA

SVWACASFQELWPSPGNLKLFERAAEKGNFEAAVKLGIAYLYNEGLSVSDEARAEVNGLKASRFFSLAER

LNVGAAPFIWLFIRPPWSVSGSCCKAVVHESLRAECQLQRTHKASILHCLGRVLSLFEDEEKQQQAHDLF

EEAAHQGCLTSSYLLWESDRRTDVSDPGRCLHSFRKLRDYAAKGCWEAQLSLAKACANANQLGLEVRASS

EIVCQLFQASQAVSKQQVFSVQKGLNDTMRYILIDWLVEVATMKDFTSLCLHLTVECVDRYLRRRLVPRY

RLQLLGIACMVICTRFISKEILTIREAVWLTDNTYKYEDLVRMMGEIVSALEGKIRVPTVVDYKEVLLTL

VPVELRTQHLCSFLCELSLLHTSLSAYAPARLAAAALLLARLTHGQTQPWTTQLWDLTGFSYEDLIPCVL

SLHKKCFHDDAPKDYRQVSLTAVKQRFEDKRYGEISQEEVLSYSQLCAALGVTQDSPDPPTFLSTGEIHA

FLSSPSGRRTKRKRENSLQEDRGSFVTTPTAELSSQEETLLGSFLDWSLDCCSGYEGDQESEGEKEGDVT

APSGILDVTVVYLNPEQHCCQESSDEEACPEDKGPQDPQALALDTQIPATPGPKPLVRTSREPGKDVTTS

GYSSVSTASPTSSVDGGLGALPQPTSVLSLDSDSHTQPCHHQARKSCLQCRPPSPPESSVPQQQVKRINL

CIHSEEEDMNLGLVRL

>Hsa-CyclinO gi|153791755|ref|NP_066970.3| cyclin-O [Homo sapiens] Gene ID: 10309 CCNO

MVTPCPTSPSSPAARAGRRDNDQNLRAPVKKSRRPRLRRKQPLHPLNPCPLPGDSGICDLFESPSSGSDG

AESPSAARGGSPLPGPAQPVAQLDLQTFRDYGQSCYAFRKAQESHFHPREALARQPQVTAESRCKLLSWL

IPVHRQFGLSFESLCLTVNTLDRFLTTTPVAADCFQLLGVTSLLIACKQVEVHPPRVKQLLALCCGAFSR

QQLCNLECIVLHKLHFTLGAPTISFFLEHFTHARVEAGQAEASEALEAQALARGVAELSLADYAFTSYSP

SLLAICCLALADRMLRVSRPVDLRLGDHPEAALEDCMGKLQLLVAINSTSLTHMLPVQICEKCSLPPSSK

>Hsa-CyclinD1 gi|16950655|ref|NP_444284.1| G1/S-specific cyclin-D1 [Homo sapiens] Gene ID: 595 CCND1

MEHQLLCCEVETIRRAYPDANLLNDRVLRAMLKAEETCAPSVSYFKCVQKEVLPSMRKIVATWMLEVCEE

QKCEEEVFPLAMNYLDRFLSLEPVKKSRLQLLGATCMFVASKMKETIPLTAEKLCIYTDNSIRPEELLQM

ELLLVNKLKWNLAAMTPHDFIEHFLSKMPEAEENKQIIRKHAQTFVALCATDVKFISNPPSMVAAGSVVA

AVQGLNLRSPNNFLSYYRLTRFLSRVIKCDPDCLRACQEQIEALLESSLRQAQQNMDPKAAEEEEEEEEE

VDLACTPTDVRDVDI

>Hsa-CyclinT2 gi|4502629|ref|NP_001232.1| cyclin-T2 isoform a [Homo sapiens] Gene ID: 905 CCNT2

MASGRGASSRWFFTREQLENTPSRRCGVEADKELSCRQQAANLIQEMGQRLNVSQLTINTAIVYMHRFYM

HHSFTKFNKNIISSTALFLAAKVEEQARKLEHVIKVAHACLHPLEPLLDTKCDAYLQQTQELVILETIML

QTLGFEITIEHPHTDVVKCTQLVRASKDLAQTSYFMATNSLHLTTFCLQYKPTVIACVCIHLACKWSNWE

IPVSTDGKHWWEYVDPTVTLELLDELTHEFLQILEKTPNRLKKIRNWRANQAARKPKVDGQVSETPLLGS

SLVQNSILVDSVTGVPTNPSFQKPSTSAFPAPVPLNSGNISVQDSHTSDNLSMLATGMPSTSYGLSSHQE

WPQHQDSARTEQLYSQKQETSLSGSQYNINFQQGPSISLHSGLHHRPDKISDHSSVKQEYTHKAGSSKHH

GPISTTPGIIPQKMSLDKYREKRKLETLDLDVRDHYIAAQVEQQHKQGQSQAASSSSVTSPIKMKIPIAN

TEKYMADKKEKSGSLKLRIPIPPTDKSASKEELKMKIKVSSSERHSSSDEGSGKSKHSSPHISRDHKEKH

KEHPSSRHHTSSHKHSHSHSGSSSGGSKHSADGIPPTVLRSPVGLSSDGISSSSSSSRKRLHVNDASHNH

HSKMSKSSKSSGGLRTSQHPRETGQEASGDQRS

>Hsa-CyclinL2 gi|88758580|ref|NP_112199.2| cyclin-L2 isoform A [Homo sapiens] Gene ID: 81669 CCNL2

MAAAAAAAGAAGSAAPAAAAGAPGSGGAPSGSQGVLIGDRLYSGVLITLENCLLPDDKLRFTPSMSSGLD

TDTETDLRVVGCELIQAAGILLRLPQVAMATGQVLFQRFFYTKSFVKHSMEHVSMACVHLASKIEEAPRR

IRDVINVFHRLRQLRDKKKPVPLLLDQDYVNLKNQIIKAERRVLKELGFCVHVKHPHKIIVMYLQVLECE

RNQHLVQTSWNYMNDSLRTDVFVRFQPESIACACIYLAARTLEIPLPNRPHWFLLFGATEEEIQEICLKI

LQLYARKKVDLTHLEGEVEKRKHAIEEAKAQARGLLPGGTQVLDGTSGFSPAPKLVESPKEGKGSKPSPL

SVKNTKRRLEGAKKAKADSPVNGLPKGRESRSRSRSREQSYSRSPSRSASPKRRKSDSGSTSGGSKSQSR

SRSRSDSPPRQAPRSAPYKGSEIRGSRKSKDCKYPQKPHKSRSRSSSRSRSRSRERADNPGKYKKKSHYY

RDQRRERSRSYERTGRRYERDHPGHSRHRR

>Hsa-CyclinG2 gi|4757936|ref|NP_004345.1| cyclin-G2 [Homo sapiens] Gene ID: 901 CCNG2

MKDLGAEHLAGHEGVQLLGLLNVYLEQEERFQPREKGLSLIEATPENDNTLCPGLRNAKVEDLRSLANFF

GSCTETFVLAVNILDRFLALMKVKPKHLSCIGVCSFLLAARIVEEDCNIPSTHDVIRISQCKCTASDIKR

MEKIISEKLHYELEATTALNFLHLYHTIILCHTSERKEILSLDKLEAQLKACNCRLIFSKAKPSVLALCL

LNLEVETLKSVELLEILLLVKKHSKINDTEFFYWRELVSKCLAEYSSPECCKPDLKKLVWIVSRRTAQNL

HNSYYSVPELPTIPEGGCFDESESEDSCEDMSCGEESLSSSPPSDQECTFFFNFKVAQTLCFPS

>Hsa-CyclinT1 gi|17978466|ref|NP_001231.2| cyclin-T1 [Homo sapiens] Gene ID: 904 CCNT1

MEGERKNNNKRWYFTREQLENSPSRRFGVDPDKELSYRQQAANLLQDMGQRLNVSQLTINTAIVYMHRFY

MIQSFTQFPGNSVAPAALFLAAKVEEQPKKLEHVIKVAHTCLHPQESLPDTRSEAYLQQVQDLVILESII

LQTLGFELTIDHPHTHVVKCTQLVRASKDLAQTSYFMATNSLHLTTFSLQYTPPVVACVCIHLACKWSNW

EIPVSTDGKHWWEYVDATVTLELLDELTHEFLQILEKTPNRLKRIWNWRACEAAKKTKADDRGTDEKTSE

QTILNMISQSSSDTTIAGLMSMSTSTTSAVPSLPVSEESSSNLTSVEMLPGKRWLSSQPSFKLEPTQGHR

TSENLALTGVDHSLPQDGSNAFISQKQNSKSVPSAKVSLKEYRAKHAEELAAQKRQLENMEANVKSQYAY

AAQNLLSHHDSHSSVILKMPIEGSENPERPFLEKADKTALKMRIPVAGGDKAASSKPEEIKMRIKVHAAA

DKHNSVEDSVTKSREHKEKHKTHPSNHHHHHNHHSHKHSHSQLPVGTGNKRPGDPKHSSQTSNLAHKTYS

LSSSFSSSSSTRKRGPSEETGGAVFDHPAKIAKSTKSSSLNFSFPSLPTMGQMPGHSSDTSGLSFSQPSC

KTRVPHSKLDKGPTGANGHNTTQTIDYQDTVNMLHSLLSAQGVQPTQPTAFEFVRPYSDYLNPRSGGISS

RSGNTDKPRPPPLPSEPPPPLPPLPK

>Hsa-CyclinL1 gi|9945320|ref|NP_064703.1| cyclin-L1 [Homo sapiens] Gene ID: 57018 CCNL1

MASGPHSTATAAAAASSAAPSAGGSSSGTTTTTTTTTGGILIGDRLYSEVSLTIDHSLIPEERLSPTPSM

QDGLDLPSETDLRILGCELIQAAGILLRLPQVAMATGQVLFHRFFYSKSFVKHSFEIVAMACINLASKIE

EAPRRIRDVINVFHHLRQLRGKRTPSPLILDQNYINTKNQVIKAERRVLKELGFCVHVKHPHKIIVMYLQ

VLECERNQTLVQTAWNYMNDSLRTNVFVRFQPETIACACIYLAARALQIPLPTRPHWFLLFGTTEEEIQE

ICIETLRLYTRKKPNYELLEKEVEKRKVALQEAKLKAKGLNPDGTPALSTLGGFSPASKPSSPREVKAEE

KSPISINVKTVKKEPEDRQQASKSPYNGVRKDSKRSRNSRSASRSRSRTRSRSRSHTPRRHYNNRRSRSG

TYSSRSRSRSRSHSESPRRHHNHGSPHLKAKHTRDDLKSSNRHGHKRKKSRSRSQSKSRDHSDAAKKHRH

ERGHHRDRRERSRSFERSHKSKHHGGSRSGHGRHRR

>Hsa-CABLES1 gi|154759423|ref|NP_001094089.1| CDK5 and ABL1 enzyme substrate 1 isoform 2 [Homo sapiens] Gene ID: 91768 CABLES1

MAAAAAAATTAACSSGSAGTDAAGASGLQQPPPQPQPQPAAAAPAQPPPEPPRKPRMDPRRRQAALSFLT

NISLDGRLPPQDAEWGGGEEGGAAKPGAGGACGARTRFSLLAAAERGGCIALAAPGTPAAGLAAGSGPCL

PQPSSLPPLIPGGHATVSGPGVARGFASPLGAGRASGEQWQPPRPAPLAACAQLQLLDGSGAAGQEELEE

DDAFISVQVPAAAFLGSGTPGSGSGSRGRLNSFTQGILPIAFSRPTSQNYCSLEQPGQGGSTSAFEQLQR

SRRRLISQRSSLETLEDIEENAPLRRCRTLSGSPRPKNFKKIHFIKNMRQHDTRNGRIVLISGRRSFCSI

FSVLPYRDSTQVGDLKLDGGRQSTGAVSLKEIIGLEGVELGADGKTVSYTQFLLPTNAFGARRNTIDSTS

SFSQFRNLSHRSLSIGRASGTQGSLDTGSDLGDFMDYDPNLLDDPQWPCGKHKRVLIFPSYMTTVIDYVK

PSDLKKDMNETFKEKFPHIKLTLSKIRSLKREMRKLAQEDCGLEEPTVAMAFVYFEKLALKGKLNKQNRK

LCAGACVLLAAKIGSDLKKHEVKHLIDKLEEKFRLNRRELIAFEFPVLVALEFALHLPEHEVMPHYRRLV

QSS

>Hsa-CyclinC gi|61676091|ref|NP_005181.2| cyclin-C isoform a [Homo sapiens] Gene ID: 892 CCNC

MAGNFWQSSHYLQWILDKQDLLKERQKDLKFLSEEEYWKLQIFFTNVIQALGEHLKLRQQVIATATVYFK

RFYARYSLKSIDPVLMAPTCVFLASKVEEFGVVSNTRLIAAATSVLKTRFSYAFPKEFPYRMNHILECEF

YLLELMDCCLIVYHPYRPLLQYVQDMGQEDMLLPLAWRIVNDTYRTDLCLLYPPFMIALACLHVACVVQQ

KDARQWFAELSVDMEKILEIIRVILKLYEQWKNFDERKEMATILSKMPKPKPPPNSEGEQGPNGSQNSSY

SQS

>Hsa-CyclinG1 gi|4757934|ref|NP_004051.1| cyclin-G1 [Homo sapiens] Gene ID: 900 CCNG1

MIEVLTTTDSQKLLHQLNALLEQESRCQPKVCGLRLIESAHDNGLRMTARLRDFEVKDLLSLTQFFGFDT

ETFSLAVNLLDRFLSKMKVQPKHLGCVGLSCFYLAVKSIEEERNVPLATDLIRISQYRFTVSDLMRMEKI

VLEKVCWKVKATTAFQFLQLYYSLLQENLPLERRNSINFERLEAQLKACHCRIIFSKAKPSVLALSIIAL

EIQAQKCVELTEGIECLQKHSKINGRDLTFWQELVSKCLTEYSSNKCSKPNVQKLKWIVSGRTARQLKHS

YYRITHLPTIPEMVP

>Hsa-CyclinH gi|4502623|ref|NP_001230.1| cyclin-H isoform 1 [Homo sapiens] Gene ID: 902 CCNH

MYHNSSQKRHWTFSSEEQLARLRADANRKFRCKAVANGKVLPNDPVFLEPHEEMTLCKYYEKRLLEFCSV

FKPAMPRSVVGTACMYFKRFYLNNSVMEYHPRIIMLTCAFLACKVDEFNVSSPQFVGNLRESPLGQEKAL

EQILEYELLLIQQLNFHLIVHNPYRPFEGFLIDLKTRYPILENPEILRKTADDFLNRIALTDAYLLYTPS

QIALTAILSSASRAGITMESYLSESLMLKENRTCLSQLLDIMKSMRNLVKKYEPPRSEEVAVLKQKLERC

HSAELALNVITKKRKGYEDDDYVSKKSKHEEEEWTDDDLVESL

>Hsa-CyclinI gi|5802992|ref|NP_006826.1| cyclin-I [Homo sapiens] Gene ID: 10983 CCNI

MKFPGPLENQRLSFLLEKAITREAQMWKVNVRKMPSNQNVSPSQRDEVIQWLAKLKYQFNLYPETFALAS

SLLDRFLATVKAHPKYLSCIAISCFFLAAKTVEEDERIPVLKVLARDSFCGCSSSEILRMERIILDKLNW

DLHTATPLDFLHIFHAIAVSTRPQLLFSLPKLSPSQHLAVLTKQLLHCMACNQLLQFRGSMLALAMVSLE

MEKLIPDWLSLTIELLQKAQMDSSQLIHCRELVAHHLSTLQSSLPLNSVYVYRPLKHTLVTCDKGVFRLH

PSSVPGPDFSKDNSKPEVPVRGTAAFYHHLPAASGCKQTSTKRKVEEMEVDDFYDGIKRLYNEDNVSENV

GSVCGTDLSRQEGHASPCPPLQPVSVM

>Hsa-Fam58A gi|196049382|ref|NP_689487.2| cyclin-related protein FAM58A isoform 1 [Homo sapiens] Gene ID: 92002 FAM58A

MEAPEGGGGGPAARGPEGQPAPEARVHFRVARFIMEAGVKLGMRSIPIATACTIYHKFFCETNLDAYDPY

LIAMSSIYLAGKVEEQHLRTRDIINVSNRYFNPSGEPLELDSRFWELRDSIVQCELLMLRVLRFQVSFQH

PHKYLLHYLVSLQNWLNRHSWQRTPVAVTAWALLRDSYHGALCLRFQAQHIAVAVLYLALQVYGVEVPAE

VEAEKPWWQVFNDDLTKPIIDNIVSDLIQIYTMDTEIP

>Hsa-CABLES2 gi|145275195|ref|NP_112492.2| CDK5 and ABL1 enzyme substrate 2 [Homo sapiens] Gene ID: 81928 CABLES2

MAAAAAGGAPGPAPGPAGPPPPAAPTSAARAPPQALRRRGDSRRRQAALFFLNNISLDGRPPSLGPGGEK

PPPPPAEAREPPAPPPPEPPTGLPARTPAPQGLLSPTQVPTGLGLDGQRQRKRVTSQRCSLEFLEDAVGC

APAQRTKHTSGSPRHKGLKKTHFIKNMRQYDTRNSRIVLICAKRSLCAAFSVLPYGEGLRISDLRVDSQK

QRHPSGGVSVSSEMVFELEGVELGADGKVVSYAKFLYPTNALVTHKSDSHGLLPTPRPSVPRTLPGSRHK

PAPTKSAPASTELGSDVGDTLEYNPNLLDDPQWPCGKHKRVLIFASYMTTVIEYVKPSDLKKDMNETFRE

KFPHVKLTLSKIRSLKREMRSLSEECSLEPVTVAMAYVYFEKLVLQGKLSKQNRKLCAGACVLLAAKISS

DLRKSGVTQLIDKLEERFRFNRRDLIGFEFTVLVALELALYLPENQVLPHYRRLTQQF

>Hsa-Fam58B gi|157502165|ref|NP_001098987.1| cyclin-related protein FAM58B [Homo sapiens] Gene ID: 339521 FAM58BP

MEGMEDAGEEAGEDAGEDAREGAAAPAARVHFRVARFIMEAGVKLGMQSIPIATACTIYPKFFCETILDA

FDPYLIAMSSIYLAGKVEEQPLWAHDIISVSNRYFNPSSEPLGLDSRLWELRDSIVQRELLMLRVLRFQV

SFQHPHKYLLYYLVSLKNWLNCHSWQRTPVAVTAWALLRDSYHGGLCLRFQAQHIAVVVLYLALQVYGVE

VPAEVEAEKLWWQAFSDDLTKPIIDTIVSDLIQIYTIDTEIP

>Hsa-CyclinI2 gi|89886237|ref|NP_001034869.1| cyclin-I2 [Homo sapiens] Gene ID: 645121 CCNI2

MASGAQLPPQPSSSEVSAVQSPGGRPGAGLEETALGVPLPPSPGEAPLPRSNRSRCPGTRQPGAASLHAA

SAAVPVRPRRGTAPAGKTADAVPAAAPEQAPRPAPQSRKPRNLEGDLDERRLLCHLQLAQDREARLWRGG

KPQDEICDAFEEVVLWLLRLQNTFYFSQSTFNLALTIFGRLLISVKVKEKYLHCATITSLRLAAKVNEEE

EFIPQVKDFTKHYGSDYSPNELLRMELAILDRLHWDLYIGTPLDFLTIFHALVVLSWPHVLELLPQRNPS

LHVASLTRQLQHCMAGHQLLQFKGSTLALVIITLELERLMPGWCAPISDLLKKAQVGDMQYSCCKELVMQ

QLRSLQSSSCTDNFVSPAN

>Hsa-CyclinJL gi|94680985|ref|NP_078841.3| cyclin-J-like protein [Homo sapiens] Gene ID: 79616 CCNJL

MMDEPWWEGRVASDVHCTLREKELKLPTFRAHSPLLKSRRFFVDILTLLSSHCQLCPAARHLAVYLLDHF

MDRYNVTTSKQLYTVAVSCLLLANGVSLLSPRLKCSGMISAHCNLHLPGSSNSPASAPHPPPTPPQVAET

TGKFEDREDHVPKLEQINSTRILSSQNFTLTKKELLSTELLLLEAFSWNLCLPTPAHFLDYYLLASVSQK

DHHCHTWPTTCPRKTKECLKEYAHYFLEVTLQDHIFYKFQPSVVAAACVGASRICLQLSPYWTRDLQRIS

SYSLEHLSTCIEILLVVYDNVLKDAVAVKSQALAMVPGTPPTPTQVLFQPPAYPALGQPATTLAQFQTPV

QDLCLAYRDSLQAHRSGSLLSGSTGSSLHTPYQPLQPLDMCPVPVPASLSMHMAIAAEPRHCLATTYGSS

YFSGSHMFPTGCFDR

>Hsa-CyclinYL gi|214830655|ref|NP_001135772.1| cyclin-Y-like protein 1 isoform 1 [Homo sapiens] Gene ID: 151195 CCNYL1

MGNTLTCCVSPNASPKLGRRAGSAELYCASDIYEAVSGDAVAVAPAVVEPAELDFGEGEGHHLQHISDRE

MPEDLALESNPSDHPRASTIFLSKSQTDVREKRKSNHLNHCDLSNILPHKEQREKVPEEYFKHDPEHKFI

YRFVRTLFSAAQLTAECAIVTLVYLERLLTYAEIDICPTNWKRIVLGAILLASKVWDDQAVWNVDYCQIL

KDITVEDMNEMERHFLELLQFNINVPASVYAKYYFDLRSLADDNNLNFLFAPLSKERAQNLEAISRLCED

KDLCRAAMRRSFSADNFIGIQRSKAILS

>Hsa-CyclinY gi|32171247|ref|NP_659449.3| cyclin-Y isoform 1 [Homo sapiens] Gene ID: 219771 CCNY

MGNTTSCCVSSSPKLRRNAHSRLESYRPDTDLSREDTGCNLQHISDRENIDDLNMEFNPSDHPRASTIFL

SKSQTDVREKRKSLFINHHPPGQIARKYSSCSTIFLDDSTVSQPNLKYTIKCVALAIYYHIKNRDPDGRM

LLDIFDENLHPLSKSEVPPDYDKHNPEQKQIYRFVRTLFSAAQLTAECAIVTLVYLERLLTYAEIDICPA

NWKRIVLGAILLASKVWDDQAVWNVDYCQILKDITVEDMNELERQFLELLQFNINVPSSVYAKYYFDLRS

LAEANNLSFPLEPLSRERAHKLEAISRLCEDKYKDLRRSARKRSASADNLTLPRWSPAIIS

>Tad-gi|196003740| ref|XP_002111737.1| hypothetical protein TRIADDRAFT_56083 [Trichoplax adhaerens] TRIADDRAFT_56083

MAQLIDKLNLMVNSKGLQHPKANSVTAAKKRTIEESSDDYPLTSKKRAVLEDLTNASQDTLLKWHNLKKQ

QTNKNNVEQVQQQNEVKDHESDLGRIKTNIHEFLKCDEDDVVDGKSSCNENEESKISTAHLSTILSEEVQ

RVLTLPNDVADIDEADLTDPFRVADYAPIIFENMKQREAQLVVNDYLERQNDITEQMRMILIDWLCEVQQ

NFELFHETLYLAVKIVDRFLSARVVSRDALQLIGATAMLMSSKIEERYPPLVDDFVYICDDAYSRQAVLD

MERDICYALDFDLNIPIPYRFLRRYGKVASLSMENLTLARYILELTLQEYQFVTFKPSMLAAGCLCLALK

MKNCGEWTQTLVHYSGYEESELNELVQKLNAMIAKPAPENCKVVKTKYSHTVFYQVANIAPLQLETDV

>Tad-gi|196005765| ref|XP_002112749.1| hypothetical protein TRIADDRAFT_24944 [Trichoplax adhaerens] TRIADDRAFT_24944

MVVSPDRNVSSESVEKPQSNKRKATSVLDVDVYADDIHSYLRKAEYFHRPKYDYMERQCDVNGTMRSILV

DWLVEVSEEYKLRERTLYLAISYIDRFLSAMSVRRSKLQLVGTAALFIAAKFQEIYPPDCAEFAYITDDT

YNIKQVLKMESLMLKVLSFNLSSPTAVDFLERYGSEAGLDSEIRELSMYLTELTLKDYGFLQFMPSLIAV

SAVSLALHTFKLKYWPQELSTYTNYQWQQVSPCLNRIFEAFRLAHTQPQRAVVEKYKSPR

>Tad-gi|196002535| ref|XP_002111135.1| hypothetical protein TRIADDRAFT_22525 [Trichoplax adhaerens] TRIADDRAFT_22525

VVDIDALDYNDPLLCSDYISDIYKNMLKQEKRCTLDPDYMTGQPVITKGMRAILLDWLVDVHLRYNFHPE

SLYLTTYIIDRYLQTTQVNRKKLQLVGIAAFYIAIKYEEIFLASTDDLLYLTENSYEINEFIQMEAKILK

ALDFSLSRPTSIHFLRRISKAASADIEQHTFARYLTEIALIEYSLLSYLPSQIAAAASLISLKIFDKSWT

PTLQYYSSYSEDSLKPVARQIAKLAWKSWTSKYQVRKCIV

>Tad-gi|195999522| ref|XP_002109629.1| hypothetical protein TRIADDRAFT_53841 [Trichoplax adhaerens] TRIADDRAFT_53841

MANERQHSQLHHHPVVYSTWATHRDKKRIGRATFKRRRICHILNLPEELLVILFQNLSIRDLAKLRRVCT

RFNDVISNCPSLWRCANFHGVWPSVDNLLAMKRAAQVGNIQALVKLAIAYLYSEGLPDEGIINASRAAEY

FWMLEEKCKGIGPIIWLFIRPPWAPTANGNCIKECVFTEINEYCQIDGVTNSLYYDVAKTYSLQEGADKA

KIVELFAIAAEKGCPEASLELWKTKDREKVIDPGYYIEAARRIREIANTGCLEAQLELCLIYAKKKFTGV

NRNQATAYVRKMVESCRNINIKLVNRIQLELNDRMRYILIDWLVEVAEMKEFSSEMLCNAIDLVDRYLEI

NPIPRSNLQLLGISCMVIASRYHCVDIMTIREAAWLTDNTYKYDEVVRMIGEVFAAVNGEIRTPSAFDYL

KIFCTISEVSQKCTYLASFILELSWLFLENSRYKSAVKAAASLLLARVLIMGNELPWTEELKSYTGLSLE

DLSSCVLHLYKKCLAEKPPKDYYNSEVKSVHNRYSGPNKYNVAETDIPSLEVIAKILLVSDLSSFIENES

MEDTSCQQIDKSKCLQTLSFDDDVDSTIHMSSSESSMSFSECSNDISDTSLYAENDRKLCETAPLSPFNN

EIETLYTDQFGDGAIISEAKPKFDGAVDIAESNMNERLTGNDFVQTDACNADALFEENYELLSRPLTSPE

FYYNEGCKGRQLDIDNGHQFNAFSTDNFDDLSNKDSRAGFIPISEIDARCNNKLKISFSSNGSLPHDQNQ

TLDCKGHMPFLE

>Tad-gi|196003236| ref|XP_002111485.1| hypothetical protein TRIADDRAFT_15757 [Trichoplax adhaerens] TRIADDRAFT_15757

LSWADRKEVWQVMIENDNNFKRSPDCFDRHPNLVPNMRGILLDWMMEVCESFKMQRETFYMAMDYLDRYL

SLSDNILKQKLQLIGTTCLFIAAKIEEIQPPQVSEFAYVTDSACSEDDIIKLELQLLQTLEFQLSPVTVT

SWLNVYVQLFNIKLSSQEPVGDMLYPTYCGDLYMKAIRLIDLCILDSWCLMHSYRSIAASAFYLIAPSKQ

LAIDCTGYLWENLTSCISWMMPKYETVKKYCTSYEIQTINNIPKLDLHRIQTHHVDLTLL

>Tad-gi|196001479| ref|XP_002110607.1| expressed hypothetical protein [Trichoplax adhaerens] TRIADDRAFT_37409

MDLLCSEGFEKQAYKDPVLQKERVLQNLLKLEENYLPNRDYFTNVQREIKPFMRCMVTNWMLDVCHGENC

NDKVFPLAVNYLDRFLSMVSIRKIRLQLVGSVCLFLASKLKDRIPLTAEKLCSWTDYSVTCQELLDWELL

LLEKLKWDLGSVTPLDFLDQILYRLSFELKDYVHMLKKHAYTLIALCCTDFQLCTNPPSMIAGGCILCAL

AGVLPQNDNSIAIVNRIVQKITSIEPDYLICCKEQVEDLLSKTNSSHKIDQIDGANGYMSETPTDLDDMQ

HLVT

>Tad-gi|196011367| ref|XP_002115547.1| hypothetical protein TRIADDRAFT_29608 [Trichoplax adhaerens] TRIADDRAFT_29608

MAANFWKSSHYSQWVLDRQEILAGREEDLSYLSEDEIFKIHMFFANFIRHLGDLLKLRQQVIATAIVYFK

RFYSRNSLKSIAPLLLAPTCILLASKAEECGIINTGRFINACTNVVKQKYSSYFGSDYPYKMPVILECEF

FLLELLDCSLIVFHPYRPLLQFVEDFEKKDALLPCAWRAINDSYNTDICLMYPPYIIALACLHTACIIQS

IDCTQWFAELSVDLDLLFEVTRQIVALYELLKTYEESAEMKSLLDKIALPKGGRYDGKLMK

>Tad-gi|196007260| ref|XP_002113496.1| hypothetical protein TRIADDRAFT_37896 [Trichoplax adhaerens] TRIADDRAFT_37896

MAVPTEFSGVVLSLENTIYPDEKLQETPSQLDGLSKEMETNLRIAGCEYIQAAGILLKLPQVAMATAQVL

YQRYYYSKSFVKYNYEICAMACIFLAAKIEEHPRRIRDVVNVFYHIRRKLNELPNKVMDYMSNEYFHKKS

EVIKAERRVLKELGFCVHVKHPHKCIVTYLKILECNDNTELIQKAWNYMNDSLRTDVFMRYTPETIACAC

IYLSARQLGIGLPSNPPWYEVFDATTKEMEVYLLIVSVIDSVDMLIYQVKYVVDIIAS

>Tad-gi|196000815| ref|XP_002110275.1| hypothetical protein TRIADDRAFT_54106 [Trichoplax adhaerens] TRIADDRAFT_54106

MADGGEKSKVVMPPVVKISAQQLIEDKDHWPEYLKIGKFIAESGIKLKLGSVVIARAATIYHRFYFLCDI

SQFDRYLVAVTCLYLASKVEDTPRRARDVITTSYKVLHKEKPILKVDSFYWQLRDSVVNFELFMLRMLKF

DVSSELPHKYLLHYLKSLQDWCGESNWTTNHINQLCWQLLQDTSLLPFILLYPPSVIATAVIYLAVKCNN

IEVPSEGSTKPWWNVFSPNLNEEGLQQLCYKFMELYDT

>Tad-gi|196007480| ref|XP_002113606.1| hypothetical protein TRIADDRAFT_27302 [Trichoplax adhaerens] TRIADDRAFT_27302

MSSWYFCSNEIVNSPSRSDGIEITAECRYRREGARFIMDVGNRMNLRYETVATGIVFYHRFYMMHSFKTI

NRLIGAAACLYLAGKAEETPKKCRDLVKAVRTILSERQMEAFGDDPKEEIISHERLLLQTIKFDLCVQHP

YKYIVKFAKNLKDDRAQIEKVVQMAWNFVNDSLSTTLCLQWKPQVVAVSLLHLAAKLSKYNLSAAPDGPH

YDHSKSWWQHFLPEINSDVLEDICLQMLDFYDKTDVGASNYNMISPPKITMKVPQSIPYRTL

>Tad-gi|196012973| ref|XP_002116348.1| hypothetical protein TRIADDRAFT_5539 [Trichoplax adhaerens] TRIADDRAFT_5539

ITPTQREALISSMINLSRKHNIKRDTLFMAVNYLDRFLKVVTVTEDCFELVGLTCMMIACKVEECQPPKM

EEFLTSCTHYYKKAEMKRLEIIILNYIDFRLSPPIAPHFLEYIIHFHQHHYHQFSQQTYIELVNIANQVL

LKILPTYRFNHIKSSILAASAFEYAK

>Tad-gi|195998946| ref|XP_002109341.1| hypothetical protein TRIADDRAFT_53260 [Trichoplax adhaerens] TRIADDRAFT_53260

MYHTSTQRKYWTFPNEEEIAQHKGALHNQFFQKLKDDDSRHIRTKDLPSVEELDHLCTFYEFELMDLCRR

FDPPMPATAAVYMKRFYLVCSVMDYHPCDIMLACVYLATKVDEYNISIDKFLTMVPENDKERAKSRTLGF

ELLVMEKLKFHLTIHCPYRPVEGLLINIMTVIPEIADKIDELRRHIDKFLSKILYCQAMLIYPPSQVGEI

ALFPSQIALAAIIEAGEKAGLDLFESVISAVLNNNS

>Tad-gi|196013340| ref|XP_002116531.1| hypothetical protein TRIADDRAFT_31077 [Trichoplax adhaerens] TRIADDRAFT_31077

MATANASSSGSSSSSCNAADFAVTNFYFSDTELQNTPSRRNDISVATELYYRQTCALCIQELGMKLGANQ

LTINTALVYMHRFYMFHSLASYNLKNIAACAIFLASKSEEHPNKLNKVITAAYEYFSHESSPLDPKSEKF

LKLSQDLVDNEYAMFFTTGFDIEIMHPHTHVIKCLHGLKGKTCIIFHSFYNILWI

>Tad-gi|196013209| ref|XP_002116466.1| hypothetical protein TRIADDRAFT_30791 [Trichoplax adhaerens]

IKNGTIHKRYSSCSSIYLDDSTVSQPNLRCTLKCFICFALRMYRHDPPRLKDIFDEELHPLMRPVRKETIINLPEHRHVY

KFFKTLFSSAQLTAECAIISLIYVERLMEYAEIDIHPSNWRRVVLGAILLASKVWDDQAVWNIDYCQILKDTTVEDMNAL

EREILQLIMFNINVPSSIYAKYYFDLRTVADENNYILPTEPLSTERARKLEVNVIPMYQSV

>Bfl-gi|260806975| ref|XP_002598359.1| BRAFLDRAFT_69715 hypothetical protein [ Branchiostoma floridae ] Gene ID: 7221586 BRAFLDRAFT_69715

MAPRALLVESAIEEFSALWKVEQLLATVGLNSPMVLDTSLDSSDDNQSVIEDIDARGDSVLEVAEYATEI

FQYLREAELRHRPKPGYMKKQPDITNSMRCILVDWLVEVAEEYKLHNETLYLAVSYIDRFLSSMSVLRSK

LQLVGTAAMFLASKYEEIYPPDVGEFVYITDDTYTKKQVLRMEHLILKVLSFDVAVPTINCFQKRFLQAA

KVNSKTESLAMYLAELTLQEGETFLKYVPSTIAAASLCLAQHTLNMQPWTPTLMHYSGYTLADLLPCVQD

MHRSFQAAPSSQQQAVREKYRSPKYHGVSTILAPATIPTA

>Bfl-gi|260800992| ref|XP_002595380.1| BRAFLDRAFT_119004 hypothetical protein [ Branchiostoma floridae ] Gene ID: 7221467 BRAFLDRAFT_119004

MKVENQMTRRRTCRRCKSVYKQCKPSVRKAPDIWALPEELQLLMLRYLPAKDLANMRLVHSSFKQLVDNN

PTLWVNTNFGGIWPSPANVEQLKKIAEYGNLELLIKLGVAYLYNEGFPEIEGEQASRNGVRAAEFFMKSE

ELVRAAGTPPFTWLFIRPPWTTTGTCCKSLAFKTIKDQAASSENTSPALPFCIAKAQGLFDDEEERSEAI

KWFEKSANMGSEYATFTMWELKHKDQMIEPVGSRHAVRLLREITASGSWQAQLELCNGYAKGLYGGVCAA

QAAEVVRQFVLSSQPSYSHRIFEIQKGMNNIMRFILVDWLVEVASMKDFSTQVLHAAVRCVDRYLMTHKT

PRSKLQLVGVASMVLVTRYLAKDILTVREAVWLTDNTYKYEDVVRMMGELTATLRGEIRALTSADYLDLF

FKLQLLDQRTKCLAAYICDLALLQTEIMGTYSPAVIAASALLLAKMTTNKDAELWSSHMTQFTGLQVVDL

LTCTLQVYHKCLCQPPPRDHHNNPLTAVRQHYGDERFCKVSEIELLSNNELIARLGLDSNVAAEPITADF

ASQKLNIDILGSSPRPVKSARPCPGQDADRSTMVTPIMELSMDNSGYEGDMESEGEYLDMSDEEMSEDDQ

GEDEVCTCCSASASSDNASDWLDVISPTATSKPLFRTQIAVDQSMTSMDVSMETPSQFLQDFNSFDIEGA

SNSSGVYHDEMGEATAKVFQPLSKGMRNITIRARRRASSPESSSPCSSACSSPRAPLRALQNISQDVANA

VLPQKKSVKRKECA

>Bfl-gi|260789607| ref|XP_002589837.1| BRAFLDRAFT_271951 hypothetical protein [ Branchiostoma floridae ] Gene ID: 7220460 BRAFLDRAFT_271951

MDLLCCEGDLIRRGYRDPALLGDNRVLNNLLITEDKYLPSTTYFKAVQDEVKPHMRQMVATWMYEVCEEQ

RCEDEVFPLAMNYLDRFLSQVPIRKNHLQLLGAVCMFIASKLKETIPLTAEKLVIYTDNSIRCQELMDWE

LLVLMRLKWDLSAITPCDFLEHILSRLPIERERSDMIAKHAQTFIALCCTEFKFAIYPPSMIAAGSVGAA

VNGLVGLGGIWASPNELLEQMQKITNIDMDCLRACQEQIEQLLATSLCNPMSHPDPLATKHQEQKDQSTT

PTDIRDIDF

>Bfl-gi|260786520| ref|XP_002588305.1| BRAFLDRAFT_265991 hypothetical protein [ Branchiostoma floridae ] Gene ID: 7230418 BRAFLDRAFT_265991

MKLLRGFWDAKRLASTLEEAIAKEASTWKPRLLKAKVNQDAEIGPSERDEAVRWLATLSTKVQAYPETFA

MSVSILDRFLNAVKARPKYLRCISVSCFFLAAKINEEDEAIPSAGELVRVSECGCTANELLRMERIILDK

LGWNLKDVTALDFLHIYHALLTTYQPQLLDTYTCMTPSRHLAHLTRKLQRCMACHQVLGFPGSVVSLGLL

SLDLEMLIPDWLAATFMLQKMVKVQNESLIRCREVIARHLAAQRDMTQNLVYIISAPANKSAKRKVAQIE

DDDIYDGIKRLYDQDWKGDPETVAVTEEVAMVTGGVACSSQLRQDRDGNLSPTLPPLQPITAMI

>Bfl-gi|260786518| ref|XP_002588304.1| BRAFLDRAFT_281367 hypothetical protein [ Branchiostoma floridae ] Gene ID: 7230417 BRAFLDRAFT_281367

MRNMCEHHAADLGRLWYQLQTAVRRAPSYQPNLIHLDMVEDDEDLRDPVVSSTQRDDAVDRLRCLNRCLR

FKHEAFALAVNILDRFLSIMKVRMRFLGCLTITCYFIAINMLEEDQELPSPVHLIRISQCRCTEADLFRM

EGIVCQKLYHDFGAVTPLTLLQLYHGICAMTGTMDAILEKRNLTPAEHLERTIAKLEACLCRSPFTKFTA

PVLAMSLLMCDLDAFDTRDNNPDLCSTLNTLKVASQINEQHLVECQKKLEEFLLQYSSPVSRRPTSRFTW

VLSSRTARQLRPSMQWAMDLPTILEDGTDGYGSYGSR

>Bfl-gi|260825762| ref|XP_002607835.1| BRAFLDRAFT_275068 hypothetical protein [ Branchiostoma floridae ] Gene ID: 7207908 BRAFLDRAFT_275068

MAGNFWQSSHCQQWILDKQDLLRDRQDDLTNFPDDEYQKVHIFYCGVIQAVGEQLKLRQQVIATATVYFK

RFYSKYSFRTIDPLLMGPTCVFLASKVEEFGVISNSRLITACQTVIKNKFSYAFNQEFPYRINHVLECEF

YLLEMMDCCLVVYHPYRPLTSYVQDMGQEDTVLPLAWRIVNDSYRTDVCLLYPPFMIALAALHMACVILQ

KDAKHWFAELSVDMEKVGVSYAMNSTVILP

>Bfl-gi|260798658| ref|XP_002594317.1| BRAFLDRAFT_260241 hypothetical protein [ Branchiostoma floridae ] Gene ID: 7216738 BRAFLDRAFT_260241

MKDCDRMSRRSVRQKARKQKLETTEEPAFVLCTRKRKASPENMGNQDVAKRRQQFQIQNRWVPISQGSHV

GTSFIVATDSEELNADFEVGSHFQIRNLLSDSRLSPLPDLNWASSRELWSPMLEKEALYNRDHNYLNRHP

GLAPRMRAILLDWLIEVCEVYRLHRETFYLAQDFIDRFLSTERDLPKHRLQHIGITALFIAAKLEEIYPP

KVTEFAYVTDGACTDEEILDMELVLLKALNWELSPMTVNSWLNVYLQLANLDAIDFEEFYLPQYSGHTFV

QVAQLVDLCMLDISSLQFSYAAIATAALYHNSCRDICLRVSGFSWEEVAPCVQWMTPYAITRREAGYVPL

KMYNQISPEAAHNIQNHTCDLAMLEKAQARQAEMSSLSRASPLSIPGMITPPQSNKKQHTTT

>Bfl-gi|260803926| ref|XP_002596840.1| BRAFLDRAFT_129102 hypothetical protein [ Branchiostoma floridae ] Gene ID: 7255588 BRAFLDRAFT_129102

MPCWYYEKKDLLCSPSATAGVDYATECRYRREGARFIIDAGTALGLYPSLYSVQKPQTFATGVVYFHRFY

MFHTFKDFNRYVTGACCLFLAGKVEETPKKCRDIIKTARALLNDKQFAPFGDDPKVQQEEVMTLERILLQ

TIKFDLQVEHPYMYILKYAKSLKEDAQSLTLDKNKLHKLVQMAWTFVNDSLCTTLCLQWEPQIIAIAIMH

LAGRLTKFDMLGAVQSNADKPVKNWWDRFEEDVSLELLEDICHQVLDLYSQPQGISTPSPPPSPASRPAK

RPRIESPGQMEPKPSTQAAANGVPSAERAEFSADDSSQEAVPSSSTQSYHSAPTSSTSSVKSSPVEPQST

ANTELVSMDISESSNPPIEQNPAPSSSTQLPTLQASASSTSVHQQVPYSYKAGGYTSQIPSASGMPAAPD

SKPPPPTQSYTQPPPPSHQPGFPPPGHPPPNYPPPNVPPPNYPPPVPPPGPPPHNTAAPPPNFPQGVPPD

PSLSQTTGTTSYLPKGTGDVNFGQQSSTGSYSSQGTGVGAASSSYGADGSSMGTGGATGYSQQGTGYGTS

QANFAQQGAPPRGMYGAKGGDNFGNAQSGHYQQAGGYGTSGAGASGYGSSGGNFGQQAGAYGAGAGGTNY

GQPAGNTAYNQSGGYPGSGTYQGGPGKGGGYSGGGYSDGSNQFSSTPYKPPHTPTTPGYPTPPMGGSSSF

QPRGPHYDTPRGPPPTGQNNSSAPSAGLPSIRITGRDGMWNRRDSRGGWNR

>Bfl-gi|260813108| ref|XP_002601261.1| BRAFLDRAFT_60750 hypothetical protein [ Branchiostoma floridae ] Gene ID: 7248490 BRAFLDRAFT_60750

MANAPFKIGDKEFSGVVITLENCLLPSEKLDATPSVNDGLEHETEGDLRILGCEYIQTAGVLLRLPQTAM

ATGQVLFQRFFYSKSLVKHNMEIVAMACVYLASKIEEAPRRIRDTINVFHHIRQRRNNRPAQPLVLDQNY

INTKNQVIKAERRVLKELGFCVHVKHPHKLIVMYLQVLDCEKNRKLVQTAWNFMNDSLRTDVFVRFSPET

IACACIFLAARQLKVPLPNRAHCPCHWYELFGASEDEVEEISLTILKIYARDKKNYEDLDKEVEKRRKVL

QEAKLRARGLLDEQGRPVDSTTGNSSPSSRPTSPKTLSAKPSPVRDASSDKKVKREDGASSGSAHSHKEN

RGGRDRSRSRSSRSRSRSPRRKGRSQSGSSRSSSRSRSDNRNRERRSHERHKRALSGKRSHAHKRRRTRS

PISRSRSRSRSRSHSRSPDRYSRKHYHKERPRHPSRSRSRERHKAAKHSSHSRDRHGRRR

>Bfl-gi|260817673| ref|XP_002603710.1| BRAFLDRAFT_93075 hypothetical protein [ Branchiostoma floridae ] Gene ID: 7245274 BRAFLDRAFT_93075

MEVQKEWWKTQLATDIHETLRTKEAELPPFKGLSPQLGLRRYLVDWLSIINEKQGVHCTALHLAVYLLDQ

FMDSYDIQESRMHLVALGCLLVACKFEEEERRVPRIKKLNQYVREVYSEEEYLQMELTILKFFQWNISLP

TPAHFLDYYMTEGVSQSDLHAGYPVCSVNKSRLYLEKYCHYFLEVSLQGEYKLVRKTRTGLKDMSTSI

>Bfl-gi|260780863| ref|XP_002585555.1| BRAFLDRAFT_290049 hypothetical protein [ Branchiostoma floridae ] Gene ID: 7240362 BRAFLDRAFT_290049

MFYSSTQRKFWTFSSEDELRKLRCQANQDIRQKHEASGKDCSKLLTPDEELMVCQYYHNRLREFCVKFQP

PMPKSVMASASSYFKRVYLQNSVMEHHPKIIMLTCVYMACKVEEFNVSIMQFVGNIRARSEERERAVDII

LNNELQLLQLLNFHLTVHNPIRPLEGLLIDMKTRHRSAEEVETLRRWAEEYLDKSFLSDACLLFPPSQVA

LAALWHSGNTVGMDVLSYVHHCLGKNGDGADQTLLMNQIKKVQEVVGNVPQLDASLVEEAKAKLAAVGSL

EVTHDSQGTKRKQGTEQQGKQAGKKKAKQAKSAQEDGGLLSLSSLE

>Bfl-gi|260786596| ref|XP_002588343.1| BRAFLDRAFT_281384 hypothetical protein [ Branchiostoma floridae ] Gene ID: 7230413 BRAFLDRAFT_281384

MFYSSTQRKFWTFSSEDELRKLRCQANQDIRQKHEASGKDCSKLLTPDEELMVCQYYHNRLREFCVKFQP

PMPKSVMASASSYFKRVYLQNSVMEHHPKIIMLTCVYMACKVEEFNVSIMQFVGNIRARSEERERAVDII

LNNELQLLQLLNFHLTVHNPIRPLEGLLIDMKTRHRSAEEVETLRRWAEEYLDKSFLSDACLLFPPSQVA

LAALWHSGNTVGMDVLSYVHHCLSKNGDGADQTLLMNQIKKVQEVVGNVPQLDASLVEEAKAKLAAVGSL

EVTHDSQGTKRKQGTEQQGKQAGKKKAKQAKSAQEDGGLLSLSSLE

>Bfl-gi|260809954| ref|XP_002599769.1| BRAFLDRAFT_205848 hypothetical protein [ Branchiostoma floridae ] Gene ID: 7224291 BRAFLDRAFT_205848

HFRIVRFMMESGIKLRMTSVPMATAAIIYHRFFSICILQDYDPYLIGMTAISLASKVEEEHLKIRDVINV

CYRYDKPPLESQTELADLRQAMASCELLIMRVLGFNVTKELPHKYLLHYLKSLGDWIDASVWDRTPIRDT

AWAMLRDLYHGKVCLQHEAQHLAVAVLYFSLLCFGIEVPLNNQAETKWWKVFSEDITEEEIKNIIEQIMD

VYDLEQRL

>Bfl-gi|260835834| ref|XP_002612912.1| BRAFLDRAFT_227829 hypothetical protein [ Branchiostoma floridae ] Gene ID: 7247723 BRAFLDRAFT_227829

MAAVERWHFTPQQLMDTPTRKCGVDADKELSYRQQAANLIQDMGQRLTVNQLCINTAIVYMHRFYMYHSF

TKFHRNALAAACLFLAAKVEEQPRKLEHVIRVAHVCLHRDSPNLDTKSETYLQQAQDLVINESILLQTLG

FEVAIDHPHTHVVKTTQLIRAPKDLAQTAYFMATNSLHLTAFSLQYKPTVVACMCIHLACKWASWEIPRS

NDGKYWWEYVDPNVTLDLLDSLTTEFLHIMDKTPSRLKRKISNYKVS

>Bfl-gi|260807385| ref|XP_002598489.1| BRAFLDRAFT_66866 hypothetical protein [ Branchiostoma floridae ] Gene ID: 7226571 BRAFLDRAFT_66866

MESNTSAYVREPWLHVQGILAAALKTTVFEQGQQHSTESSILYWCPVTSFVRNRRGLTAVDWVVAVFQDV

TVGVEAEEKDAWKGKEEVHPLCAEEIVSLCDEGQYTRGQLRMLERKVLSTLGFHLTVPTSILFLEHLAEA

GRYDHLTSCLARHLLVTSLQDYVICQHAPSSLALAALNLAASLVNNTRD

>Bfl-gi|260820262| ref|XP_002605454.1| hypothetical protein BRAFLDRAFT_74268 [Branchiostoma floridae]

MGNANACCVAPQGQRGKKKDRSDVYAPEPEVPKEESSANLQHISDREVAEDHEEDPSNHPRASTIFLMKS

TAPNGKVLDGLLVHHSSKKRNNHHINHVYLERLLTYAEIDIAPCNWKRIVLGAILLASKVWDDQAVWNVD

YCQILRDITVEDMNALERQFLELLQFNINVPSSVYAKYYFDLRSLADANELIFPVEQLSKERAQRLEATS

RACEEKMKEGNKPELKRTVSAENMSPRLSRVVLS

>Cin-gi|198428764| ref|XP_002125998.1| LOC100176577 similar to cyclin A [ Ciona intestinalis ] Gene ID: 100176577 LOC100176577

MSHNVLSTRSTSHVGDNRQKPFNENAGARPKTTQGRAVLGVISQNTASGRVQPFRAAKQNATTEGFFSGQ

PPVQKLSGQNFKIFDECSSNNDAKVQQKPSVPAIASNQLHPALTALPSTRQPLVNLPVNVSLSSVDSPMV

LDTSDEERLNIFDIDSNAGIYGLSEYATEIFQHLREAEPNYMRKQQDITVGMRAILVDWLVEVADEYKLH

TETTHLAVNYIDRFLSHMAVLRGKLQLVGAAAMFIAAKFEEIYPPDVGEFVYITDDTYTKKQVLRMEHLI

LKVLNFDVAVPTSNQFLKRYLKSAGADKKTEFLAQFLCELALVEFDCTQYLPSMIAASSVCLASYTVSGK

IWDETMEHYMQYQLQDLAPCIKRLHEILAGASKNSLQALFEKYKDAKYDCVSNITATPTLPFDEMEAEEC

>Cin-gi|198433631| ref|XP_002126215.1| LOC100184393 similar to cyclin B [ Ciona intestinalis ] Gene ID: 100184393 LOC100184393

MATTLANRLENQIQENLPAKHGAIIKKRTAIPTTRPALETIDNKKTSKAVGGAKQKMQLQKPTTTRNALK

ASNIPMKPRTTLQKSVQNKPTLKVNHNKVLSQPKALPTSQPIKQEIQKLLPSSVKDSPMLIGSPCKIDTD

DLSSNIFDLNLKSNVENIDENDCENPQLCSEFVNDIYHYMLYLESESPIRRNYFKDTGFKPRVRCILVDW

LVQVHHRFQLLQETLYLTIAILDRFLQVHPVPKVKLQLAGVTAMLLASKYEEMYAPEVSDFVYITDKAFT

QAQILSMEILMLKTINFSLGRPLPLHFLRRNSKAGQVDATQHTLAKYLMELSLVDNDMCHVPPSQLAAGA

LCLSIKLLEDSEWTPTLEHYSTYTKEDLIPVVCHLAKNLKSAEKSSYQQAVKSKFSSHKMMKIARIDAVK

GTMVEELAEQAASHKL

>Cin-gi|198423591| ref|XP_002126500.1| LOC100184160 similar to cyclin B3 [ Ciona intestinalis ] Gene ID: 100184160 LOC100184160

MKSPNRKKPSRSYSIEEEITWLSLTGKNKVGGKPSSKRPATSPAKGAVHKRSAFGDITNAGVVNVKAIAK

PEATKKVDSKKNKTKRHSLRLSKQVIKTKQDTSANVEKLSQITQDFFISSSQETSSSSASSVYESLPNSP

ATPTCFIEHKASPRRRALNFTDVDAENVNNINEAPEYAFTIFEYMRAREQSFPINENYMVEKQTEITPEM

RSILVDWMVEVQENFELNHETLYLAVKLVDCYLQQVKIKKEKLQLIGATSLLIAAKFDERQAPYLDDFLY

ICDDAYNKQQMMQMERTLLKTIGFDINIPIAYRFLRRYAKCAKSSMEVLTLARYIMELSLQDISFIGKSA

SLMAASALWLAFKMKKTNFQWNDTLVYYSSHNEQDIIELAVQLNHMLSSRDTKLKTIFTKYSHSIFYQVA

KTTCLSQVEINEETSRIQHFTQL

>Cin-gi|198421112| ref|XP_002123915.1| LOC100187071 similar to AGAP012413-PA [ Ciona intestinalis ] Gene ID: 100187071 LOC100187071

MIANRRPLGNLDPNSTRVQPTRAAKSGHKWLTNRRSLLSDEIHNNNLALQSRDTNETSNSILNHKHNQNA

KQTNKNHRPGKENCDNYKFVHPDSDLEKVTLLSSTREVLSASQKYELEDDQCFLTPNGEKSNTSCVSMEM

LTSSLSSLDSSKRSISQPHDVALDTDEILNVIQRKDPETDILDEYTEDILRYMVYSEAKYQPRKDYLEKQ

NEISSTMRVKLIDWLIEVQDEYKLQNETLHLAVAYVDRFLSEMSVSRPKLQLLGTTSMFLAAKFEEIYPP

DADEFAYVTADTYARSEVLLMERLMLSQFKCTLAVPTTLQFLNIFHKKSNLSEDAKQLSFYLSELALLHD

VYLQYSPSVRAAAAISLAVCTLRQAHNCDVDNGNADTACGIKTPYSTILVHDNMVPTKVLLSSVLQRCMK

DKLQKVFDCTHELARSHVVESNEKNRHFIFEKYSMTKQRSVTAFSPLLSNEVTSQSHLTWEKVMTMWMT

>Cin-gi|198428427| ref|XP_002121291.1| LOC100178750 similar to cyclin K [ Ciona intestinalis ] Gene ID: 100178750 LOC100178750

MEKPKIKHKITSPCWYYDKADLKKTPSFLDGVNPETEGRYRREGPRFIFDMGTRMGLHHDTIATGIVFFH

RFYMFHSFKKFPRHITATCCLFLAGKVEETPKKCKDLIKVARGLLNEAQFVQFGNDPKEEVLTFEKVLLQ

TIKFDLTVEHPYKYMLQYAKKLKGDRAKIEKLVQMSWIFINDSFYTTLCLQWEPAIVAVAVMHLAGRLCK

FEPQDWAYNRGRWWEQFIDDISMELLEDICHQVLDQYPHSKGKPQIPNYTNPAPPQPPIAPPTPPAPQRQ

PFPQYPGSYDGNYHQFTTPEMYQQNKAYPPAYSGMNQAPTGLATVRITGRRETNPTQQWP

>Cin-gi|198425946| ref|XP_002125614.1| LOC100185975 similar to MGC81420 protein [ Ciona intestinalis ] Gene ID: 100185975 LOC100185975

MDLDWPGCKNDLAKDIHDTLKARENFMLRLTGSSKLMYWRRYLVDWLALTCQKYRLNSNAQHLAVCLYDR

FTDQFQLGVEDLQMLVLCCLLVASKFEEREEKIPKFKILMDHLQWNLNAAEYMTMEIRLLSAFEWDIGFP

TASHFKEYYMQVALGTRDLHAGQPLTNREQVYMYLEKNVSYFLEVSLQDQAFLVFKPSLITASCVAASRI

CLHIAPTWTVELHKVTNFAWHHLVPCIEILLRLHDEDRRAMHSNGSSSHVSGTLSIAHQQVLNSTGSTPV

TSPVPPLPFTSDNQRNQHMPTTIPTGRYNRGYLV

>Cin-gi|198417861| ref|XP_002127570.1| LOC100185796 similar to cyclin E1 [ Ciona intestinalis ] Gene ID: 100185796 LOC100185796

MMPKDIAADRKQKTNPKIKKESAILTSSTTSLNTSRNRKRLREESAKKEATILMSSEEKEDSKRRCVMDT

RVENRRQQFYTKTDFKAIDHEMFPTPQKEGADGENDDPMVLTSPIKHQGKRGGPLQEINVGKAEHPLSSH

YQFRNIFTSAPISRASPLPVLSWADSDQLWKSMVNKETVYCRNSSYMDRHADLQPRMRSILIDWIMEVCE

VYSLHRETFYLAVDYIDRYLSATKNIHKTRLQLVGVTALFIAAKLEEIYPPKLSDFAYVTDGACTDDEIL

SQELIMLTALKWSLSPITAISWLNVYLQTAHAAFAPNPSSASFFLPQYPQETFVHISQLLDLCVLDIESL

EFSSGLLAASALYHFSSRELATQVSGFHFKDLAACVHWMAPFAITVRDTGLQPLKQFKRVLETDSHNIQT

HANNIDTLDSAFENRSRVHAEVTCQSPTEVGGFMTPPKSGQKQSVPVTPQTGVMGT

>Cin-gi|198417413| ref|XP_002123834.1| LOC100186373 similar to cyclin D2 [ Ciona intestinalis ] Gene ID: 100186373 LOC100186373

MSLSCEETCVVERTLRAKKDPALLDDPRVLRNLLELEDRYLVSTSYFECVQKDVQPYMRKVVATWMMQVC

EEQKCEDDVFPLSMNYLDRFLSVHPIHRTQLQSLGSACMLIASKVKETLPLTTEKLVVYTDHSVGQDELL

KFELLLLMRLKWDVLSITPIDFVDQILHRLHMDESTVAVAKKHAHTFIHLCCTDHTFSVYTPSMVAAGSV

GAAVVGLQSASRIWTSRQLLLEKLHEITGVDLDILRECLAQVETTLRANLLHISNHPNKSAEHPGDATAH

LAAGCRQVDNTPTDVDMVF

>Cin-gi|198429958| ref|XP_002128852.1| LOC100182825 similar to cyclin T2 [ Ciona intestinalis ] Gene ID: 100182825 LOC100182825

MRETQERVLFTALAVTFRFDWVMASDNNRKWQYSREDLDQTPSRKDGIDADKELGYRQQAANLIQDMGQR

LSVNQLTINTAIVYMHRFYVYHSFTVFSRYAIAPTALFLAAKVEEQPKKLEHVLKICYVCLHPDKPHLDT

HSDSYLKQAQELVQNELVLLQTLGFDISVDHPHTHVVKCTQLVKASRDLSQMAYFMATNSLHLTTFCLLY

KPTVVAAMCIHLSCKWSKYEIPLSNDGKAYWTYMDPIITEPLLDTIIEEFLKILNRCPTRLRKLKNYKPS

LTASSSNKETYEFSEKDVKHHPSSNTPVSAAGEKPENNHPPPKLKEKKPAMSLQEYKRTHPNTRVPTKPA

PPTQPVHSSSGHTKPVESRNNQNPMDPSRSTLPQSRPHPHQQPHPSSRAPPQKPDLNRSHNRPTAHPEAH

RNMMKPQQPPNEKKPDQSKGWHQAPHSQQPKQPKPEPSSTPDDLAAVNKDLEGRIRTEKLKQRAAPESHH

NRSATHSGVTSSGRHLPKDAQEARRHHSEKTHAAHSHSQKESKALDYKAMCELYKRNPEKLHHYLKQKAA

AVALSTEEKNLLGKLRAKEEERRKRKQAEAALKHQQQKSTRPPPPPPPAAATAASSEASVAPLRIKLSSS

DGQLNTSVAKIVGSSSGSGSEVSPVRARKRGHLDSGGQVEHSAAKLSKRGHHHHHSGNGSSSGGSKHKHH

HQHRHHSKSSKKHSEILNDAIFQKSLSDAISKSHMQVKQTSGKSIPTVTLKRAANGSGPPVFTPMPNTAM

FDPSAPIPMSMPDEIFEDEEDSGAFVAGSLPTGGSYDSARSSPEPGEINESPPHRPKQPAKVPGLMSPSS

GSDTKLHRGYGYHSNNEKDFSSPPPLVQRSTVN

>Cin-gi|198429948| ref|XP_002128340.1| LOC100177336 similar to Cyclin-O (Uracil-DNA glycosylase 2) (UDG2) (Cyclin-like uracil-DNA glycosylase) [ Ciona intestinalis ] Gene ID: 100177336 LOC100177336

MNNLCKTPVVLLHENEAKQQTHVDGYAAKRRDEFYLQHLKGTVCSLEAPSHKSTKCGTGLRNIVGSNPDF

TVDGFNYDKNNNNCSDGLLQGNICKQNGSSKTKKTSSGSSKRRRSNGRKVAIAAPSSIRSSVSKLGRCGF

GSTKRSSGDSKIQNLAKRFRSSEYLARSPNLSEKPGFQNDYLGMSETCVMYDDYVVIEKSSEKSYVHYYE

KWSNESDKSSPNASLATPDSTLYCYEEAAFDDKSVTGCEFSDIGLAIFAVTSLDKLDIRDVYWQMKAKEY

YSHPSTSLCEQKIITQRCRFILVEWLVMVTHHYKIQGETLHLAVNILDRYLEREFVTIRKKYLQLIGITS

LLIAAKQLEVEIPPVSACLGLCRNLYTRPQLLSLERVLLITIGFDLNVPSSHLFYDTIISIHYDVYVQKC

HVVLVKDEPADVQEYFIALLCLGRSFLEKGLTNYQFVQFPPSVAARAAFRLSTSILDEPASSVGLKVFQD

YSWPKYDSRASFNAGSWDGVTITEEELLEYEEENVLSCLHSIRSLYDEAELKSFSRSTAAFVPKYLY

>Cin-gi|198414425| ref|XP_002128551.1| LOC100176109 similar to Cyclin-L1 (Cyclin-L) [ Ciona intestinalis ] Gene ID: 100176109 LOC100176109

MGSVVTDTVASMQQEYAGVKLTLENCLFPTEKIEHTPSSSDGLNASTEEDLRLLGCEYIQEAGIMLKVPQ

VAMANAQVLFQRFFFAKSFVKNKMEEVAMACIWLASKVEEAPRRVRDVINVFHYIRQRRVTKSPTPMQLD

SNYIMLKNNVIKSERRLLKELGFCVHVKHPHKIIVVYLQVLEMEKNRDLVQTAWNYMNDSLRTTVFVRYT

PETIACACIYMAARVLQVPLPNQPHWFCLFNATEEDIQQICMDLMRLYQHKKATHDELEKQVDIRRKFLQ

QEKAKAREAAGLSTASVQNSPVTNSSPFQDNASPKRDDKKRPSSLSRDHRSDAGDSRSRKRTHHSTDNAH

ERTRHKRRERSPRSLSGSRSPAYKPRHHDGNGVSSDLPKSYSISKDRRDSSEEKERYRHKRRDHSGSRDR

YRNEKRHKNGRKDDGDERSSKLKKHKKHRSRHHGSPRKDRH

>Cin-gi|198414966| ref|XP_002131678.1| LOC100185167 similar to MGC116479 protein [ Ciona intestinalis ] Gene ID: 100185167 LOC100185167

MAGNFWQSSHCKQWILSPEFLVRERELDLQVMGEVDYQKLMMFFANVIQSIGEQLKLRQQVIATATVYFK

RFYSKHSLNSCDPLLLAPTCIFLSSKVEEFGVISNSRLISVVTTVIKSKYSYAFPNEFNYRIHHVWECEF

YLLELMDCCLVVFHPYRPLVQYVNALGMADALLPIAWRIANDSLRTDVILLYPPFQIALACLHMACVVQN

QEAATFQWFADLNVDMEKIIEITNLILKLYELWKAFDEKSEIPGLLKKMPKVLLSQQRDGKNDSNSGRNN

VSVKDQNKHI

>Cin-gi|198438473| ref|XP_002130166.1| LOC100186282 similar to Cyclin-related protein FAM58A (Cyclin-M) [ Ciona intestinalis ] Gene ID: 100186282 LOC100186282

MDSDQKTHIEVVKFIVKCSIKLSLQDAVQASSSILYHRFFKHCSVEEYDPYTIAATAICLATKVEEQHTR

LRDIVNVCHRTCHPDLKPLELDSEFWNLRDTIASCELLMLRVLKFNVTCIHPHKYLLHYLMSLSHLFTRT

EWLKSMVSDVAWALLNDSYISNTCLNHGPEIYAISVIDLALQSCKIKVPLNEHADKKWWQVFYEAATKEA

MLMVQRDIAHTINLANSIAQNNNKNHK

>Cin-gi|198427565| ref|XP_002129501.1| LOC100177816 similar to Cyclin-H (MO15-associated protein) (p37) (p34) [ Ciona intestinalis ] Gene ID: 100177816 LOC100177816

MFNSSTQLKNWTFEHEEELIKLRNNANTKYRTSYCDVNNHVTPDADTFFLTVSEHCFLVQHYERKLQEIC

WKFKPPMPLNVVGTSCMYLKRLNLRKSVMDYHPRLMHLACIWLACKTEEFNISMDQFVQQVAHGNEEIGD

AILTIELILIQELNFHLTIHNPFRPLEGFLIDLKTRYRNLENAEQLRKPAKDFLVRSLNTDVGLLYAPSQ

LALAALLSAASMRNLNIDRYVTSVLLVGQPQTVLEQTIQRIKKIRTIVKNAVSAPLPSDQIEILEQKLSH

CYNKEKDPTSYEYSCLQQEIESQKYDKQTKKRKLEHERRRKIETELLGF

>Dme-cycA-gi|24662962| ref|NP_524030.2| CycA Cyclin A [ Drosophila melanogaster ] Gene ID: 39340 CycA

MASFQIHQDMSNKENPGIKIPAGVKNTKQPLAVIGGKAEKNALAPRANFAVLNGNNNVPRPAGKVQVFRD

VRNLNVDENVEYGAKKSNVVPVVEQFKTFSVYEDNNDTQVAPSGKSLASLVDKENHDVKFGAGQKELVDY

DLDSTPMSVTDVQSPMSVDRSILGVIQSSDISVGTETGVSPTGRVKELPPRNDRQRFLEVVQYQMDILEY

FRESEKKHRPKPLYMRRQKDISHNMRSILIDWLVEVSEEYKLDTETLYLSVFYLDRFLSQMAVVRSKLQL

VGTAAMYIAAKYEEIYPPEVGEFVFLTDDSYTKAQVLRMEQVILKILSFDLCTPTAYVFINTYAVLCDMP

EKLKYMTLYISELSLMEGETYLQYLPSLMSSASVALARHILGMEMWTPRLEEITTYKLEDLKTVVLHLCH

THKTAKELNTQAMREKYNRDTYKKVAMMESVEMSKDDFDQLCEAYNCKQKEDEHQQPDINTKSNVNLFYK

F

>Dme-cycB3-gi|24649824| ref|NP_651303.2| CycB3 Cyclin B3 [ Drosophila melanogaster ] Gene ID: 42971 CycB3

MAPTKATTRAAITSGHHQLQQAVNPILGALGAATRKGLTRRAAATGNIDPNVENMQTRAKRKADHSPIKN

DKIKRSALGNLTNNVKIMTLHPAQDEEQSGVGKKPTAQQLQALMDAKKQENLSVNVFGASKMTTRASSKV

EDSVENCHKVLDKLEEALARPKPRPKAVPAAKKTVLGEVQLPAMPNPMQIPVLLPPTHNLAAPQVAAVKP

VRRISNDFNKTEDSLYMSALEDVSSCDSMRLSGNFEAARRRSAKLQQKTEQQPQPLLLTLPETAPSQVVP

IPPVPEEVEDFDRKNWDDPFQVSHYAMDIFNYLKVREAEFPIADYMPRQIHLTTWMRTLLVDWMVEVQET

FELNHETLYLAVKIVDLYLCREVINKEKLQLLGAAAFFIACKYDERQPPLIEDFLYICDGAYNHDELVRM

ERETLRVIKYDLGIPLSYRFLRRYARCAKVPMPTLTLARYILELSLMDYANISFSDSQMASAALFMALRM

HGGPGQLDKQTWTSTLIYYTGYQLADFAEIVTALNAGLHRKPRATIKTIRNKYSHKIFHEVAKVPLLTNQ

ELFQGNLDLNESNLS

>Dme-cycB-gi|24658567| ref|NP_726244.1| CycB Cyclin B [ Drosophila melanogaster ] Gene ID: 37618 CycB

MVGTTLKMRGDENASENFKQVQLKKLTVPSMEATTKRAALGDLQNRGISRPIAAKDAAQKDSKDLKLTDA

LRNAKARVDSHWKKQPLGSTNGNGNGAVPPKVNEGGVSAFLRSNSVRNRVPTKTTVEPTKVTVKSSSSEN

VNEPTLKREDSNLSKKSLTKLRAALAKPVMGVSGIRREPVAVSRKEAETKKELPETKKDSLEVKKDATRM

PLIRGNSAVTTTTSTMPTTMSLSSKRLAGIEDIDANDKENLVLVSEYVNDIYDYLYQVELEQPIHKDHLA

GQKEVSHKMRAVLIDWINEVHLQFHLAAETFQLAVAIIDRYLQVVKDTKRTYLQLVGVTALFIATKYEEL

FPPAIGDFVFITDDTYTARQIRQMELQIFKAIDCNLSRPLPIHFLRRYSKAAGAEDEHHTMSKYFIELAS

VDYEMATYRPSEIAAASLFLSLHLLNGNHRAGTGFNDRHWTPTLTFYSRYSAAHLRPITRLIAKLARDAP

QAKLKAIYNKYQGSKFQKIALRTELTGALMDSIVGQSQRK

>Dme-cyc-gi|17136874| ref|NP_476960.1| CycE Cyclin E [ Drosophila melanogaster ] Gene ID: 34924 CycE

MKLEQKRKFIEMDPELGFEPPSAKRQQRLPALYGSEQGNLSSVASSVYTSPVVSVDGQSTQELLSIRSSP

AEDLSEAPHSPLPDSPDSPPSPDRGSKQTPVVVRYAAEQVVTSTVVTQKTEDDDLLDDSCEDYSYDEDDE

DDVEEEDDDVEIYSSTISPASSGCSQQQAVNGERTPGLPKHQEQIHHPVSDLMINMRTPMSPAVENGLRQ

CPLPALAWANAADVWRLMCHRDEQDSRLRSISMLEQHPGLQPRMRAILLDWLIEVCEVYKLHRETFYLAV

DYLDRYLHVAHKVQKTHLQLIGITCLFVAAKVEEIYPPKIGEFAYVTDGACTERDILNHEKILLQALDWD

ISPITITGWLGVYMQLNVNNRTPASFSQIGRQKSAEADDAFIYPQFSGFEFVQTSQLLDLCTLDVGMANY

SYSVLAAAAISHTFSREMALRCSGLDWQVIQPCARWMEPFFRVISQKAPYLQLNEQNEQVSNKFGLGLIC

PNIVTDDSHIIQTHTTTMDMYDEVLMAQDAAHAMRARIQASPATALRAPESLLTPPASSHKPDEYLGDEG

DETGARSGISSTTTCCNTAASNKGGKSSSNNSVTSCSSRSNP

>Dme-gi|21357017| ref|NP_650721.1| koko kokopelli [ Drosophila melanogaster ] Gene ID: 42215 koko kokopelli

MQCCCFSCCSCSLAFTRELWVSLNHIYSMVSGEGGAEWARERRRESERAVDIDNPPFLLLGAGARPCPAA

QRQRPGAQAGAARGQHLRRATADDGQYPAIQPDCHRNGEHGGGGTRRTGRAEGVAATHHAPPGGRRSGSV

QIEGHHSEVEQGGALQQDHPNPDYHSGVRPGSSSMKNVLDVMSMQQHVELNKAQTMKPIDYRKMNKPGVV

PMYIFECAAKLKMKPLTAACAAIVFHRFFREVKASDYDEFLIAAGSLYLAGKIKEDESVKIRDVINVAYC

TLNRGNDPVDLNDEYWSMRDAIVQAELLITRTLCFDLNIDLAHKYLLHYMKTLQDWVGTEVWNSVPIAKA

AASYLQDFHHSANILKYKPTHVAIGCLSLALQTYGIQVPLTDESDESAMWYKPLVKDFTRENQWEIIENV

IEVYKNEGFLNAT

>Dme-gi|18921115| ref|NP_569980.1| CG16903 [ Drosophila melanogaster ] Gene ID: 31178 CG16903

MATRGAGSTVVHTTVTALTVETITNVLTTVTSFHSNSVNISNNNSSSGAAPGADAAGGDAGGVAAAQADA

NKPIYPRLFNRIVLTLENSLIPEGKIDVTPSSQDGLDHETEKDLRILGCELIQTAGILLRLPQVAMATGQ

VLFQRFFYSKSFVRHNMETVAMSCVCLASKIEEAPRRIRDVINVFHHIKQVRAQKEISPMVLDPYYTNLK

MQVIKAERRVLKELGFCVHVKHPHKLIVMYLQVLQYEKHEKLMQLSWNFMNDSLRTDVFMRYTPEAIACA

CIYLSARKLNIPLPNSPPWFGIFRVPMADITDICYRVMELYMRSKPVVEKLEAAVDELKKRYIDARNKTK

EANTPPAVITVDRNNGSHNAWGGFIQRAIPLPLPSEKSPQKDSRSRSRSRTRTHSRTPRSRSPRSRSPSR

ERTKKTHRSRSSRSRSRSPPKHKKKSRHYSRSPTRSNSPHSKHRKSKSSRERSEYYSKKDRSGNPGSSNN

LGDGDKYRNSVSNSGKHSRYSSSSSRRNSGGGGDGRSGGGGGGGGGGNGNHGSRGGHKHRDGDRSRDRKR

>Dme-cycC-gi|17136688| ref|NP_476848.1| CycC Cyclin C [ Drosophila melanogaster ] Gene ID: 41801 CycC

MAGNFWQSSHSQQWILDKPDLLRERQHDLLALNEDEYQKVFIFFANVIQVLGEQLKLRQQVIATATVYFK

RFYARNSLKNIDPLLLAPTCILLASKVEEFGVISNSRLISICQSAIKTKFSYAYAQEFPYRTNHILECEF

YLLENLDCCLIVYQPYRPLLQLVQDMGQEDQLLTLSWRIVNDSLRTDVCLLYPPYQIAIACLQIACVILQ

KDATKQWFAELNVDLDKVQEIVRAIVNLYELWKDWKEKDEIQMLLSKIPKPKPPPQR

>Dme-cycT-gi|24666004| ref|NP_524127.2| CycT Cyclin T [ Drosophila melanogaster ] Gene ID: 39961 CycT

MSLLATPMPQAATASSSSSASAAASASGIPITANNNLPFEKDKIWYFSNDQLANSPSRRCGIKGDDELQY

RQMTAYLIQEMGQRLQVSQLCINTAIVYMHRFYAFHSFTHFHRNSMASASLFLAAKVEEQPRKLEHVIRA

ANKCLPPTTEQNYAELAQELVFNENVLLQTLGFDVAIDHPHTHVVRTCQLVKACKDLAQTSYFLASNSLH

LTSMCLQYRPTVVACFCIYLACKWSRWEIPQSTEGKHWFYYVDKTVSLDLLKQLTDEFIAIYEKSPARLK

SKLNSIKAIAQGASNRTANSKDKPKEDWKITEMMKGYHSNITTPPELLNGNDSRDRDRDRERERERERDP

SSLLPPPAMVPQQRRQDGGHQRSSSVSGVPGSSSSSSSSSHKMPNYPGGMPPDAHTDHKSKQPGYNNRMP

SSHQRSSSSGLGSSGSGSQRSSSSSSSSSQQPGRPSMPVDYHKSSRGMPPVGVGMPPHGSHKMTSGSKPQ

QPQQQPVPHPSASNSSASGMSSKDKSQSNKMYPNAPPPYSNSAPQNPLMSRGGYPGASNGSQPPPPAGYG

GHRSKSGSTVHGMPPFEQQLPYSQSQSYGHMQQQPVPQSQQQQMPPEASQHSLQSKNSLFSPEWPDIKKE

PMSQSQPQPFNGLLPPPAPPGHDYKLNSHPRDKESPKKERLTPTKKDKHRPVMPPVGSGNSSSGSGSSKP

MLPPHKKQIPHGGDLLTNPGESGSLKRPNEISGSQYGLNKLDEIDNSNMPREKLRKLDTTTGLPTYPNYE

EKHTPLNMSNGIETTPDLVRSLLKESLCPSNASLLKPDALTMPGLKPPAELLEPMPAPATIKKEQGITPM

TSLASGPAPMDLEVPTKQAGEIKEESSSKSEKKKKKDKHKHKEKDKSKDKTEKEERKKHKKDKQKDRSGS

GGSKDSSLPNEPLKMVIKNPNGSLQAGASAPIKLKISKNKVEPNNYSAAAGLPGAIGYGLPPTTATTTSA

SIGAAAPVLPPYGAGGGGYSSSGGSSSGGSSKKKHSDRDRDKESKKNKSQDYAKYNGAGGGIFNPLGGAG

AAPNMSGGMGAPMSTAVPPSMLLAPTGAVPPSAAGLAPPPMPVYNKK

>Dme-cycD-gi|281360953| ref|NP_523355.2| Cyclin D [ Drosophila melanogaster ] Gene ID: 32551 CycD

MDLLCSEIIVYESDPSLYRLNKRQQQMSSPAIIRDQSSICSSLGDAESDSHPDAQNNAVVCNMKELVYIY

ASVDSAAKNPEQLEPPPPPPPPPPPPPTATQSIQSYPRYISQEPPTSHCQRLDERLTTTADPPATDNVNT

AIGDPTLYSDRCLENFLKVEEKHHKIPDTYFSIQKDITPPMRKIVAEWMMEVCAEENCQEEVVLLALNYM

DRFLSSKSVRKTQLQILAAACLLLASKLREPSCRALSVDLLVVYTDNSIYKDDLIKWELYVLSRLGWDLS

SVTPLDFLELLMMRLPIGSKNFPDINIGKVRGHAQAFISLAAKEHKFAKFSASTIAASSIAASMNGLKWH

LRSGHNLHFLLSLMTDLTSVEQAQVRDCMLHMEDIFKEHSRNLEPFLVNIDPKEMSTLYYKRRFQIHQSQ

HLSQISIRPLPLPKAPAECVEQHQQQHNFGSAAPHRTHTCKMQAQAQAQNEIQDVTF

>Dme-cycJ-gi|24656859| ref|NP_523903.1| CycJ Cyclin J [ Drosophila melanogaster ] Gene ID: 38428 CycJ

MEQKVAAEQNIFVVDRKLKKTCPQADVERLAKTHWLTDYARDIFLTMREQELSRRPLFYLSPQLNERRRM

LQLLKLATSAHKLSRCALHLAVYYMDRFVDYYKIRPDKLLLVAITCLHIAAQIENTDAFIPRYSEMNRLV

KNAYTAFEYKAVERKILCFLNFELIRPTTASFVELFACSFLTRSDFKNYIEMLDEYERNHHTQPYQRYIS

FEEMLSILAQLLLRMADYTLYISRFANDLPSLLAAACIAAVRQVSGVRRWSEYLVGLTSYTEANVEPYMN

VLTDYYYYHVIQTDYGSPSVQTNQSLASPDSGFEESFTENTNLVVSDEVVTVETYNIITVQLQDPSPHSS

TFLPKEQTNLKRSRFEDDTENQHPLKHAKVESVAKD

>Dme-cycK-gi|28574303| ref|NP_788082.1| CycK Cyclin K [ Drosophila melanogaster ] Gene ID: 49816 CycK

MPCWYYDKKELRETPSILDGISFETERRYRKEGARFIMECGTKMGLGHNTMATGVVYFHRFYMFHSFRSF

PRYVTACCCLFFAGKVEETPKKCRDIIKTARGILTDNYFYSFGDDPKEEVMTLERILLQTIKFDLQVEHP

YTFLLKYAKCFKGDQQKLQKMVQMAWNFVNDSLSTVVCLQWEPEIIAVALIHLASKLSKFTVQDWEGRQP

QQQRWWDMFVSDVTMEILEDICHQVLDLYQSTQKEALQPTSPPQKPPSRADSPKSGKLTNSTGIGPLSVP

QQPPPPVCGNGGVLEVSNIQSIKSLASGVPIIGPSDSQNLLQGPTPPGQPSGYHIYHAPPPPPPPVVPMP

PYPSSGWGVQQTVVGHYSGSAAPPPPMPPSLPPGASGYYNAGGRPSQQRY

>Dme-cycG-gi|24651671| ref|NP_524609.2| CycG Cyclin G [ Drosophila melanogaster ] Gene ID: 43724 CycG

MSVPVRYSSAAAEYAAEVDCELESTLQQQQQLHLQQQYEQYQHYQYQREQDIAYYCQLQAARQQEQLMQQ

RTSMSSSVMPGLALPQDHQDHPAALLNGPHNNNIGLAMDAHSINAILVDDEQPSTSAQAAAAAAASAGGS

AGAGSGSGLGGAIGGGKLANGINRNAEMPTDWMRIADEGRYGTPGAAGLEYQKYEQQQQLEDLAESEAGA

VGGASNNNGESSSSLKKLEDQLHALTSDELYETLKEYDVLQDKFHTVLLLPKESRREVTAGGRDGSAYVL

RCLKMWYELPSDVLFSAMSLVDRFLDRMAVKPKHMACMSVASFHLAIKQLDLKPIPAEDLVTISQCGCTA

GDLERMAGVIANKLGVQMGHAPITSVSYLRIYYALFRNLAKEIGGDFFKFYQQLIKLEELENRLEILMCD

VKTTVITPSTLALVLICLHLDFHIKESYTRGSPELKHVFEYILFLQQYMRIPDRVFTCGFSIVSGILSHY

NGQNKAPYKQRLVWKLSSRTLRVLRPINRFSSDLPTIEEGIPNALDDGLRSRTESISSEEEEDWPTSPII

PIFEQC

>Dme-cycH-gi|17737725| ref|NP_524207.1| CycH Cyclin H [ Drosophila melanogaster ] Gene ID: 40429 CycH

MYPVSSQKRSWTFANEGQLMEFRVEQNSKYIESHEEEAQGRDLNEHFLTSAEERLLLKQYEIYLFDFCRR

FEPTMPKCVVGTAFHYFKRFYLNNSPMDYHPKEILATCVFVACKVEEFNVSINQFVNNIKGDRNKATDIV

LSNELLLIGQLNYYLTIHNPFRPIEGFLIDIKTRSNMQNPDRLRPHIDSFIDSTYYSDACLLHTPSQIAL

AAVLHAASREQENLDSYVTDLLFVSAREKLPGLIDAVRKIRIMVKQYQQPDREKVKAIEKKLDKCRNQAN

NPDSELYKERLRRLYTDEDDMPAEDASFHIADVSSDTSAMNISQ

>Dme-cycY-gi|19921156| ref|NP_609519.1| CycY Cyclin Y [ Drosophila melanogaster ] Gene ID: 34593 CycY

MGNKNSCCAYSSPQSDRKSKDMPPVFEERIIQLGHPPHTSQHQLDGHHGSASAVHLHHHHHGHNQQQNQQ

GGGGDNYENQQNLQHISEREALEGEEDPSVDPTAATMFLERSKVENGGMTRKRSQQQIAQQAGSGGGGGG

GGGGGSTPGGNSCGGGGGMKKSSSCSTIYLDDSTVSQPNLKNTVKCVSLAIYYHIKNRQSDRRLDIFDEK

LHPLTHDQVPDNYDTHNPEHRQIYKFVRTLFNAAQLTAECAIITLVYLERLLTYAELDVGPCNWKRMVLG

AILLASKVWDDQAVWNVDYCQILKDITVEDMNELERQFLELLQFNINVPSSVYAKYYFDLRTLAEANELN

FPTEPLSKERAQKLEAMSRVMQDKVTAEALKNGIKKWSSMDNISQGGPRRSVAILS

>Spu-gi|47550945| ref|NP_999646.1| cyclin A [Strongylocentrotus purpuratus] Gene ID: 373210 CycA

MAFSIGNPNASDIMANMAQGGQQFGRKTKRDDITSRNGPQACKRAALGTITNVSSTRVQPSRAAKQFRVS

GENSFPVFQDENAHSRIPQGKPFGIPSAGAAPAFSIHVDTTSYVQSSTSTSIKSTDKENEHILLDTALSL

PIPQAQRIPLRTFPDVEDNNVSLNEESLTSSEFSPMLLDTSLDAKCISPRTVDIRDLSLGEPEYAEEIYQ

YLKTAESKHRPKHGYMRKQPDITNSMRCILVDWLVEVSEEYRLHNETLYLAAAFIDRFLSQMSVLRAKLQ

LVGTASMFVASKYEEIYPPDVKEFVYITDDTYSIKQVLRMEHLILKVLSFDLAAPTINSFLPRFIKAAKA

NSKTEHLTQYLAELTLQEYDFIKYAPSMIAASAVCLANHTLNNEEWTPTMAHYTDYQLGDIYPCVQDLHQ

LFIKAPTMEQQAVREKYKSQKYSGASMTPVPTTLPTQ

>Spu-gi|75677617| ref|NP_001028696.1| cyclin B [Strongylocentrotus purpuratus] Gene ID: 576779 LOC576779

MAHATRNPNMTTLGFKKLQSFENENVGARLGGKSMAVQKPAQRAALGNISNMVRTTHAGGKKVVKKELRT

KTLAKSKATSSLQSVVSLPVDNVKTDICRSPLPEVVDQMEVDPIESAIEAFSQRLIDLQVEDIDKDDGDN

PQLCSEYAKEIYLYMRTLENQMKVPAGYLDREGQVTGRMRHILVDWLVQVHLRFHLLQETLFLTVQLIDR

FLVDHAVSKGKLQLVGVTAMFIASKYEEMYPPEINDFVYITDQAYTKTQIRQMEVFMLKGLKYSLGKPLC

LHFLRRNSKAAGVDPQKHTLAKYLMEITLPEYSMVQYDPSEIAAAAIYLSMALLGSEDNWGAKMTHYSMY

SEDHIKPIIQKMATAVTREDAMSEKYHAVKTKYRSNRFMTISSLSQLKSDFMKTLVEES

>Spu-gi|72065482| ref|XP_795905.1| PREDICTED: similar to cyclin B3 [Strongylocentrotus purpuratus] Gene ID: 591240 LOC591240

MPVNLKRNGGVAAQIREDKLPMITRAKRKSNEVGGIQGPSKKRAAFGDITNAFTSKNVLGQPKKGLAKKP

PLKSSIPIKVVSKVKVNAPKDPRVPPAETQKAVTAILPDSKPSTSSKEAGFVESMNKRNLVVESPSVEIK

DAVAAISLECNAGEEEQTRVKEEEEIEKYTDIDAENFGDPNLAPTYAAGIFRYLKEKEESSKIDDYFDMQ

KDITRHMRSVLVDWLVEVQENFELNHETLYLAVKLTDMYLAKCKIAKDLLQLLGATSLFIACKFDERIPP

ALDDFLYICDDAYSRNQFTDMERKVLKMVNFALGVPLSYRFLRRYAKCAHATLETLTLARFILELSLMES

SFITVADSLIAASALLLAFRMKNNGTWDVTLRHYSGYVEEDLKQCMNQLNSMLNSPPNKQLATVRNKYSH

PLFYEVAKIPCIDPLEL

>Spu-gi|115707311| ref|XP_790134.2| PREDICTED: similar to cyclin F [Strongylocentrotus purpuratus] Gene ID: 585206 LOC585206

MTKQSPTSLSSRATSLHVRVLRSNSKLPKTMLTDLPDELLIYLLKFLPMCDLQNMYRKFIDFFSLCSLYV

FYRVAELGNLEALIKLGVAYLYSEGVESSLCKDGSRAAKYFSSAEKLAPCSSPFTWLLIRPPWSNCGTCC

KARVFQGMKDISNESKEADRSVLFCLAKLHSFIEDMDPSMKKKNGHQSESRQWLKKAADAGSVAASYEYW

KNSLNDSTNDHATNLNILRQLREFASQGCLEAKLRLYSEYAKGKLGGVSQSHAREAMRGFVKESQPTKVH

TLLRSKNGLNSAMRYILVDWLVEVGSMKDYSCLTIHSAVQLVDRYLMARNISRSTLQLVGITCMVICSRL

LEDDIITIREAAWLTDGTYKYEDVVRMLGDVVATLKGNLRELTILDYLHLFCQVVSADSKMEYLALYISE

LSLLHADFGQYSRALIAACSLFLARLVLGADFPWPTPLVEHTGFEICDLVQCTLHLHNKCFLDDPVKDHR

DVTLAAVKQRFSEDLFLRVGELDIMVHPTLRTMLCVEEDEEVVDEEPADVSRDNSMPSSKDGMDAFLLMS

PSRKSRGQMKPNVAEYLHDDRSDRVSTPTHEFPEDEDPWLGVDVVPFDISDSDINPQITDKPREEAQPKN

DSRDHVATRSSASWVILDESQSVSVRTRTSHPSSTSSKGLSLTSGDIPSTSNCFLPLSTALSSSSSSLKS

KSQVTYTISASGRLCQQKVKARRVGGSPVSSRSRSVPQTSSLKTTPSKTVRYQFRQRTPVQSVKRKSMED

SSDEGAAGH

>Spu-gi|72015188| ref|XP_785047.1| PREDICTED: similar to cyclin E [Strongylocentrotus purpuratus] Gene ID: 579860 LOC579860

MSRRSGRLQSRQDNQPLSECISDENNLPMCTRKRKTREQDTTGVSKAEEVQRRRQQFTIENRWVPISESS

SIETSLLVPMQTKEPSTPSEELMDTANWVTFRNLFPAHVSDRASPVPLLHWDDLPEVWTIMTRKEALCPR

KHDCLKSHPSLGERMRAILLDWLIEVCEVYRLHRESFYLAADFVDRYLAAKENVPKTKLQLIGITSLFVA

AKLEEIYPPKLHEFAYVTDGACTDDQILDQELIMLMTLNWDLTPITVNTWLNAFMQICNAEEIAHRKTNF

HFPSYSSTEFVQVAQLLDVCTLDIGSMDFDYSILAASALYHVTNEEVTLSVTGLKWDDIAACVQWMSTFA

MTIREVGVAQLKNFKNIYAGDAHNIQTHCSSLELLDKSHEKQRLLREASCYSPVQVPGVLTPPQSDKKTK

KGVL

>Spu-gi|115727280| ref|XP_798281.2| PREDICTED: similar to Ccnj protein [Strongylocentrotus purpuratus] Gene ID: 593718 LOC593718

MAHLDQWHEDPELASEVYASLCHEESRILPFQGKSPQLNLRRFLVDWLAIVSENLDIESPARHLAVYLLD

RTMDRFTVSGEAYLQRLALVCLLIATKFEEKEIKVVKLTNLANQINTDETAKKEFFQMELLLLDFFDWNI

SVPTSLHFVDYFLMDAVGPHDLHGGKPLTNFDAPIYLERYAQYFLEISLQAAICIAASRICLQLSPTWTN

QLKKLTKYSWGQISPFIETMLKAHESDESAAKRNKGVKKSHHGYVTSAPTSITVPPTAC

>Spu-gi|115926275| ref|XP_001195649.1| PREDICTED: similar to Cyclin K [Strongylocentrotus purpuratus] Gene ID: 591068 LOC591068

MPCWYYEKEDLEHTPSIKDGIDPGTEARYRREGSRFIIEAGTTQKLRYDTMATGVVYFHRFYMFHSFKEF

PRYIMGAACLFLAGKVEETPKKCKDIIKIAKNILSEQHFAAFGDDPKEEIMTHERILLQTIKFDLQVEHP

YSYLLKYAKTFKGDKDKIQKLVQMAWTFVNDSLCTRLCLQWEPHIVAVGFLYLAGRLSKSDLMDWSGKSS

KSKWWEQLTEDISLDIMEEICHKLLDLYAAGQHKGPTSQGTPTKAPPPKRPKPKSAQDGSHGDPKAKMIK

QEHSREKQRHPPPPPPSAGTAPPVHPSQANALHERRQASPAPPPPPRVHTSGYPSNASTHKPHPGSSVLP

PPQPPPQKQAPPPQQQAQPHQAPPLQVPPYQAPQQQAPPPQAPHVPHQVPHPATHIVTHPVPTPVPHSAL

PHQPLQTQHHQPPVMHHQPPQPALQTAVPQVPPPPATVTHPNPYQQVPAYTHPNPPVSTYAAPQYSAPHY

STGSVQPPMLPPATQTQPVIPPPAPAPAPVSSSGHLPPSQPPYQIQGQFMNHPPPAAMPHAYRPPPATTP

AQQYHQPPPVQSAVPQQPPIYRPPPPRPAVPGMPQTTATMPLQFPPRQLPPPGQLPPNQAPPSLPNQYQP

PTPPIPTPTTQYRSHTPPVSHPVAQSQYSFSRSQSDETTPFPAVPSTATSAAPPPPGIVPNYPPPTSSQP

APGQPPLDKSMMSIAQEYAAQQLQSLGVPRNVAMQPVSGFVGQQQQQQQQGTTNQPPGTVAQQPQQQQQQ

QQLINNPRLRYTVCK

>Spu-gi|115712073| ref|XP_794154.2| PREDICTED: similar to cyclin I [Strongylocentrotus purpuratus] Gene ID: 589420 LOC589420

MLDIPPIKRVQLKELNSRLCSAINKERQSWKPLLIKDPQDSETEIGPNQRDEMVQWLLELNVKFRFCPET

YMLSVTLLDQCLMAVKARPKYLRCITITCFFLAAKMKEEDEMVPATHDFVRDSQCGCTVSEVLRMERVVL

DKLKWELNFVNGLDFLQIFHALLMTQRPTLLEDLHHMTPSRHLSMLTGRLSQCMTNHQLAACRGSTLALA

MLSLELEVLAEDWLPLTIVLQRMVGVDNQWLIRSRELITYTLFGHSASHRMPSTRPISPPNPVSPPVVNS

QKATPNKAKRKKSEIHENVYGNVKRLIDKGKCDMTLPTCPIPAISAQGGKTTVTPPGSCGKEVRQQDGDE

SLPSSHPPLTSIPVT

>Spu-gi|72089181| ref|XP_788820.1| PREDICTED: similar to MGC83944 protein [Strongylocentrotus purpuratus] Gene ID: 583834 LOC583834

MDAKAHLVKMMQVAVETEDFYQPYLEYICQQQEDSDCILPYMHDVILDRLRSLSRFFQLCPETFFLAVNT

MDRFLSLVKARPHHLMCIAISSYNLAIKALEPSESLISAEDLVRISQCGCSVNDVLRMERIILQKLQCDL

QAPTAHRFFKLFHAYSVVQGILGNDTAMQNTQLESSTQKLEACLCYFPLTLYKPSVLALALLTHELPNAS

CEVHNPNDIRWMRIVWDIQRVSQVSDNDLVSCRSQVSECLRMYSSPVSRMPHSRLTWIVSTRTARQLKFS

AQISSDLPPIPENRVLNSSLLSDGDTDESCEILCDVPDIQSSPVEVEVDDSDDDKSSSARTLVLNTHQLA

SLSTDDSICVERKLFTYEDESLAYCPTRIHGGS

>Spu-gi|47550981| ref|NP_999664.1| cyclin D [Strongylocentrotus purpuratus] Gene ID: 373245 LOC373245

MDNLLCFEQEYFDHAKSPMSYVDQVLLRDKNLDNLLAVEEQYVLSADYFGNHFQRELRPSMRKLVVDWMF

EVCEEQQREEDVFPLSVNYLDRFLSIERISRDKFQLLGATCMFLASKLLETIPLTSEKLIIYTDNSITLE

QLLKFEQLVLTKLKWDLMAITPNAFLEHIFHRLPVDKEQAALLRKHAQTFIVLCATDYNFAMQPPSLIAA

SGVAAAANGLRMHIPKVIDLLHRITKIETDYLILVRDRMENLLSKNLVPGSPTKTAGHTTVQKTRSMDES

DKPCTPTGVDEVEIITMPSGLC

>Spu-gi|115620254| ref|XP_790064.2| PREDICTED: hypothetical protein [Strongylocentrotus purpuratus] Gene ID: 585134 LOC585134

MADDDKPSTLAKSSEISYGNVIITLDNVLIPDAKLSQTPSANDGLETETEMDLRILGCEFIQMAGILLKL

PQVAMATGQVLFQRFYYSKSLVKHNMETVAMGCINLASKIEEAPRRLRDVINVFHHIRQKRNNKASEPII

SDQKYINLKNQVIKAERRLLKELGFCVHVKHPHKMIVTYIQALECESNTQLVRTAWNYMNDSLRTDVFVR

YTPETVACACISLSARQIGLPLPSNPPWYGLMGATDEQVEDISLIILRLYTRKIKSYEYLDKKVERCRMV

IQEAKLRAKGHLAESGANSQAGTPSSFSPHTSRPSSPKLNNDSPSGLPKMYKVNSNSIKEEAHSGSASGS

TKSRENGKADKHKRTPSSSISTRSRSSSRGRRSNSHDSAMDRHSSRDRSSRSRSTSPRNNRKRQSSSKRK

RDDHKRSRRDRSVSPKHRHGHKTKRNRTPPRGYSSRSRSRSRSRSRERRHKRHYSPYNDSTKESSHSRHK

SRSKHRRERSTSRSRSPISRSRKSSKRDSGRSHANDHRRR

>Spu-gi|72137687| ref|XP_797502.1| PREDICTED: hypothetical protein [Strongylocentrotus purpuratus] Gene ID: 592909 LOC592909

MDADGGEVFNIAREDLDKKTHFKVIHYVMEAGIKLHLESVTLASACCIYHRFFAECELNNYDPYLIGATA

IYLATKVEEQHVKLRDIINVCYRILHKEETPLEVGKQYWELRDSLVNCELLLVRMLKYNPKIGDLPHKYL

VHYLKSLSHWMDRDVWDQTPVCRTAWAMLRDSYHSDIALRTKPQHMAVAVMYFSLQCYGLEVPLNDEAAN

PWWKAFSEDISEEIIQKIVTELIELYELDDKR

>Spu-gi|115841184| ref|XP_787341.2| PREDICTED: hypothetical protein [Strongylocentrotus purpuratus] Gene ID: 582289 LOC582289

MYHTSSQLREWTFQSQEELNEKRAASNRRYREEHQAKSEGKDPATFFLSEAEERTLCESYEFLLRAFCKK

FQPPVPPAVVGTSCAYFKRFYIYNTAMDYHPKYIMLTCVYLACKVEEFNVSISQFCGNLQPEEQEKMAEL

ILSHELLVMQQLNYQLTIHNPYRPMEGLFIDIKTRFPAMKQPELLRKGAEEFINRSLATNACLLCSPSQI

ALAALVSSSARQDTNIDKYVTDYLMRGRDNPKDLPDIVNTIKREHGSKATSYFLFA

>Spu-gi390364883| ref|XP_003730706.1| PREDICTED: cyclin-Y-like [Strongylocentrotus purpuratus]

MGNKHSCSCLRSCHKNPTRRAHRTSSQEIERYEPSPEDIDVDERSNLQHIGDRETGIEEDVLDPSVHPRTSTIFLTKAES

LEVTQDRRNHHNDSRLSSVSKKFSSCSTIFLDDSTVSQPNLKSTIRCVALAIYYHIKNRDSSRQMDIFDEKQYPLSKTPV

PEDYDKHNPEHRHIYKFIRMLFNAAQLTAECAIVTLVYLERLLTYAEINITPSNWKRMVLGAILLASKVWDDQAVWNVDY

CQILRELTVEDMNELERQFLELLQFNINVSASVYAKYYFDLRELADTHELAFPLEPLSTERALKLEVFSKPTDDKPRDFP

PHTSVKRSRSADSIQVVPRFSNAIIS

>Nve-gi|156375154| ref|XP_001629947.1| NEMVEDRAFT_v1g188880 hypothetical protein [ Nematostella vectensis ] Gene ID: 5509415 NEMVEDRAFT_v1g188880

MVIDSDFSSDDEDIASSSPSRDQIHNIDSVAADPILGVPEYASDIFKYLKQAELNNRAKPGYMRKQPDIN

NSMRAILVDWLVEVAEEYKLLPQTLYLTVNYIDRFLSAMSVLRGKLQLVGTACMLLASKFEEIYPPEVSE

FVYITDDTYTAKQVLKMEQLVLKVLTFDLSVPTILNFLERFIKATNVPESMAPKVEALARYLCEISLLDS

EPFLKYLPSTIAASAIVLSLHTLGLSYWNNTLSHYTGFELHDLQTCIQDLHRSFAYAPNHPQQATREKYR

SAKFHSVSNLSPPDCLPLA

>Nve-gi|156364707| ref|XP_001626487.1| NEMVEDRAFT_v1g126046 hypothetical protein [ Nematostella vectensis ] Gene ID: 5505740 NEMVEDRAFT_v1g126046

MDMADFSEALNECFPTDVEDIDSGDYDKPQLCAEYAKEIMRFLRAMEEHYSVSPTYMNNQQEVNEKMRAI

LLDWLVQVHLKFRLLQETLYITMSIIDRFLAVHQVSKRELQLVGVGAMLLASKYEEMFAPEIGDFVYITD

HAYTKKQIRQMESLIFRKLDFSLGKPLCLHFLRRNSKAGAVGAEEHTMAKYLMELTLIDYQSIKFLPSEI

AAASLSLAMRVMGKGSEWTPTLEHYSGYSEKKLSTCMQRLAQLVLGARDSKQKAVYNKYASSKFMKISTM

SCLSTSTITTLAAQDQS

>Nve-gi|156380901| ref|XP_001632005.1| NEMVEDRAFT_v1g208415 hypothetical protein [ Nematostella vectensis ] Gene ID: 5511637 NEMVEDRAFT_v1g208415

MEKQKELTISMRAILVDWLVEVQESFELYHETLYLGVRVLDNYLMRSYVERENLQLVGAVSLYIACKVEE

RHPPCLDDFLYICDDAYQQKAFVAMEKKILNSLEFNINMPIPYRFLRRFAKVASADVKTLTLSRFILETT

LHHYKFIVHKPSFLAAACLRLALRMKGCDDWTPTVVHYTGYSVAQLDGCVIELNEMISEPPKQNLMTVRN

KYSHKVFHEVALIPPLDSLNL

>Nve-gi|156363375| ref|XP_001626020.1| NEMVEDRAFT_v1g128041 hypothetical protein [ Nematostella vectensis ] Gene ID: 5505144 NEMVEDRAFT_v1g128041

SPLPDLGWADSWELWSYMLEKDRKYTKDHLYLRQHPHLQPRMRAILLDWLIEVCEVYRLHRETYFLAVDF

VDRYLSVKKDIPKQRLQLVGTTALFIAAKLEEIYPPKLSEFAYVTDGACKEDEILQQELLMLQDLNWKLC

PITSNTWLNIYMQLHWLSRNSCEALKDHSNFNFVIPRYSQPEFIKVSQLLDICSLDIESLQFSYSVLAAA

AMYHVIPVSIEEITCHKREDLSPCIQWMGPFAATMRDQEPPCIRMFDQIKPEDSHNIQTHIVDLGML

>Nve-gi|156350442| ref|XP_001622285.1| NEMVEDRAFT_v1g237657 hypothetical protein [ Nematostella vectensis ] Gene ID: 5500914 NEMVEDRAFT_v1g237657

MDLLCCEGPRFRFAYKDPAILKDDRVLTNLLACEERYLPSCNYFKIVQTEVEPHMRKLVATWMLEVCEEE

RCEEEVFALSMNYLDRILSLLPVKKFQLQLLGAVCMFIASKMKETSPLTAEKLCIYTDNSITTEELLDWE

LLVLGKLKWDVSAVTPHDFLDQIFSRLPLDRSTLDVLRKHASTFIALCCTDDKFLLYTPSMLAAASVCAA

FTGLGISSHVSSRSWTATHLASLLHAITNIEPECLRSCQDLMEEVLHLSVKADPTRGKEAHTPSTPSTPT

DLQEIQF

>Nve-gi|156359300| ref|XP_001624708.1| NEMVEDRAFT_v1g41570 hypothetical protein [ Nematostella vectensis ] Gene ID: 5503756 NEMVEDRAFT_v1g41570

RDTVEDWASVSSYRSQIMRYAMALENKYQLPENFLEKQEEVSHQARAVLIDWLIEVHLFYNFPQDCLYLI

VALVDRYMSLRTVPVAHFQLLGMACLLVACKYEDRFVPTREELVAMADQAFDQSELMHMETRLLTCLEFD

LAQPLPTFFLRPIARASAIDLETYVVSKFIMEAAMLDAIMVTFKPSIIAATAFFMAR

>Nve-gi|156389378| ref|XP_001634968.1| NEMVEDRAFT_v1g99661 hypothetical protein [ Nematostella vectensis ] Gene ID: 5514810 NEMVEDRAFT_v1g99661

MAAVNSVSSDERWYFTKEQLQNSPSRRMGMDAERELSYRQQAATLIQDMGQRLSVSQLTINTSIVYMHRF

YMCHPFQKFHRHAMAPCCLFLSAKVEEQPRKLEHVIRVAHACLHRDGPPLNPESEEYLQQAQDLIENESI

LLQTLGFEVTVHHPHTYVVKGIQLVRASKDLGQASYFMATNSLHLTTLCLQFKPPVVACACIHLACKWCN

YEIPQSSDHKYWWQYINPTVTKKLLDEIAQEFVNIMEKCPSRLKK

>Nve-gi|156407073| ref|XP_001641369.1| NEMVEDRAFT_v1g81761 hypothetical protein [ Nematostella vectensis ] Gene ID: 5521580 NEMVEDRAFT_v1g81761

MVEECSSSSLASDIHEVLREKEARIPNFMAASPQLKIRRYLVDWLAVIGEKLGSSHGVVHLAIYYMDFFM

DKFIIQESQLHLLALTALLLAAKFDENENQIPDISTLNKFVNNTYQHAEYHQMELLLLEFFNWNIDLPTP

VQFLEYYLAKATIDYKESEMTIDYKKTSTYLRKYVYYFLEISFQDHTFLSFSPSLITSSCIAASRICLNL

IPSWTNELSKVTNYDWDKIAHCTEIMLR

>Nve-gi|156397054| ref|XP_001637707.1| NEMVEDRAFT_v1g91756 hypothetical protein [ Nematostella vectensis ] Gene ID: 5517695 NEMVEDRAFT_v1g91756

EERAARRKATYAYLKEHFKVTQFIMESGAKLSLPQNAMSSACVLYHQFWKGCDPKDFDPYLIGMTAIYLA

SKAEECPCKVRDVINVCYRSSHKDSPCLEINARYWELRESVVNCELLMLRVLGFRVSYDNPHKYLLHYLK

VLQDWTCPGMWERSQVPQISWSYLLDSHHIPLCLEYPPAHVAVALLHFAVECVGLEVPSQEAVRPWWKAL

CSDVTPELIQSITEDVMDMYDFENKPV

>Nve-gi|156405242| ref|XP_001640641.1| NEMVEDRAFT_v1g83332 hypothetical protein [ Nematostella vectensis ] Gene ID: 5520718 NEMVEDRAFT_v1g83332

MPNWLFTHDGLNRTPSRLDGIDYATECRYRREGTRFIMECGNKMGLRYDTMATGAVYFHRFYMIQSFKNF

PRWVTGAACLFLAGKVEETPKKCRDIIKTANSLLTPPQFEAFGPDPKEEVMIYERILLQTIKFDLQVEHP

YPCLLKLGKGLKGDRAKLNKLVQMAWTFINDSLSTTLCLKHRSEVIANAMLALAAKLNNYQ

>Nve-gi|156381346| ref|XP_001632226.1| NEMVEDRAFT_v1g167568 hypothetical protein [ Nematostella vectensis ] Gene ID: 5511895 NEMVEDRAFT_v1g167568

MFHTSTQRKHWIFKNQEEVQKLRENVNSAYKSRHSEAYPDKKNVKYLTVEEEKKLVEYYELVIVEVSAKF

QPPVPRSVTATAITYLKRFYVKTSVMDHPPKEMFLVCLFMACKVEEYNISVENFVQILPRDRREKVMDFI

LAHELLLMERLDFHLTIHHPFRPMEGFLIDIKMYLSEGKVNPESWRIKAEEFLLRAMRTDVAFHFSPSQI

ALAALSVGSTGGELQKYVNEKFGVTDKGTALMDTINSIVNMVTSHIVTVTKDQVKALESKLKTCRNPENN

PDSKMFKRRTSGNDENQVEQMEIESP

>Nve-gi|156365799| ref|XP_001626830.1| NEMVEDRAFT_v1g124800 hypothetical protein [ Nematostella vectensis ] Gene ID: 5506097 NEMVEDRAFT_v1g124800

LPEEVLIIILKFLPAQDLVNIRLVSTNLKHLVDESPTLWMTVSFPSIWPSQKNRAVLERAANVGNIEALI

KLGLAHLYNEGSNNTNASENGRQAAELFCTAERMTCDPFTWFFIRPPWAPSGSCCKACVFKNMVEYCSNA

EPCDSLNKSLLFCIGKILSLHEDEKRRSECIDWLQRASNLGSSHAAFEMWKMKSLEHALEPSAMLQSLRE

LRDIAMNGNAEAQYTLAMQYAAGNMGGASKDHAAEFLTQFLQKSKALNSHKLFGFQTELNNTMRYILVDW

LVEVALMKDFSSQIVHIAVHCVDQYLMKRKVQRSELQLLGITCILIAARFQGKDIVTIREASWLTDDTYS

YEEVVRMMGEVMSCLRGEVR

>Nve-gi|156359932| ref|XP_001625017.1| NEMVEDRAFT_v1g193420 hypothetical protein [ Nematostella vectensis ] Gene ID: 5504083 NEMVEDRAFT_v1g193420

MAANFWLSSHCNQWMLDVDEIMIGRQQDLQFLTEVEYQKVHIFYSNFMQSLGEHLDLRQQVIATATVFFK

RFYSKNSLKSIDPLLIAPTCVYLASKVEECGAISNNKLISASSSVVKNKYSYAFQMEQFPYRMNQVLECE

FYLLEMLDCCLIIYHPYRPLTQYVSDLGMEEAILPTAWRIINDSLRTDIFLIYPPYLIALAAIHMACVIQ

QKDSKQWFAELSVDMDQIVEITHHILRLYEIWKNFEEKQEIRGILNKAPKPKVRPSSTTPGHTPSPITTV

DT

>Nve-gi|156408249| ref|XP_001641769.1| NEMVEDRAFT_v1g35442 hypothetical protein [ Nematostella vectensis ] Gene ID: 5521900 NEMVEDRAFT_v1g35442

RDKSVCILLHINRHCGFQPETFALAVNLLDRFLSVVKANPKYLPCISISCMFLAAKMVEEDEAIPTAGNL

IGVSGLSCTPSDLLRMERIILDKLGWNLSAVTPLQLLQVFHALCVSKGYLDNCPVSEHLHHITLKLEELL

CNHKFTFFKPSTLALSLLSCEISSLTNVWIEATIMLQDMAQVRL

>Nve-gi|156365937| ref|XP_001626898.1| NEMVEDRAFT_v1g124606 hypothetical protein [ Nematostella vectensis ] Gene ID: 5506187 NEMVEDRAFT_v1g124606

MAVNGAQSEKTYAKVLITLENCILSPDKLTETPSVKDGLDKNVEEDLRIIGCEFIQTSGLLLKLPQVAMA

TGQVLFQRFYYTKSFVKHDVEVGSCTCYYCTSKRNGQIKATKHRIVVVQYASSVRGWLFYLNRPIQPLEY

MGNLYFNRKNQVVKAERRVLKELGFCVHVKHPHKIIITYLQILECETNQELAQLAWNHMNDSLRTSAFVR

FAPETIACACIFLASRLLKICLPSNPPWYELFDAQLSDLEVTFLILYTCFQVSLDSLQKKVAVLQKELED

KKKKKEGEEKDG

>Nve-gi|156406586| ref|XP_001641126.1| predicted protein [Nematostella vectensis]

PGKGLKKCNSCSTIFIDDSTVSQPNLKSTIKCVSLAVYYHIKNRDPEVRTVDIFDEKLHPLMNDPVPNNYDKIDPEQRHI

YKFIKTLFHAAQLTAECAIITLVYLERLLTYAEIDVYPGNWKRILLGAILLSSKVWDDQAVWNVDYCQILKDVTVEDMNE

LERAFLEFLQFNINVPSSVYAKYYFDLRSLADANDLMFPLQPLSKERAKKLEALSVITDTRLDDLASSSLKRSASLDHLN

PHRGSFAIIS

>Aqu-gi|340374274| ref|XP_003385663.1| PREDICTED: g2/mitotic-specific cyclin-B3-like [Amphimedon queenslandica]

MERAKRTSSLKALQAIKENSHAGRNAAKLKKIESEALAINPTKRHSSPTAFHPPEPKRNPLGNVTNVSTN

ITIKPKQELTSISDNEDLYEQAPRRSKRLLRKNSSSPIPEEPLALTDSLICSESLDTTPMTDVQNVLRAT

TLDDKLEESQLWKDIDEAESHDPLFSSEYAPDIYQYMREREVKFKVSSYLDHQPLINSSMRSILIDWLVE

VQENFELFHETLYLAVKIVDRYLEKKEVKKEYLQLVGATSMLIAAKFEELSPPLVDDFIYLCDDAYQHDE

LLSMERNILATLEYDVNAPVAYRFLRRLARAAGADMETHTLARYICESTLQEYEFVSDDPSHIAGAAMYL

SIRMKGLGGWTPTLQHYSQYEASNLLPMVQRLNDLISRPAGNTSTVRSKYSHEVFHKVALILPLQDVHED

YLLSLEESS

>Aqu-gi|340380414| ref|XP_003388717.1| PREDICTED: cyclin-F-like [Amphimedon queenslandica]

MSCHAVTSFMDLPDEVLAHILSYLPLLDLYNTRLVSARLCDLVEDSRYSHTLWASACLESTTWPSYRNLS

IIERAARHGNIEALIKLAVAYLYSQGVPDGPGKGTINAFNGQKASNYLKRAESLVTHRQPFSWVFIRPPF

ATTCVCCKACVCHNMKNHCISPNKELIPSLFYVVGKAMSHYENHEVEERRQAYQWFKQGSLHGCPYSSFE

NWKTEQCFQDEVQSVRQLRKIVTSRDTCIDAQFELGKLYAKGKFGGITRFHAYQYVQQIVRSSPSLLSAS

IYDAQPDVNEEMVFILLDWIVEVAEMKSFSTKTLHLAISLIQRYMVARKLKRSRLQLLGVTALLLAARWT

AVPIITIREAAWLTDNTYRYDEVVCMMGEIVSTLHGEIQKPTVPDYLEMFELLVNADKKSSCLAAYVSES

AVLFPDFGRYTAAQIAAGCLLLARVLLEQELPWPSALVEATGLTVPDLYHCTSLLYSKCLTDDGVVTDYR

GVKLCAIKTRYSDERFLCISEMEFMNYYMMKDKLGVRPYYQRRSSNSSLLKYRELVSQSFVEEFDAQSLS

DVSFVTSPLPPIVPSSADGYSPRASIDSFVPSPIIFEDSNSCSSPSPSSSSSSSSPTCERQERPVHSIKK

ERRKKKERRHSSNKLLSSSLPLLPGIEPLVISEETVGFNSSLMVTQRNVHTPPTSRRKSLLQHWNSTIKA

SSNHGHSPIKANPVRKGQRRPLSSLASSHCSLSNVSPVCNHEEGEAEMNERGLEPPHVPRPASSREERED

GERSVHNHQHKALQVEKRAFSDQNLLLTHNKRHRSFKSNNCM

>Aqu-gi|340379787| ref|XP_003388407.1| PREDICTED: g1/S-specific cyclin-E1-like [Amphimedon queenslandica]

MYMEPFSLSCKENVPPPVGLTLRQGGGVLQTLSTSHQTPQTPPPPLSSSSGHEVLKGEEKKKRINSSSLS

SPSPILTRGVAPMLARVAPSPITFLPSPLPSLSWTDSTNLWREMRLKDTSQAAPGTELRLRHPSIMPTMR

TILLDWMLEVCEEYRIHRETYYLSLELFDRFMDTQTNVQKEQLQLIGVTCLFIASKIEEIYPPKLADFAY

VTDGACNSEEIVFMELMICKALKWRLHHCSVSVNTWVNLYMQLVSSYFRPHGLKAREFEYPAFSPFEFIR

VMQVLDLCTLDITSRQFCNSILAASALYLVSEKCQMHLNLVTGFQLADIHVCVQWLNAFVSVINRMAQPV

QKAFRGVILQDAHNIQTHEVNINMLEEAQDIIKYQEMIADTAGGGRGLKSNQQVVPMDCSALMTPPRQER

RCLAPINSTTTIV

>Aqu-gi|340376468| ref|XP_003386754.1| PREDICTED: prelamin-A/C-like [Amphimedon queenslandica]

MSQQQATSSPSLPTPERRTRMKEKEDLQLLNDRFVTYINRVRRLRDEKEKLNVTLEHMQTTTQEESGAIK

RMFEKELQDARLLIDETAKEKARYQIQSSKTEDRIKDLETELKEFREDYDRMQQELAEAKKSAETFESLY

KSSLVDNQSAKQKIQDLEEEIDDLRARLREVQDSLEQETLAKVDIQNQNQSLREELAFKKKVYDEELLTI

KRTGLTTVHRDGVEETDDDFAERLQAAIDEAREEIEKETEQFKLDLEKSYKTRLESLESQSQRDASARVR

LDGELKNCHTTLAKYNRDIAKLQEKNEQLESSLREKSEDLARAKQLHSAELANLMDEMRALRVNYDQKMK

EYEELFDLRIQLEQEIATLSALLQEEEQRLNLQTTPREKRPRVARDGEGPRSKRKKQVAATSSSAVGFIQ

IIDVDPDGRFVQIKNMSDKVTEIPKNKLQLVGVTAMLIASKYEEMYAPEVADFVYITDSTYSNTEIKAME

RNILKTLDYSFGNPLCLHFLRRNSRAGDATPQMHTMAKFLMELCLPDYSMLEYLPSLVAAAALYISNKLY

SDGEWTPALRHYSQYTEPDVLPCVGKMASLVLSMHTAKQQAVKNKYCSSKFMRIAKEPCLQGKIMKELAS

VSLSQ

>Aqu-gi|340379451| ref|XP_003388240.1| PREDICTED: cyclin-C-like [Amphimedon queenslandica]

MAGNFWESSHFKEWLLDKQEIEVWRQKDVSYFSSSEDYQKLMIFFANFIQTLGGEQLKLRQQVISTAIIY

FRRFYSRHSLGDVDPFLLGPTCLYLASKVEECGVVQPGTLYIRCKSLIRQKYQSIYNQDYSYKAQLIMEC

EFLLLEMLDCCLIVYHPYRPLTQYVTDLGQEDILLPTAWKIVNDTYRSDICMLYPPYLIALVAIHMAAVV

HKKDVKAWFAELSIDMNKIIEITNLILDLYKMWKSYDEAKDVPELLAKMPKPTKCGSPGEPTPSPNK

>Aqu-gi|340373128| ref|XP_003385094.1| PREDICTED: cyclin-K-like [Amphimedon queenslandica]

MELEEKDPSPEDIKWIFSAEVLEHLTPSRKKGISHEMERRYRREGARFISNTSNTLKLRRDTLATGTVFF

HRFYMVQNFADFDKYVVAAACVLLAGKVEETPKKCKDIVRVAKRFLSAEQSKSFGEKPLEELISFERVLL

QTIRFDLQVDHPYGYLLKFAKHMKGEKQTIEKVLQMAWTFINDSLCTTLCLQWEPPVVAVALLYLAGKLS

KFDLQSAFQAKSRSWWRQFVLTVDAHDLESICHQVLDVYSEEEKEQEAKKAKTVIQGDPVPPQDNASSPP

PPPPPTNHTHHKQHITPLKGPSSGPSTSTASNSGSYMTGTSCATGIVHGAGMVLPPGQNNMMLPAPPPPH

SMLHPTPSGSSVNSSPMTNNHYHSSLLGQPPQPFMPHGMNYTQSPSQIQLSAGGPVGMGSQLNPGYPSIP

PPSNQIYGSNSMGHAIPNLPRPPLPPIPPSSSLPNNYHPTSGWR

>Aqu-gi|340369567| ref|XP_003383319.1| PREDICTED: cyclin-J-like [Amphimedon queenslandica]

MTCAVDSSSGSYPHPQPTYHIPYYYQAHHHPGQHQEEVVLLEDNWLQTEVYHDFVLPAAAVAGGSCCSSG

CCSSTTSLTPTDPTALLYRPQNKLSSLGAGTKANSTGPKAPPWWESELSQPIHETLKKREAAISSIQFKS

PQLHLRRELVEFITAVSKDLGLSDGTRFLAIRLADQFMDGHNVMEYRLRLMGLTCLLLAAKSEEIDDNVP

SIEMLQHAARMNDSSNTSAYSRQEFHTLELYILKYFKWCLSHPSVAHFIDYYLHTSLKGDENLPGLSKWE

AIMLENQMKEFTAYFMEVTLRGIKANSLSRHFLINCKDAAGILPAASCKWLVYFKTYIVLH

>Aqu-gi|340380250| ref|XP_003388636.1| PREDICTED: cyclin-T1-like [Amphimedon queenslandica]

MAAEREEAPPRRWYYSREQLSNSPSRADGVDPEKELRYRQDAASLIQDMGPKLNLNVLCMSTAIVYMHRF

YMINSFKAFDRVLLATAALFLAAKVEEHPRKLEHVAKCSYSLVNRDKPDRLDLDVQSEVYTKLIDDITYH

ELVLLQTLGFDVQVKHPHPHVVQCMNLVGVSRDLSQAAFFLAHNSQLLTTFCLEHPPTVVACMCIHLTCA

WKGLEIPRSSDDKNWWEYVDRSVTYDKLEGLATEFLNIVDKSPSRLKKRIQDNIRMAVEGGRPLASSSQG

TSRHPSTPATPSLHHKHKDRPSSSQTRKQQAPPPSSGKSHPPVSQKIQRSSPPPPPPPIHGDRPPHSLPP

DQQKMRKHKEAYHHHHLHHQNNSGGAGGMVNKQTTPSSNQHKHSLPKDSHYPKNKKIKMEEASSQPSASS

LSKQSLSLSDYRQSKHHHHRHHGNSDPNRPPKQDRHHSSNHVSNKHHHTLPPLPPPLPLSEAPPPPPPPP

PN

>Aqu-gi|340373665| ref|XP_003385361.1| PREDICTED: cyclin-L2-like [Amphimedon queenslandica]

MDPVKVFDRIYLSSSNLLVPKERLINTPSRKDGMSRDLEIDMRVTGCHYIQSAGILLKLPQVAMATAQIL

YHRFYYAKSFVKFKCYYTMMACLFLAAKLEESSRRLRDVINVFHHLRNKRQGSPPVVMDYVGEEYFRLRN

LIIKHERYILKELGFCVHVQHPHKLIISCLQILELEKNTPLIQKAWNYMNDSLRTNIFLRYNVQTIACSC

IYIATGHLKVSLPLQPPWWELFDVNYTDMKTISLELIALYQREIKKLQELEKQVDQLQQLLISKKDSNKE

MTPSSQTVSPAHLGVMSQDSQEKSPKEMSQLTVSRSDKEEAPPPTTSNELKPLNGHKRPLSATPPLSSTQ

EKRSKLDNHDTKASSKPIISPIKLSSDSEDESSRYHEVHKNDKSRRMKSRHHERSGRHKTERKSRKREAR

SNSRDRRSWSRERSSSRERWRERRYHHYHRDDDYHIKHYRK

>Aqu-gi|340370065| ref|XP_003383567.1| PREDICTED: cyclin-I-like [Amphimedon queenslandica]

MASPSTPPSPSPRVTDWVSLRRQSERSMEGELQQLRAVLETESKTYTYSQAIYHIEENPRSYKYVTARDR

DEQVSWFRNITHDLSFSISVFYQSTLLLDTFLSTKKTKREFLQTMAASCYSIATKLVESCDSVVALSHKL

VSKYYSGCSVTDVEKMEAFLIQTPDIASARSLHTIQDYIKKFHRLAERSEMFSLPQSLTLSSHLCHLYKC

AQHVVCNHELMKYRPSMLGLAILGCHLKQLNCDWLSTLLYLEEILQIRGGELSICYEAVAAYFPTYFAST

PLPRLPSSQLTPSKPPRLQKWCDSSGSHGDNDDDEEDDGTHKEGVIKEKKVIRRKNRRRYRPPTSRTINV

AIV

>Aqu-gi|340377763| ref|XP_003387398.1| PREDICTED: cyclin-H-like [Amphimedon queenslandica]

MFHSSTQKSHWLFQHRQQLTRQRAETNASFCRKYSDAAISKECQFLTPTEEESVCLYYMKKLFEFCNVFR

PPVPRGVLGTAGAYFKRFYLLTSVMDYHPKEIFLSCAYLAFKIEEYNVSLDEFVYMLSPELRQSSSEMIL

NNELMMLKRLKFHLTIHSPFRPLEGFLIDMKTRSSIPNVERLRKEADSFLMSSLYSDVLFLYPPSQIALA

ALYYASTVIEVDISSKRKSDEVSEVVLRKKIKEEHDVEETGLTSLTGFGSLT

>Aqu-gi|340376480| ref|XP_003386760.1| PREDICTED: cyclin-related protein FAM58A-like [Amphimedon queenslandica]

MADSAAGTEDKNHEKSGYSPVLEETLFVAKAGVSWLRLRMSEVAIATALQYYHTFQSTMEKNRFDENLVA

MACLFLAAKAQEVSLRLSDLVNTCYHILHHDKPQLEVSSLYWQLKESVAKMELVLLRALKFEFQLDLPHR

YLLHHLLSLSHWVEPSQWHSSHVTRLSWSLLQDSFHTTLNHIHPPNKMAVAVLYLAVKVSRLVIPSPGSR

YQWWEVMCPGVTEPELQTLCEAIMNLYQNEK

>Aqu-gi|340378990| ref|XP_003388010.1| PREDICTED: cyclin-Y-like protein 1-like [Amphimedon queenslandica]

MGNCCSLGSDPPPPPSPPPELTNSHNTAPAAPMMIPPDTPPILATQELDPTAISQTLDHISDREDNSNKDETMFLKLSHA

AMKMMKTGRHRHSSVRMLASPSSKDSPLVIKKSNSCSTIYTDDSTVSLPDLKFTLKCASLAVFYIVKGRPRDRPPKTLDI

FDEKLHPLTRDPVPDHYADFTPDHKLIYKFIKTLFHAAQLTSECAIITIIYLERLLQYSELDLHPCNWKRILLGAILLAS

KVWDDQAVWNVDYCQILREITVEDMNELERVYLEQIQFNINVAAKMYAKYYFDLRTLSEENGLTFPNEYLPLTKERALKI

EALSHAADNKVVPGRRVRRSQSLEQISPKNTLILS

>Mbr-gi|167523717| ref|XP_001746195.1| cycB cyclin B [ Monosiga brevicollis MX1 ] Gene ID: 5891326 cycB

MPAKTFGQAQHVKRAPLGDIGNNAAALGLNQAKKVKSQERAGPMTRAAARNASSEPMPADDQAMETQDET

MHDIRMPTLDGPAPALALPAGVENIDEEDTENPQMATEYVADIYNYMREMEVRLCCDPAYLQSQPEVNER

MRAILIDWLVEVHYRFELLQETLYLTVDVLDRFLSSERTSRSQLQLVGVTAMLIASKYEEMYPPEVGDFV

YISDNAYRREQILAMEQTMLRVLDFNLGKPLPLHFLRRDSRAGHADGTMHTFAKYFMELTLCSPRFLGYK

PSQVAAAATYISREVVGEQQLWTPTIEFFADYTLTDIMPVILDMKAILRESPTAKQQAVRTKFSRSKYMR

ISREPMLEKYISEL

>Mbr-gi|167517989| ref|XP_001743335.1| cycA cyclin A [ Monosiga brevicollis MX1 ] Gene ID: 5888686 cycA

MRKQRDINHTMRSILIDWLIEVTEEYKLTLQTFFVTVGYVDRFLSEMAVQRSKLQLVGVTCMLLACKYEE

IYPPTIDDFVYITDKTYSRPQVMKMEHVILKVLRFDMGSCTPLTFLYYFLNAIPHHDDTKWLAQYLCELS

AYDGRRSLGQRPSTTAAAAIVIALHTFELHPLPPALVSVIRQGPEELQAAVNTLHEIFSVYPNLQHEAIK

EKYSTARFNRVAEVPPTRHAPILFT

>Mbr-gi|167522783| ref|XP_001745729.1| MONBRDRAFT_37010 hypothetical protein [ Monosiga brevicollis MX1 ] Gene ID: 5890801 MONBRDRAFT_37010

MAGRGAAAKEQPESTREGTAASAWVSSLAGGVAGSASRFATNPFDVLKIRMQLQAEPIRRGQAAGKYQGV

VHCLRTIVTEEGVQALWKGHLASQLLTITYCAVQFPVYETLVAQTRTHLTNQALAARQLVLLDLPDDALI

RIFRHLSYRDLSVLAQTCVRFARLSQLPSLQCHASFNNEWPIPRTMPAYQRAISHSNLHATIKLGVARLY

GEGVDANDVEAAHWLIQAEAVLPPCAPFIWMLFRPPWSSDSCSKAKIYKFLRKEVDEFTGGEYERTCTIG

RLAYCVGRTLELAENPQMTEAERYYRISLDCHCSHAALRLVDICGRIDANFSTLDRMALCLKASSLDNDF

ADLALIGNYVNRDYGNRTPHECMAFSREAFARGAAPVRRGGLRQPDLDREGKMRFILMDWLIEVADLKTF

GGETLFVAMDLVDRFLQHCRITRKTLQLLGIACMVMAARYLEEGVITIREAAWLTDSMYSYDQIVRTIGQ

VLVDVSGNVIRPTTFHYLNLLLQIGGATPAVFLLGQHMAEALILTIPLTEFPPAKLAAAMACCTFALAGV

PQPWSSTMERWSGLELRTIYDLAIKCFSLFRKQERVVDHRGTELRAVKDRYNRQVSIKDVANLARESTLS

LEEFQAMLDENMELDRDSKREAELTKRAENRRLAAEDEAQTSKYAQLKSANKKDKNAAFRPKAKREKEAA

LAAAKERQKQGGAVAEQSDLTENLNRLSMEGAATNVDDALRVLSGEASGSSVEKMTFSEFEAQVLPELKV

CRCVPRKELMPIG

>Mbr-gi|167526102| ref|XP_001747385.1| MONBRDRAFT_9572 hypothetical protein [ Monosiga brevicollis MX1 ] Gene ID: 5892600 MONBRDRAFT_9572

MTTSALAALNAQAITSEVHRNMRVQESQLQLAQYCGPLADLRPRCVQRIHRLARAFRFHRLTRDAAIFYF

DRMLFLFHMHESHLELAVQTAFLMAAKCQEAEEVVPTHHDLHRAGCAVVPTAHLKAFEASYLERVDWILT

SVTPSDFLDYYARFSISSQDCLAGLPLHNVHNVEAFTLDLADQVLQEASMANHVATFLPSHRAAAAITTA

RLIVDIKPAWSPTLQAVSGLTWREISPCVDAMLQLNLNPSCHREMAALRQCLQHQEAEPACRSPHAERTL

SMDSGIWSAPESPMCADDSVFDDPLLESSHVHPTHTQRETQPVHQLQQCSTRHKLDPAAHHLPHTPTKQF

RVTYGQPAVAHAPVRALRLGAHVPLQRPPTDQLLTRPVARYPDSLLARAFRDLIV

>Mbr-gi|167524110| ref|XP_001746391.1| MONBRDRAFT_26082 hypothetical protein [ Monosiga brevicollis MX1 ] Gene ID: 5891585 MONBRDRAFT_26082

MTSIYHGISPTPSANALYFAIAYLQRYAVTTTLMEHHPDLICLTCWLLAVKASEAKVPLHCLVAVAQRQP

HTPLAQLAAKDVEDQVRQLESSLAQKLNYEFYVSLPSRAMRAILIELQGVTDPAPPAVDDEKSIMAALLN

LWASPAQLQMAPVQLCLAALGRVRGVAAVAAYLQRVQPEASSEALARAEAIAKKVEAAAGTPPPDSDFMR

LLDKVDKCLNPFLDPRTPEFATLQKLKREKAKRSAEAAA

>Mbr-gi|167526156| ref|XP_001747412.1| hypothetical protein [Monosiga brevicollis MX1] ccnL

MAQAEQQNVRIEKHVFPCLDSDLPRFDLANTPSRRRGIPAELELRLRIAGCELIQKTGMLLGCKQVVMAC

AQMLLQRAYCRLDISRHSLQWVGLACLFLAAKTEEDHQRLRSILLVGRQVAHRMTREYAEKQTELAPMIV

GDDDYHELKNNVIKSERRVLKELGFCVHLKHPHKDVAQLAWNYMNDALRSDVFLRFEVAVIACACIDLAT

RKLDIPMPDLWFQSFGVHPDDFEQTCATILQLYRQSPVYLDDLARELELALQGEDLTDPRLAAPKKHRGR

SPQREGSPARVMHMADQRHDRTVIVTSVTKIAAVMSAHMGVEAVGVDSRFG

>Sro-gi|326426811| gb|EGD72381.1| cyclin A [Salpingoeca sp. ATCC 50818]|PTSG_00400.1

MNPHQPADLATSEPAPTRQPGGSKRLHDERADGQKLKRSALQDMTNSTTSGQTASRMAGATFTRRTHIQP

DTTGLPELAARREREERQQSRPATTAATAMPDLRVQQDDQTLPPRQPTQHMTRHPVAFARQDVRPTPALT

HFHPPSQTTTTTTTALPAATRLSRGSSAAFTQARQHQQQQPLPQGPAYDRAYSHPSVATASTCAPATTAV

VPPVSMPVPVSRTTSSSSTSSSSSSTTLASTSASLSAAPSTTAVMPNHQHHTSTSVASVVPQQQGLTPLP

ALLGSADFVDIDRPYAHDEGRVTEYVEKVMTYLRHLEKKFRPHAGYMGRQRDINHNMRSILVDWLVEVTE

EYRLQLQTLYIAVGYIDRFLSNMAVQRSKLQLVGVTCMLLAAKYEEIYPPSVNEFVYITDNTYRREQVLK

MEHVVLKVLRFDMGACTALTFLVRFIHAASATPPSHCLALYLAELSLLLGNKFIQYLPSVKAAAAICLSQ

HTFARPVWTPTFERYCRLSPEEVQPCLNDMFEAMTSAPHLEYQAIREKYMERRFHSVAGIAAPTSCPRLV

DLRGGSQV

>Sro-gi|326435281| gb|EGD80851.1| hypothetical protein PTSG_01437 [Salpingoeca sp. ATCC 50818]|PTSG_01437.1

MTDRAKRRHDESGIDIPEDDDALDLTPSSTTSHRRMSKAGRAHLYHKFPALQRGKQQPQDEAIAGDKENA

SSPKVVAVPSSSTTSRSRSQAGRSARPRPVTSASNTRRPQKQPRSSAPVPASREAPPQHQPSQDVPHSDV

MSLNYVDAIYAKNLQREEKYLLNPRPSRGINLAMRTVVIDWMIEIQVSFKLRDETLFCAVDILDRYLAAR

PDEQRHDYQCCGATSLWIASKFLEVLPPELADFEYVCAGLYPRQAFIDKELTMLTALRFYVMNVTPLDFI

SVYAIVLQLSLEGMALAEYLITLPLQEQRFYGLRPSVRGAAAVHIASKTCDGPGWSEDHSALFKLDHRHI

MLVAQQLRGLANERPEKYINCRSRFLDPAKFGVAGRNLLV

>Sro-gi|326437566| gb|EGD83136.1| CCNYL1 protein [Salpingoeca sp. ATCC 50818]|PTSG_03772.1

MGCRISQHRPEMSTHKGQYDDDERVEHISDRQDHLDHEIQVLHNVLPLSSEAPSPPSRKLSRSTSRRFSV

AEDDLQPLELANSRKFNSTSTLFVDSTVSSPNLEETLRCVALALSYVVEDGHKQDNPRLFSEKFDEKRHP

ITDRVRRDYASRIPSENRIYKFMFQLFNSAQLTAECAIITLVYVNRLIAYTSLTLHASTWKRVVLGAILL

ASKVWDDQAVWNVDFCSMLPSVAVEDMNDLERTFLEMLDFNIDVDSCVYAKYYFELRALAEKFEKDFPLK

PLNKDQAKRLEALSERRHDLMKGFGMRGAQSLDPDEFRAKAIIS

>Sro-gi|326437558| gb|EGD83128.1| hypothetical protein PTSG_03765 [Salpingoeca sp. ATCC 50818]|PTSG_03765.1

MTQMKPTTKPRRRSIREVFEARLGRSRTTGRRASTGSHTGRSSDGADHNNNSPTTNNTTTTTTNNNNNDT

INNGVTRSARRSTSDRSDRLGIDRNWRQPGPPTTITTSTSTSTTGRATGVPTAAAAAAAATENTQAALAT

TRALTTEEGDLTPKRAHMSVSALSSRPRPPALALVPLELTTPRHKVQARMHPLPGLGDSRLAASPASTPA

TTPTLGSATPATVTATEATVEASSAVASSASSSSTSTAAGAATATPGARSQHHKITVRMADASLLTRRMR

YKRSVSLGDVPLARAQTPKPLSLQNKPGRVTKASSVSTAQAAPSQVSGASFKPSSSNPATAATTAAAAST

SAGNRSVFFPTPSPSSTSSPSASLLPFPLKQAPSASSHRSTTTHTATLYPVAIKFPEAQQYYRVLEQRLA

SCEVSINALKMQPCMPPRLRARVFHWLADVCDRARMTLDTLFFAITYFDTYCSVRHGAVTVANMQLLACA

CLRLAAKIEETRVPSLRLLSRLTDGACQPTGIAQFELDLAAVLKWRLIRSTPLHWTRFFIGVALGDPSTL

VGSAPSCVDPAWLFRSCQVLQLAMSDAWALRFDARQMAAAAVLLSATRPIDILAVTGLDKCALRTCLRWM

HCFARVISEQEAFPSTLTRVLDEPGRVHCVTWISTATTLRKAQALVKTAFRQEPQACPPANSREPGLPTT

STVLVRSTPPVKRVAACGVVGGGDGAANSALDEEVQAAFSQPDASEEGVGVAATASAVSTAPARAIAVGF

CHDGSSCGASGLKQRGALVRQRASALSSTASSSASSDNEGDDDEDDDTDDDDALLVRAAGSH

>Sro-gi|326437989| gb|EGD83559.1| hypothetical protein PTSG_04164 [Salpingoeca sp. ATCC 50818]|PTSG_04164.1

MDTDQASHHGTVPEGFFTNMPDELTSNILAFLDMKDLCQVAQTCRKMNELAYTRSVLSKVSFEGLWPTKD

TADAFARAADAGNNEAALKYGIACLYGKRVDSDATTALKYLLQAEKLCAGTTPFVWLLFRPPWTNDTCSK

ACVFQQMHAMAEDPSTDPHLAGRMYYCVAKTLELHEERDEREVVEALQKAELMHCADAVHDLFVKDPTAY

NDDEEHSFTREKRLLQAARECPRLRFAAIRHFTRAREMYEPKKRELFRETVELGMEKLRLRSDAPPPAQP

ELDSQGKMRFILVDWLLEVASLKMYSIDTLHCAVDMVDRYLATRTITRRTLQLLGITCMVIAARFLEQDV

VTIREAAWLTENTYDYEDVVQMVAAVLAVADGHVRRPSPNDYITIFAELSNVPVHIRCFMDYVSESCLLH

QPTLTHAPAALGAAIYFVSMHLVGCKSAWPSSLTSNSDLRVSHFQEEIMEVFRCLFVREKITDHRGLELR

AVEDRYTRALERGNMLHLRFTLESQTSDNLQKTIDRHIMEERRMIRSSAQTPTPPLSKMTSLPLHEITNV

PARPPKPQFSP

>Sro-gi|326428978| gb|EGD74548.1| cyclin B [Salpingoeca sp. ATCC 50818]|PTSG_05912.1

MSSLMRFGMPRSAAPKDVVEAPAAHRKERKALGDISNRVTDNINLTKKPKLVHKAPRGMEVDQDVEEKHA

QNVMPAPRPIPANVEDVYEDDFENPQMVAEYVEPIFEYMRELEVRLHVPANYFKIQTEINARMRDVLVDW

LAEVHHRFELIQETFHLTVHLLDRYLSKEPVTRDDVQLVGITAMMVAAKYEEMYPPELGDYVYITDKAYS

EDRILAMERKLLRVLDFSLGKPLPLHFLRRNSKAGHADATMHSMGKYMIELSLGSHAMLKYVPSQLAAAA

TYISREIVGEHELWNPTLEHYAKYSLEDIAPVVHDMRAVLKHSTVSRLQAIRNKFCRSRYLRVSKNPQLN

EYIERL

>Sro-gi|326429298| gb|EGD74868.1| hypothetical protein PTSG_07096 [Salpingoeca sp. ATCC 50818]|PTSG_07096.1

MVAWLTKEQVADTPSRKDGIDADREARYRRECIHFIKQLAIRFQLSPRVYMTAMVIFHRFFLTHSFKDLS

RLNFAAACLFIGGKIEEQPKRMQDFLPVVHEMKQRARKLAPSPLSPNGYARLRFILQGCERAVLQTIDFE

LSYDHPLEPLLQYAKTEIAAALLELASRKSSVRLTYPAPAAPATPNKSAKPSSASSSTAPPPAPSSSSSS

SNAAPAASTRGSEERPWFEHALPHKTDFKFVDEIIETLDELYRPKAPAGGSGANSSEVSTPKESALSNST

TPIADDGTSPTKRARLAPHSAQQRHVQLQQQQQQQQQQQQQQQQQQQQGARQAPSASTSTTVQATVTTTT

STQRA

>Sro-gi|326437823| gb|EGD83393.1| hypothetical protein PTSG_12114 [Salpingoeca sp. ATCC 50818]|PTSG_12114.1

MICSLPRYDLQNTPSRAHGVSEELETRLRVAGCEIIQSAGILLNCNQVVMACAQILYQRFYYRQSFATQR

FEVTAMGCLFLASKVEEEQQRLRILMNVCRHVLFTMSKNYEPGQLVEPLELGGDAYHNLKHRVIKAERLV

LKELGFCVHLDHPHKLIISMQSVLSLEDNEALAQRAWNYMNDGLRTTVFVRYTTATIACACLDLACTDVG

ISLPDQWYELFDASESHVAHARNTIRALYQMGPVVLDDIVSSISNVADACGSSAYRRMPALQLRAVGDTS

SVSTGASVSRRGSPATGTPSATHTTPDLTPATSDTATAAATATRAGASESASAPVSSARPALITQPPMAP

PQPGLMGFAPAPSMGAPAAPAAPAAPAAFGGPINTAPPSRLRVARGPPPSSSSQQQQQQHAAHSRPSSLD

RERDRLRDAERRRGGEEDRYRDDYRSRSRHDRRRGAGRR
