# Supplementary figures and images for: Phylogenetic analysis of CDK and cyclin proteins in premetazoan lineages
Source: BMC Evol Biol. 2014 Jan 17;14:10. doi: 10.1186/1471-2148-14-10 (PMC3923393; doi:10.1186/1471-2148-14-10)

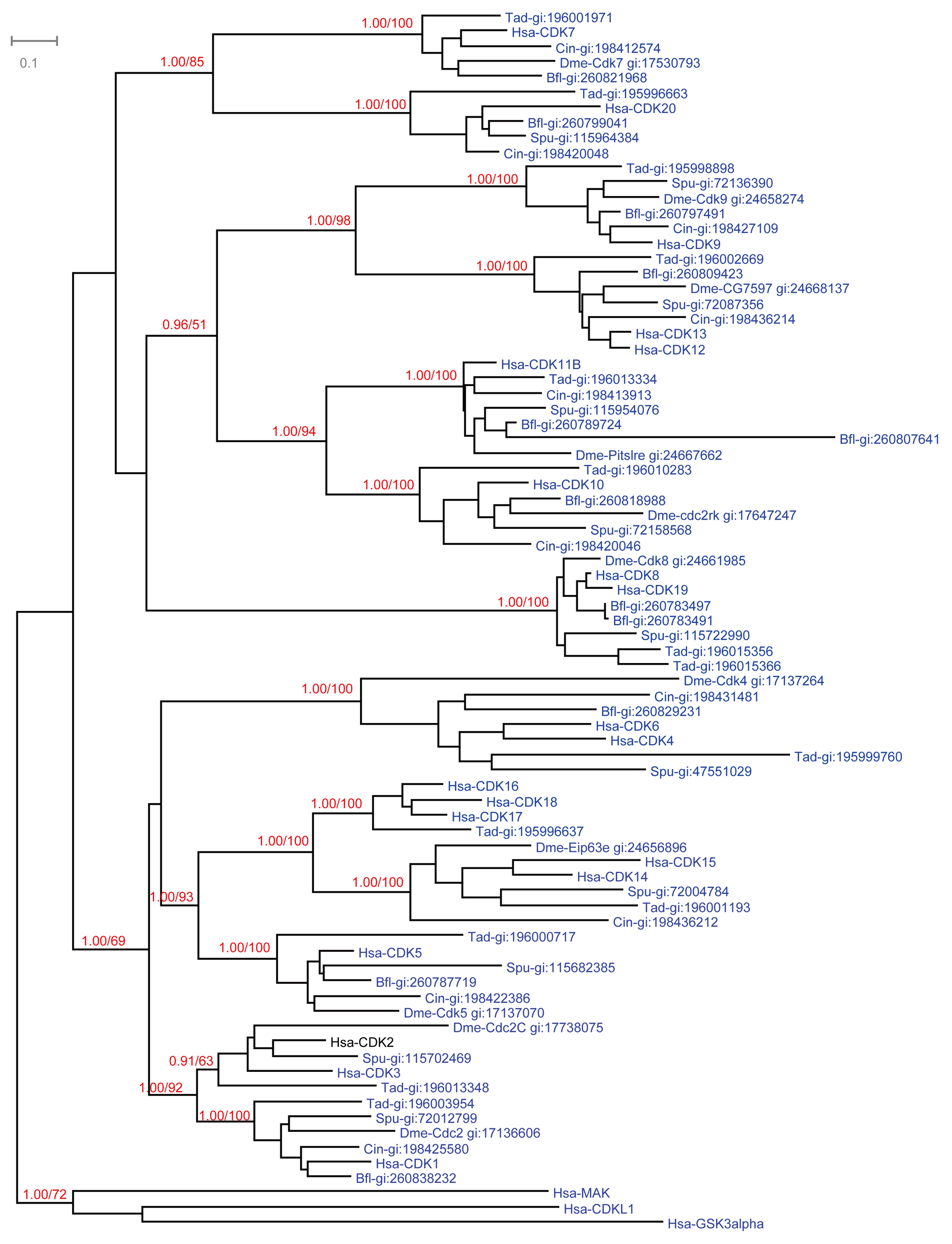

Supplement: Additional file 4: Figure S1 — Phylogenetic analysis of CDK family proteins in H. sapiens, T. adhaerens, C. intestinalis, B. floridae, S. purpuratus and D. melanogaster. Maximum likelihood analysis was conducted using RAxML program, and Bayesian analysis was carried out using PHYLOBAYES 3.3. Both methods produced trees with nearly identical topologies. The first numbers above branches indicate Bayesian posterior probabilities (only these key branches are labeled), and the second numbers above branches indicate ML bootstrap percentages. The scale bar shows the number of substitutions per site. The sequences of Hsa-GSK3alpha, Hsa-MAK, and Hsa-HCDKL1 were used as outgroup. All proteins are labeled with their accession numbers and their specie name as prefix. Abbreviations: Hsa: H. sapiens; Tad: T. adhaerens; Cin: C. intestinalis; Bfl: B. floridae; Spu: S. purpuratus; Dme: D. melanogaster. [file 1471-2148-14-10-S4.tiff]

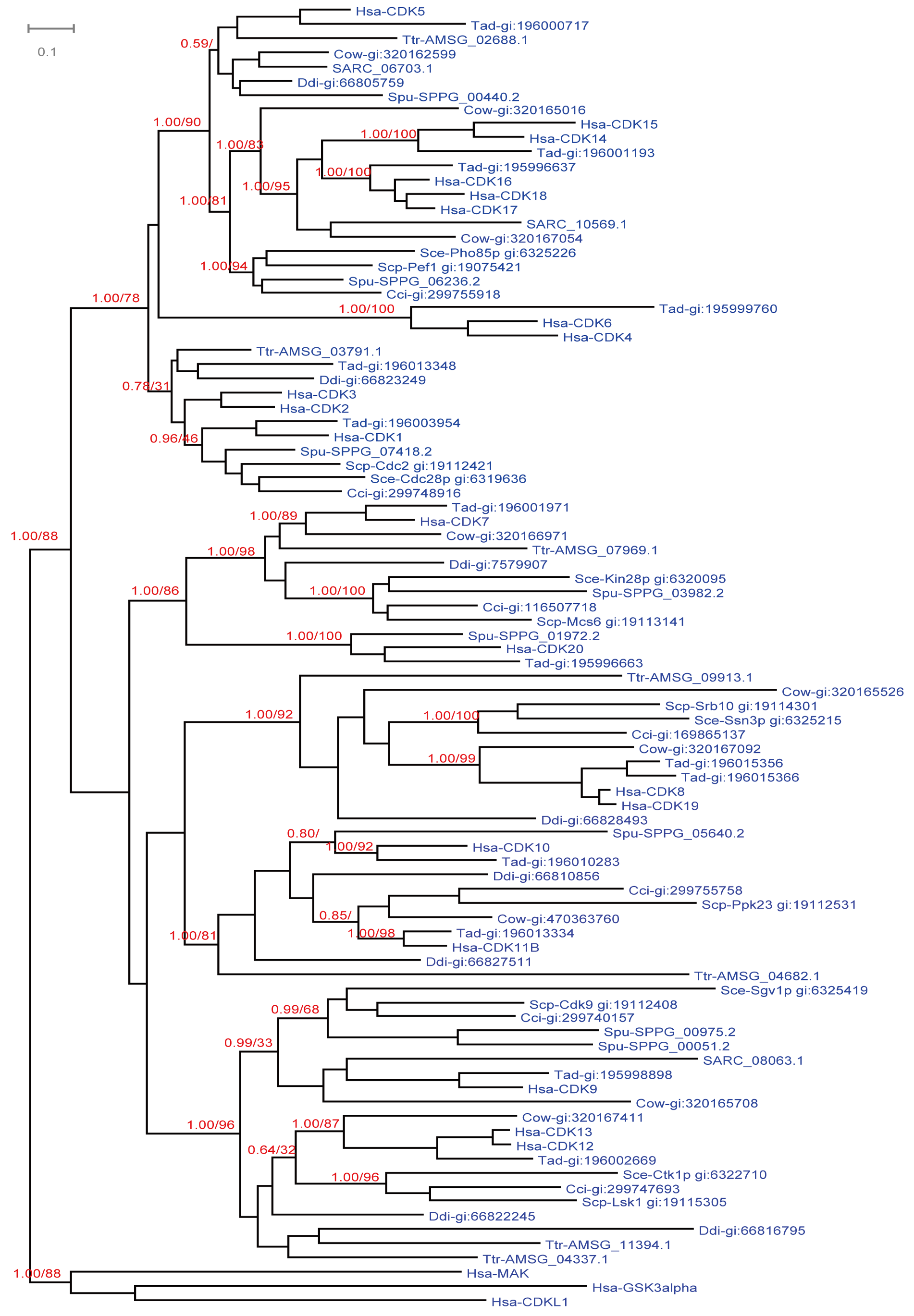

Supplement: Additional file 5: Figure S2 — Phylogenetic analysis of CDK family proteins in H. sapiens, T. adhaerens, C.owczarzaki , S. arctica, S.cerevisiae, S.pombe, C. cinerea, S. punctatus, T. trahens and D. discoideum. Maximum likelihood analysis was conducted using RAxML program, and Bayesian analysis was carried out using PHYLOBAYES 3.3. Both methods produced trees with nearly identical topologies. The first numbers above branches indicate Bayesian posterior probabilities (only these key branches are labeled), and the second numbers above branches indicate ML bootstrap percentages. The scale bar shows the number of substitutions per site. The sequences of Hsa-GSK3alpha, Hsa-MAK, and Hsa-HCDKL1 were used as outgroup. All proteins are labeled with their accession numbers and their specie name as prefix. Abbreviations: Hsa: H. sapiens; Tad: T. adhaerens; Cow: C.owczarzaki; Sar: S. arctica; Sce:S.cerevisiae; Spo:S.pombe; Cci:C. cinerea; Spu:S. punctatus; Ttr:T. trahens; Ddi:D. discoideum. [file 1471-2148-14-10-S5.tiff]

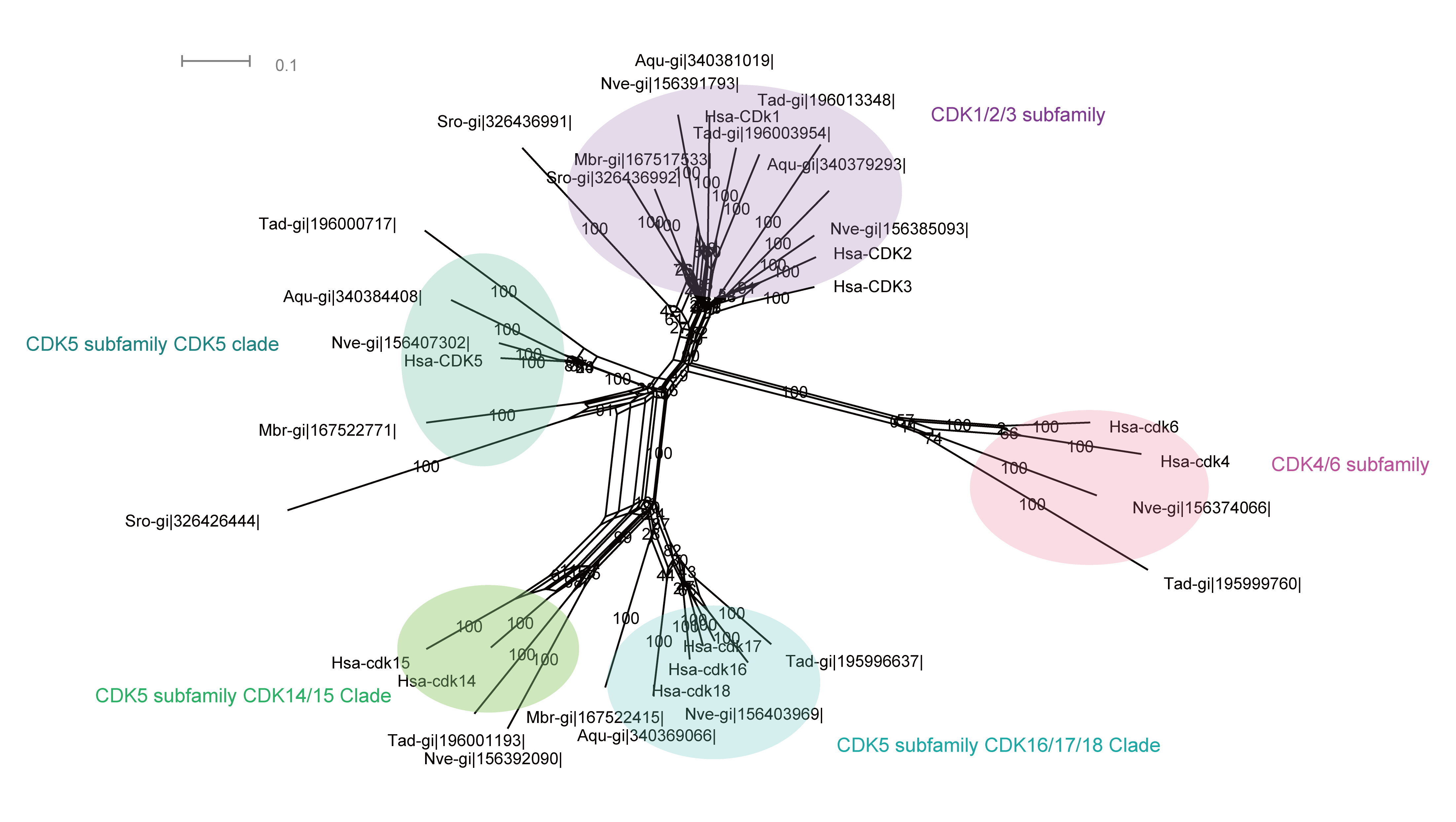

Supplement: Additional file 7: Figure S3 — Phylogenetic network analysis for CDK4/6, CDK1/2/3, and CDK5 subfamily proteins from H. sapiens, N. vectensis, T. adhaerens, A. queenslandica, M. brevicollis, and S. rosetta. Neighbor-Net analysis was conducted using SplitsTree v.4 program [56] with 100 bootstrap resamplings. All proteins are labeled with their accession numbers preceded by their species names. Species abbreviations are as follows: Hsa, H. sapiens; Nve, N. vectensis; Tad, T. adhaerens; Aqe, A. queenslandica; MBr, M. brevicollis. The alignment used for this analysis is found in Additional file 1: File S3. [file 1471-2148-14-10-S7.tiff]

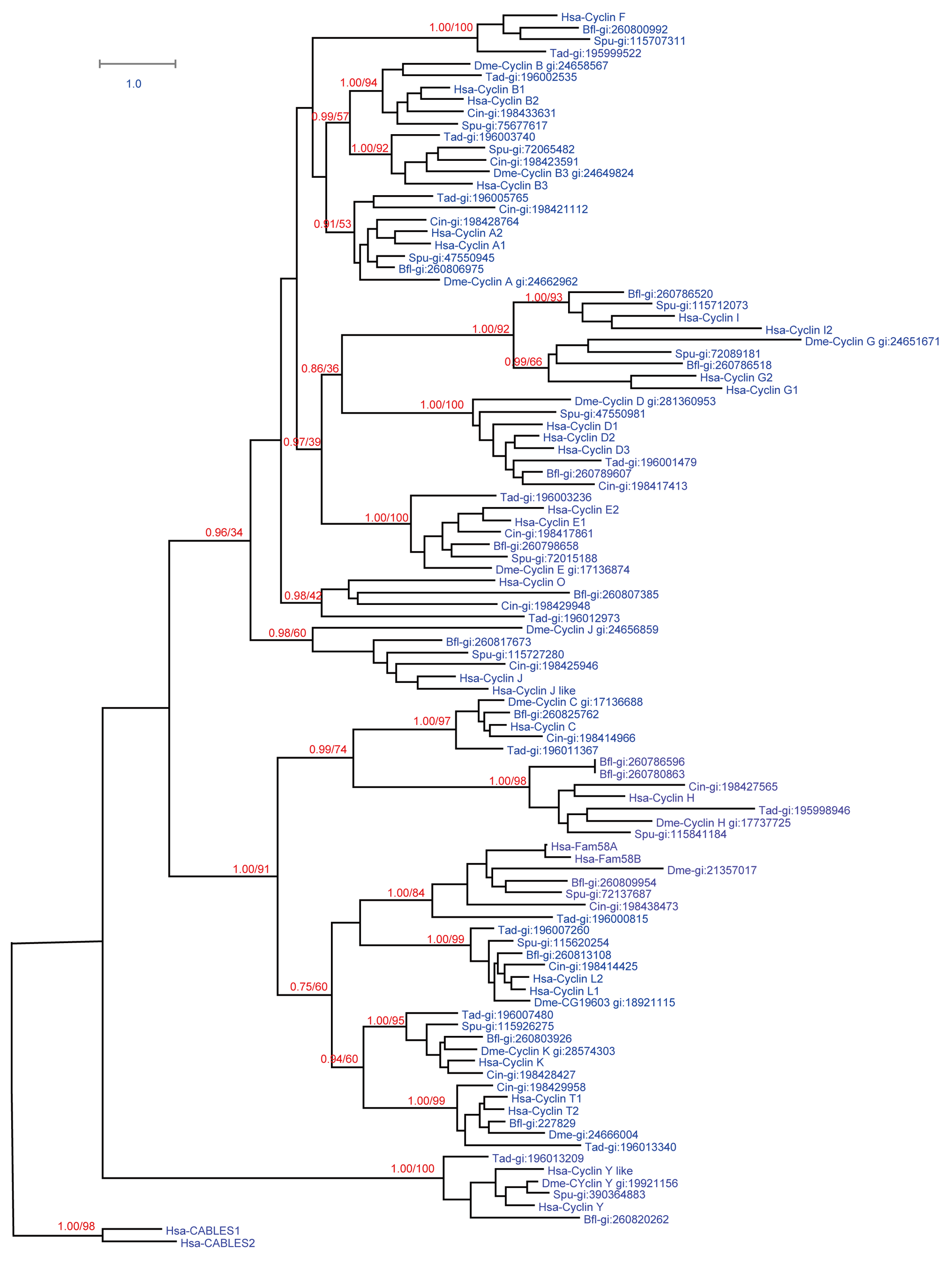

Supplement: Additional file 8: Figure S4 — Phylogenetic analysis of cyclin family proteins in H. sapiens, T. adhaerens, C. intestinalis, B. floridae, S. purpuratus and D. melanogaster. Maximum likelihood analysis was conducted using RAxML program, and Bayesian analyses were carried out using PHYLOBAYES 3.3. Both methods produced trees with nearly identical topologies. The first numbers above branches indicate Bayesian posterior probabilities (only these key branches are labeled), and the second numbers above branches indicate ML bootstrap percentages. The scale bar shows the number of substitutions per site. The sequences of Hsa-Cables1 and Hsa-Cables2 were used as the outgroup. All proteins are labeled with their accession numbers and their specie name as prefix. Abbreviations: Hsa: H. sapiens; Tad: T. adhaerens; Cin: C. intestinalis; Bfl: B. floridae; Spu: S. purpuratus; Dme: D. melanogaster. [file 1471-2148-14-10-S8.tiff]

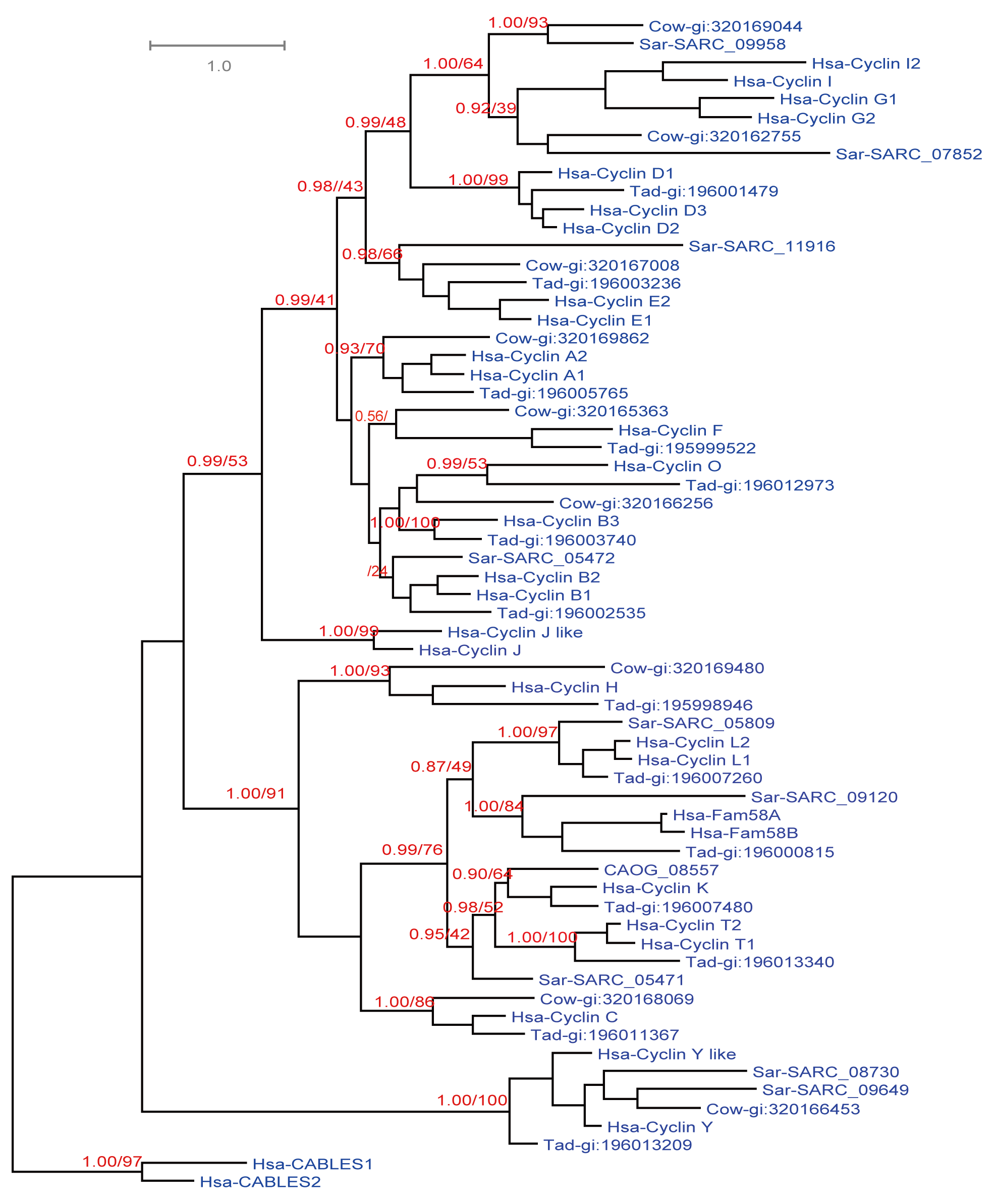

Supplement: Additional file 9: Figure S5 — Phylogenetic analysis of cyclin family proteins in H. sapiens, T. adhaerens, C.owczarzaki , and S. arctica. Maximum likelihood analysis was conducted using RAxML program, and Bayesian analyses were carried out using PHYLOBAYES 3.3. Both methods produced trees with nearly identical topologies. The first numbers above branches indicate Bayesian posterior probabilities (only these key branches are labeled), and the second numbers above branches indicate ML bootstrap percentages. The scale bar shows the number of substitutions per site. The sequences of Hsa-Cables1 and Hsa-Cables2 were used as the outgroup. All proteins are labeled with their accession numbers and their specie name as prefix. Abbreviations: Hsa: H. sapiens; Tad: T. adhaerens; Cow: C.owczarzaki; Sar: S. arctica. [file 1471-2148-14-10-S9.tiff]

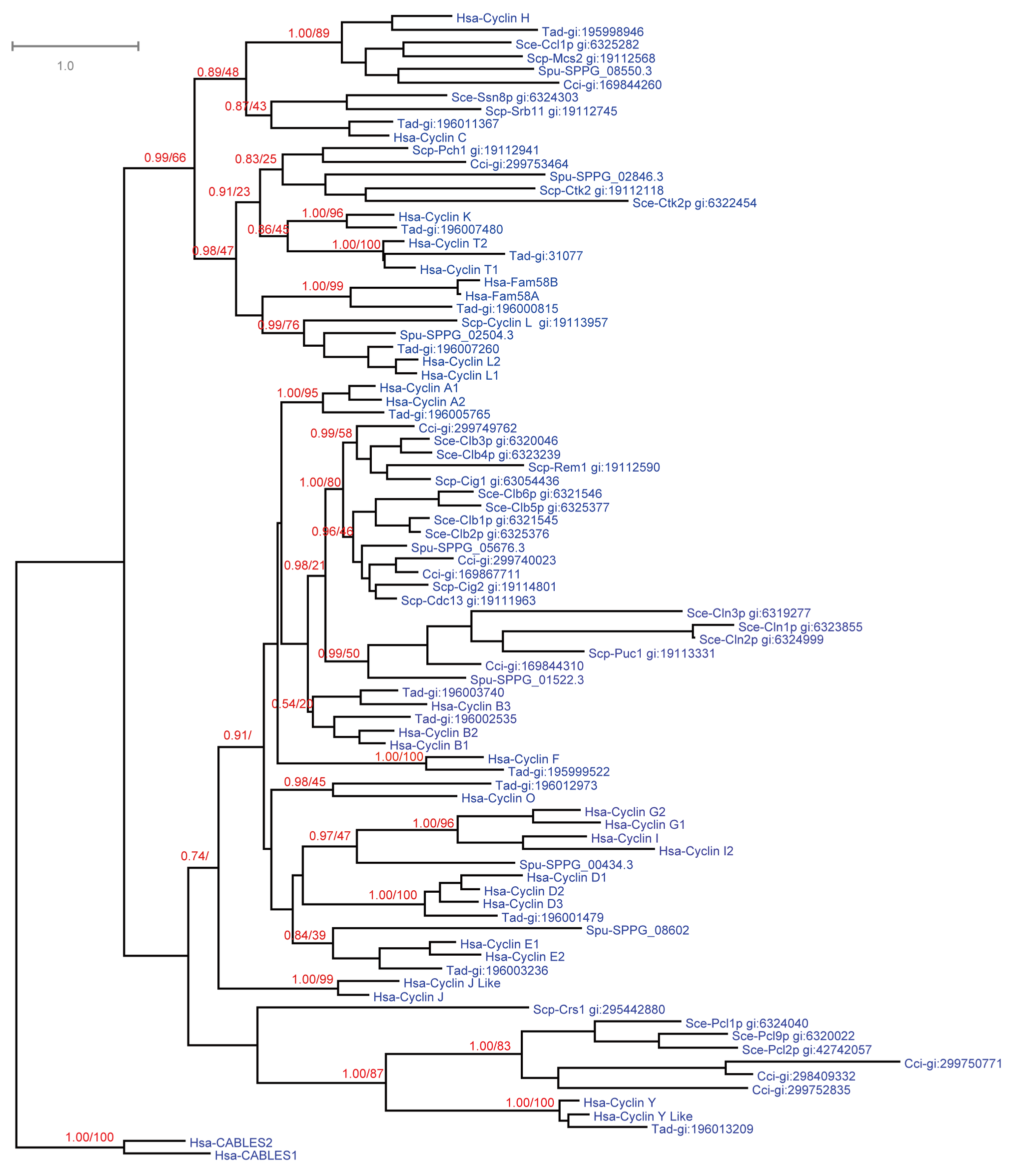

Supplement: Additional file 10: Figure S6 — Phylogenetic analysis of cyclin family proteins in H. sapiens, T. adhaerens, S.cerevisiae, S.pombe, C. cinerea, and punctatus. Maximum likelihood analysis was conducted using RAxML program, and Bayesian analyses were carried out using PHYLOBAYES 3.3. Both methods produced trees with nearly identical topologies. The first numbers above branches indicate Bayesian posterior probabilities (only these key branches are labeled), and the second numbers above branches indicate ML bootstrap percentages. The scale bar shows the number of substitutions per site. The sequences of Hsa-Cables1 and Hsa-Cables2 were used as the outgroup. All proteins are labeled with their accession numbers and their specie name as prefix. Abbreviations: Hsa: H. sapiens; Tad: T. adhaerens; Sce: S.cerevisiae; Spo: S.pombe; Cci: C. cinerea; Spu: S. punctatus. [file 1471-2148-14-10-S10.tiff]

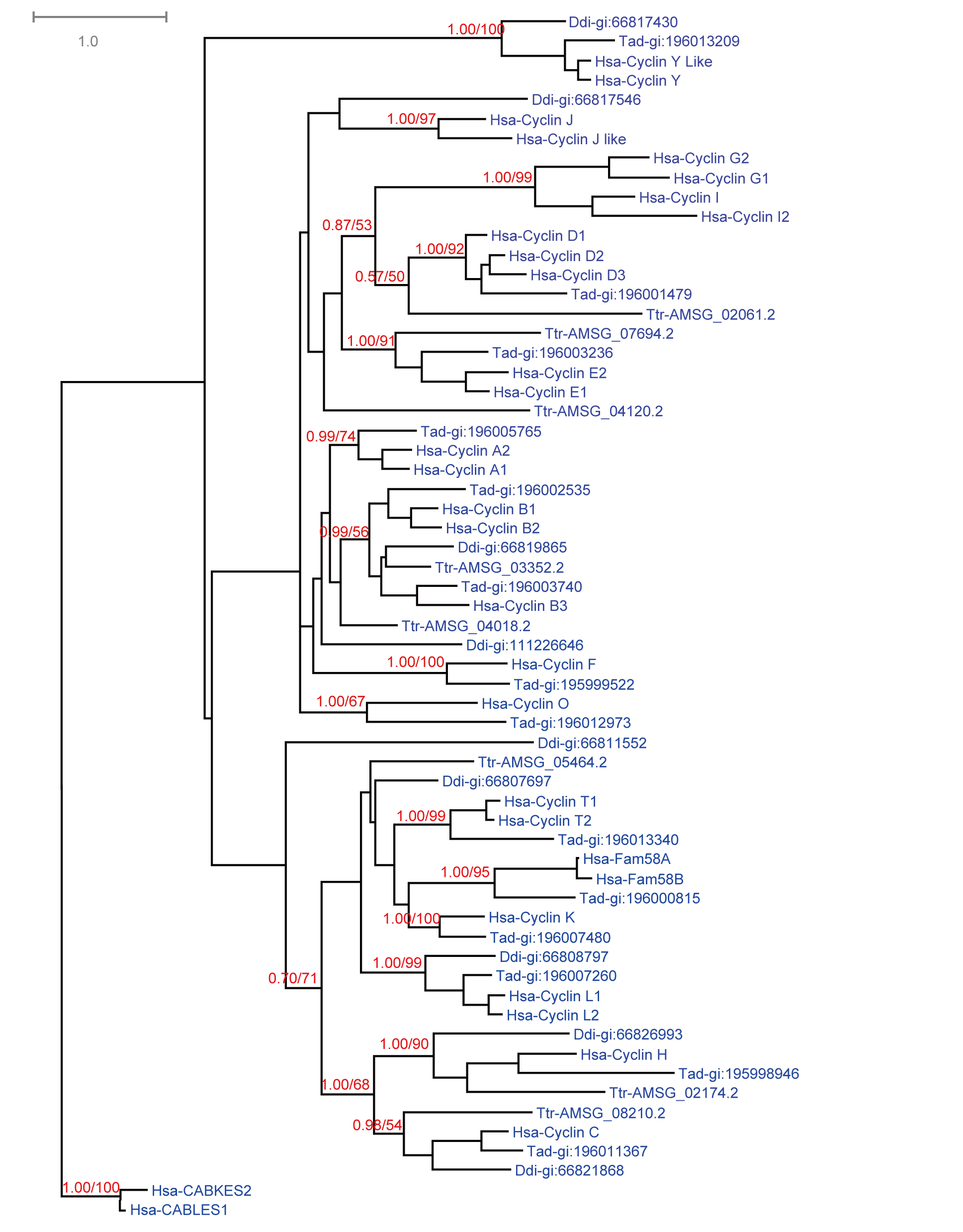

Supplement: Additional file 11: Figure S7 — Phylogenetic analysis of cyclin family proteins in H. sapiens, T. adhaerens, T. trahens and D. discoideum. Maximum likelihood analysis was conducted using RAxML program, and Bayesian analyses were carried out using PHYLOBAYES 3.3. Both methods produced trees with nearly identical topologies. The first numbers above branches indicate Bayesian posterior probabilities (only these key branches are labeled), and the second numbers above branches indicate ML bootstrap percentages. The scale bar shows the number of substitutions per site. The sequences of Hsa-Cables1 and Hsa-Cables2 were used as the outgroup. All proteins are labeled with their accession numbers and their specie name as prefix. Abbreviations: Hsa: H. sapiens; Tad: T. adhaerens; Ttr:T. trahens; Ddi:D. discoideum. [file 1471-2148-14-10-S11.tiff]

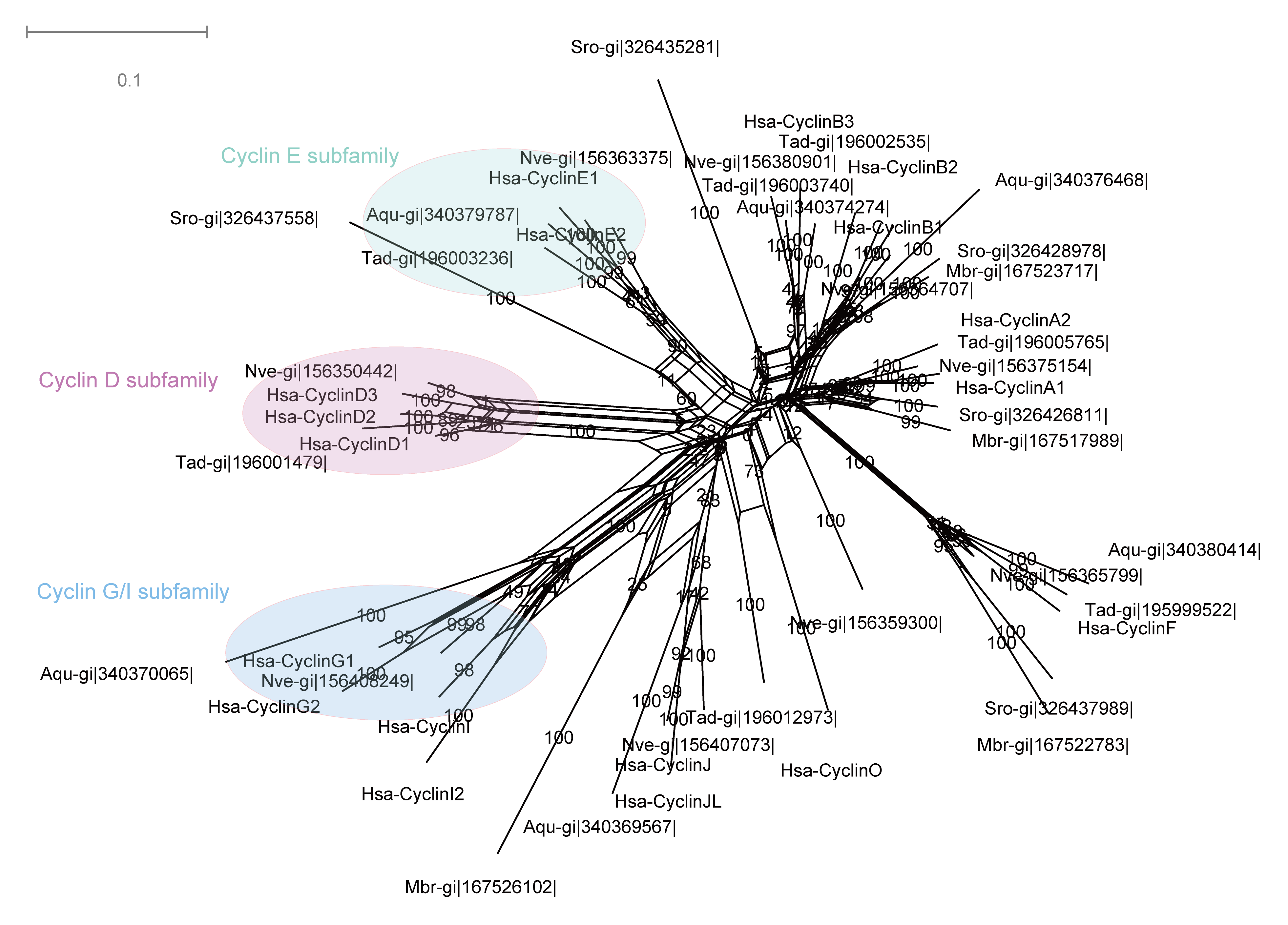

Supplement: Additional file 13: Figure S8 — phylogenetic network analysis for Cyclin B like group proteins from H. sapiens, N. vectensis, T. adhaerens, A. queenslandica, M. brevicollis, and S. rosetta. Neighbor-Net analysis was conducted using SplitsTree v.4 program [56] with 100 bootstrap resamplings. All proteins are labeled with their accession numbers preceded by their species names. Species abbreviations are as follows: Hsa, H. sapiens; Nve, N. vectensis; Tad, T. adhaerens; Aqe, A. queenslandica; MBr, M. brevicollis. The alignment used for this analysis is found in Additional file 1: File S3. [file 1471-2148-14-10-S13.tiff]
